# Structural Color from Cellulose Nanocrystals or Chitin Nanocrystals: Self-Assembly, Optics, and Applications

**DOI:** 10.1021/acs.chemrev.2c00836

**Published:** 2023-11-27

**Authors:** Bruno Frka-Petesic, Thomas G. Parton, Camila Honorato-Rios, Aurimas Narkevicius, Kevin Ballu, Qingchen Shen, Zihao Lu, Yu Ogawa, Johannes S. Haataja, Benjamin E. Droguet, Richard M. Parker, Silvia Vignolini

**Affiliations:** † Yusuf Hamied Department of Chemistry, University of Cambridge, Lensfield Road, Cambridge CB2 1EW, United Kingdom; ‡ International Institute for Sustainability with Knotted Chiral Meta Matter (WPI-SKCM^2^), Hiroshima University, 1-3-1 Kagamiyama, Higashi-Hiroshima, Hiroshima 739-8526, Japan; § Department of Sustainable and Bio-inspired Materials, Max Planck Institute of Colloids and Interfaces, Am Mühlenberg 1, 14476 Potsdam, Germany; ⊥ B CUBE − Center for Molecular Bioengineering, Technische Universität Dresden, 01307 Dresden, Germany; ¶ CERMAV-CNRS, CS40700, 38041 Grenoble cedex 9, France; # Department of Applied Physics, Aalto University School of Science, P.O. Box 15100, Aalto, Espoo FI-00076, Finland

## Abstract

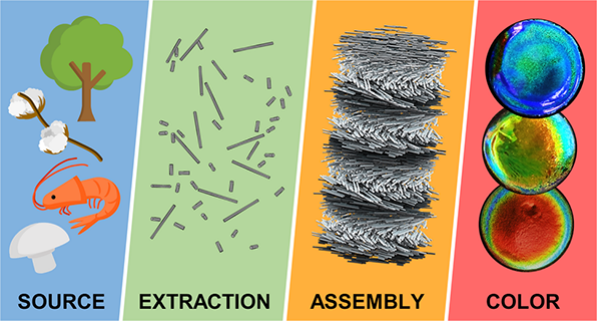

Widespread concerns over the impact of human activity on the environment have resulted in a desire to replace artificial functional materials with naturally derived alternatives. As such, polysaccharides are drawing increasing attention due to offering a renewable, biodegradable, and biocompatible feedstock for functional nanomaterials. In particular, nanocrystals of cellulose and chitin have emerged as versatile and sustainable building blocks for diverse applications, ranging from mechanical reinforcement to structural coloration. Much of this interest arises from the tendency of these colloidally stable nanoparticles to self-organize in water into a lyotropic cholesteric liquid crystal, which can be readily manipulated in terms of its periodicity, structure, and geometry. Importantly, this helicoidal ordering can be retained into the solid-state, offering an accessible route to complex nanostructured films, coatings, and particles. In this review, the process of forming iridescent, structurally colored films from suspensions of cellulose nanocrystals (CNCs) is summarized and the mechanisms underlying the chemical and physical phenomena at each stage in the process explored. Analogy is then drawn with chitin nanocrystals (ChNCs), allowing for key differences to be critically assessed and strategies toward structural coloration to be presented. Importantly, the progress toward translating this technology from academia to industry is summarized, with unresolved scientific and technical questions put forward as challenges to the community.

## 1 Introduction

### 1.1 Bio-Inspired and Sustainable Photonic Materials

Polysaccharides make up a class of biopolymers that are widely exploited in living organisms for a diversity of applications, ranging from structural integrity to energy storage. Among the numerous types of polysaccharides found in the natural world, cellulose and chitin are the most abundant. Cellulose is widely found in plants, while chitin is found in both fungi and animals (predominantly arthropods), where they are typically provide mechanical support and structure. In such systems, these polysaccharides are often organized into semicrystalline nanoscale fibrils, which can be combined or assembled in numerous ways to form hierarchical organizations that expand their range of functionalities.

One of the most fascinating applications of such hierarchical architectures is structural coloration, which can be produced by the helicoidal ordering of cellulose within the pericarp of some fruits, or by chitin within the cuticles of insects.^1,2^ The color-selective reflection from these tissues arises from the periodic ordering of the fibrils that comprise the underlying nanoarchitecture. When the periodicity (or “pitch”) is comparable to the wavelength of visible light, constructive interference occurs from these otherwise transparent materials, resulting in a strong reflection over a narrow range of wavelengths. Importantly, the intensity of the coloration from such nanostructures is generally an order of magnitude larger than the reflection obtained from absorption-based pigments, which is why it is often used to generate the most vivid appearances in nature. Moreover, due to the inherent chirality of a helicoidal structure, this reflection is circularly polarized, allowing for interesting optical effects. Lastly, because the particular hue reflected is dependent on the precise dimensions of the periodic structure, rather than the specific absorption bands of a dye molecule, it can be tuned across the entire visible spectrum and does not photobleach.^3−7^

Crystalline nanoparticles of cellulose and chitin can be extracted from natural sources by acid hydrolysis. Interestingly, it was discovered that aqueous colloidal suspensions of these “nanocrystals” can spontaneously self-organize on the nanoscale to mimic this natural helicoidal architecture. Upon drying, this ordering can be retained into the solid-state, enabling the specific reflection of visible light. Using this self-assembly approach, colors from across the entire visible spectrum can be produced and combined with striking visual effects, such as iridescence or a metallic shine. While there are also top-down ways to construct analogous helicoidal architectures (e.g., using layer-by-layer deposition^8^), the advantage of the spontaneous self-assembly route is the simplicity of fabrication, translating into better scalability and lower fabrication costs.

When taking bioinspiration as a design principle and seeking to employ the same building blocks that nature uses,^9^ it is thus clear that cellulose and chitin nanomaterials are strong candidates to develop sustainable colorants for a wide range of sectors, from packaging to cosmetics and food.^1^ Cellulose and chitin are renewable resources that can be extracted from a wide range of biological sources, including agricultural and industrial byproducts.^10,23,99^ When processed into nanomaterials, they retain the biocompatibility and biodegradability of the source and therefore represent a sustainable alternatives to reduce carbon dioxide emissions when compared to traditional materials.^11−13^ In the specific example of coloration, cellulose and chitin nanomaterials have the potential to replace current inorganic pigments based on mica or titania, which respectively suffer from energy extensive (and unethical) extraction processes, and health concerns leading to classification as a category two carcinogen by inhalation by the EU in 2020.^14,15^ While the benefits of these materials do not translate yet in the life cycle assessments available in the public domain that are otherwise carried on limited, pilot-scale production, other environmental and socio-economic impact assessments support the wide adoption of these materials when compared to synthetic analogues.^16^ As such there is enormous potential for sustainable polysaccharide-based nanomaterials to disrupt the colorant industry.

### 1.2 Cholesteric Self-Assembly in a Nutshell

**Figure 1:**
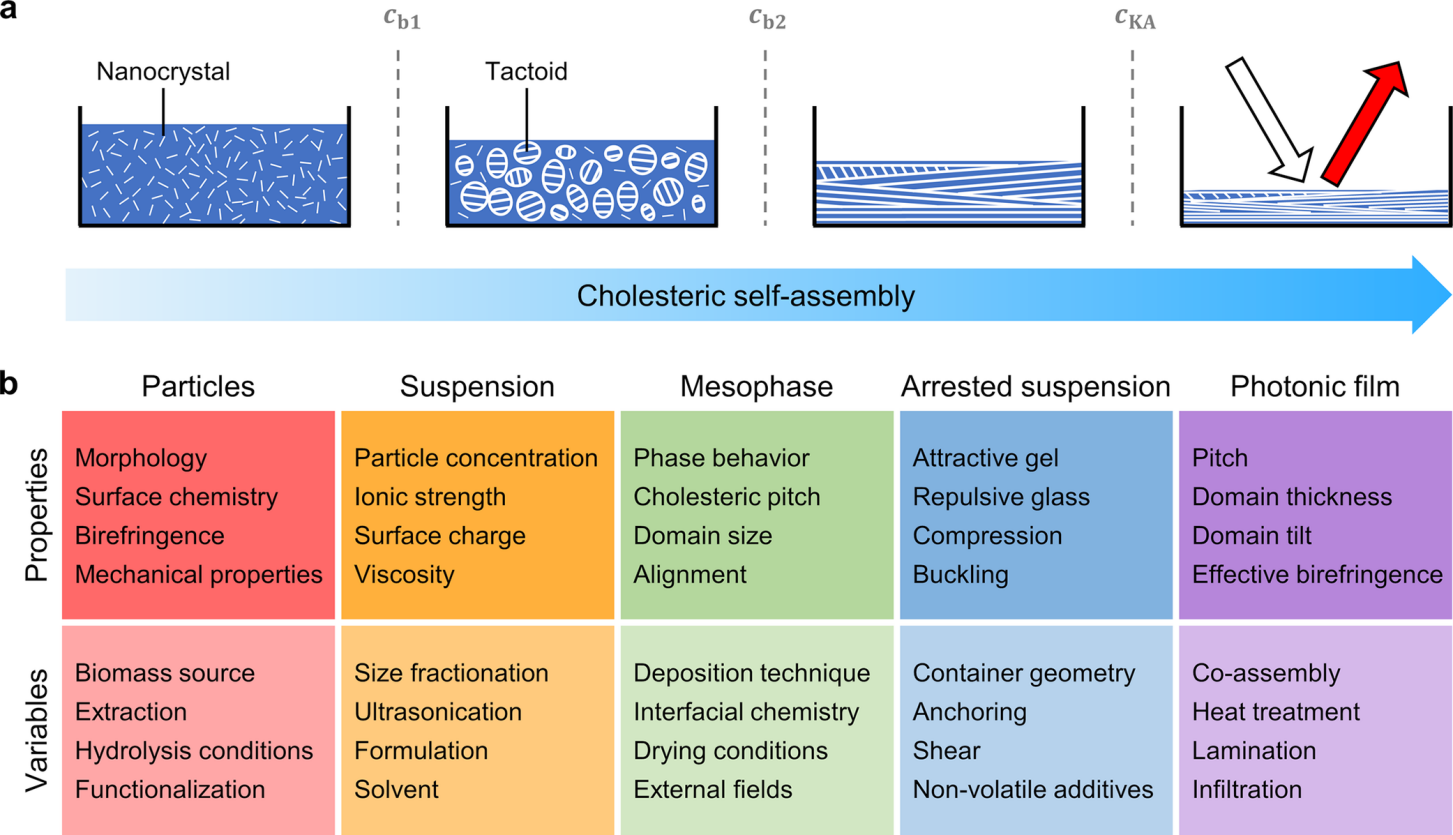
(a) Schematic of the cholesteric self-assembly process. The concentrations *c*_b1_, *c*_b2_, and *c*_KA_ correspond, respectively, to the isotropic-biphasic transition, the biphasic-cholesteric transition, and the onset of kinetic arrest. (b) Summary of key physical properties at each stage of the self-assembly process and the associated experimental parameters can be used to determine them.

Cellulose nanocrystals (CNCs) (and chitin nanocrystals (ChNCs)) are elongated, birefringent nanoparticles that, due to their high aspect ratio, behave as a colloidal liquid crystal when dispersed in water. In this regard, CNCs lie at the intersection of two distinct classes of soft matter systems, combining properties of colloidal particles (e.g., surface charge) and liquid crystals (e.g., lyotropic behavior). In this review, we describe how these properties can be exploited to produce solid films with structural color via “cholesteric self-assembly”. However, this process is complex, with multiple parameters that can influence the process in a multitude of ways and at different stages. To help orient the reader, we first provide a brief overview of this process, as illustrated in Figure 1a.

A dilute CNC suspension initially exists as an isotropic phase in which the orientations of the CNCs are random and uncorrelated with each other. The suspension must be both colloidally stable (so that CNCs are not aggregating over time) and fluid (so that the CNCs can move freely). If the solvent is allowed to evaporate, the CNC concentration increases and the average interparticle distance decreases, leading to stronger interaction between CNCs. Above a threshold concentration, CNCs spontaneously organize into a cholesteric liquid crystal, often synonymously referred to as a chiral nematic phase. In this phase, the individual nanoparticles are locally aligned along a common direction that spatially rotates to describe a left-handed helicoid with a defined periodicity known as the pitch *p*. This process occurs by first forming small cholesteric droplets, termed “tactoids”, which increase in size and number with increasing concentration. These tactoids are at a higher concentration than the surrounding isotropic phase and thus will also sediment and coalesce over time into a continuous cholesteric phase. As the suspension dries further, the pitch of this cholesteric phase decreases as the CNCs are brought into ever closer proximity, which enhances their chiral interaction. However, the pitch in suspension is always in the micron range (in contrast to molecular or polymer cholesterics, which can have submicron pitches in the liquid phase). In parallel, the viscosity of the suspension increases due to a combination of increasing particle concentration and ionic strength (from free electrolytes and counterions). At a certain point in time, the suspension becomes too viscous for the CNCs to thermodynamically relax and the system thus becomes kinetically trapped. Beyond this point of kinetic arrest, any further changes to the pitch arise primarily from geometric distortions upon loss of solvent. For the common case of a suspension cast in a dish, drying induces a vertical compression of the arrested structure, resulting in a corresponding reduction of the pitch. If the final pitch in the produced dry film is on the order of the wavelength of visible light (i.e., *p* ≈ 250–450 nm), then selective reflection can occur from this periodic birefringent structure, resulting in visible coloration.

The process described above has often been referred to as “evaporation-induced self-assembly” or “EISA”.^17^ However, the principal driving force in this process is the increase in CNC concentration, and while solvent loss is often the underlying mechanism, evaporation is not a requirement. In contrast, EISA is more appropriate when referring to particle assemblies specifically arising from nonequilibrium processes during evaporation, which are typically driven by capillary forces.^18^ We therefore discourage the use of the term EISA in the case of CNC suspensions. As an alternative, we recommend the term “(cholesteric) self-assembly” when referring to the process above in its entirety, and the term “self-organization” when describing the liquid crystalline behavior alone.

### 1.3 Scope and Outline of the Review

Since the first report of liquid crystalline gels from cellulose nanocrystals (CNCs) in 1959,^19^ and of their cholesteric phases in 1992,^20^ followed by the discovery of their photonic behavior in solid state films in the following years,^21^ the field has massively expanded. In contrast, while the liquid crystalline behavior of chitin nanocrystals (ChNCs) has also been known for decades,^22^ their application in photonic materials is a relatively new field of research, with the significant advances needed to unlock visible coloration only reported within the past decade.

In this review, we provide a comprehensive overview of the state-of-the-art for photonic materials self-assembled from CNCs and ChNCs. As illustrated schematically in Figure 1b, this encompasses their production and self-assembly into helicoidal films, the resulting complex optical response of these materials, and strategies to translate this burgeoning biosourced technology toward commercial application. However, it is important to note that many of these topics are broad enough to be the subject of a review in their own right, and as such we recommend the following articles for further information on the production and characterization of CNCs,^23,24^ or their liquid crystalline properties.^18^ Similarly, the production, properties, and applications of nanochitin have recently been comprehensively reviewed.^25^ Furthermore, we consider the following topics beyond the scope of our review: (i) the assembly of nanocelluloses into highly disordered materials for enhanced whiteness via scattering;^26^ (ii) other bottom-up assemblies for nonoptical applications of nanocelluloses,^12,27−31^ most notably mechanical enhancement of composites or Pickering stabilization of emulsions; (iii) other polysaccharide nanoparticles, such as starch, which have not yet been shown to produce structural color;^32^ (iv) structurally colored polymer mesophases derived from polysaccharides, such as hydroxypropyl cellulose (HPC) and other cellulose derivatives,^33,567^ which follows a noncolloidal cholesteric self-assembly pathway; and (v) applications of fibrillated (non-nanoscale) cellulose.^27,34^

The structure of this review is as follows. We first define what CNCs are, and introduce their fundamental properties, which originate from both the source material, namely cellulose, and their extraction history (section 2). Next, we review CNC self-organization into a cholesteric suspension (section 3), and how the CNC mesophase can be further aligned at larger length scales by additional phenomena (section 4). This is complemented by a discussion of the colloidal behavior of CNCs, and how this leads to the suspension becoming kinetic arrested (section 5). The consequences of kinetic arrest are then considered in the context of subsequent drying into a solid film or particle (section 6). An overview of the fundamental optics necessary to understand the photonic response of CNC films then follows (section 7). Having provided the reader with the necessary understanding of the key stages in the CNC self-assembly process, we then summarize the wide variety of methods that have been developed to control the organization of CNCs in suspension (section 8) and influence its drying into a photonic film, coating, or particle (section 9). Lastly, routes to enhance the performance or functionality of photonic CNC films are considered, such as postprocessing or by forming composites with other materials (section 10). Although much of the physical mechanisms underlying CNC self-assembly can be transposed to ChNCs, their distinct surface chemistry can cause them to act differently in suspension. As such an overview of their individual properties and suspension behavior is compiled in the final section, which also includes strategies to overcome their much lower birefringence to achieve structural color (section 11). We then close this review by contemplating unresolved scientific questions and outstanding technical challenges that the wider community should seek to address (section 12).

## 2 Cellulose Nanocrystals (CNCs)

This section introduces the crystalline structure of native cellulose and describes how CNCs can be isolated from natural sources. We then provide an overview of the key properties of individual CNCs relevant for photonic applications, which are either inherited from native cellulose or emerge from the production process.

### 2.1 Cellulose

Cellulose, a linear homopolymer of β-(1→4) linked d-glucose residues,^35^ is the most abundant biopolymer on Earth, with over 10^12^ tonnes of biomass produced each year.^36^ It is produced by a wide variety of organisms, ranging from bacteria, amoeba and microalgae to macroalgae and marine invertebrates.^37^ However, it is most commonly associated with vascular (higher) plants, where it plays an essential role in maintaining the structural integrity of cell walls.

The biosynthesis of cellulose and the properties of the resulting fibers are comprehensively reviewed elsewhere.^38−41^ Cellulose is synthesized at the plasma membrane, where a terminal complex (TC) composed of cellulose synthase enzymes produces multiple cellulose chains in parallel.^35,42^ As cellulose chains are extruded from the TC into the extracellular space, they spontaneously organize into elongated crystalline domains known as microfibrils, which are bound together by dispersion forces and hydrogen bonds.^43−45^ At the next level in the structural hierarchy, microfibrils are frequently observed to be laterally aggregated into polycrystalline microfibril bundles.^46,47^ In the case of plants, these cellulose microfibrils are generally associated with amorphous compounds (e.g., hemicelluloses, lignin and pectin) that mediate their mechanical properties, from the very soft and elastic primary cell walls of growing plants to the thick and mechanically strong secondary cell walls found in wood.^48^

The exact number of chains that comprise an “elementary fibril” of crystalline cellulose remains elusive for higher plants, as the relatively small microfibril cross-section poses a challenge in their structural characterization. While a 36-chain microfibril was the standard model for a long time, the current understanding (based on scattering and spectroscopy analysis) is that microfibrils in higher plants contain either 18 or 24 cellulose chains.^46,49−51^ The morphological analysis of higher plant TCs also supports an 18-chain model.^52^ However, the apparent lateral dimensions of microfibrils vary between plant species: for instance, cotton and flax microfibrils have a significantly larger cross-section than microfibrils from woody plants.^53^ The origin of this variation is yet to be elucidated.

The crystallinity and morphology of cellulose fibers have long been debated, as reviewed elsewhere.^54^ Native cellulose, while having defects and dislocations in the crystal structure, contains no extensive amorphous regions.^38^ However, there is evidence of periodic disorder along the microfibril chain axis of cellulosic fibers, as observed by small-angle neutron scattering^55^ and, more recently, super-resolution microscopy.^56,57^ While this would explain the leveling-off degree of polymerization (LODP) observed for the hydrolysis of cellulose microfibrils, the origin of these periodic disorders along the microfibrils is a matter of debate.^38^

**Figure 2:**
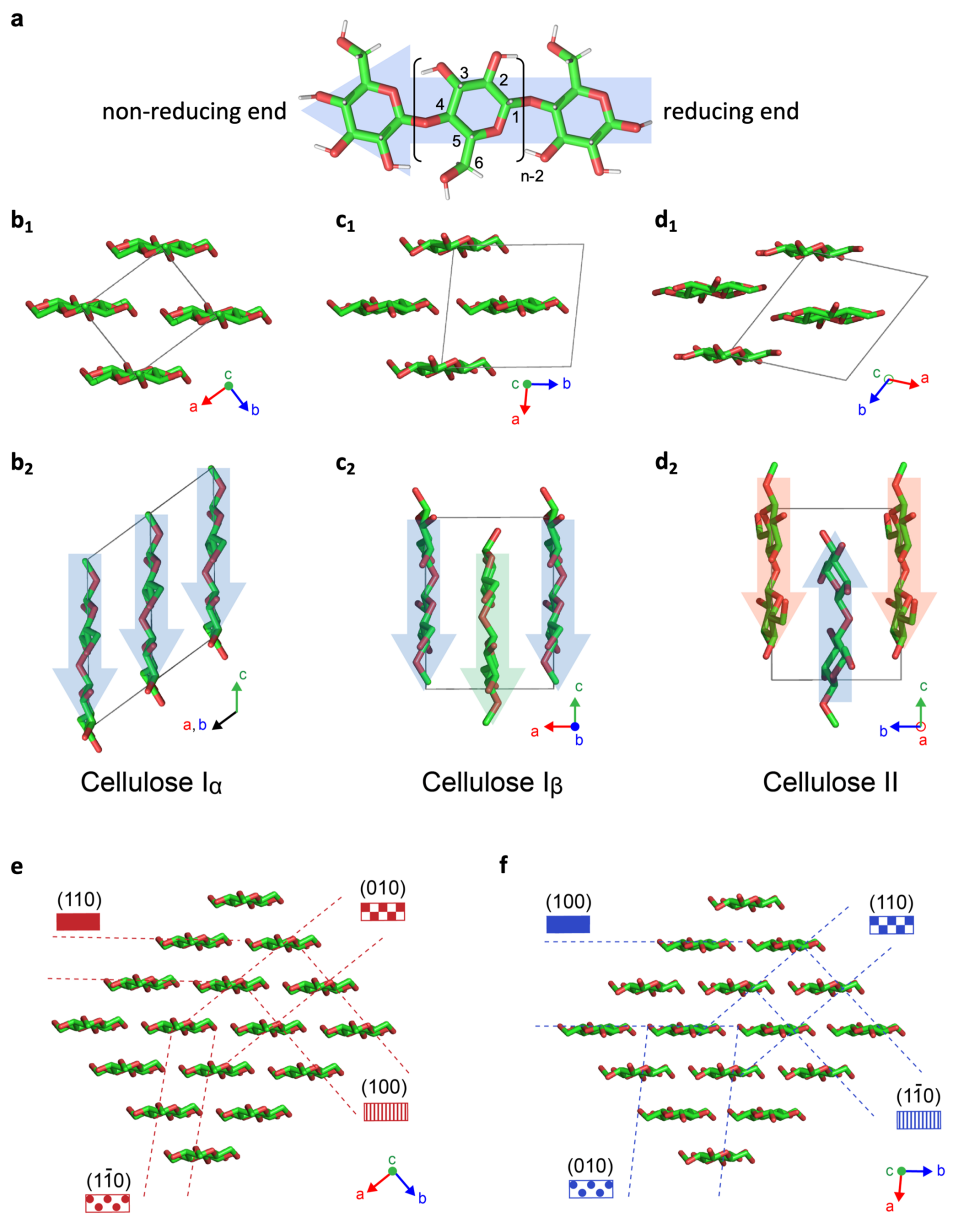
Cellulose molecular and crystallographic structure. (a) Cellulose chain, illustrated as the building block of cellulose allomorphs. (b–d) Crystal structures of (b) cellulose Iα,^70^ (c) cellulose Iβ,^70^ and (d) cellulose II,^71^ in the (b_1_, c_1_, d_1_) cross-section and (b_2_, c_2_, d_2_) side projections. Arrows indicate the molecular polarity of cellulose chains. (e, f) Cross-sectional projection providing the correspondence between crystallographic planes and the orientation of the anhydroglucose monomers (e) in cellulose Iα,^70^ and (f) in cellulose Iβ.^70^

There are several ways to laterally associate cellulose chains, which result in different crystal structures, known as allomorphs (Figure 2). In TCs, cellulose is formed by sequentially attaching glucose units to the nonreducing end of the growing chains. Consequently, native microfibrils are composed of parallel unidirectional cellulose chains, a configuration known as cellulose I. While it was initially believed that cellulose I represented a single allomorph, subsequent studies have established that native cellulose exists as a mixture of two distinct crystal structures, now known as cellulose Iα and Iβ (Figure 2a,b), with the relative amount of each allomorph varying between species.^38,39,58^ While these two allomorphs are crystallographically distinct, with cellulose Iα being triclinic and cellulose Iβ monoclinic, they both present a parallel, noncentrosymmetric alignment of the cellulose chains. Dislocations inside the native crystal structure (such as those produced by shear) can thus easily cause local coexistence of both Iα and Iβ allomorphs in various amounts.^59^ Dissolution of cellulose I followed by regeneration results in cellulose II, an allomorph with an antiparallel arrangement of chains (Figure 2d). Additional cellulose allomorphs can be produced by further physicochemical treatments of cellulose I and II: cellulose III is obtained via swelling in liquid ammonia and amines with subsequent regeneration,^60−62^ while cellulose IV is prepared using high temperature and pressure, often in the presence of glycerol.^63−65^

As an interesting side-point, the anisotropic molecular packing in the crystal structure of native cellulose microfibrils leads to crystal surfaces with preferential hydrophilic and hydrophobic interactions (see Figure 2 and Figure 4).^66^ This amphiphilicity enables the use of low-surface-charge CNCs as Pickering emulsion agents that can stabilize air–water or air–oil interfaces,^67,68^ while cellulose films of variable wettability can be made by controlling the orientation of crystal facets.^69^

### 2.2 Production of CNCs

CNCs are produced by extracting crystalline cellulose from a biological source material with the aim of creating high-aspect-ratio nanoscale fragments that retain the crystal structure of the microfibrils. This process consists of several stages, purification of the lignocellulosic source, homogenization into a cellulose feedstock, and acid hydrolysis to isolate CNCs, which are summarized below.

At this point, it is worth clarifying why it is necessary to isolate nanocellulose by a top-down process from natural cellulose, rather than produce crystalline cellulose by a bottom-up process from individual cellulose chains, analogous to the synthesis of other polymer nanoparticles. First, regenerated cellulose made by dissolution and recrystallization is usually in the form of cellulose II, as discussed above. This is undesirable for self-assembly, as cellulose II nanoparticles have a less elongated morphology and assemble much more slowly into a cholesteric phase than native cellulose particles.^72,73^ While in vitro synthesis of cellulose I has been achieved using biologically derived enzymes,^74,75^ it is not currently possible to easily tune the morphology of the resulting crystallites, and they therefore exhibit substantial polydispersity.^76^ At present, controlled degradation of native cellulose by acid hydrolysis or other methods is therefore the most effective way to produce CNCs of desired size and crystal structure.

#### 2.2.1 Nanocellulose

The term nanocellulose, or cellulose nanomaterial, is used to describe objects predominantly made of cellulose with at least one nanoscale dimension. The terminology used to describe nanocellulose has evolved over the past few decades and has only recently begun to be standardized.^77^

**Figure 3:**
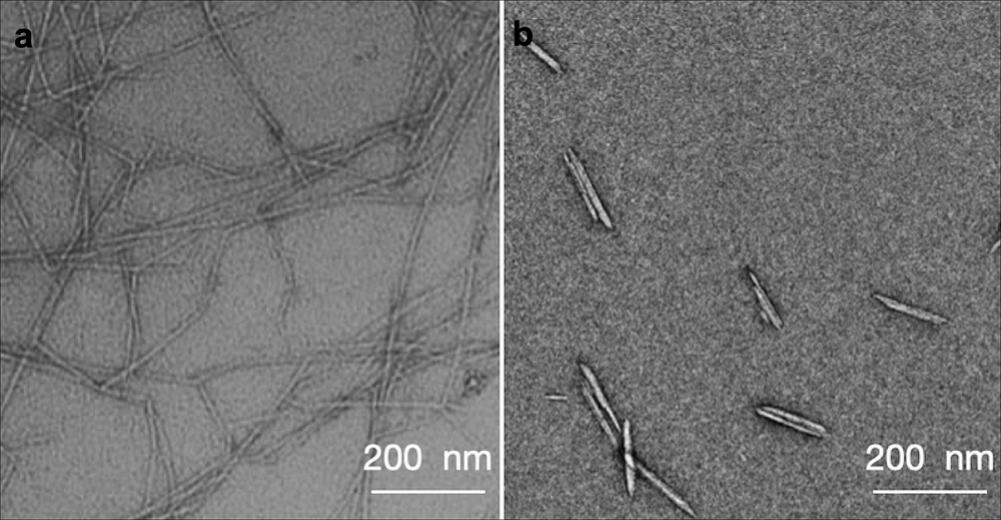
Example transmission electron micrograph (TEM) images. (a) Cellulose nanofibers (CNFs). Reproduced with permission from ref (90). Copyright 2007 American Chemical Society. (b) Cellulose nanocrystals (CNCs). Data from ref (89).

Nanocellulose can be divided into two main types, known as cellulose nanofibers (CNFs) and cellulose nanocrystals (CNCs). CNFs are long fragments of cellulose microfibrils with an aspect ratio typically above 100, as shown in Figure 3a.^78,79^ As such, CNFs are often entangled even at low volume fractions in water, where they usually form hydrogels.^80^ In contrast, CNCs (Figure 3b) are much shorter nanoparticles with a lower aspect ratio. Several terms have previously been used to describe CNCs, including nanocrystalline cellulose (NCC), cellulose (nano) whiskers, microcrystallites,^81^ or (incorrectly) micelles.^82^ However, the growing commercial relevance of nanocellulose has led to initiatives to standardize the terminology,^83−86^ which have converged on the term “CNC”. While both CNCs and CNFs have potential for a wide range of applications, the following sections will focus solely on CNCs, due to their ability to self-organize into a cholesteric phase that results in a photonic film upon drying.

What constitutes a CNC can be defined from a “top-down” perspective (focusing on colloidally stable nano-objects) or from a “bottom-up” perspective (focusing on distinct crystalline elements). At a fundamental level, CNCs are composed of short (ca. 100 nm) crystalline segments of native cellulose microfibrils, here referred to as *crystallites*. From the bottom-up perspective, these crystallites are synonymous with CNCs in general. However, many of the individual nano-objects observed using electron microscopy (Figure 3b) or atomic force microscopy have a poly crystallite (and therefore polycrystalline) composition, being formed of two or more crystallites laterally connected together to produce a “bundled” or “agglomerated” morphology. Crucially, these bundled particles are a native feature of CNC suspensions and not simply a measurement artifact.^87−89^ Consequently, from the top-down perspective, a CNC can be defined as any individual nano-object in suspension, irrespective of its composition. In this case, the term crystallite refers to both a subset of the CNC population and the building blocks from which all other CNCs are made. In this review, we adopt the top-down perspective in defining CNCs, as the functional elements of the suspension relevant for self-assembly are the composite particles, and not the fundamental crystallites.

#### 2.2.2 Cellulose Source

**Figure 4:**
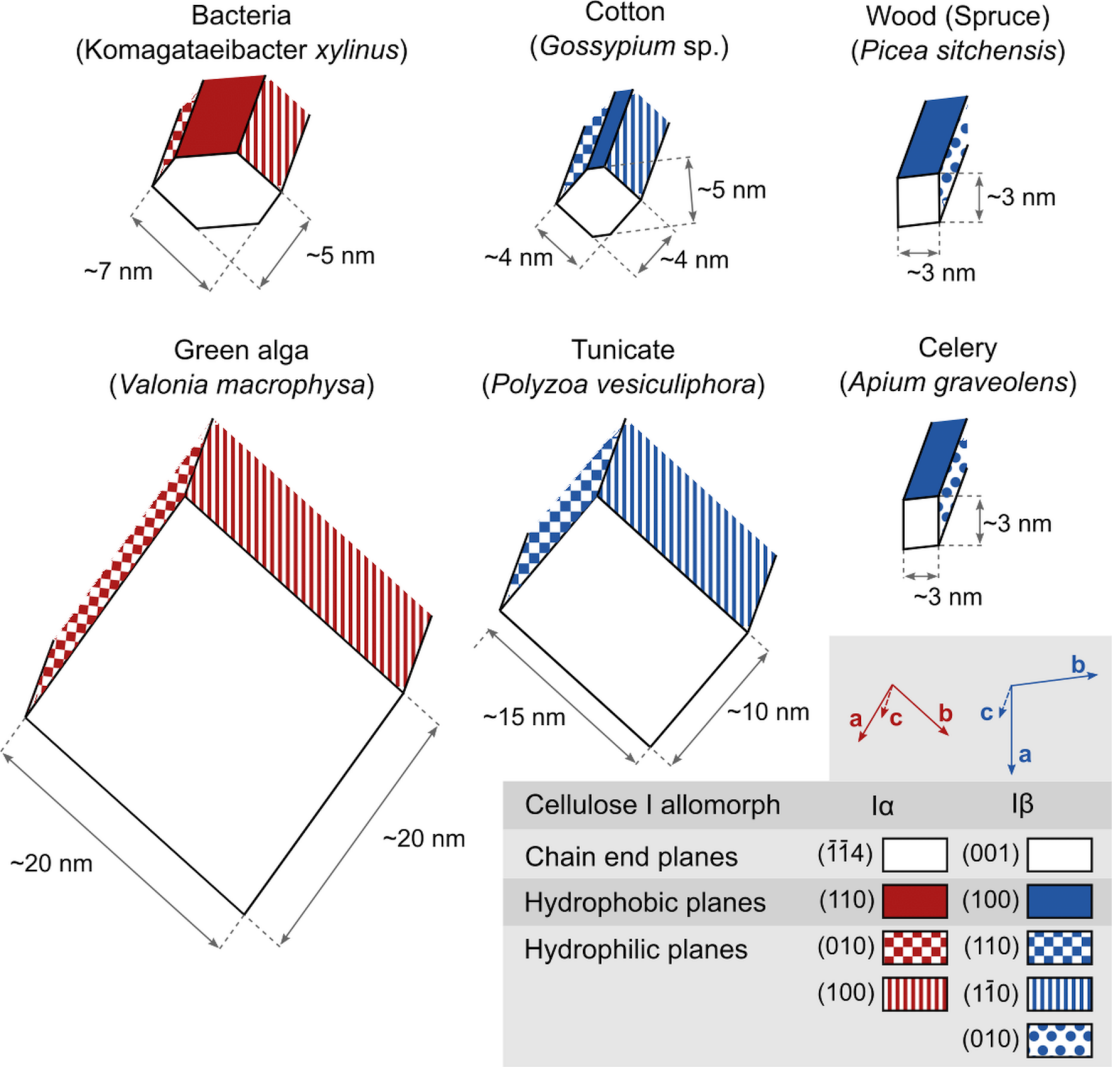
Cross-section dimensions of the microfibrils of cellulose from different origins. Values taken from Sugiyama et al. (1985),^114^ Helbert et al. (1998a, 1998b),^115,116^ Martínez-Sanz et al. (2015, 2017),^117,118^ Fernandes et al. (2011),^46^ and Thomas et al. (2012).^119^ In this view, the white facets correspond to the exposed cellulose reducing ends.

The production of CNCs has been reported from a vast range of natural cellulose sources, as summarized in previous reviews.^23,39,91,92^ Indeed, CNCs can theoretically be extracted from any organism that produces crystalline cellulose. However, the predominant crystal allomorph and microfibril cross-section vary considerably between sources (Figure 4). The length of crystalline domains within microfibrils also varies with cellulose source, which sets an upper bound on the dimensions of the crystallites that comprise the resulting CNCs (i.e., the lengths of crystallites within bundled particles).

CNCs are typically produced from lignocellulosic biomass (i.e., plants), due to the natural abundance and commercial availability of these sources.^23^ However, most of the research on CNC-based photonic films has focused on wood and cotton, with CNCs from these sources typically exhibit lengths ranging from 100 to 250 nm, and aspect ratios in the range of 5–50.^88,93−95^ There is also growing interest in valorizing other lignocellulose biomass to produce CNCs, including nonwoody plants (e.g., bamboo, sugar cane and maize),^91^ as well as bast fibers from plants such as ramie, hemp, and jute;^81,96^ however, few studies have explored the use of these feedstocks for photonic films, with sisal and sugar cane as notable exceptions.^97,98^ Alternatively, various agricultural lignocellulosic byproducts and end-of-life textiles could also potentially be used to produce CNCs for cholesteric self-assembly.^23,91,99−101^

Aside from lignocellulosic biomass, cholesteric ordering has been reported for CNCs derived from other sources, including bacteria, algae, and tunicates. CNCs isolated from these nonlignocellulosic sources tend to have larger crystallites: for instance, CNCs from bacterial cellulose typically have lengths ranging from 0.5 to 2 μm and an aspect ratio of 30–100,^102−105^ while the CNCs derived from algae have a wide range of lengths from 0.1 to 4 μm.^106−109^ Tunicates are marine invertebrates that produce cellulose as a component of their outer body covering, known as the tunic or mantle. CNCs can be extracted from this tunic,^110−112^ with typical length of 0.1–3.0 μm^88,105,106,113^ and aspect ratio of 50–150.^88,113^

#### 2.2.3 Pre-Treatments

Cellulosic biomass from vascular plants is typically associated with other noncellulosic components that must be removed or degraded to make the cellulose fibers more accessible to acid hydrolysis. Although a few sources (e.g., cotton) are almost pure cellulose, woody biomass contains a considerable amount of lignin and hemicelluloses, which makes the cellulose extraction process more complex.^120^ As a result, CNC production from wood requires several processing steps, most notably the removal of lignin, which is known to be an inhibitor for hydrolysis due to the formation of solid matrices around the cellulose fibers, as previously reviewed elsewhere.^121−124^

A common chemical process for pretreatment of woody biomass is pulping, in which lignin and hemicelluloses are broken down to liberate the cellulose fibers.^121^ Pulping is typically achieved via the kraft process, in which wood chips are processed in a mixture of sodium hydroxide and sodium sulfide at high temperatures (ca. 170 °C), resulting in the depolymerization of lignin into smaller fragments that are soluble in alkaline conditions.^125−127^ Alternatively, the wood chips can be treated with sulfur dioxide and a cationic base, known as sulfite pulping. This process has a less pronounced impact on fiber properties but results in higher cellulose content compared to the kraft process.^121,125,126^ If residual lignin is present after the cellulose extraction process, it can be removed through a bleaching step, which enhances the accessibility of cellulose to hydrolysis^128−130^ and leads to the separation of fiber bundles. However, bleaching can also result in a reduction of the fiber diameter.^129−133^

After purification, additional treatments are often employed to prepare the purified feedstock for hydrolysis and facilitate its handling. For example, purified cellulose fibers are often dried, which decreases their ability to swell^134^ and increases their lateral aggregation.^135^ Drying is also likely to increase the number of defects, making fibers more sensitive toward hydrolysis.^134,136^ Some cellulose sources, such as cotton, are by default harvested in a dry state, and thus may already possess some irreversible effects of drying. Dried material is commonly cut, ground or shredded to increase the surface area accessible for hydrolysis.^101,137,138^ However, studies have demonstrated that extensive ball-milling damages the native crystalline structure of cellulose fibers, which can lead to the formation of spherical cellulose nanoparticles.^139−141^

Microcrystalline cellulose (MCC, commercially known as Avicel) is an alternative starting material for CNC production. MCC is a partially fragmented and depolymerized form of cellulose obtained by partial hydrolysis of wood pulp. This results in CNCs with similar average lengths to those obtained directly from the original wood pulp, but with reduced lateral dimensions and lower polydispersity in length and width.^88^

Cellulose I fibers can be converted into cellulose II by immersion in concentrated NaOH solution, a process patented by John Mercer in 1844 and therefore referred to as mercerization.^142,143^ This widely used commercial treatment involves the swelling of fibers and recrystallization of the cellulose chains, without complete dissolution of the crystal structure used to produce regenerated cellulose.^144^ Nanocrystals of cellulose II (CNC-II) can thus be produced by mercerization of native cellulose followed by acid hydrolysis.^73,145^ An analogous swelling and recrystallization of cellulose fibers can be obtained by the controlled addition of sulfuric acid at relatively low temperatures (around 0 °C, but avoiding complete dissolution at −20 °C),^146^ which can control the amount of conversion from cellulose I to cellulose II prior to the initiation of acid hydrolysis at higher temperature.^147−151^

#### 2.2.4 Acid Hydrolysis

The most widely used process for isolating CNCs from purified cellulose is hydrolysis with sulfuric acid.^152,153^ At the laboratory scale, the source is typically either wood (in the form of pretreated pulp) or cotton (in the form of linters or shredded filter paper). These purified cellulose fibers are typically immersed in 64–65 wt % aqueous sulfuric acid solution at temperatures ranging from 45 to 65 °C, and continuously mixed for a period of 30–60 min,^93,154,155^ though conditions outside of these ranges have also been employed.^91^

Acid hydrolysis is thought to attack the glycosidic bonds at the periodically arranged disordered regions of the fibers (section 2.1), leading to a rapid reduction in the molecular weight followed by a leveling off of the degree of polymerization (LODP).^55−57^ This breakage, along with cellulose affinity for the solvent, induces the separation of laterally assembled fiber bundles. In accordance with the lower critical solution temperature (LCST) behavior of cellulose in sulfuric acid,^146,156^ the use of low hydrolysis temperature (<50 °C), favors the presence of residual oligosaccharide chains (7–20 DP) solubilized from the crystallite surfaces.^157^ These phenomena are facilitated by significant esterification of the surface alcohols (R-OH) into sulfate half-esters (R-OSO_3_H), provided that the acid concentration is high enough.^158,159^ Eventually, this process leads to the formation of highly crystalline particles, whose less accessible glycosidic bonds are more slowly hydrolyzed.

Once the hydrolysis is complete, the medium is quenched with cold water with a two- to 10-fold ratio. During this step, solubilized oligosaccharides present in the medium precipitate onto the CNC surface, decreasing the dependence of their viscosity with ionic strength in suspension.^157,160^ In some cases, an alkaline solution is used to neutralize the pH during quenching. The suspension is then centrifuged and redispersed in water several times to remove the acid and soluble compounds. After centrifugation, the suspension is dialyzed against deionized water until the pH or the conductivity reaches a plateau. During this step, the remaining sugars, acid molecules, and ions are removed from the CNC suspension. Finally, an optional step is to increase the concentration of the suspension to a desired value. This can be achieved using a rotary evaporator or by dialysis in an osmotic bath of a neutral high-molecular-weight polymer (e.g., dextran, polyethylene glycol).^103,161^ The morphology and surface charge of the resulting CNCs strongly depends on the hydrolysis conditions, as discussed in detail in section 8.1, and also depends on any postprocessing of the suspension (e.g., filtration, ultrasonication, surface modification), as discussed in section 8.2.

As a final comment, numerous alternative methods for CNC isolation have also been developed,^23,162^ mainly intending to reduce the large volumes of acid and water required in the process.^163,164^ These methods include the hydrolysis of cellulose using various acids such as hydrochloric acid,^165^ phosphoric acid,^166,167^ hexanoic acid,^168^ and acid blends.^169,170^ Similarly, ionic liquids and deep eutectic solvents are being extensively explored.^171−173^ CNCs produced by hydrochloric acid hydrolysis are not charged, but can be colloidally stabilized by oxidation of the surface alcohols to carboxylic acid groups using the (2,2,6,6-tetramethylpiperidin-1-yl)oxyl radical (TEMPO).^174,175^

#### 2.2.5 Commercial Production of CNCs

Wood is the most industrially exploited source because of its abundance and availability. Industrial processes for isolating CNCs are already well-established in the pulp and paper industry, with many companies now offering commercially available CNCs isolated from wood pulp. Table 1 outlines the characteristics of commercially produced CNCs. CelluForce currently has the largest production capacity (approximately 300 tonnes/year) with several other suppliers also capable of producing over 100 tonnes/year. Notably, CNCs from the University of Maine Process Development Center are commonly used to produce photonic films, despite the relatively low output capacity of this supplier. In contrast, for CNCs from emerging suppliers, their ability to produce photonic films is often unknown and difficult to predict from the supplier datasheets (compounded as the essential properties of CNCs for cholesteric self-assembly are still not well-defined).

**Table 1:** Summary of Commercial CNC Production Methods and Their Properties

Producer	Country	Output Capacity (kg/day, approx.)	Biomass Sources	Type of hydrolysis	Supplied form	Surface state	wt %	pH	Ref
CelluForce	Canada	1000	Bleached softwood kraft pulp	Sulfuric acid hydrolysis	Spray-dried/never-dried suspension	Sodium cellulose sulfate	Powder/5 or 8 wt % suspension	Neutralized to sodium form	(152, 179)
University of Maine Process Development Center	USA	10	Strip-cut dissolving pulp	Sulfuric acid hydrolysis	Freeze-dried/never-dried suspension	Sodium cellulose sulfate	Powder/∼12 wt % suspension	Neutralized to sodium form	(24, 180, 181)
Nanocrystacell	Slovenia	Unknown	Unknown	Unknown	Freeze-dried/never-dried suspension	Unknown	Powder/2–5 wt % suspension	8.7	(182, 183)
Blue Goose Biorefineries	Canada	10	Viscose-grade hardwood dissolving pulp	Transition metal-catalyzed oxidation	Never-dried suspension	Carboxylated CNCs	∼8 wt % suspension	5.8 (Na-CNC)/ 6.48 at 0.2 wt %	(153, 184)
FPInnovations	Canada	Unknown	Bleached chemical wood pulp	Sulfuric or phosphoric acid	Freeze-dried/never-dried suspension	Sodium cellulose sulfate	Unknown	Neutralized to sodium form	
GranBio (formerly American Process)	Brazil/USA	500	Various biomass (woodchips, wood wastes, agricultural wastes)	Sulfur dioxide/ethanol (patented AVAP method)	Unknown	Not charged/coated with lignin	Unknown	Unknown	(152, 185)
CelluloseLab	Canada	Unknown	Dissolving cotton pulp or bleached sulfate hardwood or softwood pulp, Sisal, Tunicate cellulose	Acid hydrolysis (various)	Suspension or powder (spray drying or freeze-drying)	Sodium cellulose sulfate (plus surface-modified)	0.5–10 wt % suspension	Neutralized to sodium form	(186)
Anomera (Rayonier)	Canada	30 (current) 1000 (upscaling in progress)	Softwood pulp	Dilute hydrogen peroxide oxidation	Spray-dried powder or never-dried suspension	Carboxylated CNCs	Powder/3–4 wt % suspension	7 (Na-CNC)/ 7.03 at 0.2 wt %	(153, 187)
Nanografi	Estonia/Germany/Turkey	Unknown	Unknown	Sulfuric acid hydrolysis	Dried; never dried suspension	Sodium cellulose sulfate	Powder/6 wt % suspension	6–7 (Na-CNC)	(188)
InnoTech Alberta	Canada	20	Various bleached hardwood or softwood pulps	Sulfuric acid hydrolysis	Spray-dried powder or never-dried suspension	Unknown	Unknown	Unknown	(189)
Melodea	Israel	650	Various bleached hardwood or softwood pulps and agricultural residues	Sulfuric acid hydrolysis	Never-dried suspension	Sodium cellulose sulfate	3.5 wt % suspension	4.5 (H–CNC and Na-CNC)/5.28 at 0.2 wt %	(11, 153, 190)
Noram (Alberta-Pacific Forest Industries)	Canada	500	Hardwood or softwood/kraft pulp and dissolving pulp	Sulfuric acid by compression reactor	Never-dried suspension	Sodium cellulose sulfate	3 wt % suspension	6.8 (Na-CNC)/ 7.03 at 0.2 wt %	(153, 191)
InnoTech Materials	USA	5	Bleached hardwood pulp or MCC	Iron-activated peroxide oxidation	Hydrogel or powder	Oxidized, amphiphilic or hydrophobic CNCs	Unknown	Unknown	(192)

Innovations have emerged to improve the classical sulfuric acid hydrolysis process. For example, the company Noram developed a compression reactor permitting hydrolysis at a low acid/cellulose ratio, thereby enhancing the sustainability of the process while reducing production costs.^176,177^ The company Melodea developed a procedure to recover the sulfuric acid from the classical hydrolysis,^153^ and it has been shown that implementing a membrane microfiltration step for the acid-CNC separation could improve the environmental impact of the purification method.^178^

### 2.3 Key Properties of Individual CNCs

This section summarizes the fundamental physical and chemical properties of individual CNCs that are relevant for their self-assembly into photonic films. Importantly, some of these properties are inherited from native cellulose and are thus universal (e.g., intrinsic birefringence), while other properties are strongly determined by the production method (e.g., morphology, surface charge) and therefore vary considerably between samples. Furthermore, while many experimental parameters can be objectively quantified, the measurement of morphological properties (i.e., dimensions and shape) is highly dependent on how these nano-objects are defined (see section 2.2.1).

#### 2.3.1 Morphology

**Figure 5:**
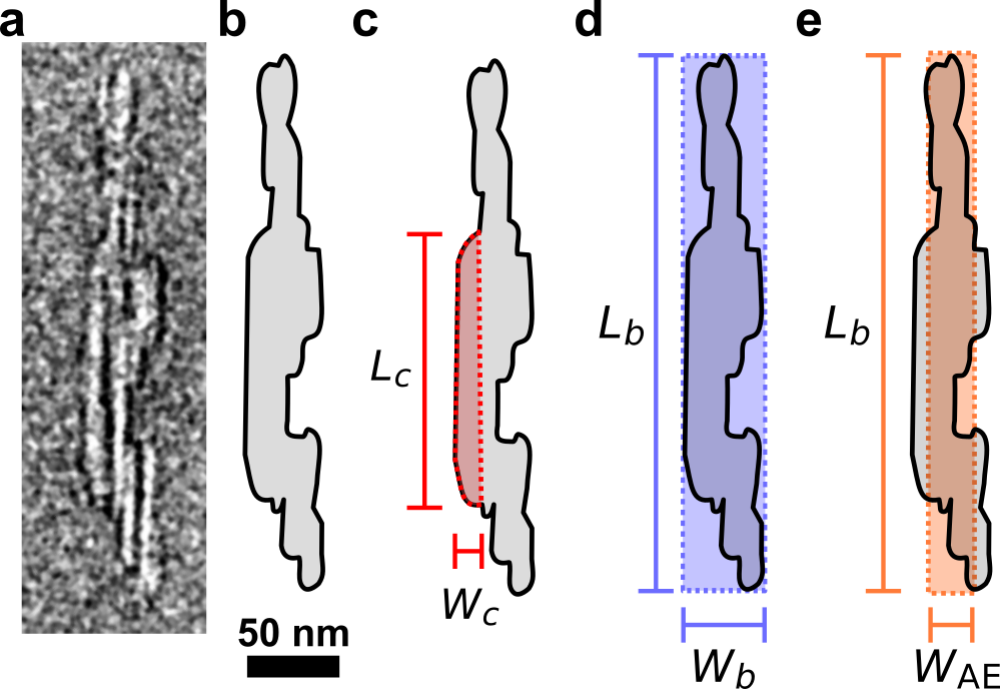
Morphology of cellulose crystallites and CNCs. (a) Example TEM image of a CNC. (b) Outline of the particle in (a). (c) For a cellulose crystallite (red) identifiable within the CNC, the crystallite length *L*_c_ and crystallite width *W*_c_ can be defined. (d) Length *L*_b_ and width *W*_b_ of the box in which the whole CNC particle fits. (e) Length *L*_b_ and area equivalent width *W*_AE_, the latter being the width of the box of Length *L*_b_ as in (d) and equivalent area *A* as shaded in (b). Data from ref (89).

Broadly speaking, CNCs are crystalline nanoparticles with an elongated but irregular shape, as exemplified in Figure 5b. Unlike spherical nanoparticles, whose dimensions can be quantified by a single value (i.e., sphere diameter), the morphological complexity of CNCs makes it challenging to assign precise dimensions to a given sample (i.e., in terms of length, width, thickness). In particular, the CNC population is inherently polydisperse in size and shape, for instance, polydispersity in CNC length, expressed as the coefficient of variation σ̂ (standard deviation over mean, σ/μ) is typically around 0.4.^88,89,193,194^ This considerable polydispersity in CNC morphology necessitates the measurement of a relatively large number of particles (at least several hundred) in order to acquire reliable statistics. It is also important to reiterate that the term “CNC” does not refer to a single homogeneous material, as CNC morphology varies considerably between samples from different cellulose sources and production methods. Finally, the experimental techniques used to measure CNC morphological properties (e.g., TEM or AFM)^24,76,195^ can introduce additional uncertainty, and two researchers measuring CNC dimensions from identical images can obtain slightly different size distributions, as highlighted by recent “inter-laboratory comparisons”.^193,194,196^ As a consequence, only very broad statements about CNC morphology can be made in general, and any number provided should only be taken as crude “typical” values for a particular cellulose source, production method, etc.

To discuss CNC morphology in a more quantitative manner, it is necessary to first address the inconsistency within the literature in how an “individual CNC” is defined. As discussed in section 2.2.1, some sources consider CNCs as synonymous with crystallites (i.e., excluding agglomerated particles),^193,197^ while other sources define CNCs as any discrete colloidally stable nano-object, which can be composed of one or more crystallites (i.e., including agglomerated particles).^72,88,89^ In this review, the latter definition is used, and a clear distinction is therefore made below between the morphology of crystallites and that of CNCs more generally (Figure 5). When comparing values across the literature it is important to consider the definition of CNCs being used; this can be challenging, however, as this choice is not always explicitly stated, and it is often difficult to distinguish crystallites within agglomerated CNC particles.^198^

The morphology of cellulose crystallites is essentially inherited from native cellulose microfibrils but mediated by the production process. The dimensions of the crystallite cross-section observed by TEM or AFM (in terms of crystallite width *W*_c_ and thickness *T*_c_, respectively) are similar to those of the original microfibrils (Figure 4), and are in good agreement with the estimated crystallite size based on the width of X-ray diffraction peaks.^88^ These observations indicate that hydrolysis does not cause significant “peeling” of the crystal structure.^155^ While the crystallite cross-sections have relatively low polydispersity (σ̂ < 0.3 in width and thickness), the crystallite lengths span a wide range of values on the scale of a few hundred nanometers, approximately following a log-normal distribution. The typical crystallite length *L*_c_ is believed to be related to the periodic disordered regions observed in native microfibrils, which have greater susceptibility to hydrolysis.^199^

CNCs often have a poly crystallite structure (i.e., they are composed of one or more laterally attached crystallites). Taking these bundled particles into account leads to higher mean width and thickness values with much greater polydispersity (σ̂ ≈ 0.5), while the CNC mean length is only slightly larger than that for individual crystallites.^200^ For example, CNCs produced from cotton by sulfuric acid hydrolysis and measured from TEM images are typically 50–200 nm long, 10–30 nm wide and 5–15 nm in thickness.^88^

The poly crystallite structure of the CNCs also raises an important point regarding their chemical polarity. CNCs are made from the native cellulose I allomorph, and the individual crystallites are therefore chemically polar (see section 2.1 and Figure 2). However, within a poly crystallite CNC, it is unclear whether the crystallites are exclusively parallel, exclusively antiparallel, or randomly oriented, as suggested by some authors.^145^ It is often assumed in the literature that CNCs made from cellulose I (CNC-I) inherit the chemical polarity of the crystallites, which would imply a parallel arrangement with all the reducing end groups on one end of the particle. While the observation of polar CNC properties (e.g., a net electric dipole contribution, see section 4.1.1) may seem to support this assumption, any imperfectly compensated arrangement of crystallites in CNC-I could lead to an overall asymmetry, but with reducing end groups present on both tips of the CNCs. Note that for CNCs made from crystallites of cellulose II (CNC-II), the antiparallel arrangement of chains within each crystallite directly leads to centrosymmetric properties in poly crystallite CNCs regardless of their internal crystallite structure.

For many applications of CNCs, including those exploiting their self-assembly into photonic films, the particle aspect ratio is one of their most important physical attributes. However, aspect ratio values vary widely, even for samples that are expected to give similar values. This diversity in estimates can be partly attributed to the distinction between crystallites and bundled particles discussed above, and partly to differences in how aspect ratio is estimated. Since the dimensions of the particles are usually correlated, the estimate of the average aspect ratio must be calculated from the aspect ratio of each particle, rather than the ratio of their average dimensions. The most common approaches to estimating CNC aspect ratio are to calculate the ratio of length and width from TEM images (*a* = *L*/*W*) or the ratio of length and thickness from AFM images (*a* = *L*/*T*). These imaging techniques give similar values for the particle length, and the length of the CNCs can be taken either as the Feret length *L*_F_ or the box length *L*_b_ in which the CNC outline fits, without causing significant difference. However, the measured width and thickness values (whether of crystallites or bundled CNCs) often differ by a factor of 2 or more, due to the anisotropy of the particle cross-section and differences of resolution between the two techniques. Moreover, the irregularity of the width outline or the height profile raises the question of the most relevant metric to characterize them. While the width of the fitting box, *W*_b_, provides the largest width the particle can locally have (Figure 5d), a probably more suitable estimate recently proposed for *W* is the area equivalent width, *W*_AE_ = *A*/*L*_b_, where *A* is the area of the outlined particle (Figure 5e).^89^ While crystallites are reasonably well-approximated as cylinders, bundled particles have a highly anisotropic cross-section, and most of the time ill-defined morphologies. In this case, a more relevant estimate of the effective 3D aspect ratio can be obtained by using ,^89,95,201,202^ although this expression requires that both the width and thickness of each particle have been estimated. Beyond the principal dimensions of CNCs, recent studies have explored other dimensionless shape properties of CNCs, such as circularity, elongation, and rectangularity, as a way to classify CNCs based on their morphology.^89,203^

While CNCs do not possess cylindrical symmetry, it is sometimes useful to approximate their morphology to cylinders or spherocylinders, as these idealized shapes are often used for theoretical models of the behavior of anisometric particles. For a CNC of given *L*, *W*, and *T*, the corresponding cylinder can be defined as having the same length *L* and a diameter  (and radius *R* = *D*/2), thus preserving the average cross-sectional area and particle volume; however, the definition  is also encountered and preserves the definition of the aspect ratio *a* = *L*/*D* = *a*_3D_.

Isolated microfibrils of native cellulose have a tendency to twist in a right-handed screw-like fashion about the chain axis with a periodicity on the order of several hundred nanometers, as experimentally observed by electron microdiffraction and morphological analysis.^136,204−207^ This axial twist has also been observed in numerous recent simulation studies,^208−211^ where the periodicity was predicted to be inversely proportional to the area of the microfibril cross-section. This relationship can be understood in terms of a simple elastic model of a beam undergoing torsion: the observed periodicity arises from the competition between the molecular torque imparted by each d-glucose residue to its neighbors (which scales linearly with the cross-sectional area) and the torsional stiffness of the microfibril (which scales quadratically with the cross-sectional area).^206^

Cellulose crystallites are also expected to have a right-handed twist, but this morphological chirality is often not very pronounced–for example, characterization of 3D crystallite morphology using cryogenic electron tomography did not observe an enantiomeric excess of a given handedness.^212^ The difficulty in observing the morphological twist on crystallites can be attributed to the relatively mild twist (1–10°/nm), which would be more noticeable for crystallites longer than one full twist, or for CNFs.^136,206^ Furthermore, drying a dilute CNC suspension onto a substrate (as done for conventional TEM and AFM imaging) creates a competition between the tendency for crystallites to twist, individually or within a single CNC, and the tendency to form a flat continuous interface with the substrate.^205^ While this effect can eliminate the twist for shorter crystallites, the buildup of distortion can cause highly localized twisting of longer crystallites that is more prominent when imaged.^136,204,205^ For CNFs dried on a flat surface, the twist periodicity has also been reported to decrease with surface charge density, suggesting an electrostatic effect on crystallite morphology.^213^ Finally, it has been proposed that bundled CNCs have a chiral “propeller-like” morphology, to account for the role they play in the cholesteric mesophase; however, experimental evidence to support this hypothesis is still lacking.^89^

#### 2.3.2 Surface Chemistry

Pure cellulose is insoluble in water and many other organic solvents.^214^ The reason for this insolubility is still investigated, but is believed to be mostly due to two factors: first, both the intrachain hydrogen bonds centered along the chain axis (*c*-axis) and the interchain hydrogen bonds formed parallel to the glucopyranose ring plane (i.e., (1 1 0) plane of cellulose Iα and (1 0 0) plane of cellulose Iβ), which compete with cellulose-water hydrogen bonds, and second, the hydrophobic stacking interactions of van der Waals origin perpendicular to the pyranose ring plane.^215^ Consequently, nanocellulose can be dispersed in water, but requires surface modification to be colloidally stable. This can be achieved by adding charged groups to the CNC surface (e.g., by sulfuric or phosphoric acid hydrolysis or TEMPO oxidation), or by steric stabilization (e.g., by grafting hydrophilic polymers), as discussed in section 8.2.1.

The isolation of CNCs from native cellulose by sulfuric acid hydrolysis results in the grafting of negatively charged sulfate half-ester groups onto the CNC surface.^82,87,216^ The surface charge for sulfated CNCs can be expressed in several units, depending on the technique used for measurement. When CNC sulfate half-ester content is determined by conductometric titration,^24,217^ the surface charge is most commonly expressed as the sulfur molality, i.e., number of moles of sulfur *N*_S_ per CNC mass *m*_CNC_, in mol/g:
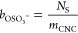
1

Alternatively, the results of elemental analysis^24^ often express the sulfur content as a mass fraction (wt %) given by
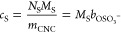
2where *M*_S_ = 32.1 g/mol is the molecular weight of sulfur. The degree of substitution for sulfate half-esters on glucose residues can then be calculated from *b*_OSO_3_^–^_ or *c*_S_ as
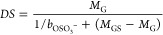
3where *M*_GS_ and *M*_G_ are the molecular weights of sulfated and nonsulfated glucopyranose units (241.2 and 162.1 g/mol, respectively). As a quantitative comparison, a typical specific surface charge of 2 × 10^–4^ mol/g (200 mmol/kg) corresponds to a sulfur mass fraction of 0.64% or a degree of substitution of 3.2%. Although the sulfur content per CNC mass is readily accessible experimentally, the more relevant physical property for thick elongated particles is the charge per surface area (i.e., areal surface charge density), which in terms of elementary charges *e* is typically on the order of 0.1 *e*/nm.^2^ However, values for the CNC surface charge density are highly sensitive to how the surface area is estimated from the morphology of individual particles (see above).^89,200^ Consequently, surface charge densities calculated from individual particle morphology should be considered order-of-magnitude estimates at best.

As discussed in sections 5.1 and 5.2, the CNC surface charge determines the range of suspension conditions (i.e., ionic strength values) in which the particles are colloidally stable. In terms of zeta potential, dilute suspensions of sulfated CNCs typically have values in the −20 to −50 mV range,^24^ indicating broad colloidal stability.

#### 2.3.3 Electric and Magnetic Properties

In general, cellulose has extremely low electric conductivity and is therefore used extensively as an insulator.^218^ For nanocellulose, the conductivity of CNFs has been estimated to be on the order of 10^–10^ S/cm at 40% relative humidity,^219^ which can mostly be attributed to ionic transport within remaining residual water. Native cellulose (both cellulose Iα and Iβ allomorphs) has a permanent electric dipole moment parallel to the chain axis, due to the accumulation of contributions from each glucose residue. This dipole moment can be detected from transient electric birefringence measurements of CNCs suspended in apolar solvents, and was experimentally estimated to be approximately 4000 D.^220^ The lack of inversion symmetry in cellulose I is also expected to lead to a piezoelectric response.^221^ In contrast, the antiparallel chain arrangement in cellulose II is not expected to result in a net permanent electric dipole or to allow for piezoelectricity.

Native cellulose is weakly diamagnetic, like most organic materials, and has anisotropic diamagnetic susceptibility due to its crystal structure. Consequently, native cellulose can be aligned by magnetic fields in dilute suspensions, but this requires high field strengths and sufficiently large particles to observe this behavior. For example, individual cellulose Iβ microfibrils were aligned using a 7 T magnetic field,^222^ while the alignment of tunicate CNCs required fields up to 18 T.^223^ Moreover, the negative diamagnetic susceptibility of native cellulose (i.e., lower susceptibility parallel to the chain axis and higher susceptibility normal to the glucose residue rings) leads to alignment perpendicular to the applied magnetic field. Importantly, while high magnetic field strengths are required to align individual CNCs, the cumulative nature of this effect allows the collective alignment of cholesteric CNC suspensions at much lower field strengths, as discussed in section 4.4.

#### 2.3.4 Optical Properties

In general, cellulose has a relatively high refractive index for an organic material. For example, ellipsometry of amorphous cellulose films found that the index decreases from *n*_ave_ = 1.56 at 400 nm to *n*_ave_ = 1.52 at 700 nm.^224^ Cellulose also has negligible absorption in the visible range and therefore behaves as an ideal dielectric material.

The refractive index of crystalline cellulose differs along the crystallographic axes, a property known as birefringence. This optical anisotropy is essential for the structural coloration of photonic CNC films, as discussed later in section 7. In principle, crystalline cellulose could have a different refractive index along each of its crystallographic axes. However, when measuring the birefringence of cellulose fibers experimentally it is only possible to distinguish between the effective refractive indices parallel and perpendicular to the fiber axis (*n*_∥_ and *n*_⊥_ respectively). Using index-matching liquid mixtures, the effective refractive indices for ramie fibers were found to be *n*_∥_ = 1.603 and *n*_⊥_ = 1.523, leading to a birefringence of *Δn* = *n*_∥_ – *n*_⊥_ = 0.080.^225^ However, estimates of the birefringence are sensitive to the alignment of chains relative to the overall fiber and the water content. Taking into account these effects, an ideal fiber of cellulose I with an assumed density of 1.592 g/cm^3^ was estimated to have indices *n*_∥_ = 1.618 and *n*_⊥_ = 1.544 so that *Δn* = 0.074, while the indices for a macroscopic cellulose fiber with an assumed standardized density of 1.520 g/cm^3^ were *n*_∥, std_ = 1.590, *n*_⊥, std_ = 1.519, and *Δn*_std_ = 0.071.^226^ As a final comment, CNCs dispersed in a medium of differing refractive index acquire an effective, extrinsic birefringence that also depends on their elongated shape, as discussed in section 7.1.

#### 2.3.5 Mechanical Properties and Density

Native cellulose is remarkably stiff and strong for a material of such low density, which has attracted interest in using cellulose nanomaterials as mechanical reinforcement for composites.^29,39,81^

The exact crystal structure of cellulose Iβ, based on X-ray crystallography, corresponds to a mass density of 1.64 g/cm^3^, and a comparison of measured cellulose density from different sources corroborates this value.^70,227^ The density can also be estimated from electron diffraction data,^228^ but radiation-dependent expansion of the crystal structure leads to lower, dose-dependent values.^229^ Experimental density values of dry cellulose powder is usually considered around 1.5 g/cm^3^ due to their imperfect crystallinity and porous structure.^226,227^ For CNCs, which are almost entirely crystalline and have a convex shape, a density estimate of 1.6 g/cm^3^, closer to the crystallographic value, is probably more appropriate.

Cellulose microfibrils have a high longitudinal elastic modulus, with estimates often exceeding 100 GPa. Although reported values are generally high, they vary significantly between measurement methods and cellulose sources. In particular, it is challenging to accurately determine mechanical properties of individual microfibrils using traditional tensile testing,^58,230^ as it is difficult to assess the transfer of stress through polycrystalline cellulose fibers and the matrix surrounding the microfibrils. The elastic properties can be measured more reliably by applying hydrostatic pressure and uniaxial stretching to the cellulose crystal structure.^231^ Alternatively, inelastic X-ray scattering (IXS) can be used to circumvent this stress transfer issue.^232^ An additional complication of mechanical testing of cellulose is that the elastic modulus values are time-dependent, with higher reported modulus values for faster deformation rates.^231^

#### 2.3.6 Toxicity

Native cellulose is biocompatible, biodegradable, and nontoxic. While it is plausible to assume that these properties are preserved in CNCs, it is appropriate to question whether their nanoscale dimensions may lead to additional complications.

The environmental health and safety properties of CNCs and other cellulose nanomaterials are reviewed elsewhere.^233−238^ In summary, no adverse effects were observed in humans following oral or dermal exposure. Ingestion tests performed on rodents using CNC-containing animal feed did not show any adverse effects, with a ‘no-observed-adverse-effect’ level similar to conventional cellulose. For dried cellulose nanopowders, short-term exposure by inhalation was found to cause transient inflammation similar to (non-nanoscale) cellulose and other low-toxicity dusts in general (i.e., readily mitigated with standard safety measures, such as the use of filtration masks or local exhaust ventilation). However, the effects of long-term, low-dose exposure have not yet been assessed. The lack of identified adverse effects has led to regulatory approval for unrestricted use of CNCs in Canada,^239^ and commercial CNCs have also received regulatory clearance for use in the US and Europe.^240^

Inhalation of nanopowder is highly unlikely when handling CNCs, both for the preparation of photonic films or for other applications, as individual CNCs are only present in aqueous suspension, while freeze-dried CNC powder consists of much larger aggregates. Furthermore, photonic CNC films contain agglomerated particles that cannot be easily redispersed.

### 2.4 Summary

CNCs are elongated nanoparticles of crystalline cellulose that are extracted from native microfibrils by acid hydrolysis. The properties of individual CNCs vary considerably depending on the cellulose source and extraction method. However, even for comparable CNCs there is variation in reported properties, which can be partly attributed to how these values are measured. As a consequence, there is a growing need to standardize the characterization of CNCs to enable meaningful quantitative comparison between independent studies.^24,152,153^ How the properties of individual CNCs influence their liquid crystal and colloidal behavior is respectively explained in sections 3 and 5, while the corresponding experimental parameters that can be used to tune these properties are discussed in section 8.

## 3 Self-Organization of CNCs into Cholesteric Suspensions

CNCs are elongated nanoparticles that, due to their high aspect ratio, behave as a colloidal liquid crystal above a threshold volume fraction. In this regard, CNC suspensions lie at the intersection of two distinct classes of soft matter systems, sharing some properties of both colloids and liquid crystals (see section 1.2). Their colloidal nature makes the pair interaction potential between CNCs the key parameter to control and understand their behavior in suspension, while their lyotropic (i.e., concentration-driven) liquid crystal nature makes the volume fraction the key parameter for their self-assembly. In both cases, the aspect ratio of the CNCs influences their collective behavior and leads to the alignment and local birefringence necessary for the development of optical effects.

In this section we introduce the self-organization of CNCs into liquid crystalline suspensions. First some fundamental properties of the nematic and chiral nematic liquid crystal ordering are introduced, before discussing the concepts behind the Onsager model for lyotropic colloidal liquid crystals, with its further refinements, to explain how nonchiral hard rods can collectively form a nematic liquid crystal. The treatment of chiral interactions between CNCs to explain the cholesteric order observed in CNC suspensions is then discussed, and finally the pathways of the transition from isotropic to liquid crystal are summarized.

For self-organization to occur, it is crucial that the CNCs are colloidally stable. As colloidal stability is linked to the mechanisms involved in kinetic arrest, a discussion of this aspect of CNC behavior will be reserved for section 5, which is dedicated to the kinetic arrest of CNC suspensions. The present section thus focuses on how CNC suspensions form cholesteric phases assuming good colloidal stability.

### 3.1 Fundamentals of (Chiral) Nematic Liquid Crystals

This section introduces general theoretical concepts about nematic and cholesteric liquid crystals, as well as a note on the terminology used to distinguish them. The common defects found in these mesophases are then presented. In this description, the liquid crystal is treated as a continuum and the detail of the mesogens (i.e., the molecules or the CNCs) is implicitly described by the properties of the liquid crystal.

#### 3.1.1 Nematic Liquid Crystals

To describe and understand the cholesteric liquid crystal phase, it is helpful to first consider the *nematic phase*. A nematic phase is a collective ordering of freely moving elongated particles, where the particles develop a long-range correlation with each other in their orientation but not in their position.^241^ In liquid crystals (also called “mesophases”), these particles are commonly referred to as mesogens, and can correspond to molecules or colloidal particles. While the direction in which each mesogen is oriented fluctuates over time, it is correlated with the orientation of neighboring mesogens, resulting in a local average alignment. A director field **n**(*x*, *y*, *z*, *t*) capturing the direction of average alignment can then be constructed as a continuous vectorial field from a spatial moving average across the sample. This average is made at a scale that is large enough compared to the size of each mesogen, but small enough to capture the local variation of collective alignment in space and time. For prolate (calamitic, i.e., elongated) mesogens, **n** is usually indicating the direction of their longest axis, while for oblate (discotic, i.e., flattened) mesogens, **n** indicates the direction of their shortest axis. Importantly, it is defined as nonpolar (i.e., the transformation **n** → −**n** has no effect on the structure),^18^ so that its sign (±) is meaningless (in contrast to ferroelectric molecular liquid crystals, where the overall orientation of mesogens alters the mesophase properties). In a spherical polar coordinate system, **n** corresponds to a polar angle θ and azimuthal angle φ relative to the chosen axis, and this mapping is unique except for the equivalence of θ and θ + π due to the nonpolar nature of **n.**

While the director **n** only represents the local average orientation of the mesogens, the orientation of individual mesogens locally fluctuates in space and time, so that there is inevitably some spread in the orientation directions around this mean value. Defining an orientation distribution function *f*(θ, φ), the variance in orientation can be expressed using the nematic order parameter *S*_2_, which is given by
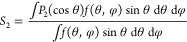
4where *P*_2_(cos θ) is the second Legendre polynomial in cos θ, namely
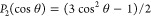
5

**Figure 6:**
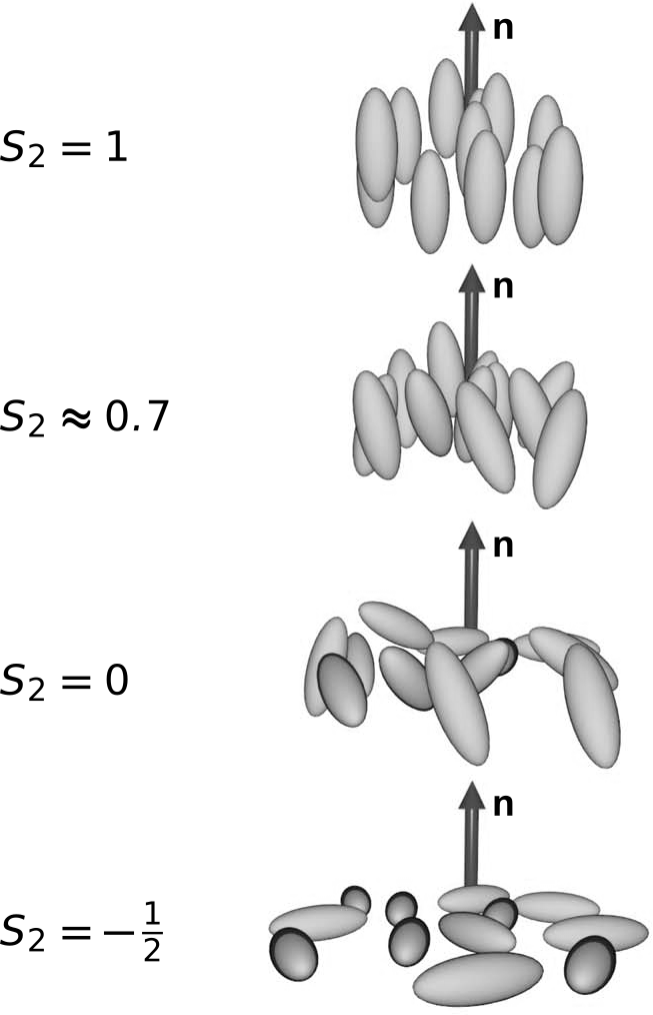
Configuration of mesogens (represented as gray ellipsoids) relative to the local nematic director **n** for values of the order parameter ranging from S_2_ = 1 to S_2_ = −1/2. Adapted from ref (243) under Creative Commons CC-BY. Copyright 2019 The Authors.

It can be seen that *S*_2_ is the average value of *P*_2_(cos θ) over the orientation distribution of the system. It should also be noted that the equation for *S*_2_ coincides with the Hermans order parameter (usually denoted *S*) which is typically calculated from X-ray diffraction diagrams.^242^ For perfect parallel alignment of mesogens, *S*_2_ = 1, while for the isotropic case of completely random alignment, *S*_2_ = 0, and for the case of perfect antialignment with mesogens perpendicular to **n**, *S*_2_ = −1/2 (see Figure 6).

**Figure 7:**
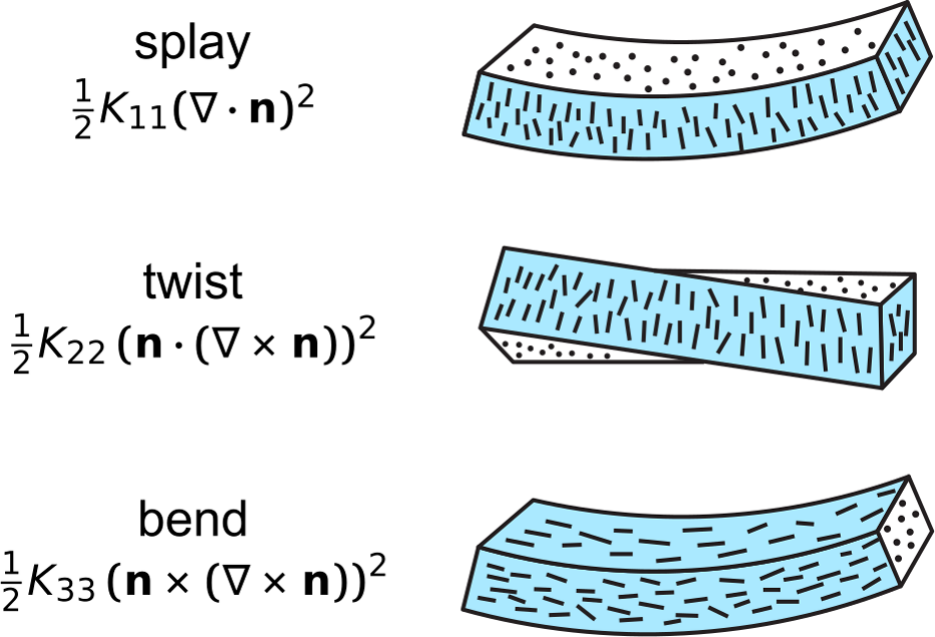
Schematic showing the three principal distortions of the director that contribute to the Frank-Oseen free energy for a nematic (i.e., *q* = 0). Adapted with permission from ref (247) under CC-BY. Copyright 2018 The Author.

Regardless of the nature of the interactions responsible for their local alignment, the long-range orientation of the mesogens in a nematic phase will tend to relax toward the most thermodynamically stable state at equilibrium. Local variations in the average orientation of the mesogens are captured by spatial derivatives of the director field **n.** The principal distortions of the director field in the bulk of nematic phases are known as *splay*, *twist*, and *bend* distortions, and are illustrated in Figure 7. It is then possible to integrate over the volume of the sample to calculate the energetic costs of all these variations in a particular geometry. The integrand is defined as the Frank-Oseen distortion free energy density , and requires knowing the Frank elastic constants *K*_11_, *K*_22_, *K*_33_, and *K*_24_, associated with different elastic deformations of nematics:
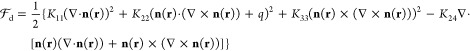
6Here, ∇ is the spatial derivative operator *nabla*. Note that the fourth term (*K*_24_) corresponds to saddle-splay distortions that occur near interfaces; some of its effects can be included into *K*_11_ and is thus often omitted,^244^ but can play important roles when confined within boundaries of high curvature.^245,246^ The total free energy of the sample is obtained after integration over the sample volume:
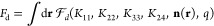
7

In a regular, i.e., achiral, nematic, the expression above simplifies with *q* = 0, where *q* is a term becoming relevant only for chiral nematic (cholesteric) systems and will thus be defined in the next section.

Typical values for the Frank elastic constants in molecular liquid crystals are in the ∼1–10 pN range and are relatively well characterized.^241^ In contrast, the Frank elastic constants for lyotropic colloidal liquid crystals such as CNCs, *fd* viruses or amyloid fibers were reported only in a small number of publications, with considerable uncertainty in their values, either because of model-dependent estimations, or because their values were determined only relatively to one another (e.g., as ratios of *K*_22_/*K*_33_, as discussed in section 4.3).^244,248−250^ One comparison between various lyotropic nematics for rod-like mesogens showed that the twist elastic constant *K*_22_ is almost 1 order of magnitude weaker than *K*_11_ and nearly 2 orders of magnitude weaker than *K*_33_,^251^ while these three elastic constants are usually of the same magnitude for a typical molecular nematic (5CB), suggesting that lyotropic nematics are more susceptible to weak chiral perturbations. Such asymmetry between the elastic constants was also deduced for CNC suspensions from the observation of the conformations of tactoids of different sizes.^244^ Analytical formulas are also available to estimate these elastic constants for lyotropic nematics made from rigid rod-like particles, but are only qualitatively consistent with a weaker *K*_22_.^244,252,253^

Additional constraints arise at the interfaces between a liquid crystal and its surroundings. The interfacial tension depends on the local angle between the director and the normal to the surface, which can cause a preferential director orientation at the interface, known as *anchoring*. In terms of free energy, anchoring is accounted for by an integral over the sample outer interface in contact with its immediate surroundings (e.g., air, another immiscible fluid, or a solid substrate):
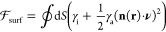
8Here, *γ*_i_ represents the isotropic component of the surface or interfacial tension of the liquid crystal phase with the material *i*, and is usually in the order of 10–100 mN/m, or it can be the isotropic phase and it is then much weaker, about 10^–4^ mN/m.^244^ The second term, *γ*_a,_ is the anchoring Rapini-Papoular interfacial tension that depends on the director and the local surface normal **ν**. Its sign is usually determined by the material and leads to either planar anchoring (i.e., parallel to the interface for *γ*_a_ > 0) or normal anchoring (for *γ*_a_ < 0) to the surface.^254^ While both positive and negative *γ*_a_ values are observed in molecular liquid crystals, prolate (calamitic) colloidal liquid crystals such as CNCs tend to only align planar to most surfaces, and in any direction within the surface plane, due to the unfavorable translational entropy cost of the positional ordering induced near the interface under normal anchoring.^255^ Moreover, while in molecular liquid crystals the anchoring is usually strong and dominates completely at the interface, colloidal liquid crystals often display so-called “weak anchoring phenomena”, where the director might not be as strongly held parallel at the interface.

At thermodynamic equilibrium, the arrangement of the director **n**(**r**) reaches a steady state configuration that minimizes the total free energy  of the system. More generally, the nonequilibrium dynamic response of nematic liquid crystals to flow is complex and beyond the scope of this review: an introduction to the relevant theoretical treatment (i.e., Ericksen-Leslie theory of nematodynamics) can be found elsewhere.^256^

#### 3.1.2 Cholesterics

**Figure 8:**
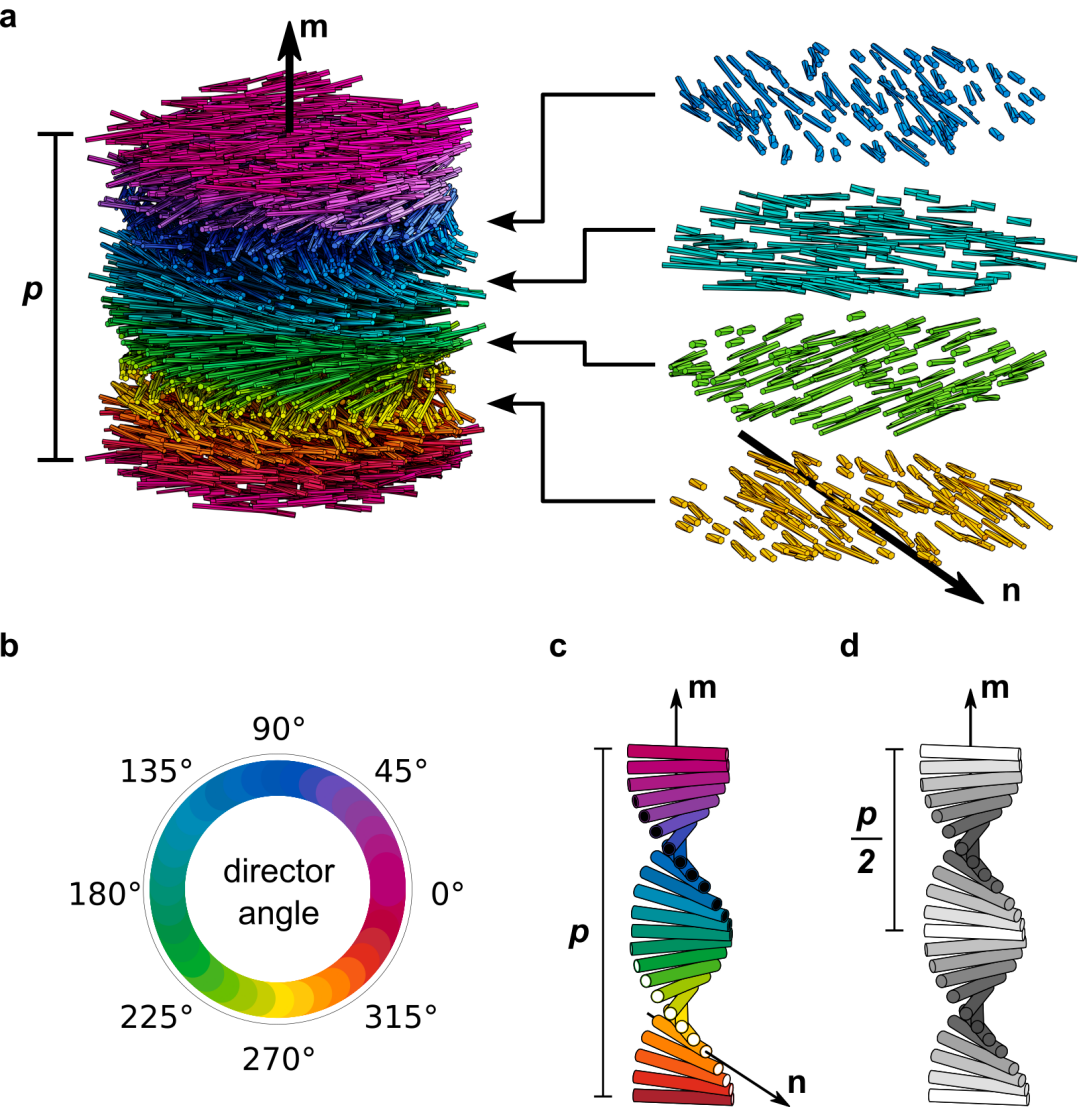
(a) Illustration of a cholesteric liquid crystal phase of densely packed rod-like mesogens, closely resembling CNCs. Although the mesogens exhibit no positional order, they have local nematic-like ordering with a director **n** that rotates in a left-handed helicoid manner about the helical axis **m**. The cholesteric pitch, p, corresponds to a full 360° rotation of the director. The mesogen rotation is highlighted by sampling out four thin slices at different locations along the helical axis, which are color-coded by their director angle (relative to an arbitrary reference direction). (b) Color wheel illustrating the color code used for the director angle in (a) and (c). (c) Schematic representation of the helicoidal director in a left-handed cholesteric, with corresponding definitions for **m**, *p*, and **n**. Note that the cylinders here represent the average local director, and not the mesogens. (d) As the director is nonpolar, the structural periodicity is *p*/2.

A cholesteric mesophase is locally aligned, resembling a nematic phase, but also exhibits a periodic rotation of the director field **n**(**r**) along one axis, known as the helical axis (Figure 8a, b). The resulting alignment can be described as a stack of continuously rotating slices of nematic alignment of infinitesimal thickness (Figure 8c). The helical axis can be described by an additional nonpolar unit vector **m**, such that the director is always oriented perpendicular to the helical axis (**n** ⊥ **m**), as illustrated in Figure 8a. If the coordinate system is chosen to that the helical axis **m** is parallel to the **z** axis, the director field can be written explicitly as
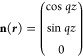
9where *q* is the cholesteric wavevector, which describes the handedness and periodicity of the helicoidal structure and already appeared in the expression for the Frank-Oseen free energy given above. The sign of the wavevector *q* indicates whether the structure is right-handed (*q* > 0) or left-handed (*q* < 0). For nonzero values of *q*, the director rotates about the helical axis with a period (pitch) *p* = 2π/|*q*|, which is defined to be always positive. However, it is important to note that due to the symmetry of the director (**n =** −**n**), the spatial period of the structure is actually *p*/2 = π/|*q*| (Figure 8d). This distinction between the periodicity of the cholesteric (*p*) and the apparent periodicity of the structure (*p*/2) is a frequent source of confusion, and can lead to incorrect pitch values being reported. The pitch *p* is typically much larger than the mesogen length or diameter, justifying why the arrangement can be locally modeled as nematic, which is just a special case of cholesteric when *p* → ∞ (i.e., q → ± 0).

In a given isocline (i.e., a virtual slice or pseudolayer through the cholesteric structure at constant *z*), **n**(**r**) has a constant value and resembles a nematic mesophase. As the pitch *p* is typically much larger than the mesogen length or diameter, the local arrangement of mesogens within a pseudolayer is accurately described as being locally nematic. However, as recently emphasized by Schutz et al.,^18^ these pseudolayers are purely a conceptual aid and do not correspond to actual discrete layering of aligned mesogens in the physical system. In the Frank-Oseen free energy formalism, the overall twist of the cholesteric phase is the result of the competition between the torque induced by chiral mesogen interactions, expressed as a chiral twist elastic constant (*K*_t_), and the restoring elastic energy of the untwisted nematic state, given by twist elastic constant (*K*_22_), yielding *q* = −*K*_t_/*K*_22_.^241,248^

#### 3.1.3 Defects in Cholesteric Phases

The tendency of the mesogens to align with each other makes sudden changes in alignment unfavorable: a discontinuity in alignment corresponds to a large derivative term, and therefore a large contribution to the Frank-Oseen free energy. Consequently, the director field usually varies smoothly and continuously in space, and it is expected that the net variation of its direction along a closed loop should cancel out to zero. When this is not the case, it indicates that the loop encloses a singularity where the director field is discontinuous, and the liquid crystal structure contains a defect. Crucially, defects can move in space but cannot spontaneously relax into a defect-free director field, making them a topologically conserved feature of the system.

**Figure 9:**
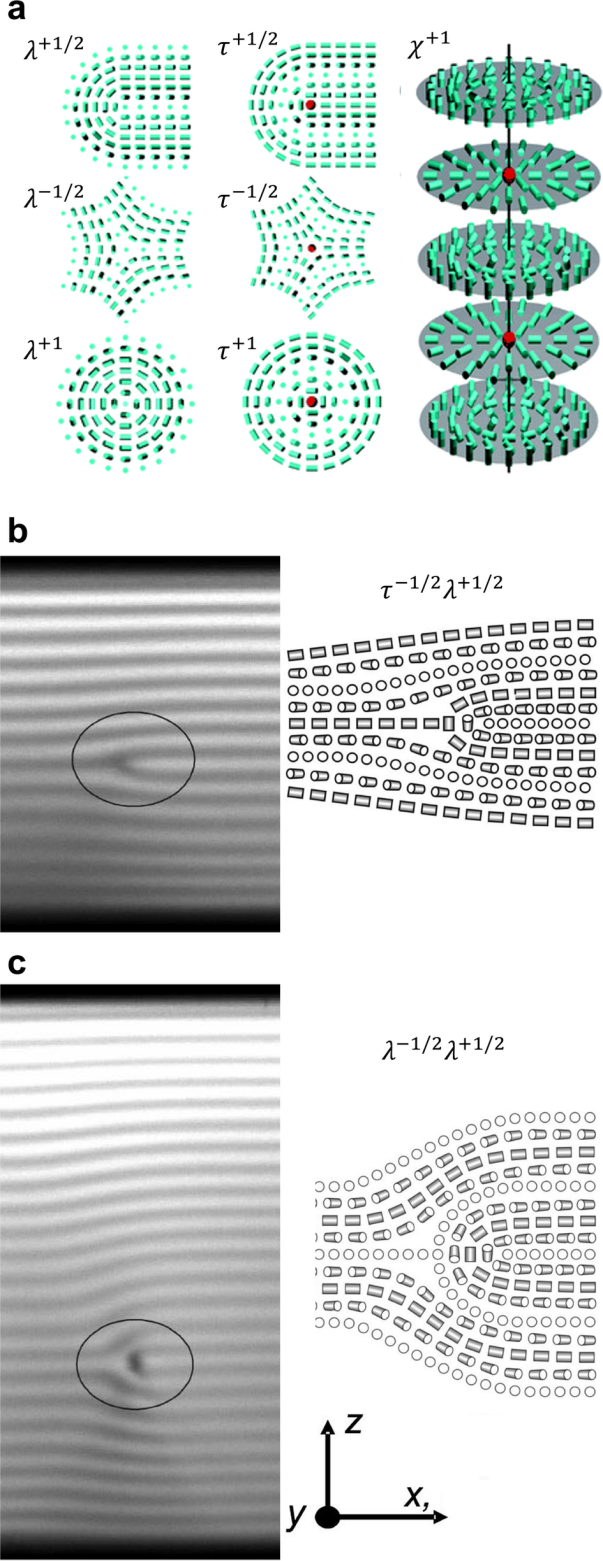
(a) Schematic representation of cholesteric mesogens (shown as cylinders) with disclination lines λ^m^, τ^m^, and χ^m^. The disclination lines are indexed by their winding number. Note that some disclinations with m = 1 are theoretically possible but energetically unstable. Reproduced with permission from ref (258). Copyright 2012 The Royal Society of Chemistry. (b, c) Three-dimensional director structures of defects of cholesteric liquid crystals imaged by fluorescence confocal polarizing microscopy and the schematic of their corresponding topological description as pair of disclination lines. Adapted with permission from ref (259). Copyright 2002 American Physical Society.

The simplest defects in liquid crystal structures are one-dimensional and are known as disclination lines, while more complex defects can be created when several disclination lines interact. A topological classification of these defects was provided by Kléman and Friedel, and is usually expressed in terms of vectors χ (along the helical axis **m**), λ (along the director **n**) and τ = χ × λ (along the axis perpendicular to both **m** and **n**).^257^ The local disclination lines are depicted in Figure 9a. In a planar geometry, such as in a well-aligned monodomain, linear defects in cholesterics usually involve pairs of such disclination lines of opposite topological charge. An illustration of these defects is presented in Figure 9b−c. These defects can be retained in the solid films obtained from CNC suspensions and can influence the final optical appearance (see section 7.3.7).

#### 3.1.4 Cholesteric versus Chiral Nematic: A Note on Terminology

Historically, the mesophase described above was first observed in molecular systems, specifically cholesteryl benzoate and other cholesterol derivatives, giving rise to its description as a “cholesteric” phase.^260,261^ As the cholesteric phase clearly shares features with the nematic phase, but with an additional chiral element, this phase has also been described as a “chiral nematic”. For a long time, the cholesteric phase was the only known liquid crystal phase with both chiral and nematic aspects, and consequently the terms “cholesteric” and “chiral nematic” have long been treated as synonymous. Moreover, the presence of chiral centers in molecular mesogens is often sufficient to induce a cholesteric pitch, which reinforced the association. In recent years, however, other systems have been found which decouple the chiral properties of the mesogens from the chiral and nematic properties of the resulting phase (e.g., nematic twist bend, blue phases, etc.), and these systems often have a director much different from the expression in eq 9. For instance, achiral bent-core molecules can form nematic twist bend phases made of a racemic mixture of locally chiral domains, in which the director field **n**(**r**) is both chiral and nematic but not cholesteric. Another example is the formation at very low ionic strength of a nematic phase of aligned CNCs, that are also known to be chiral, at least at the molecular level, resulting in a chiral, and nematic, liquid crystal that is not cholesteric. It is therefore correct to say that a cholesteric phase is both chiral and nematic, but a chiral mesophase with local nematic ordering is not necessarily a cholesteric. Just as a nematic that is forcibly twisted due to external constraints into a high energy-state is not a cholesteric (as *q* is zero for a nematic phase, as defined in the expression of its free energy), a forcibly untwisted cholesteric is not a nematic. The ubiquitous distortion of the cholesteric order at length scales smaller than the pitch upon anisotropic compression, as described in section 6, makes makes the final structure chiral, and nematic, but no longer cholesteric. However, localized distortions of the cholesteric phase at length scales larger than the pitch, due to local defects or long-range variation of the helical axis, are common in cholesteric phases, and so the terminology remains appropriate. Throughout this review, we therefore refer to the liquid crystal phases observed in CNC and ChNC suspensions as cholesteric.

A related issue is the use of nematic terminology to describe the properties of cholesterics at small scale. The nematic phase corresponds to a limiting case of cholesteric when *p* → ∞ (i.e., *q* → ± 0), which makes it tempting to include the nematic case as a subset of cholesterics. Since the pitch of a cholesteric is usually large compared to the distance between two neighboring mesogens, their average alignment is, to the first approximation, unidirectional along the director field **n**(**r**), and justifies most attempts to model cholesteric systems as a nematic with a chiral perturbation.

At this point, it is also worth discussing the relationship between the terminology for the liquid crystal phase and the resulting photonic films. When a colloidal liquid crystal such as a CNC suspension is solidified (e.g., by drying) and retains the cholesteric order, it results in a solid material that is nonflowing and no longer behaves as a liquid crystal. This solid material is arguably a solid phase that is no longer liquid crystalline, so calling it cholesteric or chiral nematic is therefore improper. However, the structure can still be referred to as displaying a cholesteric or chiral nematic order. If distortions in the director field leads to nonsinusoidal modulations of the director, then even the terminology “cholesteric order” is inappropriate, while one could argue that “chiral nematic” could still be valid, as the director field would still retain both the chiral and the nematic characteristics. We have therefore chosen to use the term “cholesteric” alone when referring to the liquid crystal, while the term “cholesteric order” will be used for its structure irrespective whether it is liquid or solid, and “helicoidal” (avoiding the term “chiral nematic”) will be used in the most general case where cholesteric ordering, or a distortion of it, is found in liquid or solid samples. For this latter case, an alternative terminology such as “arrested cholesteric” could also be valid, since it indicates that the starting order was cholesteric but is, since the onset of the kinetic arrest, unable to relax internally and its structure should inherit all of the distortions subsequently imparted to it.

### 3.2 Lyotropic Colloidal Nematic Description of CNC Suspensions

The description of liquid crystals provided above treats mesogens (whether molecular or colloidal) with the same theoretical framework as a continuous medium with a given set of properties. However, the continuum model does not cover how these collective properties emerge from the properties of the individual mesogens.

The conditions under which mesogens form a nematic phase depend on their morphology and interactions. In molecular liquid crystals, the formation of the liquid crystal phase is highly sensitive to temperature. In contrast, liquid crystal phases of colloidal particles are relatively insensitive to temperature, while phase formation is triggered by a change in particle concentration in the surrounding solvent. These two cases are referred to as a thermotropic and lyotropic liquid crystals, respectively.^241^ Phase formation for systems of macromolecules such as cellulose derivatives (hydroxypropyl cellulose, ethyl cellulose, etc.) lie in the intermediate regime and can display both temperature and concentration dependence.^33^

In the following section, we provide some basic concepts from the Onsager theory necessary to understand lyotropic liquid crystal phase formation, and then some modifications that render the theoretical description more relevant to CNCs, as extensively reviewed elsewhere.^18,262^ Experimental observations of the isotropic–anisotropic phase transition of CNC suspensions will then be presented and discussed.

#### 3.2.1 Onsager Theory for Hard Rods

In colloidal sciences, the behavior of dilute suspension is often described as a van der Waals (vdW) gas, in which the colloidal particles act as the “molecules” and the dispersing suspension acts as the gas “volume” in which the particles can move. The macroscopic properties of a gas (e.g., pressure Π and temperature *T*) are related by a “gas law” of the form Π = Π(*T*). For an ideal gas composed of ρ particles per unit volume, the gas law has the simple form Π = *ρk*_B_*T*, where *k*_B_ is Boltzmann’s constant. Deviations from the ideal gas law can be described using the virial expansion
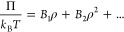
10with the ideal case corresponding to *B*_1_ = 1 and *B*_*j*_ = 0 for *j* > 1. The higher-order virial coefficient can be directly related to the pair interaction potential between particles (*V*_12_), in particular, the second virial coefficient is
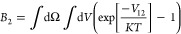
11Here, *B*_2_ is obtained by averaging over all positions in space and, in the case of anisotropic particles, over all mutual orientations. Using this definition *B*_2_ has units of volume, but can alternatively be defined as *B*′_2_ = *B*_2_*N*_A_/*M*_w_^2^, and is then expressed in units of (L mol g^–2^), with *N*_A_ the Avogadro constant and *M*_w_ the molar mass of the particles.

The vdW gas is a modification of the ideal gas law to include two main corrections: the inclusion of a term corresponding to the particle covolume (corresponding to the volume per particle that cannot be occupied by other particles because of impossible physical overlap) and a term to account for short- to medium-range interactions between particles, aside from their hard-particle repulsion, which could be either attractive or repulsive. For spherical particles, these terms can be introduced fairly easily, while for anisotropic particles it is necessary to include the effects of the mutual orientation of the particles, which greatly complicates the expressions.

By considering the B_2_ contribution to the total free energy, Lars Onsager demonstrated how particle shape anisotropy can lead to spontaneous collective alignment above a threshold volume fraction.^263^ Onsager considered the interactions of hard (nonoverlapping), rigid (inflexible) spherocylinders of length *L* and diameter *D* ≪ *L* (and thus aspect ratio *a* = *L*/*D* and volume ). He found that above a certain volume fraction, collective alignment of the rods gives the state of lowest thermodynamic free energy because the decrease in orientation entropy from rod alignment is accompanied by a greater capacity for the rods to move without overlapping, thereby increasing their translation entropy and giving a net entropy gain overall.

Importantly, Onsager showed that the excluded volume *v*_cyl_^exc^ of such particle depends on their relative orientation, and that in the case of an isotropic distribution, can be written as
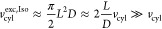
12

For an isotropic suspension of such particles interacting purely as hard rods, *B*_2_ is positive and is simply equal to the covolume, namely . It is therefore convenient to introduce the *Onsager excluded volume fraction*, defined as Φ̃ = Φ*a* since it corresponds to the expression of the volume fraction of *B*_2_ for the corresponding isotropic hard rods given above.^263,264^ Onsager found that rods undergo a first-order phase transition with increase concentration: a pure isotropic phase would be unstable above volume fraction of Φ > Φ_*b*1_ = 3.34 *a*^–1^, while the pure anisotropic phase is unstable below Φ < Φ_*b*2_ = 4.49 *a*^–1^. This implies that for a range of volume fractions Φ_*b*1_ < Φ < Φ_*b*2_, a macroscopic suspension of rods should be biphasic between these two binodal points (note, these volume fractions of coexistence are commonly referred to as Φ_0_ and Φ_1_, respectively).

Later numerical studies confirmed these findings, although the positions of the phase coexistence boundaries depends on the trial function used for the nematic orientation distribution function: for instance, a Gaussian trial function leads to Φ_*b*1_ = 3.45 *a*^–1^ and Φ_*b*2_ = 5.12 *a*^–1^.^265^ While the qualitative behavior of Onsager-type models applies to many colloidal nematic systems, it is quantitatively accurate only in highly ideal circumstances. Since Onsager’s original work, some refinements have been introduced to bring the predictions of the model into better agreement with real experimental systems such as CNCs, as discussed below. The chirality of a cholesteric phase can also be considered a deviation from ideal nematic behavior, but this special case will be introduced and discussed separately in section 3.3.

#### 3.2.2 Case of Low Aspect Ratio *a* = *L*/*D*

Onsager’s model assumes that the second virial coefficient of the free energy expansion is dominant and higher-order virial terms can be neglected, an assumption that holds in the limit of infinite aspect ratio (i.e., when *a*^–1^ → 0 and Φ_*b*1_ and Φ_*b*2_ ≪ 1), and is a good approximation for *a* > 100.^262^ The effect of higher-order terms has been explored using Monte Carlo simulations, which show that Φ_*b*1_ and Φ_*b*2_ deviate significantly from the predictions of the Onsager model for *a* < 100.^266,267^ CNCs from most sources have aspect ratios on the order of 10, and even CNCs from tunicate (with the highest reported aspect ratios) rarely reach the *a* > 100 regime.^268^ Note that in the case of noncylinder particles such as CNCs, the 3D aspect ratio *a*_3D_ (introduced in section 2.3.1) is more appropriate to be fully consistent with the definition of *B*_2_.

#### 3.2.3 Presence of Electrostatic Interactions

In the presence of additional repulsive interactions, which could be of either steric or electrostatic origin, the particles usually repel at a distance larger than their bare diameters. If they effectively prohibit overlap at a distance with a gap smaller than 2δ, the dimensions of the particles can be renormalized to effective dimensions such as *D*_eff_ = *D* + 2δ and *L*_eff_ = *L* + 2δ (for analytic and experimental expressions of δ in the case of electrostatic repulsion, see section 5.1.1 and eq 28). From these, another useful quantity to introduce is the effective volume fraction Φ_eff_ of the particles so that this additional shell of thickness δ is included in the effective volume *v*_CNC_^eff^ of each particle. For a spherocylinder with *L* ≫ (*D*, δ), thus assuming *L*_eff_ ≈ *L*, one finds that  and thus
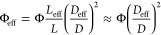
13

This effective description of the particle dimensions also decreases the rod aspect ratio from *a* = *L*/*D* to *a*_eff_ = *L*_eff_/*D*_eff_, leading to
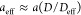
14

The ionic double layer around charged (achiral) rods creates a soft repulsive barrier before the hard particle surface. The effect of the double layer can be incorporated into the expression for the second virial coefficient, in what is usually referred to as the “SLO theory”, named after its authors, A. Stroobants, H. Lekkerkerker, and T. Odijk.^269^ The effective diameter of the rods is also increased from *D* to *D*_eff_ = *D* + 2δ, where δ is expected to be on the order of the Debye length κ^–1^. Moreover, this soft electrostatic interaction is maximal when the rods are parallel, resulting in an effective torque twisting the rods away from this unfavorable configuration toward a perpendicular configuration. Local interactions between charged rods therefore tend to align them perpendicular to each other, whereas rods entropically aim to be parallel, and the equilibrium between these two states is some nonzero twist angle. However, for achiral rods this twist has no preferred handedness, so it will not lead to a chiral mesophase.^265^ The increase in effective particle diameter both decreases the effective aspect ratio *a*_eff_ ≈ *a* (*D*/*D*_eff_) and increases the effective volume fraction Φ_eff_ = Φ (*D*_eff_/*D*)^2^. When the Onsager conditions are stated in terms of effective quantities Φ_eff_ and *a*_eff_ and then rewritten in terms of the bare aspect ratio and volume fraction that are more accessible experimentally, the resulting two critical volume fractions of coexistence finally scale as
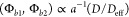
15effectively shifting the phase transitions to lower particle volume fractions as δ increases.^270^ Since most photonic CNC films are made from casting aqueous suspension of CNCs, experimental parameters that affect δ are therefore of particular importance for controlling the self-assembly process.^271,272^ Aside from electrostatic interaction, the description of particle shape using effective dimensions is also relevant for surfactant-stabilized CNCs in an apolar solvent, with δ corresponding to the thickness of the surfactant layer in that case,^273^ or in the hybrid case where both electrostatic and steric effects are present (e.g., negatively charged CNCs coated with an adsorbed layer of polymer).^274−276^

#### 3.2.4 Effect of Polydispersity

It is possible, albeit complicated, to expand the analytical treatment of Onsager from one to many types of particles. Early theoretical studies considered the simpler case of a bidisperse system,^277^ while the later work of Wensink and co-workers tackled the general problem of polydispersity for continuous length distributions.^278^ These studies predict several modifications of Onsager’s theory for polydisperse systems, most notably (1) a widening of the biphasic region, with both lower Φ_*b*1_ and higher Φ_*b*2_, and (2) size fractionation of the particles, with high-aspect-ratio particles preferentially entering the nematic phase during the early stages of the phase separation. In the limit of high polydispersity, i.e., for coefficients of variation σ̂ > 0.5 with σ̂ = σ/μ (standard deviation over mean), an unusual separation into three phases (I–N–N) is also predicted, with different rod size distributions in each phase. These predictions have been confirmed in various colloidal nematic systems, including boehmite rods,^279,280^ sepiolite clay rods,^281^ and *fd* viruses.^282^

As discussed in section 2.3.1, CNCs exhibit considerable polydispersity in length, aspect ratio and other morphological properties: for instance, CNC lengths typically have σ̂ ≈ 0.4. Given the considerable polydispersity observed for CNC length and width, the effects of polydispersity are expected to be significant for CNC suspensions. The fractionation effect in biphasic CNC suspensions has repeatedly been used as a refinement process to produce suspensions with lower polydispersity,^271,272,284^ as discussed further in section 8.2.3. The self-assembly of samples produced by sequential fractionation is generally consistent with their differing aspect ratios, with smaller aspect ratios phase-separating at higher volume fractions.^272,284,285^ Fractionated samples present a narrower biphasic range of concentrations than their unfractionated source suspensions,^284^ in agreement with theoretical predictions.^278^ Three-phase equilibria have been reported in several studies for unfractionated suspensions of tunicate CNCs, which can be attributed to the unusually high polydispersity (σ̂ > 0.5) of these systems.^113,268^ In suspensions of other highly polydisperse rods, fractionation can lead to formation of additional liquid crystal phases (e.g., smectic), but this behavior has not been reported for CNCs.^286^

The specific effects of polydispersity of cholesteric suspensions have also been investigated analytically using a Onsager-type model with Straley-type chiral interactions (as introduced later in section 3.3).^287^ It was found that polydispersity increases the twist energy constant *K*_22_ but not *K*_t_, so that polydispersity “stiffens the nematic fluid”, making it harder to twist the mesophase and causing an increase of the pitch. Furthermore, it was predicted that doping the system with long rods of constant diameter should increase the pitch (i.e., long rods should reduce the chiral strength). The coexistence concentrations in the biphasic region were also predicted to depend on overall rod concentration due to fractionation effects, with the pitch in the biphasic region between 70% and 100% of its value at Φ_*b*2_.

#### 3.2.5 Observations of Liquid Crystalline Phase Separation of CNCs

**Figure 10:**
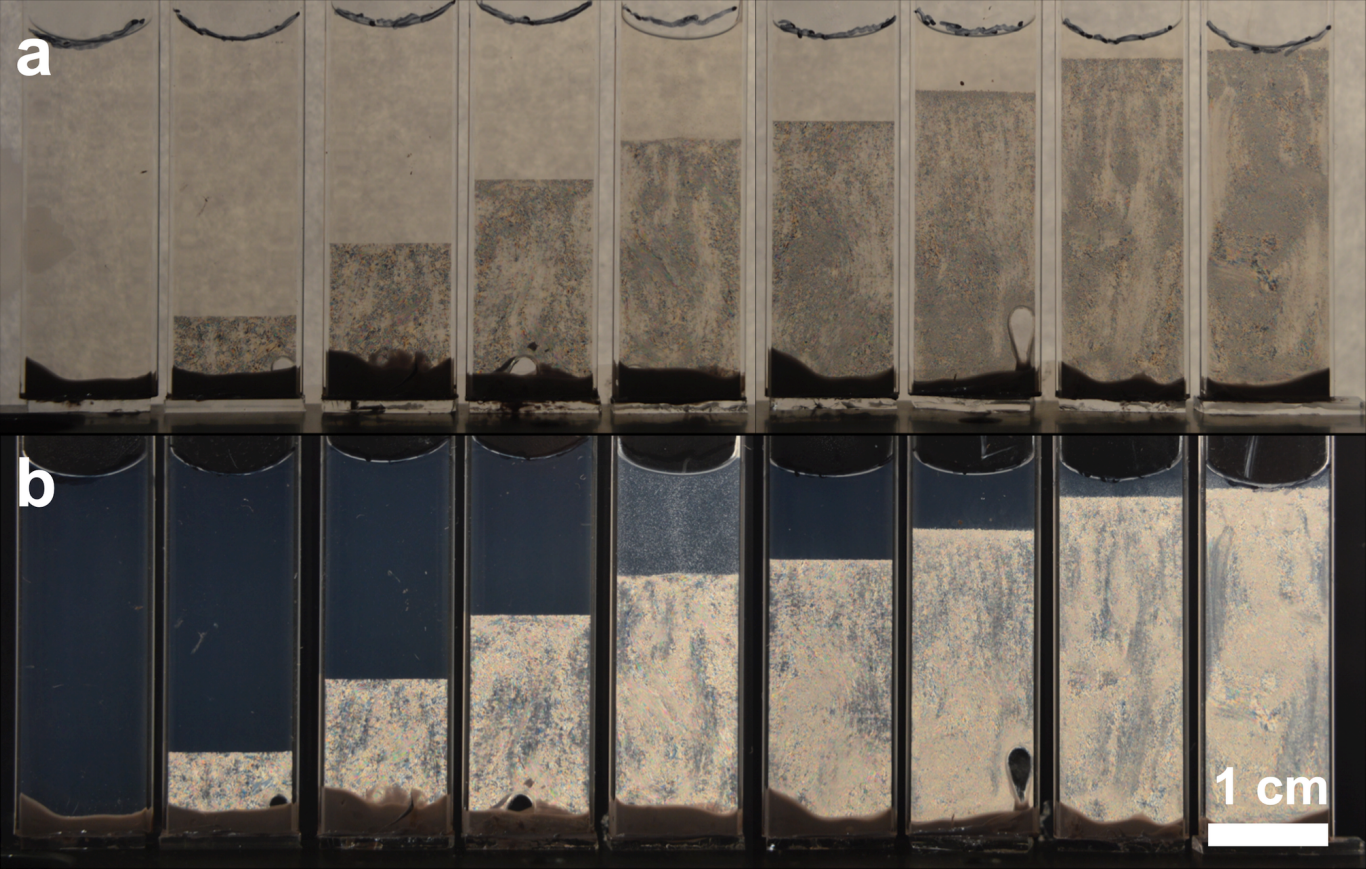
Capillaries of increasing volume fraction of CNCs from left to right, observed between linear polarizers (a) in uncrossed (parallel) configuration and (b) in crossed configuration. Adapted with permission from ref (288) under CC-BY. Copyright 2016 The Authors.

**Figure 11:**
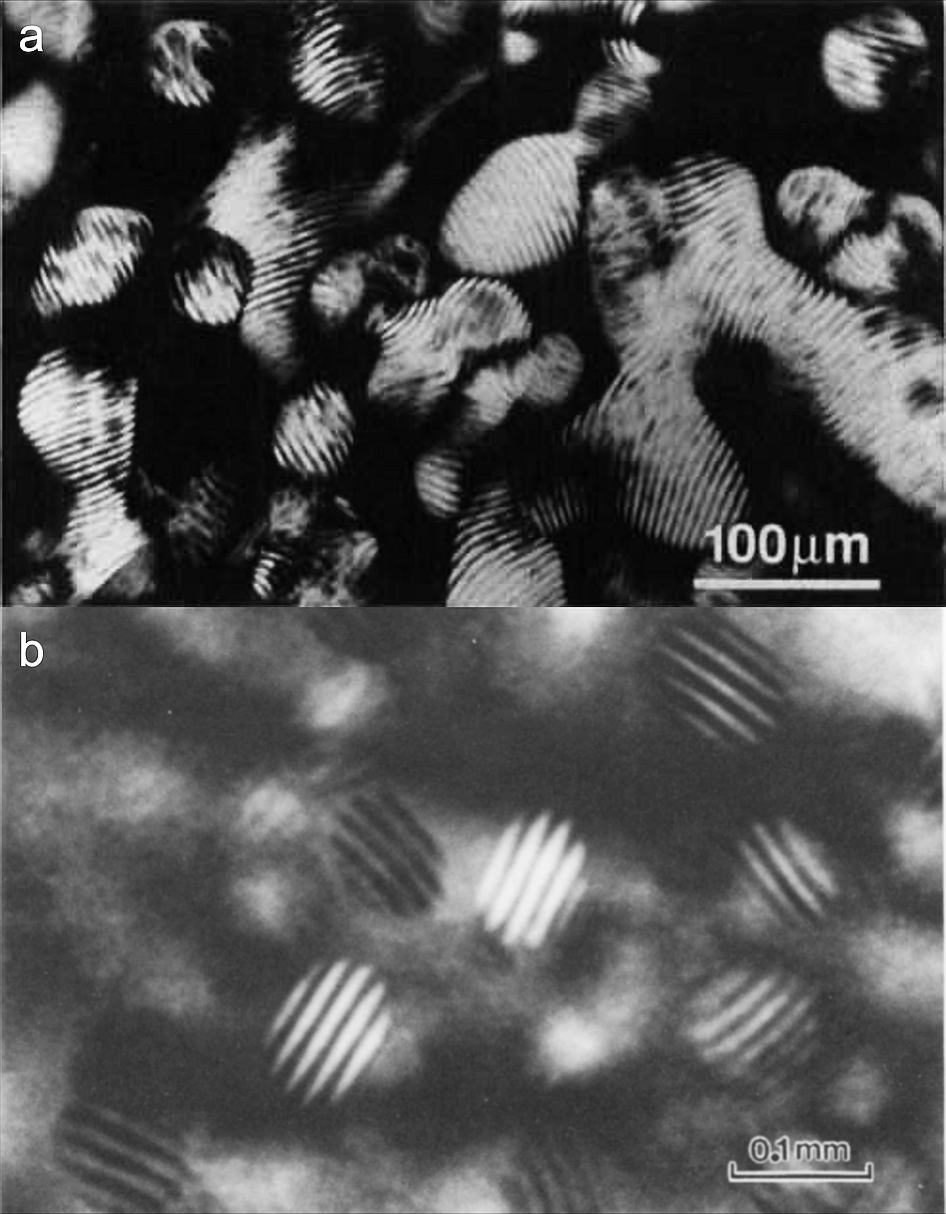
POM images of tactoids in biphasic CNC suspensions, imaged in transmission between crossed polarizers (with a monochromatic camera) (a) Tactoids formed upon concentration increase. Reproduced with permission from ref (137). Copyright 1994 Taylor and Francis Ltd. (b) Tactoids observed a few minutes after ceasing agitation of the sample. Reproduced with permission from ref (20). Copyright 1992 Elsevier.

**Figure 12:**
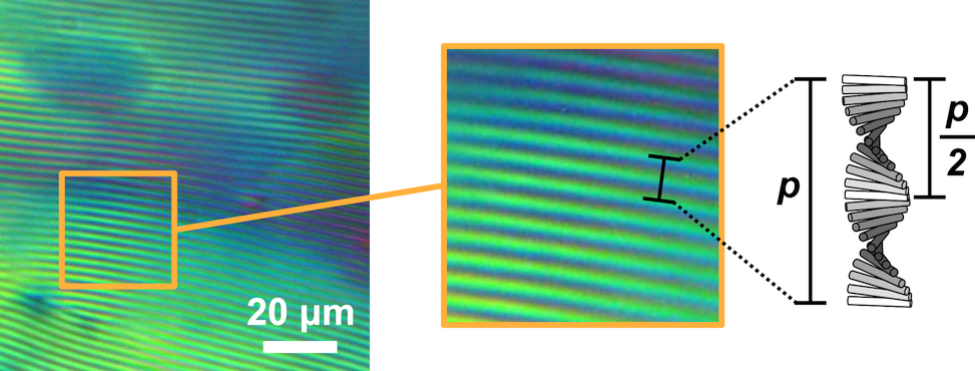
Anisotropic (cholesteric) phase of a suspension of cotton-derived CNCs at *c* = 10.2 wt %, as observed by optical microscopy performed in transmission mode between crossed polarizers. The fingerprint pattern, characteristic of the cholesteric ordering when the helical axis is in the observation plane, can clearly be observed in the inset. The cholesteric pitch corresponds to twice the periodicity of the pattern, as illustrated with the sketched helicoid. Adapted with permission from ref (288) under CC-BY. Copyright 2016 The Authors.

As first reported by Revol et al., CNC suspensions above a critical concentration spontaneously separate into two coexisting phases.^20^ The phases are easily distinguished when viewed between crossed polarizers, as the lower, anisotropic phase appears bright while the upper, isotropic phase appears dark (see Figure 10). When examined using polarized optical microscopy (POM), the bottom phase has a periodic “fingerprint” pattern of evenly spaced parallel bright and dark bands (see Figures 11 and 12). These observations are consistent with the formation of a cholesteric liquid crystal phase, in which the CNCs are locally aligned with each other within a large-scale helicoidal structure. The anisotropic arrangement of birefringent CNCs leads to the bright appearance of the bottom phase between crossed polarizers, while the isotropic, random arrangement of particles in the upper phase appears dark.

If a dilute sample (i.e., initially in the isotropic state) is concentrated beyond a certain threshold, a phase separation process is usually observed over time. The aligned phase typically first appears in the form of droplets within the isotropic phase, known as tactoids, which grow with time (Figure 11). The fingerprint pattern can be observed for sufficiently large tactoids (with diameters much greater than the pitch). After sufficient time (typically from a few hours to a few days), macroscopic phase separation is observed. In almost all reported suspensions, the aligned phase is denser and is therefore found underneath the isotropic phase (Figure 10), with the notable exception of a recent work where the order of the two phases was reversed (but without the formation of a cholesteric phase).^289^ The cholesteric phase then appears continuous in POM, and exhibits a periodic “fingerprint” pattern (Figure 12).

As CNC phase separation is dependent on their concentration, it is worth briefly discussing how CNC concentration is measured and reported. Experimentally, it is convenient to estimate the CNC concentration by drying the CNC suspension and weighing it before and after to determine the amount of water lost. This method results in an estimate of the CNC concentration in solvent as a mass fraction, *c*, (wt %, wt/wt). However, the CNC volume fraction Φ (vol %, vol/vol) is a physically more relevant quantity, which can be estimated from the CNC mass fraction, *c*, as
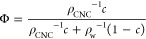
16where ρ_CNC_ and ρ_w_ are the densities (mass per volume) of CNC and water, respectively (see section 2 for further discussion of the CNC density). Alternatively, the mass concentration of CNCs (ρ_CNC_Φ, in mg/mL) can also be estimated. In some cases, it is also relevant to estimate the CNC number density (number of particles per volume), but this is highly dependent on having an accurate estimate of the individual volume of CNCs and how to correctly average them.

In stable colloidal suspensions, the negatively charged CNCs repel each other more than particles of the same shape experiencing only hard rod interactions. Consequently, the Onsager-like lyotropic behavior of CNCs cannot be fully described by their bare volume fraction Φ. A possible approach to overcoming this issue is to express CNC concentration in terms of their effective volume fraction Φ_eff_, when accounting for their nonoverlapping volume. While this approach allows for an easier application of models assuming hard-rod interactions, it is also model-dependent and depends on the knowledge of the size and shape distribution of CNCs and the range of the repulsion between the CNCs. Due to these uncertainties, the effective volume fraction Φ_eff_, when used to report the phase and pitch dependences in CNC suspensions, should not be reported in place of *c* or Φ but only in addition to them, with clear explanations of the conversion used.

**Figure 13:**
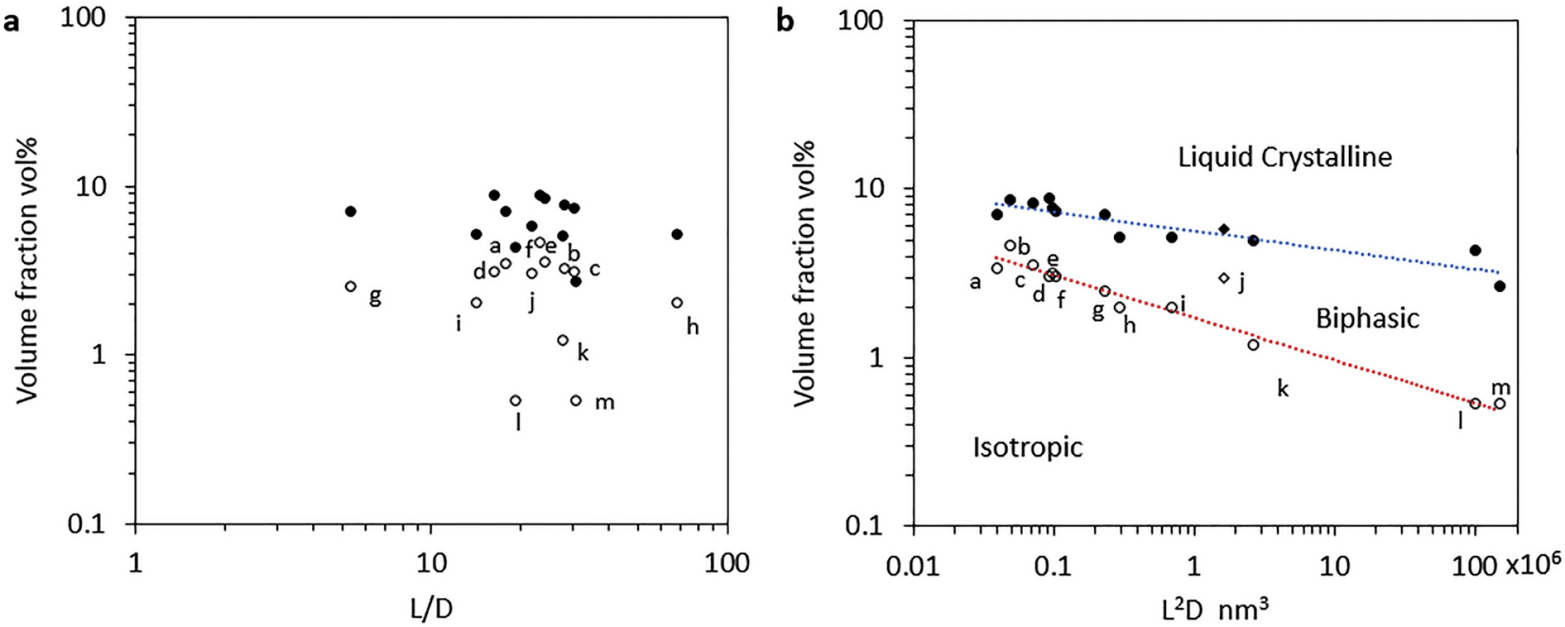
Volume fraction of CNCs at the isotropic-biphasic (Φ_*b*1_) and biphasic-anisotropic (Φ_*b*2_) transitions in aqueous suspensions. Literature values of Φ_*b*1_ (open circles) and Φ_*b*2_ (filled circles) are presented (a) as a function of *a* = *L*/*D* and (b) as a function of L^2^D. Data sources were selected for CNCs of similar proportion of surface sulfate groups (*c*_S_ ≈ 0.89–1 wt % on a dry cellulose basis) and polydispersity (σ̂ = 0.3–0.5 aside from point j, which has a narrow particle size distribution). Labels correspond to data from the following publications: a (kraft^290^), b (black spruce^94^), c (black spruce^94^), d (filter paper^271^), e (black spruce^94^), f (eucalyptus^94^), g (cotton powder^291^), h (wood^292^), i (wood^293^), j (cotton^106^), k (algae^106^), l (tunicate^106^), m (bacterial cellulose^104^). Lines are best fit for the two transitions (point j excluded from fitted data). Reproduced with permission from ref (264). Copyright 2019 Elsevier.

The boundary conditions of the biphasic regime from a variety of CNC sources and bare aspect ratios were compared by Xu et al. and showed poor correlation with the bare aspect ratio *a*,^264^ (see Figure 13a) deviating from the predictions of Onsager theory.^263^ Reporting the same data in terms of *L*^2^*D*, proportional to the particle hard-rod excluded volume, they found a general agreement across most available data in the literature, except when the polydispersity of the CNCs was not comparable (Figure 13). In this representation, the boundary conditions followed an empirical relationship:
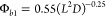
17
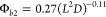
18

While the clear spread of the data along these two lines suggests some underlying connection with the relevant physical parameters, it is important to notice that neither the dimensions (*L*, *D*) nor the volume fraction Φ did include effective quantities to account for the correction from hard-rod repulsions. The implications of these boundaries on the self-assembly and how they interplay with the onset of kinetic arrest will be discussed further in section 5.4.2.

### 3.3 Including Chirality in Lyotropic Colloidal Nematics

While the previous models were mostly relevant for achiral colloidal nematic liquid crystals, some source of chirality is required to drive the formation of a cholesteric order. In this section, we first introduce the challenges in understanding the chiral interaction between CNCs, before presenting some experimental clues to identify its origin, and finally discussing possible mechanisms responsible for their chiral behavior in relation to existing models.

#### 3.3.1 Challenges of Understanding the Origins of Mesophase Chirality

To predict the behavior of cholesteric mesophases, it is necessary to extend the models described in the previous section to account for chiral interactions. As mentioned in section 3.1.2, chirality can be introduced in the Frank-Oseen free energy as an additional term −*K*_t_***n***(***r***) · (∇ × ***n***(***r***)) where the strength of the chiral distortion is captured by the parameter *K*_t_ defined as the chiral elastic constant,^259^ also known as the chiral strength.^294^ The equilibrium cholesteric wavevector *q* is then given by *q* = −*K*_t_/*K*_22_, with negative values for *q* corresponding to left-handed cholesteric structures, and vice versa.

Although the inclusion of chiral contributions into the effective elastic free energy description of nematics is straightforward, there is a multitude of ways such a contribution can arise in practice. Chirality, in essence, is the absence of inversion symmetry (i.e., mirror symmetry),^295,296^ and consequently any local interaction that breaks this symmetry could potentially lead to a chiral nematic mesophase. Furthermore, chiral interactions between mesogens are typically only a small perturbation from their dominant nematic alignment. While this means that the insights from Onsager-type models of lyotropic nematics also generally apply to cholesterics, the relative weakness of chiral interactions also makes it harder to identify their origin.

For a given experimental system that exhibits cholesteric ordering, there are essentially three main questions to address: (1) what is the microscopic origin of the observed mesophase chirality, (2) what is the mechanism of chirality transfer from the mesogens to the mesophase, and (3) to what extent can the cholesteric wavevector be quantitatively predicted from the properties of the mesogens. Answering these questions, especially (3), is notoriously difficult for all systems that exhibit cholesteric behavior, including other rigid rod-like particles such as fd-virus,^248,297^ flexible colloidal particles such as DNA origami filaments,^298,299^ lyotropic liquid crystal surfactants,^300,301^ and for the broad class of thermotropic molecular liquid crystals, despite over a century of research.^302−304^ There is also an extensive literature on the related question of how best to quantify the chirality of a given object both for molecules and for rigid geometric objects, which is discussed in detail elsewhere.^295,305^

To address these questions in the case of CNC suspensions, it is useful to first review the clues provided by experimental observations of cholesteric CNC suspensions (section 3.3.2 below), which can then be compared to the predictions of particular models of cholesteric assembly (section 3.3.3).

#### 3.3.2 Experimental Clues about Chiral Interactions Between CNCs

The cholesteric pitch *p* = 2π/|*q*| decreases with the CNC volume fraction Φ, as observed for many other lyotropic cholesteric mesophases. The pitch dependence is typically described as a power law *p* ∝ Φ^–ν^. The power law exponent has been reported to have values in the range of ν = 1–2 for CNCs in both aqueous^89,94,268,272,288,292,306−309^ and nonaqueous^202,249,268,310^ suspensions, with exponent values at the lower end of that range for cotton CNCs and at the higher end of that range for wood-derived CNCs. However, it is important to bear in mind that power-law fitting of *p*(Φ) for CNC suspensions is problematic for several reasons. First, the effective volume fraction of CNCs is often difficult to estimate accurately due to the effects of electrostatic screening (in aqueous solvents) or the stabilizing surfactant layer (in apolar solvents). Second, for biphasic suspensions Φ is often taken as the average volume fraction across the suspension, rather than the volume fraction of the cholesteric phase alone, which substantially affects the observed trend. Furthermore, fractionation of the CNCs into different subpopulations between the isotropic and the cholesteric phases can cause additional variations to the pitch (see sections 3.2.4 and 8.2.3). Finally, the concentration range in which a CNC suspension is fully cholesteric and not kinetically arrested is typically less than an order of magnitude, which makes it difficult to accurately estimate the power law exponent.

Insight into the mechanism of chirality transfer can also be obtained by investigating the dependence of pitch on the mean particle length or width, which can also be described as power law relations (i.e., *p* ∝ L^γ^ and *p* ∝ W^γ′^). In this case, the data in the CNC literature is conflicted. A comparison of CNC suspensions with comparable diameters produced by different methods found a positive correlation between pitch and length (γ > 0).^94^ In contrast, a comparison of phase-fractionated CNC suspensions reported a negative correlation with roughly γ = −1, concluding that larger CNCs favor a smaller pitch.^272^ More recently, a morphological analysis by TEM and AFM coupled with pitch monitoring in suspensions and films on cotton CNCs suggested that a subpopulation of larger CNCs promoted a smaller pitch, with a scaling of 1/*p* proportional to their volume weighted fraction within the overall CNC population.^89^ In that case, the higher cross-sectional aspect ratio *a*_XS_ = *W*/*T* of larger CNCs, rather than their length, was considered the crucial factor. The positive correlations between, on one hand *L* and *W* and, on the other hand, *W* and *a*_*XS*_, then infer both γ < 0 and γ ′ < 0.^89^

In aqueous CNC suspensions, modifying the ionic strength has also been shown to affect the pitch. The addition of electrolytes typically leads to a pitch decrease (as discussed further in section 8.3.1),^271^ provided the colloidal stability of the suspension is not compromised. A higher ionic strength decreases the Debye length κ^–1^ and thus the effective size and shape of the CNCs, reducing the distance at which the CNCs can approach each other, allowing them to experience a stronger short-range chiral interaction. In the limit of very low ionic strengths, CNC suspensions with apparent nematic order have been reported, with a measurable pitch appearing upon the addition of only 0.1 mM NaCl.^103^ This observation suggested that the CNC morphology can be compared to twisted ribbons interacting as chiral objects, which naturally form cholesteric phases unless the electrostatic repulsions at very low ionic strength make their chiral shape undistinguishable from an achiral spherocylindrical particle.

Two unusual observations of pitch and ionic strength correlations are worth mentioning. The first one is a nonmonotonic variation reported by Hirai et al., where the pitch first decreased and then increased as more salt was added.^104^ In this case, the cause is unclear, and the partial aggregation of CNCs into larger objects could be a possible scenario.^311^ The second observation was reported by Azzam et al. on CNCs stabilized by grafting of the polymer Jeffamine, where the pitch was found smaller than in their nonfunctionalized counter-parts, and the addition of NaCl led to a clear pitch increase.^306^ A first possible reason is the salt-sensitive LCST of Jeffamine (around 80 °C, though far from room temperature). A more interesting explanation is that, since the CNCs are also sterically stabilized by the grafted polymers,^312^ the ionic strength may reduce the magnitude of the chiral interaction without affecting their effective shape. By decoupling the two effects of ionic strength, this could reveal a general phenomenon that is otherwise masked in charged CNCs by the dominant effect of how salt influences the minimal distance CNCs can approach each other. In this context, analogous studies on fd virus suspensions may provide insights.^297,313^

The application of high-intensity ultrasonication to CNC suspensions causes particle fragmentation and leads to an increase in cholesteric pitch (as discussed further in section 8.2.2).^89,314^ Notably, this pitch increase occurs despite the apparent release of ions into the suspension upon ultrasonication, which would usually be expected to lead to a pitch decrease as discussed above. As mentioned above, it is also possible to permanently modify the effective CNC morphology by polymer grafting or adsoption and still observe cholesteric ordering (see section 8.2.1 for further discussion), although the presence of the coating has been shown to modify the *p*(Φ) power law behavior.^274,276,306^

Useful insights into the self-assembly of CNCs can also be gleaned from their collective behavior in apolar solvents.^268,315^ In these systems (discussed further in section 8.3.3), the onset of the biphasic region occurs at much higher particle mass fraction than in aqueous CNC suspensions, and even when estimating the volume fraction of CNCs (excluding the surrounding surfactant layer), the effective volume fraction appears to be higher than in aqueous CNC suspensions.^202^ The low dielectric permittivity (*ε*_*r*_ < 3) of these apolar solvents leads to the collapse of counterions onto the CNC surface so that the electrostatic interaction between CNCs is negligible. Furthermore, the van der Waals attraction (long-range dispersion forces) between CNCs is reduced due to better index matching between the CNCs and the solvent. In this system, the effects of particle shape and higher-order interactions are therefore dominant and it can therefore be readily compared to hard-particle simulations.^273^

#### 3.3.3 Proposed Mechanisms for Chirality Transfer in CNC Cholesterics

The mechanisms to consider to explain the chirality transfer require both an emergence of chirality from the molecular to the CNC level, and from the individual CNC particles to their collective chiral behavior.

##### 3.3.3.1 From a Molecular Chirality to a Chiral Shape

As discussed in section 2.3.1, microfibrils and crystallites of cellulose I have a right-handed longitudinal twist arising from the parallel packing of cellulose chains. This morphological twist is therefore widely considered to be the “ultimate” origin of mesophase chirality in CNC suspensions. However, chiral assemblies of cellulosic fibers are often found in the cell walls of some plants. The formation of these assemblies could be spontaneous or biologically driven, and different plants present different chiral arrangements. The chirality of CNCs extracted from such sources could thus be solely due to the tendency for their constituents (the cellulose crystallites) to individually twist spontaneously, or it could also be inherited from the hierarchical organization of cellulose in the source material. At the same time, microscale structural chirality of the individual particles is not necessarily sufficient to produce macroscopic chirality, for example, it has been shown that rod-like fd viruses have a chiral morphology and form cholesteric phases, whereas suspensions of the Pf1 virus, which also have a chiral morphology, exhibit nematic ordering without a discernible pitch.^248^

The polycrystallite nature of the CNCs, being made of several elementary crystallites bundled together, can enhance their individual twisted nature and result in a twisted hard-bundle model of dimensions *L* ≫ *W* > *T*. It has recently been proposed that these crystallite “bundles” have a chiral propeller-like morphology.^89^ In that case, the key morphological feature, beyond the particle microscopic pitch, is how much the particle shape deviates from a twisted cylinder, which is achiral. This effect can be captured by a higher cross-sectional aspect ratio *a*_XS_ = *W*/*T* of the particles.^89^ While quantitative analysis of the 3D morphology of these bundled CNCs is still lacking, it is clear that poly crystallite particles play an important role in CNC chiral self-assembly.

##### 3.3.3.2 From a Chiral Shape to a Chiral Assembly

First, a chiral steric interaction due to a twisted particle shape is sufficient to lead to a cholesteric mesophase. In the highly idealized case of hard screw-like particles, Straley was the first to show that a twist away from perfect alignment enabled denser screw packing, with the handedness and twist angle determined by the screw morphology.^316^ However, Straley found that the pitch is expected to be independent of particle volume fraction (i.e., ν = 0), as the chiral strength scales as *K*_t_ ∝ Φ^2^*S*_2_(Φ) and the twist elastic constant was assumed to scale as *K*_22_ ∝ Φ^2^ (although this assumption is in disagreement with some models for rigid rod-like nematics,^244,252,253^). Alternatively, based on generic scaling arguments, Odijk found that the cholesteric pitch of an assembly of hard screw-like particles is inversely proportional to the concentration, namely *p* ∝ Φ^–1^ (i.e., ν = 1).^317^ Using generalized Straley-Onsager theory,^318^ Wensink et al. also predicted ν = 1 for rod-like particles, but with a pitch leveling off at higher volume fraction. In simulations, both density functional theory (DFT) and Monte Carlo (MC) methods applied to hard single-stranded helices suggested that there is no universal power law behavior for pitch versus concentration.^319^ A similar approach was used to simulate chiral “hard bundle” particles, intending to mimic CNC suspensions in apolar solvent, which also concluded that the pitch did not follow a clear power law variation throughout the entire volume fraction range.^273^ However, at the onset of the cholesteric phase, the data were found to follow an empiric linear fit of the form *p* ∝ (Φ – Φ_offset_)^−1^, where Φ_offset_ < Φ_*b*1_ is a fitting constant, similar to that proposed for aqueous CNC suspensions.^272^ In terms of power law behavior, this fit suggests that ν > 1 initially and then decreases to a value <1 at higher volume fraction.

Another consideration is the helical twisting power (HTP), which is usually defined for chiral dopants when added to achiral liquid crystals (e.g., nematics) to quantify how strongly they induce the formation of a chiral phase (usually a cholesteric). For a low concentration of dopants *x*_d_, the induced pitch is given by *p* = 1/(2*βx*_d_), where β is the HTP.^241^ Since CNCs are not employed together with another achiral nematogen to form cholesterics, it is not immediately clear that the concept of HTP is applicable to CNCs. However, CNCs are polydisperse in size and shape, and it is therefore plausible that the effective HTP therefore varies across the particle population. In particular, particles with more pronounced chiral morphology (e.g., more propeller-like bundles) may promote more effectively a decrease of the pitch. Beyond the morphology of individual particles, the ability for functionally chiral particles to influence their less chiral neighbors also depends on their shape compatibility, and it has recently been proposed that the 3D isoperimetric quotient of the two species must be similar to maximize their ability to influence each other’s alignment.^89,320^

The twisted morphology of cellulose crystallites may also lead to significant chiral interactions at longer range. For instance, a twisted crystallite is expected to have a helical variation in its charge distribution, and Revol et al.^20^ was perhaps the first study to suggest this as the origin of CNC mesophase chirality. A helically varying charge distribution could arise from uneven distribution of charge across the CNC surface (i.e., charges on some crystal faces and not others), and even if the grafting of charged groups were evenly distributed across the surface, the anisotropic crystallite cross-section would also lead to nonuniformity in the charge distribution when uneven counterion condensation is considered. Whatever the cause, the effective charge distribution is expected to have a double helix structure (i.e., twisted ribbon-like or propeller-like) or even quadruple helix structure (i.e., twisted cuboid-like), rather than a single helix structure (i.e., corkscrew-like), for which the expected interaction would be different.^308^ Aside from electrostatic interactions, it is possible that the chiral interactions could arise for chiral dispersion (vdW) forces.^321−326^ Optically anisotropic (i.e., birefringent) materials are expected to have anisotropic dispersion forces, leading to an effective torque,^327^ which can lead to a chiral interaction if the optical anisotropy varies in a helical fashion about the particle axis. The resulting helicoidal variation in the permittivity of individual CNC crystallites could therefore be an additional driving mechanism for chirality transfer;^325^ however, experimental evidence in support of this specific mechanism is lacking.

### 3.4 Kinetic Pathways of Liquid Crystal Formation

#### 3.4.1 Analogy with the Liquid–Gas Phase Transition for Lyotropics

The phase transition from isotropic to anisotropic (nematic) phase, as predicted by Onsager theory, is a first-order process.^263^ In this respect, the isotropic–nematic phase separation is analogous to the liquid–gas transition described by the van der Waals (vdW) equation of gases. Similar to the vdW equation, there is a certain concentration range within which the free energy of the whole suspension is lowest if it separates into two phases of different concentrations and volumes: a dilute “gas” phase (here the isotropic phase) and a denser “liquid” phase (here the anisotropic phase) at a given temperature *T* and osmotic pressure Π. This concentration range is bounded by the two critical volume fractions Φ_*b*1_ and Φ_*b*2_ predicted qualitatively by Onsager: below Φ_*b*1_ only the gas phase is stable, above Φ_*b*2_ only the dense phase is stable, while for Φ_*b*1_ < Φ < Φ_*b*2_ the sample should be biphasic, meaning that it should eventually split into two phases, an isotropic phase at a local volume fraction Φ_*b*1_ and an anisotropic phase at a local volume fraction Φ_*b*2_. At equilibrium, the particles in the two phases have the same chemical potential, meaning that their associated Gibbs free energies per particle are the same. However, at the threshold of these boundaries, metastable states are still possible. For instance, as the suspension crosses the boundary into the biphasic concentration range (from either direction), it will remain monophasic for a certain lag time until nuclei of the second phase appear. This nucleation process is usually facilitated by impurities, but these play a much weaker role in colloidal liquid crystals compared to molecular systems. Once nucleation is initiated, the phase grows from the nuclei to form droplets: for an initially dilute, isotropic suspension, droplets of denser phase form and may coalesce and sediment, while for an initially concentrated, anisotropic phase, droplets of isotropic phase appear, coalesce and eventually migrate to the top of the anisotropic phase. This mechanism is known as “Nucleation and Growth” (NG). In the binodal regime, the nucleation requires an activation energy that, to a first approximation, is simply related to the interfacial tension of the droplet via the Laplace pressure associated with its small size.

Within a smaller range of volume fractions contained within the biphasic range, a monophasic suspension becomes unstable and spontaneously phase-separates without any need for nucleation. To understand why, the liquid–gas analogy is once more useful. For an ideal (non-vdW) gas, the osmotic pressure is Π = Φ*k*_B_*T*/*v*_part_ (with *v*_part_ the volume of an individual particle). In this relation, it can be seen that ∂Π/∂Φ > 0, indicating that an increase in Φ will lead to a monotonic increase in osmotic pressure. Consequently, any transient local increase in concentration causes an increase in osmotic pressure that acts as a restoring force to spread the particles out and dampen the local fluctuation of volume fraction toward its average macroscopic value. However, for a nonideal vdW gas (at sufficiently low temperatures and within a certain range of volume fraction), there is a regime where ∂Π/∂Φ < 0. In this regime, any local fluctuation of Φ will inevitably result in a spontaneous phase separation, e.g., a localized higher Φ would cause a pressure drop and further collapse, while a localized lower Φ would cause a pressure rise and further expansion. Unlike nucleation and growth, this phase separation will occur simultaneously across the entire sample volume, with no time lag. This mechanism, known as “Spinodal Decomposition” (SD), is expected in the region of the phase diagram delimited by the spinodal line, which is located within the binodal regime.

Regardless of the mechanism, a biphasic suspension is expected to evolve toward macroscropic phase separation, facilitated by gravity and interfacial tension between the two phases, with the denser phase sedimenting to the bottom. One may therefore expect that observation of the sample a long time after phase separation would not reveal which pathway was taken. In practice, however, samples with fast internal relaxation times tend to evolve quickly via NG into a collection of tactoids, while SD can give very different patterns that can sometimes be trapped by slow relaxation times and kinetic arrest.^268^

While the liquid–gas analogy holds in many aspects, key differences are found, for instance, when considering the relaxation times associated with the degrees of freedom. While the liquid–gas phase separation is limited by the kinetics of particle translation, the Onsager transition is initiated by changes in particle orientation, which does not require particle transport and can therefore occur more quickly in dense suspensions.^328,329^

In the following, NG and SD will be discussed, each with first a general description for lyotropics and then more specifically for CNC suspensions.

#### 3.4.2 Nucleation and Growth (NG)

##### 3.4.2.1 NG in Lyotropics

A hallmark of the NG pathway is the appearance of cholesteric droplets within the isotropic phase. These droplets are known as **tactoids** (see Figure 11). Historically, tactoids were first reported in suspensions of ribbon-like vanadium pentoxide fibers,^330^ and CNC phase separation was also first observed in the form of tactoids.^20^

Following NG, tactoids are usually between 10 and 100 μm in size, with a lenticular or spindle-like shape that becomes more spherical as they grow in size. The shape is determined by the competition between the elasticity of the nematic phase and its tendency to align, versus the tendency of the surface tension to make the droplet spherical. A theoretical discussion of the evolution in droplet shape and internal director field **n**(**r**) with tactoid size can be found elsewhere,^331^ and its relevance for CNCs is discussed in a recent review article.^18^ The shape of cholesteric tactoids has also been studied in detail for the analogous system of amyloid fibril suspensions.^250,1500^ Interestingly, the evolution of tactoid morphology with size can be used to obtain estimates of the elastic constants of the cholesteric CNC phase.^244^ Notably, CNC tactoids are primarily observed to be homogeneous and do not exhibit bipolar alignment.^18,244^ While they can be ellipsoidal, they are rarely seen to be spindle-shaped, probably because of their moderate aspect ratio,^18^ with the notable exception of bacterial CNCs.^103^

The local alignment of CNCs within a tactoid leads to birefringence, and tactoids can thus be observed by optical microscopy when illuminating in transmission between crossed polarizers. Cholesteric tactoids appear stripy when viewed perpendicular to their helical axis, with a periodicity equal to half the pitch (Figure 11).

In the opposite case of an almost entirely anisotropic suspension, transient droplets of the isotropic phase within the anisotropic bulk are sometimes observed. These droplets are sometimes called “antitactoids” or “atactoids” (an example might be seen in the background in Figure 12).^289^ In general, a biphasic sample that is shaken and then left to relax back to equilibrium can display various shapes of droplets of resuspended cholesteric and isotropic phases into each other. While they could arguably be also termed tactoids and antitactoids, their uncontrolled disturbance and still ongoing relaxation makes their shape analysis less appropriate for the discussion above.

##### 3.4.2.2 Signs of NG in CNC Suspensions

While tactoids were observed long ago in CNC suspensions by optical microscopy,^20,137^ an observation of tactoids in situ within a drying suspension was only recently investigated to understand tactoid dynamics upon film casting.^333^ This was achieved by adding a precursor to the CNC suspensions and then UV-polymerizing to interrupt the evaporation process, followed by high resolution imaging of the tactoids by cross-sectional SEM. This approach was used to observe events such as tactoid coalescence, trapping of disclination lines, sedimentation and reorientation of the bottom cholesteric layer to the most favorable anchoring condition, namely with a helical axis normal to the substrate. These observations thus captured snapshots of the “rain of tactoids” onto the substrate, including early droplets that had already sedimented and merged at the bottom, late droplets that were still sedimenting, and the remaining volume of isotropic phase surrounding them. In a separate study, cross-sectional observations of dry CNC films were combined with optical analysis to demonstrate the existence of a stratification of the CNCs inside a film, where the bottom of the film was a well-aligned, monodomain cholesteric, the middle part was composed of still trapped tactoids whose orientation was not as well aligned, and a top layer with no clear order.^334^ In this description, the tactoids still floating freely are not sensitive to the outer anchoring conditions, and result in a less homogeneous optical response. The structural and optical consequences of this effect will be discussed in detail in section 6.

If given enough time, tactoids merge to form a continuous phase, where the helical axes of the domains slowly relax to adopt a uniform vertical direction. In practice, pausing the solvent evaporation once the cholesteric phase is formed, and then resuming the evaporation, provides sufficient time to anneal many of these defects.^335,336^ As a result, the 2D grain boundaries surrounding regions of mismatching helical axes disappear, leaving a network of persisting disclination lines. In very thin films (≲ 10 μm), these defects are responsible for the appearance of RGB mosaic patterns (see Figure 16 in section 4.2.1.).^337^ In small flat films slowly dried under a layer of oil, most of these defects are gradually expelled or cancel each other over time, as the system approaches the thermodynamic equilibrium state of a single monodomain.^338^

#### 3.4.3 Spinodal Decomposition (SD)

##### 3.4.3.1 SD in Lyotropics

As introduced earlier, the spinodal regime is located inside the binodal boundaries, and does not require a lag time to occur, unlike NG. A sample cannot enter the SD regime without first crossing the binodal boundary, which allows NG to occur first. If it does, the tactoids will deplete the isotropic phase from particles, and the two nascent phases will both evolve away from the spinodal regime, preventing SD. To trigger SD, the sample must therefore be quenched into the spinodal regime faster than the expected nucleation lag time. Observing the SD pathway would require the ability to rapidly increase the volume fraction (i.e., in a time scale shorter than the nucleation lag time), which is challenging to achieve experimentally when concentrating large quantities of suspension. One way to increase the chances of observing SD is to initially induce a single aligned phase by applying an external stimulus, such as a strong flow alignment^339^ or an electric field to an initially biphasic suspension,^249^ then suddenly remove this stimulus and allow the system to relax.^340^ Another possibility is to minimize the sample volumes, as it is easier to evaporate smaller volumes, but hard to monitor in real time. Finally, if the internal relaxation time of the suspension become excessively long (e.g., by adjusting its composition) so as to be approaching an arrested state, it can delay nucleation enough to allow the system to pass the spinodal boundary. While SD has been reported for other elongated colloidal particles, such as PBLG rods,^341^ boehmite rods,^342^ and *fd* viruses,^328,329,340^ it has only been proposed for CNC suspensions in a few cases (as discussed below).

##### 3.4.3.2 Signs of SD in CNC Suspensions

Experimental observations consistent with SD were reported by Elazzouzi in tunicate and Avicel CNC suspensions observed in sealed capillaries.^268^ In this case, SD could arise from either the slowing down of the internal relaxation times in these suspensions or a shear-induced alignment of CNCs upon filling the capillary with these suspensions, or a combination of both. The nature of the SD was estimated on the grounds of the emerging characteristic pattern that was trapped by the slow internal relaxation time of the sample, showing signs of kinetic arrest.

In another study combining rheo-SAXS and SALS, aqueous CNC suspensions where sheared and then allowed to relax and showed various relaxation behaviors.^343^ In the high-shear-rate case, three relaxation times were observed: a fast reassembling of the CNCs into a nematic-like order up to the micron-range (in the first few seconds), then a slower relaxation time which they ascribed, due to the time-delay, to a NG process (up to 15 min), and finally a randomization of the helical axis orientation across the sample (observed within 30 min). While the authors ascribed the fast relaxation time to a possible liquid-crystal characteristic behavior of these colloidal particles, this observation could also potentially be explained by transient SD.

Another possible observation of SD was reported in apolar biphasic CNC suspensions, where the isotropic and the cholesteric phases were first aligned under a strong electric field into a single phase with the director parallel to the field, and then the external field was abruptly removed.^249^ The relaxation of the suspension into a polydomain cholesteric (presumably in local coexistence with some isotropic phase) was monitored by laser diffraction, and showed a strong anisotropic relaxation. Similar observations were reported for *fd* virus suspensions shortly after a zero-shear rate quench.^328^

In other studies, the occurrence of SD has been claimed based on circumstantial evidence in wood pulp CNCs,^344^ and in cotton CNCs.^345^ In the former case, the wood pulp was quenched with salt, which poses the problem of the validity of the description of CNCs as still being stable colloidal particles, since the resulting phase was an attractive arrested nematic hydrogel (described as a “hydroglass” in the associated study). In the latter case, dry cotton films that present clear signs of tactoid formation also exhibit clear boundary between these tactoids and the upper CNC layer covering them, much like in the stratified architecture observed in some CNC films.^334^ However, cross-sectional SEM analysis, coupled with hyper-spectral imaging, revealed that the upper layer was also cholesteric, which poses the question of its self-assembly pathway. The proposed answer is a late SD of the remaining CNCs, triggered by a set of conditions: the smaller aspect ratio of the remaining CNCs, the vertical displacement of the liquid–air interface displacing and forcing the horizontal alignment of CNCs from the isotropic phase, the reduced thickness of the film enabling fast evaporation at the latest stage, and the proximity between spinodal and binodal threshold concentrations in polydisperse rods.^342^

## 4 Collective Alignment of CNCs in Suspension

While the liquid crystalline behavior of CNCs allows for their spontaneous local alignment at the micron range, the quality and the direction of the long-range alignment at much larger scales (i.e., larger than the pitch) is influenced by other phenomena that need to be considered.

In this section, the focus will be placed on the most relevant for film casting, namely the effects of flow, anchoring, elastic constraints, and external fields.

### 4.1 Shear and Elongation Flows

CNCs in stable colloidal suspensions can be locally aligned by flow. Experimentally, the behavior of CNC suspensions under flow are usually studied by rheological experiments, with the extraction of the variation of the storage modulus *G*′ and loss modulus *G′′* with the shear rate.^346^ This can be complemented with the collection of additional visual or structural information,^347^ such as, e.g., cross-polarized optical imaging to assess the birefringence of the phase and its macroscopic rearrangement,^348,349^ or 2D-SAXS or SANS to assess the orientation order parameter of individual CNCs.^285,343,350^

For dilute suspensions, shear alignment causes transient local birefringence and therefore creates visible textures observed between crossed polarizers,^351,352^ which can be used as a sign of a well-dispersed suspension.^353^ This alignment under shear is also responsible for a shear-thinning behavior in the rheological characterization of dilute suspensions, but with minimal structural formation upon shear.^354^ Shear causes a statistical alignment in which CNCs spend, on average, more time along the direction of the flow, but additional behavior such as periodically tumbling, kayaking, log-rolling or wagging is also expected,^355,356^ and as observed experimentally for CNCs^357^ (or fd viruses^339,358^) and supported by theory and simulations.^359^ In contrast, elongational flow, such as that occurring at the inlet of a bottleneck, causes an aligning torque on each CNCs, without tumbling. Moreover, elongation flows can be uniaxial, while shear is by construction always a biaxial geometry (i.e., normal to the fluid flow, there may be differences between the direction parallel or perpendicular to the velocity gradient).^360^ Interestingly, the behavior under purely extentional flows can be extrapolated from the characterization of alignment profiles under shearing flows, which are more accessible experimentally using the common rheo-optical techniques.^361^

For concentrated CNC suspensions, flows are incompatible with maintaining a cholesteric order. The application of shear tends to reduce the viscosity of cholesteric suspensions (rheo-thinning), which is usually interpreted as due to the progressive alignment of cholesteric domains, with unwinding of the cholesteric occurring at higher shear rates.^343,347,362^ An investigation of the relaxation of a shear-aligned nematic-like suspension into an equilibrium cholesteric, observed by POM, postulated the presence of transient twist-bend structure.^363^ From a theoretical point of view, the effect of alignment occurring in cholesterics under various constraints is part of what is called nematodynamics. Cholesterics are notoriously viscoelastic and their flowing behavior can be described by the Ericksen-Leslie-Parodi equations and their refinements, which reach beyond the scope of this review.^364^ The interested reader can find more details in specialized reviews on the rheological behavior of CNC suspension in various regimes.^347,365,366^

An important feature of flow alignment is its transient nature: as the flow is interrupted, the alignment usually relaxes. While in the dilute, isotropic regime the relaxation is complete, the cholesteric suspension usually does not completely relax to a random configuration and retains a memory of the previously applied flows, related to the violation of the Cox–Merz rule observed in rheological measurements.^291,357,367^ This phenomenon can lead to the appearance of specific patterns, either due to liquid crystal relaxation,^339,368^ or phase separation in the case of biphasic samples.^340^ To prevent most of the alignment relaxation, a second transition, termed *kinetic arrest* (as discussed in section 5), should be triggered to prevent the loss of the acquired alignment. Indeed, high shear was reported to lead to the unwinding of the cholesteric phase, together with the alignment of the CNCs parallel to the direction of the flow,^285^ leading to the deposition of aligned CNCs.^109,369−371^ A milder shear applied by applying a circular translational displacement to a Petri dish filled with a CNC suspension led to cholesteric films with more vertically aligned domains.^372^ When the suspension is not colloidally stable, the suspension can exhibit flow above a threshold and undergoes gelation after the flow is suppressed, leaving a permanent record of the flow revealed by the local birefringence, as discussed further in section 5.^344,373^

Similar to shear alignment, the directional application of ultrasound (at 20 kHz) to concentrated suspensions (i.e., those in the anisotropic regime) has been shown to align the constituent CNCs parallel to the sound propagation direction.^362^ In this study, ultrasound and shear were also applied simultaneously in orthogonal directions to a CNC suspension flowing through a capillary, which allowed the transition from shear-induced to ultrasound-induced alignment to be observed.

### 4.2 Anchoring and Geometry

The simplest way to control the alignment in most liquid crystalline systems is to control the anchoring at the boundaries of the sample. This is straightforward for molecular liquid crystals, as various simple surface treatments can be used to induce either homeotropic or planar anchoring (where the director lies either normal or parallel to the surface plane, respectively). Furthermore, for planar anchoring, the rubbing direction imprints a preferred azimuthal orientation within the surface plane. In contrast, it is much more difficult to control the anchoring of colloidal liquid crystals: only planar anchoring has been reported, and its azimuthal orientation cannot easily be controlled (while unidirectional planar anchoring could be expected on grooved surfaces, this has not been explicitly demonstrated). For the case of a colloidal cholesteric, the planar anchoring of the director forces to the helical axis to be perpendicular to the surface (i.e., parallel to the surface normal).

#### 4.2.1 Confinement between Parallel Interfaces

In the standard geometry of a suspension drying on a substrate or in a Petri dish, the region far from the edge is then mostly confined between two parallel interfaces, namely the air–liquid interface at the top and substrate–liquid interface at the bottom, with both favoring a horizontal alignment of the director and thus a vertical alignment of the cholesteric helical axis. As some time is required for tactoids to align and coalesce, such anchoring is most efficient when the evaporation is paused (e.g., by placing and removing sequentially a lid on the dish,^335^ or using a perforated lid^345^). Alternatively, the CNC suspension can be covered with an immiscible fluid (e.g., oil) through which water can slowly diffuse, which also reduces convection flows often occurring near phase boundaries.^338^ Finally, covering a drop of CNC with a coverslip also leads to good degenerated planar anchoring,^374^ although this confinement between two rigid interfaces can affect how the sample contracts after kinetic arrest (see section 6).

**Figure 14:**
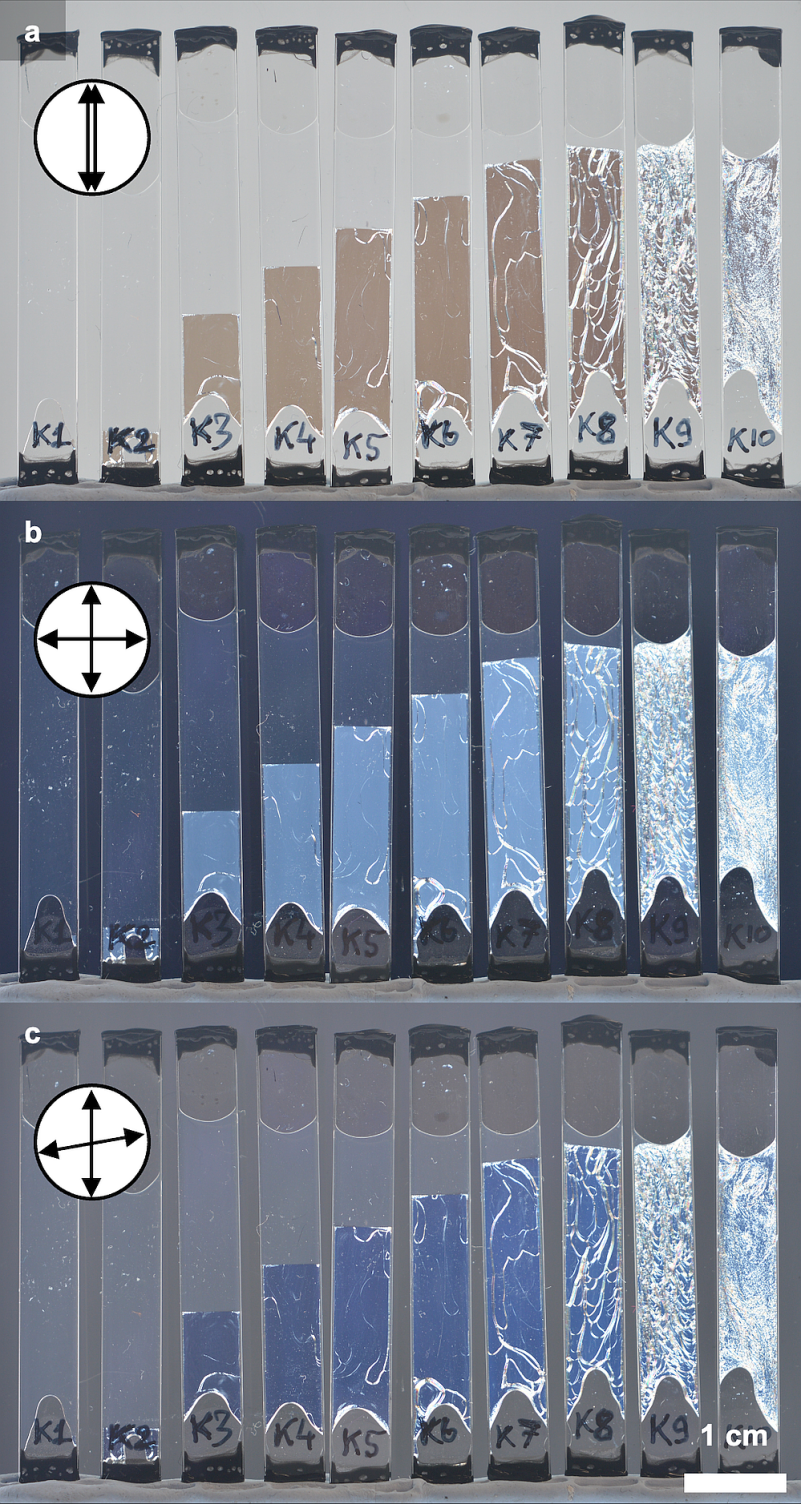
Spontaneous alignment inside flat glass capillaries, known as the Grandjean structure, with defect lines known as “oily streaks”. CNC concentration increases from left to right. The planar anchoring of CNCs at the glass surface causes the helical axis to lie normal to the flat interface, parallel to the viewing direction, which leads to optical rotation (discussed further in section 7.3.3). (a) Between “uncrossed” (parallel) polarizers, the texture appears darker and slightly reddish, while (b) between crossed polarizers, the texture appears brighter and slightly blueish, and (c) between slightly uncrossed polarizers, the background appears dark gray and the cholesteric appears fully dark. Images correspond to a data set (labeled 25 mmol/kg) from ref (161).

**Figure 15:**
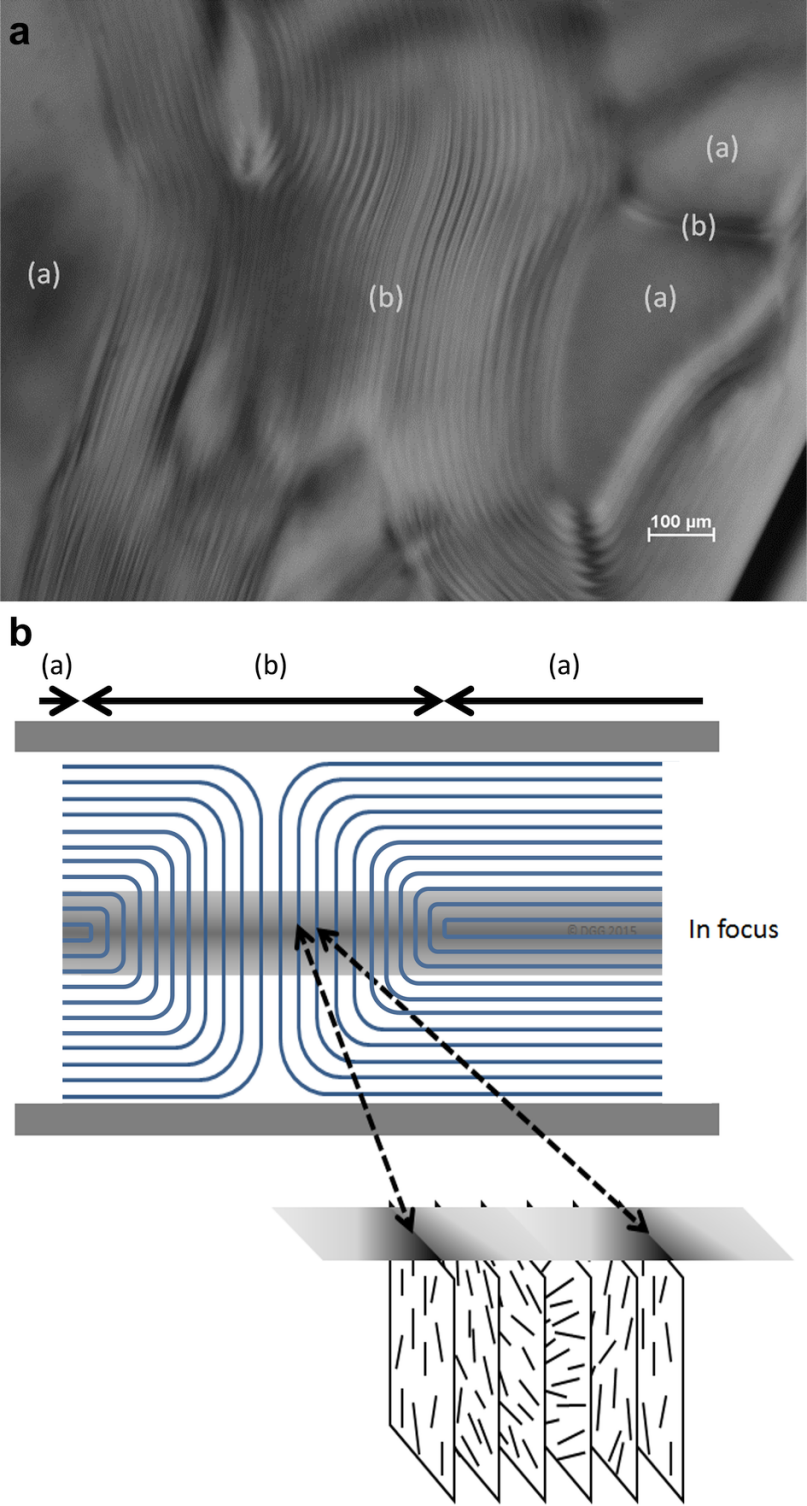
(a) POM image of an aqueous CNC suspension between two parallel glass walls, observed in transmission between crossed polarizers, with (a) regions of planar alignment and (b) fingerprint texture. (b) Schematic cross-sectional view of the same regions (a) and (b), with the gray shaded region corresponding to the imaged plane in POM. Spacing between schematic blue lines represent half-pitch distance as depicted. Adapted with permission from ref (375) under CC-BY. Copyright 2015 The Authors.

In a capillary with a rectangular cross-section, the helical axis tends to align along the direction of the smallest gap (i.e., planar anchoring occurs along the faces of the capillary). The resulting alignment leads again to the Grandjean texture, with defects known as the “oily streaks” running through.^161^ Within these streaks, the helical axis is locally pointing within the sample plane, thus appearing highly birefringent. The streaks are illustrated macroscopically in Figure 14 and microscopically in Figure 15. The oily streaks defects observed in the Grandjean texture are made possible by the existence of disclination lines of various topological nature and charge (usually spaced from one another by a distance of several pitches when they are clearly distinguishable macroscopically, as in Figure 14). While anchoring is eventually effective in aligning the cholesteric helical axis normal to the interface throughout the sample, it is less effective at eliminating defects, as the disclination lines require both a significant activation energy and topological complementarity to meet, merge and cancel each other.

**Figure 16:**
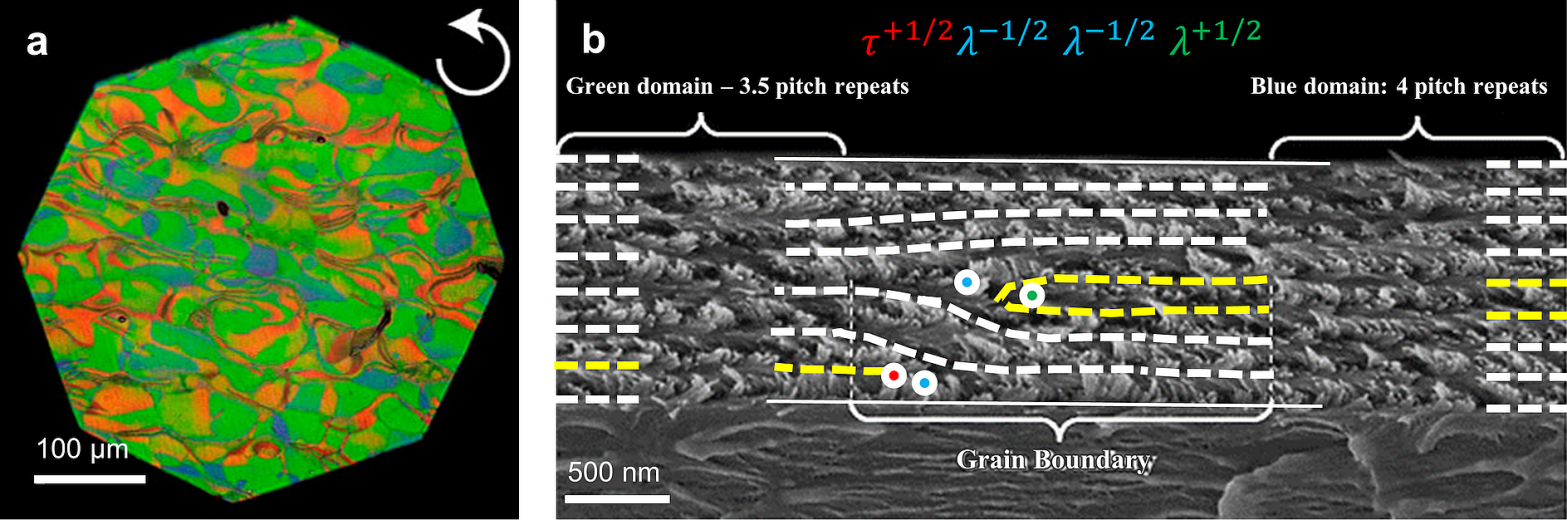
Illustration of the effect of disclination lines on the discrete variation of the pitch in thin uniformly aligned CNC films in planar confinement, observed after drying. (a) Discontinuous mosaic patterns observed via reflection optical microscopy of a CNC film dried slowly in air. (b) SEM cross-section of the grain boundary with overlay of the corresponding disclination lines, indicated here as colored points. Adapted with permission from ref (337) under CC-BY. Copyright 2014 American Chemical Society.

If the gap *h* between the two planar surfaces is small (a situation that can be achieved in thin films dried slowly in air,^337^ under oil,^338^ or between two parallel glass plates^374^), a single pair of discrete disclination lines running through a well-aligned cholesteric region should cause a discontinuous variation of the pitch, as illustrated in CNC films with a planar cholesteric order.^337^ In the following, we propose a more detailed explanation of these observations, based on the topological analysis of disclination lines observed in cholesterics.^259^

If a single pair of discrete disclination lines is separated by a distance *p*/4 (e.g., for two lines of topological charge τ^–1/2^ and λ^+1/2^), the pitch varies discontinuously from *p* = 2*h*/*N* (where *N* does *not* have to be an integer in the case of degenerated planar anchoring) to *p*′ = 2*h*/(*N* ± 1). By combining the two expressions, it can be shown that the pitch variation across the defect is given by
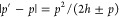
19which is clearly larger for smaller *h* (i.e., a narrow gap leads to bigger jumps in pitch).

Similarly, a pair of disclination lines separated by a distance *p*/2 (e.g., for two lines of topological charge λ^–1/2^ and λ^+1/2^) should lead to *p*′ = 2*h*/(*N* ± 2) and a pitch jump of |*p*′ – *p*| = *p*^2^/(2*h* ± 2*p*). This second type of defect is much more stable and requires a much higher activation energy to merge and cancel with a complementary defect, which usually results in stabilized oily streaks.

While these defects form in the suspension state (as discussed in section 3.1.3), examples of large discontinuous pitch variations in CNC systems have been reported in dried films, where they lead to striking structural color patterns (Figure 16). The interested reader can find out more on these disclinations here.^259,376^

Notably, if a small tilt is introduced between the two interfaces to make the height *h* increase linearly with a lateral position, similar periodic defects with regular integer increases of *N* arise, from which the equilibrium value of the pitch can be determined using an expression analogous to (eq 19) (see section 7.3.7 about Grandjean-Cano wedges).^377,378^ While this technique, or its later refinements,^379^ are widely used in the molecular liquid crystal community, no example of its implementation to determine the pitch in CNC suspensions could be found in the lirerature.

#### 4.2.2 Confinement in Other Geometries

In cylindrical capillaries, the planar alignment is imposed symmetrically and leads to a radial alignment of the helical axis with a core defect (corresponding to a disclination line λ^+1^ if fully cholesteric, or to an isotropic core).^380,381^ It is unclear whether cylindrical geometries, for which the curvature is not degenerated within the interface plane, leads to nondegenerated anchoring, but the example of corrugations such as on a grating should impart some azimuthal component to anchoring that should be curvature-sensitive.

**Figure 17:**
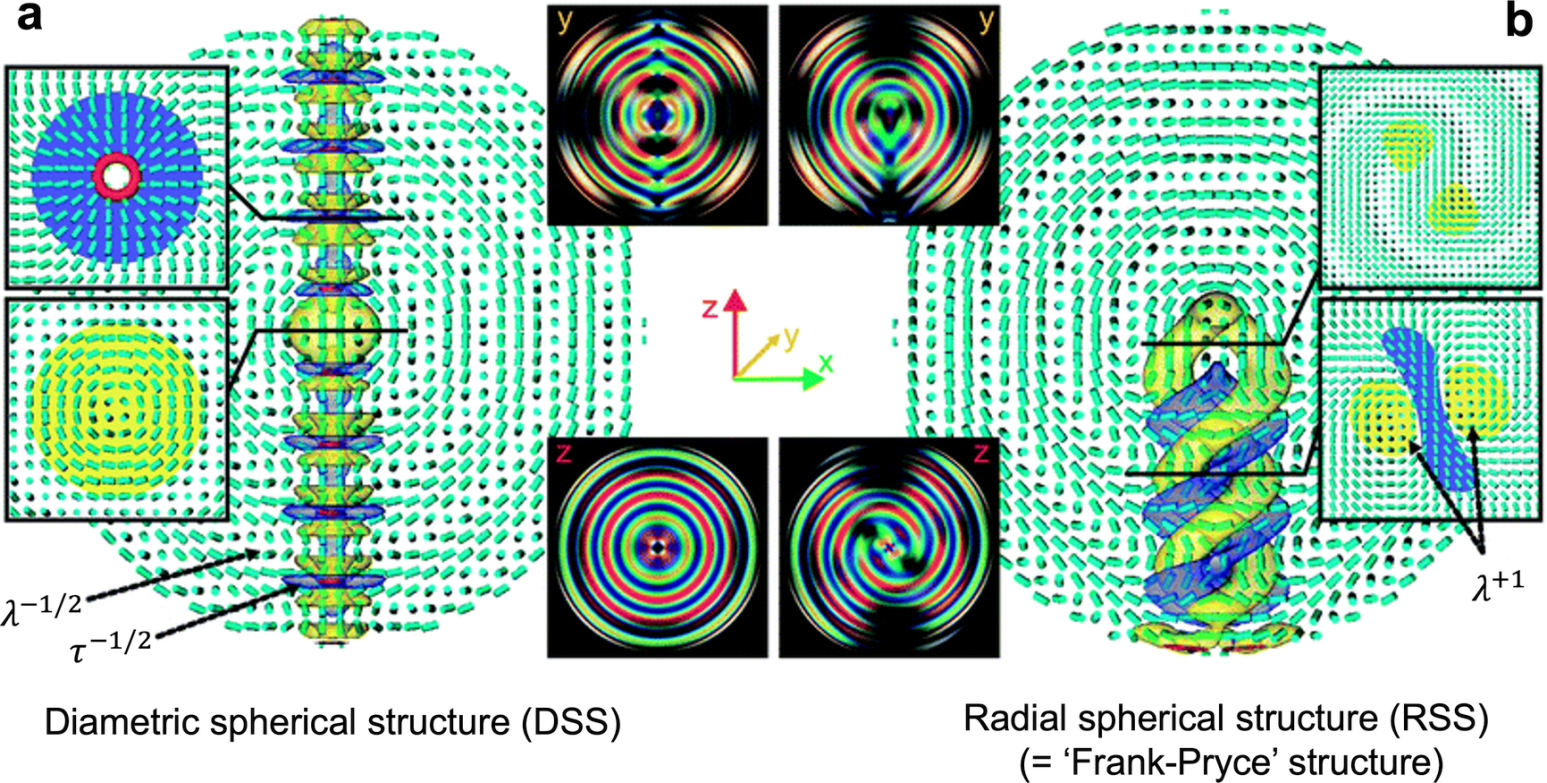
Two most stable “Frank-Pryce-like” (FP-like) structures in cholesteric droplets for larger droplets (diameter Ø ≥ 4p), numerically calculated for (a) the diametric spherical structure (DSS) and (b) the radial spherical structure (RSS), also known as the original “Frank-Pryce” structure. Adapted with permission from ref (258). Copyright 2012 The Royal Society of Chemistry.

Finally, the case of the confinement within a spherical droplet leads to a frustrated configuration since such anchoring has to generate two topological defects at the surface. If the pitch is much smaller than the droplet diameter, a radially aligned Frank-Pryce structure is formed (Figure 17),^258,288,307,382−384^ in which the radial alignment of the helical axis propagates the cholesteric order inward toward an isotropic core defect, along with two disclinations lines connecting the surface defects to the core. The role of curvature on liquid crystal arrangements in spherical and cylindrical confinement is reviewed in detail elsewhere.^245^

### 4.3 Elastic Instabilities

**Figure 18:**
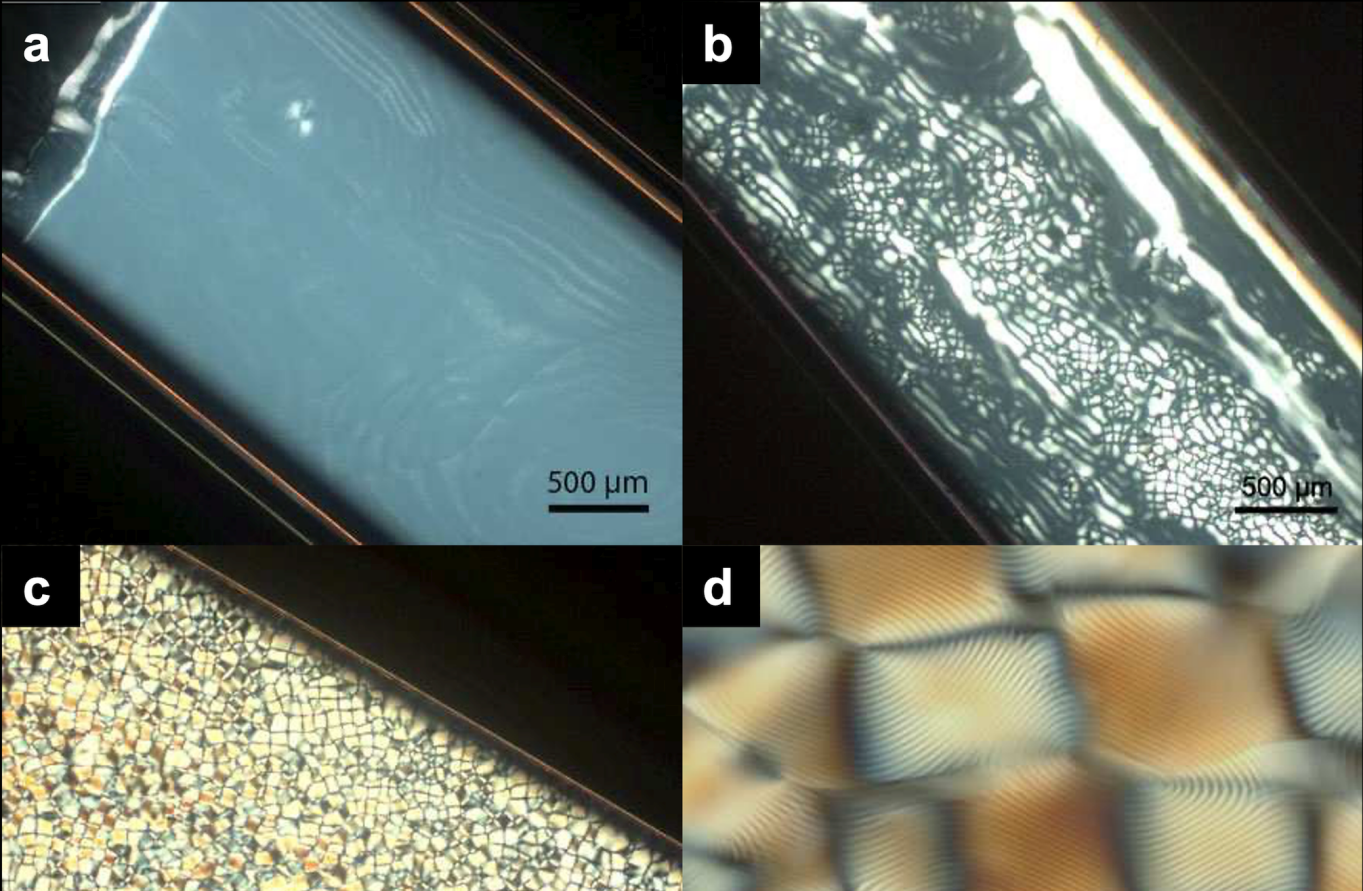
POM (between crossed polarizers) of aqueous CNC suspensions stored in glass capillaries (from cotton, 8.8 wt %, acid form), (a) imaged after six months, (b) same capillary, after six additional months. (c) same capillary, after an additional week at 60 °C, (d) same capillary as in (c), at higher magnification. Scale bars in (a–c): 500 μm, in (d): 50 μm. Adapted from ref (268). Copyright 2006 CERMAV.

**Figure 19:**
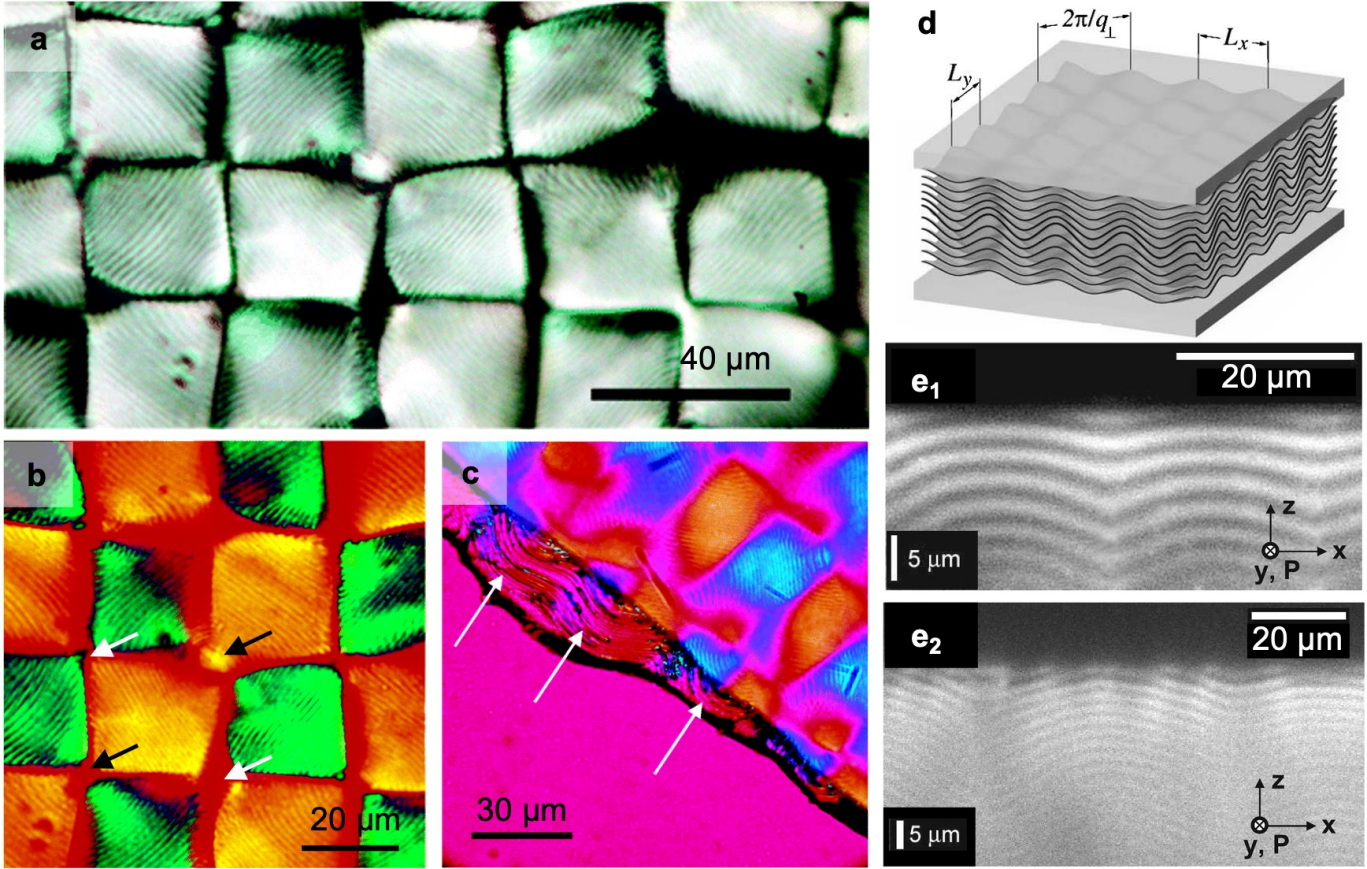
Checkerboard pattern observed in a CNC film obtained upon suspension evaporation. (a) POM image observed between crossed polarizers. (b) POM using an additional full-wave plate to discriminate different directions of alignment (black arrows describe type-1 defects, white arrows type-2 defects, see text for detail). (c) Same as in (b), where the cross-section is also visible in a film fracture and reveals undulation (white arrows). (d, e) Helfrich-Hurault (HH) instabilities observed in confined molecular cholesteric, subject to planar confinement (favoring a horizontal director). (d) Schematic of the HH instability. (e) Side view of the instability using fluorescence confocal polarizing microscopy, in the presence of either, e_1_, a strong anchoring or, e_2_, a weak anchoring. The undulation does not contain focal conics, which develop only at higher fields. (a–c) Adapted with permission from ref (388). Copyright 2005 American Chemical Society. (d, e) Adapted with permission from ref (391). Copyright 2006 American Physical Society.

Elastic instabilities are a general class of phenomena that can also occur in liquid crystals under situations of frustrated constraints, such as conflicting bulk elasticity and interfacial conditions. A notable example is the Helfrich-Hurault (HH) instability,^385,386^ which usually arises when an external field is applied to a cholesteric in the presence of strong anchoring and confinement conditions. However, the observation of similar HH instabilities can be triggered by the presence of deformable interfaces and variable boundary conditions, as comprehensively described in a recent review.^387^ In particular, CNC suspensions in flat capillaries subjected to heat treatment exhibited “checkerboard” patterns (Figure 18),^268^ with similar patterns seen in solid films after drying (Figure 19a−c).^374,388^ These structures are otherwise known in cholesteric phases as *quadratic polygonal field textures*.^389,390^

In the case reported by Elazzouzi,^268^ a sealed capillary of suspension was left to equilibrate for six months, resulting in a uniform texture that indicates planar anchoring of the CNCs at the glass interfaces (Figure 18a). The appearance of the checkerboard pattern after a further six months (Figure 18b) is thus intriguing. Heat treatment of the sealed capillary at 60 °C for about a week led to even stronger development of this second conformation (Figure 18c). The presence of a fingerprint texture within the checkerboard pattern confirmed that a cholesteric phase was still present (Figure 18d). Although no interpretation was proposed in the original study, the most convincing explanation is a heat induced desulfation of the CNCs (discussed further in section 8.2.1), which would increase the ionic strength of the suspension. The resulting pitch contraction under confinement could then induce a HH instability. Notably, desulfation is known to take months at room temperature but only several hours at 60 °C, consistent with the experimental observations.

In the case reported by Roman and Gray,^388^ the authors did not speculate on the mechanism, but their observations can also be explained by a pitch contraction under confinement as the suspension dries, leading to a HH instability. The analysis of the checkerboard structure proposed by the authors reveals two types of defects at the corner of each square: on one hand, two diagonally opposite corners are made of focal conics of opposite orientation (with respect to the vertical axis), relabeled in this review as type-1 defects (black arrows on Figure 19b), and on the other hand two other diagonally opposite corners are the location of two other defect regions, here referred to as type-2 defects (white arrows on Figure 19b).

To explain the emergence of a HH instability upon drying in a shallow dish, the drying CNC suspension can be considered as confined between two horizontal interfaces (substrate-suspension and suspension-air) of initial separation *h*_init_, with uniform cholesteric order of initial pitch *p*_init_ and planar anchoring (i.e., with a vertical helical axis, normal to both interfaces separated). Upon solvent evaporation, the water that evaporated increases the CNC volume fraction from Φ_init_ to Φ > Φ_init_ and displaces the free air-suspension interface downward, from *h*_init_ to . However, the higher CNC volume fraction also causes the equilibrium pitch value to decrease, usually as a nonlinear function of their concentration. As already discussed in section 3.3.2, a power law with an exponent ν ≥ 1 is often observed, giving . For any value of ν > 1, one can deduce that upon increasing Φ, the pitch *p* will decrease faster than the height *h*. As a result, the number of pitches (full turns of the cholesteric structure) within the height *h* increases with concentration as
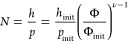
20

Pitch relaxation is a collective mechanism requiring cooperation, and the faster the evaporation and the thicker the initial height *h*_init_, the more turns the cholesteric would be required to wind up to keep up with minimizing its bulk elastic free energy, while the concentration increase usually comes with a higher viscosity. This suggests that in practice, the pitch of a monodomain cholesteric confined between such interfaces would lag behind its equilibrium pitch and build up twist elastic energy, as if the cholesteric was actually stretched and not compressed, which might appear counterintuitive (in reality, it is unwound compared to its equilibrium state). This also explains why in the first example with sealed capillaries, aged and heat-treated samples developed the instability. They could also explain the pitch difference reported between polydomain suspension in large capillaries and the monodomain Frank-Pryce-like droplets prior to kinetic arrest.^288^ This explanation was also recently proposed to account for highly buckled structures in dried cholesteric solutions of hydroxypropyl cellulose,^392,393^ a system for which 2.5 ≤ ν ≤ 4. Importantly, at the onset of the HH instability there are no focal conics, but rather a simple undulation of the cholesteric helical axis, which is exactly the structure visible in the reported cross-sectional view (Figure 19c) and much different from the structure proposed in ref (388).

Instead of long-range winding of the cholesteric across the sample cross-section, a faster relaxation mechanism is to tilt the local helical axis with respect to the thickness direction, and to revert the direction of the tilt periodically, as shown in Figure 19d. This instability has a lateral periodicity (wavelength) *λ*_HH_ given by^394^
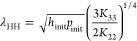
21

In this expression, *K*_22_ and *K*_33_ are Frank elastic constants of the cholesteric. This wavelength is influenced by the strength of anchoring and can evolve from its value at the instability threshold, as documented elsewhere.^391^ However, this simple relationship for *λ*_HH_ is in fairly good agreement with the observed periodicity of the checkerboard patterns in the two examples given above. To make a quantitative comparison, only a crude estimate of the ratio *K*_33_/*K*_22_ is required, due to its dependence with a weak power law. This ratio can be estimated for CNC suspensions, with values of *K*_33_/*K*_22_ varying from 3.85 to 115, depending on the derivation methods (i.e., a ratio (*K*_33_/*K*_22_)^1/4^ from 1.4 to 3.3).^244^ Applying this to the first example in the capillary (with *h*_init_ = 200 μm and *p*_init_ = 17 μm) and assuming a ratio *K*_33_/*K*_22_ ≈ 40, we find that *λ*_HH_ ≈ 160 μm, which overestimates slightly the periodicity of the checkerboard pattern (about 100–120 μm). To apply this to the second example in the dried film, one needs to first assume the initial height *h*_init_ before the suspension solidified, as well as the pitch. Assuming the instability happened at about 10 wt % ≈ 6 vol %, we find that *h*_init_ = 22 μm/0.006 ≈ 360 μm and, combining it with *p*_init_ = 3 μm (assuming it crudely from the periodicity of the final pattern in top view as *p*_init_ = 2Δ), we find that *λ*_HH_ ≈ 90 μm, again overestimating slightly experimental observations (50–60 μm). This overestimation can come from the evolution from the threshold value already mentioned,^391^ or, as discussed in section 6.1.2, might be related to the misinterpretation of the top-view periodicity Δ as being half the pitch, since the real pitch *p*_init_ = 2Δ*s*in θ, with θ being the tilt angle between the helical axis and the viewing direction, clearly apparent in Figure 19c,e_2_. For tilts θ = 30°, we find that *p*_init_ = Δ, and thus *λ*_HH_ ≈ 115 μm for the first example in the capillary, and 65 μm for the second example in the dried film, both consistent with the reported periodicities.

### 4.4 External Fields

Active control of CNC collective alignment can also be achieved using external fields. In the following section, we discuss the use of electric and magnetic fields in various settings and geometries.

#### 4.4.1 Electric Fields

External electric fields, both AC and DC, have been used to align dilute CNC suspensions (i.e., initially isotropic suspensions with negligible particle interactions) parallel to one another in the direction of the field, or to manipulate cholesteric suspensions of CNCs both in water or in apolar solvents. The use of electric fields gives interesting insights into the coupling between individual CNCs and collective phenomena, but it remains mostly relevant to manipulate suspensions rather than to produce oriented films, as drying phenomena in the presence of an electric field causes several practical difficulties (geometric constraints, electro-wetting, ionic transport, water splitting, etc.).^395^ In this section, we will thus only give a brief overview of the coupling between CNCs and external electric fields.

Dilute CNC suspensions exposed to external electric fields behave as a paranematic, i.e., CNCs tend to align parallel to one another in the direction of the field, with an associated quadrupolar orientation order *S*_2_ = ⟨3 cos^2^ θ – 1⟩/2 > 0 (Here, the brackets indicate average quantities, and θ is the angle between the particle long axis and the reference direction). Due to the limitations of high voltages in water (i.e., water splitting occurs above 1.23 V), strong electric fields were applied either with small voltages between small electric gaps of few microns,^396^ or with high voltages on the centimeter length scales but this required using CNC suspensions in apolar solvents.^220,397,398^ Transient electric birefringence (TEB) experiments, consisting of applying an abrupt reversal of the direction of a weak DC electric field and monitoring the transient birefringent relaxation as a sign of the reorientation of the CNC alignment, were applied to CNC suspensions in apolar solvents (i.e., toluene, in the presence of BNA surfactant) and showed that such relaxation does occur.^220^ This transient relaxation is expected for rod-like particles with a permanent electric dipole (alongside any induced dipole), while a purely induced dipole is not expected to lead to any transient relaxation.^399,400^ This suggests a dipolar orientation order *S*_1_ = ⟨cos θ⟩ > 0 in addition to a quadrupolar orientation order *S*_2_ > 0. However, complex polarization mechanisms may also occur in colloidal systems,^401,402^ including slow polarization that can lead to an apparent permanent dipole signature.^403^ The absence of centrosymmetry of CNCs and in cellulose I allomorphs, as well as the strong molecular dipole of glucose, suggests a real permanent dipole moment is expected along the chain axis, and that the measured permanent dipole is genuine. From the alignment dependence at low fields, a saturation of the alignment for cotton CNCs is expected around 20 kV/cm.^220^

While AC fields enable steady state control of the alignment of CNCs, DC fields can lead to an accumulation of charge, among other effects (particle migration, flow, aggregation, etc.) that can compromise the colloidal stability of the suspensions, so they are usually applied in short pulses. Electric fields can cause CNC transport by electrophoresis (particles migration proportional to their net effective charge and the applied field) and dielectrophoresis (particle migration proportional to their polarizability and local field gradient). Note that electrodeposition (i.e., collapse of charged colloidal particles onto an oppositely charged electrode) is voltage-dependent rather than field-dependent, and is discussed in the context of cholesteric CNC suspensions in section 9.3.2.

Concentrated CNC suspensions (initially in a cholesteric or biphasic phase) exposed to external electric fields have also been investigated both in aqueous and apolar solvents. These suspensions were usually studied in large cells where anchoring phenomena were not dominating on the time scale of the experiments, or on freely floating tactoids where the surrounding isotropic phase did not impart any preferred orientation.

In apolar solvents (e.g., toluene, with BNA surfactant), application of electric fields to polydomain cholesteric CNC suspensions first causes a reorientation of the domains, so that the helical axes point perpendicular to the external AC field. At higher field strengths, the cholesteric order within each domain is distorted, so that a growing proportion of its director aligns toward the electric field, leading to a progressive unwinding of the cholesteric. Above a critical field *E*_*c*_ (typically between 0.25 kV/cm to 0.60 kV/cm rms at 1 kHz), the cholesteric is completely unwound, and this threshold can be used to estimate the Frank elastic twist constant *K*_22_, provided the local anisotropic susceptibility of the suspension (i.e., parallel vs perpendicular to the director) is known.^249^ If the applied field is initially above *E*_*c*_ and is then abruptly removed (within a second), the sample relaxes within a few minutes into a myriad of disoriented small cholesteric domains that produce a macroscopically uniform texture across the sample. In contrast, slowly decreasing the applied field from above *E*_*c*_ to almost 0 kV/cm (decreasing progressively over 1 h) allows for a progressive winding-up of the CNCs into a polydomain cholesteric structure with a helical alignment perpendicular to the field (i.e., degenerated in the plane perpendicular to the field), and therefore does not lead to the formation of a uniform cholesteric domain.^249^ Intriguingly, the appearance of periodic patterns in apolar CNC suspensions was reported at much higher AC fields (≳ 4.6 kV/cm at 1 kHz) and attributed to electrohydrodynamic convection instabilities.^404^

For biphasic aqueous CNC suspensions exposed to external electric fields, different alignment directions were observed for freely floating tactoids, depending on the frequency of the applied AC field.^405,406^ At higher frequencies (25 kHz), an alignment analogous to apolar suspensions was observed, with helical alignment perpendicular to the field, prolate tactoid distortion along the field direction and an increase in pitch. At lower frequencies (100 Hz), the opposite behavior was observed, with the helical axis reorienting to lie parallel to the field, an oblate contraction of the tactoids along the helical axis and a decrease in pitch. This peculiar low-frequency behavior was not explained by the authors, but is reminiscent of other past observations on charged colloids.^407−409^ The observation of this behavior only at lower frequency suggests that Maxwell–Wagner–Sillars polarization, involving charge transport by ions, may play a crucial role. Indeed, similar inversions of the sign of the induced birefringence (via the Kerr constant) have been reported on flexible polyelectrolyte solutions, which suggests that the condensed counterions give a positive contribution to the birefringence and free counterions a negative one.^401^

#### 4.4.2 Magnetic Fields

Magnetic fields have also been used to induce some degree of alignment of CNCs, for both initially isotropic and cholesteric suspensions. In both cases, the CNCs tend to align their long axis perpendicular to the field direction, due to the small anisotropy of cellulose diamagnetic susceptibility. Alignment can also be achieved by adding ferromagnetic or paramagnetic material in the CNC suspension, either by grafting it onto the CNCs or by adding species that align more easily in a magnetic field and which induce indirectly an alignment of the CNCs by anisotropic steric effects. Regardless of the presence of added magnetic particles, an important advantage of magnetic field alignment compared to electric fields is the relative absence of other transport effects on diamagnetic materials, allowing for a much easier control of the deposition upon drying.^161^ A selection of recent examples is discussed below.

##### 4.4.2.1 Suspensions of CNCs Only

The magnetic alignment of cellulose microfibrils has been first reported on tunicate CNCs dried under a magnetic field of μ_0_*H* = 7 T.^222^ While these fibers were dried and could also have aligned by the anisotropic compression upon drying (see section 6.1), microdiffraction analysis showed that their axis [100] (i.e., one of the two short directions of the CNCs, alongside [010] for cellulose Iβ) was pointing parallel to the field, which would not be expected simply in the case of a drying-induced alignment. The alignment of [100] parallel to the field also implies that both [010] and [001] axes (the latter being the CNC long axis) were perpendicular to the field.

For dilute CNC suspensions (i.e., suspensions that are initially isotropic with negligible particle interactions) in an applied magnetic field, there is a balance between the Zeeman energy of each rod, arising from the anisotropy of the diamagnetic susceptibility of cellulose and favoring alignment of the CNCs perpendicular to the field direction, and the thermal energy, which randomizes the CNC orientation. This balance was quantified experimentally by measuring the birefringence induced in a CNC suspension from tunicates (about 0.5–5 μm long) in a constant magnetic field (known as the Cotton-Mouton effect).^223^ Field-induced antinematic ordering was observed, with an order parameter of *S*_2_ ≈ – 0.45 for applied fields close to 18 T (*S*_2_ = −0.5 being the case for a perfect antinematic order), with an estimated diamagnetic susceptibility of Δχ^CNC^ ≈ 0.95 × 10^–5^. The Laplace additivity rule (used to estimate the diamagnetic anisotropy of cellulose Iβ by summing up the components of all the diamagnetic unidirectional anisotropies of each covalent bond in each crystallographic directions) led to an estimation of Δχ^CNC^ ≈ – 0.68 × 10^–5^, and indicated that the alignment of cellulose was expected to arise from both a preferential alignment of the [100] axis parallel to the field and of the [001] axis away from it.^223^ Smaller CNCs from other sources (cotton, wood) or even ChNCs (from crab chitin, also aligning with *S*_2_ < 0) did not reach alignment saturation at that field magnitude.^223^ Interestingly, rotating magnetic fields (i.e., fields whose direction is rotating within a fixed plane) induce an alignment of the cellulose microcrystals toward the normal to the plane of rotation, thus with a positive order parameter *S*_2_ > 0.^410,411^ Biaxial alignment using elliptically rotating fields has been claimed, but without reporting the X-ray diffraction patterns that would lift all ambiguity.^412^

The exposure of cholesteric CNC suspensions to an external static magnetic field favors an alignment of the helical axis parallel to the field, without causing the distortion or the unwinding of the cholesteric order. Notably, the collective alignment of CNCs within the cholesteric phase aids their magnetic alignment, so much smaller fields can be used, as initially demonstrated by Revol et al. at 7 T with CNCs from cotton or wood pulp,^137^ and later with weaker fields of 0.5–2 T and other CNC sources.^113,161,285,413,414^ Indeed, since their long axes are on average pointing along the director **n**, and their [100] axis point away from **n**, and since **n** is always perpendicular to the helical axis, the magnetic energy of each CNC particle is minimized when the whole cholesteric domains align their helical axes parallel to the field, which does not require distorting the cholesteric order. The magnetic torque acting on the helical axis **m** of the cholesteric results from the contribution of each CNC adding to one another, and thus scales as the tactoid volume V, its CNC volume fraction, and the local *S*_2_ parameter within the cholesteric phase indicating how the direction of the CNCs couples on average with **n**:
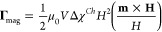

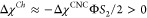


For a cholesteric tactoid of 10 μm diameter, the magnetic torque **Γ**_mag_ should already be several hundreds of *k*_B_*T* and only needs to overcome the friction of the phase surrounding it.^415,416^ For a tactoid floating in an isotropic suspension, this friction is much weaker than in a fully cholesteric polydomain suspension where strong viscoelastic effects occur.^346^ Interestingly, it has also been shown that the cholesteric structure of tunicate CNC suspensions can also be unwound by applying a rotating magnetic field (5 T at 10 rpm), as the field favors the orientation of the CNC long axes [001] in the direction perpendicular to the plane of rotation of the field.^113^

If the field is applied perpendicular to a substrate, magnetic alignment is enhanced by planar anchoring. In contrast, applying the field in a different direction leads to a competition between planar and nonplanar alignment that depends on both the degree of confinement and the magnitude of the field. This usually results in a HH instability (see section 4.3), with a planar configuration at low fields, that is distorted above a threshold field strength that depends on the size of the confinement gap. It is interesting to note that, if a coarse-grained description of the cholesteric order is adopted, in which the helical axis is considered instead of the director, the HH instability is qualitatively analogous to a type of Fréedericksz transition occurring in nematics, in the sense that in both cases a dominating uniform alignment for smaller gaps or weaker fields is observed, while a distorted structure is found for larger gaps or stronger fields.^417^ These effects becomes relevant when investigating the effect of tilted magnetic fields in suspensions as they are left to dry in a dish (see section 9.1.4).^416^

##### 4.4.2.2 Suspensions of CNCs with Magnetic Nanoparticles

Suspensions of CNCs have recently been combined with magnetic nanoparticles and subjected to magnetic fields.^418^ The description of these particles as “magnetic” requires some clarification: while diamagnetism is a weak but general effect occurring in all materials, we use the term “magnetic” to refer to a material presenting any other effect, usually orders of magnetic stronger than diamagnetism, such as ferro- or ferrimagnetism, paramagnetism, antiferromagnetism, etc. Two main cases should be distinguished. In the first case, the magnetic nanoparticles are attached onto the CNCs, while in the second, they are freely moving and form a colloidally stable species alongside the CNCs.

In the first case, the magnetic particles were usually nucleated onto the CNC surface and produced particles that were either unable to form a cholesteric,^419^ or provided weak alignment even in fields as high as 4 T.^420^ These magnetically grafted CNCs were also combined with regular CNCs, but without much improvement of the uniformity or optical response.^421^

In the second case, where the presence of freely moving and colloidally stable magnetic nanoparticles, such as those constitutive of a ferrofluid, much more interesting phenomena were observed. These magnetic nanoparticles are usually negatively charged and can be simply mixed into the CNC suspension without aggregating or precipitating. In biphasic suspension, it has been shown that smaller nanoparticles (Ø ≈ 50 nm) tend to mix well within the cholesteric phase, while larger particles (Ø ≈ 180 nm) are expelled from the cholesteric phase and accumulate in the isotropic phase.^307,422^ Magnetic nanoparticles typically fall into the range of smaller nanoparticles, but can nevertheless preferentially enter the isotropic phase in a biphasic CNC suspension. Consequently, the application of a moderate magnetic field gradient (μ_0_ ∇ *H* ≈ 10 T/m), which can feasibly be achieved using commercially available NdFeB magnets, has been shown to attract the isotropic phase (rich in magnetic nanoparticles) at the expense of the CNC-rich cholesteric phase.^423^ A striking demonstration of this effect was the magnetically induced inversion of the isotropic and cholesteric phases. Another effect reported in the same work was the orientation of the cholesteric phase so that the helical axis lies perpendicular to the magnetic field. The authors identified that the alignment mechanism must be of a different origin from the usual CNC alignment in external magnetic fields, but without offering a clear alternative.

More recently, the coassembly of bacterial CNCs with freely moving Fe_3_O_4_ particles was shown to exhibit unwinding of the cholesteric order under moderate magnetic fields (μ_0_*H* ≤ 250 mT) and in the presence of field gradient (no value reported).^424,425^ A phase separation between CNCs and magnetic nanoparticles was also reported by the local field gradient, including in resulting cast films. The proposed mechanism for the induced nematic-like alignment of CNCs was a flow-alignment induced by the magnetic field gradient.^424^ Since this scenario requires a gradient and a transport mechanism, it could also explain the previous observations from ref (423); however, it would not be sufficient to explain similar alignment in a spatially uniform magnetic field, as described next.

The alignment of the cholesteric axis perpendicular to the magnetic field mentioned above (in ref (423) is very similar to the observation from another study, where rod-like (prismatic) paramagnetic nanoparticles of lepidocrocite (LpN) were added to cholesteric CNC suspensions in various proportions, and the orientation of the long axis of the CNCs was monitored by 2D-SANS under uniform magnetic fields (1–6.8 T), and using contrast-matching to only detect the CNC contribution.^426^ In this second case, the cholesteric phase was preserved (reported for 1% LpN, while suspensions at 10% LpN showed signs of demixing, and the cholesteric order was not explicitly reported by the authors). The authors reported that for 10 vol % of added LpN (CNC-LpN 10%), the orientation of the CNCs was first pointing parallel to the field (*S*_2_ > 0 for μ_0_*H* ≲ 2 T) and then evolved to a perpendicular alignment at much higher fields (*S*_2_ < 0 for μ_0_*H* ≲ 2 T). The orientation mechanism proposed by the authors is that the paramagnetic LpN are initially more responsive in weaker fields and contribute to the parallel alignment, however as the field becomes stronger they are out-numbered by the CNCs that tend to align perpendicular to the applied field, which prevails at higher fields.

The examples discussed above suggest a cooperative effect between CNCs and free magnetic particles leading to the alignment of CNCs parallel to the magnetic field, even without magnetic field gradient. Similar effects were also found in other systems and could better explain the alignment phenomena reported above. As a notable analogous example, rod-like particles (*L* ≈ 230 ± 70 nm, *D* ≈ 46 ± 20 nm) were aligned by the effect of a uniform magnetic field in the presence of magnetite (Fe_3_O_4_) nanoparticles of ca. 10 nm in diameter.^427^

### 4.5 Summary

The individual and collective alignment of CNCs provides a way to control the orientation and periodicity of the cholesteric phase across samples from the micron to the centimeter scale. The examples provided above can be considered separately or combined together, and rely on anchoring and geometry, on flows of various types (including shear,^343,362,363,428,429^ elongation,^360^ vortices,^430^ etc.), gradients (concentration,^428^ temperature,^431^ surface tension,^430^ etc.), on the tactoids reorganization dynamics,^333,335,336^ and the interface displacements (pinning-depinning of the air–liquid interface,^336,375,429^ or on the propagation of evaporation or phase separation front,^432^ etc.

Regardless of the chosen pathway, translating the structure from the cholesteric phase into a solid film requires that the achieved order can be locked in place to prevent further rearrangement of individual CNCs. This transition between these two regimes can be conceptualized by the notion of kinetic arrest, discussed in the next section.

## 5 Kinetic Arrest (KA) in CNC Suspensions

The colloidal stability of CNCs is an essential prerequisite for the liquid crystalline ordering discussed in the previous section. An unstable CNC suspension would lead to early flocculation and would compromise its ability to form a cholesteric liquid crystalline phase. In the process of producing solid CNC films, however, there must inevitably be a transition from a liquid-like, flowing suspension of freely moving CNCs to a gel-like, nonflowing sample with CNCs displaying reduced degrees of freedom. This transition can be referred to as the onset of *kinetic arrest* (henceforth referred to as KA), also known as dynamic arrest.^433^ The onset of KA therefore plays a critical role in the evolution of the CNC self-assembly into solid films.

Crucially, a solid photonic film with a helicoidal microstructure can only be produced if KA occurs after the liquid crystal transition. If instead the sample undergoes KA before liquid crystal ordering has occurred, an isotropic structure (e.g., hydrogel) will be produced, and the film will display no structural color. While CNC preparation, formulation and casting conditions provide ways to control the system (see section 8), the liquid crystal transition and the onset of KA are not external parameters that can be independently tuned, but rather characteristics of the system that can only be influenced indirectly. It is therefore essential to understand the mechanisms of KA in CNC suspensions in order to identify ways to modify the onset of KA (i.e., delay or trigger it) and thus influence the final properties of the resulting films. While the importance of the KA transition has already been acknowledged in the colloidal liquid crystal community,^434,435^ its key role in the self-assembly of CNC photonic films was first highlighted by Lagerwall and co-workers,^311^ and has subsequently been investigated over the following years by other groups,^288,308,416,436,437^ as recently discussed in detail elsewhere.^18^ From a rheological point of view, the interplay between gelation and liquid crystalline ordering in CNC suspensions has also been explored independently,^285,291,367,264,343,347^ and adds to gain a better understanding of the role of KA upon solvent evaporation.

In this section, the conditions of colloidal stability of dilute suspensions will first be discussed, starting with the DLVO formalism applied to rod-like particles and leading naturally to the other scenarios in which colloidal stability is lost, and to a general discussion of the various types of KA that can occur. The experimental tools to identify the conditions and the type of KA will then be presented, and finally the observation of KA in CNC suspensions will be reviewed and discussed.

### 5.1 Colloidal Stability of Dilute CNC Suspensions

This section discusses the conditions of colloidal stability of CNCs in terms of the DLVO formalism (defined below), including the scenarios of poor colloidal stability leading to aggregation and flocculation.

#### 5.1.1 DLVO Theory Applied to CNCs

CNCs produced by acid hydrolysis form stable suspensions in water due to their negative surface charge, which leads to electrostatic repulsion between CNCs. This behavior is common to electrostatically stabilized colloids and can be understood qualitatively with the Derjaguin–Landau–Verwey–Overbeek (DLVO) theory, which was originally developed for spherical particles.^438−440^

A colloidal dispersion will be stable when the short-ranged van der Waals attractive interactions, scaling as a power law of the gap *h* between the particle surfaces, is overcome at larger distances by an electrostatic repulsion that scales with the Debye length κ^–1^ (as introduced below). The DLVO approach assumes the overall pair interactions between two particles is well approximated by the sum of these two contributions, neglecting other effects such as the size of the ions, hydration layers, entropy of the solvent, or additional surface effects. Moreover, while this approach is useful to assess the colloidal stability of given particles in dilute suspensions, extrapolating their collective behavior to dense assemblies usually reaches outside the domain of validity of the theoretical expressions. In particular, the pair potential given by the DLVO approach is no longer applicable to account for the interaction between multiple particles in high volume fraction range and when the Debye length is comparable to the average distance between particles.

The DLVO pair interaction potential for elongated rods with cylindrical symmetry has been applied to CNCs in several studies,^284,441,442^ and is provided in section S1 of the Supporting Information.^224,327,443−445^ While the van der Waals attractive potential is not easily influenced by formulation, the repulsive potential is easily modulated by the control of the particle charge and the suspension ionic strength, via the Debye length κ^–1^ given in its general expression as:
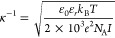
22Where ε_0_ is the permittivity of free space and *ε*_*r*_ is the dielectric constant of the solvent. In an aqueous medium and at room temperature, this expression can be approximated as
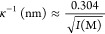
23

In the equations above, the ionic strength *I* is defined as
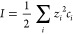
24with *c*_*i*_ the molar concentration (in M or mol/L) of each ionic species in the aqueous phase, and *z*_*i*_ is their valence. Equation 23 is valid for a solution of free electrolytes at infinite colloidal particle dilution, and does not include the counterions associated with the charged colloidal particles. Otherwise, the concentration of counterions can be taken into account as
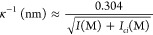
25where the ionic strength *I*_*ci*_ associated with the counterions is
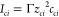
26with *c*_*ci*_ the molar concentration of each counterion, and *z*_*ci*_ their valence, and Γ the Donnan salt exclusion coefficient:^446^
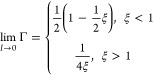
27

Here, we introduce ξ = *λ*_B_/*l*_eff_, which can also be written as ξ = *λ*_B_*ν*_eff_/*e*, with *λ*_B_ the Bjerrum length (0.714 nm in water at 25 °C), *l*_eff_ the effective distance between two elementary charges ± *e* along the particle long axis, and ν_eff_ the effective linear charge density ν_eff_ = ± *e*/*l*_eff_. The Donnan exclusion coefficient Γ is introduced to account for a phenomenon known as Manning condensation:^446^ if the linear charge density on the particle surface exceeds a certain threshold, counterions become condensed onto the particle surface, reducing the effective linear charge density value ν_eff_. This causes additional grafting of charged species to become ineffective and ν_eff_ then reaches a plateau, as thermal energy cannot prevent the additional counterions from condensing onto the particle.^446^ For ChNCs, the same consideration is valid, although in that case the Γ term should also be multiplied by the ratio of protonated groups to total groups present at the surface.^271,447^

**Figure 20:**
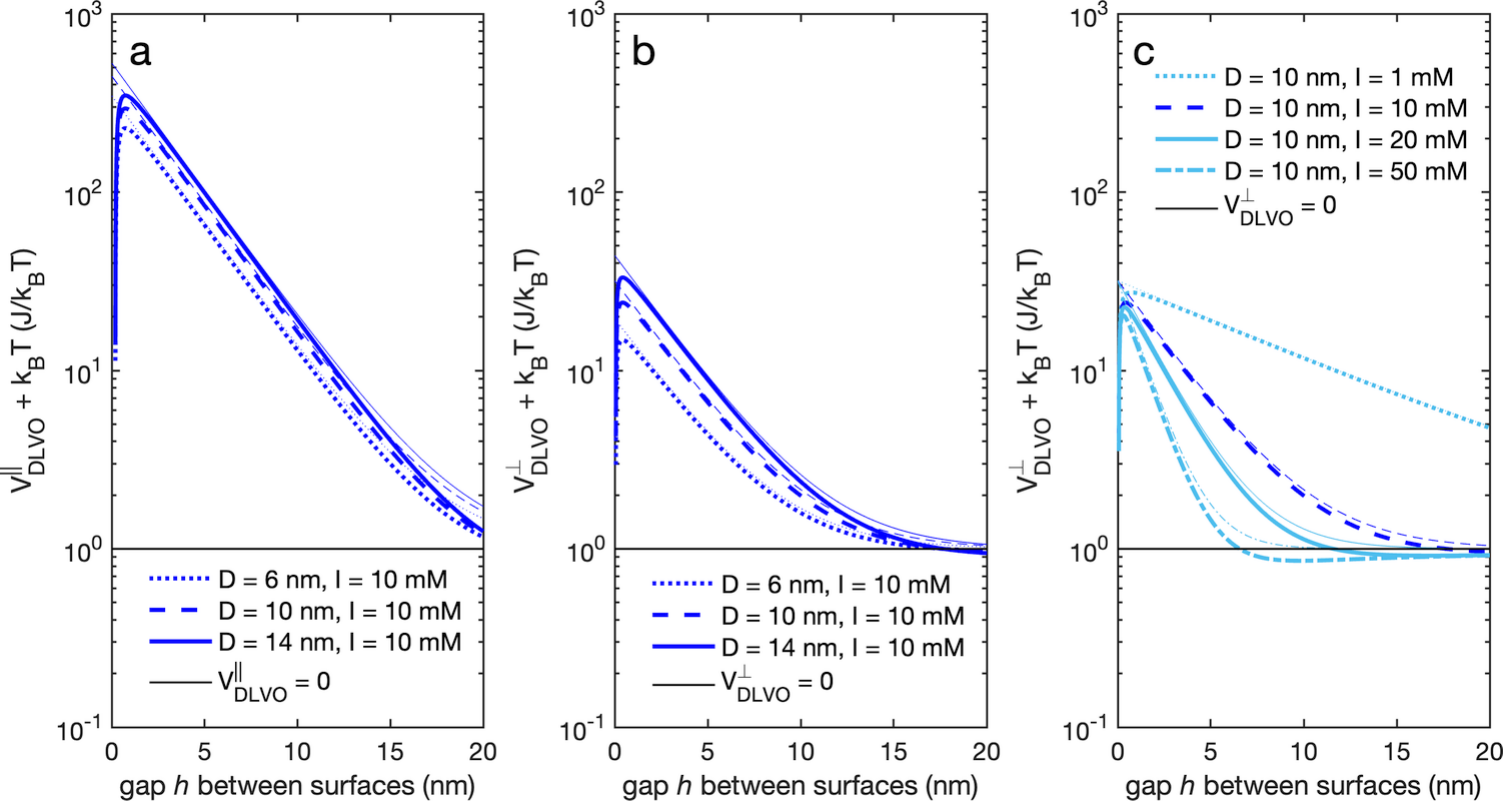
Pair interaction potentials between charged cylindrical rods (a) oriented parallel to one another and (b) oriented perpendicular to one another, for various diameters *D*, and (c) oriented perpendicular to one another for various ionic strengths. The potentials were evaluated from the provided expressions in the Supporting Information and plotted as *V* + *k*_B_*T* (in *k*_B_*T* units at room temperature) to allow using lin-log axes, assuming ψ_0_ = −60 mV and *L* = 200 nm.

Examples of applying DLVO models to CNCs to assess the effect of surface potential and ionic strength are shown in Figure 20, as also explored elsewhere.^442^

While the average interparticle distance is set by the CNC concentration, the electrostatic repulsion between CNC determines their typical range of interaction (and in particular, what proportion of their time they spend in close proximity). To a first approximation, electrostatic repulsion can be accounted for by modeling the CNCs as hard particles with an effective diameter *D*_eff_ that is greater than their bare diameter *D* = 2*R* (which can be arguably defined as  or ). Different methods have been proposed to estimate the effective diameter *D*_eff_, which rely on different theoretical grounds and thus have limited validity range. In the regime where the particles are stabilized by electrostatic repulsion, the effective diameter is expected to increase with the Debye length κ^–1^, which introduces a dimensionless parameter ϱ so that
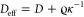
28

The expression for ϱ is difficult to estimate in practice. It can be derived from the electrostatic potential of the DLVO interaction, provided the expression for its potential is valid for the range of particle concentration, surface charge and ionic strength (which is unfortunately no longer the case at high Φ and low *I*). In the case of strong electrostatic repulsion and neglecting the vdW interactions, ϱ can be estimated as^271,447,448^
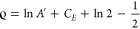
29where *C*_E_ ≈ 0.5772 is Euler’s constant and
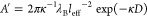
30

Different expressions can be used to derive *l*_eff_ (or equivalently, ν_eff_), such as for instance:^271,449^
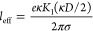
31where *K*_1_(*x*) denotes the first order modified Bessel function and σ is the surface charge density (in C/m^2^) of the particle surface. Alternatively, for *κD*/2 ≥ 1 it has been proposed that *l*_eff_ saturates as *l*_eff_^sat^ ≈ λ_*B*_/(*κD* + 3/2).^447,448^ Note that a different expression for *D*_eff_ is obtained when using the analytical formula proposed by Sparnaay for electrostatic repulsion.^444,450^

**Figure 21:**
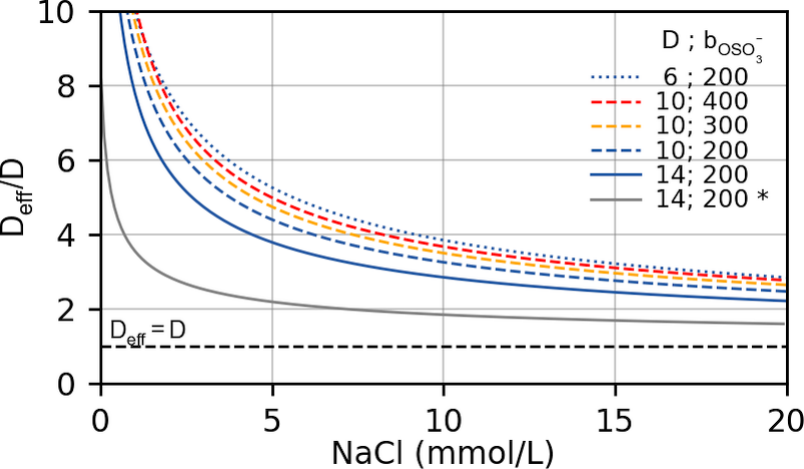
Comparison of D_eff_ evaluated from equation 28 using the analytical formula of electrostatic repulsion (neglecting vdW interactions, and assuming mass fraction of 5 wt % and a length *L* = 200 nm) and experimental estimations from rheological data interpreted with the modeling of its effect on the aspect ratio (in gray, marked with *, from ref (264). Legend shows curves in the same order of appearance from top to bottom, with the surface charge *b*_OSO_3_^–^_ expressed as molality (i.e., mmol of OSO_3_^–^ per kg of CNCs).

There are also experimental methods to estimate *D*_eff_ from rheological measurements at various salt and particle concentrations, based on the dependence of the viscosity with the effective aspect ratio of the particles.^264^ An example of such experimental determination for CNCs from wood pulp led to ϱ ≈ 3.9, in the high volume fraction regime (for Φ ≳ *a*^–1^, with *a* = *L*/*D*), including when the CNCs are experiencing kinetic arrest.^264^ The tumbling frequency upon steady shear might be another rheological method to estimate an effective aspect ratio *a*_eff_ = *L*_eff_/*D*_eff_ of the rods, and thus, if knowing *a*, a value for ϱ, yet applicable rather in the semidilute regime (for *a*^–2^ ≲ Φ ≲ *a*^–1^).^355−357^ The analytical and experimental values of *D*_eff_ are compared on Figure 21, and show that overall the analytical formula overestimates *D*_eff_. Values at higher salt concentration are not reliable as the vdW interactions need to be accounted for. As mentioned in the beginning of this section, the DLVO approach is most adapted to describe dilute suspensions. However, applying the resulting *D*_eff_ to describe the CNC collective packing at high volume fraction is justified more qualitatively than quantitatively.

#### 5.1.2 Conditions of Colloidal Stability

Using DLVO theory, the conditions of colloidal stability can be determined (i.e., where the particles repel each other sufficiently to remain individually dispersed). At low ionic strength (<10 mM for CNCs), the repulsive electrostatic potential dominates at long-range and the particles repel efficiently as they approach each other (Figure 20). Although the total potential has a global minimum at very short-range, corresponding to an aggregated state, the potential barrier that must be overcome to reach it is high (>10 *k*_B_*T*), so this state is not accessible within a reasonable time scale using simply thermal activated processes. The suspension is thus considered colloidally “stable”. Furthermore, in this case the second virial coefficient (as introduced earlier in section 3.2.1 and noted *B*_2_ or *B*′_2_, depending on its units) is positive. At high ionic strength (typically >60–100 mM of a 1:1 electrolyte), the electrostatic interaction decays at too short-range and the attractive van der Waals interactions dominate, leading to aggregation.

Aggregation requires that a given particle diffuse toward a growing cluster, and then remain in the vicinity of the cluster long enough to become bound (overcoming any potential barrier to binding). If the attraction is very strong, particle diffusion is the limiting factor, and large aggregates form quickly via diffusion limited cluster aggregation (DLCA). At low volume fraction, DLCA leads to flocculation, causing the sedimentation of the CNCs as a thin pellet. The aspect ratio of the rods is expected to increase the fractal dimension of the aggregates (e.g., from 1.81 ± 0.02 for spheres to 2.3 ± 0.2 for rods of aspect ratio 30).^451^ The valence of the added ions is found to have a drastic effect on the concentration of particles and electrolytes required to trigger flocculation, and is usually expected to follow the Schulze-Hardy rule (∼*z*_*i*_^–6^), a relation that can be deduced from the DLVO theory.^438,440,452,453^ If the attraction is more progressive (typically within 25–60 mM of a 1:1 electrolyte), the aggregation is slower and leads to aggregates that grow over time.^454,455^ In this situation, the Reaction Limited Cluster Aggregation (RLCA) model is usually most appropriate, where the kinetics of aggregation are determined by the height of the activation barrier *V*_b_ = max [*V*_DLVO_^⊥^(*r*_axes_)] required to overcome the long-range repulsion. The frequency of random encounters between two CNCs depends on their concentration and their Brownian motion in the surrounding medium, while the likelihood that each encounter results in a bond depends on *V*_b_. The Fuchs ratio, *w* (defined as the inverse of this likelihood) can thus be accessed experimentally and related to the barrier height as^373,456,457^
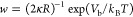
32

The fractal dimension of these aggregates, usually around 2.13 ± 0.07 for spherical particles, is barely increased to 2.2 ± 0.15 for rods of aspect ratios about 30.^458^ At the onset of aggregation, usually around 20 mM for typical CNCs, the second virial coefficient is found to be positive (e.g., *B*′_2_ = 0.7 × 10^–8^ L mol g^–2^).^454^

While aggregates in an infinitely diluted suspension are expected to sediment at long times, their formation in less dilute suspensions can also lead to gelation, which will be discussed below in the context of other types of kinetic arrest.

### 5.2 Types of Kinetic Arrest Transitions

The mechanisms of KA in colloidal systems are the subject of ongoing research. Much of our current understanding comes from studies on spherical colloidal model particles, where the particle interactions are tuned by varying the particle surface charge, the ionic strength, the volume fraction, the temperature, or adding depletants to introduce short-range attraction.^433,459^ The KA then correlates with specific particle positional arrangements, characteristic of the type of KA involved. However, anisometric particles, e.g., rods or platelets, can exhibit more complex behavior, where their mutual orientation also plays a major role. Moreover, how the microscopic phenomena (e.g., aggregation) relate to the macroscopic phenomena (e.g., rheological signature) is complex and still not well understood.

It is also important to mention that KA does not necessarily occur for all degrees of freedom of the system simultaneously: indeed, some degrees of freedom (e.g., positional or rotational), both of individual CNCs and their collective behavior (e.g., pitch or helical axis), can “freeze” or become “locked” (i.e., “arrested”) before others. The characterization of KA therefore depends on the degree of freedom being tracked, which in turn depends on the experimental technique chosen.

For all types of KA, the transition is sensitive to the relative time scales at play in the system, as there is a lag time between phenomena at the micro- and the macroscopic length scales. The speed of the quench into the arrested state, relative to thermodynamic equilibration, is particularly important. For CNCs, this quenching process is assumed to be relatively slow, as the sample is typically dried at a time scale of several hours to days.

One of the difficulties in describing and understanding KA is a lack of consistency in how the terminology is applied to different systems. The distinction between glasses and gels is not universally agreed upon. In a recent review of colloidal KA,^433^ it was proposed that a low-density state driven by attraction be called a gel, whereas a low-density state driven by repulsion be a glass. A high-density state driven by repulsion or attraction is also considered a glass.^460^ Moreover, within highly repulsive systems, a distinction can be drawn between a glass transition, usually occurred in thermally quenched systems and where thermal processes (such as Brownian motion) plays the main role, and the jamming transition, usually occurred in granular materials upon a volume reduction and is essentially athermal (but the particles may be brought in motion with respect to each other, as in shearing, compression, etc.). In soft systems, both effects can coexist and lead to different regimes.^461^ With the introduction of anisotropy and the long-range degrees of freedom that emerge from it (such as the helical axis and the pitch in a cholesteric suspension), the notion of KA becomes even more complex to address and remains vastly unexplored. In the following, a brief description of simple categories of KA mechanisms will be given.

#### 5.2.1 Repulsive Glasses from Hard Particle Repulsion

Starting from the simplest case of particles with only hard-particle repulsion, it is expected that particle motion will be arrested when their packing density exceeds a certain threshold. In the case of monodisperse particles, packing is optimized by forming an ordered state, such as the crystalline lattice formed by densely packed spheres. However, such ordered structures cannot occur if the polydispersity in particle diameter is too great (σ̂ > 0.1). A polydisperse sample will instead become arrested at a threshold packing density typically above 50 vol %. The resulting structure is a high-density colloidal glass in which the particles are in close contact. In the specialized literature on this topic, a distinction is often made between a true glass transition, in which the Brownian motion enables thermally activated relaxation events, and a jamming transition, in which the thermal contribution is negligible and the transition is mainly driven by its compactivity,^462−464^ such as in granular systems.^461^

#### 5.2.2 Repulsive Glasses from Long-Range Repulsion

Particles with long-range repulsive interactions are expected to undergo a glass transition like simple hard particles. However, KA of repulsive particles is expected at a much lower threshold packing density than hard particles due to their greater effective size.

As first predicted by Wigner for electrons, repulsive particles can form a stable low-density periodic configuration, referred to as an “electron crystal”.^465^ An analogous crystalline state has been achieved for highly charged colloidal particles with long-ranged electrostatic repulsion.^466^ Alongside the crystalline state, factors such as polydispersity can lead to a glass-like, amorphous arrested state at low packing fraction, which has been since then dubbed a “Wigner glass” or “ionic glass” state.^467^ The strong electrostatic repulsion can be viewed as extending the excluded volume of each particle beyond its bare volume, to account for the volume of a soft repulsive shell, whose thickness corresponds to the range of the repulsion. Equivalently, an effective volume fraction accounting for this shell can be considered as more relevant than the bare volume fraction for triggering the glass transition beyond a certain threshold.^468,469^

As a system of repulsive particles approaches KA, the motion of each particle becomes increasingly constrained by the presence of its immediate neighbors. Consequently, the central particle can only be displaced by the coordinated displacement of the surrounding particle “cage”. If the particle can eventually escape the surrounding “cage”, the system is eventually able to relax. However, with increasing particle concentration the caging time diverges, leading to KA of the suspension. In practice, this divergence is experimentally defined as longer than the time scale of the observation. More information on this phenomenon can be found in dedicate reviews, e.g. ref.^470^

In colloidal systems, the long-range repulsion required to produce a Wigner glass is provided by electrostatic interactions, with a Yukawa-type potential.^433^ This Yukawa-type potential occurs for charged spherical particles in the Hückel regime (i.e., at very low ionic strength, so that *κR* ≪ 10, where *R* is the particle radius), and can thus be expected for low-density suspensions of charged CNCs in absence of additional free electrolytes (i.e., no free salt or acid). Interestingly, particles with short-range attraction and long-range repulsion of competing magnitude can, after undergoing a first stage of partial aggregation, form small clusters that build up an electrostatic charge and thus a dominating long-range repulsion. In this case, these clusters of particles act as “meta-particles” that evolve toward a repulsive glass, termed a “Wigner cluster glass”.^471,472^

#### 5.2.3 Attractive Gels and Attractive Glasses

As discussed in section 5.1, particle aggregation occurs when the attraction between colloidal particles is dominant (e.g., at excessively high ionic strength). The size and density of the resulting clusters depends on the strength of the attractive interaction, while the cluster structure has a fractal dimension that depends on the aggregation process and particle aspect ratio (see section 5.1.2). Interestingly, all colloidal gelation mechanisms based on attractive interactions can be seen as analogous to equilibrium liquid–gas phase separation, rather than being triggered by a kinetic phenomenon,^473^ but with characteristics of a second-order phase transition.^474^

If the particles are denser than the surrounding medium (as is the case for CNCs in water), aggregates are expected to sediment over time. However, the mass fraction of aggregates decreases with their diameter due to their fractal structure. Consequently, the aggregate mass fraction will eventually approach the average sample mass fraction (which occurs more easily in concentrated suspensions), and in this case the aggregates can percolate into a network throughout the sample before sedimentation occurs, leading to a bulk hydrogel. If the cohesion forces between the aggregates are comparable to the attractive bonds causing the aggregates to form, the gel can be termed *an attractive gel*. The suspension is then kinetically arrested at large scale, although it can retain some mobility at smaller scale. Over time, samples with stronger attractions that already formed a volume spanning network may further contract and expel some water, a phenomenon called *syneresis*.^475^ This effect, which can be observed in sufficiently dense CNC suspensions,^454^ differs from the sedimentation occurring at lower volume fraction, whereby many aggregates rapidly flocculate into a pellet without first forming a volume-spanning gel.^454^

KA into an attractive gel can be triggered by increasing the ionic strength and/or the CNC volume fraction, typically upon solvent evaporation, and can be relevant in triggering the KA in CNC suspensions after forming a cholesteric phase. For even higher volume fraction, the suspension experiences both aggregation (due to attractive interactions) and crowding effects (from hard particle repulsion), leading to more exotic situations that are more difficult to study and classify, and have been less studied in the literature. As such, some arrested phases were considered as being repulsive glasses or attractive glasses.^460^ In this review, we adopt a less restrictive definition for “attractive gels” that can includes such “attractive glasses”, but we reproduce the term “attractive glass” whenever the term was preferred in the cited literature. Finally, while the binary classification of the interactions between particles as being either attractive or repulsive is a useful simplification, more complex pair interaction potentials are possible (by varying e.g. depth of the attractive potential well, height of the repulsive interaction or range of the repulsion). This is especially true when allowing for non-spherical shape (discussed below) or the presence of attractive or repulsive patches on the particles. Consequently, a much richer variety of exotic arrested states is possible in general.

#### 5.2.4 Effect of the Particle Morphology

Anisotropic particles (including prolate elongated particles, such as CNCs, and oblate disk-like particles, such as clay platelets) exhibit much more complex KA behavior as the interactions between particles depends on their mutual orientations as well as their mutual positions.^451^ Consequently, KA of anisotropic particles includes the phenomena mentioned above, alongside alignment phenomena described by the Onsager model. The simultaneous occurrence of KA, nematic phase separation and local rod alignment can therefore result in hybrid, partially aligned phases such as “nematic gels”.

In general, theoretical analysis of the KA transition for anisotropic particles is less tractable, as the criteria for KA depend on the local degree of orientation (usually captured by the local order parameter *S*_2_ introduced in section 3.1). Many models for KA of hard rods circumvent this issue by assuming that the rod orientations are isotropic and uncorrelated (i.e., *S*_2_ = 0). Exact expressions for the KA threshold can then be obtained for isotropic suspensions of monodisperse hard particles, but the polydispersity in rod length and width, which are often observed experimentally, will strongly affect the KA transition.^476^ In the other limit, the percolation threshold for highly aligned nematic (i.e., *S*_2_ ≈ 1) may be determined by particle connectivity (i.e., range of interaction) rather than particle density.^477^

This competition between liquid crystalline phase transitions and KA has been explored in experimental studies on systems of anisotropic particles such as laponite clay,^478−481^ other clay platelets,^434,435^ and other anisometric particles.^482^ However, the acquisition of experimental observations that can address these questions is limited, and lead sometimes to unexpected behavior, which may be general or specific to the chosen model system.^482^

As a final comment, it might be useful to remind that while gels and glasses are often considered isotropic disordered states, anisotropic particles in an arrested state usually interact anisotropically and thus do not locally arrange with a local isotropic symmetry, but are more often stuck in an aligned out-of-equilibrium state. Alignment and KA are thus not exclusive of one another. This local alignment can also be inherited from their processing history, and then typically develop as a long-range orientational order, typically after experiencing some shear.^264^ The sequential order in which KA and the liquid crystalline transition occur will thus greatly impact the final spatial arrangement of the particles.

### 5.3 Common Detection and Discrimination Methods of Kinetic Arrest Pathways

Numerous experimental methods can be used to identify the onset of kinetic arrest as the CNC concentration and particle interaction are varied. Many of these methods can also distinguish between different types of arrested state and are listed below. Experimental observations of KA in CNC suspensions will be covered in section 5.4.

#### 5.3.1 Visual Inspection

One of the simplest methods to assess the arrested state of a sample is the so-called “vial inversion test”, where the sample is turned upside-down to determine if it remains stuck to the bottom of the vial.^284,483^ This very simple and commonly used method is suitable for screening obvious signs of arrested states, but also suffers from some important drawbacks. First, its implementation is often poorly defined (e.g., volume of suspension, diameter of the vial), which limits its reproducibility and validity. Indeed, the vial radius must not be smaller than the capillary length (about 2.7 mm for water), nor the vial insufficiently filled, as even nonarrested sols would then remain trapped in the vial upon inversion. Arrested suspensions could, on the other hand, flow out of the vial if the cohesion forces are too weak,^454^ or if not handled gently enough. However, the arrested state often causes an abrupt change of rheological properties (with storage moduli increasing across orders of magnitude) and this method, though imperfect, remains a useful tool in many situations.^284,483^ Furthermore, if visual signs of syneresis are present at longer time scales, it demonstrates that the sample is an aging attractive gel (see section 5.2.3).

For cholesteric suspensions in capillaries, visual inspection of the sample by optical microscopy can reveal textures that do not relax over time, indicating KA.^484^ This effect is most obvious for initially biphasic suspensions, where it is possible to observe that tactoids do not sediment and remained trapped in the isotropic phase even after several weeks. Shear alignment of an arresting suspension as the capillary is filled can lead to Schlieren-like textures similar to nematic phases, as well as nonrelaxing shear banding.^268^ Finally, features that could be initially caused by spinodal decomposition (SD, see section 3.4.3), which are not a feature of KA, can become frozen in. Examples of such stripes, which were ascribed to arrested SD, have been reported in suspensions from tunicate CNCs.^268^

#### 5.3.2 Rheology

**Figure 22:**
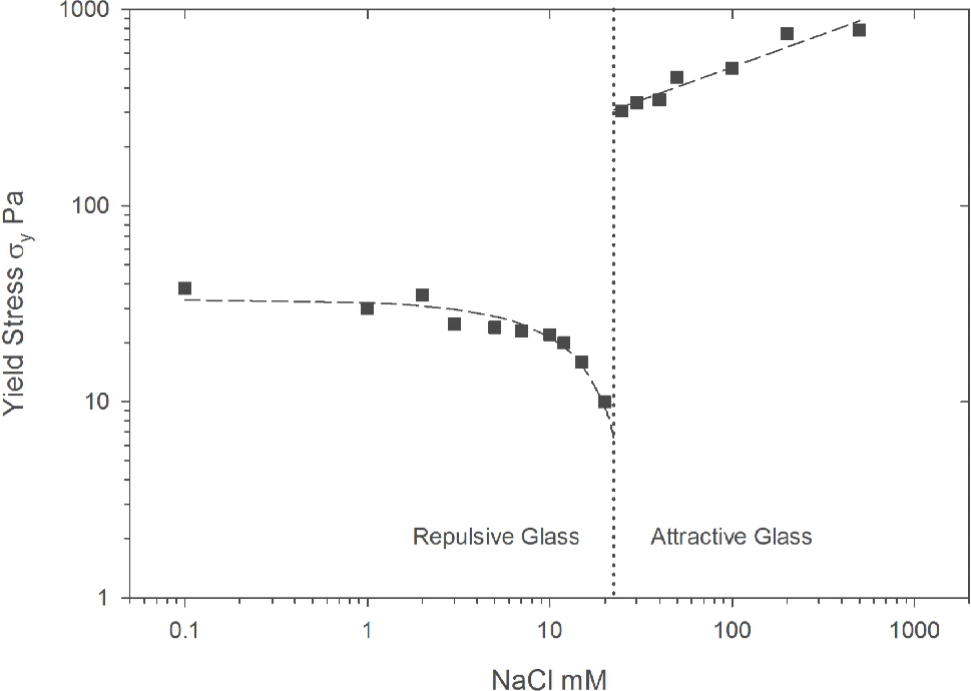
Observation of a transition from a repulsive colloidal glass to an attractive colloidal glass in CNC suspensions, from the discontinuity of the yield stress σ_Y_ (determined from a shear stress sweep test where the viscosity drops) as a function of NaCl concentration, shown on log–log scale (11.9 wt % CNC). The dashed line is a guide to the eye. Note that the value of σ_Y_ for zero added salt was plotted at 0.1 mM to fit it on the log scale. Adapted with permission from ref (373). Copyright 2018 The Royal Society of Chemistry.

The KA transition, whether by gelation or glass transition, is associated with dramatic changes in the viscoelastic properties of the suspension that can be probed using oscillatory rheology. Above the KA threshold, the storage modulus *G*′ exceeds the loss modulus *G*′′, indicating a transition from predominantly viscous to predominantly elastic response to shear. As *G*′ and *G*′′ are frequency-dependent properties in the viscoelastic liquid state,^346^ an additional condition often used to identify KA is that the crossing point must be independent of frequency.^485^ Arrested phases driven by attractive and repulsive interactions can be distinguished by their yield stress (Figure 22),^478^ or creep compliance.^373^ The aging of glasses and gels can also affect their rheological response. It is important to bear in mind that rheology measurements can significantly perturb the microstructure of the system and therefore affect the KA transition. Consequently, near zero-shear rheological methods, or passive microrheological tools, may be more appropriate to capture KA as it occurs *in situ* under the low strain rate conditions of a slowly drying suspension.

A range of rheological measurements can be performed to identify the different regimes of interaction of the suspension. It can be used to identify the liquid-to-solid transition and its nature, and to discriminate the different types of arrested states, in particular by monitoring how some adjustable parameters during the sample preparation, such as the ionic strength, affect the rheological properties.^486^ A first example is the use of creep recovery tests, where the sample was left to relax following a stress-relaxation sequence, allowing for the estimation of the recoverable part of the compliance, which drops in samples that are still liquid-like.^373^ A second example is the observation of a clear transition in the yield stress as the sample transits, at high volume fraction, from an arrested repulsive glass to an arrested attractive gel (termed “attractive glass” by the authors, Figure 22).^373^ It can also be used to extract the dependence between the ionic strength and the effective diameter *D*_eff_. The interested reader will find more on this topic in this review,^264^ while rheology coupled with other techniques to probe the structure are reviewed elsewhere.^347^

#### 5.3.3 Static Light Scattering and Small Angle Scattering

The scattering (of visible light, X-rays or neutrons) from an arrested sample depends on the system and its characteristic length scales. For instance, the fractal-like microstructure of attractive gels introduces an additional length scale, the mesh size of the gel ξ, above which the average volume fraction in the sample is relatively uniform. Depending on the length scale to be probed, scattering measurements may be performed by visible static light scattering (SLS), or by small angle scattering with X-rays (SAXS) or neutrons (SANS). These techniques give access to the structure factor *S*(*Q*), which is informative of the mutual arrangement of the particles in the sample.

In a colloidal gel, where KA is driven by attractive interactions, one finds that *S*(*Q*) > 1 in the Guinier regime (i.e., for scattering vectors *Q* < 1/*R*_g_, where *R*_g_ is the gyration radius of the aggregating particles).^453^ For particles at the size of typical CNCs, the low *Q* regime in the presence of aggregation is mostly accessible by SLS, where *S*(*Q* → 0) = *N*_agg_ ≫ 1 corresponds to an increase of light scattering (where *N*_agg_ corresponds to the average number of aggregated particles per cluster). Aggregation also causes the suspension to appear more turbid, which can also be monitored by measuring direct light transmission of the unscattered beam (*Q* = 0).^454^

In a sample where repulsive interactions dominate (such as in a repulsive glass, but also in a nonarrested dense suspension), the scattering is maximal at a scattering vector that usually scales as the inverse of the interparticle distance *d* (*Q* = 2π/*d*, with *d* ∝ Φ^–ν^*R* with ν = 1/3 for spheres), termed a “correlation peak”, while for lower scattering vectors *Q* < 1/*R*_g_, one finds that 0 < *S*(*Q*) < 1, indicative of a rather homogeneous and uniform distribution of the particles at larger scales. In stable isotropic suspensions, a power law of ν ≈ 0.4 has been reported, ascribed to packing of CNCs mainly through space filling rather than tightly bound clustering.^487^ In stable cholesteric CNC suspensions, a power law of ν = 0.45 has been observed,^292^ closer to ν = 1/2 (expected for infinitely long rods), where *R* then scales as the average radius of a cylindrical model particle. In this regime, the asymptotic value of *S*(*Q* → 0) is proportional to the thermodynamic isothermal compressibility of the glass (i.e., the differential change in volume due to a change in osmotic pressure). A more uniform distribution of CNCs across the sample is thus expected to result in a less compressible gel with lower turbidity.

An alternative, indirect way to investigate the nature of the interactions between particles in an arrested phase is to investigate their behavior in the dilute regime (provided the ionic strength is kept identical), to isolate the effect of attractive interactions. Common analysis of their colloidal interactions in the dilute state involves the construction of a Zimm plot using static light scattering (SLS) at different CNC volume fractions, from which the particle bare volume *v*_CNC_, their gyration radius *R*_g_ and the second virial coefficient *B*_2_ can be estimated.^488^ However, the interpretation of the regime at low scattering vector *Q* (small angle) for polydisperse rod-like particles is usually not very robust because of missing data at lower *Q* values and uncertainty due to the presence of larger impurities that increase forward scattering.^489^

#### 5.3.4 Particle Diffusion via Light Scattering Methods

A variety of microrheological techniques, based on particle diffusion, can be used to track the onset of KA.^490,491^ While dynamic light scattering (DLS) is commonly used for measuring the average hydrodynamic diameter and can be employed to follow aggregation kinetics, it implicitly relies on the validity of the Stokes–Einstein relationship for the translational degrees of freedom of individual probing particles, relating their passive behavior (i.e., Brownian motion) to the fluid resistance to their active manipulation (i.e., the viscosity of the fluid). In a system undergoing kinetic arrest, the active and passive behaviors of the system are no longer in correspondence as the sample is no longer ergodic, meaning that time-averaged quantities are no longer equivalent to ensemble-averaged quantities. Several methods have been used to measure these complementary quantities as a way to track the loss of ergodicity at the onset of kinetic arrest. The direct observation of individual tracer particles, e.g., by differential dynamic microscopy,^482^ can be used to monitor a change in the diffusion behavior of the tracer particles over time, from diffusive to subdiffusive. The comparison of microrheological methods in passive mode (e.g., estimating the viscosity from Brownian motion) and in active mode (e.g., using optical tweezers, magnetic fields, etc., to directly measure the viscosity) allows for directly testing the Stokes–Einstein relationship.^492−494^ Alternatively, a regular dynamic light scattering (DLS) setup can be used to track the loss of ergodicity, by measuring the drop of the intensity of the autocorrelation function.^483,495,496^ The autocorrelation function, usually produced by the DLS autocorrelator software as an intermediate result, typically decays exponentially with correlation time, with a maximum value at time *t* → 0 that is usually slightly below unity in stable and dilute suspensions (ca. 0.9). This value suddenly drops (ca. < 0.7) when the sample begins to show signs of nonergodicity.^483^ A more complete set of information can be extracted from these samples if an ensemble-average autocorrelation function is also collected, usually implemented experimentally by rotating the (cylindrical) cuvette.^496^ Exploiting further this idea, diffusing wave spectroscopy (DWS) is a powerful technique for probing into dense and highly scattering samples to access the response in a much higher frequency range, usually by tracking tracing particles and extracting the storage and loss moduli of the matrix from their diffusive motion,^344,497,498^ with similar refinements such as sample rotation to acquire both time-averaged and ensemble-averaged information.^499^

#### 5.3.5 Post-Dilution Particle Sizing

Another way to distinguish between repulsive glass and attractive gel states is to dilute the arrested sample, as the liquid-glass transition is generally reversible, whereas gelation due to aggregation is generally irreversible.^433^ While a diluted repulsive glass will become a liquid, the dilution of a weakly cohesive attractive gel may also produce an apparent liquid sol, making it difficult to distinguish between these states in a first approximation. However, the nature of the particle interactions can then be identified by checking for a change of the intensity of the light scattering (using static light scattering, SLS) or by their associated hydrodynamic diameter (by dynamic light scattering, DLS).^500^ Finally, monitoring the flocculation or sedimentation after dilution is another way to qualitatively identify attractive gels.^455^

### 5.4 Experimental Observations of KA for CNCs

In recent years, several studies employing various measurement techniques have converged on the conclusion that CNC suspensions can exhibit both glass-like and gel-like KA states.^103,284,308,373,501^ Furthermore, the interplay of kinetic arrest and liquid crystal phase formation results in a complex phase diagram for CNC suspensions, as discussed in a recent review focusing primarily on rheological findings.^264^

To produce photonic films from self-assembled CNCs, it is essential that the liquid crystalline phase formation occurs before the microstructure is frozen by the onset of KA. The self-assembly pathway will determine whether the cholesteric phase will have time to rearrange, both on the microscale, where processes are faster, and on the macro-scale, where the processes are slower. Eventually, the suspension will become unable to relax, although different degrees of freedom may freeze at different points in time.

In the following section, the experimental observations of KA for CNCs will be discussed, first in the isotropic case and then in the anisotropic case. The isotropic case is relevant to understand how to avoid KA before the liquid crystalline order develops, but also because it offers a simpler (but possibly too simplistic) framework to understand how CNCs interact collectively. The anisotropic case is however more relevant to understand how aligned CNCs suspensions deviate from the isotropic case once the assembly occurred, since they locally interact differently due to the directionality of their colloidal interactions, but also via the coupling of short and long-range degrees of freedom.

#### 5.4.1 KA in Isotropic CNC Suspensions

If the onset of KA occurs before the conditions for liquid crystal phase formation are satisfied, a CNC suspension will form an isotropic arrested structure. For the case of attractive gels, the KA occurs due to insufficient colloidal stability, via a building up of the ionic strength as the suspension dries. Alternatively, at very low ionic strength a repulsive glass state may be created (as discussed in section 5.2).

One of the first thorough investigations of the KA transition in dilute CNC suspensions was conducted by Capron and co-workers, by combining SANS and light scattering.^454,455^ In a first study using sulfated CNCs from cotton in acid form,^455^ SANS over an extensive scattering vector range (*Q* = [0.001 Å^–1^ – 0.4 Å^–1^]) was used to show that while the CNCs were stable at [NaCl] = 2 mM, an early sign of attractive interactions was observed already at 10 mM (an extrapolation of *S*(*Q* → 0) = *N*_agg_ ≈ 1.7 could be interpreted as some weak and/or transient associations of CNCs in pairs, corresponding to a slightly negative *B*_2_). The authors reported overall no clear sign of aggregation below 20 mM. A clear sign of aggregation was observed at 50 mM (with *N*_agg_ ≈ 7.5). At even higher ionic strength (200 mM), the scaling of the structure factor at low *Q* indicated *N*_agg_ > 50 and followed the scaling *S*(*Q*) ∝ *Q*^–δ^, consistent with a self-similar structure of fractal dimension δ = 2.1. This fractal dimension is slightly lower than the one expected for pure RLCA, even when accounting to the high 3D aspect ratio *a*_3D_ of the CNCs, evaluated to  17.

**Figure 23:**
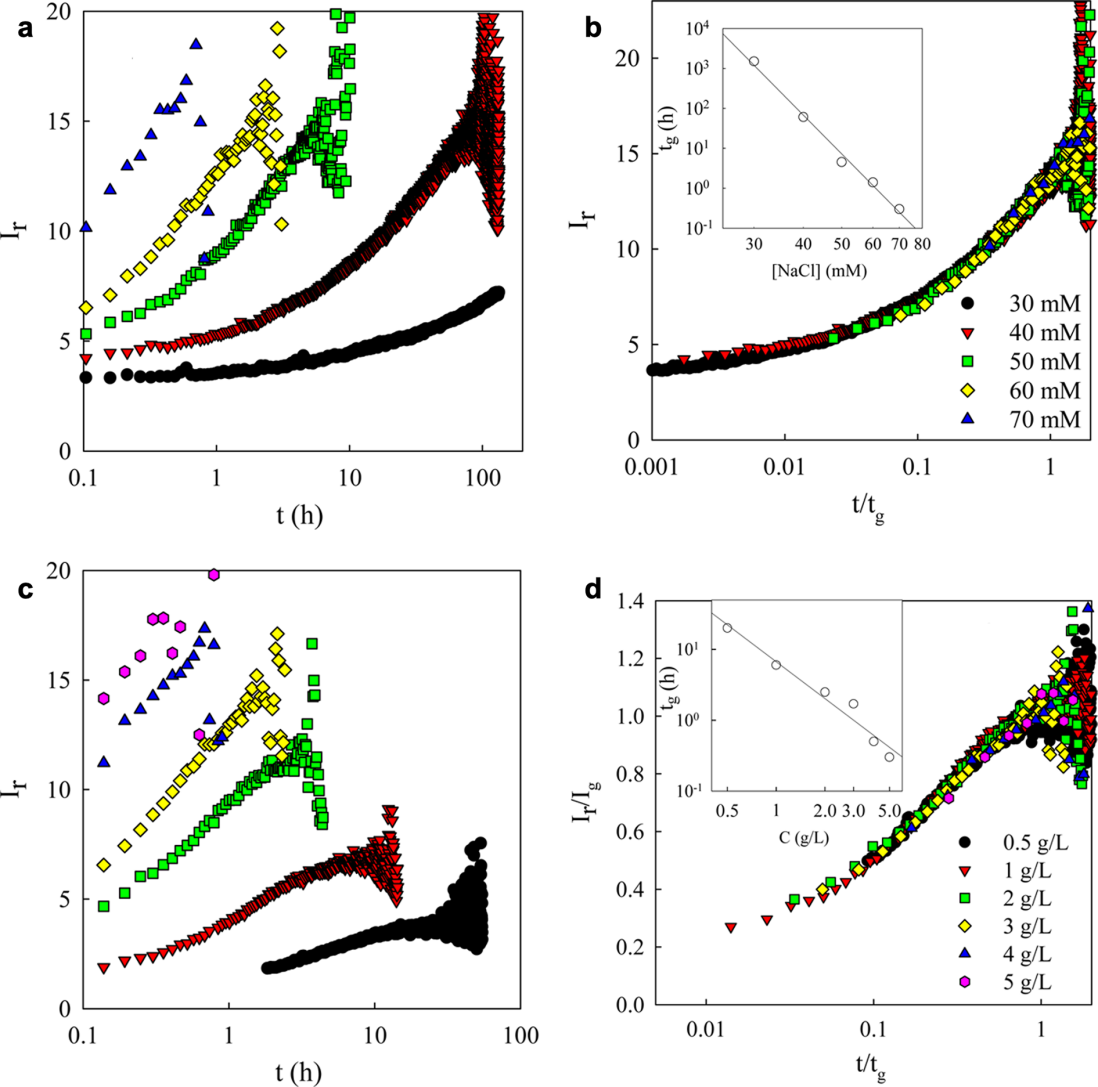
Evolution of the scattered light intensity of CNC suspensions as a function of time after preparation. (a) Intensity profiles at a CNC mass fraction of *c* = 0.5 g L^–1^ and various [NaCl]. (b) Same data in terms of dimensionless time *t*/*t*_g_, with inset showing the dependence of *t*_g_ with [NaCl] on a log–log scale. (c) Intensity profiles at [NaCl] = 70 mM and various values of c. (d) Same data in terms of dimensionless time *t*/*t*_g_ and normalized intensity *I*/*I*_g_, with inset showing the dependence of *t*_g_ with *c* on a log–log scale. Reproduced with permission from ref (454). Copyright 2016 American Chemical Society.

In a subsequent study,^454^ the aggregation and gelation process was monitored over time with SLS, varying the scattering angle, the sample concentration, the temperature and the ionic strength. While no aggregation was observed for [NaCl] ≤ 20 mM, they observed at higher salt concentration that the scattered intensity increases over time until reaching a gelation time *t*_g_. The gelation time at *T* = 20 °C followed a power law dependence both with [NaCl] and the CNC mass fraction *c*:
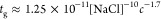
33(with *t*_*g*_ here expressed in hours, [NaCl] in M (i.e., mol L^–1^) and *c* in g L^–1^), which allows for the reduction of the scattering profiles to a single master curve (Figure 23). From their reported data, one can propose a stretched logistic curve to fit to their master curve:
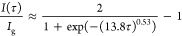
34where τ = *t*/*t*_g_ is the elapsed time since the quench (i.e., salt addition). The scattered intensity in this expression is normalized by the intensity scattered at the gelation point, *I*_g_, which increases initially linearly with *c* and then saturates at high concentrations (using the units of the original study, the intensity at gelation is *I*_g_ = 20.2(1–exp (− 0.45 *c*))). In practice, for a suspension at *c* = 0.5 g L^–1^, *t*_g_ varied between 2 months at [NaCl] = 30 mM and less than 30 min at [NaCl] = 70 mM. The dependence with temperature was also reported (*T* varied 10–60 °C for [NaCl] = 43 and 53 mM) and showed a decrease in the gelation time with the increase in temperature, with a peculiar plateau at low temperature (∼15 °C) that is not expected from a simple Arrhenius or Eyring-Polanyi law. Finally, in a highly aggregated gel at 70 mM, the reported power law dependence of the scattered intensity at low *q*, which is analogous to a *S*(*q*) ∝ *q*^–δ^ discussed in their previous work, is consistent with a self-similar structure of fractal dimension δ = 1.6, contrasting with δ = 2.1 observed at 200 mM. This indicates a significant increase in the density of the aggregates with the ionic strength, and is also consistent with the power law δ = 2.3 observed when all the surface charges of the CNCs are removed.^455^

The kinetics of recovery and aging dynamics of attractive CNC gels was further investigated at various salt concentrations using already arrested gels and monitoring their rheological response after cessation of a strong shear that rejuvenates the microstructure.^502^ In that study, the authors observed that the recovery of the storage modulus *G*′ obeys a time–composition superposition principle, and can be rescaled onto a universal sigmoidal master curve spanning beyond 10 orders of magnitude in time for a wide range of salt concentrations, indicative of universality. The inflection point of the sigmoid separates two regimes of distinct stretched exponential variations. Interestingly, the time at this inflection point, t*, is more appropriate than the typical crossover time *t*_c_ time (measured at 1 Hz; *t*_c_ is defined when *G*′ becomes larger than the loss modulus *G*′′) to estimate the *true* gelation time *t*_g_, which varies as a steep power law of the ionic strength:
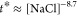
35

It is notable that this power law is very close to the previous exponent (*t*_*g*_∼[NaCl]^−10^) from the aggregation kinetics mentioned above,^454^ which reinforces the relevance of these kinetics of recovery and aging dynamics to understand the evolution of unstable CNC suspensions into attractive gels.

The effect of the aspect ratio on KA was investigated using CNCs and CNFs (which have much higher aspect ratio than CNCs) in order to access aspect ratios spanning across 2 orders of magnitude,^483^ and further discussed in a recent review on the colloidal properties of CNCs.^503^ In that study, the intensity autocorrelation function was used to define a criterion for KA, which led to the conclusion that suspensions of elongated cellulosic particles should become arrested above a volume fraction Φ = 1.54 *a*^–1^, where *a* = *L*/*D* is their aspect ratio. The authors interpreted this scaling with the aspect ratio as consistent with the transition from the semidilute regime (*a*^–2^ ≲ Φ ≲ *a*^–1^, where the rods are mainly prevented from rotating in three dimensions and are still allowed to translate or rotate parallel to each other) to the concentrated regime (Φ ≳ *a*^–1^, where the particle mobility is severely constrained by multiple contact points with neighboring particles and only rotation nearly along the long axis and vibration can occur). This definition, if prefactors are neglected, corresponds to an arrested glass, and their argument is convincing at least for CNFs, which exhibit the highest aspect ratios. This behavior is also consistent with the boundaries predicted for isotropic colloidal glasses, namely a Φ_KA_ between Φ = 5.4 *a*^–1^ and Φ = 0.7 *a*^–1^.^451^ While the particle aspect ratio is a crucial criterion for KA, this analysis seems to neglect the role of attractive interactions. In particular, it was assumed that only repulsive glasses would redisperse if diluted. However, a loose network in an attractive gel can also be disrupted upon dilution.^18^ Moreover, the role of attractive interactions, as illustrated in the studies mentioned above, makes this criterion alone less obvious, and the authors also acknowledged that aggregation has been observed in the chosen ionic strength regime. Finally, while the comparison of many references offers the potential of a more robust analysis of general trends, it is hindered by conflicting methods of measuring and defining experimental parameters between studies, such as the confusion between bare or effective dimensions, differing methods of estimating the particle aspect ratio, and the chosen definition of KA to locate Φ_KA_ with its associated error bars. All this suggests that the behavior of CNC suspensions by this simple inverse scaling should be considered only approximative at best.

An alternative and more complex analytical expression for the boundary conditions of the percolation volume fraction threshold has also been proposed, which also involves as an additional parameter the minimum approach distance between particles required to consider the particles as connected, and was in reasonable agreement with Monte Carlo simulations (suggesting a moderate under-estimation of the analytical formula for Φ_KA_).^504^ While this additional parameter suggests a less universal description of KA in elongated rods, it also offers the possibility to account for the effects of different short-range attraction potentials that are otherwise absent in the previous description of the KA of hard-rods.

#### 5.4.2 KA in Anisotropic CNC Suspensions

While the previous subsection was focused on KA in a collection of randomly oriented elongated particles, this assumption is no longer valid to account for the KA of liquid crystalline phases, or when the arrested state leads to local alignment.^18^ In practice, even if KA occurs very rapidly in a suspension, it is possible that some local alignment of the particles occurs before the structure becomes arrested, simply due to their locally anisotropic mutual interactions (see section 5.2.4). Indeed, in several of the studies on KA mentioned above as examples of “isotropic KA”, fingerprint patterns characteristic of cholesteric assembly were observed, indicating that the assumption of locally randomly oriented CNCs at the onset of KA was not satisfied. The analysis of KA for partially aligned rods is considerably more difficult since the degree of local alignment at KA is not a directly adjustable parameter, and the influence of alignment on KA has been explored for rod-like particles,^477,505^ including studies dedicated to CNCs.^18,308^

One of the first studies of KA in anisotropic CNC suspensions identified the transition from liquid to arrested state by vial inversion tests, which was confirmed by oscillatory rheology.^308^ A subsequent study explored the phase behavior and KA of CNC suspensions that were size-fractionated by phase separation (a method discussed further in section 8.2.3).^284^ It was found that the onset of liquid crystal behavior occurred earlier for longer (higher-aspect-ratio) CNCs, consistent with Onsager theory, while the onset of KA, as observed by vial inversion and oscillatory rheology, was found to be independent of CNC length. Instead, KA was shown to occur above a threshold value for the total ionic strength of the suspension (accounting for both the CNC counterions and added electrolytes), suggesting colloidal attractive gelation as the driving mechanism. Consequently, a drying suspension of shorter CNCs would be expected to have a shorter “self-assembly time window” (see section 9.1.3), compared to a suspension of longer CNCs cast at the same initial volume fraction.

**Figure 24:**
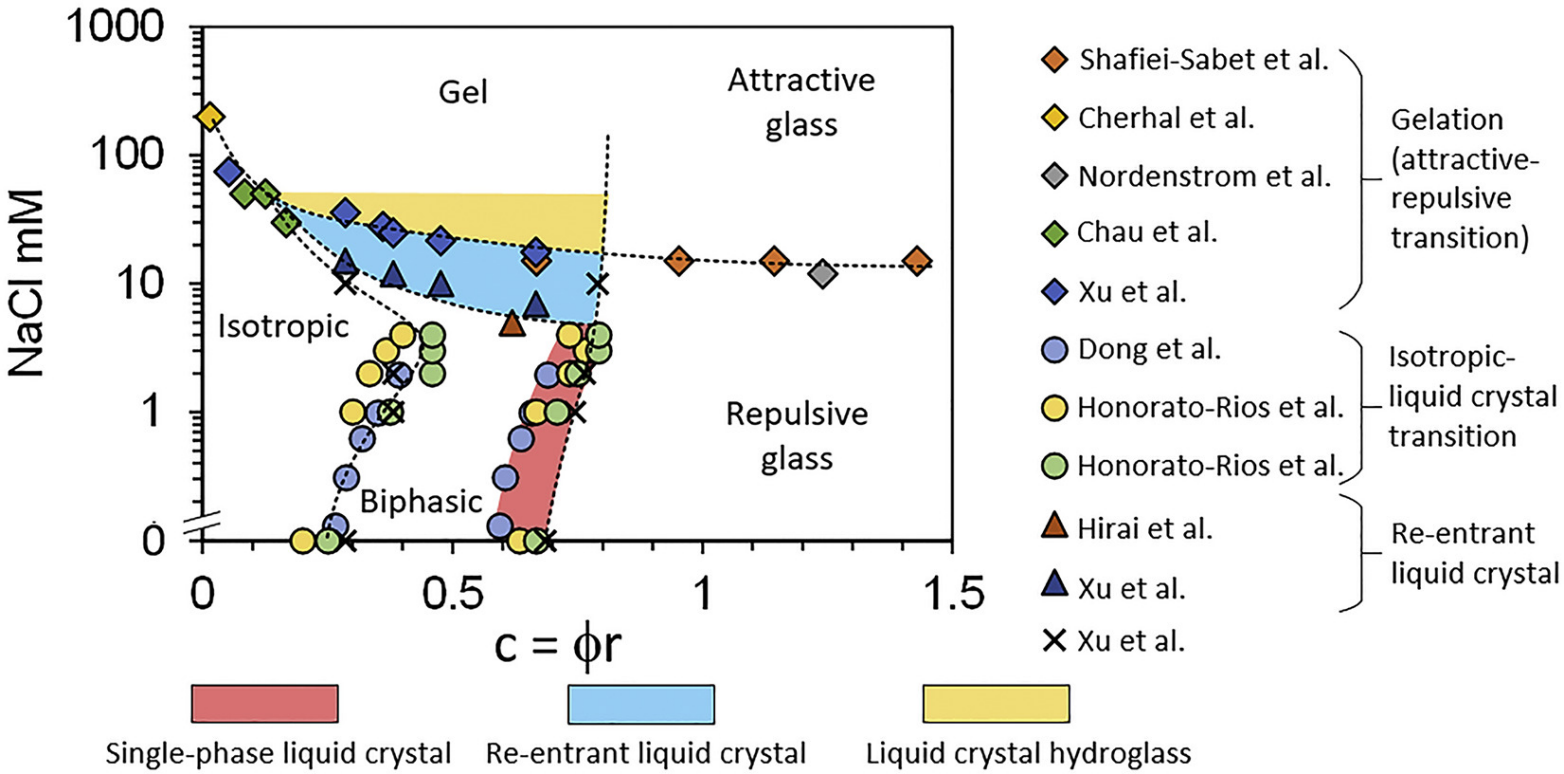
Generalized phase diagram of CNC suspensions as a function of renormalized CNC volume fraction (Φ̃ = Φ*a*, denoted ‘*c* = ϕ*r*’ by the authors) and added [NaCl] (note the lin-log scale of the axes). The effective volume fraction and aspect ratio of CNCs were not included in Φ̃. Conditions for liquid–solid and isotropic-liquid crystal transition are taken from experimental data reported in the literature, including Shafiei-Sabet et al.,^506^ Cherhal et al.,^455^ Nordenstro̷m et al.,^483^ Chau et al.,^507^ Dong et al.,^271^ Honorato-Rios et al.,^284^ Hirai et al.,^104^ and Xu et al.^293,373^ Dotted lines and points designated with a cross are phase boundaries determined from rheological measurements in ref (373). The conditions for a single-phase liquid crystal state are obtained from extrapolating the reported dependency of liquid crystal fraction on CNC volume fraction in refs (271 and 308). Adapted with permission from ref (264). Copyright 2019 Elsevier.

A significant contribution to the study of KA that justifies more attention came from the studies of Xu et al. on the rheological behavior of suspensions of CNCs,^344,373,486^ which were recently summarized in a review.^264^ They propose a generalized phase diagram for the arrested phases of CNC suspensions (reproduced in Figure 24), depending on the bare CNC volume fraction renormalized by their bare aspect ratio, Φ̃ = Φ*a*, and the salinity, expressed in terms of added free electrolyte, usually NaCl (see Figure 24). This unified view allows for a mapping of both the liquid crystalline behavior, with region labeled isotropic, biphasic (which is macroscopically defined but microscopically inaccessible) and fully liquid crystalline (cholesteric), and the rheological properties indicating arrested states with their nature (termed “repulsive glass” or “attractive glass” by the authors). The region at high ionic strength leads to gel samples, with an upper boundary in added NaCl that decreases at higher CNC concentration (not very apparent on the figure due to the lin-log scale of the axes). This decay can be understood from the increasing role of the counterions to the overall ionic strength as the CNC mass fraction increases. This region seems to stabilize between 10 and 20 mM, in agreement with observations from Capron et al.^454^ Next, this line is prolonged at higher volume fraction where the suspension is arrested into either an attractive or a repulsive glass, the nature of which can be discriminated using the discontinuity of the yield stress between the two regimes. This limit is located somewhere between 10 and 20 mM (or slightly above 20 mM according to Figure 22).^264,373^ The region at low CNC and NaCl concentration behaves as a viscous liquid, with a change to viscoelastic liquid at the CNC mass fraction increases, consistent with the increasing interaction between rods,^346^ and at higher volume fraction with the additional formation of a cholesteric phase, while even higher CNC mass fraction (keeping [NaCl] low) leads to a second transition into a repulsive glass. Notably, these two transitions are slightly dependent on [NaCl], allowing for re-entrant behaviors: near the first transition (ca. *c* ≈ 5 wt %), the sample can evolve at a fixed CNC volume fraction and increasing [NaCl] from a biphasic to an isotropic and again to an biphasic suspensions, and near the second transition (ca. *c* ≈ 9 wt %), the sample can evolve at a fixed CNC volume fraction and increasing [NaCl] from an arrested state (repulsive glass) to a relaxing state (viscoelastic liquid) and to an arrested state again (attractive gel).

At low salinity, increasing Φ̃ leads to a repulsive glass, quoting the observation of Honorato-Rios on fractionated CNCs. The observation of KA at identical volume fractions Φ for large or small aspect ratios results, once expressed in terms of Φ̃, as an earlier KA for small than for large CNCs, leading to a re-entrant behavior also visible in Figure 24. This invites a reinterpretation of the KA as a repulsive glass scenario, as labeled in the figure.

Regarding the terminology, the authors also chose to distinguish between the gels, at low Φ̃, from the attractive glasses at high Φ̃. In this nomenclature, very diluted gels retain their isotropic structure. For intermediate Φ̃, the produced gels have to cross first a re-entrant liquid crystalline region (marked in blue), and thus inherit from their alignment. These are termed “liquid crystal hydroglass” by the authors and correspond to the region marked in yellow. Finally, the region labeled “attractive glass” cannot be reached directly before entering first another arrested phase and thus have properties that are not uniquely defined.

**Figure 25:**
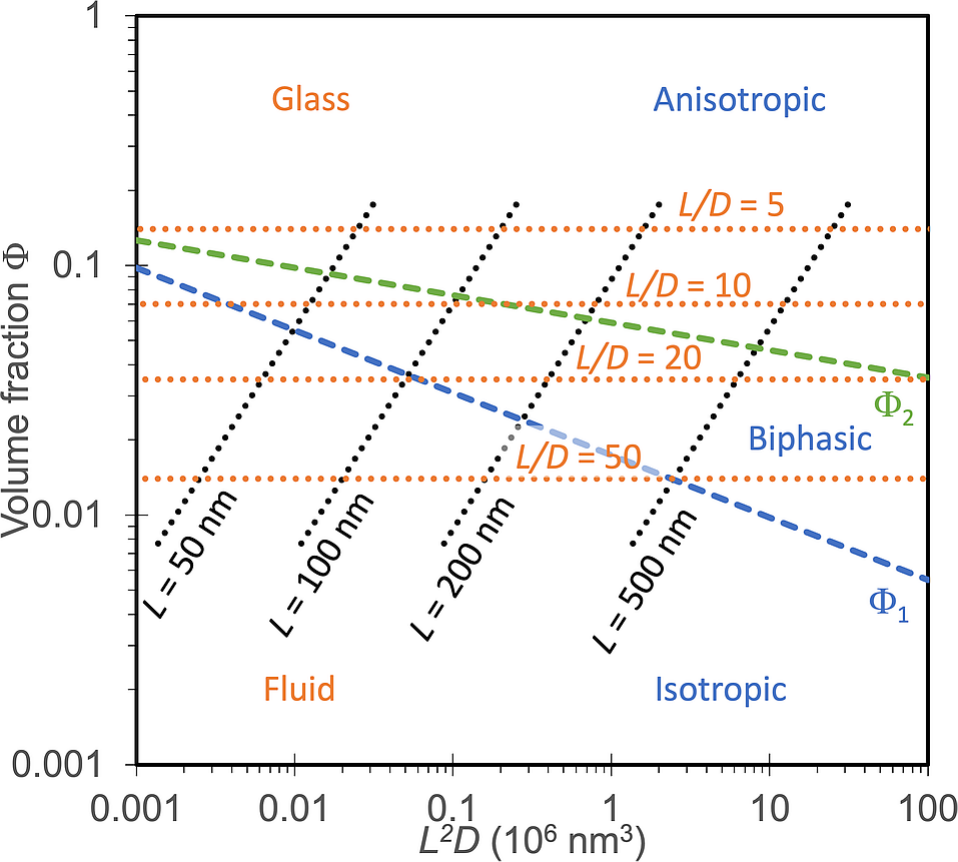
Proposed phase diagram for CNC suspensions in terms of CNC volume fraction and calculated particle excluded volume term *L*^2^*D*, assuming cylindrical particles. Dashed lines represent Φ_*b*1_ (blue) and Φ_*b*2_ (green) (see section 3.2.5 for further details). Boundaries of the glass transition are plotted as dotted lines, reported in a parametrized form to suit the axes: orange dotted lines represent the boundary for constant CNC aspect ratio a while varying particle length; black dotted lines represent the boundary for constant L while varying aspect ratio. Adapted with permission from ref (264). Copyright 2019 Elsevier.

Next, Xu et al. proposed that the aspect ratio is the main criterion for triggering KA in the isotropic state, also using the criterion from Solomon and Spicer (see section 5.4.1),^451^ while the phase transition would follow a different power law of the hard rod excluded volume of the CNCs (from an empirical rule they observed, see section 3.2.5 and Figure 13). The resulting diagram they obtain is reproduced in Figure 25 (with corrected axis units from original publication). This diagram is interesting as it proposes an alternative (but mostly qualitative and phenomenological) explanation for the observations that in cholesteric suspensions, shorter CNCs tend to become trapped into KA before they manage to form a liquid crystal phase. However, this criterion for the KA in isotropic phase assumes it is mostly due to a repulsive glass transition, which again invites reinterpretation of the observation from Honorato-Rios et al.^284^ Another interesting point is that for shorter CNCs, the biphasic region becomes narrower and occurs deeper into the glass region. A consequence of that is that the relaxation time of the shorter CNCs may become increasingly longer as Φ increases while still being able to relax. This can result in the incapacity to efficiently nucleate the cholesteric phase, pushing the system into the unstable spinodal regime.^345^

While the description provided by Xu et al. is interesting, a few issues remain unaddressed, such as the criterion of the time scale for the KA to occur, the effect of polydispersity and fractionation that could occur in parallel, the effect of the CNC charge, or the external aligning factors occurring in a suspension upon drying. Its formulation in terms of bare volume fraction and aspect ratio allows for using more easily measurable parameters but is difficult to bridge with theoretical concepts provided by the Onsager formalism. For both attractive and repulsive glasses, unidirectional (vertical) compression induces an alignment in the perpendicular plane (see section 6.1),^345^ which has been exploited to produce anisotropic hydrogels from CNFs.^508^ Most importantly, the relaxation or the locking of the twist between CNCs, which ultimately controls the pitch, is not discussed. Our current understanding of kinetic arrest in cholesteric CNC suspensions is still lacking and remains an active field of research.

#### 5.4.3 KA in Cholesteric CNC Suspensions

The specific case of KA in a cholesteric CNC suspension means that eventually the direction of the helical axis and the pitch will no longer equilibrate macroscopically and instead will be partially unable to evolve from the individual motions of the CNCs, but rather as the properties of a soft solid. This can also involve the locking of the size and morphology of the various cholesteric domains, the interruption of relaxation mechanisms such as coalescence and defect healing, which will have different impact depending on whether a cast suspension drying in a dish or a filled, sealed capillary is considered. In cast suspensions such as used to produce films, qualitative observations of these phenomena were reported using timely polymerization of acrylamide present in the suspension.^333,336^ The final structure of the films, as well as their optical response, inherit from the effect of these interrupted relaxations.^334,345,416^ Quantitative detections can rely on rheological methods such as discussed in the previous section, however they might correspond to different criteria: a gel-like rheological response could be identified at a certain Φ_KA_ for a certain probing stimulus (e.g., detected upon oscillatory shear independently of the chosen frequency and amplitude), while another Φ_KA_ for the KA of the cholesteric structure upon solvent evaporation could occur for the particular case of the internal stresses occurring upon drying, and arising from the departure from the most energetically favorable cholesteric structure at a rate dictated by the evaporation conditions. This invites us to introduce the notion of *KA of the cholesteric structure* to distinguish it from other related yet distinct concepts.

The quantitative observation of the KA of the cholesteric structure is crucial to determine the final optical properties of the films, as it will evolve very differently upon solvent evaporation once it becomes “arrested” (see section 6). It is important at this point to clarify the terminology used to describe KA in the context of cholesterics. First, a twisting angle φ can be defined, which corresponds to the average angle expected between neighboring rods along the helical axis. This angle is given by φ = 2*πr*_*φ*_/*p*, where *r*_*φ*_ is the typical distance between two neighboring CNCs along the helical axis. In the arrested state, this twist angle is constant (i.e., φ = φ_KA_) in absence of shear. Upon further solvent evaporation and sample contraction, the mutual orientation between the rods will not relax, while the pitch, defined as *p*(Φ) = 2*πr*_*φ*_(Φ)/φ_KA_, will “evolve” only because the distance *r*_*φ*_(Φ) will continue to decrease. Consequently, it is useful to think of the KA of the cholesteric structure as a whole in terms of the KA of the twist angle φ, rather than the “KA of the pitch”.

Upon solvent evaporation prior to kinetic arrest, the pitch must decrease in response to the changing volume fraction of the CNCs to minimize the free energy of the system. If the pitch dependence with the volume fraction follows an apparent power law steeper than , much like discussed in section 4.3, the cholesteric domain itself has to adjust the local φ and thus to reconfigure itself internally as it contracts, to keep up with its most thermodynamically stable pitch value. Slow relaxation times for the pitch value are known to occur in cholesterics and scale as
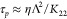
36where η represents the viscosity of the cholesteric, Λ the relevant distance over which the volume fraction of mesogen varies (e.g., the wavelength of an applied ultrasound, or a compressed tactoid or cholesteric domain expelling solvent), and *K*_22_ the twist elastic constant.^509^ Importantly, this relaxation time will eventually be insufficient for pitch relaxation, since the drying rate of the suspension in a dish is near constant and leads to a hyperbolic increase of the CNC concentration over time, which will eventually outcompete *τ*_*p*_. From eq 36, the relaxation time is proportional to the suspension viscosity, which also leads to an increase in *τ*_*p*_ over time, reinforcing this effect. Consequently, as a suspension dries, the time scale of pitch relaxation is outpaced by the drying rate before the viscosity diverged at the onset of KA as defined by rheology. This phenomenon can also explain the blue shifting effect caused by a mask placed above the suspension as it dries,^510^ or the red-shifting effect of heating locally the drying suspension,^431^ as will be discussed in section 9.1.3.

Ignoring prefactors, Equation 36 shows that the time required for a uniform cholesteric region to adjust its pitch will scale quadratically with its size. This effect arises because the twist angle φ between neighboring rods cannot relax locally without a coordinated rotation of all the rods to wind up the tactoid or cholesteric domain to further reduce its pitch. This reconfiguration resembles a physical twisting of the tactoid, and requires a rotation of the director that scales with the physical dimension of the tactoid. The coalescence of tactoids without the merging of their local cholesteric alignment will add additional topological constraints that will lock the angle φ in each tactoid at various stage of their pitch decreasing trajectories *p*(Φ).

Qualitatively, this will result in a pitch value that will lag behind its most thermodynamically stable value, and this discrepancy should depend on the size of the domains. A polydispersity in domain size may thus translate into a spreading of the cholesteric pitch values as the suspension is approaching KA. This prediction may explain why samples with larger domains tend to present larger pitch values, compared to polydomain ones.^288^ This can also explain the broadness of the reflection peaks of typical CNC films, despite their small birefringence (Δ*n*/*n*_Ch_ ≈ 0.05). This also suggests that if a suspension forms a macroscopic monodomain, it will struggle to adjust its angle φ as the solvent evaporates. Future studies could investigate this point by a quantitative analysis of the pitch in well-aligned spherical structures inside emulsion droplets that are slowly shrinking due to solvent removal.

##### 5.4.3.1 Effects of Domain Size on Pitch Relaxation in Emulsion Droplets

Starting from a Frank-Pryce-like (FP-like) organization of the CNCs, the further contraction of the droplet upon solvent loss through the oil (similar to an evaporation) leads to the decrease of the pitch with the volume fraction with a power law *p* ∝ Φ^–ν^ with ν ≈ 1 before the KA is reached. Curiously, the same suspension observed in capillaries showed a steeper pitch dependence with Φ at higher volume fraction (still before KA), although not clearly a power law if including the lower volume fraction range. When the same CNCs were assembled with a higher salt-to-CNC ratio, the tactoids did not assemble into a regular FP-like monodomain and the pitch agreed with the data measured in the corresponding capillaries.^288^ This suggests that the pitch in the FP-like structure, while not being completely arrested, showed some lagging and was not able to easily contract as it would have in the capillary. This was ascribed by the authors to the larger size of the cholesteric domain in the FP-like structure, which requires more time for the pitch to adjust due to long-range topological constraints. Using the dependence of the relaxation time provided above and ignoring prefactors, an initial droplet of diameter Ø ≈ 140 μm at Φ ≈ 4.7 vol % contracting by 25 μm h^–1^ experiences an increasing strain rate ε̇ as the droplet becomes smaller (for Φ ≈ 7.5 vol %, ε̇ ≈ −5.8 × 10^–5^ s^–1^, and for Φ ≈ 12 vol %, ε̇ ≈ −7 × 10^–5^ s^–1^), which puts a conditional cutoff value for the viscosity *η*_*c*_ ≈ 2 × 10^2^ Pa·s to distinguish the regimes of perfect synchronicity from regimes of ineffective relaxation (assuming *K*_22_ = 0.04 pN from ref (244)). In a separate study, rheological measurements performed under controlled shear rate γ̇ showed steady shear viscosities η(γ̇ ≤ 0.1) ≈ 0.1–1 Pa·s for CNC suspensions in the range Φ ≈ 5.0–7.5 vol %, suggesting prompt relaxation of the pitch.^291^ Instead, η(γ̇ = 0.1) ≈ 1–10 Pa·s for CNC suspensions in the range Φ ≈ 7.5–10 vol %, and a very weak variation at lower shear rate γ̇ suggesting η(10 vol %, γ̇ = 10^–5^) ≲ 100 Pa·s, which is much closer to the rough estimation of *η*_*c*_ proposed above. Finishing with this analysis, η(γ̇ = 0.1) ≈ 200 Pa·s for a CNC suspensions at Φ ≈ 12.5 vol % with a scaling η ≈ 1/γ̇, suggesting η(γ̇ = 10^–5^) ≈ 2 10^5^ Pa·s that would completely lock the pitch dependence to its scaling with its compression factor α_iso_, as detailed in section 6.2.2.

##### 5.4.3.2 Effects of Domain Size on Final Pitch in Films

The fact that the pitch relaxation time depends on the size of the domain could explain contradictory observations regarding the effect of the CNC size on the final pitch: recent observations of CNC suspensions at equilibrium showed that larger CNCs, which have also a larger 3D aspect ratio and accumulate in the anisotropic phase of a biphasic suspension, form cholesteric phases with smaller pitch values.^272,284^ This is also observed when ultrasonication is used to produce suspensions of different sizes of CNCs, with the larger CNCs giving the smaller pitch values.^89^ This is also supported by DFT and MC simulations on twisted hard-bundles using CNC dimensions found in experimental samples.^273^ It would thus be expected that they should lead to smaller pitch values in films. However, Revol et al. reported (however without providing data) that when using fractionated CNCs, longer rods from the anisotropic phase gave larger pitches in dry films, while shorter gave smaller pitch values.^21^ These observations can be reconciled by considering the effect of local fractionation in a drying suspension. If upon casting, the longer rods phase-separate first and form a uniform cholesteric monodomain at the bottom of the dish, this domain would require more time to adjust its pitch upon solvent evaporation, and could be trapped at KA in a larger-than-equilibrium pitch. The pitch is thus dependent on the competition between the internal relaxation time of the cholesteric phase (which depends on intensive quantities like concentration, temperature, ionic strength, CNC dimensions etc. but also extensive quantities like the size of the cholesteric domains), and the drying time (which also depends on both intensive and extensive quantities such as e.g. the initial volume fraction and the suspension volume, as illustrated in section 9.1.3).

This dependence of pitch relaxation on domain size could also account for the conflicting observations that applying a vertical magnetic field can lead to a smaller pitch (by improving the vertical alignment of the cholesteric domains, the pitch shrinks more efficiently upon vertical compression, and the resulting pitch is smaller, as observed here for long drying times^161^) or a larger pitch (as it allows domains of various orientations to align together and merge into larger domains that then struggle to adjust their pitch upon drying toward an equilibrium value, as observed here for short drying time, and in a fashion that depends on the drying rate^511^). An important consequence of this is that the uniform cholesteric layer at the bottom of the dish, because of its vertical alignment, should only contribute to the specular optical response of the film, while the smaller disconnected domains should have a contribution in both specular and off-specular conditions, as will be shown in section 7.4.4.

It is important to note that the pitch relaxation upon volume fraction increase can have another relaxation pathway, which can occur before or after KA, namely the spontaneous shear of the cholesteric, leading at a larger scale to buckling. Such shear occurs in molecular liquid crystals where no KA occurs, and are selected because the energy cost of long-range bend distortions is less than the twist energy cost of under-winding (see section 4.3 on elastic instabilities). For CNCs, current observations suggests that these buckling events most often occur after KA and will be discussed in section 6.

### 5.5 Summary

Kinetic arrest of a colloidal cholesteric sample is a complex phenomenon that involves the lack of relaxation toward equilibrium, with some degrees of freedom that can become locked before others, progressively turning a flowing suspension into what will become a solid film. The understanding of KA in cholesteric CNC suspensions as it occurs upon casting is still limited, and many questions about the mechanisms of this transition are still open (see discussion in section 12.2).

While the most common methods to investigate the KA in soft systems were presented in section 5.3, the case of cholesteric colloidal structures such as those formed by CNCs offer an additional, indirect way to specifically probe the KA of the cholesteric structure. In absence of the relaxation of the twist angle φ between neighboring CNCs in an arrested cholesteric, the pitch in vertically aligned domains will evolve as *p* ∝ *r*_*φ*_, i.e., linearly with the distance between the neighboring CNCs along the helical axis. Since this will depend on the geometry of the compression, the pitch will depart from its natural, thermodynamically most stable value in a way that will depend on the geometry. This can provide a tool to access the pitch value at the point of KA, as well as the volume fraction at which it occurs, complementing rheological data, as detailed in the following section 6.

## 6 Helicoidal Structures after Kinetic Arrest: The Role of Geometry

The CNC concentration at KA, typically around 8–16 vol %, corresponds to a sample that contains much more solvent than cellulose. Consequently, a substantial reduction in volume is required as the arrested suspension dries into a solid film.

For CNC photonic materials, the KA of the cholesteric structure will be of particular interest, which justifies that throughout the section 6, KA will refer specifically to the KA of the cholesteric structure (as discussed in section 5.4.3). Indeed, from that point onward, the pitch evolution upon further solvent evaporation will no longer be driven by the minimization of the internal elastic stresses arising from the deformation of the cholesteric liquid crystalline phase, but rather by the geometry of the system and its evolution upon drying. These geometric effects can be divided into two categories: bulk compression of the structure, which typically obeys scaling law behavior, and buckling, which may modulate the local compression.

This section discusses these geometric effects for dish-cast CNC suspensions and spherical emulsion droplets, using theoretical descriptions when available and illustrating the key concepts using experimental examples. Note that several geometric effects that act before KA are discussed elsewhere in this review, including liquid crystal anchoring (section 4.2) and wetting and capillary forces (section 9.1.2).

### 6.1 Compression and Buckling in Dish-Cast Films

As a dish-cast suspension is horizontally pinned to the walls of the Petri dish, evaporation of water requires a vertical compression of the sample. A model of such compression and its consequences on the initial cholesteric structure in the suspension was recently proposed,^161,416^ based on the assumption that after KA, compression will distort the pitch according to a simple homothetic rescaling of the director field **n**(**r**). This assumption suggests that KA for the cholesteric pitch coincides with KA for the rheological properties of suspension. However, this assumption may not be exactly true, which would require a refinement of the previous model. In this review, we thus generalize the results of ref (416) to allow for the possibility of horizontal compression by adopting the notation from ref (512). In this review, we propose using the notation from another work to generalize the conclusion from this work (ref (416) for cases where a small horizontal compression is also possible, justifying to explicitly provide some intermediate results.^512^

#### 6.1.1 Theoretical Formalism for Anisotropic Compression

In general, the compression of the director field can be described by a compression matrix α̿:
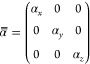
37where 0 < *α*_*i*_ ≤ 1. After compression, the new director field is **n**′(**r**′) = α̿ **n**(**r**), where **r**′ **=** α̿ **r**. The contraction in sample volume is given by det(α̿) = *α*_*x*_*α*_*y*_*α*_*z*_.

For isotropic compression, where *α*_*x*_ = *α*_*y*_ = *α*_*z*_ = α, the homothetic transformation is a simple rescaling of the structure by a factor α in each direction, without distortion. This results in a preserved orientation of the different helical axes in the sample from the arrested state until the final dry state (**m**′(**r**′) = **m**(**r**)), and a rescaling of the pitch as *p*′ = *α p* (see section 6.2.1 for further discussion). In the following discussion, the pitch in the final state, *p*′ will be denoted *p*_f_, while the initial pitch, *p* will be referred to as *p*_KA_ (indicating the pitch at KA), with matching suffixes for related quantities.

**Figure 26:**
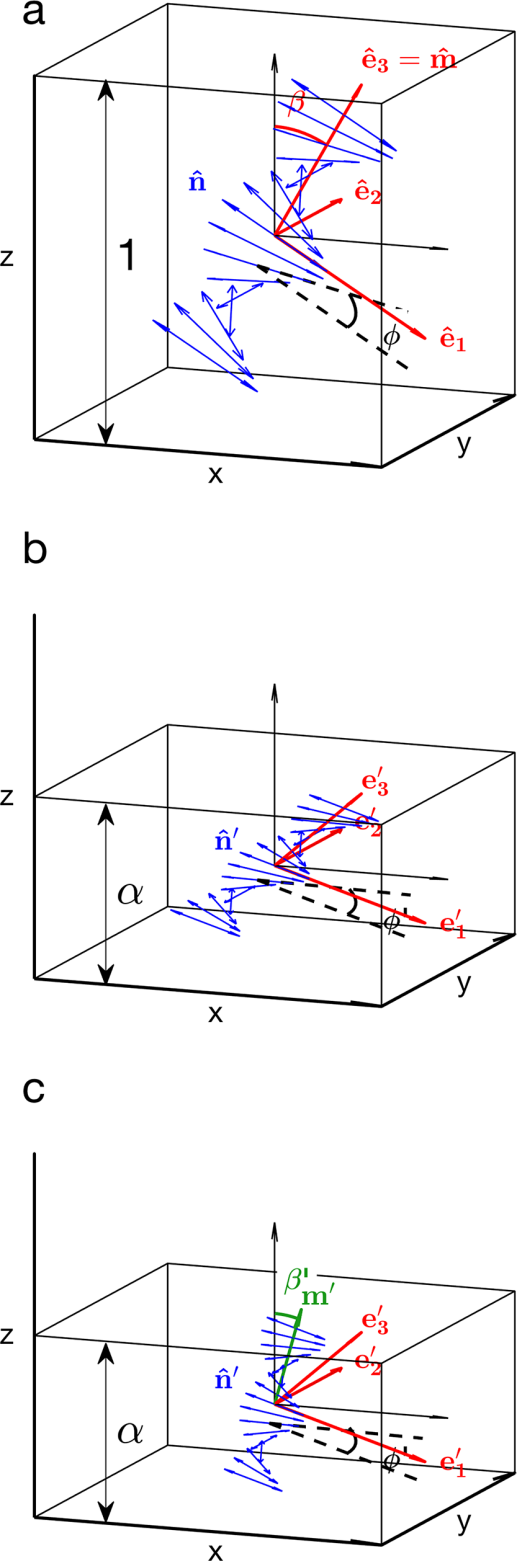
Compression of a helical domain. Schematic diagram of a tilted, left-handed cholesteric domain (a) before deformation, oriented along ***m***, (b) after an anisotropic, unidirectional compression along ***ẑ***, scaling with a factor α (i.e., *α*_*z*_ in our notations) and (c) after redefining the helical axis as ***m***′. The directors ***n*** and ***n***′ are depicted by double-headed arrows (in blue) to account for their symmetry by inversion. Reproduced with permission from ref (416). Copyright 2019 American Physical Society.

In general, nonisotropic compression of the structure can cause distortion of the director field, a reorientation of the helical axes and a nonlinear variation of the pitch (Figure 26). Without loss of generality, the coordinate system can be chosen so that a given helicoidal domain has a helical axis **m** tilted away from the vertical direction (**z**) by an initial angle β in the (**x**, **z**) plane. The final orientation of the helical domain after compression is then given by
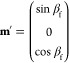
38where
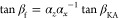
39

The final pitch *p*_f_ is given by
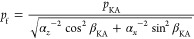
40which can also be expressed in terms of the final helical tilt as
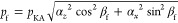
41

The helical modulation of the director is no longer sinusoidal, and instead varies as
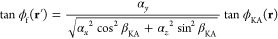
42which can be rewritten as
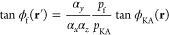
43

Here, the angle ϕ_KA_(**r**) = *q* (**m** · **r**) is the phase of the helically modulated director field **n**(**r**).

For an arrested suspension drying in a dish, the assumption 0 < *α*_*z*_ ≪ (*α*_*x*_, *α*_*y*_) ≈ 1 is valid, while for the case of a perfectly unidirectional compression along the **z** axis, this simplifies exactly to *α*_*x*_ = *α*_*y*_ = 1 and only remaining parameter is 0 < *α*_*z*_ ≪ 1.

Finally, the CNC volume fraction at KA is given by
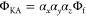
44where Φ_f_ is the CNC volume fraction in the final film. It is convenient to define an effective compression ratio α_eff_ = α_*z*_α_*x*_^–1^ between the **x** and **z** axes, as this quantity can be estimated from the pitch variation *p*(β_f_) of a photonic film using angle-resolved optical spectroscopy (see section 7.4.4). In terms of α_eff_, the CNC volume fraction at KA is
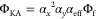
45and for *α*_*x*_ ≈ *α*_*y*_ ≈ 1, this simplifies to Φ_KA_ ≈ *α*_*z*_Φ_f_ and α_eff_ ≈ *α*_*z*_. For a pure CNC photonic film, where Φ_f_ ≈ 1, then
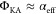
46allowing for an estimation of the Φ_KA_.^437^ However, small contractions along **x** and **y**, as well as traces of water in the final film (i.e., Φ_f_ ≲ 1), can lead to an overestimation of Φ_KA_.^416^ If a nonvolatile cosolvent is present in the suspension, the compression of the structure upon drying is reduced, as the cosolvent cannot be removed. In this case the final CNC volume fraction can be approximated to
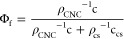
47where *c* and *c*_cs_ are the initial mass fraction of CNC and nonvolatile cosolvent in the cast suspension, respectively. From eq 47, it can be deduced that the addition of cosolvent reduces the final CNC volume fraction. Therefore, assuming that the pitch and CNC volume fraction at KA are not affected by the presence of cosolvent, the ratio α_*x*_^2^α_*y*_α_eff_ = Φ_KA_/Φ_f_ will increase toward 1, indicating that the structure is less compressed and corresponding to a pitch increase with increasing cosolvent concentration. Notably, the red-shift observed upon swelling of a photonic CNC film upon solvent uptake (e.g., upon immersion or exposure to solvent vapor), as discussed further in sections 10.1 and 10.2.2, is essentially the opposite effect of drying: solvent uptake corresponds to the increase of *c*_cs_ and the decrease of the effective Φ_f_, and thus reduces the compression of the structure and increases the pitch, while the optical index of the chosen solvent affects in a lesser degree the reflected wavelength and its intensity.

Finally, the compression matrix formalism can also be used to understand the effect of an anisotropic swelling or deswelling of a cholesteric elastomer,^513^ or the anisotropic compression or stretch of a cholesteric elastomer at constant volume (incompressible), where *α*_*x*_*α*_*y*_*α*_*z*_ = 1, which in first approximation implies .^512,514^ For a flowing molecular cholesteric interacting with a network of cross-linked polymeric matrix, more sophisticated models have also been developed.^515^

#### 6.1.2 Consequences of Anisotropic Compression

The distortion of the structure by compression has several important consequences for the optical response of the resulting photonic films.

First, this model highlights the importance of vertical compression in enabling the pitch to reach the submicron range necessary for visible structural color. Other methods to solidify the structure without solvent removal, such as photopolymerization, produce structures with pitch values in the micron range. However, the decrease in pitch due to vertical compression depends on the original tilt of the domain β_KA_, as shown by Equation 40. Even if the structure has the same pitch across the whole sample at KA, variation in the helical axis tilt angle, combined with compression, will lead to a range of pitches in the resulting film. The smallest pitch is expected for vertically aligned domains (for β_KA_ = β_f_ = 0°, *p*_f_ = *α*_*z*_*p*_KA_), whereas horizontally oriented domains would, according to this model, not be compressed at all (for β_KA_ = β_f_ = 90°, *p*_f_ = *α*_*x*_*p*_KA_ ≈ *p*_KA_). The orientation distribution function, describing the proportion of domains pointing in a certain range of polar angles β_f_, will thus not be uniform, even if the initial distribution were uniform (i.e., if the orientation of **m** across the arrested sample were overall isotropic). Nonisotropic orientation distributions, which may arise from anchoring or an external magnetic field, can also be considered.^416^

An important take-home message, which stems from the above and is worth reiterating here, is that a CNC film, unless perfectly monodomain, does *not have a unique pitch value*, but rather a *distribution of p*_f_(β_f_). When a pitch value is reported for a film, it is usually the value *p*_f_ = *α*_*z*_*p*_KA_ of the well-aligned domains at β_KA_ = β_f_ = 0°, responsible for the photonic response in specular conditions (see section 7).

A second consequence of vertical compression is to reorient the helicoidal domains toward the vertical direction (i.e., β_f_ ≤ β_KA_ due to eq 39). As a quantitative example, a domain with β_KA_= 45**°** compressed 10-fold (*α*_*z*_ = 0.1, *α*_*x*_ = *α*_*y*_ = 1) will have a final tilt angle of β_f_ = 5.7°. Consequently, the reflection from CNC photonic films is typically concentrated around the specular angle.

A more subtle consequence of distortion is the nonsinusoidal variation of the director (eq 42). It is worth reconsidering the choice of the terminology to describe this structure, since tilted distorted domains no longer display true cholesteric ordering in the strictest sense. The structure could be described as a “chiral plywood” and, for the sake of clarity, will only be referred to as “helicoidal” henceforth (as discussed in section 3.1.4). The optical consequences of the nonsinusoidal director are the appearance of additional, higher order reflection bands (which would not be observed along the helical axis for a tilted but undistorted cholesteric structure) and a net linear birefringence in the (**x**, **y**) plane (as discussed further in section 7.3.6).

**Figure 27:**
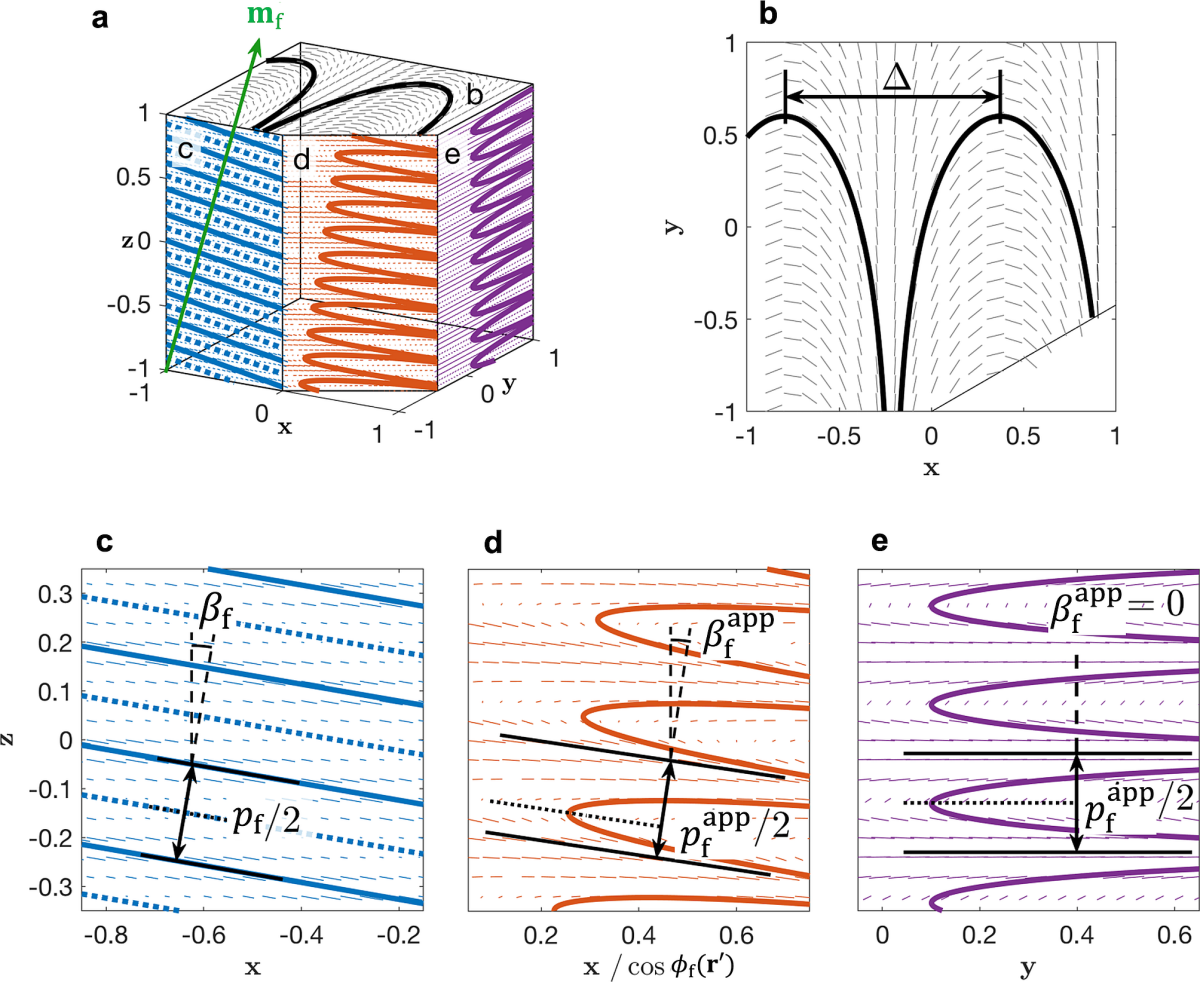
Multiple top- and cross-sectional views of the director field of an initially cholesteric domain with an original orientation β_KA_ = 60° in the (**x**, **z**) plane, after a vertical compression by α_*z*_ = 0.1, α_*x*_ = α_*y*_ = 1, and finally tilted by β_f_ ≈ 9.8°, adapted from ref (416). The local projection of the orientation of **n**′ (**r**′) onto each section is depicted by small dashes. The aligned pattern it produces is highlighted with thick full lines (e.g., the Bouligand arches) while thick dotted lines indicate director pointing normal to the section plane. (a) Corner view in perspective, (b) top view, generating a pattern of periodicity Δ, usually in the micron range, (c) cross-section in the (**x**, **z**) plane containing **m**′ (**r**′) (i.e., at an azimuthal angle φ = 0°), (d) cross-section at an azimuthal angle φ = 30°, leading to asymmetric Bouligand arches, arising from the distortion of the cholesteric order (the dotted line highlights the asymmetry), (e) cross-section in the (**y**, **z**) plane (i.e., at an azimuthal angle φ = 90°), leading to symmetric Bouligand arches despite the distortion. Reproduced with permission from ref (416). Copyright 2019 American Physical Society.

The resulting three-dimensional structure is best illustrated in Figure 27, where different top and cross-sectional views are shown on a single distorted domain. From this figure, the final tilt and final pitch are best assessed when viewed in the (**x**, **z**) plane containing **m**′, while other cross-sections only present apparent values for the tilt β_f_^app^ and the pitch *p*_f_^app^, given by
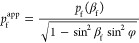
48
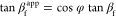
49where φ indicates the azimuthal angle of the cross-section with respect to **m**′, with φ = 0° in the (**x**, **z**) and φ = 90° in the (**y**, **z**) plane. Note that in this plane, the characteristic arched pattern, commonly referred to as the Bouligand arches,^516^ is not visible; instead, a stratified pattern is observed (often wrongly ascribed to nonexisting “layers” in the cholesteric structure), which can be seen in SEM and due to the periodic roughness variation of the film when fractured. More interestingly, in a cross-section made at an azimuthal angle φ ≠ (0°, 90°, 180°, 270°), the Bouligand arches appear asymmetric due to the distortion, which can be directly observed on cross-sections of CNC films by SEM.^416^

The variable compression of tilted helicoidal domains, and therefore the fact that there is not a unique *p*_f_ value in a film but rather a distribution of *p*_f_(β_f_), is often overlooked when cross-sectional SEM is used to naively estimate the pitch in a CNC film. Moreover, it is difficult to estimate the tilt of a domain from the cross-sectional pattern, as only partial information is accessible: an apparent β_f_^app^ ≈ 0 with Bouligand arches indicates some β_f_ > 0, while a tilted β_f_^app^ indicates a lower boundary for β_f_ (because 0 < β_f_^app^ < β_f_). Regarding the apparent pitch, the dependence with both β_f_ and φ appears weak for typical β_f_ values (e.g., for β_f_ = 10°, 1–sin^2^ β_f_ ≈ 0.97) and justifies *a priori* that any measurement of the periodicity pattern is representative of the real pitch *p*_f_ of that domain. However, since the pitch varies strongly with the real tilt β_f_, measuring the average *p*_f_ will be highly affected by the values measured on tilted domains and will contribute to produce an average pitch that is higher than the smallest pitch *p*_f_(0) (i.e, the one responsible for the specular optical response), and with a standard deviation that results from the angular spreading of *f*(β_f_) in the final film.^416^ For instance, for β_f_ = 10° the ratio between the two pitch values is *p*_f_(10°)/*p*_f_(0°) ≈ 1.5 for *α*_*z*_ = 0.15, and nearly *p*_f_(10°)/*p*_f_(0°) ≈ 2 for *α*_*z*_ = 0.1 (assuming *α*_*x*_ = *α*_*y*_ = 1).

The top view shows that a tilted domain also causes the appearance of a pattern of periodicity Δ that is reminiscent of the fingerprint pattern observed in top view in the still liquid crystalline suspension. For that reason, this is often believed to be equal to half the pitch of the film in many publications. According to this model, the periodicity Δ does not depend on *α*_*z*_ and has the form
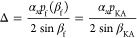
50

From eq 50, it is clear that for small tilt angles β_f_, values of Δ, much greater than *p*_*f*_, are expected. This can also be expressed in terms of the pitch in the arrested suspension *p*_KA_, but multiplied by a term 1/sin β_KA_ to account for the orientation relative to the horizontal focal plane of the optical microscope (as well as by a correcting factor *α*_*x*_ ≈ 1).

While this model appears satisfactory for explaining the optical properties of films containing domains with small values of β_f_, it is unable to predict the wavy patterns observed in highly tilted domains, suggesting buckling of the structure upon vertical compression, which will be discussed in the next section.

#### 6.1.3 Buckling Phenomena in Dish-Cast Films

**Figure 28:**
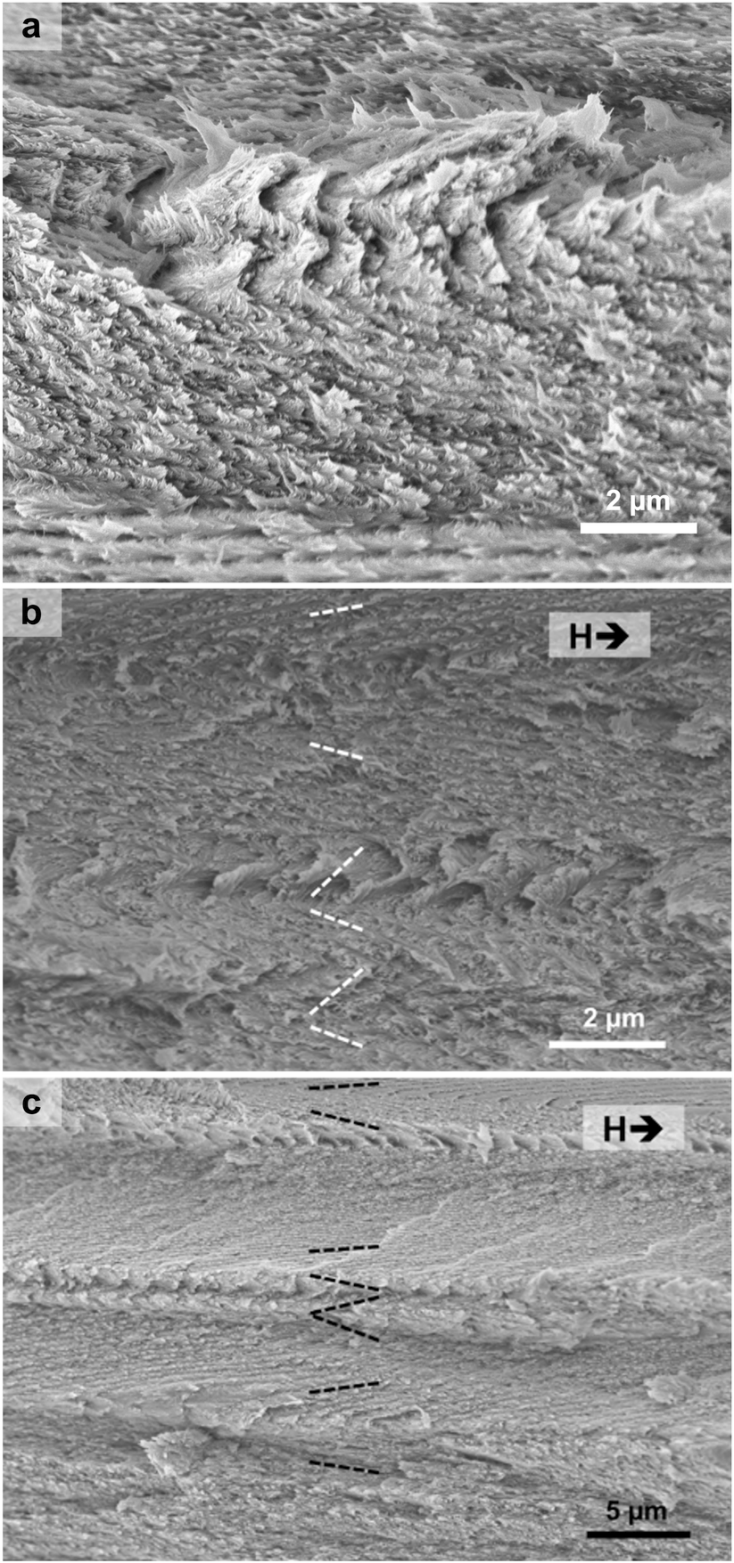
Cross-sectional SEM images of CNC films with buckled structures. (a) Buckling on a small domain. Reproduced with permission from ref (416). Copyright 2019 American Physical Society. (b, c) Buckling on large domains obtained by magnetic alignment in a horizontal field (field direction indicated by the arrow. Reproduced with permission from ref (161) under CC-BY. Copyright 2017 The Authors.

Cross-sectional SEM of CNC films reveals buckling of the structure for cholesteric domains that were initially highly tilted prior to vertical compression (Figure 28). However, similar buckling patterns were also observed for smaller tilts when the domains were much larger.^161^ This buckled morphology is reminiscent of multilayered geological formations, where alternating soft and stiff rock layers wrinkle and fold into large buckled vertical bands of opposite tilts in response to a horizontal compression (i.e., with a component in the plane of the initial layers).^517^ In the analogous case of CNC cholesteric domains, regions where the director is aligned with the compression direction are expected to be less compressible than regions where the director is perpendicular, creating periodic stiff and soft regions. The vertical compression then causes a buckling of horizontal bands of opposite tilts (for more about buckling in other geometries, see section 6.2.2).

### 6.2 Compression and Buckling in Emulsion Droplets

**Figure 29:**
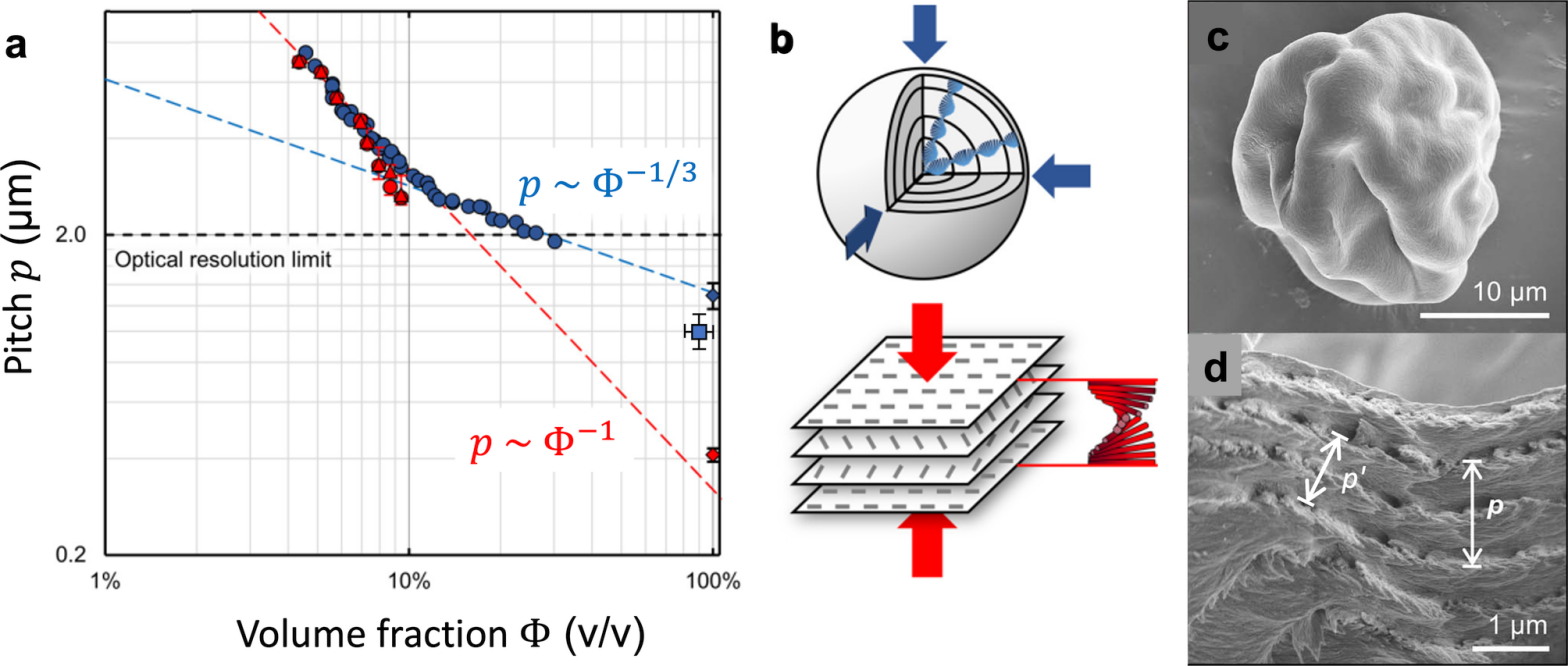
(a) Scaling of the pitch *p* vs CNC volume fraction Φ on a log–log scale, showing the sharp transition in power law behavior *p* ∝ Φ^–ν^ from exponent ν ≈ 1 to ν = 1/3 at the onset of KA around *Φ*_*KA*_ ≈ 12 vol %. The two diamonds represent the pitch measurements by SEM reported for the microparticles (in blue corresponding to *p* in d) and for the film (in red), assuming *Φ*_*f*_ ≈ 1 in both cases. The blue square represents the updated value for the pitch measurement by SEM in the microparticle after reinterpreting the data (corresponding to *p*′ in d and section 6.2.2). Note that the capillary data (red circles) show steeper decrease than ν ≈ 1, indicating that the pitch in the FP-like droplet begins to lag behind equilibrium before KA is reached (above Φ ≈ 7.5 vol %). (b) Comparison of compression in a spherical geometry (blue) and the unidirectional compression in a film (red). (c) SEM of the final microparticle, with visible buckling. (d) cross-sectional SEM with highlighted *p* reported in the article (approx. 1.3 μm, measured in the hinge) and *p*′ if measured in the limb (1 ± 0.1 μm, see section 6.2.2). Adapted with permission from ref (288) under CC-BY. Copyright 2016 The Authors.

As discussed in sections 3.1.3 and 9.2.3, the self-organization of CNCs in water-in-oil emulsion droplets leads to a Frank-Pryce-like (FP-like) cholesteric arrangement, with a radial alignment of the helical axis and a small isotropic core connected to the surface by disclination lines. For a drying droplet exhibiting this FP-like structure, the evolution of pitch with CNC volume fraction shows a sharp transition from one power law at low concentration to a power law with exponent ν = 1/3 at higher concentrations, as shown in Figure 29a. This sharp transition can be attributed to *KA of the cholesteric structure* (see section 5.4.3), after which the orientation of each CNC with respect to its closest neighbors is locked. Note that this method of determining the point of KA requires that the pitch at KA is large enough to be resolved by POM (i.e., above the optical resolution limit of ca. 1 μm for half a pitch).^288^

The evolution of pitch directly after KA can be attributed to a uniform isotropic contraction of the spherical structure, which differs from the rather unidirectional compression expected in a film (Figure 29b). However, SEM imaging of the final dried particles shows clear evidence of structural buckling, leading to pitch variation (Figure 29c,d). The following sections discussed these two effects (isotropic compression and buckling) in greater detail.

#### 6.2.1 Theoretical Formalism for Isotropic Compression

The compression of a spherical cholesteric structure after KA, irrespective of the FP-like arrangement, can be modeled as a uniform isotropic spherical contraction. As the CNCs can no longer relax individually, the angle between two neighboring CNCs is fixed and only the distance between them decreases upon further drying.^288^ This model is valid for any spherical geometry where the pitch is much smaller than the diameter, so that the core and the disclination lines can be safely neglected.

The contraction in the spherical geometry can be described by a compression matrix
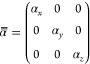
51where *α*_*x*_ = *α*_*y*_ = *α*_*z*_ = α, and so the homothetic transformation is a simple rescaling without distortion, by the quantity (*α*_*x*_*α*_*y*_*α*_*z*_)^1/3^ = α in each direction, as mentioned earlier. In this case, the FP-like configuration of the CNCs is preserved, but with the pitch rescaling as
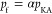
52

The volume fraction of CNC at the kinetic arrest is then given by
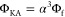
53where Φ_f_ is the volume fraction in the dry film.

This relationship can be rewritten to express the pitch *p*_f_ expected in the final microparticle obtained from a uniformly dried droplet with an FP-like structure:
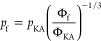
54where the experimentally observed power law dependence with the exponent ν = 1/3 is retrieved. Since Φ_KA_ < Φ_f_ ≲ 1, it implies that *p*_KA_Φ_KA_^1/3^ ≲ *p*_f_ < *p*_KA_, so that the final pitch cannot decrease much lower than a factor Φ_KA_^1/3^. The final pitch is therefore much greater than that obtained by unidirectional compression: even for a value of Φ_KA_ as low as 5 vol % (i.e., approx. 3 wt %), which is an unrealistically early KA if cholesteric assembly is also desired, the reduction in pitch is, at best, a factor *p*_f_/*p*_KA_ ≈ 0.35. As the pitch at KA is typically ≳2 μm, the isotropic spherical contraction is therefore insufficient to reduce the pitch into the 250–500 nm range necessary for visible structural color. Consequently, microparticles assembled from an FP-like organization of CNCs, which contract primarily by isotropic compression and have only moderate buckling (Figure 29c), exhibit a micron-scale final pitch (Figure 29d) and appear colorless. However, stronger buckling of spherical emulsion droplets can be exploited to produce microparticles with a small enough pitch to reflect light in the visible range, as discussed below.^309^

#### 6.2.2 Buckling Phenomena in Spherical Emulsion Droplets

Buckling in spherical emulsion droplets can arise from two main phenomena.^309^ First, the alignment of the CNCs into a cholesteric should render the structure more compressible along the helical axis: well-aligned fibers can lead to easier compression (or swelling) in the direction perpendicular to the CNC long axis,^518,519^ and also lead to anisotropic Young moduli in the dry state.^520^ For the radial alignment of the helical axis in an FP-like structure, this results in an imbalance of radial to orthoradial compressibility upon volume contraction,^515,521^ leading to an excess of surface area to maintain a spherical shape. Moreover, the compressibility of the cholesteric structure in the orthoradial directions is expected to vary with a *p*/2− periodicity along the radial direction. Consequently, orthoradial compression can lead to wrinkling of this structure, as also expected for a layered plywood structure upon in-plane compression.

Second, CNC concentration across a drying spherical droplet is nonuniform, and the concentration front propagating inward in the arrested droplet can lead to a denser and stiffer shell that resists more orthoradial compression upon further volume contraction.^522^ Buckling of spherical shells created by this mechanism have been clearly observed when drying droplets of a suspension of monodisperse spherical particles.^523^ It has been shown that in some cases buckling can be prevented by maintaining a high permeability of the solvent,^524^ which supports this explanation, and suggests that buckling can be solely driven by the kinetics of water loss.

A third proposed source of buckling is of liquid crystalline origin, in analogy with the Helfrich-Hurault instability (see section 4.3), as suggested in a recent review.^387^ In this case, the mechanism is a trade-off between long-range bend distortion and short-range twist distortion, which is qualitatively very similar to the first mechanism proposed above. However, this mechanism would occur before KA, and experimental observation of CNC emulsion droplets indicates that wrinkling only occurs after KA, so this third mechanism can be discounted in this particular case.

For both the first and second proposed mechanisms above, mass conservation implies that an insufficient orthoradial contraction should be compensated by an enhanced radial compression. This more general concept can be used to explain the pitch contraction upon buckling observed in spherical droplets of CNC suspension, regardless of the responsible underlying mechanism.^309^

##### 6.2.2.1 Pitch Scaling with Φ after Buckling

To account for buckling, regardless of its origin or underlying mechanism, the local distortion of the arrested cholesteric phase in a FP-like structure can be modeled by three successive transformations: (1) isotropic compression, as discussed above, (2) anisotropic, incompressible (i.e., volume-conserving) distortion along the helical axis, and (3) shear perpendicular to the helical axis. Applying the three transformations in this order does not require inferring that the suspension undergoes these transformations in that exact order, but it allows modeling any of the intermediate structures occurring upon drying as the result of these three transformations applied in that order. Since the third transformation does not change the pitch value, it is irrelevant for estimating the pitch scaling with the volume fraction and can be safely discarded for that purpose. Since this distortion is indicative of a system with more solid-like attributes and is especially evident in the final dry state, in the following text the buckled object will be referred to as a microparticle.

The isotropic compression followed by the unidirectional, incompressible distortion along the helical axis can be challenging to describe in a spherical geometry, since they lead to a complex buckled geometry. However, on the local scale, the deformation of the cholesteric order is similar to a uniform cholesteric structure that is compressed more along the helical axis and less in the two other directions. Such distortion is described by a compression tensor of the more general form α_b̿_, where the index ‘b’ relates to the buckled structure:
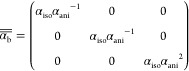
55Here, 0 < α_iso_ ≤ 1 is a scaling parameter for isotropic compression and 0 < α_ani_ ≤ 1 is a parameter describing the anisotropic incompressible distortion.

According to eq 55, the pitch *p*_b_ of a domain pointing along the **z** axis scales as
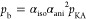
56

The condition det(α_b̿_) = α_iso_^3^ is identical to the previous case without buckling and thus leads to the same relationship between volume fraction and compression:
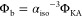
57

In a spherical droplet with FP-like cholesteric structure, the helical axis is radial and the preferential contraction along the radius implies a reduced compression in the orthoradial direction, normal to the helical axis. Eq 55 defined locally for a domain oriented along the *z* axis becomes applicable globally at the particle level only if the helical axis orientation is radial.

Adopting this hypothesis, the anisotropic distortion parameter α_ani_ can be estimated by measuring the global morphological properties of the buckled microparticles. The microparticle surface area *S*_b_ and the volume *V*_b_ can be estimated as
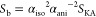
58
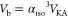
59where *S*_KA_ = 4*πR*_KA_^2^ and *V*_KA_ = 4*πR*_KA_^3^/3. The transformation of the radius of the particle requires some additional considerations. By mass conservation, the buckled structure must fit inside the same unbuckled volume *V*_p_, so that the average radius of the particle should scale as the unbuckled radius
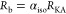
60

The cross-section of such particle would lead, from these scaling arguments, to a perimeter  and a cross-sectional area Σ_b_ given by
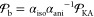
61
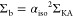
62where  and Σ_KA_ = *πR*_KA_^2^ if passing through the center of the particle. The anisotropic distortion parameter can then by estimated from the isoperimetric quotient *Q*_b_, defined as
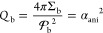
63

This scaling of *Q*_b_ with α_ani_ is valid only for a radial FP-like conformation. However, it also allows discussing some interesting limit values regardless of the geometry:

- The case α_ani_ = α_iso_ corresponds to a vertically aligned domain in the drying geometry of a flat film: *p*_b_ = α_iso_^3^*p*_KA_, with *α*_*x*_ = *α*_*y*_ = 1 and α_*z*_ = det (α̿) = α_iso_^3^. The pitch evolution is then  and no buckling is observed.
- The case α_ani_ = 1 corresponds to a FP-like aligned domain in the spherical geometry in absence of buckling, i.e., *p*_b_ = α_iso_*p*_KA_ and thus . In that case, the requirement for an initial radial alignment is no longer needed.

More possibilities are possible when considering the dependence of α_ani_ as a function of the volume fraction Φ_*b*_.

##### 6.2.2.2 Experimental Observations of Buckling

**Figure 30:**
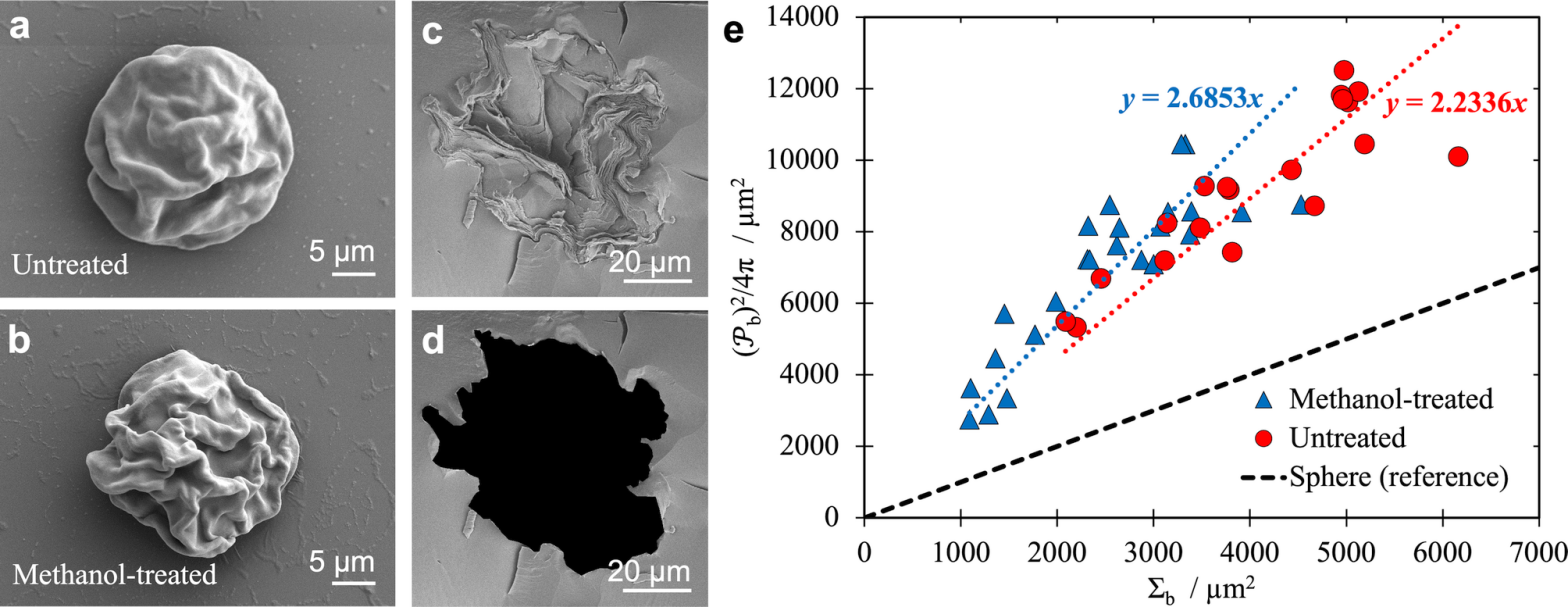
(a, b) Example of SEM images of particles before and after methanol treatment. (c) Cross-sectional SEM of a typical buckled microparticle and (d) the corresponding shaded area from which are estimated the perimeter  and the surface Σ_b_. (e) Estimation of the isoperimetric quotient *Q*_b_ of the untreated and treated particles as the inverse of the slope. Adapted with permission from ref (309) under CC-BY. Copyright 2022 The Authors.

The production of buckled microparticles via emulsion route will be described in more detail in section 9.2.3 dedicated to their optical properties, as they appear structurally colored. When these microparticles are observed in SEM, they appear buckled, yet after washing with methanol, they appear even more buckled (Figure 30a,b) and their color significantly blue-shifted. Similar buckling and blue-shifting observations were observed when the particles were heated, and TGA revealed that a significant amount of water was still present in these particles prior to such treatments.

The characterization of the cross-sectional SEM images allowed for the experimental estimation of the isoperimetric quotient *Q*_b_ of the particle cross-sections as the inverse of the slope of  in function of Σ_b_ (Figure 30c–e).

**Figure 31:**
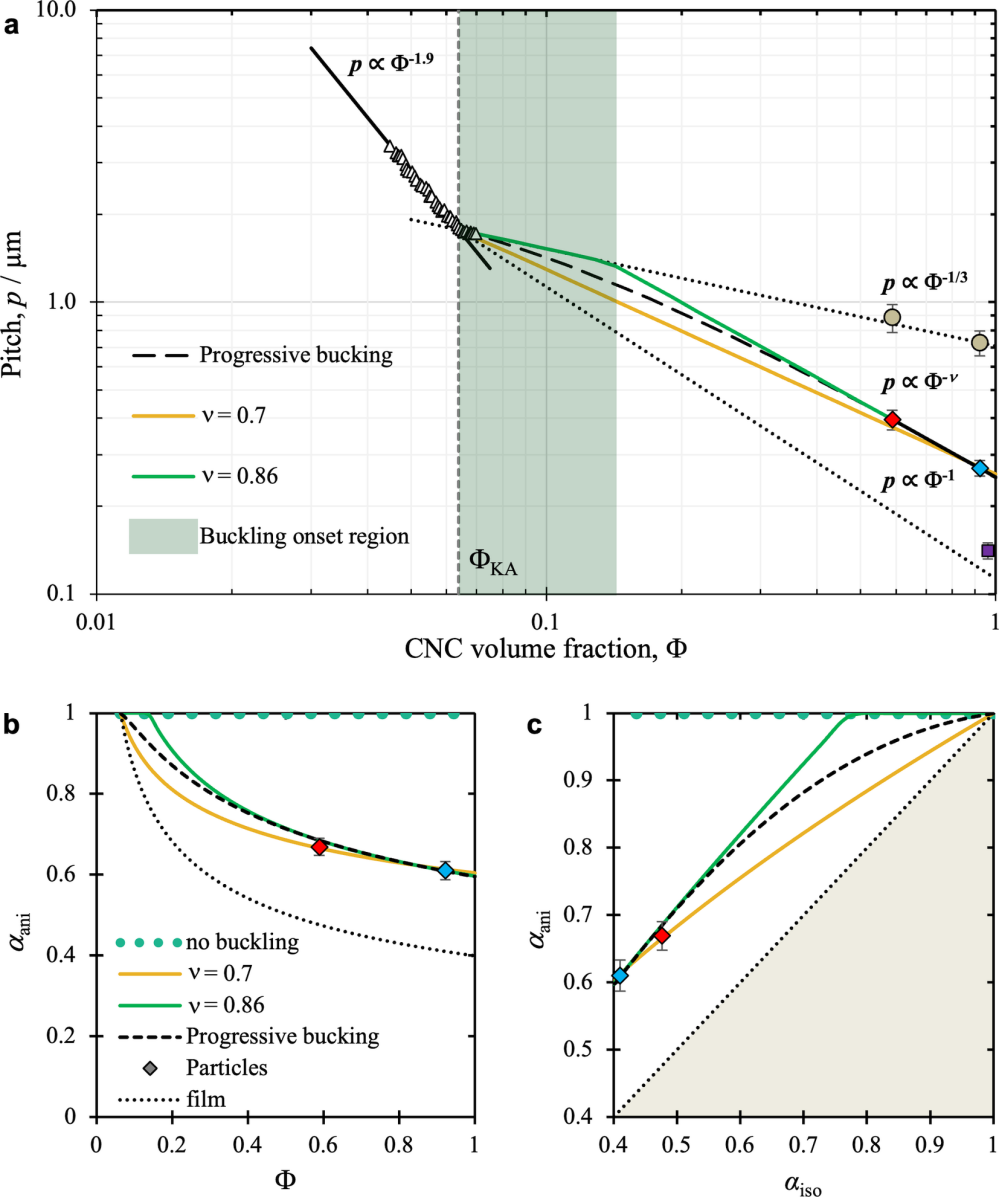
(a) Evolution of the pitch *p* with the volume fraction Φ in an emulsion droplet. Open triangles: Experimental pitch *p*(Φ) from POM, diamonds: *p*_b_ of microparticles before (red) and after methanol treatment (blue) using pitch from reflection wavelength and water content from TGA), circles: *p*_b_*Q*_b_^–1^ expected if no buckling had occurred. Various power laws are indicated with dotted lines. Φ_KA_ is indicated by a vertical dashed line, while the onset of buckling occurred within the shaded Area. (b, c) Various buckling scenarios with corresponding dependences (b) between α_ani_ and Φ and (c) between α_ani_ and α_iso_. Adapted with permission from ref (309) under CC-BY. Copyright 2022 The Authors.

The pitch evolution with respect to the volume fraction can then be revisited to include the effect of buckling (Figure 31).^309^ The point of KA is clearly visible at the point where the power law changed to follow the same *p* ∝ Φ^–1/3^. When reporting the *p*_b_ of microparticles before (red) and after methanol treatment (blue) using pitch from reflection wavelength and water content from TGA), the pitch appears clearly smaller than this scaling law, which is ascribed to buckling. Note that revisiting the data from a previous publication also gives smaller pitch than this limiting power law, as shown in Figure 29a.^288^

Since the isoperimetric quotient is expected to explain the blue-shift upon buckling, it infers that the pitch of buckled particles should depart from the *p* ∝ Φ^–1/3^ power law by this same quantity. This is indeed observed in Figure 31, where *p*_b_*Q*_b_^–1^ appears to lie exactly on the curve .

While this scaling law seems universal, it relies on the initial structure being well oriented with a radial helical axis before buckling occurs. Departure from this can cause inhomogeneous compression of the domains and thus a collection of particles of different colors. This was observed when the suspension was diluted, which led to the formation of tactoids and a skin formation that buckled less uniformly. The structure can also change with the formulation, e.g., less salt leads to less blue-shift, and with the size distribution of CNCs, which is affected by fractionation. Using the anisotropic phase leads to fully cholesteric suspension at low enough volume fraction and provides enough time for the self-organization of the FP-like structure, while using the isotropic phase causes a competition between the phase-separation and the gelation, and leads to the buckling of a thin skin with faint structural color.^309^

### 6.3 Summary

The local compression experienced upon drying can lead to buckled structures that strongly affect the resulting optical properties. In solid polydomain films, these buckling structures are usually negligible and do not pose a big issue, but for more uniform regions, such as in magnetically aligned tilted domains, the buckling becomes critical beyond a certain tilt. In a different geometry, such as in the Frank-Pryce-like structures found in emulsion droplets, the radial alignment of the helical axis is lost upon buckling of the structure, and allows the final pitch to reach sufficiently small values to cause structural color in the visible. In both cases, a description of the mechanical properties of the arrested CNC cholesteric domains upon contraction along or perpendicular to the helical axis, such as upon solvent loss, would enable better control of the self-assembly process to produce a desired final structure. Useful insight could potentially be found in different scientific communities studying analogous processes, such as the formation of geological folds.

The interplay of liquid crystal transition, kinetic arrest and geometric factors upon further solvent evaporation leads to solid films that retain some helicoidal structure, with various levels of ordering at different length scales. The interaction of light with these aligned structures can cause complex optical effects that will be discussed in the next section.

## 7 Optical Properties of Cholesteric CNC Structures

This section provides a theoretical overview of structural color mechanisms present in CNC materials, and discusses experimental methods and numerical simulations used to analyze these effects. As the local birefringence of aligned CNC phases and films is a prerequisite for many of the optical effects discussed below, this section begins with a discussion of how the macroscopic birefringence of these structures arises from the individual birefringence of CNCs. The most common mechanisms of appearance of structural color in CNC materials are then introduced by considering model systems, namely thin films, birefringent plates and ideal cholesteric structures, followed by a discussion of the optical consequences of distortion and defects often found in CNC films. Relevant experimental techniques for optical characterization of CNC films and suspensions are then presented, accompanied by practical examples and guidance for data processing. Finally, numerical tools for simulating CNC structures are discussed.

### 7.1 From Individual to Macroscopic Birefringence

The effective optical properties (i.e., refractive indices and birefringence) of structures assembled from CNCs can differ substantially from the optical properties of pure crystalline cellulose, mainly due to the elongated shape of CNCs, their imperfect alignment, and the contribution of any other materials present. This section discusses how these effective optical properties can be estimated for CNC suspensions and solid films.

Note that it is possible to assign bulk optical properties to individual CNCs as, despite their nanoscale dimensions, the particles are still much bigger than the constitutive glucose residues. Consequently it is possible to ascribe a refractive index along each of their main crystallographic directions, rather than considering their (tensorial) polarizability, as would have been expected for molecular mesogens.

#### 7.1.1 Birefringence of Perfectly Aligned CNC Suspensions and Composites

It is beneficial to first consider the effective optical properties of suspensions and composites containing perfectly aligned CNCs before considering the effects of nonideal alignment. For a system composed of inclusions of one material inside a matrix of another material, the effective optical properties result from the intrinsic optical properties of the bulk materials and the geometric properties of the inclusions (i.e., their shape and orientation). In the case of CNCs suspended in a solvent (usually water), the birefringence of the suspension can be estimated by considering the CNCs as elongated, uniaxial birefringent inclusions in a continuous medium of refractive index *n*_*s*_ (see section 2.3.4 on the possible biaxiality of crystalline cellulose). However, this description is only appropriate if the refractive index contrast is relatively low |*n*_CNC_ – *n*_*s*_| ≪ 1 and the inclusions are relative small (i.e., of diameter *d* such that 2*π d* |*n*_CNC_ – *n*_*s*_| ≪ λ), in which case the Rayleigh-Debye-Gans (RDG) approximation is valid. In general, the optical properties of inhomogeneous media are complex, and may require consideration of Mie resonances and multiple scattering. For CNCs in water, the RDG approximation is at the edge of validity.

For a composite (denoted “comp”) containing inclusions of birefringent rods with ideal parallel alignment (*S*_2_ = 1), the optical properties can be estimated using the Maxwell-Garnett effective medium theory, which is valid if the volume fraction of inclusions is low (Φ ≪ 1). In this case, the refractive indices parallel and perpendicular to the rod axis are given by^525^
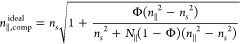
64
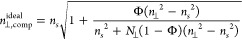
65where *N*_∥_ and *N*_⊥_ = (1 – *N*_∥_)/2 are depolarization coefficients that are dependent on the inclusion shape and aspect ratio (e.g., *N*_∥_ ≈ 1 for thin platelets,  for spheres and *N*_∥_ ≈ 0 for long rods),^526^ (*n*_∥_, *n*_⊥_) represent the intrinsic refractive indices of the rods, and Φ is their volume fraction. The resulting birefringence is then Δ*n*_comp_^ideal^ = *n*_∥, comp_^ideal^ – *n*_⊥, comp_^ideal^ and corresponds to the birefringence of the composite with ideal internal alignment, as would be probed by incident light.

In the case of spherical particles, the composite would only be birefringent if the particles themselves were intrinsically birefringent (*n*_∥_ ≠ *n*_⊥_) and their optical axes were at least partially aligned. However, an effective birefringence can also emerge for nonbirefringent (*n*_∥_ = *n*_⊥_) particles, provided they are anisometric (*N*_∥_ ≠ *N*_⊥_), aligned, and have refractive index contrast with the solvent, a phenomenon referred to as “shape birefringence” or “form birefringence”. For the more general case of both anisometric and birefringent particles, the extrinsic birefringence at perfect alignment is given by the equations above. Notably, the overall birefringence is not a simple sum of contributions from intrinsic birefringence and shape birefringence.

Finally, for birefringent inclusions in an isotropic matrix, it is not possible to simultaneously achieve index-matching for both refractive indices. However, if birefringent rods are placed in an index-matching birefringent matrix with aligned optical axes, then the refractive index *n*_*s*_ is replaced by (*n*_∥_, *n*_⊥_), and the effective birefringence of the composite is simply equal to the intrinsic birefringence of the rods, namely Δ*n*_comp_^ideal^ = *n*_∥_ – *n*_⊥_. Examples of such configuration could be a CNC within a perfectly aligned dry film, or a cellulose microfibril within a dense cellulose fiber (with perfect internal alignment and no swelling).

When the volume fraction Φ of CNCs is comparable to the volume fraction of their surrounding medium, the refractive indices (*n*_∥, comp_^ideal^, *n*_⊥, comp_^ideal^) of the composite material (assuming ideal, perfect alignment) are better described by Bruggeman’s effective medium theory:^288,528,529^
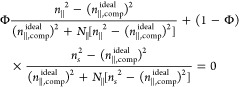
66
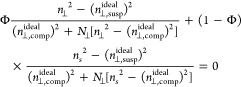
67where (*n*_∥_, *n*_⊥_) represent the intrinsic refractive indices of the CNCs. This expression, which is usually solved numerically to obtain *n*_∥, comp_^ideal^ and *n*_⊥, comp_^ideal^, is more relevant than the Maxwell-Garnett model when the volume fraction of CNCs is comparable to that of the matrix.

#### 7.1.2 Effects of Non-Ideal Alignment of CNCs and Extrinsic Birefringence

The alignment of particles within a nematic or cholesteric liquid crystal is never perfect, and this nonideal alignment reduces the effective birefringence of the resulting material. The alignment of the particles is well captured by the quadrupolar orientation order parameter *S*_2_ (introduced in section 3.1.1) and is associated with the average orientation of the long axis of the CNCs along the director **n**. For partially aligned birefringent rods in an isotropic matrix, the birefringence of the composite, Δ*n*_comp_, can be approximated to
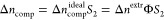
68where Δ*n*^extr^ is the extrinsic birefringence of perfectly aligned CNCs, given by the equations in section 7.1.1. This relationship can be used to estimate *S*_2_ (by measuring Δ*n*_comp_ and estimating Δ*n*_comp_^ideal^ for the inclusions). Conversely, if Δ*n*_comp_ is measured and *S*_2_ is known, then the CNC extrinsic birefringence Δ*n*^extr^ can be estimated. If the shape of the CNCs is known (providing values for the depolarization coefficients *N*_∥_ and *N*_⊥_), the extrinsic birefringence at Φ ≪ 1 can be calculated from the refractive indices of the CNC and the surrounding medium:
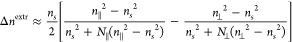
69

For infinitely high aspect ratios, the approximation of *N*_∥_ = 0 and *N*_⊥_ = 1/2 leads to an asymptotic value of Δ*n*^extr^ ≈ 0.122(5) for CNCs in water, in fair agreement with calculations assuming typical dimensions for polydisperse CNCs sourced from tunicates (Δ*n*^extr^ ≈ 0.120).^11^ Note that by convention reported refractive index values are usually measured at the sodium double D-line (around 589 nm). As refractive indices vary slightly with wavelength (as mentioned in section 2.3.4), the reported values are therefore only approximately correct for other wavelengths in the visible range.

#### 7.1.3 Local Alignment and Birefringence in CNC Films and Suspensions

In a nematic liquid crystalline phase, realistic values for *S*_2_ are expected typically between 0.55 and 0.85, and is expected to increase with the volume fraction.^308,530^ It is interesting to consider the birefringence of CNC films estimated from experimental observations as a way to estimate *S*_2_ in the suspension at KA, before the anisotropic compression affected their orientation. For that, we can assume that the volume fraction of CNCs in dried solid films is Φ ≈ 1, and that the local birefringence inside the film would only depend on the intrinsic birefringence of the individual CNCs and their relative alignment in the film, leading, in first approximation, to
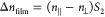
70with (*n*_∥_, *n*_⊥_) being the intrinsic refractive indices defined earlier for CNCs.

To justify this assumption, solid dried films made only from CNCs and no other additive appear to be fairly compact and can be expected to have little porosity: This porosity was estimated by swelling measurements of ultrathin CNC films made by spin-coating and showed about 21% swelling at ambient RH (ca. 40%) for thicknesses of 15 nm, but only about 4–5% swelling for slightly higher thicknesses of 26 nm.^531^ Typical photonic CNC films are about 2 orders of magnitude thicker (i.e., at least a few microns), and exposing a CNC film to a dry (0% RH) atmosphere usually leads to a small blueshift of no more than Δλ/λ ≈ 2.5%.^338^ While swelling is arguably not the best method to estimate the volume fraction of the pores in the gaps between the CNCs, an estimation of Φ ≥ 95% appears reasonable and is supported by thermogravimetric measurements.^526^

An experimental estimation of the birefringence of cholesteric CNC films from the fitting of reflection spectra gave the following values:
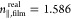
71
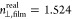
72with a birefringence Δ*n*_film_ = *n*_∥, film_^real^ – *n*_⊥, film_^real^ = 0.062.^337^ Assuming that the intrinsic CNC birefringence is *n*_∥_ – *n*_⊥_ = 0.074,^337^ this local birefringence value therefore suggests that the orientation order parameter in the film, *S*′_2_ is approximately 0.84.

The orientation order parameter of the film, *S*′_2_, is expected to be higher than *S*_2_ in suspension due to the vertical compression upon drying, which favors horizontal CNC alignment. The value of *S*_2_ before compression (i.e., at kinetic arrest) can be estimated by the following method. First, in a cholesteric structure, the variance in local mesogen orientation is related to *S*_2_ by ⟨cos^2^θ⟩ = (2*S*_2_ + 1)/3 where θ is the angle between the mesogen orientation and the local director. By cylindrical symmetry, the direction cosines in the other two orthogonal directions, **z** (vertical) and **z** × **n** (horizontal), are both equal to (1 – *S*_2_)/3. Uniaxial vertical compression by a factor *α*_*z*_ affects the **z** component of the mesogen orientation without affecting the orthogonal directions (**n** and **z** × **n**). As a consequence, the variance in mesogen orientation after compression is given by  = , and the final order parameter is given by *S*′_2_ = 3⟨cos^2^θ ′ ⟩/2–1/2. For realistic values of the compression factor (*α*_*z*_ ≈ 0.1–0.2), α_*z*_^2^ ≪ 1 and so the **z** component after compression is negligible. Applying this analysis for *S*′_2_ ≈ 0.84, an initial value of *S*_2_ ≈ 0.71 at kinetic arrest is obtained, which lies in the typical range for cholesteric liquid crystals.

In summary, the birefringence of a CNC composite (film or suspension) can be approximated by the following expressions:
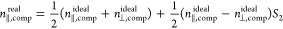
73
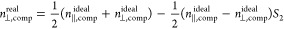
74where the values for (*n*_∥, comp_^ideal^, *n*_⊥, comp_^ideal^) were provided earlier, and the order parameter is *S*_2_ ≈ 0.7 for cholesteric suspensions (or uncompressed arrested structures, such as photopolymerized suspensions), or *S*′_2_ ≈ 0.85 for films obtained by drying.

From this analysis, the optical properties of CNC-based materials can be considered in the continuum limit, without explicitly referring to the individual CNCs for CNC suspensions, composites and films.

### 7.2 Common Structural Color Mechanisms in Non-Helicoidal Structures

Structural color can be achieved using several different mechanisms that all rely on interference, in contrast to coloration achieved using chemical compounds (e.g., dyes, pigments) with selective spectral absorption. Interference phenomena are well-described using the classical description of light as continuous electromagnetic waves: despite the widespread use of the term “photonic” to describe CNC photonic materials, a quantized (i.e., photon-based) description of light is not necessary to understand their optical response. In this classical description, interference occurs when coherent light waves that have traveled along different optical paths are combined. As interference effects depend on the geometry of the sample and the wavelength of the incident light, they often lead to angle-dependent coloration, otherwise known as iridescence.

To understand the variety of optical phenomena observed in CNC-based materials, it is beneficial to first consider light interference in simpler ideal geometries. Throughout this section, and this review more generally, the symbol λ will be used to refer to the light wavelength in vacuum. For circularly polarized light, the handedness (LCP or RCP) is defined ‘from the point of view of the receiver’, as used in many standard optics texts (e.g., ref (532)) and recommended by SPIE and IUPAC. Unless otherwise stated, materials are assumed to be dielectric and nonabsorbing (i.e., with no imaginary component to the complex refractive index), which is a good approximation for cellulose in the visible range.

#### 7.2.1 Thin Film Interference

**Figure 32:**
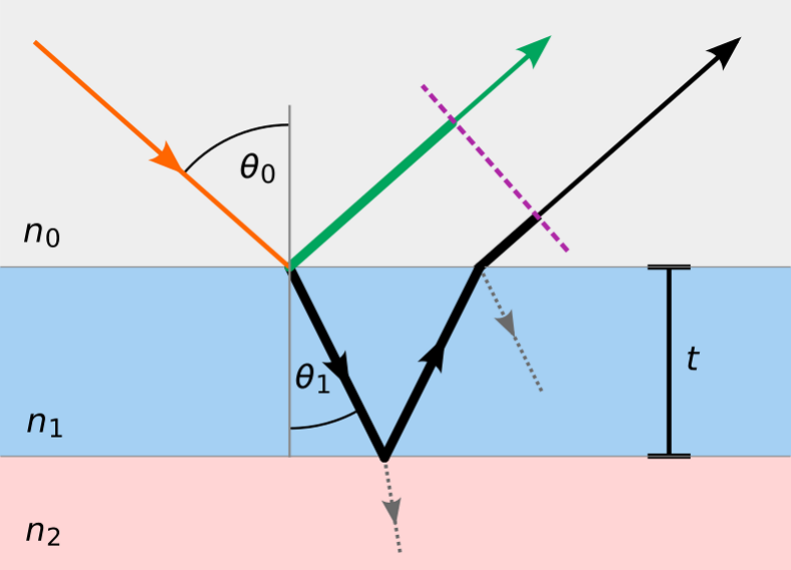
Schematic illustrating thin film interference in a system with three regions (0, 1, 2) of differing refractive index. Incoming light (orange arrow) in region 0 (refractive index *n*_0_) is incident upon a smooth interface with region 1 (the “film”, blue) at an angle θ_0_. Some light is directly reflected (green arrow), while the remainder is transmitted into region 1 at an angle θ_1_. A component of this transmitted light (black arrows) is reflected off the boundary with region 2 (the “substrate”, red) back into region 1 and is then transmitted through the 1–0 interface. The path difference between the two reflected rays in region 0 (thicker lines) leads to interference.

One of simplest geometries that gives rise to structural color is the case of a thin planar film, as illustrated in Figure 32. While thin film interference is a well-known phenomenon, it is worth briefly summarizing its main characteristics before considering more complex geometries. A more detailed introduction to interference can be found in elsewhere.^5,532^

For light incident on a transparent thin film at an angle θ_0_, the principal contributions to the reflected light come from reflection at the first (air–film) interface and reflection at the second (film–substrate) interface, as illustrated in Figure 32. Interference occurs between these two contributions as the waves have a relative phase shift dependent on their optical path difference (OPD). The OPD varies with the thickness of the film, the refractive indices of the different media, and the incident angle of the light:
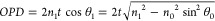
75

Here the OPD is expressed as a function of the angle θ_1_ inside the film, and also as a function of the incident angle θ_0_ in the starting medium 0 (usually air, with *n*_0_ = 1), the latter expression obtained using Snell’s law of refraction:
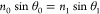
76

While the OPD is the principal contribution to the phase difference between the wave components, an additional π radians phase shift, equivalent to half a wavelength (λ/2), must be added whenever a wave is reflected from an interface with a medium of higher refractive index. Two situations can then occur. In the case when *n*_0_ < *n*_1_ < *n*_2_ (e.g., reflection from air onto a film of immiscible oil on water), constructive interferences occur for
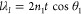
77where *l* is a positive integer. Note that this expression is mathematically equivalent to Bragg’s law, which will be introduced later. In the case when (*n*_0_, *n*_2_) < *n*_1_ (e.g., soap bubbles, a film of water with air on both sides), constructive interferences occur for:
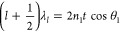
78

In both cases, the wavelengths interfering constructively in reflection also interfere destructively in transmission and vice versa.

These interferences, for both eqs 77 and 78, are often termed “thin film interferences” since they result in spectral oscillations of reflected and nonreflected light that are visible by eye (i.e., they are resolvable by human trichromatic vision). However, this effect is only visible for films of less than a few microns in thickness, as for thicker films the peaks and dips are too close to one another in wavelength. Indeed, the distance in wavelength separating two reflected peaks (Δ*λ*_*l*_ = *λ*_*l*_ – λ_*l*+1_) quickly decays with the film thickness as
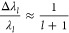
79

From eq 79, it is clear that higher orders (*l* ≫ 1) result in dense oscillations of peaks. By inserting either eq 77 or 78 into eq 79, it can be shown that for a given illumination wavelength λ ≈ *λ*_*l*_, the spectral distance Δ*λ*_*l*_ decays quickly with the film thickness, as  at normal incidence.

The discussion above considered only the principal contributions to the reflected light, and did not take into account the relative intensity of each contribution. Considering all contributions quantitatively, it can be shown that the reflectance  for a film of thickness *t* at normal incidence is given by
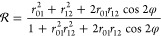
80
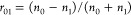
81
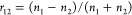
82
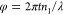
83

While interference occurs in any thin transparent films, its intensity is usually low: for a free-standing film, the maximum possible reflectance is
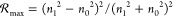
84and the resolution of the spectral peaks scales as Δ*λ*_*l*_/2. It is therefore impossible to decrease the peak width around a selected band without getting additional peaks.

**Figure 33:**
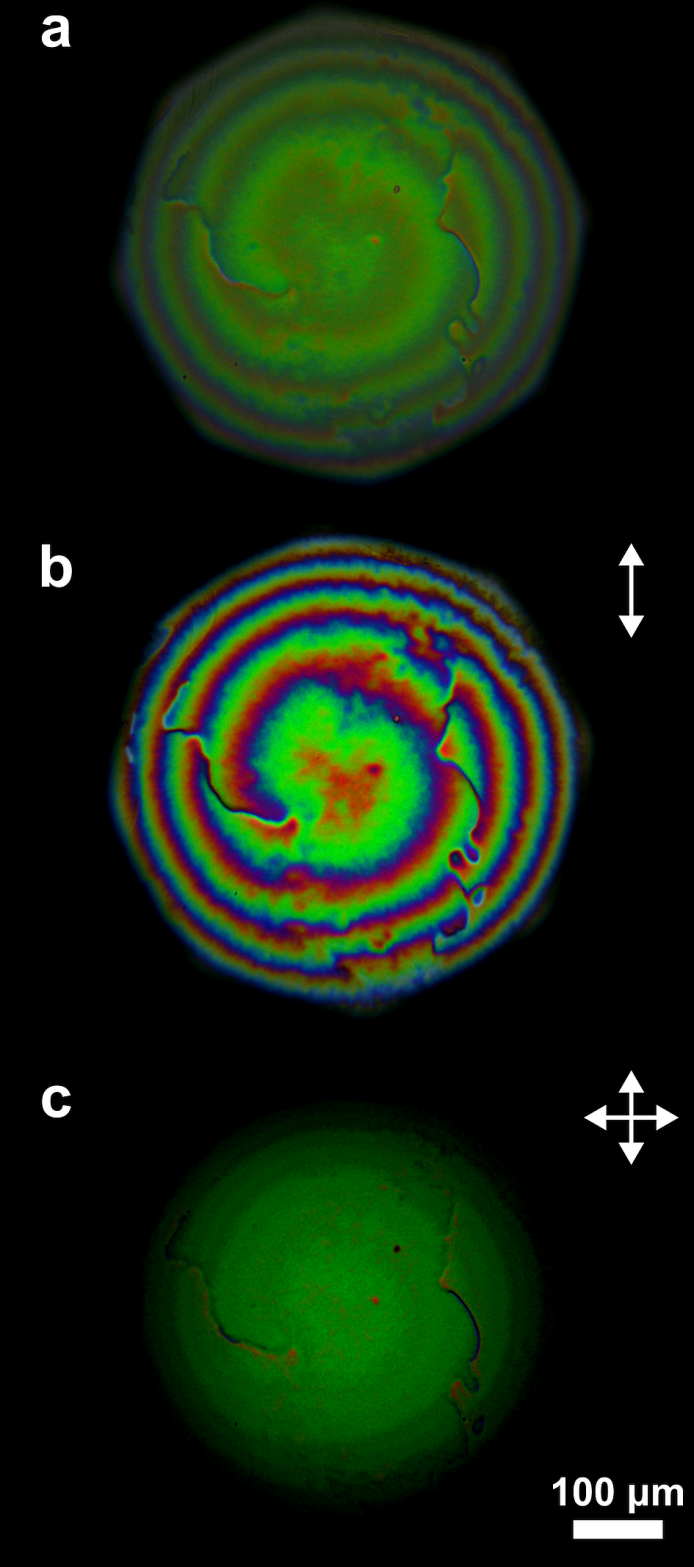
POM in reflection mode of a thin CNC photonic film also displaying thin film interference. Images acquired for (a) unpolarized light (b) between parallel polarizers and (c) between crossed polarizers. Adapted with permission from ref (338) under CC-BY. Copyright 2018 The Authors.

Thin film interference is sometimes visible in photonic CNC films, especially at the edges of the films where the thickness is not homogeneous. Interference fringes are especially apparent for thin CNC films, as shown in Figure 33.^338^ Notably, since these interferences are polarization maintaining (i.e., they do not modify the polarization state of the incoming light), the fringe contrast can be enhanced by observing the reflected light between parallel linear polarizers (Figure 33b), while the fringes are not visible for a film viewed between crossed polarizers (Figure 33c).

While thin film interference does not produce sharp and intense color, it is possible to stack thin films of alternative high and low refractive indices on top of one another as a multilayer, leading to what is sometimes called a 1D photonic crystal or a distributed Bragg reflector.^533^ The resulting effect, which can be optimized if the two alternating materials have overlapping reflected wavelengths (i.e, *n*_1_*t*_1_ = *n*_2_*t*_2_), is the appearance of several stopbands centered around *l λ*_*l*_ = 2(*n*_1_*t*_1_ + *n*_2_*t*_2_) at normal incidence, and, for the main stopband at *l* = 1, with a width Δλ scaling with the thickness of the individual layers and in first approximation with their refractive index contrast (i.e, |*n*_1_ – *n*_2_|). Inside these stopbands, the light is mostly reflected irrespective of its polarization, while outside the stopbands it is mostly transmitted (for the corresponding expressions of  see refs.^533,534^ This allows for combining strong reflection and spectral purity, otherwise impossible in thin films. As will be shown in section 7.3, the optical response of cholesteric structures share some analogies but also important differences with these systems.

#### 7.2.2 Birefringent Plate

In uniaxial anisotropic materials, the refractive indices may be denoted *n*_∥_ and *n*_⊥_ as used above. Since *n*_∥_ corresponds to one specific axis, while *n*_⊥_ corresponds to the other two axes, these refractive indices are sometimes denoted *n*_*e*_ and *n*_*o*_ respectively, with the subscripts indicating the indices along the “extraordinary” or “ordinary” optic axes. The birefringence of the material is then defined as Δ*n* = *n*_∥_ – *n*_⊥_.

For a thick birefringent plate (i.e., a birefringent film of thickness *t* ≫ λ), illumination with unpolarized light will not result in any visible interference. However, interference colors can be observed if the incident light is passed through a linear polarizer and the transmitted light exiting the birefringent plate passes through a second linear polarizer (often called an “analyzer” in the context of optical microscopy and spectroscopy). If the extraordinary optic axis lies in the plane of the plate, linearly polarized incident light can be split into two overlapping beams with different polarization states that experience different refractive indices as they travel through the plate. Due to their different optical paths, these beams can then interfere with each other after exiting the plate. The phase shift between the two beams, φ, is given by
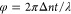
85

Note that no interference will occur if the polarization axis of the linearly polarized incident light is parallel to either the extraordinary or ordinary optic axes. The strongest interference occurs when the polarization of the incident light is at 45° with respect to the optic axes of the birefringent plate and the two beams have equal intensity. In this case, the polarization of the light exiting the plate can be expressed as an electric field vector **E**, given by
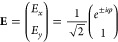
86

Here the coordinate system is defined such that the light is propagating along *z* axis, *n*_⊥_ is in the plane (*y*, *z*), and *n*_∥_ lies along the *x* axis. From eq 86, it can be seen that in general, the transmitted light is no longer linearly polarized, and birefringent plates can therefore be used to modulate light polarization (as discussed below). Note that as the wave phase is cyclic and repeats after 2π, only the value of φ modulo 2π matters. This implies that several thicknesses of identical φ modulo 2π value would be equivalent, but only when a single wavelength is considered.

If the light exiting the plate is then passed through an analyzer aligned perpendicular to the polarization axis of the initial incident light, the transmittance spectrum (proportion of light transmitted versus wavelength) is given by
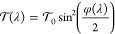
87where  is a constant that accounts for some losses in the system. From eqs 85 and 87, it is clear that a nonbirefringent plate would have zero transmittance (since φ = 0), while in general a birefringent plate has an oscillating wavelength-dependent transmission spectrum. This explains why isotropic materials appear dark in optical microscopy performed in transmission mode between crossed polarizers, whereas birefringence materials appear bright. Note that similar observations are not found for optical microscopy in reflection mode, since the nontransmitted color is absorbed by the analyzer instead of being reflected.

From eq 87, it can be shown that the wavelengths of maximum transmittance (i.e., constructive interference) are given by
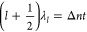
88

If instead the birefringent plate is observed between parallel (i.e., “uncrossed”) polarizers in transmission, the constructive interference condition is given by
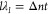
89

Notably, these expressions are similar to those obtained for thin film interference, but with the birefringence replacing the refractive index. Similarly, the spectral resolution of the interference peaks is given by . Crucially, since Δ*n* ≪ *n*, the interference peaks are visible for a much wider range of sample thicknesses. The interference colors seen in the background of POM images in misaligned cholesteric CNC suspensions (*t* ≳ 200 μm) are therefore expected from this effect (e.g., Figure 12).

A given phase difference φ corresponds to a specific transmission spectrum, and therefore color. Consequently, for an inhomogeneous sample, regions of constant φ correspond to contours of identical color, known as *isochromes*. A common and useful example of isochrome observation in the CNC literature is the so-called “fingerprint pattern” of cholesteric suspensions (see e.g. Figure 12 and Figure 15), which is often used to estimate the pitch. In this case, the periodic variation in CNC alignment can creates alternating bright and dark fringes (corresponding to the extraordinary optic axis lying in the plane of observation or out of this plane, respectively) with a periodicity of *p*/2 (i.e., half the cholesteric pitch).

As mentioned above, a birefringent material will have no interference pattern if its optic axes, or their projections in the plane of observation, are aligned with the polarization axes of the polarizer or analyzer. In this case, a birefringent material observed in transmission between crossed polarizers will appear dark. For inhomogeneous samples, this can result in patterns of identically aligned regions called *isoclines*. Isoclines can be seen in Figure 18d and Figure 19a as the dark lines separating, on these images, the square-like fields of the checkerboard pattern.

Finally, eqs 85 and 86 show how a birefringent plate can be used to convert linearly polarized light into a different polarization state. While an arbitrary phase shift φ will, in general, lead to an elliptically polarized state, specific values for φ deserve special attention:

a. If φ = ± π/2, then the OPD is Δ*n t* = λ/4 and the plate is therefore known as a *quarter-wave plate*. It can be used to convert linearly polarized light into either right-circularly polarized (RCP) or left-circularly polarized (LCP) light, and vice versa. In practice, this is how CP spectroscopy or microscopy is performed: by combining a quarter-wave plate and a linear polarizer at ±45° with respect to each other, it is possible to create an analyzer for circularly polarized light.
b. If φ = ± π, then Δ*n t* = λ/2 and the plate is called a *half-wave plate*. It can be used to convert linearly polarized light at +45° to linearly polarized light at −45°, and vice versa (so that the polarization appears have rotated by 90°, and the sample appears bright between cross polarizers). A half-wave plate can also convert RCP light into LCP light and vice versa. As a consequence, a bilayer consisting of a half-wave plate atop a left-handed helicoidal structure will reflect RCP light: incident RCP light is first converted into LCP by the half-wave plate, reflected from the helicoidal structure as LCP light, and then converted into RCP light as it passes through the half-wave plate a second time. This effect can be utilized to create multilayer structures that reflect more than 50% of incident unpolarized light, despite using only one handedness for all the cholesteric reflectors used, as illustrated in section 10.2.4 with laminated CNC films.
c. If φ = ± 2π, then Δ*n t* = λ and the plate is called a *full-wave plate*. Consequently, the plate does not affect the polarization of the light at a wavelength λ, but will modulate the polarization of light at other wavelengths that do not satisfy the full-wave condition. Consequently, a full-wave plate, also known as a *first-order lambda plate*, *tint plate* or *sensitive-tint plate*, can be used in POM to distinguish the sign of the birefringence Δ*n* in a sample. Since the full-wave plate has a retardation that is perfectly adjusted only for a specific wavelength λ, white light illumination on a full-wave plate adjusted to green wavelengths usually over- and under-compensate for shorter and longer wavelengths according to eq 87, leading to the faint transmission of both blue and red in similar amounts. A sample without birefringence will therefore appear magenta. Any additional birefringence then adds or subtracts to the phase and disturbs the blue to red balance into a cyan or yellow color, allowing for the distinction of positive and negative Δ*n*. If the sign of the birefringence is known (e.g., CNCs have positive Δ*n*), the coloration of the sample directly indicates the direction of alignment.

### 7.3 Helicoidal Structures

The optical response of helicoidal structures (i.e., with an underlying cholesteric order) can be modeled, in the first approximation, as a finite stack of thin birefringent plates with the extraordinary axis of each plate pointing along the local nematic director **n**. If the thickness of these hypothetical plates is then taken to be infinitesimally small (i.e., much less than the wavelength), the resulting optical response is mainly defined by the total thickness *t*_Ch_ of the cholesteric, the pitch *p*, and the refractive indices (*n*_∥_, *n*_⊥_). It is worth stressing that these plates, like the “pseudo-planes” sometimes used to describe the cholesteric structure, are purely a conceptual aid, and are not present in the real experimental system (as explained in detail elsewhere^18^).

Since the cholesteric structure presents a periodic modulation of its refractive index along the helical axis, it shares several similarities with a multilayer composed of alternating physical layers of isotropic materials and known as distributed Bragg reflector (see section 7.2.1). While the optical response of both structures can be described with a Bragg-like relation (see below), such superficial similarities can lead to misunderstanding of the underlying mechanism.

It is also important to note that the optical properties arise from the anisotropic structure, and not from its liquid-crystalline behavior: a solid, continuous twisted plywood structure, however it is assembled, will exhibit the same optical effects as a cholesteric phase. However, for simplicity, and to maintain validity for liquid-crystalline systems, this structure will be referred to in this section as “*cholesteric*”, regardless of its other liquid-crystalline (i.e., dynamic) properties.

To describe the optical response of helicoidal structures, it is convenient to first define the following notation for the anisotropic dielectric permittivity of a uniaxially birefringent material:
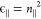
90
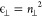
91
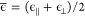
92
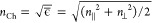
93
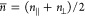
94
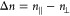
95

Note that there are two ways of averaging the refractive indices: *n*_Ch_ is the square root of the mean of the dielectric constants, while *n̅* is the mean of the refractive indices directly. In practice, these two values are very similar for cellulose (*n*_Ch_/*n̅* ≈ 1.0003).

#### 7.3.1 Reflectance at Normal Incidence

The optical response of cholesteric structures was first described analytically by Oseen, who considered illumination of a structure at normal incidence with a helical axis, **m**, normal to the interface, so that the local refractive indices vary only as a function of the depth, *z*.^535,1501^ In that case, strong reflection is observed within a wavelength window
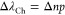
96centered around
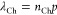
97

This reflection has several notable features. First, reflection only occurs for incident circularly polarized light of the same handedness as the cholesteric structure (i.e., LCP in the case of CNC cholesterics), while light of the opposite handedness is transmitted. Furthermore, the reflected light preserves its handedness (i.e., LCP light is reflected as LCP), which contrasts with what is observed on conventional, nondiffractive reflection on a smooth interface, where a handedness inversion occurs (i.e., LCP light is reflected as RCP). Another interesting feature is the absence of higher order reflection models (i.e., there is no reflection at wavelengths *l λ*_*l*_ = *n*_Ch_*p* for *l* > 1). Finally, since both λ and Δλ_Ch_ are proportional to *p*, the width of the reflection peak scales with peak wavelength.

The LCP reflectance (normalized by the total incident LCP light) of a left-handed cholesteric plate at normal incidence, as given by de Vries,^536^ can be rewritten using a notation adapted from Belyakov et al. as^537^
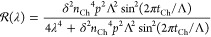
98where the local optical anisotropy of the structure is expressed as a dimensionless quantity:
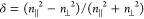
99which is related to the birefringence by
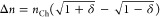
100and a local, effective wavelength Λ defined as
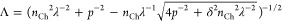
101

Here, the wavelength Λ is real outside the interval Δλ_Ch_ and imaginary inside Δλ_Ch_. Notably, this expression does not account for the mismatch of refractive indices at the air–film and at the film–substrate interfaces and requires using the associated Fresnel coefficients. Numerical tools that aid in the estimations of these contributions are discussed in section 7.5.1.

**Figure 34:**
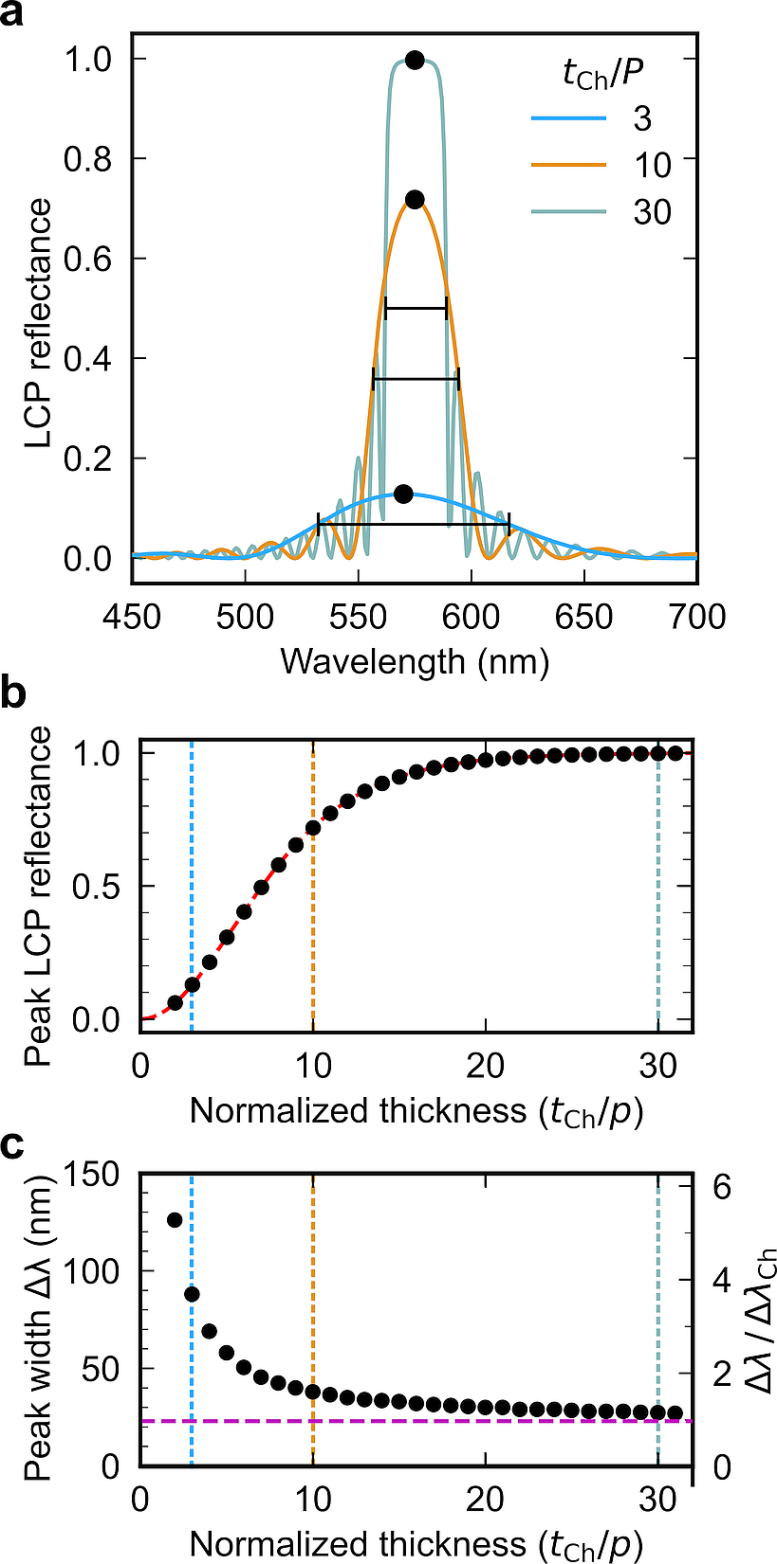
(a) LCP reflectance spectrum  at normal incidence for uniform cholesteric domains of three different normalized thicknesses, calculated using the refractive indices (*n*_∥_, *n*_⊥_) of typical CNC films assuming *p* = 370 nm. The peak maxima and the peak widths Δλ(*t*_Ch_) defined as the full-width at half-maximum (fwhm) are highlighted for all three cases. (b) Reflectance at the center of the stopband expressed in function of the cholesteric thickness, expressed as *t*_Ch_/*p*. (c) Peak width Δλ(*t*_Ch_) (left axis), also expressed as the ratio Δλ(*t*_Ch_)/Δλ_Ch_ (right axis), for a uniform cholesteric domain in function of the normalized film thickness *t*_Ch_/*p*. The dashed lines highlight the values of *t*_Ch_/*p* used in (a).

From the expressions above, the expected reflectance spectra  for a uniform cholesteric monodomain of various thicknesses with a pitch *p* = 370 nm and refractive indices (*n*_∥_, *n*_⊥_) of a typical CNC film are shown in Figure 34a (n.b. a different pitch value would only shift the position of the spectrum along the λ axis without affecting the vertical axis).

In the center of the stopband, where λ_Ch_ ≈ *n*_Ch_*p*, eq 98 yields^538^
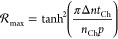
102

The dependence of  with the thickness is reported in terms of pitch repeats, *t*_Ch_/*p* in Figure 34b, and shows saturation for Δ*n t*_Ch_ ≳ *n*_Ch_*p*.

Interestingly, this expression is almost identical to that obtained for a Bragg mirror (BM), also known as a distributed Bragg reflector, when rescaling the thickness by their respective stopband widths, i.e., .^533,534^

While the stopband width Δλ_Ch_, as described from the photonic stopband analysis developed below (see section 7.3.2), is rigorously equal to Δ*np*, it corresponds to the effective reflection band only for perfect and infinite cholesteric domains, while smaller thicknesses usually lead to broader peaks. Experimentally, this was observed on well-aligned, thin photonic films made from CNCs.^337,338^ To illustrate this point, the peak width Δλ(*t*_Ch_) defined as the full-width at half-maximum (fwhm) of the reflection peak, as numerically determined from the analytical expression of , is reported in Figure 34c for increasing normalized thicknesses *t*_Ch_/*p*. It shows that even for a uniform cholesteric domain of thickness *t*_Ch_ = 10*p*, we get Δλ(*t*_Ch_)/Δλ_Ch_ ≈ 1.68, which is substantially larger than unity.

#### 7.3.2 Photonic Stopband at Normal Incidence

The section above provides the essential results for the optical response of cholesteric structures at normal incidence. However, for completeness, this supplementary section provides an explicit calculation of the photonic stopband for a cholesteric structure at normal incidence. More on the theory of the cholesteric stopband, with graphical illustrations, can be found elsewhere.^539^

The reflection from a cholesteric structure can be rewritten in the more conventional notation of wave propagation by introducing:
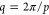
103
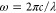
104
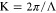
105

Here, *q* is the cholesteric wavenumber, ω stands for the angular frequency of light and *c* is the speed of light in vacuum. The quantity Κ, as it appears also in de Vries’ analysis,^536^ is the internal wavenumber (i.e., the wavenumber of the light at angular frequency ω as it propagates *inside* the material) which is defined in a spatially rotating coordinate frame. Note that unlike ω, Κ is not simply proportional to 1/λ.

The dispersion relation in terms of the internal wavenumber, ω(Κ), has four solutions given by
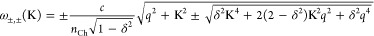
106while the inverse relation, Κ(ω), is given by
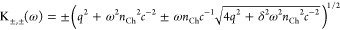
107

**Figure 35:**
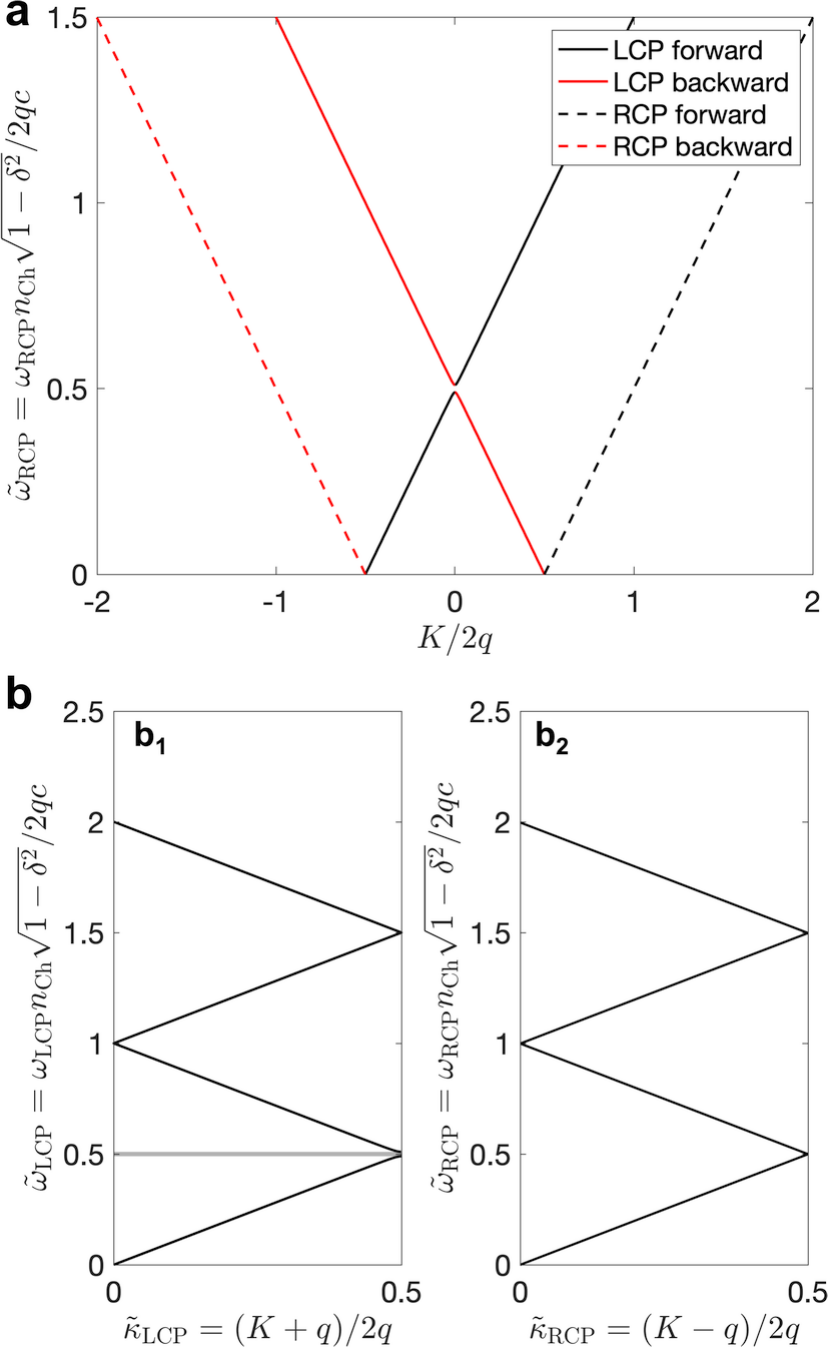
(a) Band diagram of the cholesteric structure as expressed in the formalism of de Vries.^536^ (b) Dispersion relations for (b_1_) LCP and (b_2_) for RCP waves, in the nonrotating reference frame, as expressed in the formalism of Bermel and Warner.^540^ The LCP stopband in indicated with a gray stripe.

The four solutions correspond to either LCP or RCP light, propagating in either the forward or backward direction. The dispersion relations for each case are plotted in Figure 35a.

Notably, the reflectance spectrum  obtained using this formalism corresponds to the solution *K* = *K*_+,–_:
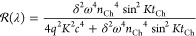
108which is equivalent to eq 98 provided above. The quantity *K*_+,–_ is only real outside the range  (i.e., for , while within this range one solution of *K* is imaginary and thus sin^2^*Kt*_Ch_ = −sinh^2^|*K*|*t*_Ch_, which leads to
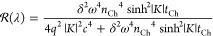
109

Dispersion relations can also be written for the stationary (nonrotating) frame by considering a coherent superposition of two waves with wavenumbers κ = *K* + *q* and κ = *K* – *q*.^540^ The selective reflection of the cholesteric structure corresponds to a gap in the dispersion relation that occurs at *K* = 0 in the de Vries analysis, which is a coherent superposition of states with κ_LCP_ = *q* and κ_RCP_ = −*q*.

As a result, the dispersion relations *ω*_*i*_(*κ*_*i*_) for *i* = (LCP, RCP) can be written as
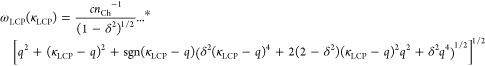
110and
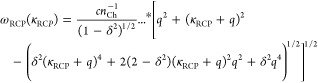
111where sgn(*x*) = *x*/|*x*|.

These dispersion relations are plotted in Figure 35b for the first Brillouin zone (κ ∈ [0, *q*]), using rescaled quantities:
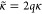
112
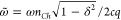
113

A stopband is clearly present in the dispersion relation for LCP light but not for RCP. From the dispersion relations *ω*_*i*_(*κ*_*i*_) above, the slope of ω̃(κ̃) is higher for LCP than for RCP, namely:
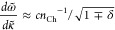
114

However, the two slopes are very similar as the local birefringence of CNC films is small (δ ≈ 0.04, see section 7.1), as seen in Figure 35b. This simplifies the calculation of the higher reflection peak wavelengths in distorted cholesterics in CNC films (see section 7.3.6).

#### 7.3.3 Optical Rotation and Circular Dichroism

As shown in sections 7.3.2, LCP and RCP light obey different dispersion relations, implying a difference in the effective refractive indices they experience within a cholesteric structure. A consequence of this effect is optical rotation, where incident linearly polarized light is transmitted as linearly polarized light with a rotated polarization axis. Optical rotatory power, expressed as an angle change per unit length, can be observed for solutions of chiral molecules that exhibit circular birefringence (*n*_*LCP*_ ≠ *n*_*RCP*_), such as monosaccharides, with values on the order of 1°/cm. In contrast, the optical rotatory power of a cholesteric structure can be several orders of magnitude larger, and is measurable at wavelengths relatively far from the stopband region. Experimentally, this explains why cholesteric suspensions with the pitch in the micron range do not appear fully dark between crossed polarizers when they are imaged along the helical axis, e.g., in flat capillaries (see section 4.2 and Figure 14).

The exact equation for the optical rotatory power is complex, and nonlinear with the thickness and other geometrical parameters.^537^ However, at normal incidence (i.e., along the helical axis), a fair approximation is given, for a right-handed cholesteric, by
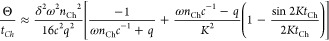
115

In the limit of large pitches (*p* ≫ λ), the angle or rotation Θ becomes linear with the thickness *t*_Ch_ of the sample and can be approximated to
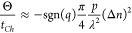
116where sgn(*q*) = *q*/|*q*| denotes the sign of the cholesteric wavevector (negative for left-handed cholesterics). This wavelength dependence of optical rotation, known as optical rotatory dispersion (ORD), can be used to determine the pitch when it is much larger than the wavelength.

The ORD of cholesteric structures implies a variation of transmittance between LCP and RCP light with the wavelength, which is usually referred to as circular dichroism (CD) and is measured as a CD extinction spectrum (see section 7.4.3). This stems from the Kramers–Kronig relationship, which demonstrates that the real and the imaginary parts of a response function (ORD and CD in this case, respectively) must respect causality.^541^ For small molecules, CD is usually a consequence of a difference in absorption of LCP and RCP light, so at first glance it is perhaps surprising that a structure made of a nonabsorbing material like cellulose should have a nonzero CD spectrum in the visible range. However, the CD observed in this case corresponds to extinction (i.e., what did not reach the detector, regardless of where it went), and the difference in LCP and RCP transmission is due to selective cholesteric reflection, rather than selective absorption.

#### 7.3.4 Reflection at Non-Normal Incidence

An ideal left-handed cholesteric structure reflects 100% of the incident LCP light within the stopband wavelength range when illuminated at normal incidence. However, at non-normal (oblique) incidence, where θ_*i*_^ext^ ≠ 0, reflection stopbands at higher orders are observed, and the reflected light is elliptically polarized, rather than being purely circularly polarized. The photonic response can then be described in terms of the *s*- and *p*- polarization components (instead of their RCP and LCP), which are defined as the linearly polarized components pointing perpendicular and parallel to the scattering plane (**k**_*i*_,**m**), where **k**_*i*_ is the incident wavevector and **m** the helical axis.^542^ For certain angles of incidence, there is a narrow wavelength range for which a stopband exists for both polarization states, depending on the azimuthal angle between the incident beam **k**_*i*_ and the nematic director at the interface **n**(*z* = 0).^537,543^ This range is sometimes referred to as a “photonic band gap” (PBG), leading to 100% reflection of incident unpolarized light. The reflectance spectrum for these structures can be expressed analytically, although the expressions are complex. Alternatively, numerical tools are available to compute with reflection and transmission spectra in each polarization state to high precision, as further described in section 7.6.

**Figure 36:**
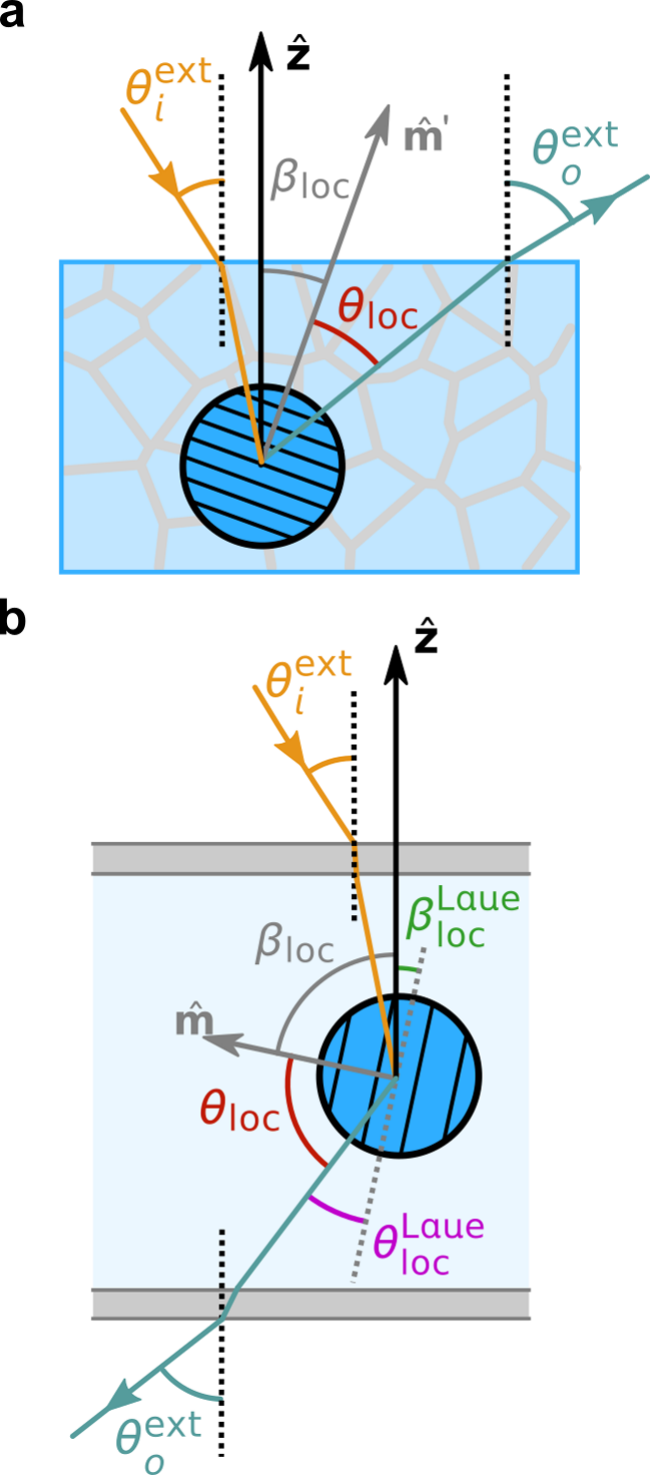
(a) Schematic of reflection from a polydomain CNC photonic film (not to scale). Incident light (orange) enters the film (blue) at an angle θ_*i*_^ext^ relative to the film surface normal (**z**) and, after refraction, is partially reflected from a tilted cholesteric domain with tilt angle β_loc_ (gray). The specular reflection from the tilted domain occurs at an angle θ_loc_ (red) relative to the domain tilt axis **m**. Outgoing light exits the film at an angle θ_*o*_^ext^. (b) Laue diffraction from highly tilted domains for a CNC suspension in a glass capillary. The angles are defined as in (a), with the Laue diffraction angles θ_loc_^Laue^ (magenta) and β_loc_^Laue^(green) also shown.

For a cholesteric structure with relatively weak birefringence (*Δn*/*n*_Ch_ ≪ 1) illuminated at oblique incidence, the wavelength of peak reflection can be obtained from Bragg’s law for a *p*/2 periodicity of the refractive index modulation:
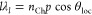
117where *λ*_*l*_ is the wavelength of peak reflection at order *l* = 1, 2, 3... Here, θ_loc_ is the local angle of incidence of light as defined inside the material with respect to the helical axis (as shown in Figure 36a). The widely quoted Bragg-like relation for photonic CNC films, namely λ = *n*_Ch_*p* cos θ_loc_, therefore corresponds to the first-order reflection peak. This relation is occasionally expressed in terms of the angle θ′_loc_ = π/2 – θ_loc_, in which case λ = *n*_Ch_*p* sin θ′_loc_. These expressions are widely reported in the CNC literature, although the angles θ_loc_, θ′_loc_ (which are defined locally inside the helicoidal structure) are often confused with the user-defined angle of incidence θ_*i*_^ext^ outside the structure. For an aligned helical axis **m** parallel to the surface normal (i.e., perpendicular to the planar interface between the cholesteric structure and air), the expression above can be rewritten explicitly in terms of θ_*i*_^ext^, using Snell’s law, as historically derived by Fergason:^544^
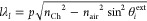
118As the cholesteric structure reflects incident light in a specular (mirror-like) manner, the outgoing angle is θ_*o*_^ext^ = θ_*i*_^ext^. More generally, if **m** is tilted by an angle β_loc_ relative to the surface normal (but the reflected light exits the sample on the same side as the incident light) it can be shown that the local angle of incidence θ_loc_ is given by
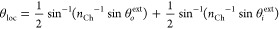
119while the local domain tilt angle β_loc_ is given by
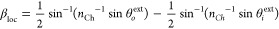
120The definitions of these angles are illustrated in Figure 36a. In this case, local specular reflection from the cholesteric domain leads to θ_*o*_^ext^ ≠ θ_*i*_^ext^. Importantly, θ_*i*_^ext^ and θ_*o*_^ext^ can take both positive and negative values, and the convention adopted here is to assume that θ_*i*_^ext^ and θ_*o*_^ext^ have the same sign when on opposite sides of the surface normal (e.g., in Figure 36a, both angles are positive).

At non-normal incidence, cholesteric domains also have an effective linear birefringence, as the *s*- and *p*-polarized waves experience different effective refractive indices:
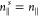
121
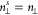
122
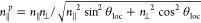
123
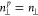
124

Consequently, any non-normal incidence with respect to **m** should result in a phase retardance between the waves transmitted through a sample, which will cause partial conversion of transmitted RCP into LCP (and vice versa).

If the CNC photonic film contains no absorbing species, the light transmitted by the sample is simply the complement of the light reflected, since what is not reflected must be transmitted. If an aligned cholesteric monodomain (β_loc_ = 0) is illuminated with unpolarized white light, all of the RCP light is transmitted, as well as the LCP light at all wavelengths except those within the stopband. For the more realistic case of a polydomain CNC film, however, both a broadening of the reflection band and a partial reflection of RCP light are observed, with the corresponding consequences on the transmitted light.

#### 7.3.5 Diffraction of Highly Tilted Domains

For highly tilted helical axes, where **m** is almost in the plane of the sample interface, the ’reflected’ light comes out of the sample from the opposite side of the incident beam. As illustrated in Figure 35b, this extremely oblique reflection leads to diffraction in the transmission direction, an effect known as Laue diffraction. The peak wavelength of diffraction in this geometry is given by^545^
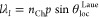
125
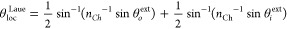
126
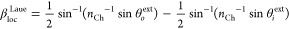
127where the complementary angle θ_loc_^Laue^ = π/2 – θ_loc_ has been defined. For highly tilted domains, θ_loc_^Laue^ ≪ 1, and so using eq 125, it can be shown that a micron-scale pitch is required to obtain *λ*_*l*_ values in the visible range. This condition can be satisfied for cholesteric CNC suspensions, and consequently laser diffraction can be used to determine the pitch of cholesteric suspensions in glass capillaries if highly tilted domains are present, as shown later in Figure 52.^249^

In the particular case of an incident beam at normal incidence to the surface (θ_*i*_^ext^ = 0) and at grazing incidence with respect to the helical axis, the light modulation simplifies to^249^
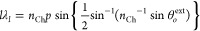
128which for small diffraction angles (θ_*i*_^ext^ ≈ 0) becomes a grating equation that does not depend on *n*_Ch_:^414^
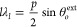
129

As a final comment, at grazing incidence (**k**_*i*_ · **m** ≈ 0), *p*-polarized light experiences a constant refractive index and only the *s*-polarized light experiences a spatially modulated refractive index:
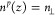
130
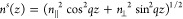
131

#### 7.3.6 Distorted Cholesterics

Geometric distortion of a helicoidal structure can have several effects on its optical response. It is instructive to first consider the simple cases of a uniaxial compression or stretching applied parallel or perpendicular to the helical axis. If the cholesteric order is only stretched or compressed along the helical axis **m**, only the pitch is altered and the helical modulation of the director alignment remains sinusoidal. In this case, the optical reflectance of the distorted structure is simply shifted in wavelength proportional to the change in pitch. Alternatively, if geometric compression or stretching is applied perpendicular to **m**, the helical axis direction and pitch remains the same, but the modulation of **n** along **m** is no longer sinusoidal, causing important modifications in the optical properties of the new structure, as detailed below. A shear applied perpendicular to **m** will have a similar effect. In the general case of geometric distortion at some angle to the helical axis, the helical axis **m** will be rotated, the pitch *p* will change, and the modulation of **n** will become nonsinusoidal, as described in detail in sections 6.1 and 6.2.

When such distortion occurs, higher reflection bands occur even at normal incidence with respect to the helical axis **m**. This was originally described by Bermel and Warner for strained cholesteric elastomers,^540^ and can be understood from a Fourier analysis of the helical modulation of **n**(**r**).^416,546^ For reasonably small birefringence values like in CNC films, the stopbands are spaced in wavelength λ as *l λ*_*l*_ = *n*_Ch_*p*, and some exist in both polarizations, leading to a total band gap in the direction parallel to **m**. The distortion of the helical order also means that **n**(**r**) is, on average, pointing more in the stretching direction than in the compressed one, and thus also partially acts as a retardation plate even along **m**, with the potential to convert RCP light into LCP, and vice versa.

#### 7.3.7 Defects between Cholesteric Domains

Defects in the cholesteric order can be of various types and geometry, which can be challenging to classify. Broadly speaking, defects are deviations of the director field **n** from a perfect, regular cholesteric order of defined helical axis **m** and pitch *p*. This can occur as smooth or abrupt variation of the orientation of the helical axis, as variation of pitch, or as disclination lines where the local nematic order is not defined. Due to conflicting continuity constraints at the boundary between regions of different **m** orientation and energy minimization criteria (elastic energy, anchoring strength, disclination lines), long-range variations usually come at the cost of local topological defects and disclination lines, all of which will in turn alter the propagation of light.

A structure made from locally discontinuous regions of different helical alignment can be considered as a polydomain cholesteric structure, each domain having a well-defined helical axis.^416^ Alternatively, the disclination lines running through a uniformly aligned cholesteric structure confined to a thin film can also be considered a polydomain structure, each domain having a well-defined pitch.^337^ The meaning given to “domains” is then very different. If we adopt the former definition for domains, the boundaries between two domains placed above one another, with a misalignment of an angle γ, they will also create periodic disclination lines that will have their own optical signature, beyond the optical signature of the domains themselves.^416^ In this regard, this is very similar to the stripes observed in Grandjean-Cano wedges,^377,378^ where the stripe periodicity Δ is given by the pitch *p* and the angle γ of the wedge, as illustrated in Figure 27:
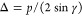
132

This periodicity has been frequently confused across the literature with the actual pitch *p* of the structure responsible for the observed reflected colors, leading to inconsistent conclusions trying to reconcile micron range periodicities with reflections in the visible. When the domains are thin enough, the mismatch at the junction between the two cholesteric regions can give rise to specific optical effects, like constructive or destructive interferences with peak splitting or shifting, as shown in thin CNC films with localized tilted domains sandwiched between cholesteric regions of planar alignment.^345^

#### 7.3.8 Interfaces, Curvature, and Polarization Effects

To conclude section 7.3, it is worth considering other effects relevant for light propagation in CNC-based materials.

First, the reflection at non-normal incidence usually results in different reflected and transmitted light intensities for the *s*- and *p*-polarized light (see section 7.3.4). These intensities are given by the Fresnel coefficients and are determined by the angle of incidence and the refractive indices on both sides of the interface. This effect is particularly useful when CNC are embedded in other transparent media. In the case of CNC flakes embedded in poly(dimethylsiloxane) (PDMS),^547^ nonpolarized light penetrating inside the transparent PDMS is expected to be partially *p*-polarized when reaching the CNC flakes. Moreover, the local variation in orientation of the flakes can modify the local angle of incidence. In practice, specular reflection dominates off-specular contributions. Consequently, the optical response of small, randomly aligned CNC flakes in a matrix is best described by Fergason’s law of a polydomain cholesteric assuming a uniform pitch *p*(0) (unlike regular CNC films where *p*(β) varies with the domain tilt β).^547^

The hard limit of the incident angle of light when defined *inside* the material then limits the blue-shift at high angles expected from the unmodified Bragg’s law, resulting in almost noniridescent coating.^547^ A normal incidence, Snell’s law puts a hard limit to the incident angle of light when defined *inside* the material. A grazing incident beam from the outside of the material (θ_*i*_^ext^ = 90°) propagates inside the material at an incident angle θ_*i*_^int^ = sin^–1^(1/*n*_PDMS_). For PDMS with *n*_PDMS_ = 1.43, this means θ_*i*_^int^ ≈ 44°, which results in a blue-shift limited in off-specular conditions to at most % instead of ≈ −29% (θ_*i*_^ext^ = 0°, θ_*o*_^ext^ = 90° or vice versa) and in specular conditions to % instead of −100% (i.e., λ → 0 for θ_*i*_^ext^ = θ_*o*_^ext^ → 90°). This same limit applies to CNC films, where the limit *n*_CNC_ ≈ 1.555 leads to θ_*i*_^int^ = sin^–1^(1/*n*_CNC_) ≈ 40°, and is already accounted for in the Fergason’s law.

Since light is both reflected and transmitted at interfaces between two regions of different refractive indices, frequent local variations of refractive index in a sample generates a large amount of scattering events that can be detrimental to the overall optical effect expected from the photonic structure. Small (i.e., 10–200 μm in diameter) photonic CNCs particles can have a rough surface (e.g., due to buckling), which can lead to substantial scattering at the particle-air interface which obscures the desired structural color.^309^ Such surface scattering can be minimized by immersing the particles in index-matching liquids, which are commercially available in well calibrated range, but such liquids are often expensive and are often sold in small amounts. For CNC-based materials, a good compromise is offered by ethyl cinnamate, a commercially available oil that is reasonably index-matching CNC-based materials (*n* ≈ 1.56) as well as being relatively inexpensive and edible.^309^

The curvature of the sample can locally modify the angles at which incident light reaches the photonic structures. This was first illustrated for cholesteric shells using molecular liquid crystals (sometimes polymerized),^548−550^ but then also qualitatively observed on CNC films locally adopting spherical curvature.^551^ The periodic modulation of the curvature, experimentally imposed to achieve a macroscopic flat film, can then also interfere with light and also act as a grating.^552^ While low curvature (∼1 mm^–1^) means the sample is locally flat and its optical properties can be understood as being locally tilted,^553^ high curvatures (∼1 μm^–1^) means the light reflected off the surface can be locally focused like a curved mirror in reflection, or like a lens in transmission. While applications based on this effect will be mentioned in section 10.3.2, curvature effects can also pose problems to correctly reference the absolute intensity measured experimentally to access the true reflectance, or transmittance, of the sample. This is due to the fact that a reference sample is usually used to calibrate the reflected intensity (usually a flat silver mirror for smooth reflecting surfaces), and that upon illumination with a cone of light (defined by the numerical aperture NA used on the optical microscope setting), the cone of light collected from a highly curved surface differs sensibly from a flat surface. Several examples of this issue were encountered in other systems with high curvatures.^554,555^

### 7.4 Characterization of Photonic CNC Films

The optical response of CNC films can be accessed by a range of experimental characterization techniques. In this section, most common optical methods are introduced with guidance for the data collection, processing and analysis required to converge toward conclusive interpretation.

These optical techniques are presented more or less in order from macroscopic to microscopic, while those specific to the characterization of CNC suspensions will be discussed in section 7.5. The section concludes with a discussion of SEM, as this nonoptical technique is a highly valuable tool for characterizing photonic structures.

To illustrate the comparison of optical characterization techniques, this section includes new experimental data acquired for a free-standing CNC photonic film (hereafter referred to as “the example film”). Details on the experimental methods are provided in section S3 of the Supporting Information.

#### 7.4.1 General Considerations for Optical Characterization

Consistent optical characterization of structurally colored materials is challenging due to their angle- and polarization-dependent optical response. The visual appearance of the sample is therefore sensitive to how the sample is illuminated and how the light that has interacted with the sample is then collected. Some important considerations relevant for many optical characterization methods are discussed below.

##### 7.4.1.1 Angular Dependence

Structural color phenomena are based on interference and are angular dependent. This means that the incident angle of the light and the outgoing angle from whoch light is collected will greatly influence the spectral composition. While techniques like angle-resolved optical spectroscopy are specifically designed to control these two angles (see section 7.4.4), others usually assume implicitly that the sample is oriented in the default orientation, namely that it is oriented normal to the incident light beam, and observed along the illumination axis, in reflection or transmission. Depending on the technique, some effects can be more problematic than others. Effects such as mild curvature can be negligible under POM but cause difficulties in angle-resolved optical spectroscopy or even photography. Surface roughness, which can be uneven and differ between the top and the bottom interface of the film, can cause additional light scattering either on its way in the sample or on its way out. Sample illumination at an angle causes different proportion of *s*- and *p*-polarizations to enter the sample, and will complicate the analysis of the polarization dependence of the sample optical response, especially its CD or ORD. While films attached to a substrate offers an excellent flatness that can be advantageous over free-standing films (see Sample Background below), they also affect the measured optical response, while free-standing films reduces the risk of artifacts arising from reflections between the sample and the substrate. When the surface roughness is highly scattering (e.g., imaging of buckled particles, see section 9.2.3), the use of an index matching liquid allows eliminating the scattering and gain control over the local incident and outgoing angles at which the light penetrates the photonic structure.

##### 7.4.1.2 Polarization

Throughout this section, the control of the polarization of the incident light and the analysis of different reflected or transmitted polarizations can be crucial to characterize the optical response across many different optical techniques, from photography and POM to angle-resolved optical spectroscopy or integrating sphere measurements. Polarizers are inserted in the optical light path between the light source and the sample to select a desired polarization state for the incident light, while analyzers are placed between the sample and the detector to select a desired polarization state for the collected light.

One of the most commonly used polarization settings for optical characterization of CNC films and suspensions is “crossed polarizers” (XP), where the incident light is passed through a linear polarizer and the collected light (either transmitted or reflected from the sample) is analyzed by passing it through a second linear polarizer perpendicular to the first. Similarly, “parallel polarizers” (PP), also known as “uncrossed polarizers”, is achieved by using parallel linear polarizers as the polarizer and analyzer. To produce incident LCP or an RCP light, the polarizer is made of two optical elements: first a linear polarizer and then a quarter-waveplate oriented with its main axes at ±45° from the linear polarizer axis. To analyze LCP or RCP light, these optical elements are used in the reverse order, namely a quarter-waveplate and then a linear polarizer. A common example of LCP and RCP analyzers can be found in modern (i.e., not anaglyphic) 3D glasses.

##### 7.4.1.3 Sample Background

Since structural color phenomena are based on selective light redistribution rather than selective light absorption, the redistributed light can interact with the immediate sample surrounding and ultimately influence the overall optical response of the sample when imaged in its environment. The role of the background thus has to be considered when taking photographs and microscope images, measuring spectra, etc. To minimize the impact of the surroundings on the optical response of the sample, it is preferable to observe free-standing samples, with a dark background placed at sufficient distance away from the sample to avoid any reflection from the substrate itself. In such configuration, the transmitted light is absorbed in the background, and the reflected light coming only from the sample can be well measured and characterized. Alternatively, if a dark and matt (i.e., nonshiny) background is placed directly underneath the sample, most of the transmitted light will be absorbed; for this reason, most structurally colored tissues in nature are found on top of dark, light-absorbing substrates.

Greater contrast can also be achieved if light-absorbing species are present within the sample itself. In this case, forward-scattered light within the sample is evanescently absorbed before it can be transmitted to the surroundings. For CNC films, this can be achieved either by the addition of absorbing species (e.g., carbon black, graphene) as contrast-enhancing agents into the initial formulation of the suspension, or by postprocessing of the film (e.g., by thermally induced darkening, as discussed in section 10.2.1). However, excessive absorption can also diminish the selective reflection of the photonic structure.

**Figure 37:**
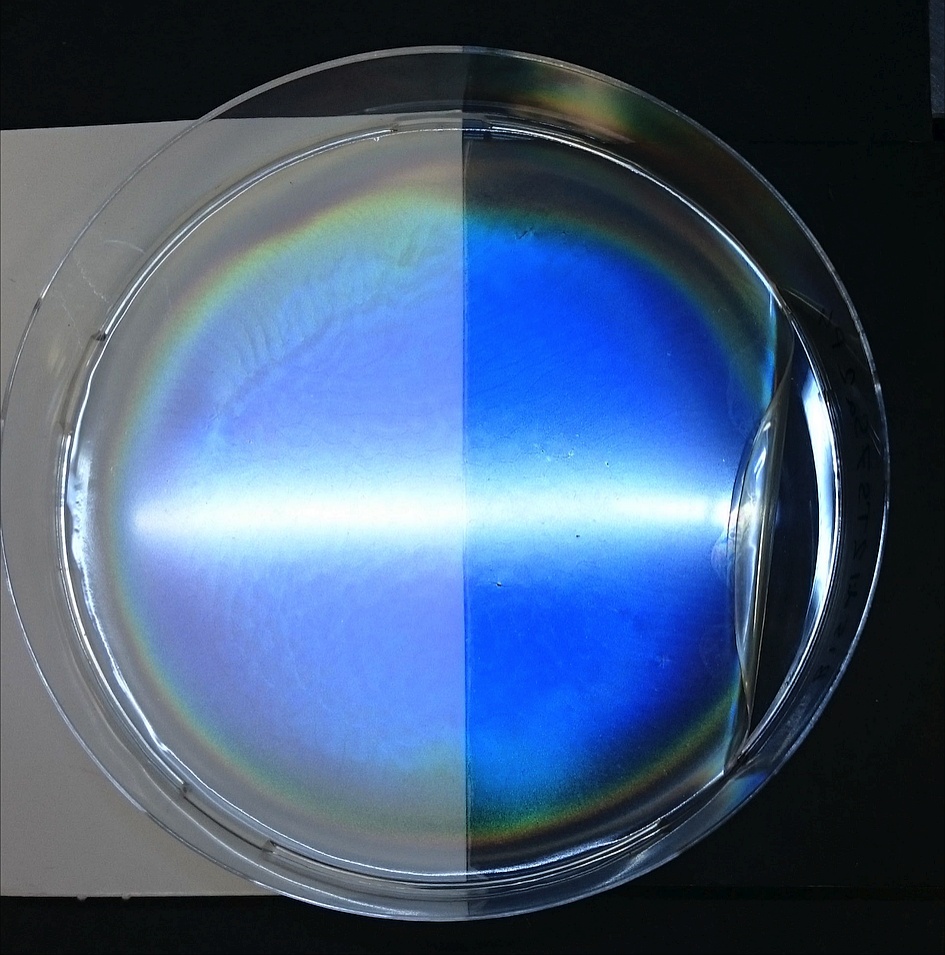
Photograph of a CNC photonic film in a Petri dish on a dark substrate. On the left side of the dish, a thick sheet of white paper is interposed between the dish and substrate. Scale bar is 2 cm.

Experimentally, samples are often mounted on transparent substrates such as glass microscope slides or plastic Petri dishes to keep the sample flat, as this is beneficial for many characterization techniques for which the illuminations and observation angles should be precisely controlled. However, transparent backgrounds introduce an offset in the reflection across the visible range and can influence the polarization of the light reflected at oblique (non-normal) incidence. Alternatively, white scattering backgrounds do not absorb the transmitted light but send it back in similar direction as the optical response, which results in a much weaker saturation of the observed color, as illustrated in Figure 37.

#### 7.4.2 Photography

**Figure 38:**
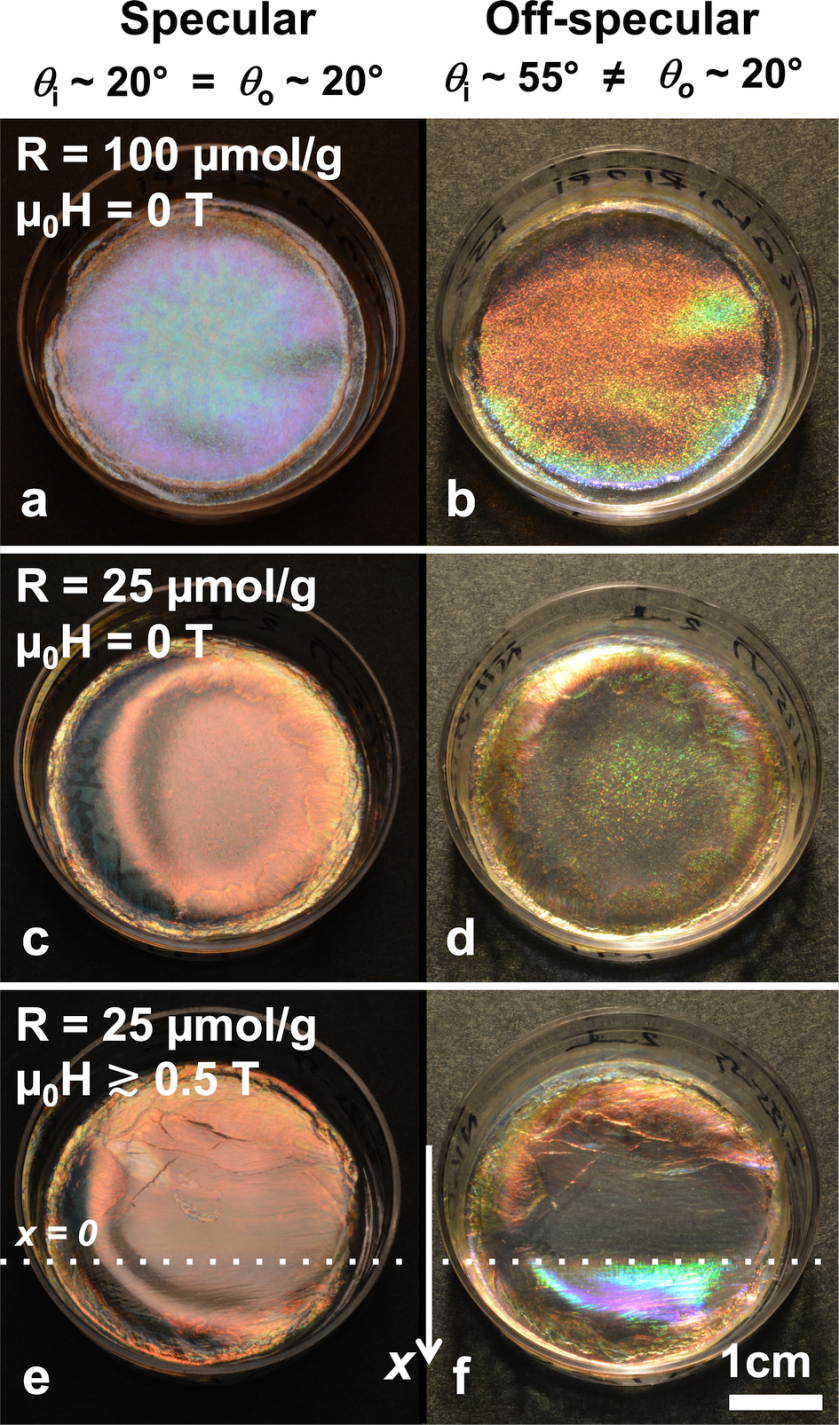
Macroscopic photographs of CNC films in specular (a, c, e) and off-specular (b, d, f) conditions. (a, b) film appearing blue in specular conditions and red in off-specular conditions, displaying the unusual iridescent behavior of CNC films. (c, d) Red to near-IR film showing faint second order green in off-specular conditions. (e, f) red to near-IR film cast in the presence of a reversing magnetic field along the x direction (turning horizontal at the dotted line *x* = 0). The off-specular conditions reveal strong second and third orders. Adapted with permission from ref (416). Copyright 2019 American Physical Society. Original data from ref (161).

It is challenging to accurately capture the visual appearance of CNC photonic films by macroscopic photography. While films usually appear colored under ambient illumination, the iridescence of the structure makes the appearance vary with both the illumination and the observation angles. If the samples have nontrivial optical response, the observation of the same sample from the same observation angle can give very different optical properties, as illustrated in Figure 38.

For a well-aligned helicoidal CNC film, the photonic response is most visible when the sample is illuminated at a fixed angle and observed at the specular reflection angle (Figure 38a,c,e). Alternative observation conditions (e.g., off-specular reflection, Figure 38b,d,f) or illumination conditions (e.g., diffuse, nondirectional illumination) will result in a different visual appearance. The sample can also be observed in transmission by illuminating through the sample, but this results in an apparent film color complementary to the reflection peak: for instance, a “blue” CNC photonic film (i.e., a film with a reflection peak in the blue wavelength range) will appear yellowish in transmission, as the transmitted light contains RCP at all wavelengths but only the nonblue LCP.

#### 7.4.3 Optical Spectroscopy

Optical spectroscopy is widely used to characterize the appearance of photonic materials by quantifying the proportion of the incident light that is reflected or transmitted at a range of wavelengths. This section provides an overview of common approaches to optical spectroscopy using fixed-angle instruments. A discussion of angle-resolved optical spectroscopy is reserved for section 7.4.4.

##### 7.4.3.1 UV–vis and CD Spectroscopy

Optical spectroscopy is often performed by illuminating the sample at normal incidence and measuring the proportion of incident light that is directly transmitted without being absorbed or scattered (i.e., the transmittance). This measurement can be performed on commercial UV–vis spectrophotometers, which are widely available in chemistry laboratories.

The loss of transmitted light, known as attenuation or extinction of light depending on the context, arises from a combination of absorption within the sample and scattering of light at angles outside the narrow range of angles collected by the detector. For incident light of intensity *I*_*i*_(λ) and transmitted intensity *I*_*t*_(λ), the transmittance is simply the ratio , while the extinction is often expressed as
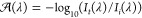
133

UV–vis transmission spectroscopy is usually performed on solutions of small molecules in a cuvette. In this case, the extinction is almost entirely due to absorption and  is therefore known as the absorbance spectrum. In contrast, the extinction of visible light in colloidal suspensions of nonabsorbing particles such as CNCs is predominantly due to scattering (turbidity) and absorption is negligible.

**Figure 39:**
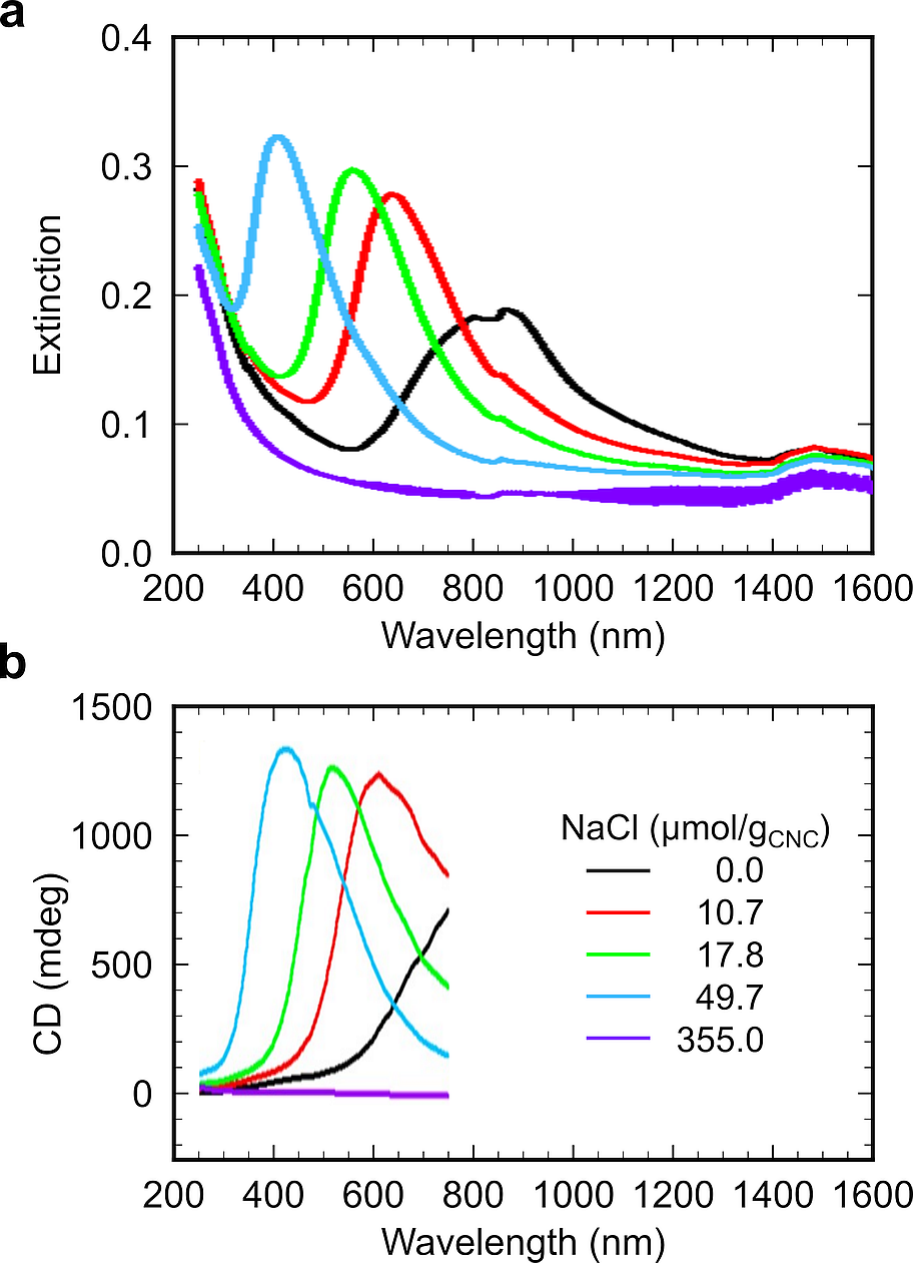
(a) UV–vis extinction spectra and (b) corresponding circular dichroism (CD) spectra of CNC films prepared with increasing proportion of NaCl in the cast CNC suspensions. Note the extended range of wavelengths compared to most optical techniques (the CD spectra can also extend to this range if necessary). Adapted with permission from ref (556). Copyright 2015 American Chemical Society.

Extinction spectra for a range of CNC photonic films are shown in Figure 39a.^556^ For well-aligned CNC films, the main extinction peak corresponds to the selective reflection of the helicoidal structure. However, the observed peak width is often much greater than that expected for a infinitely thick cholesteric monodomain (Δ*λ*_*Ch*_/*λ*_*Ch*_ = Δ*n*/*n*_*Ch*_ ≈ 0.04, using the results in section 7.3.1). As a quantitative example, such a monodomain, with a pitch *p* = 370 nm and the optical properties of a CNC film, is expected to have a stopband in the range 575 nm ±11 nm (i.e, *λ*_*Ch*_ = 575 nm, Δ*λ*_*Ch*_ = 23 nm). While the spectrum shown in red in Figure 39a roughly corresponds to this pitch value in terms of peak position (*λ*_*Ch*_ = *n*_*Ch*_*p*), the experimental peak width Δλ (defined as the full width at half-maximum, fwhm) is over 150 nm (Δλ/*λ*_*Ch*_ > 0.25, or Δλ/Δ*λ*_*Ch*_ > 6.5). As discussed in section 7.3.2, a broadening is expected for films of finite thicknesses, but this effect alone cannot explain a broadening of this magnitude (see section 5.4.3 for its possible origins). The resulting effect is however beneficial for the human eye, for mainly two reasons, related to human biology. The human eye does not easily discriminate the wavelengths that are centered on the sensitivity of each cone cell responsible for seeing blue, green, and red colors. For that reason, broader reflection peaks mean more light received from the sample and thus stronger color, without necessarily a big sacrifice on the color sharpness. Moreover, the broader peaks make it easier to identify small variation of pitch, in applications where the pitch variation is used for colorimetric sensing. For narrow peaks, the cone cells of the human eye (or RGB cameras) are only sensitive to changes in wavelength in the blue-to-green and green-to-red transition ranges, and are otherwise fairly insensitive. This phenomenon is well explained in a recent study involving very narrow reflection peaks in a non-CNC multilayer system.^557^

Scattering from imperfections in the sample leads to an additional background extinction that is independent of the position of the main extinction peak and decreases strongly with wavelength. As the illumination area is on the scale of cm^2^, the spectra are averaged macroscopically over a large number of helicoidal domains (in contrast to other methods discussed below). It is important to note that peaks in the extinction spectrum  correspond to dips in the transmittance spectrum  and vice versa. As a consequence, the extinction spectrum will resemble the reflectance spectrum (discussed below).

The polarized optical response of CNC photonic films can be quantified by circular dichroism (CD) spectroscopy. CD is the differential extinction of LCP and RCP light by a sample (i.e., the difference in how much incident LCP light is absorbed or scattered, compared to RCP light), given by
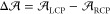
134

As with (unpolarized) UV–vis spectroscopy, CD spectroscopy can be performed using a commercially available instrument that is often found in chemistry laboratories, and the illumination area is also on the scale of cm^2^. Such instruments are typically used to measure CD spectra for solutions of chiral molecules, where the CD signal arises from differential absorption of LCP and RCP light. The CD signal is typically expressed in terms of the ellipticity of the transmitted light, which is quantified by the ellipticity angle, θ, given in degrees (deg) by
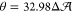


For solutions of small chiral molecules, the CD signal is often small () and typical values of θ are on the order of 10^1^–10^3^ mdeg.

In the case of CNC photonic films, the selective reflection of LCP light in the photonic stopband leads to a greater extinction of LCP light in this wavelength range, resulting in a positive CD peak, as shown in Figure 39b. For an ideal helicoidal monodomain, which reflects all LCP light and transmits all RCP light in the photonic stopband, the CD signal could theoretically approach θ_max_ = 45° (i.e., 45000 mdeg), but realistic samples have CD signals on the order of 10^2^–10^4^ mdeg, depending on the domain size and degree of ordering in the sample. Note that the presence of a CD peak for CNC photonic does not reflect an intrinsic circular dichroism of the sample, but rather an effective, extrinsic circular dichroism due to differential reflection.

As a final comment, UV–vis and CD spectroscopy collect the residual transmitted light from the sample after any reflection and scattering has occurred. This is a significant drawback when characterizing CNC photonic materials, whose visual appearance is usually based on reflection.

##### 7.4.3.2 Integrated Transmission and Reflection Spectroscopy

**Figure 40:**
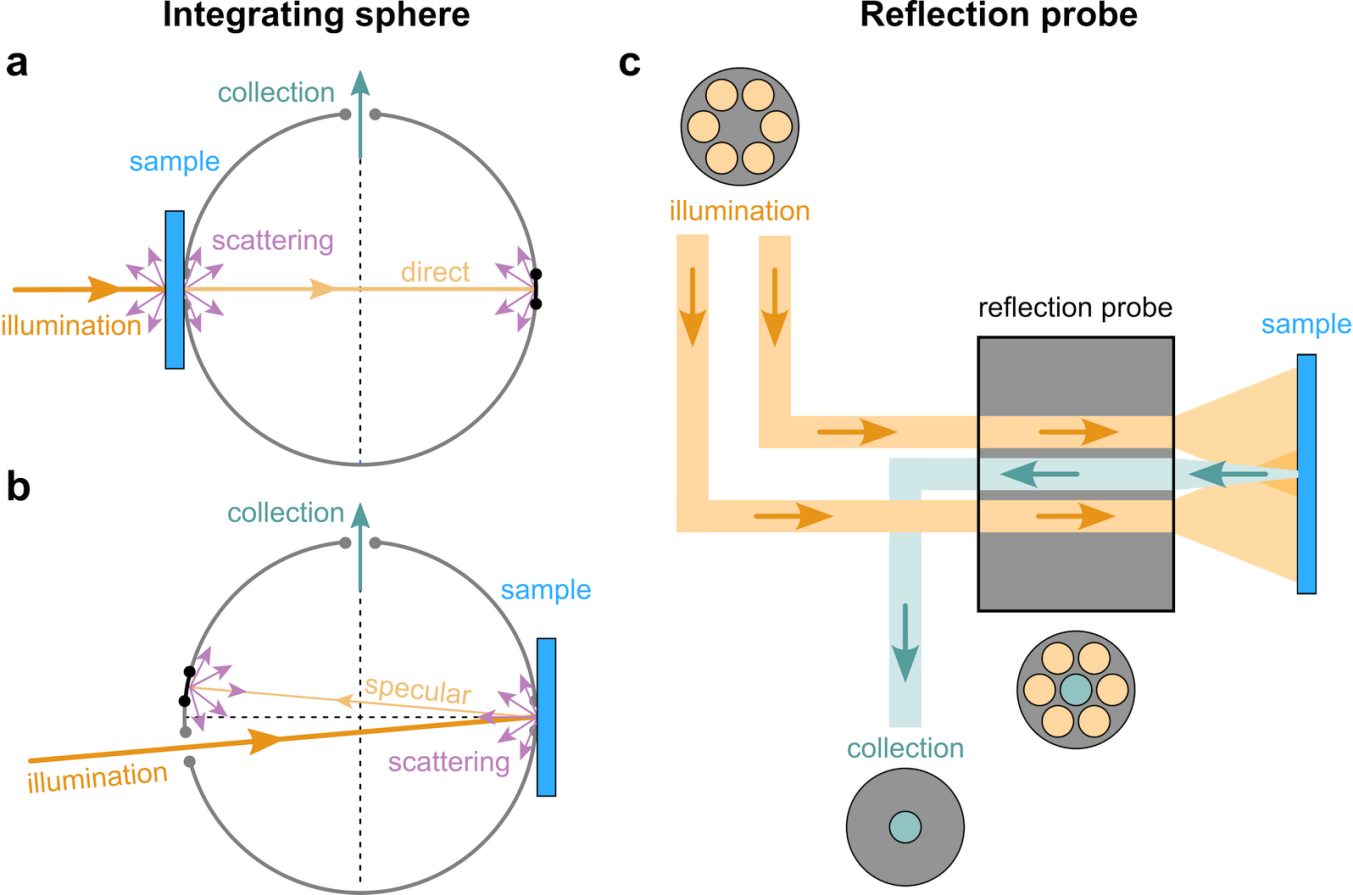
Schematic of experiment setup for (a) integrated transmission and (b) integrated reflection spectroscopy. Black lines indicate the positions of optional ports, shown closed here, as discussed in the main text. (c) Schematic of spectroscopy using a reflection probe. The sample is illuminated with a bundle of six fibers (orange) using the reflection probe (gray, not to scale). Reflected light is collected by a seventh central fiber and relayed to a spectrometer. Gray circles represent the fiber bundle cross-section at each end of the probe.

Transmission optical spectroscopy can also be performed by collecting the total transmission from the sample over all forward scattering angles (θ_*o*_^ext^ ∈ [0,90°]), a measurement known as integrated transmittance or diffuse transmittance spectroscopy. In a typical measurement, the forward-scattered light is collected by placing an integrating sphere (a spherical cavity with an inner coating of white, diffusely scattering material) immediately after the sample in the beam path, as shown in Figure 40a. A fiber-optic cable coupled into the sphere perpendicular to the beam path is then used to collect the integrated transmission and relay it to a spectrometer.

Integrating spheres are sometimes included in commercial UV–vis spectrophotometers, but also exist as standalone setups. Various settings for illumination, sample positioning and referencing can be used, and the reader is encouraged to refer to the specialized literature for this technique.^558^ For example, one alternative integrated transmission measurement uses essentially the same setup as shown in Figure 40a, but includes the opening of a port on the opposite side of the sphere to the sample (shown in black in Figure 40a), which allows directly transmitted light to exit the sphere. In this case, the integrated spectra includes diffuse forward-scattered light but without direct transmission. Note also that the illumination of the sample is not necessarily at normal incidence: if θ_*i*_^ext^ = 0°, as shown in Figure 40a, the integrating sphere setting is termed “0°/d”, but other configurations may be employed.

An integrating sphere can also be used to characterize the reflection and backscattering from the sample (θ_*o*_^ext^ ∈ [ – 90°,0]). A typical configuration for integrated reflectance measurements is shown in Figure 40b. A sample mounted on the opposite side of the sphere to the incident beam is illuminated at a low angle of incidence (typically θ_*i*_^ext^ ≈ 5°) and the diffuse reflection is collected. The direct (specular) reflection is often included in the integrated reflectance spectrum, but may be removed by opening an optional port (shown in black in Figure 40b).

**Figure 41:**
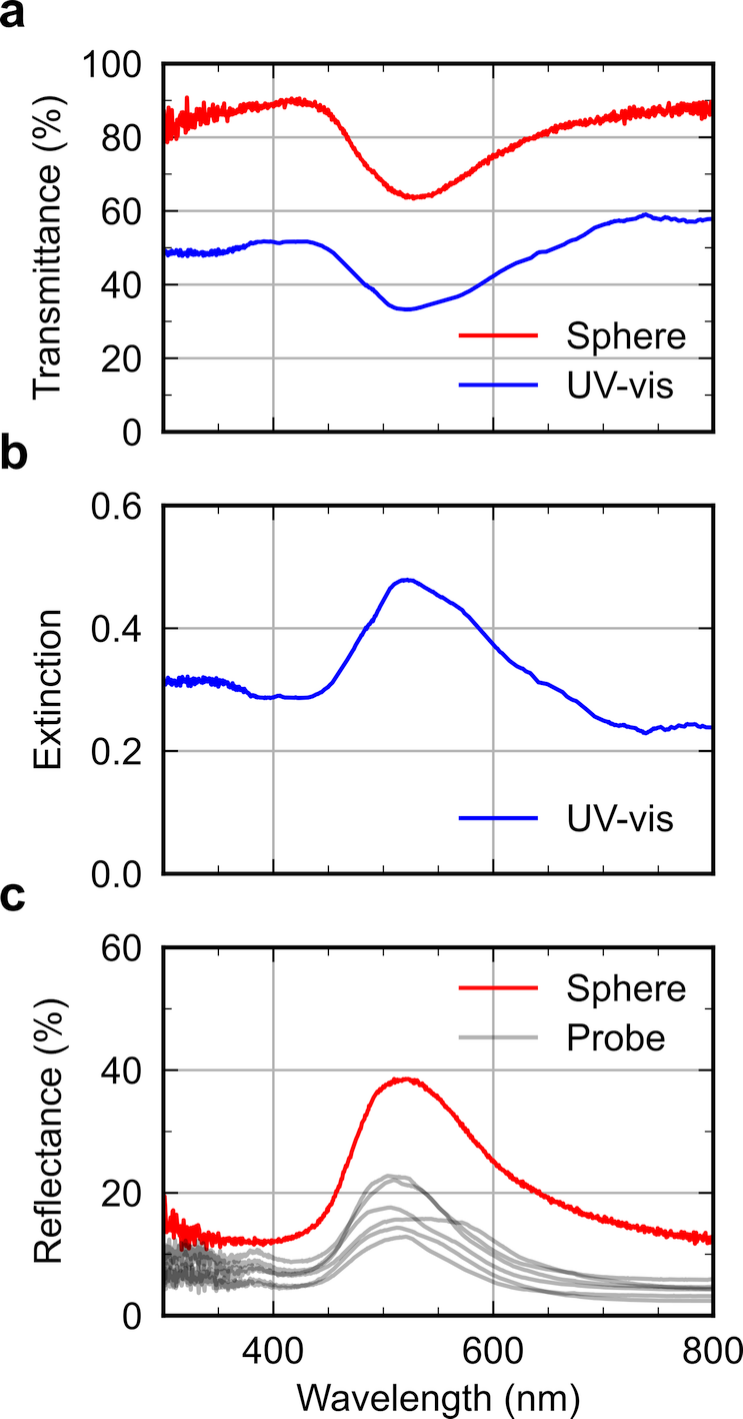
Optical spectroscopy of the example film. Transmission spectra plotted in terms of (a) transmittance and (b) extinction, reflection spectra plotted in terms of (c) reflectance. Transmission spectra were obtained using UV–vis transmission spectroscopy (blue) or an integrating sphere in transmission mode (red). Extinction is plotted for the UV–vis data only. Reflection spectra were obtained using a reflection probe (gray) or an integrating sphere in reflection mode (red). For the reflection probe, several spectra are shown to illustrate the variation between measurements; for other spectroscopic methods, no significant variation in the spectra was observed.

Integrated transmission and reflection spectra for the example sample are shown in Figure 41. Due to the integrated (averaged) nature of this measurement, the resulting spectra can be challenging to interpret. For instance, the sample affects the reflectivity of the sphere itself. Moreover, the role of the cutoff angles for light collection is easily overlooked (e.g., if the specular reflection escapes the sphere or is collected in the spectra), which for CNC films can strongly affect the position of the reflection peak.

##### 7.4.3.3 Reflection Probe Spectroscopy

A reflection or backscattering probe consists of an optical fiber bundle, with one end held in close proximity to the sample while the other end splits into six optical fibers connected to a light source and a seventh optical fiber connected to a spectrometer, as shown in Figure 40c. This “double-ended probe” enables the collection of reflection spectra at almost normal incidence on a large surface area, which is useful for averaging reflection properties on inhomogeneous samples.^559^ However, the reflected intensity is strongly dependent on the distance between the probe and the sample, and this distance must therefore be constant to ensure consistency. Structurally colored samples such as CNC photonic films, which exhibit high variability in their angular response, can make it challenging to convert the observed reflection spectra into properly referenced reflectance.^161^

##### 7.4.3.4 Comparison of Spectroscopy Methods

The spectroscopic techniques described above were applied to the example sample, producing the reflection and transmission spectra shown in Figure 41. Note that CD spectroscopy of the example film was also attempted, but the very large CD signal (>2000 mdeg) saturated the available equipment (Chirascan Q100, Applied Photophysics).

As shown in Figure 41, all these spectroscopic techniques are relevant to locate the position of the stopband of the cholesteric film. The difference between them is mostly due to the inhomogeneity of the sample, as different types of disorder will affect the spectra differently, depending on the technique. First, the baseline in UV–vis transmittance spectra due to scattering can affect the reading of the position of the photonic bandgap, especially at shorter wavelengths (a better example for that is visible in Figure 39b). Since the UV–vis is not chiral-selective, locating the deep assumes that the sample has a cholesteric order, but does not prove it if taken in isolation. A CD spectrum provides a better proof of the chiral nature of a film (which can be desirable in some cases), and a flat baseline across the scanned wavelength region, but also has drawbacks. It requires a more elaborate equipment, it can present saturation issues on some equipment, and the recorded CD can interfere with existing linear birefringence in the sample, making the collected CD signal vary with the azimuthal orientation of the sample in the spectrometer. Tilted domains, if they are present in the film, will redirect some of the incident beam back in reflection since they display larger pitch values (see sections 6.1.2 and 7.4.4), and thus they will subtract a significant amount of the incident light at larger wavelengths. The collection from an integrated sphere will capture all this reflected light, while it will be missing in the direct beam collected in the UV–vis. Thus, to a first approximation, they should then be equivalent and consistent with each other, as also suggested by Figure 39. Due to the large sample region over which the spectra are collected, these techniques are well adapted to compare samples, but they do not provide a good information about their appearance in reflection, which is ultimately the primary configuration where CNC films are employed. The reflection (backscattering) probe (and even more so the microspectroscopy performed under POM, discussed in section 7.4.5), allow for the collection of the local response of particular regions of the film, and offer a complementary information that fluctuates with the probed position of the sample. While this is desirable to capture the optical response of individual domains, it is also more variable and less characteristic of a given macroscopic sample, so that averaging over many positions is sometimes required. The geometry employed in the techniques above (apart from microspectroscopy, see section 7.4.5) is such that either all or none of the scattered (i.e., off-specular) light is expected to be measured, which in practice involves some cutoff values in the collection cone that can be slightly different, and cause more or less inconsistencies depending on the level of disorder in the sample, its surface roughness, etc. In this context, the measurement of a reflection (or transmission) spectrum at various illumination and collection angles allows for overcoming these limitations and give more insights into the optical properties of the samples.

#### 7.4.4 Angle-Resolved Optical Spectroscopy

The angle-dependent visual appearance of structurally colored materials such as CNC photonic films makes it useful to resolve their optical response not only in terms of wavelength (i.e., spectroscopy), but also in terms of the angles of illumination and observation. While this technique has been used to characterize a variety of materials, from inorganic optical coatings^560^ and gyroid metamaterials^561^ to biological photonic structures,^559,562,563^ angle-resolved optical spectroscopy of CNC photonic films has only begun to be investigated in the past decade,^416,564,565^ most likely due to limited availability of this equipment in most laboratories studying CNCs and the higher level of technical expertise required to perform accurate measurements. The unparalleled level of detail afforded by this technique makes it a powerful tool for understanding the local microstructure of CNC photonic films. In the following section, the methodology of the technique is first described, followed by interpretation guidelines and finally some experimental examples.

##### 7.4.4.1 Experimental Setup

**Figure 42:**
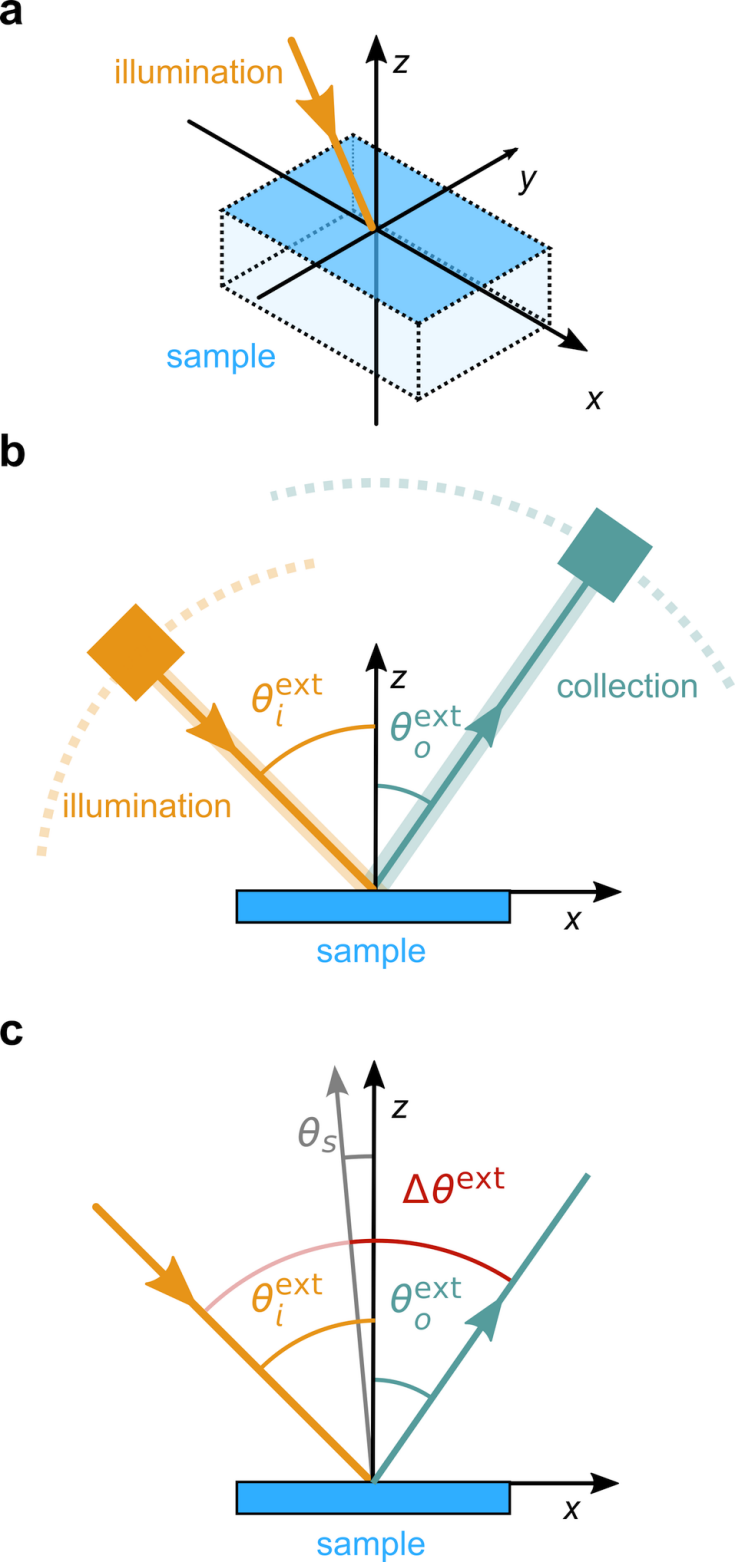
Schematics for angle-resolved optical spectroscopy. (a) Axonometric 3D projection, showing illumination (orange) of the sample (blue). The coordinate system is chosen so that the sample is fixed and the plane of incidence is the (**x**, **z**) plane, where **z** is the sample surface normal. (b) For a given angle of incidence θ_*i*_^ext^ chosen by the user, outgoing light can be collected at any angle θ_*o*_^ext^ (green) and relayed to a spectrometer. Dotted lines indicate rotation of the illumination and collection directions relative to the sample. Note that unlike θ_*i*_^ext^, the angle θ_*o*_^ext^ is defined as increasing with clockwise rotation from the **z** axis: the combination (θ_*i*_^ext^, θ_*o*_^ext^) = (45°, 35°) is shown as an example. (c) Same as (b), with the additional angles θ_s_ (gray) and Δθ^ext^ (red) indicated. In this example, (θ_*s*_, Δθ^ext^) = (5°, 40°).

A typical experimental setup for angle-resolved optical spectroscopy is shown in Figure 42. The sample is placed on a rotating stage and illuminated with collimated white light at a given angle of incidence, θ_*i*_^ext^. The spot size projected onto the sample is controlled by the optics used to develop the illuminating beam, which is usually composed by a fiber coupled light source collimated with a set of lens and pinholes. The incident light interacts with the sample, resulting in light propagating away from the sample at all angles. For a flat sample, the outgoing light is primarily confined to the plane defined by the incident light direction and the surface normal. Consequently, the optical response can be characterized by collecting the light leaving the sample at a range of outgoing angles θ_*o*_^ext^ in the plane of incidence, and then relaying the collected light to a spectrophotometer.

The precise rotations required for angle-resolved optical spectroscopy can be performed using an experimental setup known as a goniometer.^559^ As with photography or optical microscopy, the polarization state of the incident and collected light could also be modulated using linear polarizers and retardation plates. A further refinement is to perform Mueller matrix ellipsometry of CNC photonic films,^565^ which provides complete information on the Stokes vector (polarization state) of the collected light, enabling estimation of the anisotropic optical properties, e.g., linear and circular birefringence.

##### 7.4.4.2 Measurement Modes

**Figure 43:**
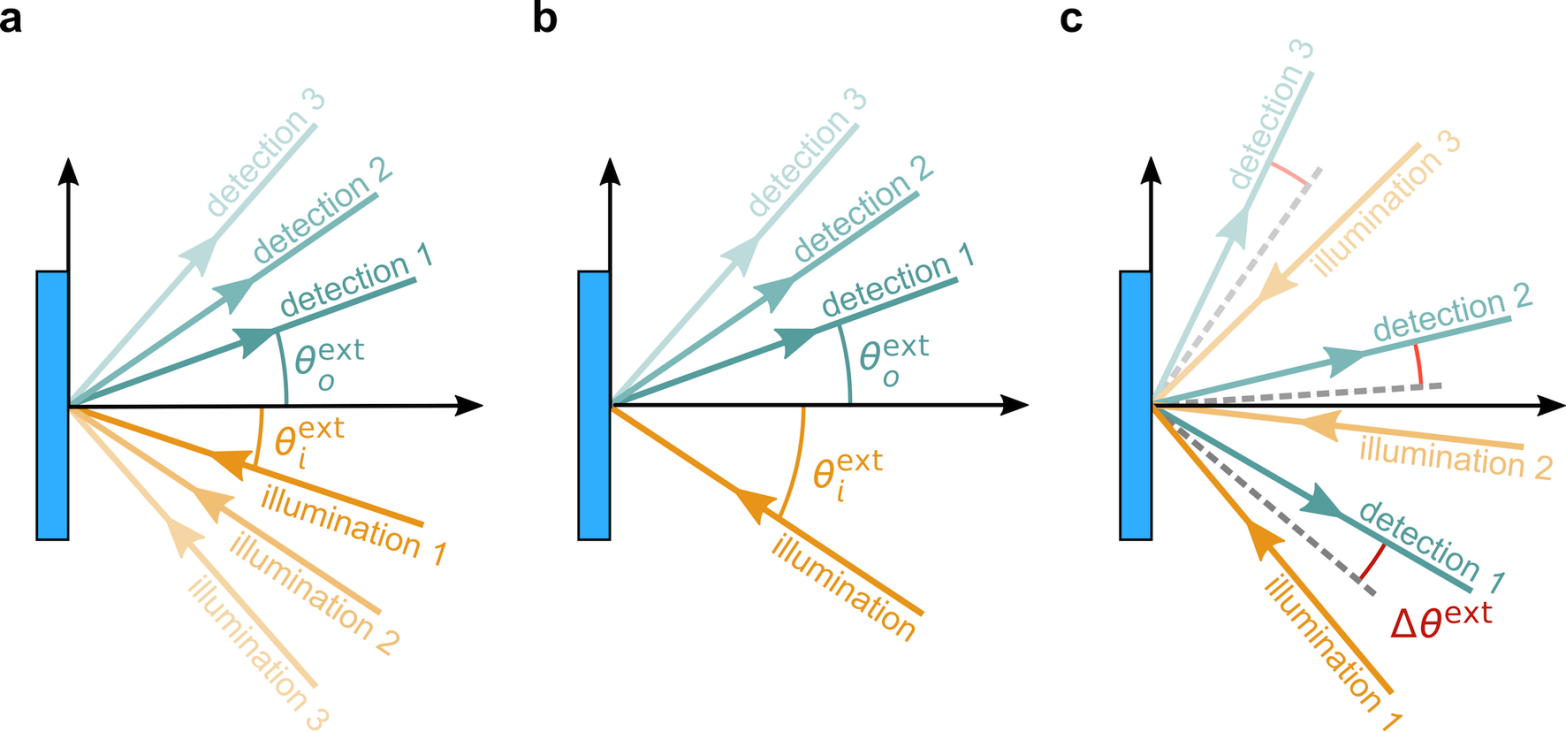
Measurement modes for angle-resolved optical spectroscopy: (a) specular scan (θ_*i*_^ext^ = θ_*o*_^ext^) (b) scattering scan (θ_*i*_^ext^ constant) and (c) a tilt scan (Δθ^ext^ constant). Adapted with permission from ref (566) under CC-BY. Copyright 2022 The Authors.

Depending on the range of detection angles chosen, angle-resolved optical spectroscopy can be divided into reflection and transmission measurements (with θ_*o*_^ext^ ∈ [– 90°,90°] and θ_*o*_^ext^ ∈ [90°,270°] respectively). To characterize the optical response of CNC photonic materials, reflection measurements are usually preferred. While it is possible to obtain spectra for any combination of angles (θ_*i*_^ext^, θ_*o*_^ext^), the essential information is often captured by performing 1D scans through this 2D parameter space. Three types of reflection scans are especially useful:

- In a **specular** scan (Figure 43a), the incident and detector angles are both varied symmetrically relative to the surface normal of the sample (i.e., the angle θ_*i*_^ext^ = θ_*o*_^ext^ = θ^ext^ is varied). This scan captures the mirror-like (specular) reflection of the sample.
- In a **scattering scan** (Figure 43b), the incident angle θ_*i*_^ext^ is kept constant, and the detection angle θ_*o*_^ext^ is varied, which captures primarily nonspecular scattered light.
- In a **tilt scan** (Figure 43c), the angle between the incident light and the detector is kept constant and the sample is tilted relative to this fixed angle. In this case the variable is the tilt angle θ_*s*_ = (θ_*o*_^ext^ – θ_*i*_^ext^)/2, while the half-range Δθ^ext^ = (θ_*o*_^ext^ + θ_*i*_^ext^)/2 is fixed. This scan captures the reflection of tilted helicoidal domains.

##### 7.4.4.3 Comparison of Measurement Modes

**Figure 44:**
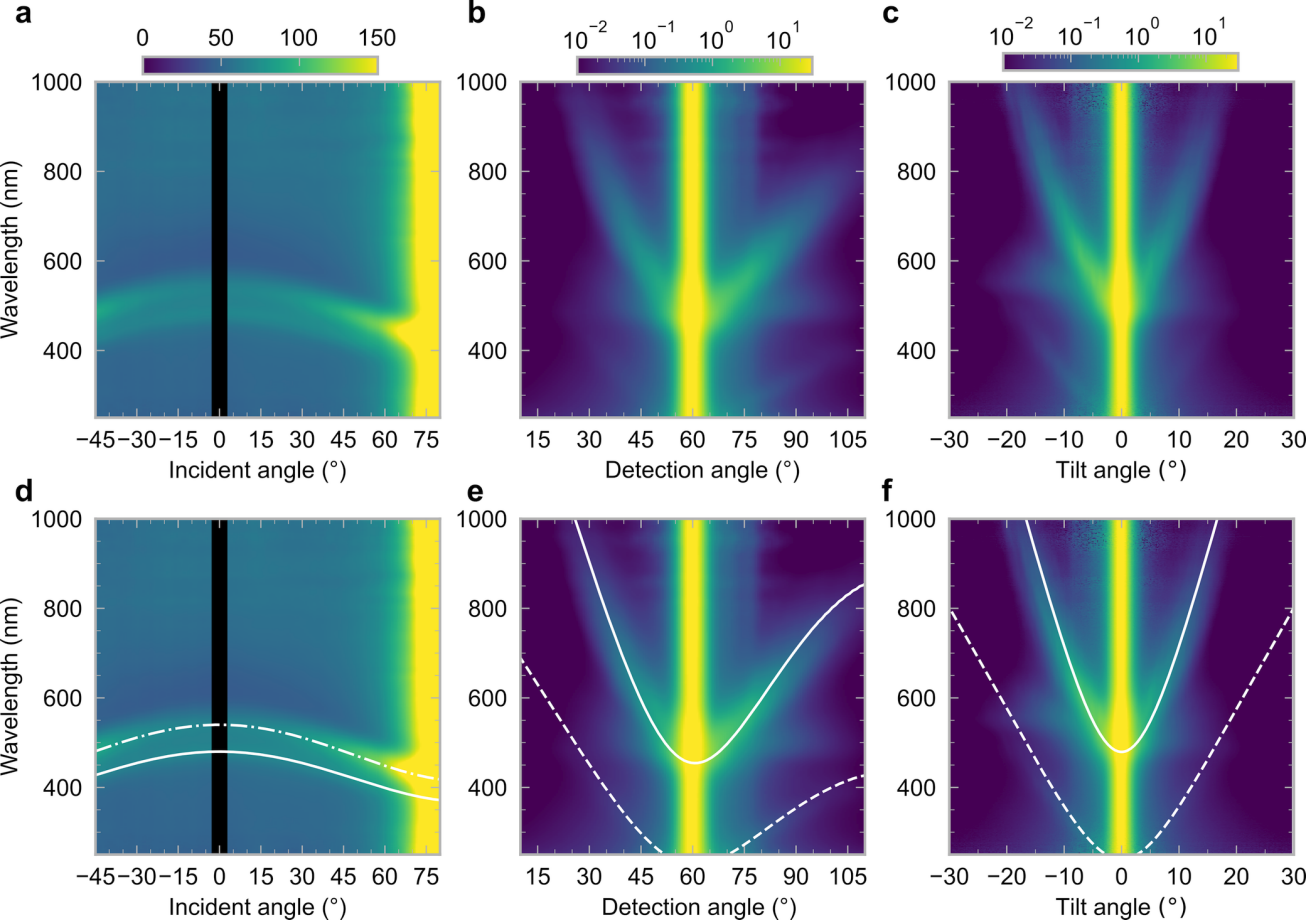
Angle-resolved optical spectroscopy of the example film (a) Specular scan, varying the incident angle θ_i_^ext^ (b) Scattering scan, varying the detection angle θ_*o*_^ext^ with θ_*i*_^ext^ = 30°, (c) Tilt scan, varying the tilt angle θ_s_ with Δθ^ext^ = 10°. The color bars above (a–c) indicate normalized reflectance (see section S3 of the Supporting Information for method). (d) Data from (a) with overlay of Bragg-like fitting, assuming two specular peaks for p_1_ = 309 nm (solid line) and p_2_ = 347 nm (dash-dotted line). (e) Data from (b) with overlay of a distortion curve fitting assuming p_1_ = 309 nm and α = 0.1. (f) Data from (c) with overlay of the distortion curve fitting, with the same fitting parameters as (e). In (e, f) both the first-order (solid) and second-order (dashed) lines are indicated. See section S2 of the Supporting Information for fitting equations.

To illustrate the optical signatures detected with each measurement mode, angle-resolved reflection spectra were obtained from the example film (see Supporting Information S3 for experimental methods). Each scan produces in a 3D data set (reflectance versus wavelength and angle) that can be visualized as 2D heatmaps with reflectance as the color axis, as shown in Figure 44. The key features of each scan can be fitted by modeling the sample as a patchwork of helicoidal domains of varying orientation and considering distortion of the helicoidal structure upon drying (discussed in section 6). The equations used for fitting of experimental data are summarized in the Supporting Information, section S2.

The specular scan (Figure 44a) exhibits two “arcs” that decrease in peak wavelength with increasing incidence angle |θ_*i*_^ext^|. These arcs correspond to Bragg-like reflection from the periodic helicoidal structure of the film. Crucially, however, a simple Bragg equation does not fit the data, as an additional correction must be included for the refraction of incident light at the air–film interface (as discussed in section 7.3.4). The fitting curves, shown in Figure 44d, correspond to pitch values of *p*_1_ = 309 nm and *p*_2_ = 347 nm. The presence of two specular peaks indicates a stratified structure within the CNC film, resulting in two apparent pitch values. Another feature of the specular scan is that the reflectance at all wavelengths increases sharply at |θ_*i*_^ext^| due to strong specular reflection of the air–film interface at high incidence angles.

In the scattering scan (Figure 44b), the highest reflectance is observed at the specular angle, namely θ_*o*_^ext^ = θ_*i*_^ext^ = 30°, which corresponds to θ_*i*_^ext^ = 30° in Figure 44a. At off-specular angles, an asymmetric arc that increases in peak wavelength with |θ_*o*_^ext^| is observed. This arc is in very good agreement with the model of unidirectional compression and distortion of tilted helicoidal domains discussed in section 6.1, as shown by the fitting in Figure 44d. This distortion also leads to higher-order reflection peaks (i.e., at wavelengths λ/2, λ/3 etc.), leading to a visible second-order reflection in the scattering scan. The data from the tilt scan (Figure 44c) are consistent with these observations: in this case, the peak is symmetric, and the specular condition occurs at a tilt angle *θ*_*s*_ = 0. Fitting of the tilt scan with the same parameters as the scattering scan is shown in Figure 44e. Interestingly, the arcs in the scattering and tilt scans correspond to the smaller of the two pitches observed in the specular scan, suggesting that before drying the sample was composed of a distribution of domains at a smaller pitch value, along with uniformly aligned domains of a larger pitch. A similar structure was previously reported, but with the uniformly aligned domains corresponding to smaller pitches.^345^

**Figure 45:**
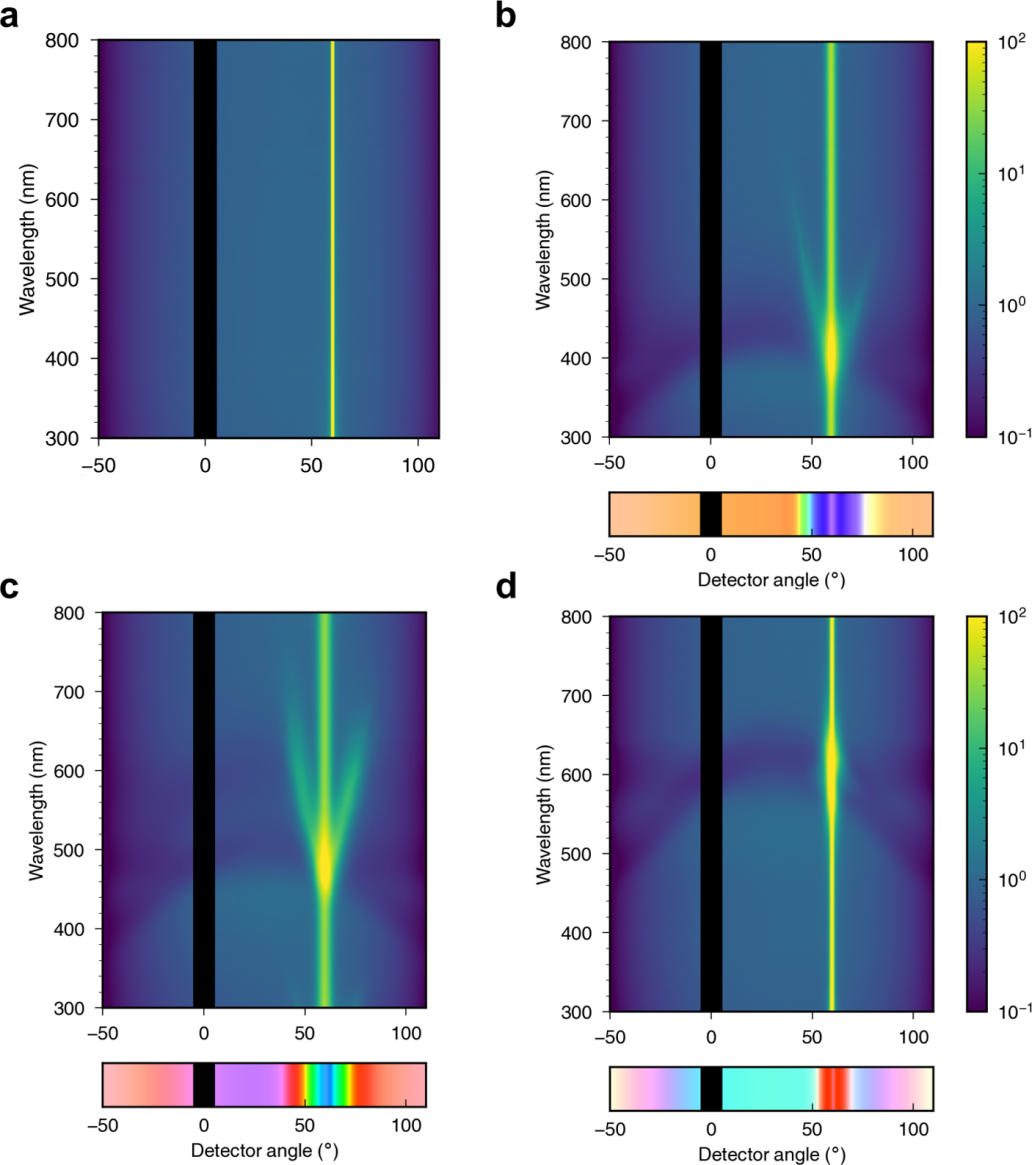
Angle-resolved optical spectroscopy scattering scans for (a) a diffuse white substrate, and substrates with a top of layer of (b) blue, (c) green, or (d) red CNC photonic film. Vertical color bar indicates normalized reflectance. Horizontal color bars in (b–d) indicate the RGB color corresponding to the spectrum at each angle. Reproduced with permission from ref (566) under CC-BY. Copyright 2022 The Authors.

Angle-resolved optical spectroscopy is also beneficial when CNC photonic films are combined with other components that have contrasting optical responses. For instance, a CNC photonic film assembled on a diffusive white substrate has conventional structural coloration (e.g., green) at the specular angle but exhibits the complementary color (e.g., magenta, white minus green) at off-specular angles, as shown in Figure 45.^566^ The off-specular coloration arises from light transmitted through the CNC film and is then diffusively scattered by the substrate. This nonabsorbing coloration achieved using CNC films has potential applications for radiative cooling materials, as discussed in section 10.3.1.^566^

##### 7.4.4.4 Practical Considerations

There are additional practical considerations that arise when performing angle-resolved measurements that are not apparent in fixed-angle optical spectroscopy. For example, the spot size (region of the sample illuminated by the incident beam) varies with the angle of incidence, which can be problematic for specular or tilt scans, as the illuminated surface is not exactly the same across the scan. Moreover, as the illumination angle increases (i.e., departs from normal incidence), the illumination area becomes elliptical and diverges at grazing incidence, causing artifacts whenever the spot size reaches the sample size. Using a narrow diameter optical fiber and a focusing lens adjusted to the distance from sample to detector, the sample distance can be used to reduce the light spot down to a millimeter or so, without compromising much on the angular resolution. To maintain good angular resolution, the orientation of the analyzed samples has to be uniform across the spot size, meaning the sample has to be flat at that scale, with a tolerance comparable with the angle window of the illumination and detection angles.

Another issue is the variation in the intensity of the optical response across several orders of magnitude. The reflection from a CNC photonic film is strongest near the specular angle, while reflection at off-specular angles is substantially less intense. Accurately capturing the entire optical signature from a CNC film without saturating the spectrometer is therefore a significant practical challenge. Implementing a high dynamic range (HDR) mode, even for one of these modes, is also useful to adjust the integration time across the variety of settings each sample might require maximizing the signal-to-noise ratio while preventing saturation of the spectrometer.

#### 7.4.5 Optical Microscopy and Microspectroscopy

Optical microscopy can be used to investigate the optical response of CNC films at the scale of individual helicoidal domains. The appearance of the sample in the resulting microscope images depends on the experimental setup of the microscope, i.e., how the sample is illuminated (e.g., bright field versus dark field illumination), how light is collected (e.g., reflection versus transmission, magnification and numerical aperture) and how the resulting image is generated (e.g., orthoscopic versus conoscopic imaging). Furthermore, the polarization state of the incident and collected light can also be controlled, and such polarized optical microscopy (POM) is especially beneficial for helicoidal structures such as CNC films.

Alongside the acquisition of microscopic images, an optical microscope can be used to collect reflection and transmission spectra from a specific area within the field of view of the microscope. This technique, often referred to as microspectroscopy, can achieve a resolution up to a few microns and can therefore be used to probe the optical response of individual domains, in contrast to the macroscopic spectroscopy techniques discussed in section 7.4.4 that provide spectra averaged over a large number of domains.

This section summarizes how different measurement modes and experimental configurations of the microscope can be used to characterize CNC photonic structures.

##### 7.4.5.1 Experimental Setup

**Figure 46:**
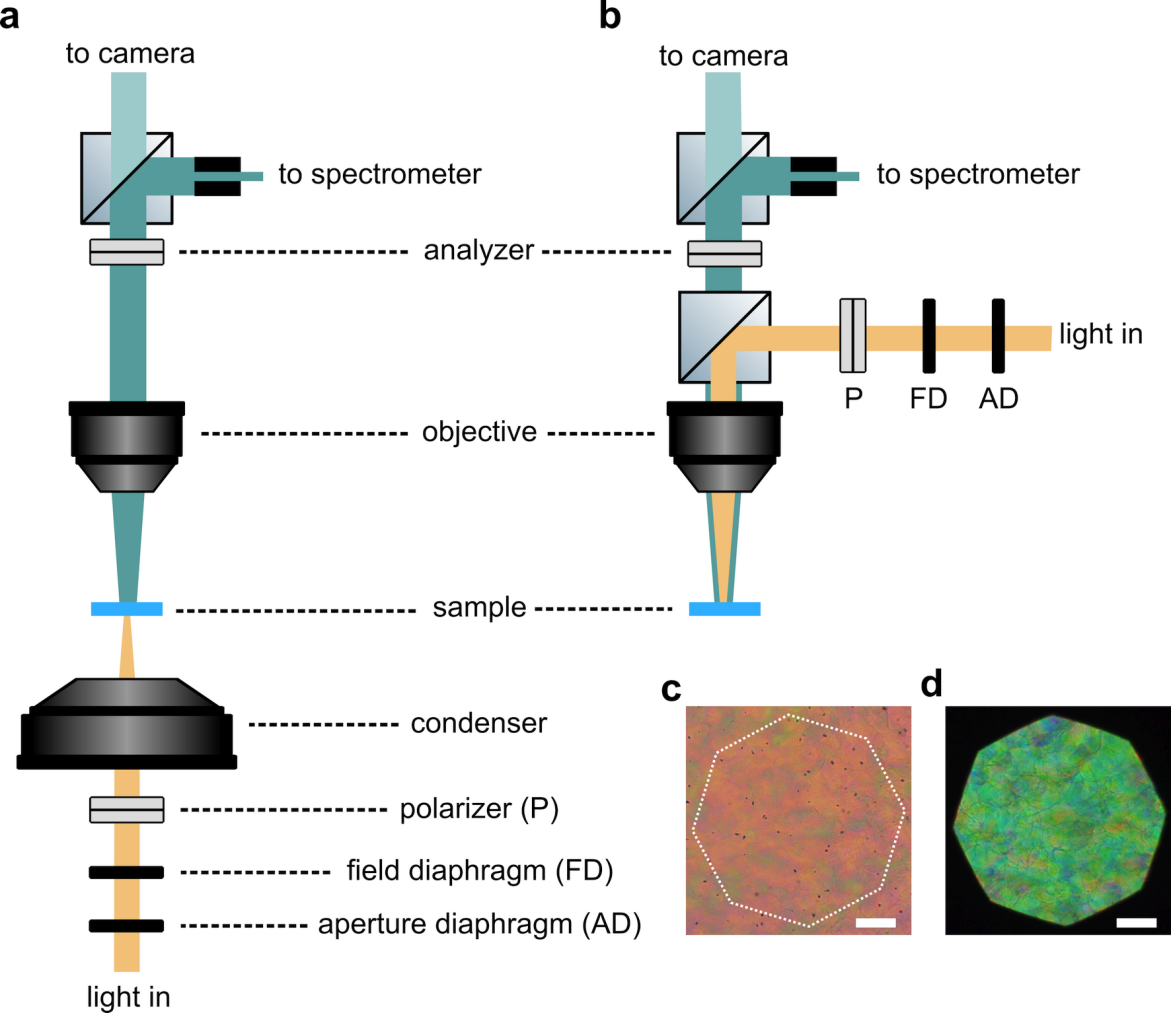
Schematic showing the light path for optical microscopy in (a) transmission mode and (b) reflection mode. The double slots in the polarizer and analyzer indicate where a linear polarizing filter and quarter-wave plate could be introduced for polarized imaging. Corresponding real-space optical microscopy images of the example film are shown for (c) transmission and (d) reflection mode, using unpolarized illumination. The octagonal boundary in (d) is due to the partial closure of field diaphragm to illuminate a selected region of the film, and matches the dotted white octagon drawn on (c) as a guide to the eye. Scale bar 100 μm.

The experimental setup for optical microscopy is shown in Figure 46. Optical microscopy can be broadly divided into transmission (trans-illumination) or reflection (epi-illumination) modes, depending on whether the transmitted or reflected light from the sample is collected for imaging. In transmission mode (Figure 46a), incident light is focused onto the sample using a condenser lens, and the transmitted light is collected by the objective lens. In reflection mode (Figure 46b) the objective lens plays both roles, focusing incident light onto the sample and also collecting the reflected light. The selective reflection of CNC photonic films is clearest in reflection mode, as exemplified in Figure 46d, while the complementary color (e.g., magenta, for a film reflecting in the green wavelength range) is observed in transmission mode (Figure 46c). Consequently, the following discussion will focus on reflection microscopy, although many points are also applicable to transmission microscopy.

##### 7.4.5.2 Illumination Conditions

The light incident on the sample can be controlled in terms of its spatial distribution (i.e., the size of the region of the sample illuminated) and its angular distribution (i.e., the range of angles focused onto the illuminated region). The size of the illuminated region is controlled by the magnification of the condenser, and by spatial filtering of the incident light using an adjustable aperture known as the field diaphragm (FD). The angular distribution is limited by the construction of the condenser to a value known as the numerical aperture (NA). For a lens separated from the sample by an air gap, the numerical aperture is NA = sin α, where α is half the opening angle of the ”light cone” illuminating the sample. An additional aperture, known as the aperture diaphragm (AD), can further limit the range of illumination angles and thus reduce the effective NA of light reaching the sample.

**Figure 47:**
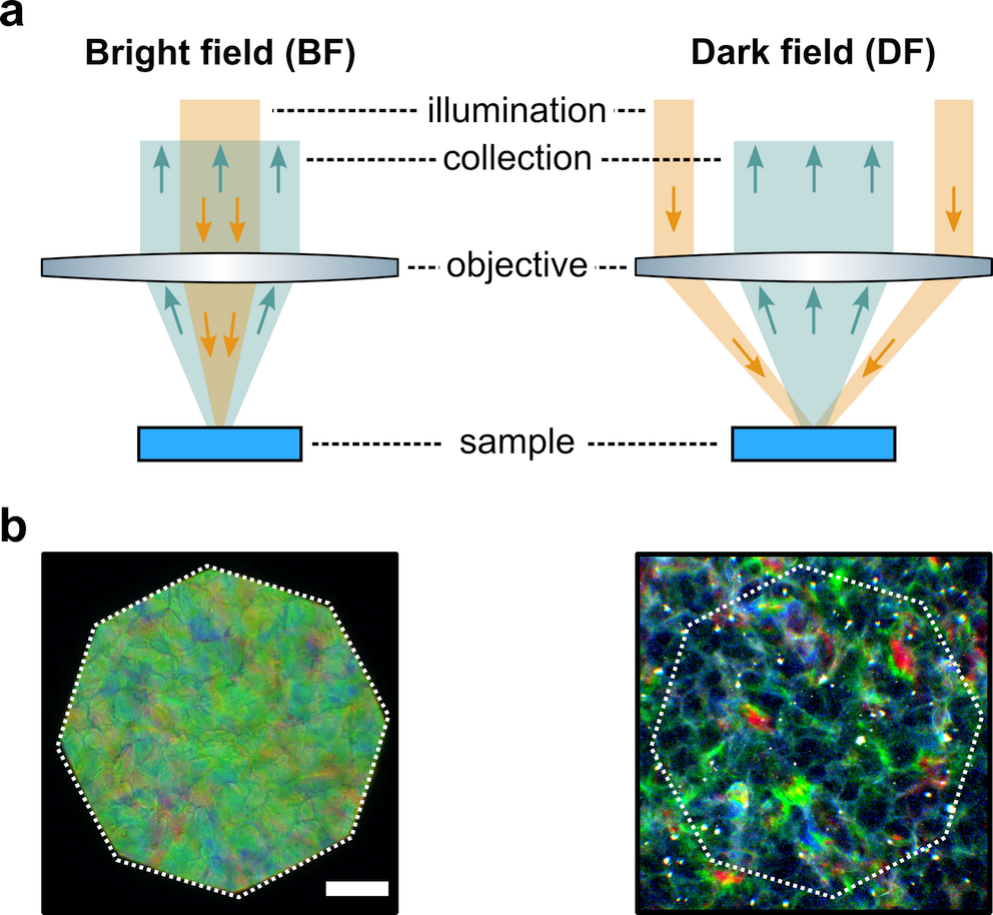
Comparison of bright field and dark field reflection microscopy (a) Schematic representing illumination and collection angles for bright and dark field imaging. Reproduced with permission from ref (567) under CC-BY. Copyright 2023 The Authors. (b) Optical microscopy with bright field and dark field illumination on a given region of the example sample. Images were acquired in reflection using unpolarized light. Dark field image was digitally modified to enhance the contrast. The dotted octagon matches the field aperture visible in (a) as a guide to the eye. Scale bar 100 μm.

Samples are typically illuminated with light as a small range of angles around normal incidence, which is known as bright field (BF) illumination. Alternatively, the sample can be illuminated only at higher angles of incidence, known as dark field (DF) illumination. By excluding the strong specular reflection that often occurs when the sample is illuminated at normal incidence, DF illumination enables the detection of weaker reflection from the structures scattering light at higher angles. A comparison of the two illumination modes is shown in Figure 47a, with images of the example film taken using bright and field illumination shown in Figure 47b.

When imaging CNC films, it is recommended to reduce the NA of illumination when seeking to measure only the direct specular response. Illumination with a larger NA will lead to a stronger signal, but averaged over a range of illumination angles.

##### 7.4.5.3 Objective Magnification and Numerical Aperture

While the illumination conditions determined by the condenser, the spatial and angular distribution of light collected from the sample is determined by the objective (although in the case of reflection microscopy, they are the same lens). The size of the region from which an image is acquired is determined by the magnification of the objective, while the range of angle collected is determined by its numerical aperture (NA). The NA of the objective has a critical impact on the appearance of photonic CNC films, as slightly tilted domains have a pronounced, red-shifted response due to reduced compression along the helical axis, as discussed in section 6.1.

**Figure 48:**
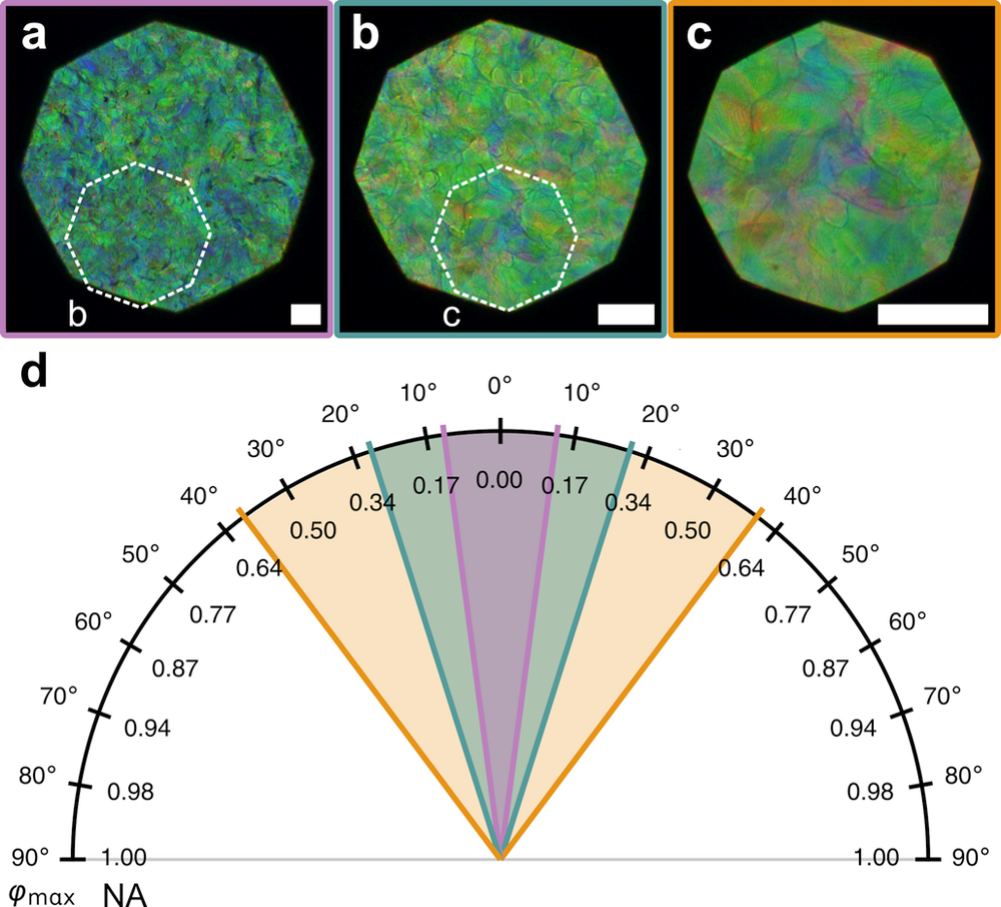
POM of a region of the example film at increasing magnification and numerical aperture (NA). (a) 5×, NA = 0.13 (b) 10×, NA = 0.30 (c) 20×, NA = 0.60. The domains are blue–green in (a), with red regions only apparent in (b) and (c) due to the higher numerical aperture of the objectives. (d) Quarter-circle indicating the half angle of the cone of light collect for each numerical aperture (line colors matching a–c). Scale bar for all images is 100 μm.

The effect of objective magnification and NA on the appearance of photonic CNC films is demonstrated in Figure 48. The example film was imaged with three objectives of increasing magnification and NA (Figure 48a–c). At low NA, the film appears green-blue, with very few red regions (Figure 48a). Switching to higher magnification objectives allows fine features of the helicoidal domains to be discerned, while a greater number of red and green domains are observed due to the higher NA (Figure 48c). The NA of the objectives can be represented as the half opening angle of the light cone, showing explicitly that a much wider range of angles can be collected at higher NA (Figure 48d).

##### 7.4.5.4 Detection Method

The light from the sample that is collected and focused by the objective must then be captured by some type of detector, typically a digital camera (for conventional optical microscopy) or a spectrometer (in the case of microspectroscopy).

Cameras typically filter the collected light to record separate intensity values for red, green and blue portions of the spectrum, resulting in three values (RGB) for each pixel. The acquired image depends not only the visual appearance of the sample but also the settings of the camera, notably the exposure time and gain, which must be kept identical between measurements to ensure consistency. Another important consideration is the color balance of the image (i.e., the relative intensity of R, G, and B channels). The broadband light sources used for optical microscopy vary in intensity with wavelength (e.g., light from halogen lamps typically appears yellow-orange), which introduces a bias in the resulting image. To remove the effects of the light source, the color balance of the camera should be standardized by performing a “white balance” using a white reference material.

Microspectroscopy is typically performed by introducing a beam splitter into the light path to divide the outgoing light between a camera and a fiber-optic cable, as shown in Figure 46a and b. The fiber-optic cable is mounted at a fixed distance confocal to the camera image plane, so that the acquired spectrum corresponds to a given spatial region of the sample. The other end of the fiber-optic cable is then coupled to a spectrometer. Spectra should be normalized to a suitable reference material: for reflection spectra, this is typically a mirror or diffuse reflectance standard, while for transmission spectra the reference is typically acquired by leaving the beam path empty. The absolute reflectance or transmittance values, when properly normalized, contain useful information about the optical response, and are therefore preferred over self-normalized spectra. For polarized microspectroscopy (discussed below), the reference material should be measured under identical polarization conditions to the sample where possible.

For a given sample, it is possible to use the measured spectrum to estimate the apparent color (in terms of RGB values) under given illumination conditions. This conversion process is nontrivial due to the nonlinear response of the human eye to light, and varies with the light source, but has been standardized by the International Committee on Illumination (CIE).^568^ The resulting values are typically plotted in a color space, such as the CIE 1931 xy chromaticity diagram.

##### 7.4.5.5 Polarized Optical Microscopy (POM)

The polarization-dependent optical response of the sample can be probed by selecting the polarization state of the incident and the collected light. The polarization mode of the microscope is determined by the optical components added into the beam path at the positions indicated in Figure 46a and b: components that modulate the polarization of the incident light are collectively referred to as the “polarizer”, while components that modulate the polarization of the reflected/transmitted light before reaching the detector are known collectively as the “analyzer”.^532^ The optical components that can be introduced are typically a linear polarizing filter (also known as a linear polarizer) and a quarter-wave plate (discussed in section 7.2.2). The way in which these components can be combined to achieve a desired polarization mode is described in the introduction to section 7.4 above.

**Figure 49:**
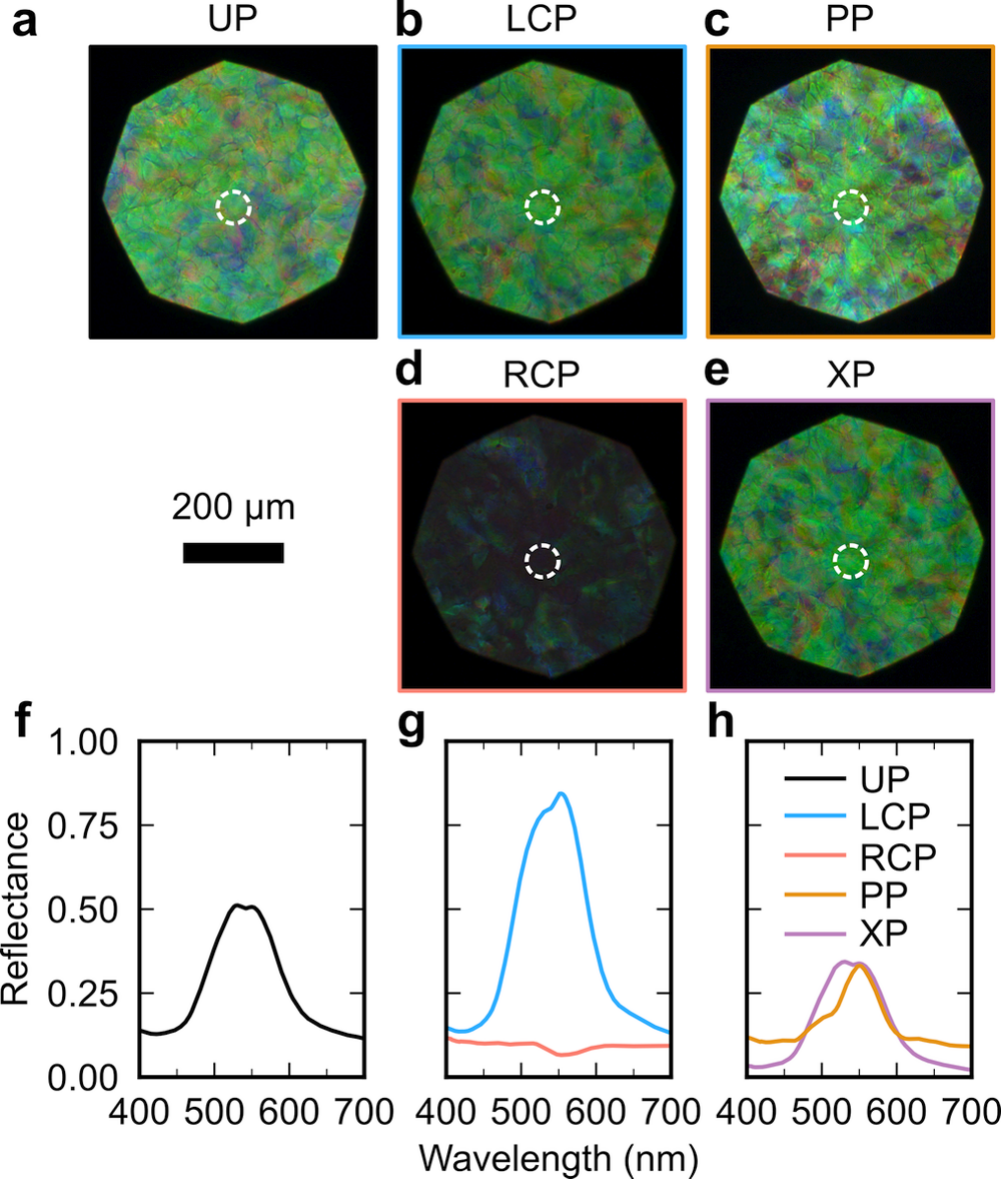
Polarized optical microscopy (POM) and corresponding microspectroscopy of a selected region of the example film. Images were acquired in reflection using (a) unpolarized light; with (b) LCP or (c) RCP analyzers, or taken between (d) parallel polarizers or (e) crossed polarizers. All images were acquired using a 10×/0.30 objective with bright field illumination. Note that the camera exposure time was adjusted between the three sets of images (UP, LCP/RCP, PP/XP) for clarity. (f–h) Microspectroscopy was performed for each polarization mode by collecting light from the region indicated by the dotted white circle in (a–e). The spectra are normalized to (f) a mirror under UP illumination, (g) a mirror with LCP or RCP analyzer, (h) a mirror between PP.

POM in reflection mode for the example film is shown in Figure 49. The visual appearance of the film in unpolarized illumination (Figure 49a) arises from the selective reflection of LCP light by the helicoidal structure, which can be demonstrated by comparing the images obtained with a LCP or RCP analyzer (Figure 49b and d, respectively). Imaging of the sample with parallel polarizers (i.e., with the polarizer being a linear polarizing filter and the analyzer being a second linear polarizing filter parallel to the first) also allows the selective LCP reflection to be observed, but enhances the specular reflection of the sample interface (Figure 49c). Alternatively, the sample may be observed between crossed polarizers, where the specular reflection is suppressed (Figure 49e).

The optical response of the sample as seen in POM images can be quantified using polarized microspectroscopy. Figure 49f–h show the microspectra corresponding to the circular regions indicated in Figure 49a–e.

**Figure 50:**
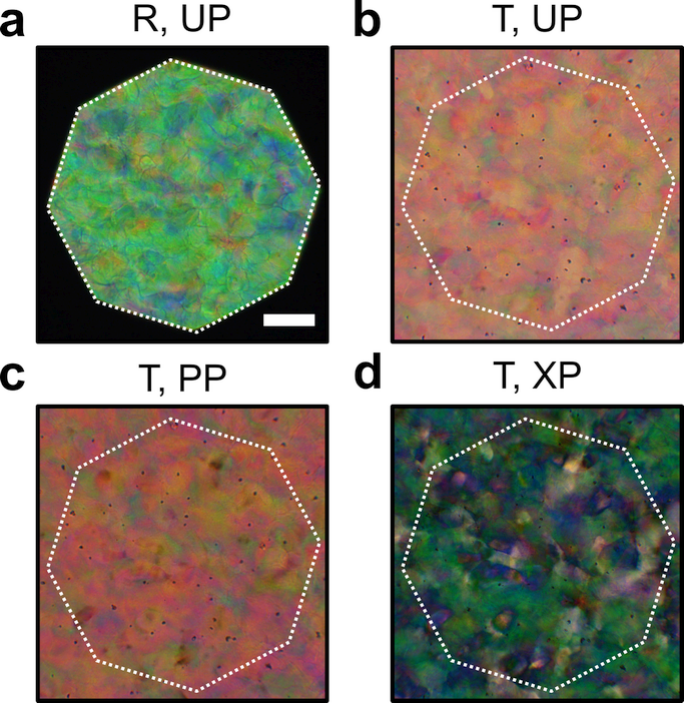
Comparison of POM images for one region of the example film viewed in reflection (*R*) and transmission (*T*). (a) Reflection with unpolarized light. (b–d) Transmission mode with (b) unpolarized light, (c) parallel polarizers and (d) crossed polarizers. The white octagonal overlay on (b–d) indicates the edge of the field aperture in (a), to aid comparison of the images. All images were acquired using a 10×/0.30 objective with bright field illumination. The scale bar in (a) is 100 μm and applies to all images.

As discussed above, CNC photonic films viewed in transmission mode exhibit complementary color to their appearance in reflection mode (Figure 50a,b). This complementary color is enhanced when viewed between parallel polarizers (Figure 50c) or with an LCP analyzer. In contrast, imaging between crossed polarizers leads to coloration similar to reflection mode (Figure 50d) as the transmitted light in the wavelength range of the photonic bandgap is predominantly RCP, rather than linearly polarized as it is outside the stopband. Using POM in transmission is not particularly of interest for assessing the main photonic properties, but rather for assessing the defects, such as the linear birefringence due to shear alignment of tilted domains. However, it has been used to show full photonic band gaps by blocking more than 50% of the light.^569^ Alternatively, transmission between slightly uncrossed polarizers can be used on well aligned cholesterics to measure the optical rotation.

##### 7.4.5.6 Advanced Imaging Methods

Optical microscopy of CNC photonic films is typically used for real-space (orthoscopic) imaging, in which the position of a feature on the image corresponds to a spatial position in the sample. Alternatively, microscopy can be used for k-space (conoscopic) imaging, also known as Fourier plane imaging, in which features appear according to the angle at which they escape the sample, with light collected at higher angles appearing farther from the center of the image.^161^ K-space imaging is achieved by inserting an additional lens (a Bertrand lens) into the conjugate back focal plane of the microscope after the objective. K-space imaging is a microscale analogue of imaging scatterometry, a technique in which an ellipsoidal mirror is used to collect the macroscopic angle-resolved reflection from a sample,^570^ as previously demonstrated for CNC photonic films.^564^ K-space imaging also has similarities to angle-resolved optical spectroscopy (section 7.4.4), but lacks spectral resolution when performed using a conventional RGB camera. A refinement of k-space imaging combines a polarizer and an analyzer (usually crossed) to construct what is known as a Kossel diagram,^543^ although this approach has not yet been applied to CNC systems.

CNC films can also be characterized by hyperspectral imaging (HSI),^345^ where a spectrum is acquired for each closely spaced point across a sample. In contrast to conventional imaging, where three values (RGB) are obtained for each pixel, a hyperspectral image records a full spectrum at every position, resulting in a 3D data set (i.e., x,y,λ). HSI can be performed by performing microspectroscopy as described above and sequentially scanning across the sample. Alternatively, HSI can be achieved using a commercially available hyperspectral camera, or by using a conventional camera combined with a set of band-pass filters in the optical path of the microscope (either in illumination or in collection). A similar experimental approach combines this approach with Mueller matrix ellipsometry (see section 7.4.4) to quantify microscale variation in the optical properties, as demonstrated (at fixed wavelength) for CNC photonic films.^571^

#### 7.4.6 Cross-Sectional Scanning Electron Microscopy (SEM)

**Figure 51:**
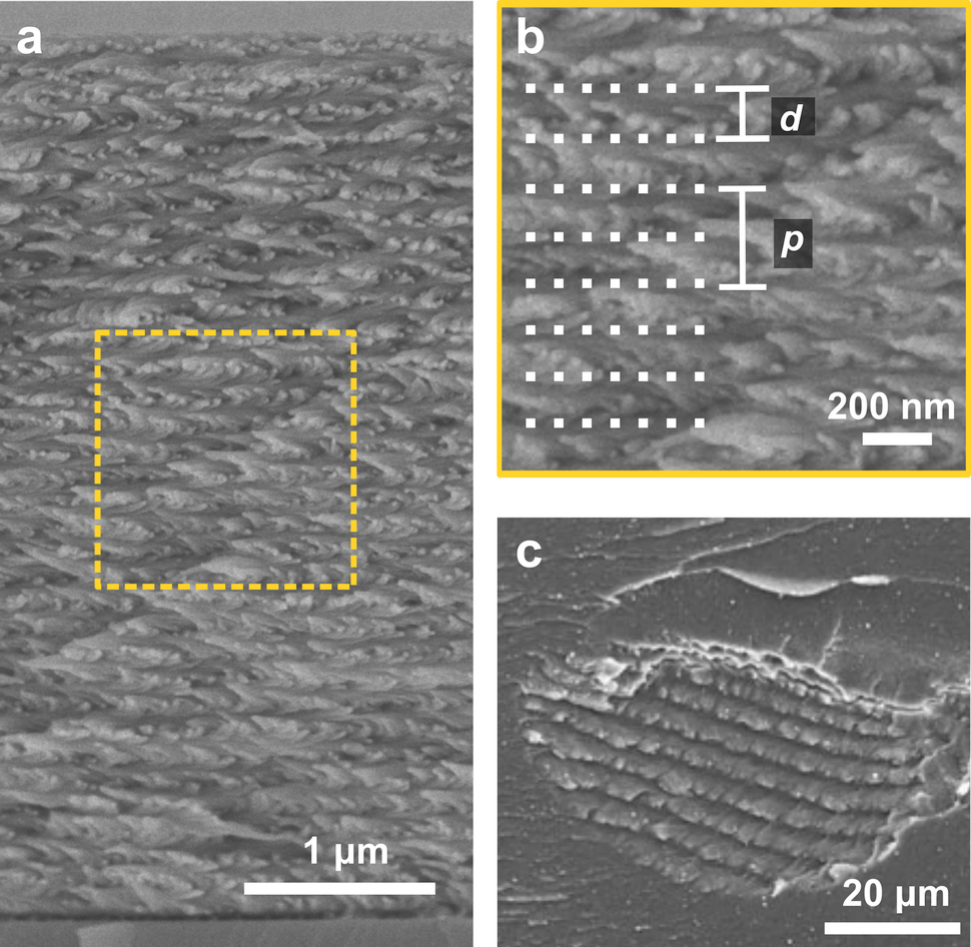
Cross-sectional SEM of photonic CNC films. (a) Full film cross-section showing uniform cholesteric ordering. Adapted from ref (338) under CC-BY. Copyright 2019 The Authors. (b) Inset of (a), with periodic texture marked by dotted white lines. The pitch *p* and periodicity *d* = *p*/2 are indicated. (c) Cross-section of a photopolymerized cholesteric tactoid. Adapted from ref (333) under CC-BY. Copyright 2016 The Authors.

Although SEM is not an optical characterization technique, the importance of cross-sectional SEM imaging for the characterization of photonic CNC films justifies a dedicated discussion here. As first introduced by Majoinen et al.,^572^ a CNC film can be pulled apart to expose a cross-section with a periodic texture, as shown in Figure 51a. The presence of “Bouligand arches” in the film cross-section is characteristic of cholesteric-like ordering, as also seen in biological twisted plywood structures.^516^ This texture can also be observed by TEM on microtomed samples,^20^ but is more easily accessed by cross-sectional SEM.

The Bouligand texture is best observed in cross-sections that are obtained by crack propagation through the film when it is pulled apart into two fragments, rather than being bent or cut. The observation of the Bouligand pattern in the cross-section, after the deposition of some thin sputter coated layer of conductive metal, is then made possible by the orientation of the CNCs periodically sticking out of the cross-sectional plane.

SEM cross-sections enable the measurement of the pitch and tilt angle of helicoidal domains within the CNC film. It is important to note that the periodicity of the Bouligand texture is a half-pitch distance *d* = *p*/2 and the pitch is therefore the spacing of two pattern repeats, as indicated in Figure 51b, as this is often a source of confusion. Quantitative analysis of the tilts and periodicity has been reported in many publications and usually consists in measuring the periodicity of the pattern and taking an average value and standard deviation, but more information can be extracted if properly interpreted.^270,553,573−575^ However, the interpretation of these numbers is subject to several considerations, namely the orientation of the cross-sectional plane, and the meaning of the average pitch values and helical orientation.

First, the fracture plane for the cross-section should be made perpendicular to the film surface, and the SEM imaging should be performed at normal incidence with respect to the cross-sectional plane, to avoid skewing the scale of the field of view. A flat cross-section will appear on focus while tilted cross-sections will contain highly blurred regions even at lower magnification.

Second, it is important to remember that for a domain that is tilted relative to the cross-section, the apparent periodicity and tilt angle of the Bouligand texture are only projections of the true periodicity and tilt onto the plane of the cross-section,^416^ as illustrated in Figure 27 in section 6.1. The apparent pitch *p*_app_ and apparent tilt β_app_ can be related to their real values (*p* and β) if the azimuthal angle φ between the domain helical axis and the cross-sectional plane is known. However, this angle is not accessible from the sole cross-sectional SEM image, and as a consequence the real values *p* and β cannot be obtained with absolute certainty, although it is necessarily true that *p* ≤ *p*_app_ and β ≥ β_app_. The Bouligand texture gives some indication of the azimuthal angle, as clear asymmetric arches (pointing either as ∪ ∪ ∪ or ∩ ∩ ∩) are expected if the azimuthal angle φ is closer to 90° (thus associated with small β_app_ values), while the arches should become more like |⋮ | ⋮ | ⋮| when φ ≈ 0°, indicative that we then have *p* ≈ *p*_app_ and β ≈ β_app_.

A further consideration when measuring the pitch from SEM cross-sections is the effect of anisotropic compression (discussed in section 6.1). The dramatic increase in *p* with tilt angle suggests that for polydomain films, the average pitch measured across many domains is likely to be much greater than the pitch at normal incidence (i.e., *p*_app_ is averaged over many values of β_app_). A probably more informative quantity approaching the real *p*(β) in the sample is the smallest pitch value  of a sufficiently representative total number of individual domains *i* observed, which should be more consistent with, e.g. the peak wavelength observed in reflection at normal incidence. The distortion of the helicoidal structure upon drying also results in asymmetric Bouligand arches.^416^ This asymmetry is the most visible in highly tilted domains (β ≠ 0) with intermediate azimuthal angle (φ ≈ ± 45) to visualize the arches, resulting in moderate apparent tilt (0 < β_app_ < β) and an apparent pitch larger than the real pitch, the latter being much larger than the pitch in nontilted domains (*p*_app_ > *p* > *p*(0)).

### 7.5 Characterization of Cholesteric CNC Suspensions

Several of the optical techniques described in section 7.4 can be applied to CNC suspensions to measure the anisotropic phase fraction and the cholesteric pitch, as detailed below.

#### 7.5.1 Photography

As discussed in section 4.2, cholesteric CNC suspensions are usually photographed in transmission between crossed polarizers (with a bright diffuse background) to visualize macroscopic phase separation between a top isotropic phase, appearing dark, and a bottom anisotropic phase, appearing bright. In uncrossed polarization configuration, the opposite occurs, namely the isotropic phase appears bright and the isotropic phase is darker (the nonbirefringent containers then remain visible). Aside from direct transmission, observation of cholesteric CNC suspensions in transmission with oblique illumination has occasionally been reported. In this configuration, the isotropic phase appear transparent, while the cholesteric phase appear highly iridescent with an intensity that depends on the helical alignment.^249^

To obtain high-quality photographs, the containers must be nonbirefringent. This condition is usually satisfied by glass containers such as vials or capillaries, as well as amorphous quartz cuvettes, but is not satisfied by many plastic containers (e.g., polypropylene centrifuge tubes or polystyrene Petri dishes). CNC suspensions in glass capillaries with a rectangular cross-section can exhibit a Grandjean texture when imaged through their thickness, where the planar anchoring on the flat sides of the capillary favors a helical alignment parallel to the viewing direction, with a few “oily streaks” corresponding to localized defects where the helical axis is perpendicular to the viewing direction. The uniform regions usually appear dark gray, instead of being fully dark, due to optical rotation (see section 4.2 and Figure 14 for a photograph, and section 7.3.3 for rotatory power), and full extinction is achieved by slightly uncrossing the polarizers to compensate for the rotatory power of the cholesteric. When directional illumination is used in the background, without any polarizer used, the cholesteric phase appears iridescent and acts as a grating, as explained in section 7.5.3.

#### 7.5.2 Optical Microscopy

As previously discussed in sections 3.2.5 and 4.2, the anisotropic phase fraction and cholesteric pitch can be observed by examining concentrated CNC suspensions in capillaries. As an example, the use of crossed polarizers (section 7.4.1) is of particular interest to observe and measure the periodicity of the fingerprint pattern in cholesteric suspensions, where alternating dark and bright fringes correspond to the alternating director direction respectively out and in the observation plane. In this case, to achieve good images, the use of long or super long working distance objectives with the smallest NA is recommended to increase the depth of field, which allows blurring the fingerprint pattern of tilted domains and thus resolve only the pitch of cholesteric regions whose helical axis lies within the observation plane.^288^ Alternatively, the cholesteric pitch in CNC suspensions could be measured using Grandjean-Cano wedges, a technique that is widely used for molecular liquid crystals (see also sections 4.2.1 and 7.3.7).^377−379^

The local birefringence of a CNC suspension can be characterized using a PolScope.^422,432,576^ This transmission optical microscopy technique uses additional optical components that enable the collection of images at various polarizer and analyzer settings, enabling the retardance and the direction of alignment to be mapped across the sample.^577^ As a consequence, the PolScope can be used to determine the local birefringence of the sample (or more precisely, the birefrigence within the plane of observation), and its direction of alignment (within the observation plane). Reusing the terminology introduced in section 3.1.2 (also section 7.2.2), the PolScope image can show the sample isochrones without its isoclines, while the azimuthal orientation of the anisotropy can be displayed separately.

As a final comment, confocal laser scanning microscopy (CLSM, also known as LSCM) can also be used to observe the fingerprint pattern in cholesteric CNC suspensions,^405,578^ as previously demonstrated for amyloid fibril suspensions.^250^ Unlike conventional CLSM, where imaging contrast is provided by a fluorescent dye, CNC suspensions are imaged between crossed polarizers so that contrast is provided by the helicoidal variation of the local birefringence. For gelled CNC suspensions, contrast can also be provided by poststaining the samples with fluorescent dye.^384^

#### 7.5.3 Laser Diffraction

**Figure 52:**
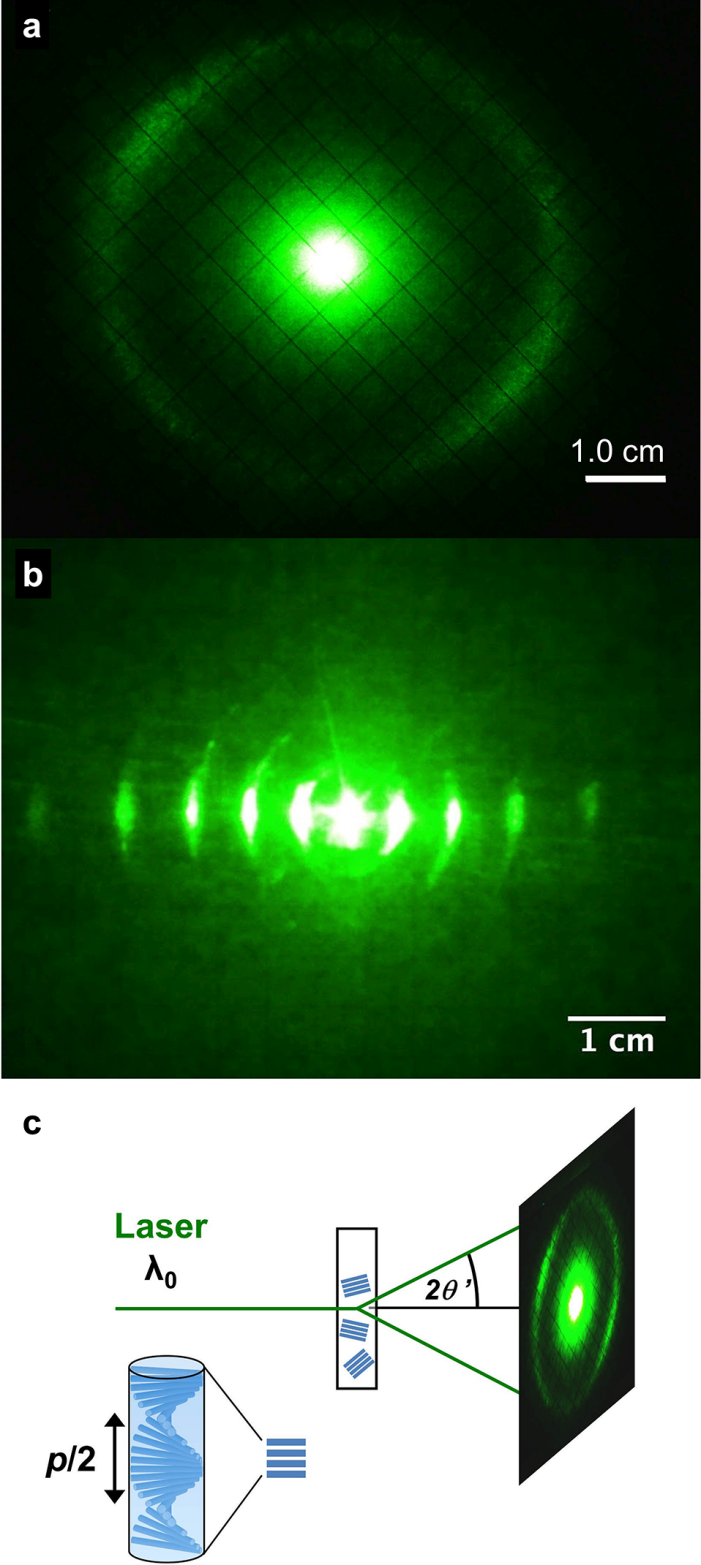
Laser diffraction from a cholesteric suspension of CNCs. (a) Polydomain structure with multiple orientations of the helical axis. (b) Monodomain structure with the helical axis in the horizontal direction. (c) Schematic of the diffraction geometry. Adapted with permission from ref (288) under CC-BY. Copyright 2016 The Authors.

Laser diffraction can be useful to characterize cholesteric CNC suspensions, to extract both the pitch values as well as the azimuthal helical axis alignment in the plane perpendicular to the illumination direction. The diffraction pattern is observed in transmission (Laue diffraction) and with visible light this is possible only when the pitch is in the micron range, explaining why this is mainly relevant to suspensions. The use of rectangular cuvettes or flat capillaries is required to keep the orientation of the local incident and diffracted light beams tractable inside the sample, and after accounting for Snell’s law correction of the Bragg’s law, the angular dependence of the reflected wavelength appears very similar to the one expected from the grating equation (see section 7.3.5). A randomized polydomain assembly of cholesteric domains of same pitch then produces a diffraction ring of a given radius on a screen placed in the background, and the angle at which it forms allows to determine the pitch knowing the laser wavelength. This technique provides the advantage of giving robust measurements of the pitch averaged upon the beam cross-sectional area and additional angular information about the orientation of the contributing domains (Figure 52).^249,288,414^

### 7.6 Numerical Tools for Simulating the Reflection of Cholesteric Materials

#### 7.6.1 Monodomain Structures

Several numerical methods have been developed to calculate the reflection and transmission spectra of cholesteric structures for both normal and non-normal incidence. These methods usually model the structure as a quasi-1D vertical stack of discrete birefringent layers and estimate the optical response from the summation of contributions for forward and backward propagating electromagnetic waves produced by the reflection and transmission at each interface. A widely used approach is the transfer matrix (TM) method developed by Berreman, known as the *Berreman 4 × 4* matrix method,^579^ which can be performed using open-source software.^580^ For example, the *Berreman 4 × 4* matrix method has been shown to be in excellent agreement with finite-difference time domain (FDTD) simulations^564^ and experimental data for molecular cholesterics.^581^ For thin monodomain photonic CNC films, fitting of reflectance spectra to Berreman 4 × 4 simulations has been used to estimate the optical properties of the CNC helicoidal structure.^337^

**Figure 53:**
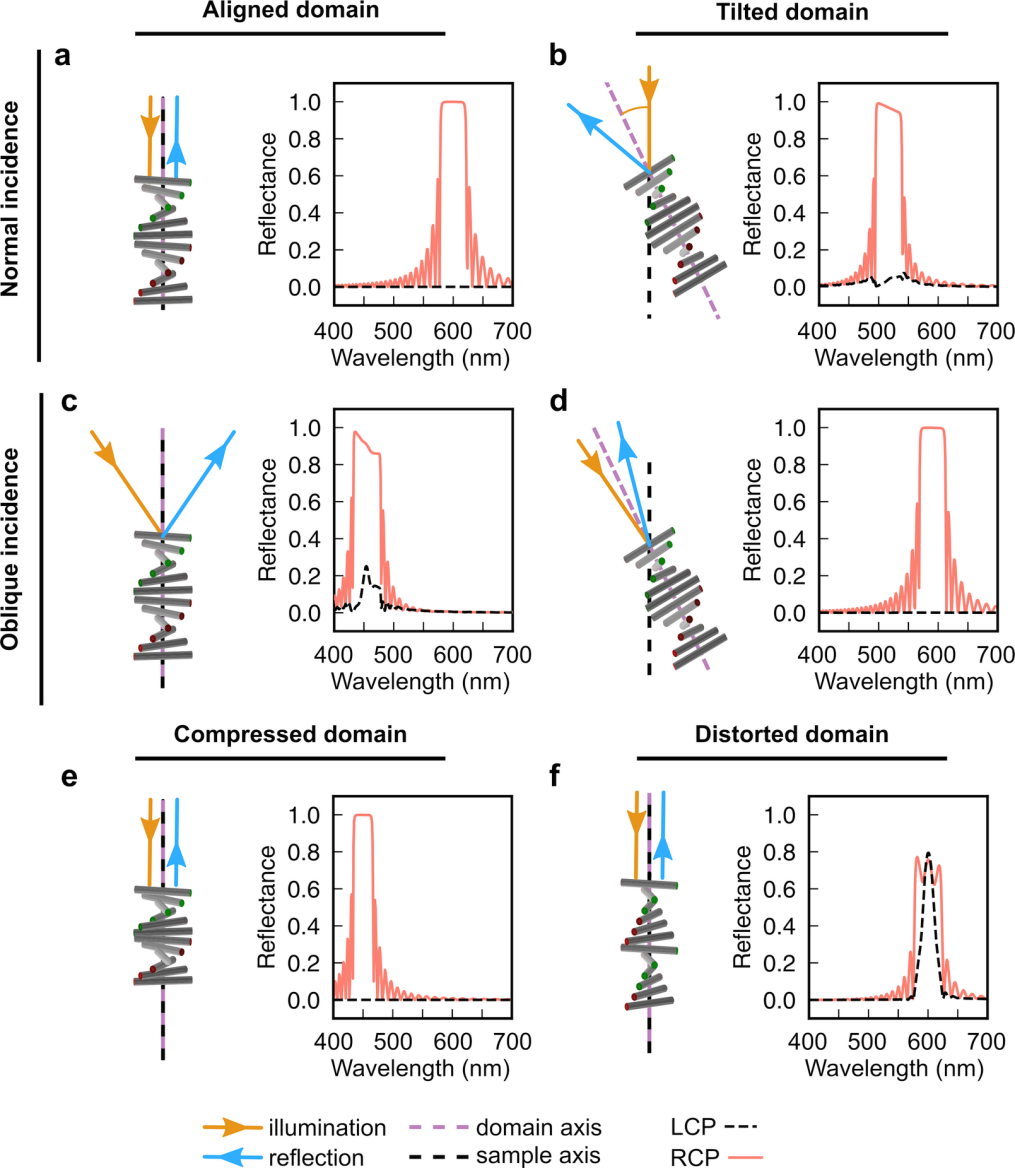
A selection of right-handed helicoidal structures, shown as 3D representations of the local director, and associated LCP and RCP reflectance spectra modeled using a SM method Python code (PyLlama^583^). Examples shown correspond to (a) an aligned domain illuminated at normal incidence, (b) a tilted domain at normal incidence, (c) an aligned domain at oblique incidence, (d) a tilted domain at oblique incidence, (e) an aligned domain compressed along the helical axis, and (f) an aligned domain with a distorted helicoidal structure. The sample is assumed to be a 10 μm-thick monodomain with the following parameters: *p* = 400 nm, *n* = 1.5, Δ*n* = 0.1. Note that a right-handed structure is assumed, leading to preferential RCP reflection, in contrast to the left-handed structure of CNC photonic films. For simplicity, the effects of the air-sample interface (e.g., refraction) are not included in these simulations. Reproduced under CC-BY from ref (567). Copyright 2023 The Authors.

TM methods suffer from numerical instabilities when the helicoidal stack is sufficiently thick (e.g., 100s of pitch repeats, assuming the optical properties of CNC films). This numerical instability arises from the coexistence of evanescent contributions in the forward and backward propagating waves, that exponentially decrease and increase with sample thickness, respectively. This issue can be avoided by employing the scattering matrix (SM) method, wich handles the evanescent wave contributions in a way that avoids the coexistence of exponentially large and small terms.^582,583^ A SM method was recently developed for multilayered anisotropic media, with a special adaptation to cholesteric stacks, which can also be used to simulate tilted and distorted cholesteric arrangements such as those found in dried CNC films,^583^ and is available as an open source Python code, *PyLlama*.^584^ As shown in Figure 53, the SM method can be used to simulate polarized reflectance spectra for helicoidal structures at any angle of incidence for both ideal and distorted structures.

#### 7.6.2 Polydomain Structures

**Figure 54:**
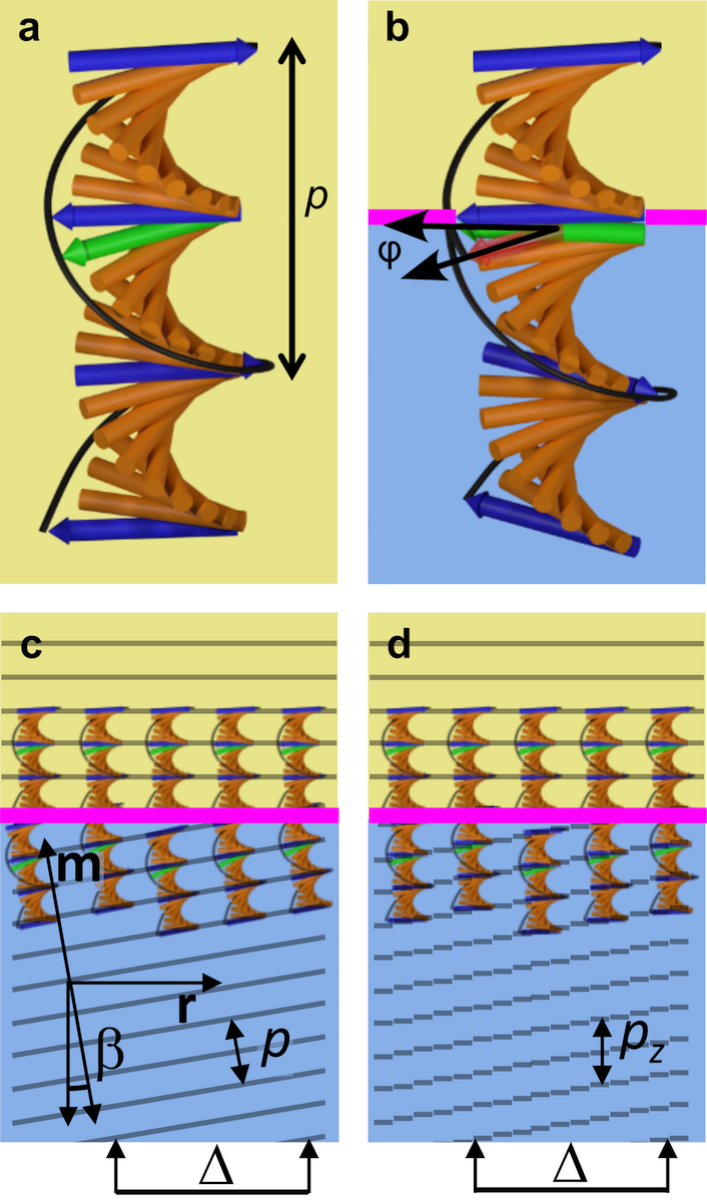
(a) Representation of the local director (orange) in a helicoidal monodomain (yellow). Blue arrows indicate half-turns of the director, with the pitch *p* also indicated. (b) A model defect between two vertically aligned helicoidal domains (yellow and blue). The local director varies discontinuously across the boundary (magenta) with a phase mismatch angle φ. (c) If the lower domain has a tilt angle β, the phase mismatch varies periodically as φ(*r*) = π*r*/Δ, where the lateral periodicity is Δ = *p*/(2 sin β) = *p*_*z*_/(2 tan β). (d) For small tilt angles, the optical response can be simulated by approximating the structure as two vertically aligned domains, where the bottom pitch is *p*_*z*_ = *p*/cos β ≈ *p* and the phase mismatch at the boundary varies as φ(*r*). Reproduced with permission from ref (345) under CC-BY. Copyright 2020 The Authors.

While a monodomain helicoidal structure (Figure 54a) can be directly simulated using TM or SM methods, polydomain structures such as those found in real CNC films present an additional simulation challenge, as the optical properties of the structure vary discontinuously at the boundaries between domains. One of the simplest models of a defect at a domain boundary is a vertical stack of two helicoidal structures of equal pitch, but with a mismatch in the phase of the director, φ, at the boundary between the two domains, as shown in Figure 54b. Although this type of defect can readily be simulated using TM or SM methods, it assumes that the two domains are perfectly aligned. However, the domain tilt angles found in polydomain CNC films are typically fairly small (on average |β | < 5°), and in this case the boundary between the two domains can be approximated by the approach shown in Figure 54c and d.^345^ A domain with a small tilt angles β can be modeled as a vertical stack with a projected pitch *p*_*z*_ = *p*/cos β ≈ *p*. In this case, the phase mismatch at the domain boundary varies periodically with lateral distance r according to φ(*r*) = πr/Δ, where the lateral periodicity is Δ = *p*/(2 sin β) = *p*_*z*_/(2 tan β).

**Figure 55:**
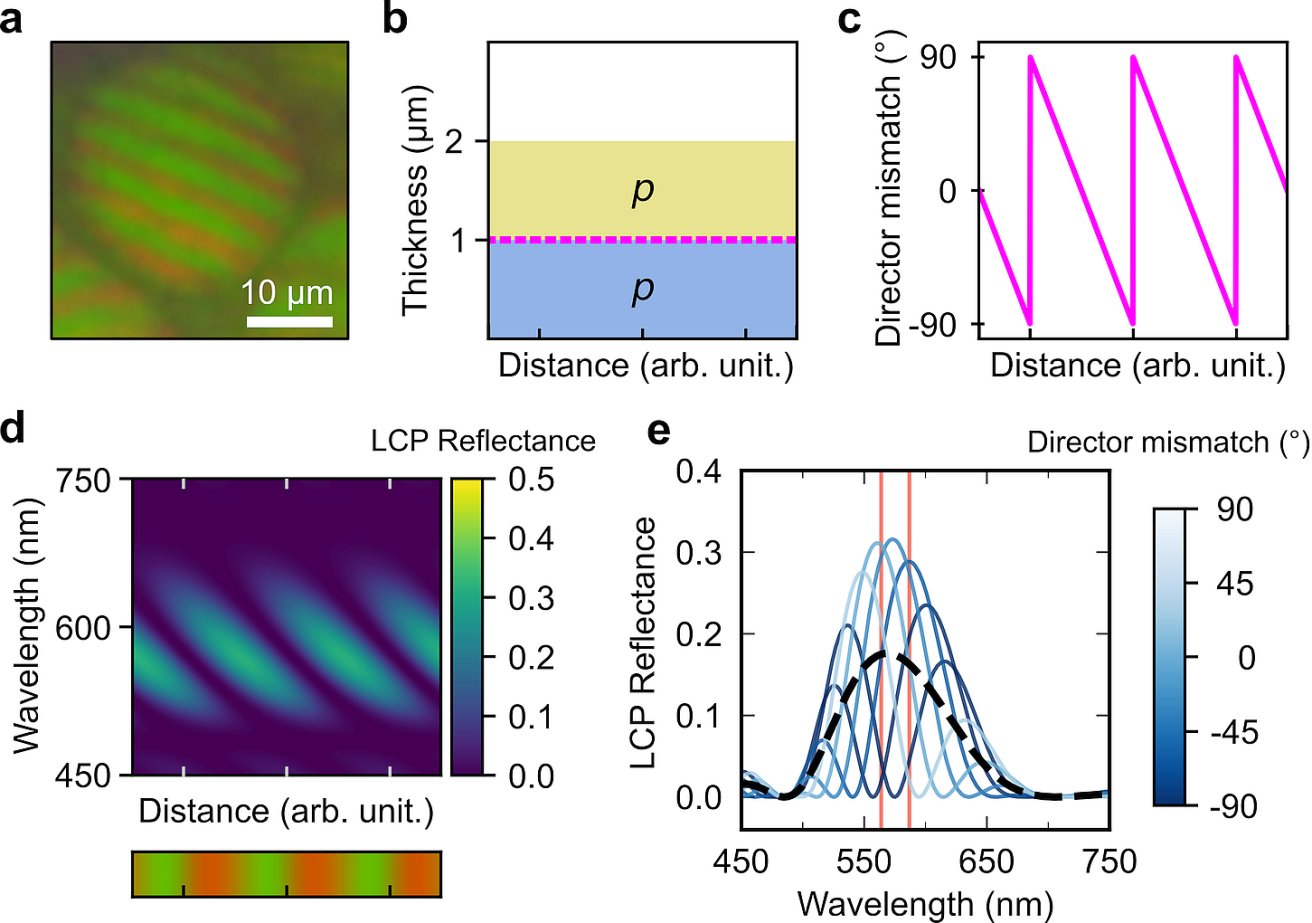
Simulation of reflection spectra arising from defects at boundaries between helicoidal domains. (a) POM image of a polydomain CNC film showing a “striped” domain. (b) Representation of the simulation geometry, with two vertically aligned helicoidal domains (yellow and blue), both 1-μm thick with pitch *p* = 370 nm, with a discontinuous director at the boundary (magenta). (c) Variation in the director phase mismatch with distance along the domain boundary. (d) A hyperspectral scan simulated using PyLlama^583^ for the geometry in (b, c), assuming realistic optical properties for CNC photonic films (*n* = 1.555, Δ*n* = 0.062). Spectra are shown as a LCP reflectance heatmap versus distance and wavelength. Horizontal bar under the distance axis indicates the RGB color corresponding to each position, showing periodic green–red stripes. (e) Reflectance spectra from the heatmap in (d) for selected values of the director mismatch. Red lines indicate boundaries of the photonic stopband expected for a CNC film with *p* = 370 nm. Black dotted line indicates average over all director mismatch values. Adapted with permission from ref (345) under CC-BY. Copyright 2022 The Authors.

A recent study used this approach to simulate the optical response of a polydomain CNC film using both *Berreman 4 × 4* and *PyLlama* methods, and found the simulation results to be in good agreement with experimental hyperspectral imaging data and POM images.^345^ POM images often show stripey pattern in reflection, as illustrated by the detail shown in Figure 55a. To model a similar optical effect, a two-domain stack was simulated by introducing a defect as shown in Figure 55b and c, assuming a periodic variation in the phase mismatch at the domain boundary. The simulated spectra exhibited periodic variation with lateral position (Figure 55d), which corresponded to a stripey pattern of red and green bands when the spectra were converted into RGB color values. It is notable that two domains of equal pitch (which should ostensibly reflect in a single wavelength range) can lead to strikingly different colors (i.e., red or green) depending on their phase mismatch. Notably, while the reflection spectra of these domains are locally peaked, the integrated peak over the stripey region contributes to give an effective broader peak (Figure 55e). However, this broadening effect no longer occurs when thicker monodomains (e.g., with a thickness 20 μm each) are stacked onto one another.^345^

It must be stressed that the periodic “fingerprint” pattern seen in Figure 55a and in other POM images of CNC films does not correspond to the local pitch in the films, as it is sometimes reported. The agreement between observations and simulations in Figure 55 only confirms this distinction, as the helicoidal domains are near-vertical in alignment, and the periodic pattern arises instead from interference between the domains due to the phase mismatch at the domain boundary.

### 7.7 Summary

The optical properties of CNC films arise from the intrinsic optical properties of CNCs (most notably their birefringence) and their microscale cholesteric ordering. This complex and optically anisotropic structures leads to structural coloration in the visible range, which can be understood in terms of classical electromagnetism for periodic structures. A range of relevant and complementary optical tools were described to characterize these systems. Despite good conceptual understanding of the optical effects involved, accurately simulating the complex optical response of real samples remains a considerable challenge.

Although some advanced techniques were already used for optical characterization of CNC films and suspensions, there is scope for their broader implementation. For example, PolScope and Mueller matrix analysis provide valuable information about the structure of the samples that is not readily available with standard POM. Hyperspectral imaging can also be combined to Mueller matrix analysis, and can often come with a compromise on the spatial or temporal resolution (i.e, causing pixelization or slow acquisition time). Angle-resolved optical spectroscopy is another important technique that is not used by many research groups but provides invaluable insight into the local light-diffracting structures inside the sample.

## 8 Controlling CNC Self-Organization in Suspension

The self-organization of CNCs into a cholesteric mesophase is determined by the characteristics of individual particles (i.e., morphology, surface chemistry) and their formulation into a colloidal suspension (i.e., solvent, additives). These properties, in turn, result from the specific experimental parameters chosen during the multistage process of preparing a CNC suspension from cellulose biomass. This section discusses how the cholesteric behavior of CNC suspensions can be tuned by varying these parameters, considering each stage of the preparation process in turn. The subsequent impact of these parameters on the visual appearance of the resulting photonic films, while strongly influenced by the thermodynamic behavior in suspension, also depends on kinetic processes that are determined by the casting conditions and the onset of kinetic arrest. The role of experimental parameters on these kinetics processes will be discussed in section 9.

**Figure 56:**
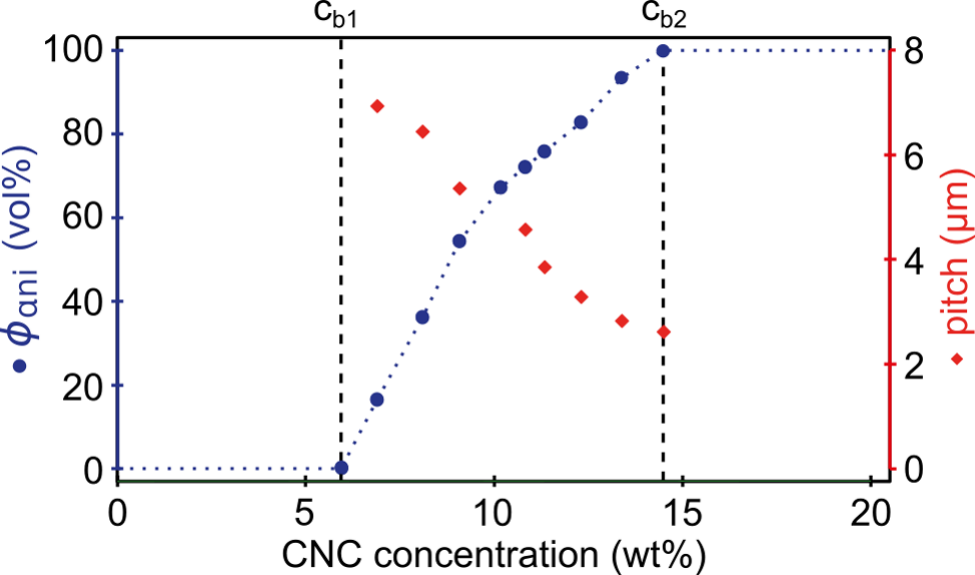
Example of a CNC phase diagram showing the anisotropic phase volume fraction (ϕ_ani_) versus CNC concentration (blue circles), and concentration-dependent cholesteric pitch (red diamonds). Data from ref (288).

The cholesteric self-organization of CNCs is generally quantified by preparing suspensions with a range of experimental parameters (typically varying CNC concentration but also other parameters such as ionic strength) and observing their equilibrium phase behavior (introduced in section 3.2.5). As shown in Figure 56, the relative fraction of the anisotropic (cholesteric) phase, ϕ_ani_, increases gradually above a threshold CNC concentration *c*_*b*1_ until a fully cholesteric phase is reached at a second threshold *c*_*b*2_. The equilibrium cholesteric behavior of the suspension is therefore captured, in essence, by the boundaries of the biphasic region (*c*_*b*1_ and *c*_*b*2_), and the concentration-dependent cholesteric pitch, *p* (Figure 56). As discussed in section 3.2, the boundaries of the biphasic range are primarily determined by the effective aspect ratio of the particles in suspension and their polydispersity. In contrast, the pitch depends on a wide range of properties of the CNCs and their formulation.

Elucidating the effect of a particular parameter on the collective behavior of CNC suspensions is challenging, as a single given parameter (e.g., degree of ultrasonication) often modifies several suspension properties simultaneously (e.g., particle morphology, ionic strength), which can each influence the cholesteric mesophase in different ways. Furthermore, quantitative comparison between previous studies is difficult, as the suspensions used often differ in terms of multiple key properties. Nevertheless, it is possible to gain insight into the main effects of each parameter and their relative importance.

### 8.1 Isolation of CNCs

CNCs can be produced from many different cellulose sources by a range of methods, as described in section 2.2 and reviewed elsewhere.^23,39,91,92,162^ This section discusses the impact of cellulose source, hydrolysis conditions and other key parameters surrounding CNC production on the cholesteric behavior of the resultant CNCs. Focus is given to sulfuric acid hydrolysis of wood pulp and cotton, as this production method is currently the most widely employed for photonic materials.

#### 8.1.1 Cellulose Source

**Figure 57:**
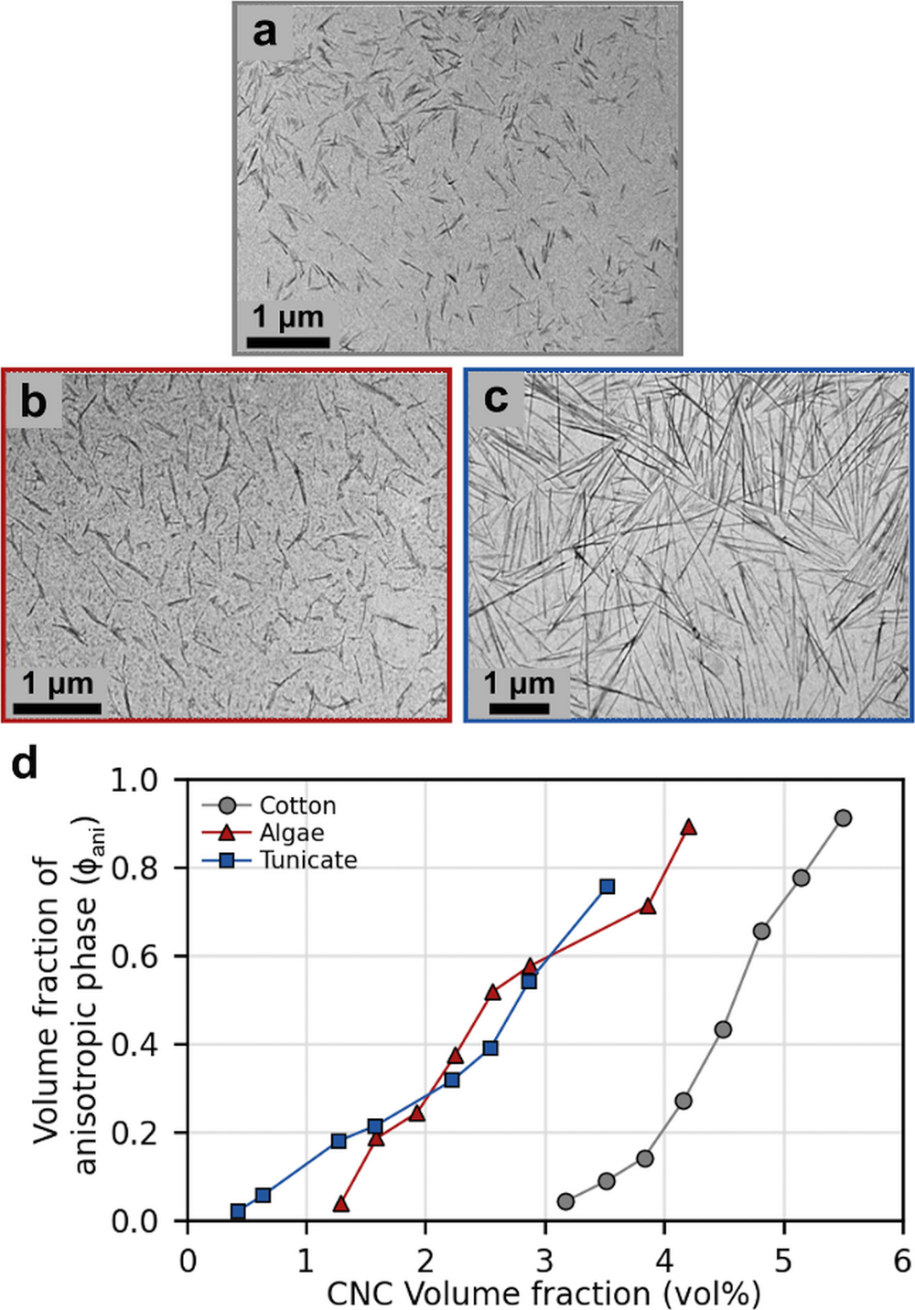
(a–c) TEM image and (d) phase diagram of CNCs from (a) cotton, (b) algae, and (c) tunicate under identical hydrolysis conditions (60 wt % H_2_SO_4_, acid/cellulose ratio 50 mL/g, 50 °C, 16 h). Reproduced with permission from ref (106). Copyright 2017 Wiley Periodicals, Inc.

The choice of cellulose biomass determines the maximum dimensions for the length and cross-section of the crystallites that compose the resulting CNCs, as these morphological properties are inherited from the original microfibrils (section 2.1). As a consequence, the mean aspect ratio varies considerably between CNCs from different sources, ranging from typical aspect ratios of 5–50 reported for wood and cotton CNCs to values of 50–150 for tunicate CNCs (section 2.2.2). As the boundaries of the biphasic region (*c*_*b*1_ and *c*_*b*2_) are expected to be inversely proportional to the particle aspect ratio, they are also expected to vary with the cellulose source. The role of cellulose source can be investigated directly by producing CNCs from multiple origins using identical hydrolysis conditions. For example, a comparison of CNCs from three sources showed that increasing aspect ratio (from cotton to algae to tunicate CNCs) was correlated with an earlier onset of the biphasic regime (i.e., *c*_*b*1_), as shown in Figure 57. However, due to differences in biomass composition, each source was prepared by a unique pretreatment and extraction protocol, which is also expected to affect the resulting particle morphology (see section 8.1.3).

Owing to their morphological similarities, CNC from wood and cotton display comparable self-assembly properties when subjected to identical hydrolysis conditions. A direct comparison reported that cotton-derived CNCs were longer than those derived from dried softwood pulp, but with a lower aspect ratio, consistent with the differences in source microfibrils (Figure 4). Consequently, the cotton-derived CNCs exhibited lower ϕ_ani_ at fixed concentration.^95^ In general, lignocellulosic CNCs exhibit a critical mass fraction for phase separation (*c*_*b*1_) of about 3–6 wt %, with a denser cholesteric phase having a typical pitch of 2 to 30 μm depending on the concentration.^94,268,284,290,292,306,308^ Increasing the mass fraction further leads to the formation of a monophasic cholesteric phase (*c*_*b*2_) typically from 6 to 12 wt %.

Using cellulose from algae typically leads to slightly bigger CNCs displaying higher aspect ratios, as described in section 2.2.2. While their behavior in suspension has not been studied as extensively as cotton and wood CNCs, the biphasic regime is shifted toward lower critical mass fractions compared to wood and cotton CNCs, consistent with their higher aspect ratio. As exemplified in Figure 57, they typically display a critical mass fraction for phase separation of about *c*_*b*1_ ≈ 1–2 wt % and the formation of a monophasic cholesteric phase above *c*_*b*2_ ≈ 7–8 wt %, with a pitch on the order of 20 μm.^106,109^

Cellulose from tunicate or bacteria typically yields CNCs with bigger aspect ratios than wood, cotton of algal CNCs while displaying similar surface charge per surface area (see section 2.3.2).^268,585,586^ As expected, CNCs from both sources exhibit lower critical mass fraction for phase separation c_*b*1_ < 1 wt %, but also reach kinetic arrest earlier, at around 3 wt %.^88,104,501,587^ Within the concentration range of cholesteric ordering, these CNCs generally have larger reported pitch values with 15–20 μm and >30 μm for bacteria and tunicate, respectively. Interestingly, in the coexistence domain of tunicate CNCs, a three-phase equilibrium has been reported in two studies, with a bottom cholesteric phase and a top isotropic phase separated by an additional anisotropic phase.^113,268^ This uncommon behavior is expected for high-polydispersity rod-like particles (as discussed in section 3.2.4) and was not exhibited by shorter and more charged tunicate CNCs.^268^ As a final comment, colored films from sisal,^98^ sugar cane bagasse,^97^ bacteria,^565^ and tunicate^588^ CNCs have been demonstrated, proving their relevance for such applications.

#### 8.1.2 Hydrolysis Conditions

The key parameters in sulfuric acid hydrolysis are the acid concentration, the acid/cellulose ratio, the temperature, and the reaction duration. By tuning these conditions, both the morphology and the surface chemistry of the resultant CNCs can be modified, with implications for their self-organization into a cholesteric mesophase. While numerous studies have investigated the influence of sulfuric acid hydrolysis conditions on CNC production,^88,93,102,158,159,589−596^ the primary aim of these studies was often to improve the yield rather than to optimize CNC properties relevant for their self-organization, such as morphology and surface charge. Nevertheless, these studies provide insight into the relationship between hydrolysis parameters and the properties of individual CNCs, which can be used to infer the consequences for CNC colloidal behavior and self-assembly. These trends are summarized in Table 2.

**Table 2:** Trends in CNC Individual Properties and Their Collective Behavior upon Varying Hydrolysis Parameters from Standard Conditions ∼50 °C Temperature, ∼60 min Time, ∼58-64 wt % Acid Concentration, and ∼8 mL/g Acid/Cellulose Ratio

		Consequence		
Parameter	Mechanism	Individual CNC	Collective Behavior	Ref
↓ Temperature	↑ cellulose solubility	↑ oligosaccharide redeposition on CNC surface after quenching	↑ stability toward ionic strength	(157)
	↓ hydrolysis of oligosaccharide chains		↑ impact of ultrasonication on pitch	
↑ Temperature	↑ hydrolysis at CNC ends	Broader distribution of lengths	↑ Δ*c*_*b*_ = *c*_*b*2_ – *c*_*b*1_ (broader range of biphasic regime)^a^	(88)
	↑ sulfate half-esters grafting	↑ surface charge	↑ pitch^a^	(88, 93, 159)
			↓ critical mass fractions^a^	
↑ Hydrolysis duration	↑ extent of hydrolysis	↓ dimensions	↑ critical mass fractions^a^	(93, 94, 130, 596)
		Narrower size distributions	↓ Δ*c*_*b*_ (narrower range of biphasic regime)^a^	
	Equilibration of sulfate half-esters grafting	↑ surface charge before plateauing	↓ critical mass fractions before reaching a plateau^a^	
↓ Acid concentration	↓ hydrolysis harshness	↓ yield	-	(93, 158, 159, 592, 594)
		↓ crystallinity	-	
		↑ dimensions	↓ critical mass fractions^a^	
		Broader size distributions	↑ Δ*c*_*b*_ (broader range of biphasic regime)^a^	
↑ Acid concentration	↑ hydrolysis harshness and cellulose dissolution	↓ yield	-	
		Narrower size distributions	↓ Δ*c*_*b*_ (narrower range of biphasic regime)^a^	
		↓ dimensions	↑ critical mass fractions^a^	
	↑ sulfate half-esters grafting	↑ surface charge	↑ pitch^a^	
			↓ critical mass fractions^a^	
↑ Acid/cellulose ratio	↑ sulfate half-esters grafting	↑ surface charge	↑ pitch^a^	(94, 290, 354)
			↓ critical mass fractions^a^	

a Expected effect that has not been experimentally studied or explicitly stated in the source.

**Figure 58:**
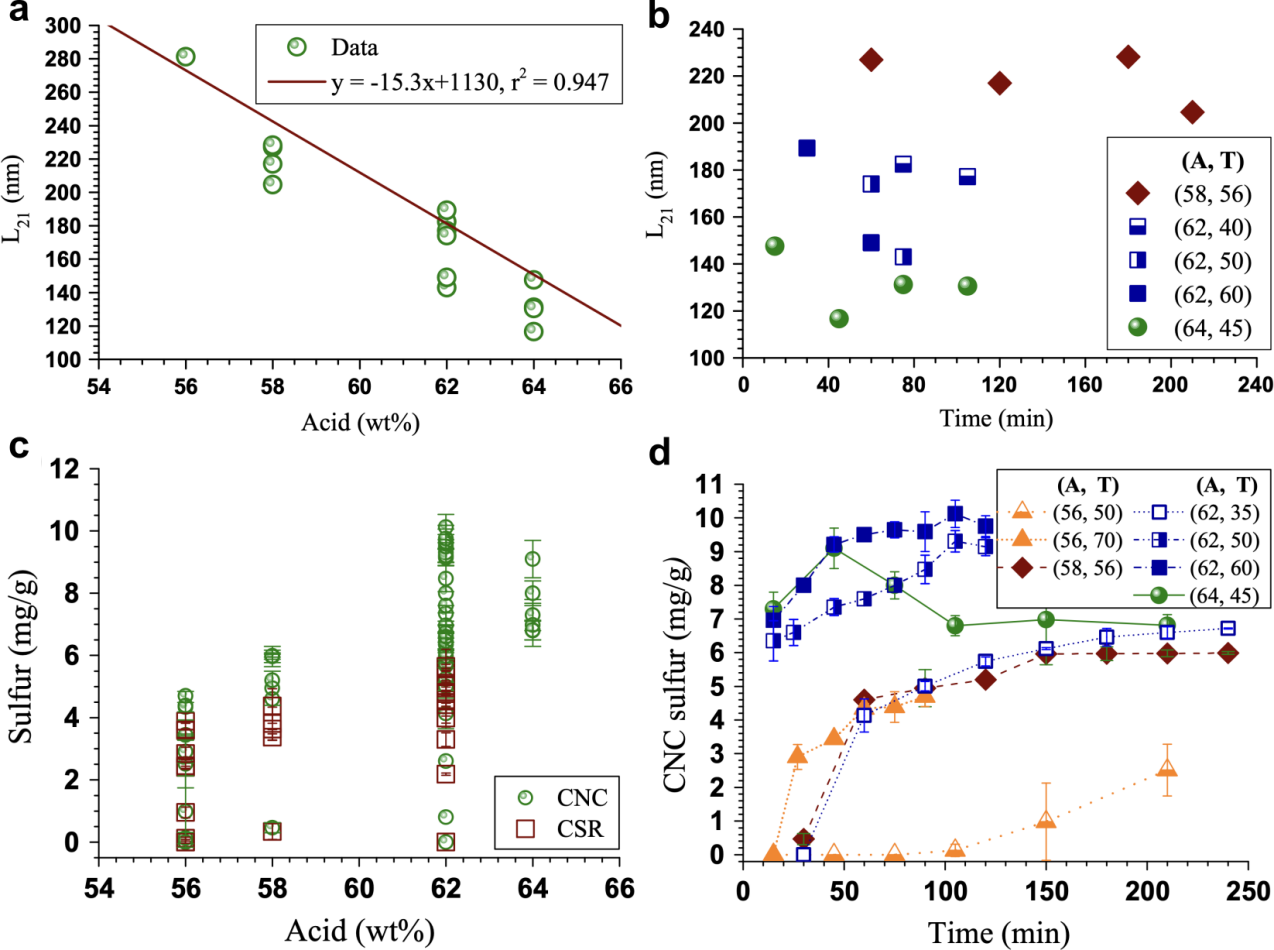
Effect of hydrolysis acid concentration (labeled in the legend as A, in wt%), duration (labeled as Time, in minutes) and temperature (labeled in the legend as T, in °C) on (a, b) the length-weighted mean length of CNCs and (c, d) sulfur content. CSR: cellulosic solid residue, obtained from precipitated partially hydrolyzed cellulose. Reproduced with permission from ref (93). Copyright 2015 Springer Nature.

In general, more intensive hydrolysis conditions (whether in terms of higher temperature, higher acid concentration, or longer duration) lead to shorter and thinner CNCs having lower polydispersity in length and width (Figure 58a,b).^93,94,130,596^ The CNC surface charge increases with both acid concentration (in the range 50–65 wt %)^93,159^ and reaction temperature (in range 40–72 °C),^93,159,268^ as these trends favor the esterification of alcohols in sulfuric acid, as discussed further in section 8.2.1 (Figure 58c,d).^597^ The surface charge typically increases rapidly at the beginning of the hydrolysis before reaching a plateau of around 200–400 mmol/kg, as reported for both wood and cotton CNCs.^93,159,596^ Finally, increasing the acid/cellulose ratio has been shown to substantially increase the surface charge,^290,354,585^ simultaneously, the CNC average length has been reported to slightly decrease,^94^ although some studies considered this decrease as negligible.^290,354^

**Figure 59:**
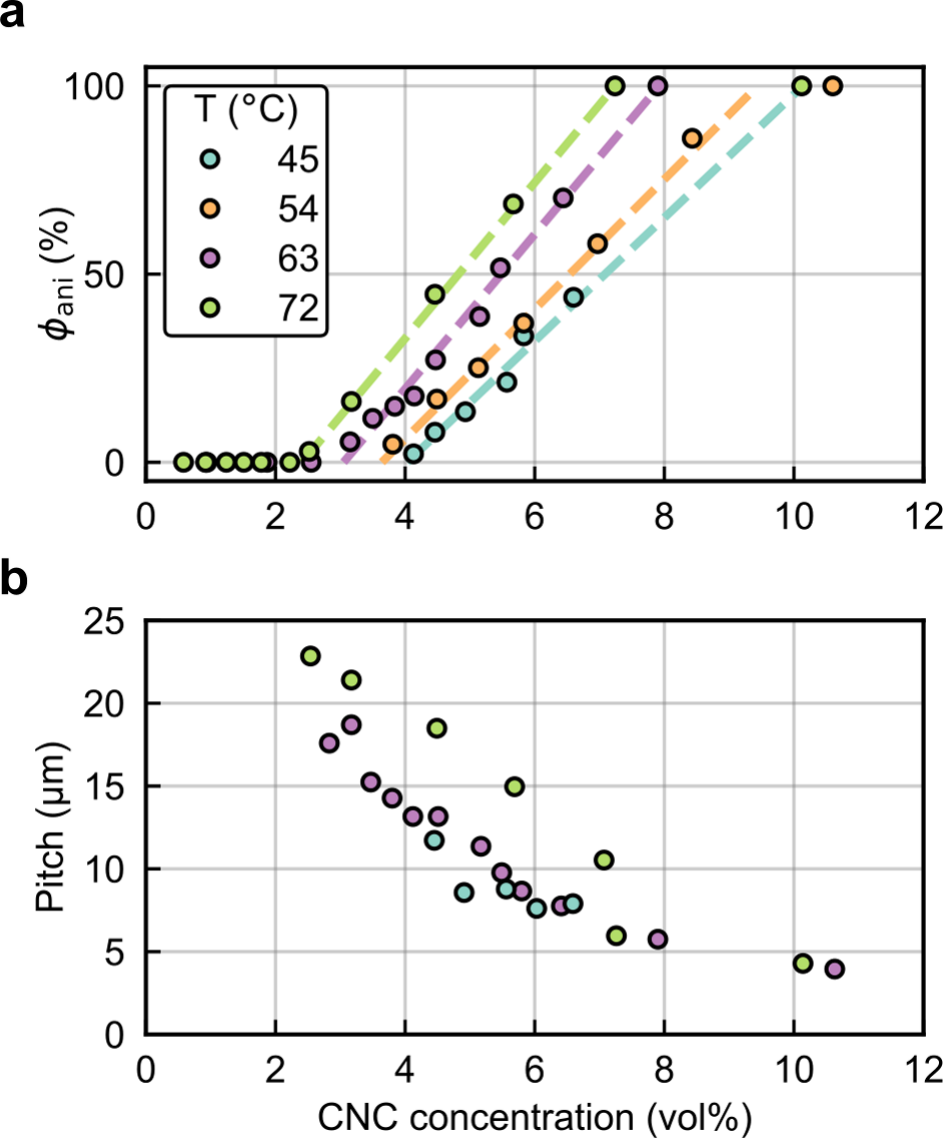
Effect of increasing hydrolysis temperature from 45 to 72 °C, while keeping the acid concentration and reaction duration fixed, on (a) the volume fraction of anisotropic phase (ϕ_ani_) and (b) the pitch of suspensions of CNCs from cotton. Data from ref (268).

The trends in morphology and surface charge with increasing hydrolysis intensity are expected to affect CNC self-organization in antagonistic ways, which may account for the conflicting observations when the literature is considered as a whole. Furthermore, relatively few studies have rigorously investigated the impact of hydrolysis conditions on CNC morphology, surface charge, and cholesteric self-organization. A study on the effect of hydrolysis temperature (45 to 72 °C) while keeping other hydrolysis conditions fixed (65 wt % sulfuric acid, 30 min duration, acid/cellulose ratio 12 mL/g) showed that increasing temperature led to a decrease in the first critical mass fraction (*c*_*b*1_) and a slight increase of the pitch (Figure 59).^268^ These trends suggest that the effect of increasing surface charge with temperature (i.e., greater repulsion between CNCs) dominates over any morphological changes.

When considering the effect of the duration of hydrolysis on the self-assembly behavior, it has been reported that increasing the hydrolysis time at fixed temperature (45 °C) and acid concentration (64 wt %) resulted in a decrease in *c*_*b*1_, before reaching a plateau after 60 min, indicating that the change in surface charge dominates.^596^ However, for the same hydrolysis conditions, increasing hydrolysis time from 25 to 45 min also led to the opposite effect, accompanied by a pitch reduction from 18 to 10 μm.^94^

Similarly, while increasing the surface charge through increasing the acid/cellulose ratio is expected to lower *c*_*b*1_ and reduce the size of the biphasic range, the opposite effect was actually observed in two different studies with few data points.^94,290^

Note that alongside surface charge, the hydrolysis temperature also determines the degree of oligosaccharide deposition on the CNC surface.^157^ For hydrolysis in 64 wt % sulfuric acid solution, a reduction in reaction temperature from 65 to 45 °C was found to slightly decrease the CNC surface charge (from 280 to 240 mmol/kg) with a negligible effect on mean hydrodynamic diameter. However, the lower-temperature hydrolysis led to greater production of oligosaccharides (degree of polymerization of 7 to 20 glucose residues), which precipitated onto the CNC surface when the reaction was quenched by dilution. When the cholesteric behavior of these suspensions was investigated indirectly by examining the reflection from helicoidal films, it was found that the red-shifting effect of ultrasonication (discussed in section 8.2.2) was more pronounced for CNCs with greater oligosaccharide deposition.

#### 8.1.3 Other Parameters of the Isolation Process

Besides the intrinsic properties of a given cellulose source and the hydrolysis parameters, the characteristics of the CNCs are also influenced by other factors involved in their isolation. As presented in section 2, cellulose can be pretreated in various ways to produce CNCs with high yield and purity. Moreover, several posthydrolysis treatments are often applied to CNCs to purify them or adapt their properties for the intended use. These treatments are known to modify the material composition, structure and/or interactions, yet their impacts on CNC characteristics or their subsequent self-assembly remained largely overlooked.

Drying of the raw material, even if rehydrated afterward, can have irreversible effects on the cellulose fibers with striking consequences on the colloidal properties of the CNCs extracted from it. For example, CNCs from dried algal cellulose appear to have a shorter average length and narrower size distribution of length than those from never dried algal cellulose, with 302 ± 113 and 513 ± 317 nm, respectively (given as mean ± standard deviation).^199^

**Figure 60:**
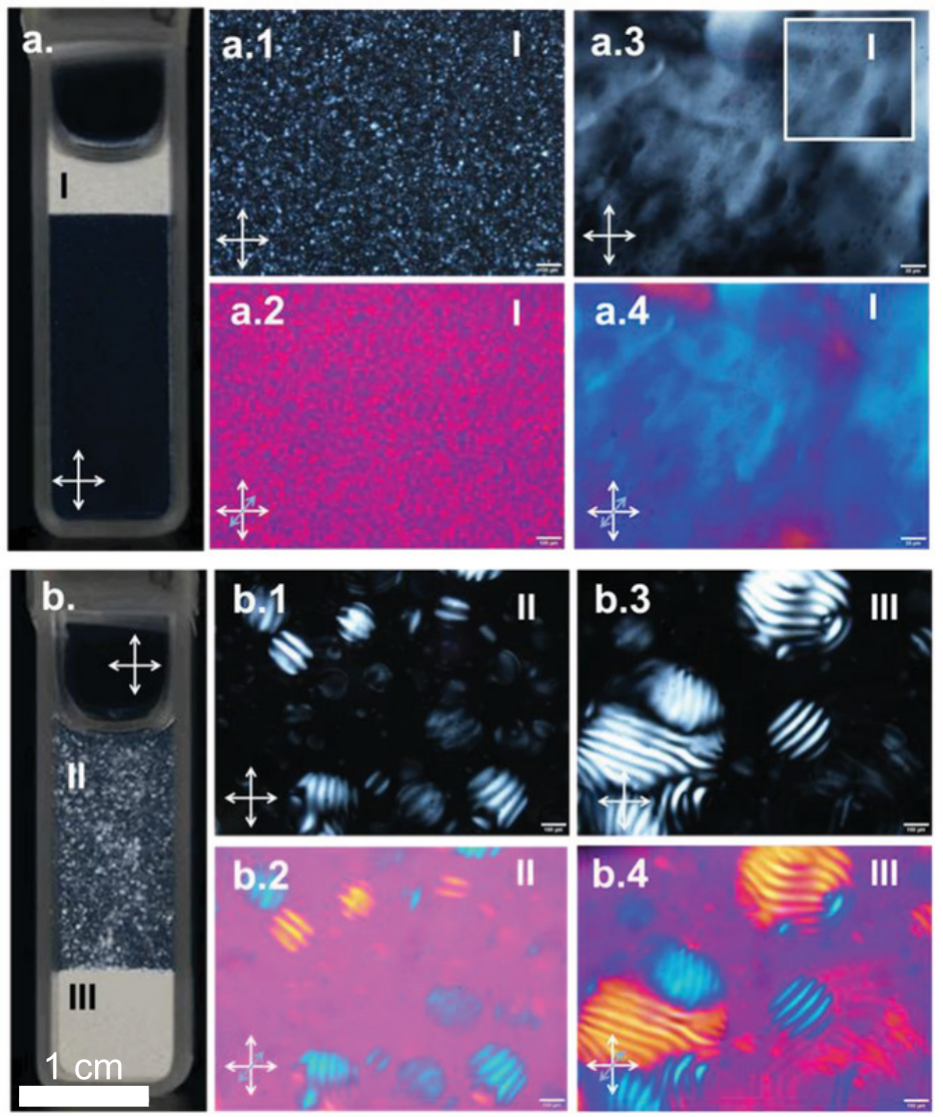
Phase separation of CNCs suspensions prepared from (a) never dried or (b) dried eucalyptus pulp and purified with a freeze–thawing step, as visualized through cross polarized light. Transmission microscope images of selected phases (I–III) confined in flat capillaries (a1, a.2, b.1-b.4) or as a droplet on a microscope slide (a.3, a.4) between cross polarizers (a.1, a.3, b.1, b.3) or between crossed polarizers with a lambda plate (a.2, a.4, b.2, b.4). Reproduced from ref (289) under CC-BY. Copyright 2022 The Authors.

The importance of drying have also been illustrated by the recent observation of a low-density CNC nematic phase (Figure 60).^289^ This behavior was only exhibited by CNCs isolated from never dried eucalyptus pulp combined with a freeze–thawing step after hydrolysis. The authors hypothesized that by reducing water exclusion, the presence of air led to a change in cellulose conformation eventually leading to a low-density phase.

As mentioned in section 2.2.3, mercerization (strong alkaline treatment) is commonly used in the textile industry to improve the mechanical properties of native cellulose fibers. Given the potential use of waste textiles as a feedstock for CNC production, the effect of mercerization on particle morphology and cholesteric ordering is therefore worthy of consideration. CNCs made from cellulose pretreated by mercerization (CNC-II) have been reported to be significantly shorter than CNCs made from the same cellulose source without pretreatment (CNC-I).^73,145,598^ The critical mass fraction for phase separation was also found to be higher for CNC-II suspensions (*c*_*b*1_ ≤ 9 wt %), with a much longer time required for self-assembly (approximately 40 weeks) and a larger equilibrium pitch (54 μm at 9 wt %).^73^

As a last example, quenching has been shown to lead to the precipitation of oligosaccharides on the CNC surfaces when they are present in solution.^157^ Interestingly, this phenomenon can be exploited to tune surface properties by adding oligosaccharides to the quenching medium.^599^ Moreover, while Soxhlet extraction have been showed to improve CNC surface reactivity and reproducibility of surface modifications after hydrolysis of cotton at 45 °C, this finding is probably related to the oligosaccharide deposition onto the CNC surface in these hydrolysis conditions.^600^

### 8.2 Post-Processing the CNCs

After the initial production of CNCs, their morphology and surface chemistry can be modified by postprocessing the suspension. This section presents a summary of the techniques available to tune cholesteric behavior by modifying the individual characteristics of the CNCs, as summarized in Table 3.

**Table 3:** Techniques to Modify the Properties of Individual CNCs and the Consequences for Self-Assembly

Technique	Principle/mechanism	Consequence on CNCs	Consequences on suspension and self-assembly	Ref
Heat induced desulfation	Substitution of surface sulfate half-esters by alcohol	↓ sulfate half-ester content	↑ ionic strength	(601−605)
			↓ pH	
		↓ surface charge	↓ stability	
			↑ critical mass fractions	
			↓ pitch^a^	
Surface sulfation	Esterification of surface alcohols by sulfuric acid	↑ sulfate half-ester content	↑ stability	(216, 501, 601, 602, 606−608)
			↓ critical mass fractions^a^	-
		↑ surface charge	↑ pitch^a^	-
Surface oxidation	Carboxylation of surface alcohols	↑ carboxylic acid content	↑ stability	(174, 609)
		↑ surface charge	Improve self-assembly	
			↓ critical mass fractions	(306)
Surface cationization	Replacement of surface sulfate half-esters by HPTMAC	↑ HPTMAC content	Good colloidal stability	(610)
		Surface charge cationization		
		↓ surface charge		
Polymer grafting	Replacement of surface charges by grafted polymer chains	↑ steric stabilization	↑ stability toward ionic strength	(174)
		↓ surface charge	↓ pitch	(174, 306, 611)
	Addition of thermoresponsive behavior	New thermoresponsive functionality	thermoreversible pitch modification	(306, 611, 612)
Polymer adsorption (no surface charge loss)	Adsorption of dense coating (poly domapine)	↑ particle width, thickness	↑ pitch	(274)
		↓ aspect ratio	↑ critical mass fractions	
		↓ cross-sectional aspect ratio		
	Adsorption of polymer (xyloglucans)	↑ particle width, thickness	↑ pitch	(276)
		↓ aspect ratio	↑ critical mass fractions (nonmonotonous at higher XG:CNC ratios)	
		↓ cross-sectional aspect ratio	CNC fractionnation (accumulation of coated CNCs into the isotropic phase)	
		↑ fractionation		
Surfactant coating	Surface coating and redispersion in organic solvent	↑ surface apolarity	Cholesteric LC in cyclohexane or toluene	(315)
Ultrasonication	CNC lateral dissociation	↓ size parameters	↑ pitch	(89, 314, 613)
	Ion liberation	-	↑ conductivity	(314)
			↓ pitch^a^	-
	Cellulose chain deposition on CNC surface	↓ crystalline index	-	(614)
Centrifugation fractionation	Selection by CNC volume through centrifugal forces	Narrower size distribution	↑ critical mass fractions^a^	(284, 615)
		↓ CNC average length	↓ Δ*c*_*b*_ = *c*_*b*2_ – *c*_*b*1_ (narrower range of biphasic regime^a^	
		↑ aggregation?		
Phase separation fractionation	Selection of CNCs in the isotropic phase	↓ CNC average length	↑ critical mass fractions	(284)
		Narrower size distribution	↓ Δ*c*_*b*_ (narrower range of biphasic regime	
	Selection of CNC in the anisotropic phase	↑ CNC average length	↓ critical mass fractions	
			↓ Δ*c*_*b*_ (narrower range of biphasic regime	

a Expected effect that has not been experimentally studied or explicitly stated in the source.

#### 8.2.1 Surface Modification

CNC surface chemistry dictates the interactions between particles and their local environment, and therefore influences their ability to self-organize. While numerous studies have demonstrated surface modification of CNCs for a range of applications,^616−618^ the potential for cholesteric self-organization was rarely explored, and consequently the relevance of these modified CNCs for photonic materials remains uncertain. However, it is possible to modify CNC electrostatic interactions (by changing their surface charge) or steric interactions (by polymer grafting) while preserving the ability to form a cholesteric mesophase.

##### 8.2.1.1 Desulfation

For sulfated CNCs, the maximum surface charge is determined by the hydrolysis conditions (section 8.1.2) but can decrease after hydrolysis due to desulfation. The labile sulfate half-ester groups can be converted into alcohols by the reverse reaction to the initial sulfation during hydrolysis, as shown in eq 135:^597^
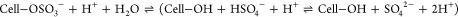
135

This desulfation process has an estimated activation energy of 26 kJ/mol assuming a first-order reaction,^605^ and can be catalyzed by acidic or basic conditions.^601,603−605,619^ Notably, in the case of protonated CNCs (H-CNCs) the reaction is self-catalyzing,^602^ and desulfation is therefore an unavoidable process that gradually decreases the CNC surface charge, increases the ionic strength and decreases of pH of the suspension (with the pH effect especially pronounced at values around p*K*_a__2_ = 2.0, where HSO_4_^–^ deprotonates). This effect becomes significant when comparing samples over the time scale of months, but can be reduced by storage of H-CNC suspensions at low concentration and temperature (i.e., refrigeration), or nearly suppressed by neutralization with a base to lead to the formation of the corresponding CNC salt (e.g., NaOH to produce Na-CNCs). As a quantitative example, storing a 5.5 wt % H-CNC suspension for 308 days at 23 °C lowered its surface charge from 240 mmol/kg to 193 mmol/kg (−19.8%), whereas an equivalent sample stored at 4 °C had a final surface charge of 221 mmol/kg (−8.1%).^602^ However, for a 5.0 wt % Na-CNC suspensions stored under the same conditions, desulfation was greatly reduced (i.e., less than 4% per year if refrigerated). Na-CNCs have the secondary advantage of permitting redispersion of CNCs in the form of a freeze-dried powder, a convenient option chosen to facilitate storage and/or shipment.^601,620^

**Figure 61:**
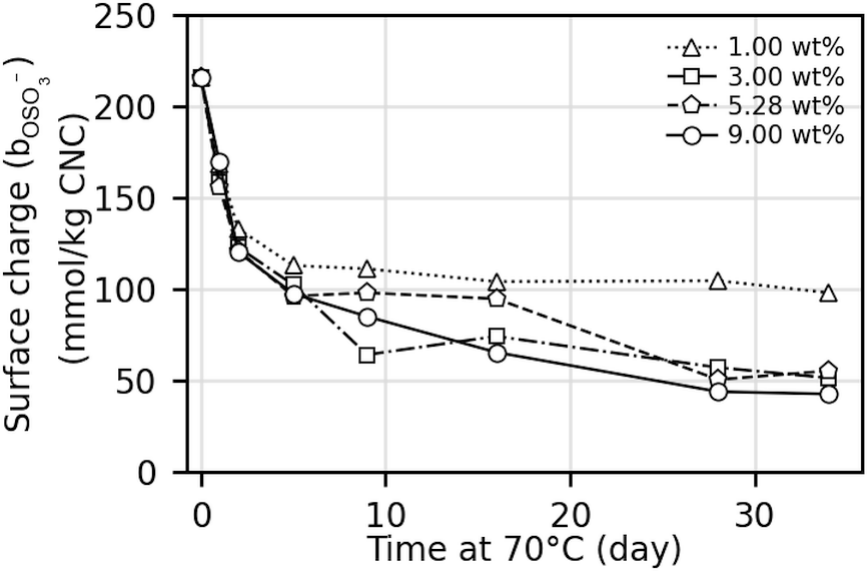
Evolution of sulfur content (mmol/kg) with time for CNC suspensions with increasing mass fractions, heated at 70 °C. Data from ref (602).

Conversely, intentional desulfation by heating the suspension has also been investigated as a method to reduce the surface charge of sulfated CNCs. For example, heating a suspension of sulfated CNCs at 70 °C led to a rapid initial decrease in surface charge, before reaching a fixed value (Figure 61).^602^ This limiting surface charge was attributed to the surface proton density dropping below a critical value, which prevents further desulfation. Furthermore, the initial rate of desulfation is highly sensitive to temperature: for instance, H-CNCs lost 65% of their surface charge after either 3–5 days at 70 °C or only 2 h at 100 °C. The process is also enhanced by higher CNC mass fraction, explained by a higher probability of being triggered by an adjacent sulfate-half ester group. While heating can also lead to the removal of other common CNC surface groups (e.g., carboxyl groups, phosphates), sulfate half-esters are by far the most sensitive to this hydrothermal treatment.^167,605^ Mild hydrothermal desulfation can thus be used to tune cholesteric self-assembly (as discussed below), while extensive desulfation leads to CNCs with very low surface charge (<0.1 e/nm^2^) that, due to their amphiphilic nature, can be used as stabilizers for Pickering emulsions.^67,68,621^

As a final comment, desulfation of CNCs in basic suspensions (typically using NaOH) has also been demonstrated as a way to gradually tune the surface charge,^622^ or can be used as an initial step toward subsequent functionalization.^610,623^ Thermally induced desulfation can also be performed in other solvents: for example, pyridine-neutralized CNCs suspended in DMSO with methanol were significantly desulfated (from 293 to 7 mmol/kg) by thermal treatment for 2 h at 80 °C.^603^

##### 8.2.1.2 Impact of Desulfation on Self-Organization

As indicated by eq 135, the loss of sulfate half-ester groups leads to a lower CNC surface charge, and the liberated ions will increase the ionic strength of the suspension. Moderate desulfation will therefore reduce both the magnitude of electrostatic repulsion between CNCs and the range of their interactions (i.e., the Debye length), offering a complementary way to tune CNC interactions as an alternative to electrolyte addition.^288^ Both of these effects are expected to shift the biphasic region toward higher concentrations (i.e., increase *c*_*b*1_ and *c*_*b*2_), meaning that for a fixed CNC concentration, desulfation is expected to cause a decrease in the volume fraction of anisotropic phase (ϕ_ani_), as observed over time in H–CNC suspensions stored at room temperature (4 days vs 3 months).^288^ For cholesteric suspensions, the reduced repulsion between CNCs is also expected to lower the pitch (*p*).

**Figure 62:**
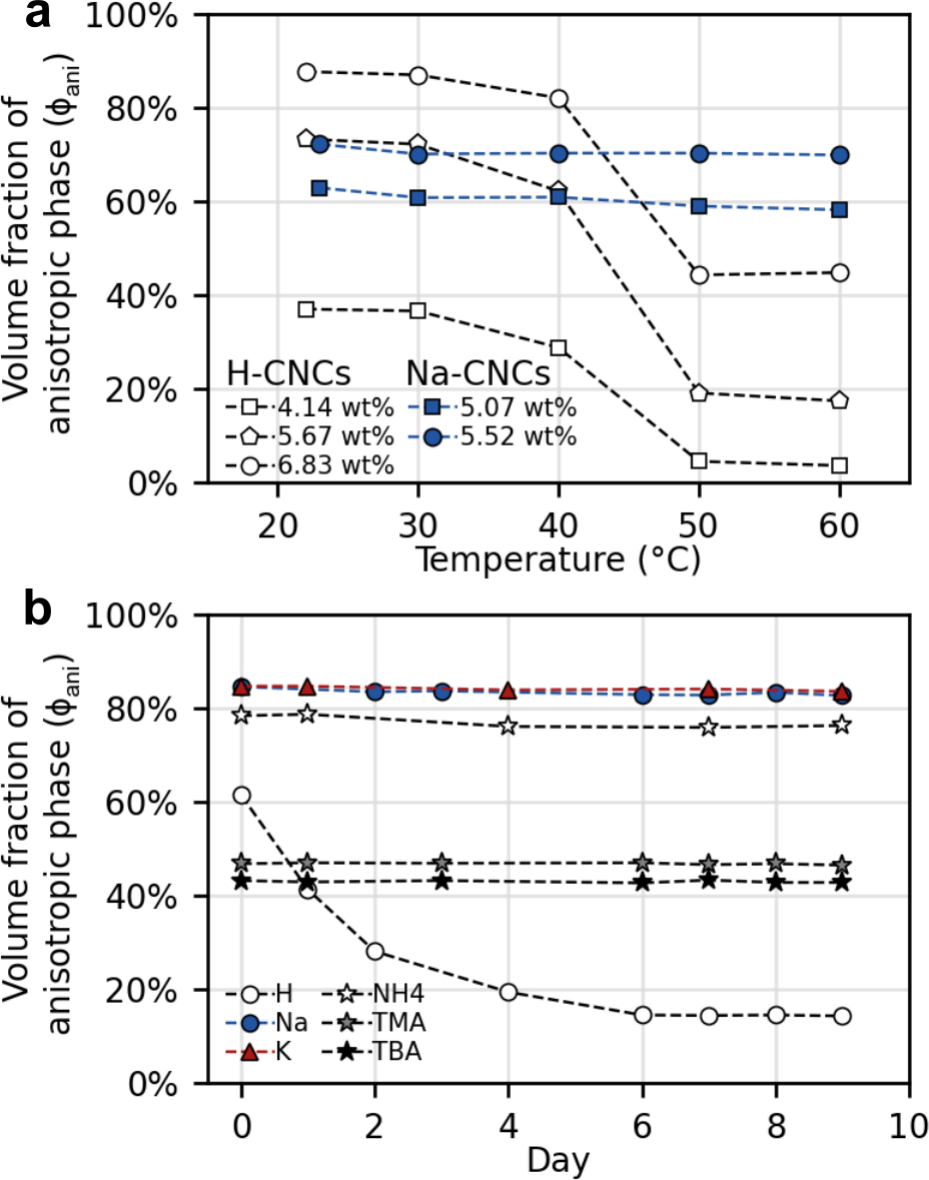
Evolution of the volume fraction of anisotropic phase (ϕ_ani_) of CNC suspensions (a) as a function of the storage temperature (3 days), the counterion (H or Na) and the mass fraction (wt%) and (b) as function of the number of days at 60 °C and of the counterion (H^+^, Na^+^, K^+^, NH_4_^+^, TMA^+^: tetramethylammonium or TBA^+^: tetra-n-butylammonium). Data from ref (601).

Experimentally, desulfation by hydrothermal treatment has been shown to reduce ϕ_ani_ (Figure 62a).^601,602^ When comparing CNC suspensions stored at various temperatures for 3 days, a dramatic decrease in ϕ_ani_ was observed above 40 °C for H–CNCs at all concentrations, whereas Na-CNCs were not strongly affected.^601^ When comparing CNCs with different counterions (Figure 62b), hydrothermal treatment for several days at 60 °C led to a gradual reduction in ϕ_ani_ for H–CNC suspensions, while no meaningful change was observed after 9 days for the other suspensions.

The effect of desulfation on the pitch in suspension has not been directly investigated, to the best of our knowledge. However, as discussed in section 4.3, a Helfrich-Hurault (HH) instability was reported for sealed capillaries of H–CNC suspension after one year at room temperature, with an enhanced effect after an additional week at 60 °C.^268^ The observation of a HH instability, which is triggered by an increase in the number of pitch repeats within a confined geometry, indirectly confirms that desulfation leads to a decrease in the pitch. Furthermore, desulfation has been reported to blue-shift the structural color of photonic CNC films, which also likely arises from a smaller pitch in suspension.^21,602,624,625^ However, excessive desulfation compromises the colloidal stability of the CNCs and leads to aggregation before cholesteric ordering can occur.^602,622^

##### 8.2.1.3 Other Surface Charge Modifications

The surface charge of sulfated CNCs can be modified in various ways, aside from desulfation, while retaining the ability to form a cholesteric phase. TEMPO oxidation, often used to stabilize CNCs produced by HCl hydrolysis, can also be applied to sulfated CNCs to increase their total surface charge without modifying their morphology, and has been reported to shift the biphasic region to lower concentrations (i.e., smaller *c*_*b*1_ and *c*_*b*2_).^306^ Alternatively, functionalization of sulfated CNCs using (2,3-epoxypropyl) trimethylammonium chloride (EPTMAC) was shown to produce colloidally stable suspensions with 112 mmol/kg of cationic surface charges.^610^ While these cationic CNCs did not exhibit liquid crystalline phase separation, concentrated gels exhibited birefringence, suggesting local nematic organization.

CNCs produced by hydrochloric acid (HCl) hydrolysis have low surface charge (<20 mmol/kg),^626^ which is attributed to a small number of carboxylic acid groups. Consequently, these CNCs have poor colloidal stability and do not exhibit cholesteric ordering.^596^ However, their surface charge can be increased through carboxylation of the surface via TEMPO oxidation (as discussed in section 2.3.2). The number of carboxylic acid groups per CNC mass is determined by the concentration of NaClO,^175^ which can lead to almost full conversion of accessible hydroxy groups (degree of oxidation, DO, of 0.1 for tunicate CNCs).^609,627^ Such TEMPO oxidation can increase the surface charge of wood or cotton CNCs to more than 1000 mmol/kg, depending on their morphology.^627^ Such carboxylated CNCs have been shown to exhibit cholesteric mesophases,^153,174,332^ suggesting they can be used to produce photonic films. Alternatively, CNCs produced using HCl can be posthydrolyzed with sulfuric acid, resulting in a coverage of sulfate half-ester groups comparable to standard sulfuric acid hydrolysis.^216^ A subsequent study demonstrated that such postsulfated CNCs also can form a cholesteric mesophase.^501^

##### 8.2.1.4 Polymer Grafting

The colloidal stability and cholesteric ordering of charged CNCs are sensitive to the ionic strength of the suspension (as discussed further in section 8.3.1). However, polymer grafting can be used to provide steric stabilization to CNCs, bringing their behavior closer to that of neutral rigid rods, while preserving the ability to form a cholesteric mesophase.^306,623^ This strategy can also lead to the emergence of new properties that extend the range of applications of CNCs.

The impact of steric stabilization through polymer grafting was first investigated by Araki et al.^174^ By grafting poly ethylene glycol (PEG) on to TEMPO oxidized CNCs, they were able to dramatically increase suspension stability at high ionic strength. While the initial CNCs aggregated in 0.5 M NaCl, the grafted samples were still stable at 2.0 M NaCl. PEG grafting did not inhibit liquid crystal phase formation, but did reduce the pitch from 14 to 8 μm (>5% solid content). Similar pitch reductions were observed by later studies on polymer grafted CNCs.^306,611,623^ These observations were explained by a lower interparticle distance caused by lower effective surface charge.

**Figure 63:**
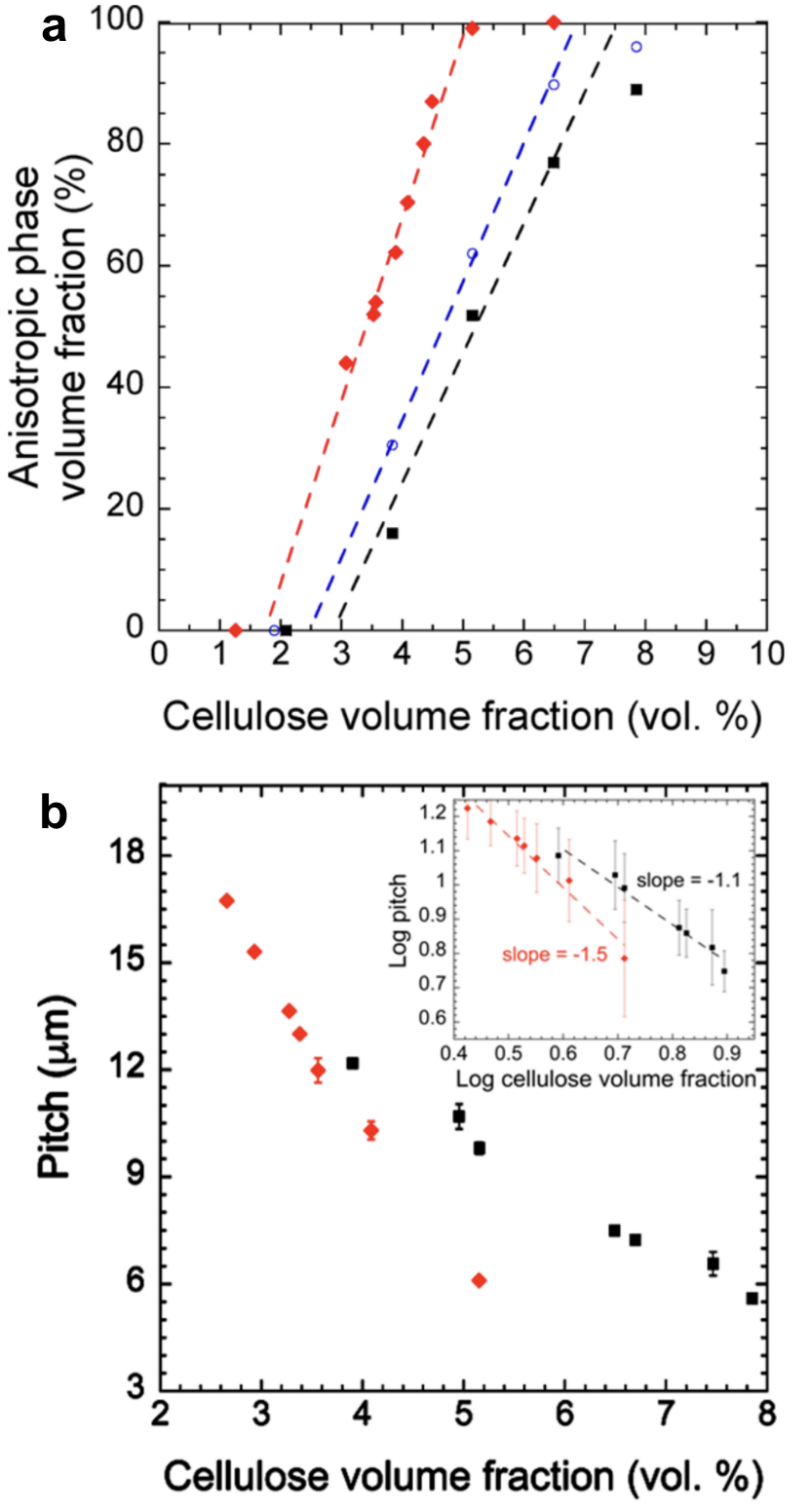
Evolution of (a) the anisotropic phase volume fraction and (b) pitch as a function of CNC volume fraction of sulfonated CNCs (black squares), TEMPO oxidized CNCs (blue circles) and polyetheramine-grafted CNCs (red diamonds) suspensions. Reproduced with permission from ref (306). Copyright 2016 American Chemical Society.

Polyetheramine (Jeffamine) can also be grafted onto carboxylated CNCs, providing steric stabilization, thermoresponsive aggregation behavior and modification of the cholesteric self-assembly.^612^ Grafting of polyetheramine shifted the biphasic region toward lower CNC volume fractions, which was explained by a greater effective volume of the CNCs due to electrosteric stabilization (Figure 63a).^306,312^ Consequently, their self-assembly behavior was closer to that of neutral rigid rods. However, at identical cellulose volume fraction, grafted samples displayed a lower pitch with a greater dependence on the CNC volume fraction as illustrated in Figure 63b. Moreover, increasing the ionic strength by NaCl addition (from 0 to 1 mM) in grafted suspension at 5.2 vol % of cellulose led to an unexpected increase in pitch from 6 to 7 μm. This peculiar observation could be related to the steric stabilization of these CNCs, which is also atypical in the literature. This invites a re-examination of the reasons for the pitch decrease when the ionic strength increases in the most conventional case with electrostatically stabilized CNCs.

Finally, CNC can be preferentially functionalized at their reducing end groups, but these modifications may come at the expense of cholesteric ordering.^628^ Recently, grafting of the reducing end groups in CNCs from cellulose I and cellulose II was performed while preserving their ability to self-assemble.^629^ Due to the chemical polarity of cellulose I and the centrosymmetry of cellulose II, the functionalization of the reducing end groups on CNC-I and CNC-II was referred to as asymmetric and symmetric grafting, respectively. However, it is unclear whether this selective grafting can lead to the grafting at only one tip of the CNCs, given their bundled nature (see discussion about chemical polarity of CNCs in section 2.3.1).

##### 8.2.1.5 Polymer Adsorption

Polymer adsorption onto the CNC surface differs from polymer grafting in a several key aspects: for instance, adsorption does not usually involve a chemical reaction, and may therefore modify the surface chemistry of the CNCs without reducing their total surface charge. In addition, a significant portion of the adsorbed polymer is expected to lie flat against the CNC surface, and so the polymer will not provide long-range steric repulsion, as would be expected from grafted polymer chains in good solvent. Studies on the cholesteric self-organization of CNCs with adsorbed polydopamine (whose structure is poorly understood),^274^ or xyloglucans (XG),^276^ have reported increases in the critical volume fraction (*c*_*b*1_) and cholesteric pitch after polymer adsorption. These trends are consistent with a reduction of the particle aspect ratio *a*_3*D*_ and cross-sectional aspect ratio *a*_*XS*_. In the case of XG-coated CNCs, additional microphase separation in the biphasic regime was observed, where XG-rich CNCs were preferentially expelled from the cholesteric regions.^276^

#### 8.2.2 Ultrasonication

Exposure to ultrasound (typically at 20 kHz) is a widely used technique to disperse aggregated nanoparticles or dried powders in suspension.^630^ Ultrasonication applied to CNC suspensions at high acoustic intensities can also be used to modify their cholesteric behavior by changing the morphology of individual particles and the properties of the colloidal suspension.

**Figure 64:**
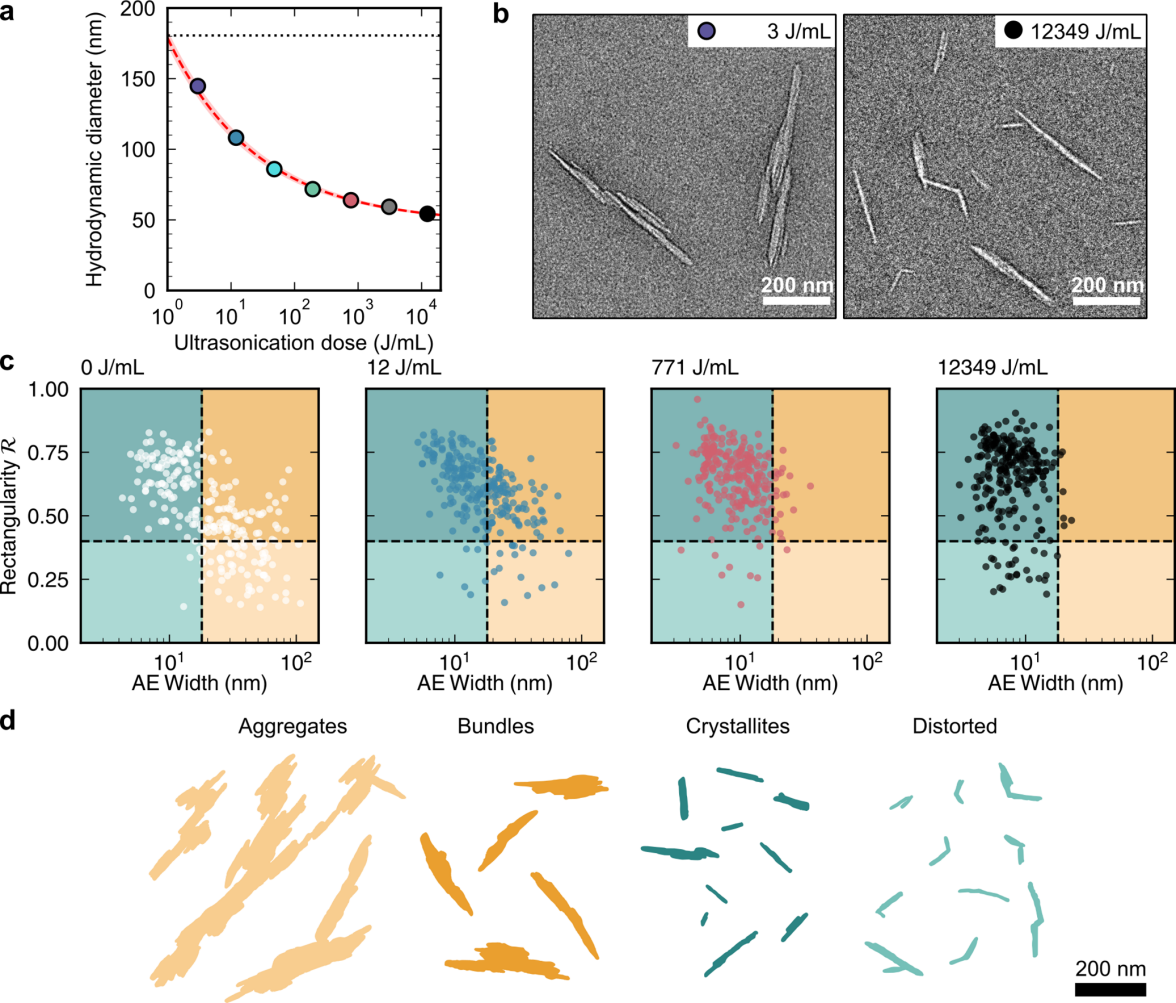
Influence of ultrasonication on CNC morphology. (a) Evolution of hydrodynamic diameter with ultrasonication dose. (b) TEM images of typical CNC morphology without (left) and with (right) significant ultrasonication dose. (c) Mapping of CNC rectangularity and area equivalent width (AE) as function of ultrasonication dose. (d) Scheme of the typical evolution of CNC morphology with increasing ultrasonication (from left to right). Adapted from ref (89) under CC-BY. Copyright 2022 The Authors.

Ultrasonication of CNCs leads to an overall decrease in the average size of individual particles,^87,314^ with negligible reduction in crystallinity.^200,631^ This effect can be observed in ensemble properties as a pronounced decrease in average hydrodynamic diameter, *D*_hyd_ (Figure 64a),^89,196,632^ and a visible decrease in suspension turbidity.^201^ Characterization of individual CNC morphology using TEM or AFM (see section 2.3.1) reveals that ultrasonication causes dissociation of composite particles, leading to a decrease in the average particle length, width and thickness (as exemplified in Figure 64b).^87,89,613^ In terms of CNC shape, ultrasonication leads to a decrease in the average 3D aspect ratio *a*_3D_, as evidenced by measurements of individual particles and from ensemble measurements,^89,613^ as well as a statistical decrease in the aspect ratio of the particle cross-section, *a*_*XS*_.^89^ As discussed in section 2, the term CNC is used throughout this review to refer to any colloidally stable nano-object in the suspension. This definition therefore encompasses both isolated cellulose crystallites and composite particles made of multiple crystallites aggregated together. Using this terminology, ultrasonication fragments individual crystallites from composite particles, increasing the total number of CNCs in suspension while reducing their average size. This mechanism is also consistent with the observed increase in the mean rectangularity of CNC outlines with ultrasonication (Figure 64c).^89^ Furthermore, by classifying individual particles according to their width and rectangularity, it is possible to separate CNCs into four subpopulations with distinct morphological characteristics (Figure 64d), ranging from large disordered *Aggregates* to laterally associated *Bundles* and isolated *Crystallites*. At very high ultrasonication doses, *Distorted* crystallites with kink defects are observed, which can be attributed to localized deformation due to bending.^631^ This kinking is more typically observed for high-aspect-ratio objects such as CNFs^136,332^ and crystallites of tunicate cellulose.^88^ Notably, for cellulose Iα microfibrils, such bending deformation has been reported to cause partial conversion to cellulose Iβ.^631^

Alongside the morphological changes described above, ultrasonication is accompanied by the release of ions into the suspension, leading to an increase in ionic conductivity and a decrease in pH.^89,314^ For acidic suspensions, ultrasonication-induced heating can lead to desulfation (see section 8.2.1) with an associated release of ions, if the heating is not mitigated (e.g., by immersing the sample vial in an ice bath). While an increase in ionic strength will impact the self-assembly (as discussed in section 8.3.1), excess ions can be removed by dialysis after ultrasonication, which allows the effects of particle morphology and suspension formulation to be disentangled.

**Figure 65:**
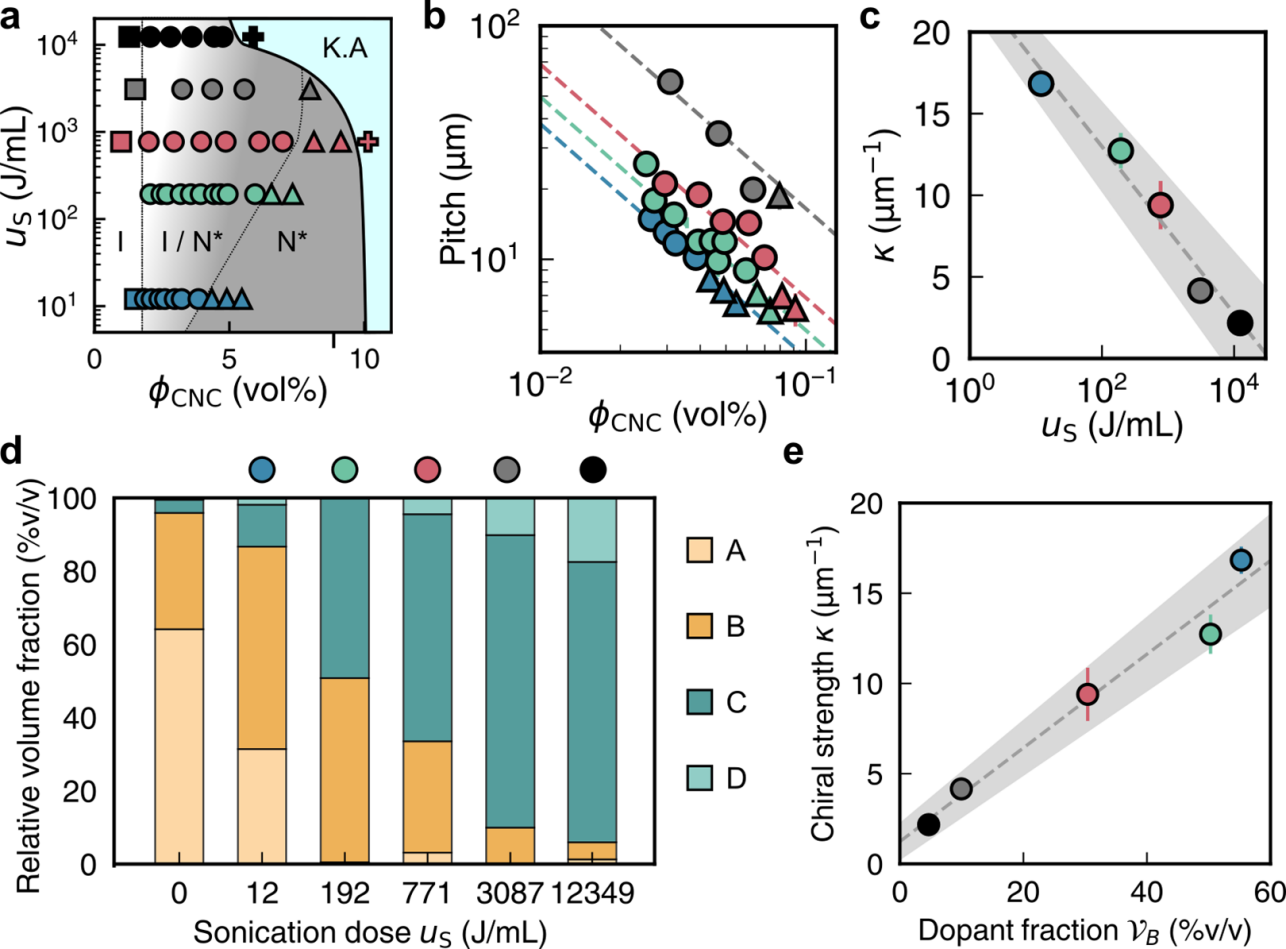
Impact of ultrasonication of CNC characteristics and resulting self-assembly behavior. (a) Phase diagram as function of ultrasonication dose (*u*_S_) and CNC volume fraction (ϕ_CNC_), with indication of the isotropic (I, squares), biphasic (I/N*, circles), cholesteric (N*, triangles), and kinetically arrested (K.A, crosses) regions. (b) Evolution of the cholesteric pitch as function of ϕ_CNC_ and *u*_S_ in the biphasic (circles) and cholesteric (triangles) regions. Dotted lines indicate power law fitting with *p* ∝ 1/ϕ_CNC_. (c) Chiral strength (κ) evolution as function of the ultrasonication dose (*u*_S_) with empirical fitting κ = κ_1_ – κ_2_ ln (*u*_S_). (d) Evolution of the relative proportion of different CNC morphologies (A: Aggregates, B: Bundles, C: Crystallites, and D: Distorted) with ultrasonication dose (*u*_S_). (e) Chiral strength (κ) evolution with the doping fraction of bundle-like objects (ν_B_). Error bars indicate standard deviation of 30 measurements, gray dotted line and shaded region indicate linear fitting and its corresponding uncertainty. Adapted from ref (200) under CC-BY. Copyright 2022 The Authors.

Ultrasonication of a biphasic CNC suspension leads to reduction in the relative proportion of the cholesteric phase.^89,314,613^ Comparison of suspensions over a range of concentrations demonstrated that undialyzed, ultrasonicated suspensions had a later onset of cholesteric behavior (i.e., higher *c*_*b*1_ and *c*_*b*2_ concentrations) and a wider biphasic range (i.e., larger Δ*c*_*b*_).^613^ However, these effects can be partially attributed to the increase in ionic strength (as discussed in section 8.3.1). For suspensions dialyzed after ultrasonication, where only morphological changes are relevant, increasing the ultrasonication dose was found to increase *c*_*b*2_ alone while *c*_*b*1_ was relatively unchanged (Figure 65a).^89^ This can be attributed to the decreasing 3D aspect ratio, consistent with Onsager theory (see section 3.2.1) combined with the widening of the biphasic region due to increasing polydispersity.^278^

For CNC suspensions at constant volume fraction, ultrasonication causes an increase in the cholesteric pitch.^314^ This pitch increase, which was observed for both undialyzed^314^ and dialyzed suspensions (Figure 65b,c),^89^ was attributed to the removal of CNC bundles that served as chiral dopants for the cholesteric phase (Figure 65e).^89^ The ultrasonication-induced fragmentation of CNC bundles (with high helical twisting power) into isolated crystallites (with lower helical twisting power) weakens the overall chiral interactions in the suspension (Figure 65d). This red-shift is enhanced by dialysis of the suspension after ultrasonication to remove the released ions.

##### 8.2.2.1 Practical Aspects of Ultrasonication

Given the widespread use of ultrasonication to tune the cholesteric behavior of CNC suspensions, it is worthwhile to discuss some practical considerations relevant to the application of this treatment to CNC suspensions, as recently reviewed elsewhere.^633,634^

The effects of ultrasonication described above require high acoustic intensity, which is typically applied by introducing a vibrating tip (also known as a probe or horn) into the sample. In contrast, bath ultrasonication, a commonly used technique for dispersing suspensions, has much lower acoustic intensity, and consequently treatments on short time scales (<1 h) have no evident effect on particle morphology.^620^ Temperature-controlled bath ultrasonication has been reported to reduce the CNC particle size and induces a red-shift in pitch, but only after impractically long treatment times (∼12 h).^635^ To complement on these milder treatments, the effect of bath ultrasonication, as well as high shear homogenization or even magnetic stirring, have been reported to affect the rheology of CNCs suspensions with minimal shift in the pitch of the produced films, but also with marked aging effects, suggesting the relevance of these treatments is relatively mild for CNCs and is mostly justified in the case of poor colloidal stability, or for substantially different systems such as CNF suspensions.^636^

The effectiveness of ultrasonication depends on the properties of the CNC suspension (e.g., sample volume, viscosity, concentration of particles), the configuration of the ultrasonicator (e.g., tip diameter, maximum tip amplitude) and the geometry of the sample container. Although many studies that use ultrasonication on CNC suspensions report experimental methods, it is not possible to reproduce their results without using an identical sonicator probe, sample volume, etc. This lack of consistency is a serious obstacle to making quantitative comparisons with historical ultrasonication data, as noted elsewhere.^633^ Moreover, given the widespread use of ultrasonication as a treatment for CNCs, this lack of consistency hinders CNC research more generally. There are two primary issues preventing quantitative comparison: (1) the lack of information on the actual power delivered to the sample, and (2) the use of inconsistent units for reporting the ultrasonication dose.

##### 8.2.2.2 Measuring Acoustic Energy Delivered to the Sample

Many studies on the ultrasonication of CNCs have estimated the acoustic energy delivered into the suspension by multiplying together the treatment duration *Δt*, tip amplitude *A* (usually reported as a percentage of its maximum possible amplitude) and the maximum electrical power of the sonicator stated by the supplier (i.e., *E*′_*S*_ = *P*_max_*AΔt*). However, the value of *P*_max_ quoted by the supplier is the electrical power rating of the device, and not the acoustic power delivered by the tip to the suspension. Furthermore, the user-defined amplitude corresponds to the magnitude of the oscillation of the tip, and does not correspond to a fixed electric power input. For example, a highly viscous suspension will require a greater electric power input than a suspension at lower viscosity, as the tip will experience greater resistance to its motion.

**Figure 66:**
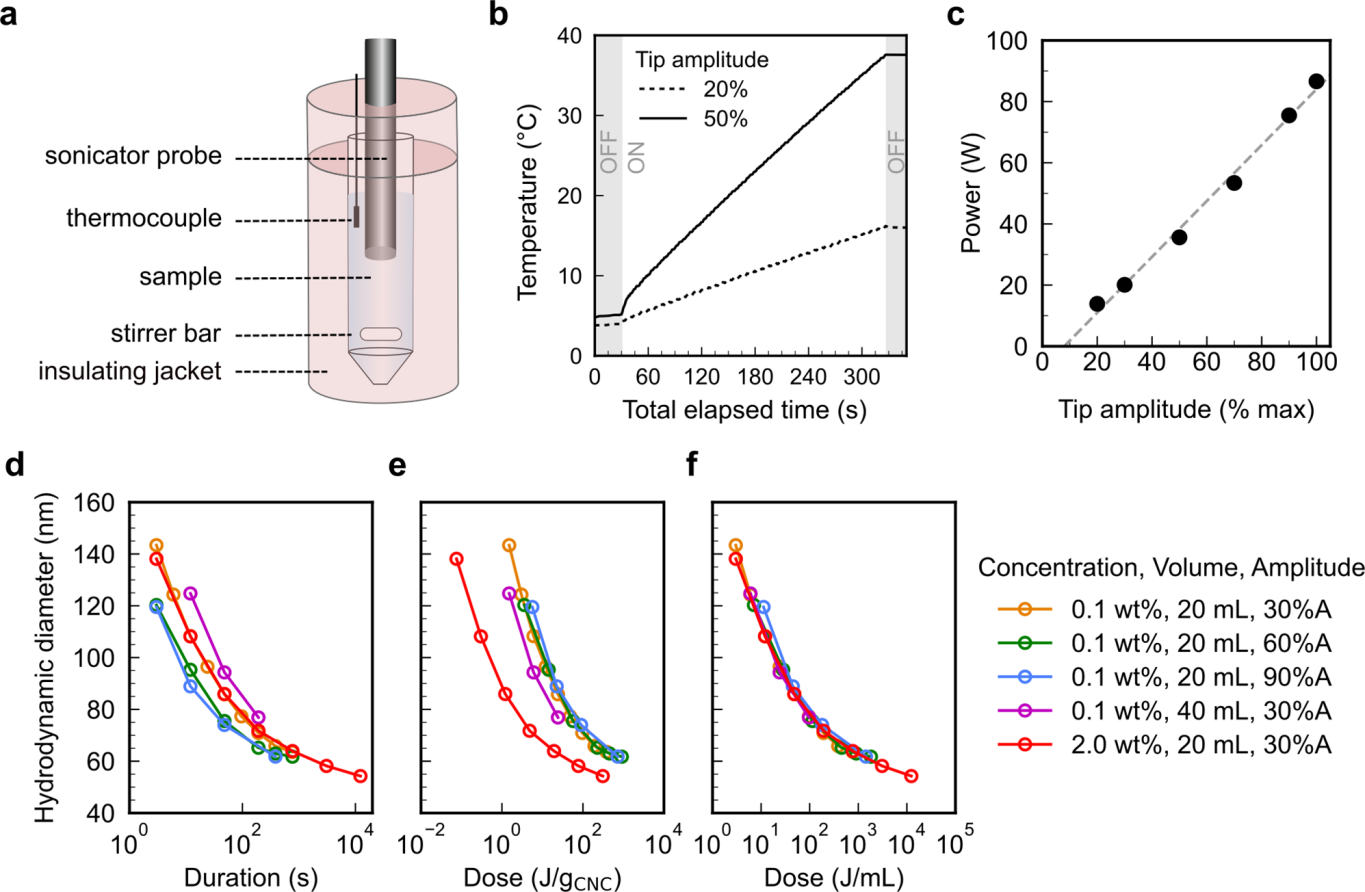
(a) Typical tip ultrasonication setup for calorimetry. (b) Temperature increase with elapsed ultrasonication time for two different tip amplitudes. (c) Calorimetric calibration of the delivered acoustic power versus tip amplitude. (d–f) CNC z-average diameter as function of ultrasonication treatment expressed in (c) duration, (d) energy per dry mass, and (e) energy per suspension volume. (a, b) Adapted with permission from ref (200). Copyright 2022 University of Cambridge. (c–f) Adapted from ref (89) under CC-BY.

As emphasized in review articles on sonochemistry^637^ and ultrasonication of nanoparticles,^630^ the acoustic energy delivered to a sample is best quantified by calorimetry (Figure 66a), and this method is now beginning to be used for CNC suspensions.^89,633,638^ In this method, the heating of the sample due to cavitation, *ΔT*, can be used to estimate the delivered acoustic energy *E*_*S*_ according to the relation
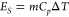
136where *m* is the mass of the sample and *C_p_* is its specific heat capacity at constant pressure (Figure 66b,c). Note that calorimetry must be performed on a well-insulated sample of a sufficiently large volume to minimize effects of heat losses and thus accurately measure the temperature increase. However, once this calibration has been performed, the values can be applied to temperature-controlled samples, which is particularly important to avoid heat-induced desulfation in acidic CNC suspensions.

##### 8.2.2.3 Quantifying the Ultrasonication Dose

For consistent application of ultrasonication between samples, it is useful to translate the acoustic energy delivered to the suspension into an ultrasonication dose experienced by the CNCs. The effect of increasing ultrasonication energy can be tracked by measuring the hydrodynamic diameter *D*_hyd_ (or alternatively by measuring suspension conductivity^633^). When comparing treatments under different conditions (Figure 66d), it can be seen that the effectiveness of ultrasonication, quantified by the hydrodynamic diameter, varies with tip amplitude and sample volume, but does not vary with CNC concentration if the tip amplitude and sample volume are kept constant.

In the nanocellulose literature, there is a historical convention of stating the ultrasonication dose as the delivered energy per CNC mass in the suspension (i.e., *w*_S_ = *E*_S_/*m*_CNC_, with units of J/g). However, this dose unit still gives inconsistent results when comparing the same set of treatment conditions (Figure 66e).^89,632^ Alternatively, the ultrasonication dose can be expressed as the delivered energy per suspension volume *u*_S_ (units of J/mL), as commonly used for other types of nanoparticles:^630,639^
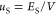
137

As shown in (Figure 66f), *u*_S_ quantifies ultrasonication dose consistently between treatments and samples and is therefore the most suitable dose unit for the concentration range considered (up to 2 wt %). For mildly concentrated suspensions, the effectiveness of ultrasonication is largely independent of the CNC mass fraction but is instead determined by the acoustic energy density (i.e., energy per suspension volume). For highly concentrated suspensions, additional effects such as poor advection and inhomogeneous ultrasonication is expected to lead to deviation from the applied *u*_S_ value. As a final comment, a hybrid dose unit *u*_S_^′^ = *E*_S_/(*m*_CNC_*V*) has also been proposed,^633^ but the excellent agreement using *u*_S_ in Figure 66f demonstrates that *u*_S_^′^ is not consistent at low CNC concentration. It has been suggested that *u*_S_^′^ may be more suitable for highly concentrated CNC suspensions,^640^ but without conclusive experimental evidence.

#### 8.2.3 Size Fractionation

CNCs typically exhibit an intrinsic size polydispersity, which arises from the cellulose source material and production method. As such, a freshly prepared CNC suspension typically contains particles with a wide range of lengths and aspect ratios. These particles can be broadly divided into elongated nanoparticles, which can form a cholesteric mesophase, and larger irregular aggregates that increase the turbidity of the suspension but do not usefully contribute to self-organization. These aggregates are typically removed by either mild centrifugation or vacuum filtration (e.g., using a membrane with 0.8 μm pore size). While the remaining suspension of elongated nanoparticles forms a cholesteric mesophase and is suitable for producing photonic films, there can be advantages to further reducing the polydispersity (see section 3.2.4).

Several methods to size-fractionate a CNC suspension have been reported, including differential centrifugation,^615^ centrifugation in a sucrose gradient,^641^ asymmetric flow field-flow fractionation (AF4),^642^ and spontaneous cholesteric phase separation,^104,272,284,285,596^ as first employed in the seminal work of Dong et al.^271^ While the first three methods primarily sort CNCs by hydrodynamic size, the final approach relates more closely to particle aspect ratio and thus is more relevant for tuning the cholesteric behavior. Furthermore, spontaneous cholesteric phase separation is more suitable as a preparatory fractionation method as it can easily be applied to larger suspension volumes (liters rather than milliliters) and does not require dilution and reconcentration steps (unlike AF4 or differential centrifugation) and/or purification (unlike centrifugation in a sucrose gradient). Moreover, while centrifugation can be relevant to fractionate CNCs according to their respective size, excessive centrifugation can also affect the aggregation state of the CNCs instead of only fractionating them, with potential effects on their self-assembly properties.^284^

**Figure 67:**
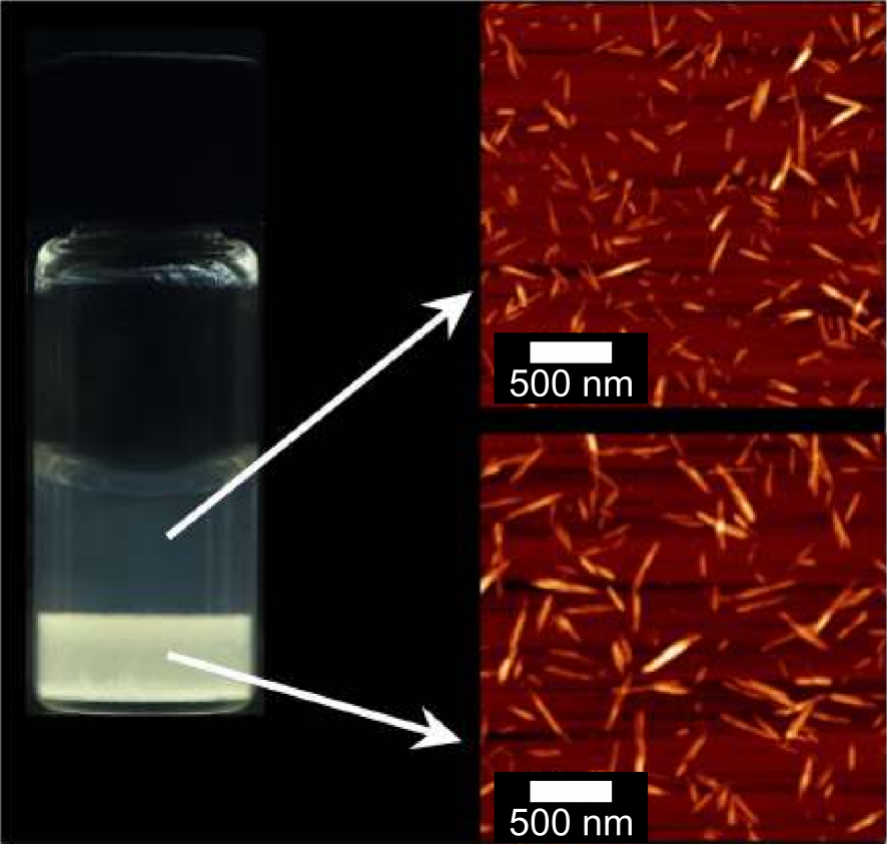
Representative phase separation of a CNC suspension in a glass vial standing between crossed polarizers. A spontaneous fractionation takes place simultaneously, with longer rods populating the bottom anisotropic phase and shorter rods in the top isotropic phase. Reproduced from ref (644) with permission from the author.

As discussed in section 3.2, a biphasic suspension will macroscopically separate into a lower cholesteric phase and an upper isotropic phase (Figure 67). If the CNCs are highly polydisperse, the higher-aspect-ratio particles will preferentially enter the cholesteric phase. As such, extracting the cholesteric and isotropic phases from an initial biphasic suspension can be used to fractionate the CNC population. Moreover, applying a moderate concentration change to an extracted phase (i.e., diluting a cholesteric phase or concentrating an isotropic phase) is sufficient to form a new biphasic suspension, allowing for multiple reiterations of this “phase-fractionation” process to enhance the discrimination between different CNC subpopulations.^272,284,285^ In principle, any method to induce phase separation (e.g., addition of depletants) could also be used for size fractionation, as previously demonstrated for gold nanoparticles,^643^ although this approach has not been specifically exploited for CNC suspensions to date.

**Figure 68:**
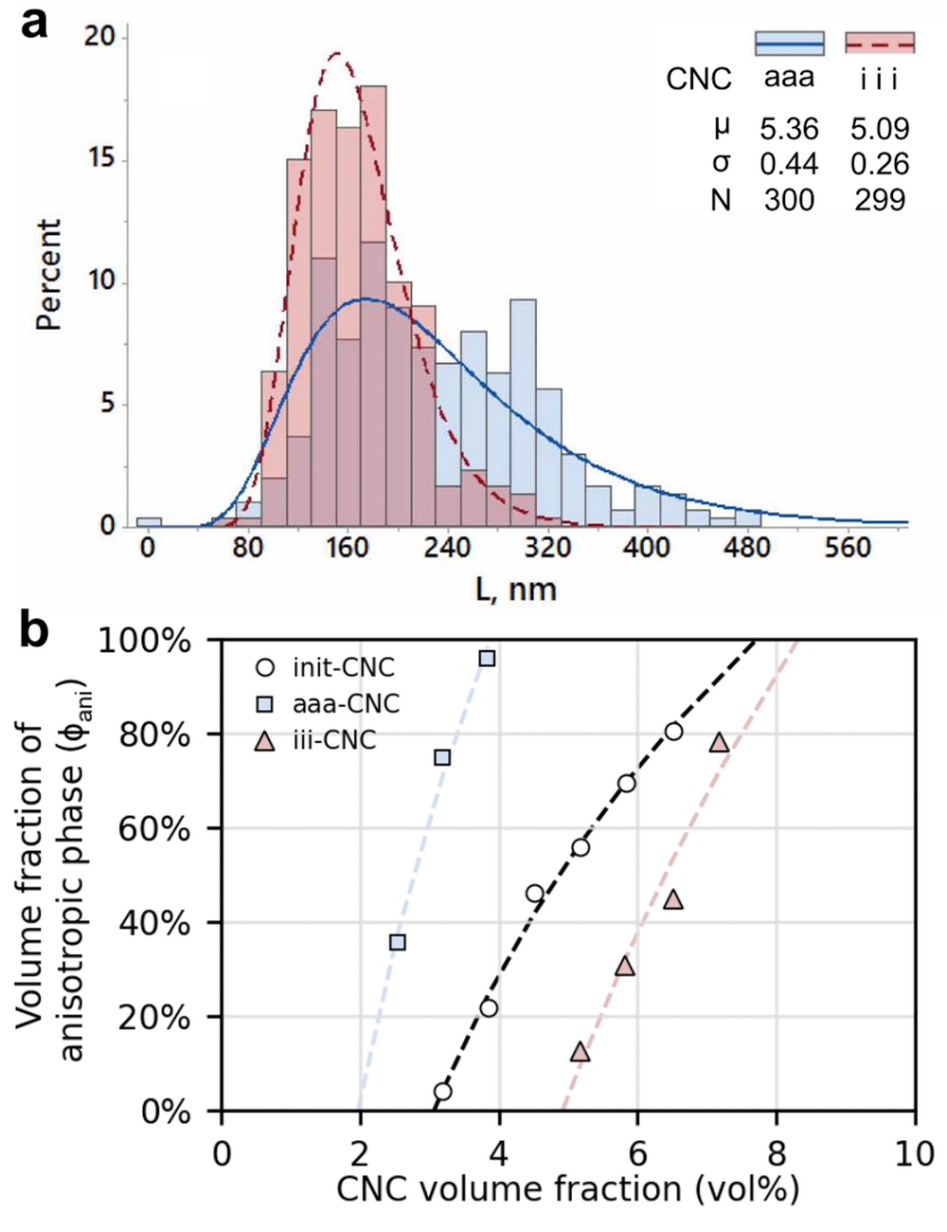
Phase behavior of 3-fold fractionated CNC suspensions, always taking either the isotropic (iii) or anisotropic (aaa) phase. (a) Distributions in CNC length (as measured by AFM), showing a clear fractionation effect. (b) Proportion of the anisotropic phase versus CNC concentration, comparing the original suspension (init) to the iii and aaa fractions. Dotted lines are visual guides (generated by logarithmic fitting). Reproduced with permission from ref (284) under CC-BY 4.0.

Although phase-fractionation has been demonstrated in several studies on CNCs from various sources, only a handful of recent studies have investigated the cholesteric behavior of the resultant fractionated suspensions.^272,284^ In a first study,^284^ an initial CNC population (init-CNC) was sequentially phase-fractionated from 50:50 biphasic suspensions (ϕ_ani_ = 0.5) to produce 3-fold isotropic (labeled (iii) and 3-fold anisotropic (labeled aaa) fractions. Substantially different size distributions were reported for the iii-CNC and aaa-CNC fractions (Figure 68a), with average lengths of 0.17 and 0.23 μm respectively, compared to 0.20 μm for init-CNC. Interestingly, while the length polydispersity of the iii-CNC fraction (measured as standard deviation over mean) was significantly lower than that of init-CNC, the aaa-CNC fraction still exhibited a broad length distribution. In terms of self-organization, both fractionated suspensions had a narrower biphasic concentration range Δ*c*_*b*_ compared to init-CNC (Figure 68b). Furthermore, the biphasic boundaries (*c*_*b*1_, *c*_*b*2_) were shifted to lower concentrations for aaa-CNC and conversely higher concentrations for iii-CNCs, consistent with the changes in mean particle aspect ratio upon fractionation. Notably, for the iii-CNC fraction, *c*_*b*2_ was shifted beyond the onset of the kinetic arrest.

**Figure 69:**
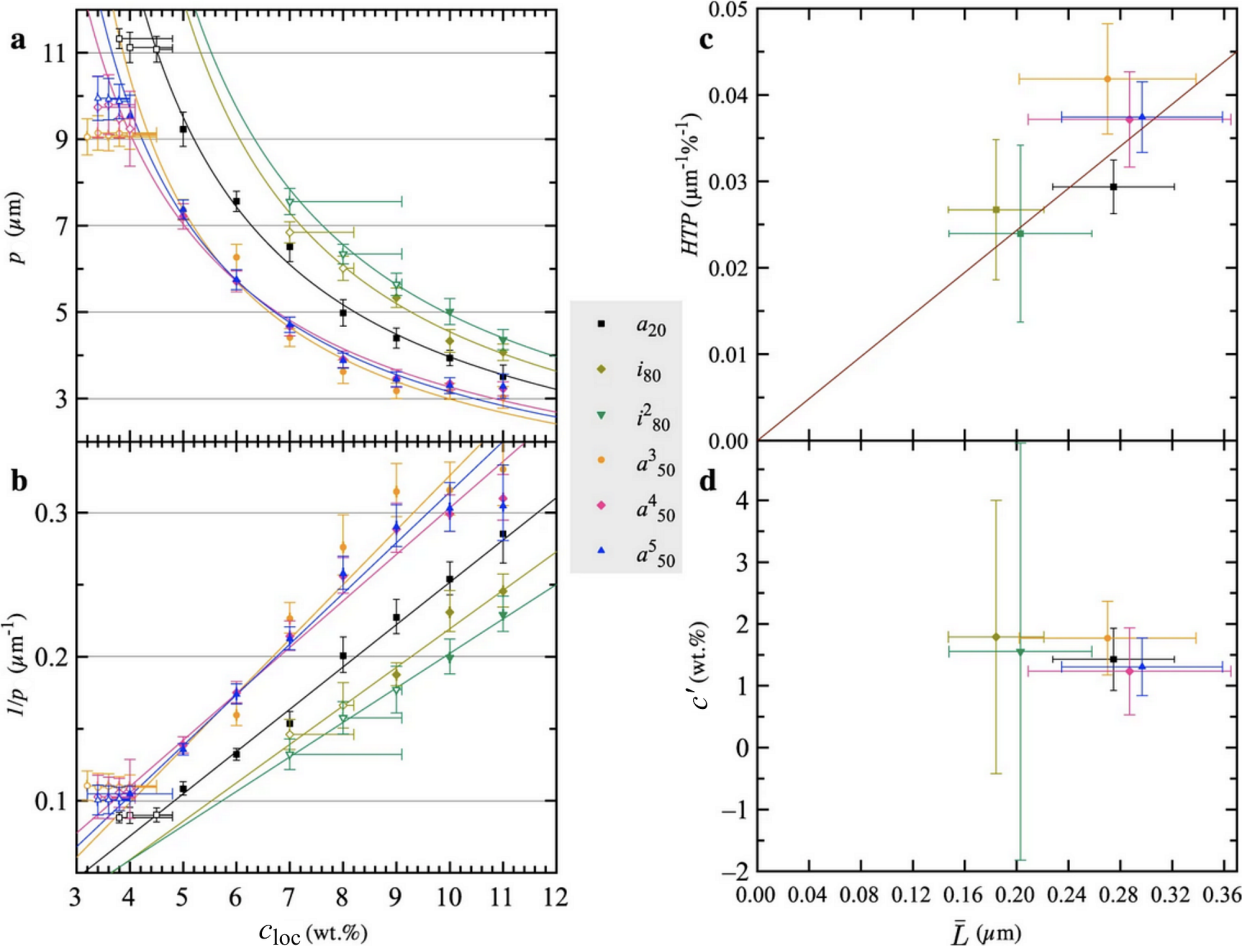
(a) Cholesteric pitch *p* and (b) reciprocal cholesteric pitch 1/*p* versus the local CNC mass fraction *c*_loc_ inside the cholesteric phase for fractionated suspensions. Lines indicate fitting with 1/*p* = HTP(*c*_loc_ – *c*′), (c) effective helical twisting power (HTP) and (d) *c*′ from fitting in (a, b) versus mean CNC length *L̅*. Adapted with permission from ref (272) under CC-BY. Copyright 2020 The Authors.

In a subsequent study,^272^ sequential phase-fractionation was used to investigate the relationship between CNC morphology and cholesteric pitch (Figure 69). The pitch *p* (denoted *p*_0_ in the original publication) was found to decrease with the local CNC mass fraction *c*_loc_ inside the cholesteric phase (denoted *W* in the original publication) according to the empirical relation 1/*p* = HTP (*c*_loc_ – *c*′), where HTP is an effective helical twisting power and *c*′ (denoted *w*′ in the original publication) is a fitting parameter. A positive linear correlation between HTP and average CNC length was observed, suggesting that longer CNCs have stronger chiral interactions. As a final observation, the kinetics of phase separation was found to strongly depend on particle length, with biphasic suspensions containing the longest CNCs (labeled *a*_5_^50^) fully separating in about a day, while the shortest CNCs (labeled *i*_2_^80^) did not reach equilibrium even after several months (Figure 69).

### 8.3 Adjusting the Formulation

In this section, methods to modify the suspension properties through changing its formulation will be presented in detail. Their respective impact on CNC suspensions and self-organization are summarized in Table 4.

**Table 4:** Tools to modify solution properties through change in formulation: respective principle/mechanism, consequences on CNC interactions and on suspension and self-assembly behavior

Technique	Principle/mechanism	Consequence on CNCs interactions	Consequences on suspension and self-assembly	Ref
Salt addition	↑ ionic strength	↓ thickness of diffuse electronic double-layer	↑ critical mass fractions	(103, 104, 271, 645)
			↓ ϕ_ani_ (volume fraction of anisotropic phase)	
		↑ impact of CNC twist	↓ pitch	
pH change	↑ ionic strength	↓ thickness of diffuse electronic double-layer	↓ ϕ_ani_ (volume fraction of anisotropic phase)	(271, 486)
			↑ critical mass fractions^a^	
			↓ pitch^a^	
Anionic dye addition	↑ ionic strength	↑ hydration radius?	↑ *c*_*b*2_ (critical mass fraction before full LC)	(646)
Neutral polymer addition	↑ particles concentration	↑ depletion interactions	↑ or ↓ pitch	(647−649)
Anionic polymer addition	↑ anionic particles concentration	↑ depletion interactions CNC exclusion	↓ pitch	(650−652)
			↑ ϕ_ani_ (volume fraction of anisotropic phase)	
			↑ Δ*c*_b_	
	↑ ionic strength^a^	↓ thickness of diffuse electronic double-layer^a^	↑ defects	(651, 652)
Solvent exchange	↑ solvent dielectric permittivity and ↓ hydrogen interactions	↑ thickness of diffuse electronic double-layer	↓ critical mass fractions	(310)
			↓ pitch	
		↑ chiral interactions	↓ pitch dependence with concentration	
	↓ solvent dielectric permittivity	↓ electrostatic interactions	sharp LC phase transition	
			earlier kinetic arrest	
Heat treatment	See Table 3			-
Ultrasonication	See Table 3			-

a Expected effect that has not been experimentally studied or explicitly stated in the source.

#### 8.3.1 Ionic Strength and pH

##### 8.3.1.1 Impact of Ionic Strength on CNC Self-Assembly Behavior

The ionic strength of a CNC suspension comprises the counterions released from the CNCs (typically H^+^ or Na^+^) and any other additional electrolytes included in the formulation (e.g., H_2_SO_4_, NaCl, ...). While the ionic strength is often considered only in terms of the latter, the counterions accompanying the CNC surface charges can also contribute to the overall ionic strength. As a quantitative example, a 2 wt % suspension of Na-CNCs with a surface charge of 300 mmol/kg will contain 6 mM of Na^+^ ions. The contribution of the counterions is most significant for a suspension dialyzed in deionized water, which has a minimal but nonzero ionic strength set by the CNC concentration and surface charge. Beyond this lower limit, the ionic strength can be increased by the addition of ions, up to the limit of colloidal stability and kinetic arrest (as discussed in section 5.1.2). For sulfated CNCs this upper limit is typically on the order of 30–50 mM for monovalent ions.^284,454,486,653^ However, this limit was found to drop for electrolytes of higher valencies, typically on the order of 3–5 mM for a 2:1 electrolyte (e.g., CaCl_2_, MgCl_2_) and below 1 mM for 3:1 electrolytes (e.g., AlCl_3_), in agreement with the Schulze-Hardy rule stemming from the DLVO theory (see section 5.1.2).^453^

**Figure 70:**
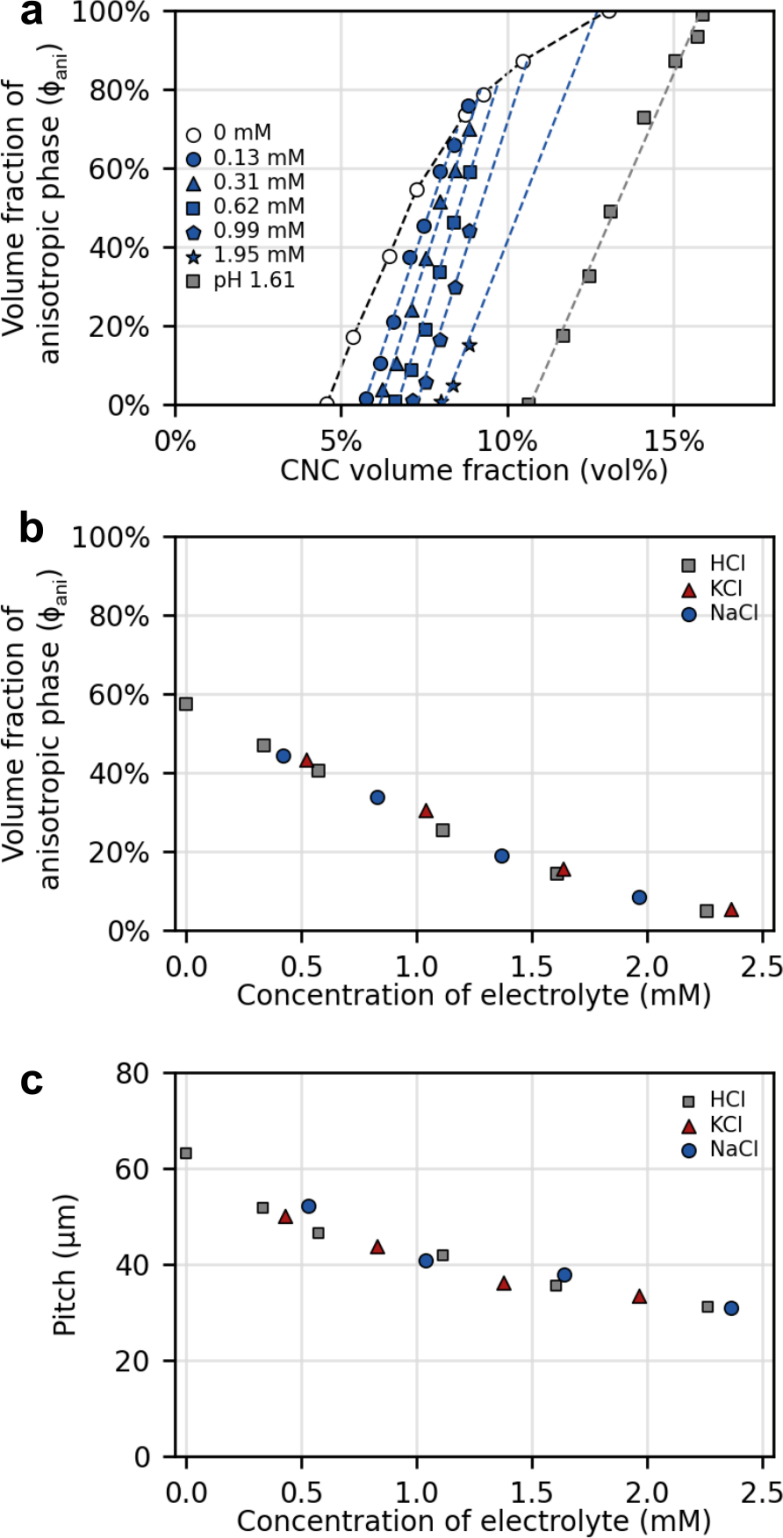
(a) Evolution of the volume fraction of anisotropic phase with the total CNC volume fraction Φ at different NaCl concentrations or at pH = 1.61 using dialysis against HCl. (b) Evolution of the volume fraction of anisotropic phase with the electrolyte type and concentration at fixed total CNC volume fraction in the biphasic sample (Φ ≈ 5.2 vol%, or *c* ≈ 8.0 wt %). (c) Evolution of the pitch p in the same conditions as in (b). Here, Φ was calculated as Φ = *c*_n_LD^2^, where *c*_n_ (in nm^–3^) is the CNC number density stated in the original study using *L* = 115 nm and *D* = 7 nm. Adapted with permission from ref (271). Copyright 1996 American Chemical Society.

Increasing the ionic strength of CNC suspensions shifts the biphasic range (*c*_*b*1_, *c*_*b*2_) to higher concentrations (Figure 70a).^271^ This effect can be attributed to a reduction in the effective CNC volume fraction, which dominates over the increase of their effective aspect ratio *a*_eff_ (see section 3.2.3). Increasing the ionic strength of the suspension enhances the screening of CNC surface charges and reduces the thickness of the electric double layer. Consequently, for a biphasic suspension at fixed CNC concentration, the anisotropic phase fraction, ϕ_ani_, decreases with increasing ionic strength (Figure 70b).^103,271^ Although the “bare” CNC concentration (in terms of vol%) is kept constant, the effective volume fraction occupied by the CNCs is reduced as the ionic strength is increased, effectively “diluting” the suspension toward the isotropic end of the biphasic region. Furthermore, this effect was found to be independent of the choice of ion species when comparing HCl, NaCl and KCl.^271^ Similarly, the choice of counterion introduced during CNC neutralization only have a small influence on the CNC self-assembly behavior.^601^

For fully cholesteric suspensions at fixed CNC concentration, increasing the ionic strength leads to a decrease in the pitch, irrespective of the choice of ionic species (Figure 70c).^271^ This effect can also be attributed to enhanced screening of CNC surface charges, but due to a different mechanism than the effective “dilution” discussed above. As discussed in section 3.3, the chiral interaction between CNCs is expected to be short-range, and therefore requires the particles to be in close proximity. At higher ionic strength, the CNC surface charges are more screened, allowing for the particles to spend a greater proportion of their time in close proximity, although the average interparticle distance remains unchanged. Consequently, the average chiral torque between CNCs is greater, leading to a smaller pitch. Conversely, in the limit of very low ionic strength, the strong repulsion between CNCs masks their chiral interactions. In this regime, the pitch is highly dependent on the presence of trace amounts of free electrolytes, which are typically challenging to remove by dialysis (see below). For example, a CNC suspension with extremely low added electrolyte concentration was prepared by centrifugation and redispersion exhibited an apparent nematic phase.^103^ However, in this case cholesteric ordering could be recovered by the addition of only 0.1 mM of NaCl.

The effect of H^+^ ions on CNC behavior is potentially more complex than that of other ions. First, as H^+^ ions contribute to the overall ionic strength, CNC suspensions are constrained to the range 1.5 < pH < 12.5 by the need to be colloidally stable (the limit at high pH being due to the analogous contribution of OH^–^ to the ionic strength). Within this range, the pH of the suspension could modify the effective CNC surface charge, depending on the p*K*_a_ value of the surface groups. It is widely believed that the sulfate half-ester groups on the sulfated CNCs have a p*K*_a_ value of around 2,^654^ which perhaps stems from a comparison with sulfuric acid, where the equilibrium between HSO_4_^–^ and SO_4_^2–^ has a p*K*_a_ value of 1.99. However, a more appropriate comparison may be to alkyl bisulfates (R-O-SO_3_H), where much lower p*K*_a_ values are reported (e.g., p*K*_a_ = −3.5 for methyl bisulfate).^655^ Potentiometric titration of H–CNC suspensions (dialyzed against deionized water) using alkaline solution exhibited an apparent p*K*_a_ value of 2.6.^656^ However, this effect can be rationalized as the release and neutralization of tightly bound H^+^ ions from the CNC surface, rather than the deprotonation of sulfate half-ester groups, as systematically investigated in the analogous system of sulfated polystyrene microspheres.^657^ Consequently, changing the pH of sulfated CNC suspensions is expected to simply be equivalent to changing the ionic strength.^500^ In contrast, pH is expected to influence the cholesteric behavior of CNCs with weak-acid surface charges (e.g., carboxylated CNCs produced by TEMPO oxidation, where p*K*_a_ ≈ 5.1), as measured experimentally,^500^ and also expected from the analogous system of aminated chitin nanocrystals (p*K*_a_ ≈ 6.3).^270^ Finally, as high pH solutions (typically above 8–9) act as a trap for atmospheric CO_2_, CNC suspensions at higher pH are expected to capture it and convert most of it into monovalent HCO_3_^–^ and divalent CO_3_^2–^, which can drastically affect their colloidal stability, especially if the suspensions are left to dry in a shallow dish in ambient conditions to favor CNC self-assembly.

##### 8.3.1.2 Controlling the Ionic Strength of CNC Suspensions

CNCs are often dialyzed against deionized water to remove excess ions after hydrolysis or other treatments. However, as more of the excess ions are removed by dialysis, it becomes increasingly more difficult to remove more of the remaining excess ions due to the Gibbs-Donnan effect:^658,659^ as ions are removed from the suspension, the greater repulsion between CNCs leads to build-up of osmotic pressure and an increased tension of the dialysis membrane. At equilibrium, the entropy gain of releasing further ions into the dialysis bath is canceled out by the associated decrease in the translational entropy of the CNCs. The Gibbs-Donnan effect can be mitigated by dialyzing at lower CNC concentrations, or by using a dialysis bath containing a deionized solution of neutral polymers to counterbalance the osmotic pressure of the CNC suspension.

Aside from extensive dialysis, the use of mixed-bed ion exchange resins has been reported to minimize the amount of free electrolytes in the stock suspension.^271^ Therefore, ion exchange resins have been used to further remove ionic impurities and improve the accuracy of surface charge measurements. However, their use is unnecessary for the latter purpose after extensive dialysis.^290^ When utilized, careful selection of the resin type is necessary, as mixed-bed type resins can consume surface protons. Alternatively, ultracentrifugation and redispersion is a quick and effective way to remove trace ions, as demonstrated for CNCs,^103^ ChNCs,^447^ and other rod-like particles,^484^ although care must be taken to ensure that centrifugation does not cause irreversible morphological changes to the particles.

To increase the ionic strength of a CNC suspension, a simple and widely used method is to mix it with an inorganic salt solution (e.g., NaCl). While it is desirable to use a concentrated salt solution to minimize dilution, care must be taken to ensure that the transient local increase in ionic strength during mixing does not overshoot excessively so as to induce some CNC aggregation. This can be avoided if the CNC suspension is instead immersed in a dialysis bath of salt solution. Alternatively, the ionic strength of acidic CNC suspensions can also be increased without external additives by heat-induced desulfation (see section 8.2.1), although this approach also leads to a reduction in CNC surface charge.

#### 8.3.2 Co-Assembly of CNCs and Additives

CNCs can coassemble with a variety of other species, such as polymers and nanoparticles. In this section, any species introduced into the suspension other than simple ions is referred to as an additive. These additives can be broadly categorized as small molecules, polymers, or colloidal particles. Their effect is primarily attributed to their ionic strength contribution, depletion interactions, and the possibility of aggregation. For additives that behave as weak acids or bases (e.g., proteins), it is important to consider the pH of the suspension relative to the isoelectric point (pI) of the additive.

Many papers have explored the impact of nonvolatile additives on the optical properties of the final photonic films, and have suggested possible mechanisms occurring in suspension. However, relatively few papers have directly investigated the coassembly of CNCs and additives in terms of their equilibrium cholesteric behavior. This section is dedicated to exploring this subject, while the impact of additives on photonic films is discussed in section 9.1, and their role in enhancing film functionality is discussed in section 10.

##### 8.3.2.1 Cationic Additives

In general, cationic additives larger than individual ions, such as cationic polymers,^650,656^ some proteins (especially near and below their isoelectric point)^660,661^ or cationic surfactants,^662^ tend to bind irreversibly with anionic CNCs, leading to aggregation and loss of self-assembly behavior. These additives should thus be avoided.

##### 8.3.2.2 Anionic and Neutral Small Additives

Nonbinding anionic dyes carrying multiple charged groups were shown to induce greater phase separation than the equivalent ionic strength of a simple 1:1 electrolyte (NaCl).^646^ Adding such dyes to fully anisotropic Na-CNC suspensions led to a phase separation between an isotropic and an anisotropic phase, effectively increasing the concentration before full anisotropic phase formation. Furthermore, increasing the number of charges generally led to an increase of the isotropic phase volume fraction, accompanied by an increase of ionic strength. However, neutral, cationic and cellulose-binding anionic dyes did not cause any separation of the anisotropic phase.^646^ Overall, these results suggest that larger hydration radius and/or the distribution of the charges on the dyes play a significant role in altering the CNC suspension properties.

Small neutral molecules are not expected to substantially affect the cholesteric behavior of CNC suspensions. For example, addition of d-glucose to a biphasic suspension at fixed CNC concentration showed no clear trend in ϕ_ani_ or cholesteric pitch (especially in the range of glucose concentration relevant for the self-assembly into photonic films).^436^ Such small additives can therefore be used to tune other suspension properties (e.g., viscosity) without affecting self-organization, although it should be noted that nonvolatile additives will in general lead to a red-shift in the pitch of the final film (see section 9.1).

##### 8.3.2.3 Neutral Polymeric Additives

Flexible neutral polymers can substantially affect the phase separation of CNC suspensions by inducing depletion interactions. There has been considerable research interest on the phase behavior of colloid–polymer mixtures, both for colloidal spheres and for colloidal rods, which is comprehensively reviewed elsewhere.^663,664^ The presence of polymers in the suspension leads to an effective short-range attraction between the colloidal particles. The strength of this depletion interaction depends on the concentration (more specifically, molarity) of the polymer, while the range of interaction depends on its molecular weight.

For rod-like colloidal particles, polymer depletants can induce isotropic–nematic phase separation for an otherwise isotropic suspension, with the polymer preferentially entering the isotropic phase. The addition of neutral polymers is therefore predicted to widen the biphasic region (i.e., lower *c*_*b*1_ and higher *c*_*b*2_),^665^ which has been experimentally verified for suspensions of elongated particles such as boehmite rods^666^ and rod-like viruses.^667^ Depending on the initial rod concentration, the addition of polymer can either increase or decrease ϕ_ani_. Finally, the impact of depletion is also dependent on the ionic strength of the suspension, as at low ionic strength the long-range repulsion between rods renders the short-range depletion interaction irrelevant, as reported for fd virus suspensions.^667^

The addition of high-molecular weight neutral polymers such as PEG (200 kDa) or dextran (450 kDa) has been reported to reduce ϕ_ani_ in a biphasic CNC suspension.^578^ However, the effect of depletants on CNC phase separation has only been quantitatively investigated using anionic polymers,^651,652,668^ where the effects of depletion are coupled to the effects of ionic strength.

##### 8.3.2.4 Depletion and Anionic Polymeric Additives

**Figure 71:**
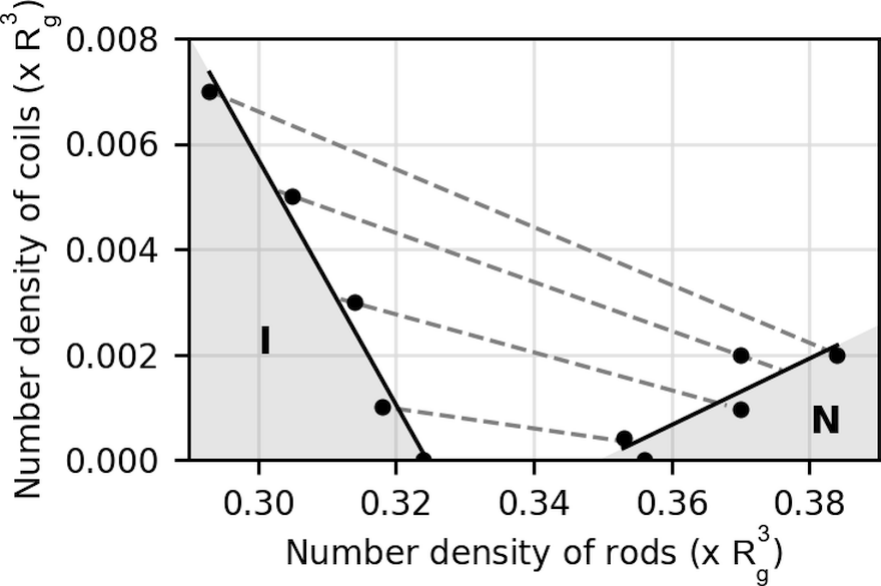
Partial phase diagram of CNC suspension with blue dextran (2,000 kDa) with isotropic region (I) and cholesteric region (N) indicated. With *R*_g_ the radius of gyration of the blue dextran (36 nm). Adapted with permission from ref (651). Copyright 2002 American Chemical Society.

Different amounts of negatively charged blue dextran (200 kDa) were added to biphasic CNC suspensions with identical mass fraction (10.3 wt %) having an anisotropic phase fraction ϕ_ani_ = 0.6.^651^ Upon dextran addition the relative proportion of CNCs in the anisotropic phase increased, while the blue dextran was concentrated in the isotropic phase (Figure 71). Similarly, adding blue dextran to a fully anisotropic sample led to phase separation into an anisotropic phase more concentrated in CNCs and an isotropic phase rich in blue dextran. Consequently, the anisotropic phase had a smaller pitch than the initial suspension, which may partially be attributed to its higher concentration, but also to the fractionation of CNCs across the two phases (see section 8.2.3). The anisotropic phase also had a less ordered texture with smaller cholesteric domains. The pitch dependence on CNC and polymer concentration was not systematically investigated.

**Figure 72:**
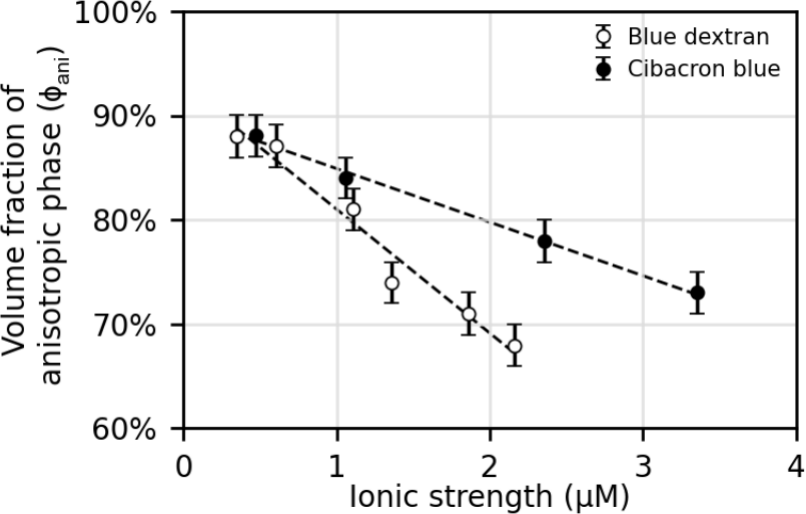
Volume fraction of anisotropic phase (ϕ_ani_) versus added ionic strength due to charged dye molecules, namely blue dextran (2,000 kDa) ligands (open circles) or free Cibacron 3G-A dye (full circles). Data reproduced with permission from ref (652). Copyright 2006 American Chemical Society.

In contrast to charged dextran, the addition of neutral dextran to anisotropic CNC suspensions did not affect the phase separation behavior.^652^ This observation was explained by the low ionic strength of the medium, which is expected to favor electrostatic repulsion over depletion interactions. However, with the negatively charged blue dextran, signs of depletion were observed through microphase separation of the polymer and the CNCs, leading to a decrease in the onset of the biphasic regime (*c*_*b*1_), and increase of the biphasic range and an increase of ϕ_ani_. Increasing the charge on the 2,000 kDa dextran with constant dextran number density enhanced the phase separation by lowering ϕ_ani_ before inducing gelation, confirming the electrostatic nature of the phenomenon (Figure 72). Interestingly, the effect was greater than by adding the free blue dye (Cibacron Blue 3G-A) confirming the importance of the depletion.

Notably, depletants can also lead to three-phase equilibria (isotropic–isotropic–nematic or isotropic–nematic–nematic), depending on the aspect ratio of the rods and the molecular weight of the polymer.^665^ Such behavior has been reported for non-CNC systems such as boehmite rods,^669^ and has been systematically investigated for CNC suspensions containing mixtures of neutral and anionic dextran.^668^ Adding such mixtures to a biphasic suspension leads to the formation of a three phase system made up from an isotropic I_1_ (blue dextran rich, CNC poor), then another isotropic I_2_ (richer in CNCs, poorer in blue dextran), followed by a cholesteric at the bottom (rich in CNC, poor in blue dextran). Increasing the relative amount of blue dextran or dextran was found to favor the formation of I_1_ or I_2_ respectively.

Finally, nanoparticles may also potentially act as depletants, which have been shown to decrease ϕ_ani_ in spherical emulsion droplets (as discussed in section 9.2.3).^307,422^ This approach has also been proposed as a facile size sorting method for polydisperse nanoparticles.^670^ However, for charged nanoparticles, as with charged polymers, the impact of ionic strength on the phase behavior cannot be discounted. For instance, negatively charged bovine serum albumin (BSA) amyloids coassembled with CNCs were found to increase *c*_*b*1_ and *c*_*b*2_, which can be explained by the increase in ionic strength, while the accumulation of BSA in the isotropic phase is indicative of depletion.^671^ Note that the coassembly of CNCs with nanoparticles more generally is discussed in sections 10.1 and 10.4.

In summary, there is scope for further investigation on the impact of depletants on CNC self-organization. In particular, the combination of added electrolytes and polymers allows the colloidal interactions between CNCs to be fine-tuned. Overall, the use of depletants favors macrophase separation, thus widening the biphasic region, with an accumulation of depletants in the isotropic phase. However, the microphase separation also stabilizes the isotropic core of disclination lines and grain boundaries between cholesteric domains, and results in a more disordered cholesteric structure. This effect is important for the domain size in photonic films (see section 10.1).

#### 8.3.3 Non-Aqueous Solvents

The electrostatic nature of CNC stabilization interactions hinders their dispersion and self-ordering properties in many nonaqueous solvents. For example, the low dielectric permittivity of apolar solvents reduces the ability for charged groups to dissociate, resulting in weak electrostatic surface charge. This loss of electrostatic repulsion, and the absence of cellulose-water hydrogen bonds, lead to CNC aggregation. Nevertheless, sulfated CNCs can be dispersed in a variety of polar solvents (e.g., DMSO or DMF), or stabilized in apolar solvents (e.g., toluene or cyclohexane) using surfactants, while also preserving the ability to form a cholesteric mesophase.^18^ Alternatively, surface modification (i.e., replacing sulfate half-esters with various nonionic groups) enables the dispersion of CNCs in a wider range of solvents. While this approach has been explored in the context of using CNCs in polymer composites,^672,673^ relatively few studies have investigated the ability of these suspensions to form a cholesteric mesophase.

**Figure 73:**
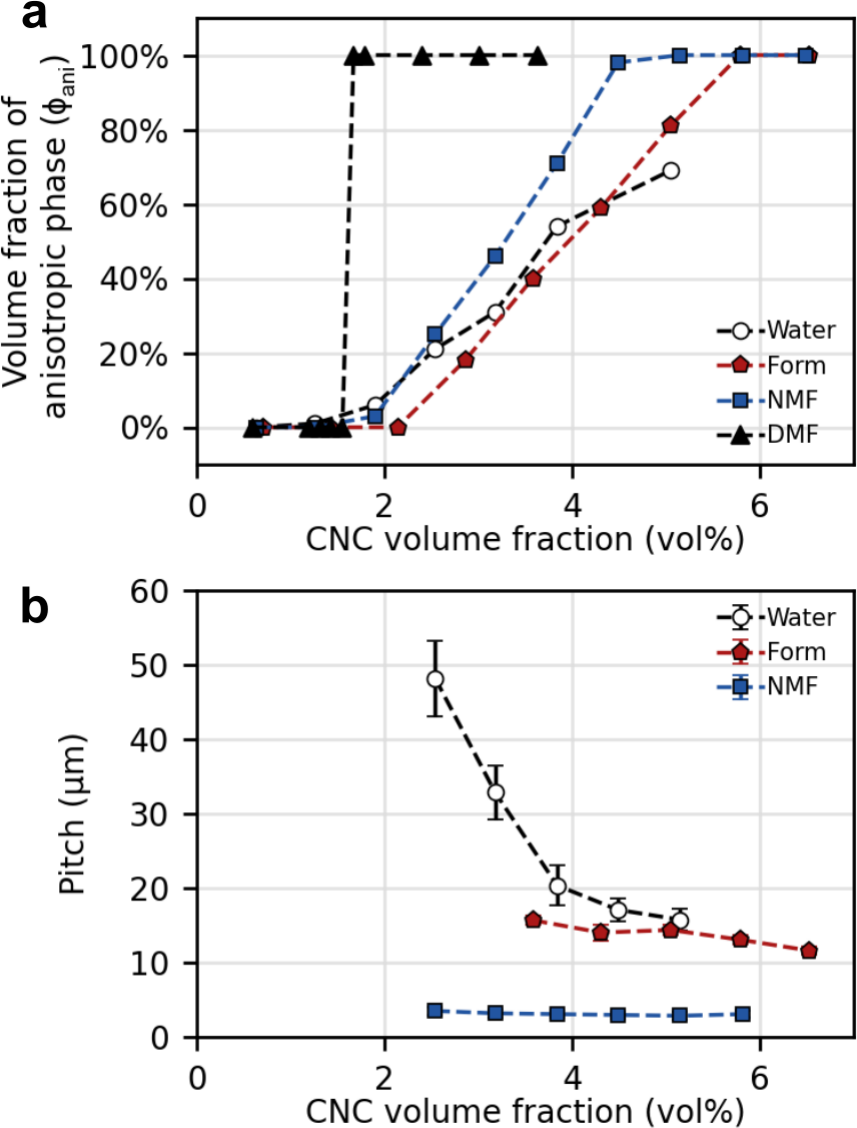
(a) Anisotropic phase volume fraction (ϕ_ani_) and (b) pitch (p) of CNC in different solvents as function of the CNC volume fraction Φ. The pitch was measured from the POM fingerprint texture. Adapted with permission from ref (310). Copyright 2016 American Chemical Society.

Cholesteric ordering has been reported for CNCs dispersed in the protic solvents formamide and *N*-methylformamide (NMF).^310^ To avoid aggregation, solvent exchange was performed by first adding the organic solvent to an aqueous suspension of CNCs and then extracting the water by evaporation. When comparing the phase behavior of these suspensions to the original aqueous suspension, *c*_*b*2_ was found to differ between the solvents, while *c*_*b*1_ was relatively unaffected (Figure 73a). These observations were rationalized by considering the dependency of the Debye screening length on the permittivity of the solvents (, where *ε*_*r*_ ≈ 80, 111, and 189 for water, formamide and NMF respectively) and their Lewis acidity.^310^ All three suspensions displayed cholesteric ordering, with increasing dielectric permittivity correlated to a smaller cholesteric pitch and a weaker pitch dependence on the CNC concentration (Figure 73b). Notably, for NMF the pitch of the anisotropic phase in the biphasic regime exhibited negligible variation with overall CNC concentration. In the same study, the dispersion of CNCs in the related, but aprotic, solvent dimethylformamide (DMF) was also investigated. DMF-dispersed CNCs exhibited a sharp transition from fully isotropic to a kinetically arrested state between 1 and 2 wt % (Figure 73a), which was attributed to the lower dielectric permittivity of DMF (*ε*_*r*_ = 38).

Using suitable surfactants, cholesteric ordering has been achieved in low-permittivity apolar solvents (cyclohexane and toluene).^315^ The CNCs were mixed with the surfactant prior to freeze-drying, then redispersed in the chosen solvent, and the excess of surfactant was removed by centrifugation and redispersion. The colloidal stability of the surfactant-coated CNCs in these solvents can be attributed to the formation of a hydrophobic layer around the CNCs. Furthermore, when compared to water, apolar solvents are often closer to cellulose in their dielectric properties (i.e., refractive index and dielectric permittivity), and as a consequence London dispersion forces between CNCs are reduced in these solvents.

**Figure 74:**
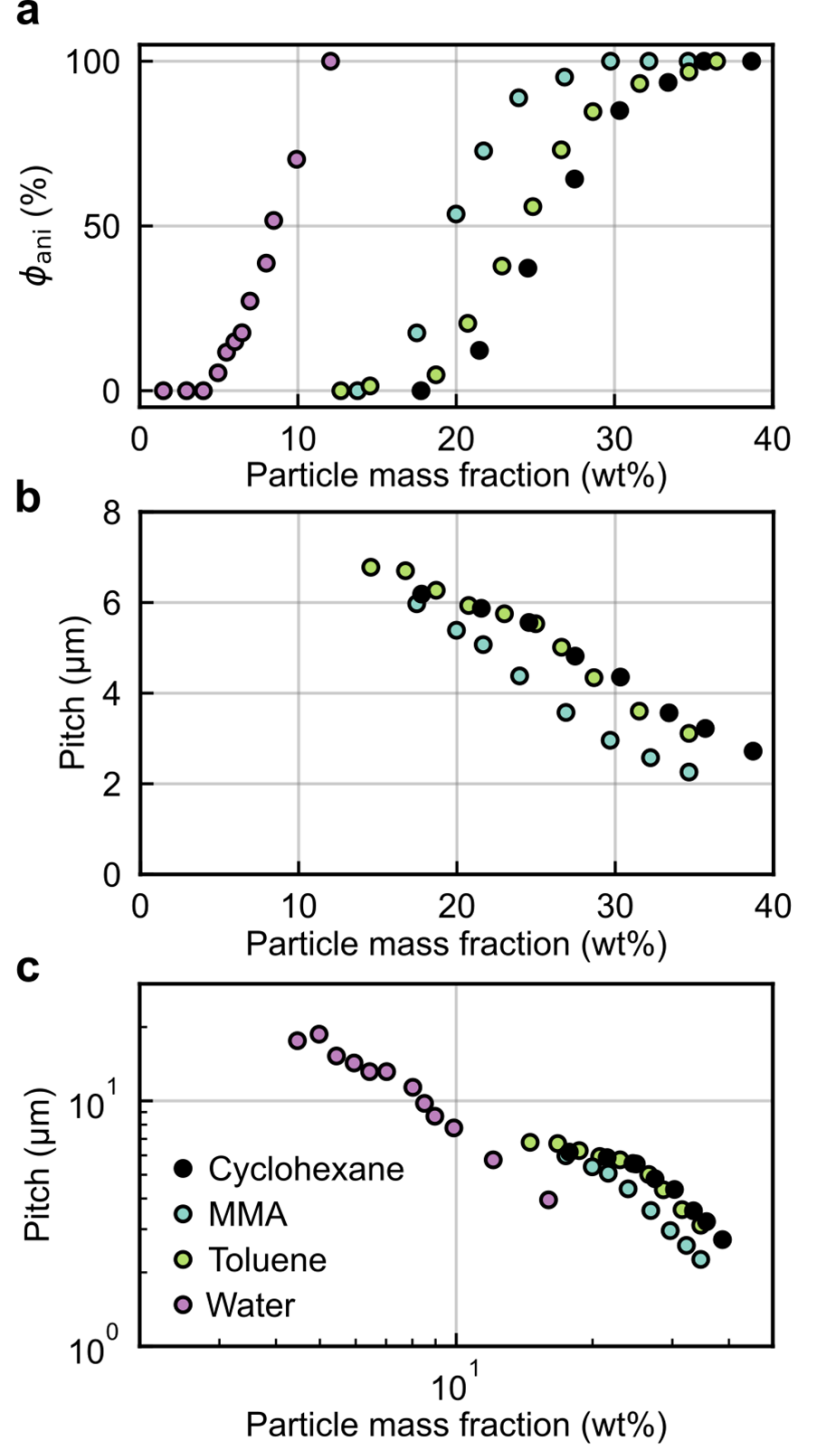
Influence of solvent on CNC cholesteric behavior, comparing suspension in water to surfactant-stabilized CNCs in selected apolar solvents. (a) Proportion of anisotropic phase versus mass fraction of particles, which consist of the CNCs and any physisorbed surfactant. (b) Cholesteric pitch versus particle mass fraction on a linear–linear scale. Water data at much larger pitch values is excluded for clarity. (c) Pitch versus particle mass fraction on a log–log scale, including water data. Note that particle concentration is expressed as total CNC+surfactant mass fraction here, rather than volume fraction, as the surfactant layer makes it difficult to accurately estimate the true particle volume. Data reproduced from ref (268).

The phase behavior of surfactant-stabilized CNC suspensions in cyclohexane, methylmethacrylate (MMA) and toluene is shown in Figure 74a, and compared to the phase behavior of the original aqueous CNC suspension.^202,268^ The onset of phase separation in apolar solvents occurred at much higher mass fraction (including the added surfactant) than in aqueous suspensions of the same CNCs (around 20 wt % versus 3 wt %, respectively). Notably, the pitch measured of the anisotropic phase of such biphasic suspensions decreased less steeply with concentration that for fully cholesteric suspensions (Figure 74b,c).^202,249,268,315^

### 8.4 Summary

CNC self-assembly is governed by their individual characteristics (morphology and surface chemistry mainly) and their environment (e.g., conditions, solvent, salts, additives). As shown in this section, relatively small experimental changes can have considerable impact on CNC cholesteric ordering. In principle, this sensitivity makes it possible for the experimental parameters to be fine-tuned to achieve the desired CNC self-assembly behavior. In practice however, it is often challenging to control all relevant experimental parameters and to decouple their effects on the observed cholesteric behavior.

From their isolation, CNC characteristics are influenced by the cellulose origin, history, hydrolysis treatment and other post-treatments. The few attempts to relate CNC characteristics to one of the several parameters involved in this process are often contradictory. As a result, while the greater trends are relatively well-defined, other smaller effects remain poorly understood or unexplored. Consequently, fine-tuning of CNC characteristics through the isolation process is still approximate, highlighting the mandatory nature of a good characterization of the isolated CNCs prior to their use.^24^

Fortunately, the sensitive nature of CNC characteristics also facilitates postprocessing modifications. After isolation, the morphology of the CNCs can be tuned through ultrasonication or fractionation. Surface charge control and steric stabilization also are powerful tools to tune CNC self-assembly behavior. This section presented the most important modifications known to have an impact on the self-assembly behavior, but numerous other surface functionalization methodologies have been developed^616−618^ as well as CNCs naturally displaying steric stabilization (“hairy” CNCs).^23,674^ Therefore, it is now up to the research community to investigate their use to widen self-assembly applications.

The behavior of the CNC cholesteric phase in suspension is a good indicator of the properties of the subsequent photonic films. However, the film optical properties are eventually determined by the drying conditions that are discussed in the next section.

## 9 Guiding CNC Self-Assembly into the Solid State

To produce structurally colored films, an initial dilute suspension of colloidal CNC nanoparticles needs to be concentrated into a dense assembly via the removal of solvent. As described in earlier sections, this process is complex, with the suspension passing through various physical transitions on the pathway to a solid deposit. Consequently, there are many key parameters that need to be considered to ensure that the final film not only has a helicoidal nanostructure, but also that individual domains have the required periodicity (i.e., pitch) and orientation to achieve the desired visual appearance.

In this section, we first provide a pragmatic overview of the key parameters for dish-casting a photonic CNC film via evaporation, which is the most prevalent approach in the literature. This foundation will then be applied to understand recent efforts by the community to investigate alternative deposition techniques, and to explore the more scalable or continuous processes that are required to unlock industrial fabrication of photonic CNC materials.

### 9.1 Adjusting the Environment for Dish-Cast Films

The environment in which a CNC suspension dries strongly determines the optical properties of the resultant film. The most common methodology is to slowly evaporate a dilute aqueous CNC suspension under ambient conditions, within a small container such as a Petri dish (Ø ≲ 10 cm). However, while this process appears straightforward there are many parameters that require optimization to achieve a strong photonic response, spanning from the formulation of the initial suspension to the physical environment upon drying, and even the presence of external fields or forces. As such, each parameter will be considered individually, loosely following the chronological order within the cholesteric self-assembly process.

#### 9.1.1 Formulation of the CNC Suspension

The formulation of the CNC suspension is crucial to control its liquid crystalline properties. As discussed in section 8.1, the cholesteric pitch of the CNC suspension arises from the morphology and surface charge of the individual nanorods and as such the choice over the source (e.g., cotton vs wood-pulp) and processing method into a CNC suspension (e.g., hydrolysis conditions) can determine the overall parameter space that the suspension can be tuned across. Then, once a CNC suspension has been selected, there are several formulation parameters that are routinely adjusted prior to casting to smoothly tune the reflected wavelength of the resultant film. For example, the final color can be blue-shifted by reducing the range and/or strength of repulsive interactions between CNCs in suspension, either by lowering the surface charge on the CNC (via heat-induced desulfation, see section 8.2), or by screening the charge between CNCs via addition of an electrolyte (e.g., NaCl, HCl, H_2_SO_4_, see section 8.3). Conversely, the final color can be red-shifted by reducing the pitch contraction upon drying by breaking apart the crystallite bundles that act as colloidal chiral dopants (e.g., via tip ultrasonication, see section 8.2), or the replacement of water with a nonvolatile hydrophilic additive, such as glucose or poly(ethylene glycol), which prevents complete collapse of the helicoidal nanostructure upon final drying (see section 10.1). Moreover, while these parameters are often simply considered in terms of which direction they shift the pitch in the solid state, it is important to note that they frequently alter both the colloidal properties of the CNCs (e.g., colloidal stability,^675^ viscosity^87,613^) and the cholesteric self-assembly process (e.g., mesophase formation, domain evolution, onset of kinetic arrest). For example, CNC suspensions at very low ionic strength are highly viscous (see section 5.2.2), but this can be significantly reduced by either adding electrolytes (to increase the ionic strength) or applying ultrasonication (which reduces the CNC aspect ratio but also releases ions).^311,454,507,676^ However, depending on the method used, this can also lead to a respective blue-shift or red-shift of the resultant photonic film. As such, these parameters cannot be simply treated as additive and instead need to be optimized concertedly to produce vibrant photonic films with a wide gamut of colors.

While the suspension parameters can dictate the pitch evolution from a dilute suspension to a solid film, the various time scales involved in the cholesteric self-assembly process makes the final structure strongly influenced by the kinetics of drying. In general, the uniformity of the helicoidal nanostructure (and thus the vibrancy of the optical response) can be improved by maximizing the time for cholesteric tactoids to form, sediment, reorient and finally merge to form large domains with minimal defects. As such, the most important drying regime for cholesteric self-assembly is between the first liquid crystal transition, *c*_*b*1_, and the onset of kinetic arrest, *c*_KA_. While these threshold concentrations are suspension-specific, the time spent in this regime (*t*_KA_ – *t*_b1_) can be controlled via the dry mass of CNCs cast (*M*_CNC_ = *c*_init_*V*_init_, i.e., the product of the initial CNC concentration and suspension volume) and the evaporation rate *R* (which is typically constant for the majority of the process for a dish geometry). This can be expressed as
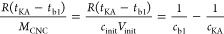
138

**Figure 75:**
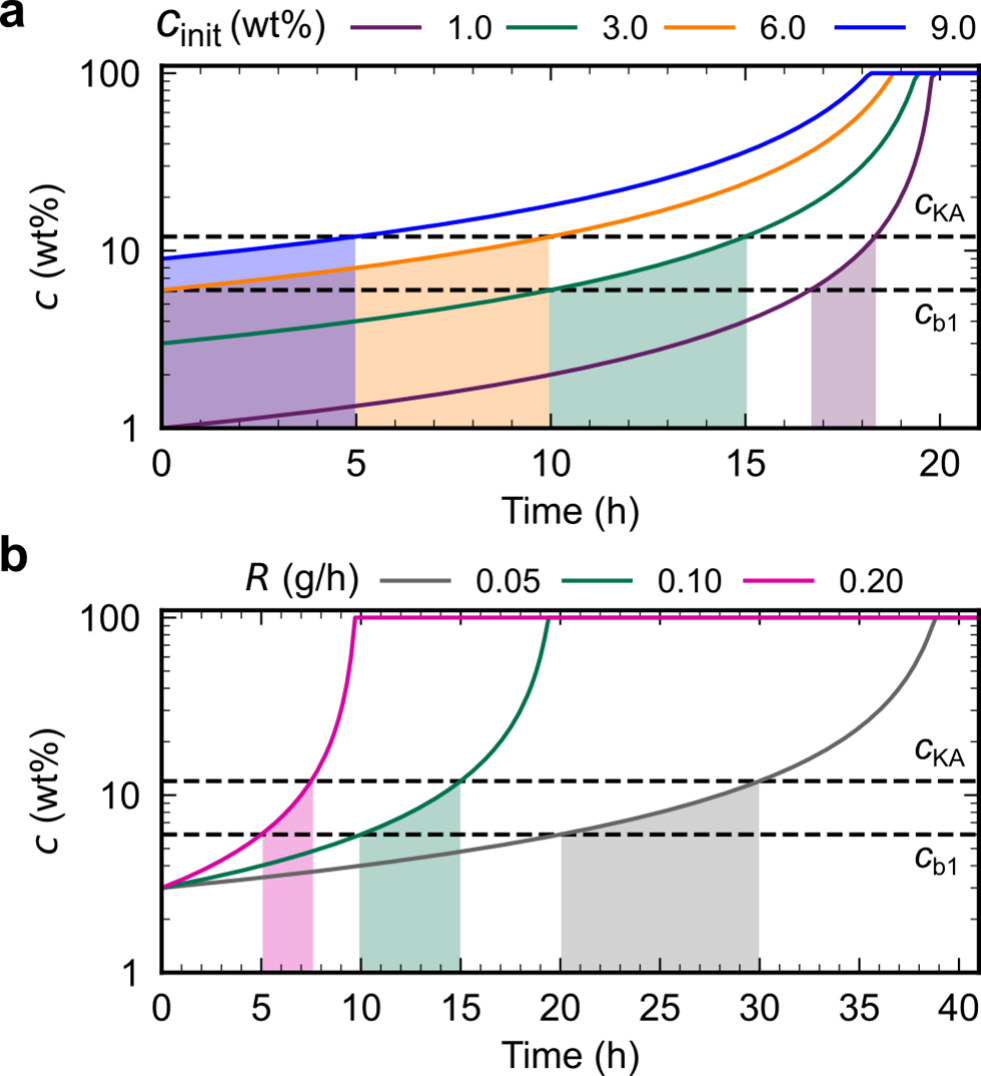
Evolution of the CNC concentration upon drying a suspension in a dish, as modeled using eq 138. (a) Effect of varying the initial CNC concentration *c*_init_ = 1–9 wt % for constant initial volume and evaporation rate (*V*_init_ = 2.0 mL, R = 0.1 g/h). Black dotted lines indicate typical values for the first liquid crystal transition (*c*_*b*1_ = 6 wt %) and onset of kinetic arrest (*c*_KA_ = 12 wt %). Shaded regions below each plot indicate the time interval spent in this “self-assembly window”. (b) Effect of varying the evaporation rate *R* = 0.05–0.20 g/h for constant initial volume and concentration (*V*_init_ = 2.0 mL, *c*_init_ = 3 wt %). The green lines in (a) and (b) correspond to identical drying conditions.

As such, if the total evaporation time is constant (e.g., by fixing the suspension volume and dish geometry), casting at a higher initial concentration (*c*_init_) can increase the time spent in this “self-assembly window” (i.e., *c*_KA_ – *c*_b1_), leading to improved optical appearance for resultant photonic film (Figure 75a).

Specifically, a comparison of different *c*_init_ values suggests that it is beneficial to start from at least c_b1_, as lower concentrations spend a proportion of the drying process simply concentrating an isotropic suspension. Conversely, while higher concentrations (*c*_init_ > *c*_*b*1_) also have a shortened self-assembly time, the suspension benefits from a degree of preorganization. By casting from a partially or fully cholesteric suspension, it avoids the in situ formation of randomly oriented tactoids, leading to a much reduced occurrence of defects within the film and improved alignment of the helicoidal domains relative to the film interfaces.^372^ Finally, by allowing the CNC suspension to phase separate and then isolating the bottom phase, it is possible to cast a fully anisotropic suspension from a lower concentration, offering advantages of both reduced viscosity and a wider self-assembly window than if the original biphasic suspension was simply concentrated further to a fully anisotropic state.^334^ By employing a more extensive fractionation process (see section 8.2.3), it has also been shown that CNCs with higher average aspect ratio can be isolated via this method, which offer both a stronger helical twisting power (leading to smaller pitches),^272^ and an earlier onset of the cholesteric mesophase without promoting an earlier kinetic arrest, thus extending the self-assembly window.^284^

#### 9.1.2 Casting Conditions

As discussed in section 7.3, near-optimal reflectance can be achieved from photonic CNC structures of only a few 10s of microns in thickness. As such, to maximize the yield of photonic material, CNC suspensions are typically cast in shallow, flat-bottomed containers, as this results in thin films (e.g., 5–50 μm) with a large surface area (e.g., 10–50 cm^2^). Although other geometries have also yielded photonic films, from uniaxial drying within a flat capillary (e.g., 50 μm × 1 mm cross-section),^432^ or between two free moving plates (e.g., between coverslips),^374,375^ to freestanding drops on a surface.^338,677^ At the laboratory scale, a convenient method to standardize film casting is to use Petri dishes, as they offer a well-defined container geometry, reproducible and tailorable surface chemistry (e.g., hydrophobic polystyrene vs hydrophilic glass) and high optical transparency.

Beyond the geometry of the container simply defining the area of the dry film, pinning the CNC suspension to the dish walls ensures (i) a near-constant evaporation rate of solvent (due to the geometrically constrained surface area of the meniscus during the majority of the drying process), and (ii) promotes the uniaxial vertical compression of the cholesteric structure after kinetic arrest, which is crucial for accessing pitch values that correlate with the visible reflection band (see section 6.1).

The wettability (hydrophilicity) of the substrate depends on the material used (e.g., polystyrene, glass, PTFE) and any chemical treatment (e.g., exposure to strong acid or base, plasma or corona treatment). However, for films produced from pinned dish-cast suspensions, the visual appearance is generally expected to be consistent between substrates, provided that the material is impermeable and chemically inert. This is because any differences in surface energy are unlikely to sufficiently perturb the strong planar anchoring of CNCs at the CNC/air and CNC/substrate interfaces, while any changes in the meniscus shape arising from a differing contact angle with the walls of the dish will be mostly localized to the edges of the film. In contrast, the wettability of the substrate can significantly affect the self-assembly geometry of an unconstrained drop of CNC suspension,^336^ with different appearances observed for CNCs drop-cast on different materials (e.g., metals, polymers, ceramic, wood).^678−680^

In general, there are two key practical considerations where the wettability of the dish can have an impact:

(i) *Upon casting a film*. It can require a larger initial volume of suspension to cover the base of a hydrophobic dish compared to a hydrophilic dish of the same dimensions, which puts a constraint on how thin a film can be cast. This issue can be partially mitigated by initially overloading the dish to enable the suspension to be pinned to the walls, followed by removal of excess suspension to thin the sample, while relying on surface tension to prevent dewetting.
(ii) *Upon detaching a film.* CNC suspensions cast in a hydrophilic dish (e.g., acid-cleaned glass) typically produce films which have good adhesion to the substrate, which can make them challenging to remove. In contrast, CNC suspensions cast in a hydrophobic dish (e.g., polystyrene) often buckle and lift-off the substrate surface upon final drying, which aids in their removal, but at the expense of flatness.

Lastly, it is useful to consider the volume of CNC suspension cast to produce the film. When casting within a container of fixed aspect ratio, the evaporation flux will be constant,^526^ but the corresponding change in CNC concentration will depend on the initial volume of suspension. Consequently, casting a larger volume of a given concentration will allow for a longer duration in the self-assembly window but will also lead to a thicker (and thus potentially more disordered) film. Relatedly, casting a fixed volume and concentration, but in a dish with a larger area will lead to both a higher evaporation rate and a thinner film. As such, the volume and concentration of the initial suspension needs to be adjusted pairwise for a given dish to optimize the visual appearance for a specific film thickness.

#### 9.1.3 Physical Environment during Evaporation

The complex dynamics of drying a CNC suspension (evaporation front and concentration gradients, surface tension and wetting, capillary forces, etc.) can all play a role in determining the visual appearance of the resultant photonic film and thus the ambient environment needs to be considered to ensure reproducibility between castings. For example, the rapid evaporation of a dilute suspension can lead to polydomain films and/or a red-shifted appearance. The polydomain structure is due to the insufficient available time for the nucleated tactoids to merge, while the redshift can be attributed to the inability of the suspension to maintain an equilibrium pitch upon rapid concentration (see section 5.4.3). Conversely, very slow or delayed evaporation of a biphasic suspension can lead to in situ phase separation, resulting in an decrease in the degree of order of the nanostructure from the bottom to the top of the film cross-section.^334^ Furthermore, additional textures can occur during the drying of CNC films,^526^ which may arise from complex flows.

A common strategy to improve the quality of the photonic response is to extend the overall time taken to dry the suspension into a film, which proportionately extends the time spent in the self-assembly window (i.e., *c*_KA_ – *c*_b1_), as exemplified in Figure 75b.^526^ Increasing the relative humidity in the vicinity of the drying suspension reduces the evaporation rate *R*, such that drying can occur slowly over ca. 1–3 days. This can be achieved using humidity-controlled chambers, which enable precise and tunable control throughout the drying process,^526,681^ or simply by placing a lid on the dish.^335^ In the latter case, it is necessary to ensure that the lid does not entirely prevent evaporation,^334^ while also not introducing inhomogeneities or gradients across the film. Conversely, differential evaporation rates can be introduced deliberately (e.g., via a mask) to impart color gradients or patterns to the photonic CNC film.^510^ This approach could potentially be also exploited to mitigate the coffee-ring effect observed near the edge of the suspension (see section 9.2.2).^682^ Lastly, it is interesting to note that the slow drying of a small volume of cholesteric CNC suspension can result in very thin, uniformly aligned CNC films (<3 μm thick) that display a distinct mosaic of colored domains (Figure 16).^337,374^ As explained in section 4.2.1, the formation of a pair of disclination lines in a well-aligned cholesteric suspension will result in a discontinuous variation of the pitch, but this difference is only significant when the thickness of the suspension is on the order of only a few pitch repeats.

While drying films over several days is acceptable for laboratory samples, such slow evaporation is incompatible with large-scale commercial production. As such, to accelerate the rate of water loss from the suspension, the evaporation rate can be raised by e.g. heat,^431^ or the water vapor removed by e.g. an air flow^547^ or mild vacuum.^683^ In such cases, the increased drying rate again leads to more polydomain films with red-shifted color, resulting in desaturated hues or loss of color in extreme cases. Moreover, it is possible to apply a pattern to the CNC film by inducing localized temperature gradients across the substrate,^431,547^ or focused vortex air flow.^684^ However, there is not yet clear understanding regarding the impact of the drying rate during the various stages of the self-assembly process,^335^ especially before and after the point of kinetic arrest.

The observation of a complex, colored band close to the edge of the dish is often ascribed to the coffee ring effect, where differential evaporation across the liquid–air interface leads to radial capillary flows. As such, the coffee ring effect is relevant for sessile drops (as discussed in section 9.2.2) where it often leads to much greater deposition of CNCs near the contact line. However, in dish-cast films these radial flows are negligible, and the slightly greater deposition of CNCs at the dish edges can be ascribed to the meniscus at the dish walls due to pinning of the suspension. Furthermore, it is not clear why the variation in thickness across the sample should lead to a variation in pitch. Alternatively, color gradients could arise from distortion and shear near the edge of the kinetically arrested suspension due to the increasing surface area of the meniscus upon final drying. This is consistent with the observation that cocasting CNCs with a nonvolatile additive, such as PEG, reduces the edge effect in dish-cast films^648^ by preventing the final stages of compression upon complete drying.

#### 9.1.4 Application of External Fields and Forces

**Figure 76:**
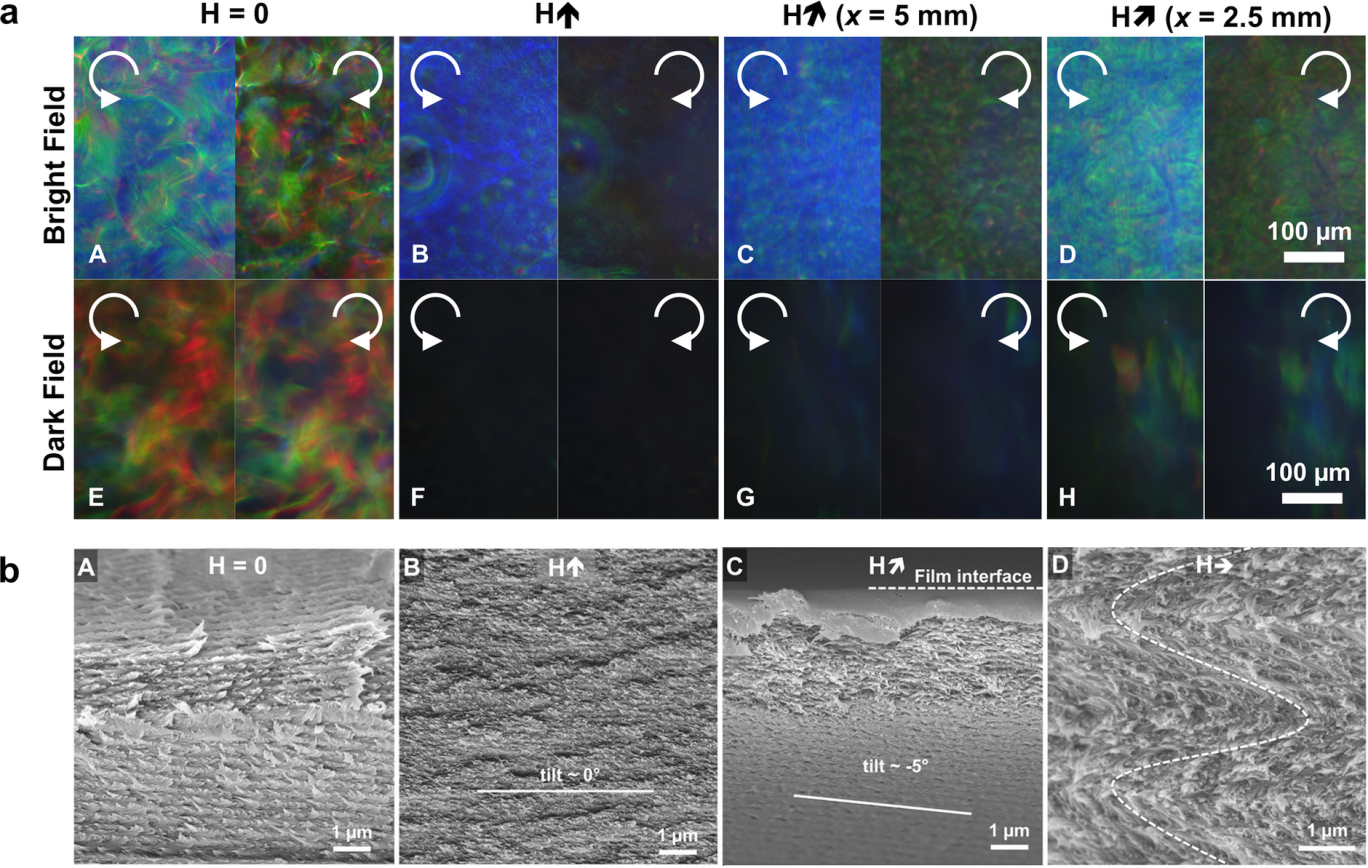
(a) Polarized optical micrographs of CNC films prepared in zero, vertical and tilted magnetic fields, imaged in reflection for bright-field and dark-field configuration and with left- and right-circular polarization filters. (b) Representative cross-sectional SEM images for the CNC films in (a), showing that the domains are aligned by the applied field. Reproduced with permission from ref (161) under Creative Commons CC-BY. Copyright 2017 The Authors.

The long-range cholesteric order within a drying CNC suspension can be further enhanced by actively applying external fields or forces. While it is known that a strong electric field can align and unwind cholesteric CNC domains in an apolar solvent (such as toluene, see section 4.4.1)^249,406^ or orientate tactoids in water,^405^ such approaches have not yet been applied while drying a suspension into the solid state. In contrast, the alignment of CNC tactoids with commercial neodymium magnets (NdFeB, μ_0_*H* ≈ 0.5–1 T) has been shown to be a highly effective route to producing well-aligned photonic films with a highly uniform visual appearance (see section 4.4.2 for mechanism).^161,413^ In particular, a vertically aligned magnetic field leads to an overall blue-shifted color with better polarization selectivity (Figure 76), due to the absence of tilted domains, which are red-shifted due to their reduced pitch compression upon drying (see section 6.1).

**Figure 77:**
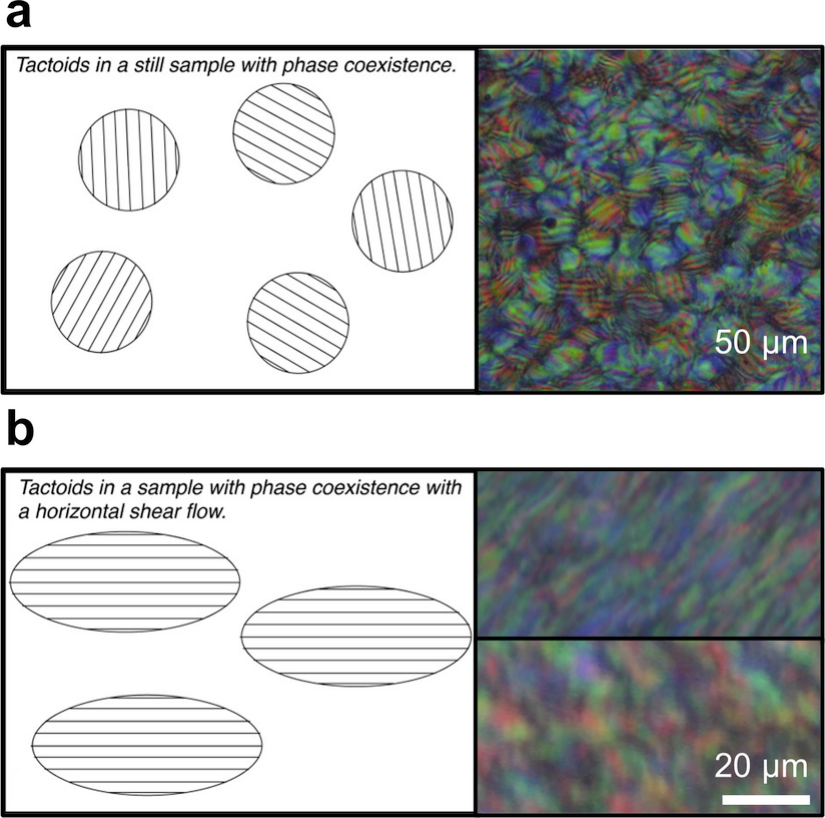
(a) The randomized sedimentation of tactoids results in a polydomain CNC film. (b) The application of orbital shear distorts and elongates the tactoids perpendicular to **m**, such that upon sedimentation they orientate vertically, leading to a more uniformly aligned film. Reproduced with permission from ref (372). Copyright 2014 John Wiley and Sons.

Finally, while shearing a cholesteric CNC suspension typically results in a nematic-like structure that does not generate a photonic response, it is possible to improve the alignment of the domains without destroying the cholesteric order by applying only a mild shear force. For example, orbital shear flow during the drying of an isotropic CNC suspension facilitates planar anchoring, leading to a more uniform photonic appearance.^372^ This is attributed to the ellipsoidal shape of tactoids formed under shear, which geometrically promotes their alignment to the substrate (Figure 77). Interestingly, shear was not found to enhance the ordering of tactoids that were present in the initial (biphasic) suspension, or for fully anisotropic suspensions.

### 9.2 Scalable Deposition Techniques

To develop photonic CNC materials for real-world applications, it is necessary to move beyond centimeter-scale dish-casting methods. This can be envisaged following three strategies: (i) scaling up the area of the film (i.e., m^2^), with the goal of exploiting existing continuous deposition methods, (ii) scaling down the area of the film (i.e., μm^2^), allowing for patterned arrays to be achieved analogous to dot-matrix printing, or (iii) employing a substrate-free approach, allowing microparticles (*cf*. pigments) to be prepared directly from an emulsified CNC suspension. The following section will discuss the current progress toward these three approaches.

#### 9.2.1 Blade Coating for Large Area Films

**Figure 78:**
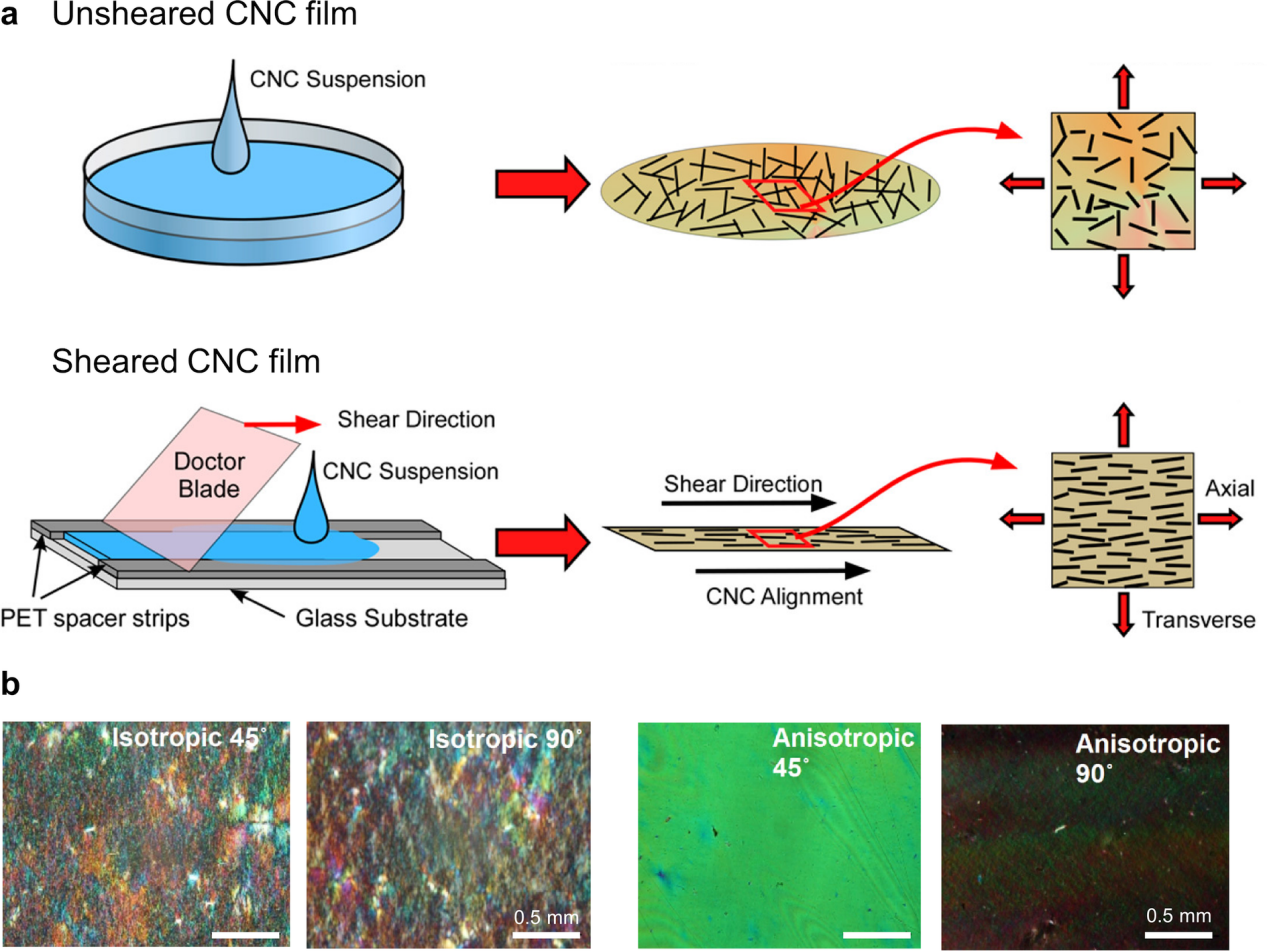
(a) Schematic of the effect of shear alignment upon a CNC suspension. Reproduced with permission from ref (688). Copyright 2013 American Chemical Society. (b) Example polarized optical micrographs of CNC films between crossed polarizers, corresponding to the geometries in (a). Reproduced with permission from ref (686). Copyright 2017 Springer Nature.

It is widely known that thin films can be deposited by dragging a doctor blade over a surface with a precisely defined gap, resulting in uniform coatings over a large area. However, such blade coating approaches can induce strong shear within the coating solution, which in the case of anisotropic nanoparticles can lead to significant uniaxial alignment. Indeed, it has been reported that blade-casting of CNCs concentrated to a gel-like state (where rotational diffusion is inhibited), results in a well-aligned film (i.e., with a Hermans order parameter *S* = 0.36, versus 0.04 for an unsheared film).^685^ Further studies correlated a high degree of alignment within sheared CNC films (reportedly up to *S* ≈ 0.95)^686^ with the presence of strong linear birefringence and anisotropic thermal expansion (Figure 78).^686−688^ Finally, it has been shown that these birefringent films (*S* < 0.65) can be produced by the industrially relevant roll-to-roll technique.^370^

**Figure 79:**
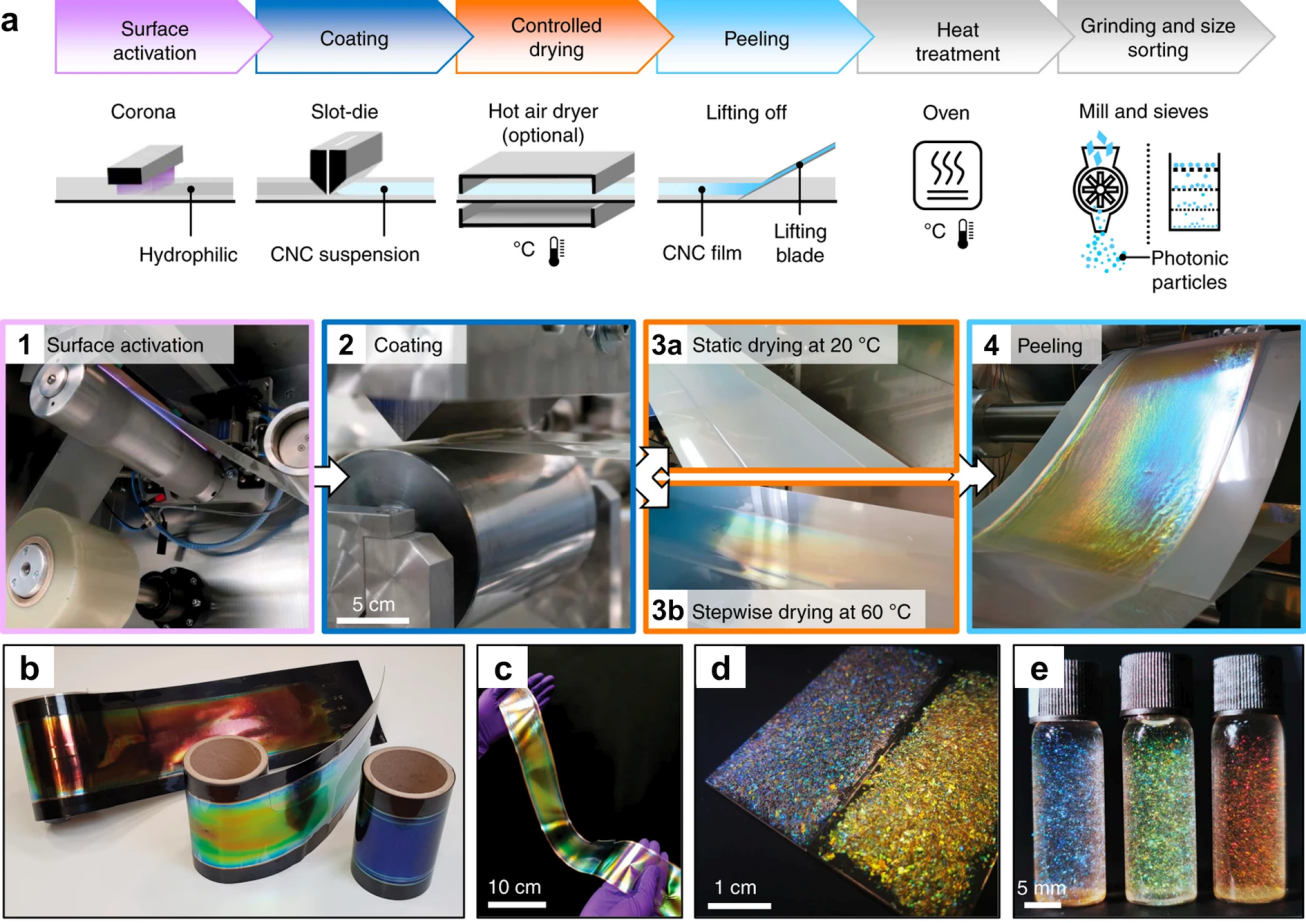
(a) Schematic showing the production of CNC glitter by roll-to-roll deposition, with example images of the key steps (1–4). (b, c) Large-scale photonic CNC films with different colors and (d, e) the resultant glitters after grinding. Reproduced with permission from ref (547). Copyright 2021 The Authors.

Alternatively, if the applied shear force is weak and/or the CNC suspension is able to relax, any uniaxial alignment can dissipate prior to reaching kinetic arrest.^285,689^ In such cases, the cholesteric phase can form as per its dish-cast analogue, resulting in a photonic CNC film as described before. While the dimensions of a dish-cast film are typically defined by the geometry of the container, photonic films cast by blade-coating are instead defined by both the coating conditions and the wettability of the substrate. For example, by pinning the spreading CNC suspension at a hydrophilic/hydrophobic boundary (via e.g. localized exposure to plasma or corona discharge), the film thickness can be defined by the coating gap,^547^ enabling vibrant photonic films with a controlled thickness (and thus reflectivity) to be reproducibly produced (Figure 79). Moreover, it was shown that this approach could be directly translated to the roll-to-roll process, allowing for the continuous production of photonic films that could be postprocessed into glitters (see section 10.2.5).^547^ To achieve this, it was crucial to reduce the typical drying time to be compatible with the translation of the web between the two rolls (i.e., from 1 to 2 days to <4.5 h in this case), while maintaining a strong photonic response. This was accomplished by optimizing the CNC suspension (i.e., taking the anisotropic phase and casting from a relatively high concentration of 6 wt %) such that the time required for the self-organization into a well-ordered cholesteric phase was minimized. Accordingly, once this was obtained, rapid drying via a hot-air blower did not significantly perturb the film structure.

#### 9.2.2 Drop-Casting for Microfilm Arrays

The evaporation of a CNC suspension confined within a sessile drop (i.e., a volume of suspension wetting a surface) introduces further complexity to the self-assembly process in terms of the dynamics of the three-phase contact line and its pinning to the substrate. In the absence of a container, the suspension initially spreads across the substrate to reach an equilibrium shape. This stage is governed by the balance of capillary and gravity forces and can be modified by both surface tension and surface chemistry. The wetting conditions constrain the maximum height of the drop and thus the volume of suspension per surface area. At the edge, the decrease in height scales laterally with the capillary length (ca. 2.7 mm for water) and leads to either a small domed drop or a larger flattened puddle, depending on the initial suspension volume (i.e., nL to μL).^690^ Upon solvent evaporation, competition between the loss of volume and the emergence of mass flows has opposing effects on the contact line displacement and leads to a variety of scenarios that strongly influence CNC deposition.

Upon deposition of a drop of aqueous CNC suspension onto a mildly hydrophilic surface (e.g., uncleaned glass), the higher evaporation rate near the edge of the drop (arising from the lower local partial vapor pressure) generates an outward radial capillary flow that results in a solid accumulation of particles. Maintaining the contact angle then favors an continued outward flow that transports further particles to the edge of the droplet (cf. coffee ring stain^682,691,692^). In the dilute regime, this flow leads to the accumulation of almost all CNCs at the periphery of the drop, with the nanorods radially aligned within the capillary flow but tangentially aligned at the contact line (i.e, orthoradially), attributed to orientation by surface tension.^428^ At higher concentrations, the deposition of CNCs at the pinned contact line ultimately leads to an asymmetric, ring-shaped deposit with a significant radial blue-shift toward the center.^430,436,677,693,694^ This can be rationalized as, in addition to inducing an uneven distribution of mass within the resultant film, these capillary flows also induce shear that can disturb any cholesteric order that is present,^695^ which can even lead to linear birefringence near the film edge (potentially enhanced by any stick-and-slip dynamics of the contact line^375^). Notably, this process can occur even when the spreading droplet is confined within a hydrophobic, nonsymmetric template, with the bounding shape influencing the mass flows and thus the local color profile.^693^ Finally, it is important to comment that on a hydrophobic surface, aqueous drops typically do not pin, resulting in much thicker CNC films without visible color.^428^ The absence of color can be rationalized by the reduced vertical compression experienced by the drying CNC suspension upon significant lateral contraction (see section 6.1), resulting in a much larger pitch than that for the equivalent dish-cast film.^338^

The simplest method to avoid color gradients within a CNC film is to suppress any capillary flows within the drop.^682^ While in principle increasing the local humidity is a good way to reduce the evaporation rate, it is in practice difficult to implement for small volumes, where the time-scale for the vapor pressure to stabilize is comparable to the evaporation time of the sample. As such, a range of alternative approaches have been demonstrated for other systems.^682,696−698^ For CNC drops, the coffee-stain effect has been suppressed by increasing the suspension viscosity (e.g., via a thickener such as glucose,^677^ or inducing gelation with electrolytes^699^), which slows deposition at the contact line and thus prevents the drop becoming pinned as it dries.^700^ Alternatively, by using two solvents of different volatility and surface tension (e.g., an ethanol-enriched atmosphere), a Marangoni counter-flow at the liquid–air interface can be induced to balance this mass transfer.^430^ However, such disturbances lead to a polydomain texture and birefringent edges. This concept was developed further by including dimethyl sulfoxide (DMSO) and a nonionic surfactant into the initial CNC suspension, which helps to reduce the surface tension, leading to more uniformly colored films.^701^ This can be rationalized by considering that the surfactant/DMSO may have inhibited pinning to the surface,^375^ allowing for the drop to additionally shrink laterally and resulting in the formation of a more uniform, but red-shifted CNC film. Alternatively, it has been proposed that a weak circular shear flow during the drying process can reduce concentration gradients, allowing for a more uniform cholesteric arrangement to form throughout the sample.^573^

Surprisingly, even in the absence of internal flows, the wettability of the substrate was shown to significantly affect the self-assembly process within a sessile drop.^336^ When drops of biphasic suspension were allowed to equilibrate on a glass substrate with a contact angle of ∼40°, it was found that the tactoids sedimented and coalesced within the droplet to grow an anisotropic phase from the glass substrate (analogous to dish-cast films). In contrast, repeating the experiment on a PTFE substrate (contact angle ≈ 100°) resulted in a preference for the anisotropic phase to initially grow from the air-droplet interface, with no preference toward the substrate. However, this behavior might originate from the distinct drop geometries that arise from these markedly different wetting angles (cf. spherical confinement within emulsified droplets).^288^

**Figure 80:**
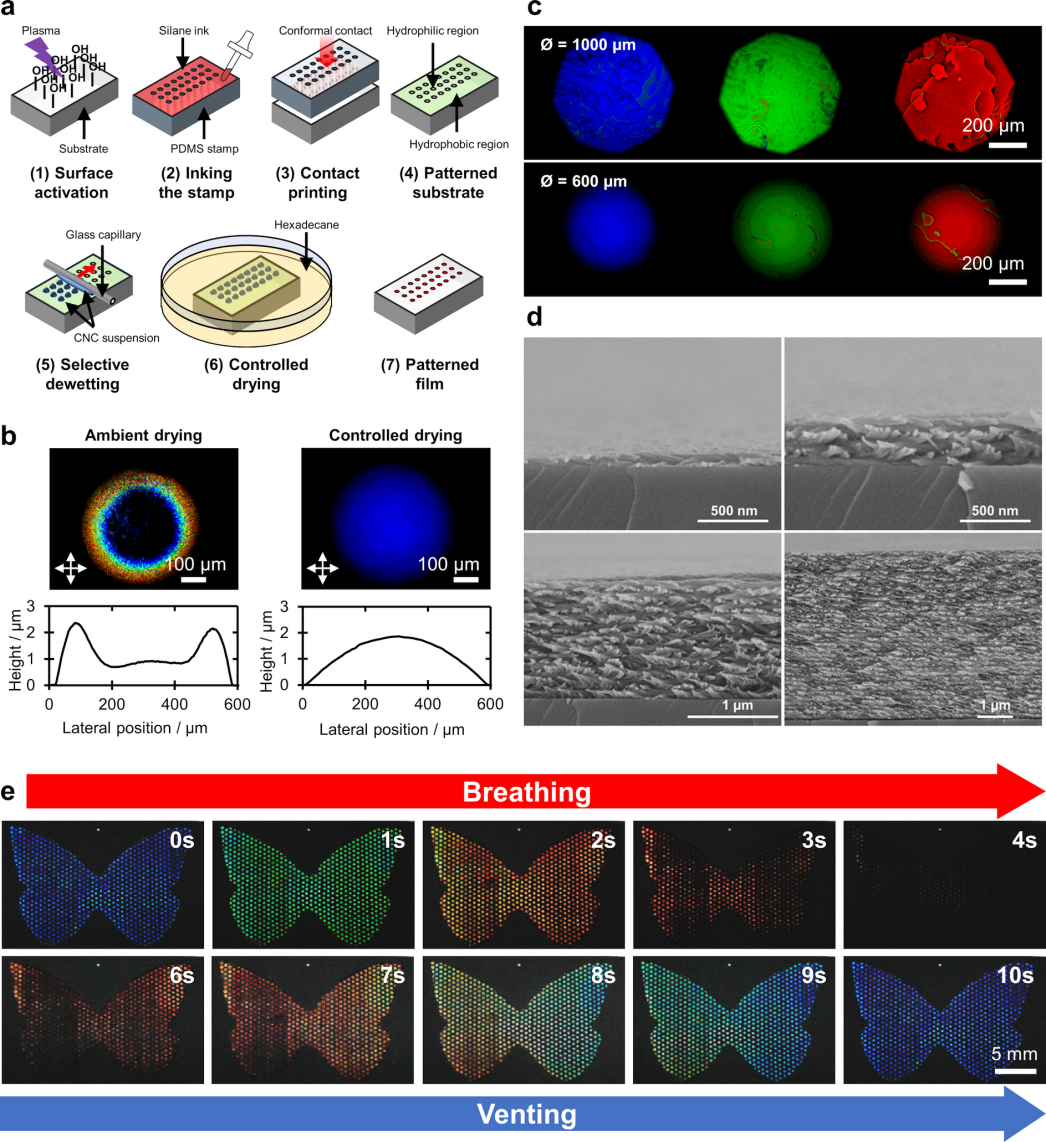
(a) Schematic of the printing process for CNC microfilms. (b) Uniform films are produced by drying under oil, which suppresses mass transfer to the periphery of the droplet (i.e., the coffee ring effect). (c) Reflection micrographs of red, green and blue microfilms with diameter, Ø = 1000 and 600 μm. (d) The highly uniform and intense color in (c) arises from the well aligned helicoidal structure, as observed in cross-sectional SEM. (e) This thin, well-aligned structure enables rapid response to changes in humidity, such as from human breath. Reproduced with permission from ref (338) under Creative Commons CC-BY. Copyright 2018 The Authors.

Finally, the ability to dry CNC suspensions within smaller sessile drops to form microfilms (i.e., diameter, Ø < 1 mm) suggests their relevance for scale-up. Drawing analogy to dot-matrix printing, arrays of CNC films can be used to coat surfaces or even produce stimuli-responsive images (Figure 80). This was achieved by combining blade-coating with a hydrophilic/hydrophobic patterned substrate (Figure 80a).^338^ Moreover, by drying under a layer of immiscible oil (e.g., hexadecane), it was shown that differential evaporation across the drop (that leads to the coffee-stain effect) could be suppressed, resulting in thin, dome-shaped microfilms with a highly uniform and saturated optical response (Figure 80b–d). Notably, the color uniformity across the microfilm arrays could be further enhanced through use of a sacrificial CNC deposition that aided pinning at the hydrophilic/hydrophobic boundary. However, translating this approach directly to commercial printers is more challenging. While it has been demonstrated that low concentration CNC formulations (0.4–2.6 wt %) can be successfully printed via a commercial inkjet printer, the rapid drying rate in such small droplets (Ø < 75 μm) resulted in disordered birefringent structures that display only interference colors when viewed between polarizers (see section 7.2.2).^702^

#### 9.2.3 Microemulsions and the Role of Geometric Confinement

Moving beyond uniaxial CNC films and coatings, the impact of geometry on the self-assembly process becomes increasingly important (see 6.2). As such, new strategies are required to achieve structural color from spherical particles or cylindrical fibers.

##### 9.2.3.1 Emulsification for Hierarchical CNC Microparticles

**Figure 81:**
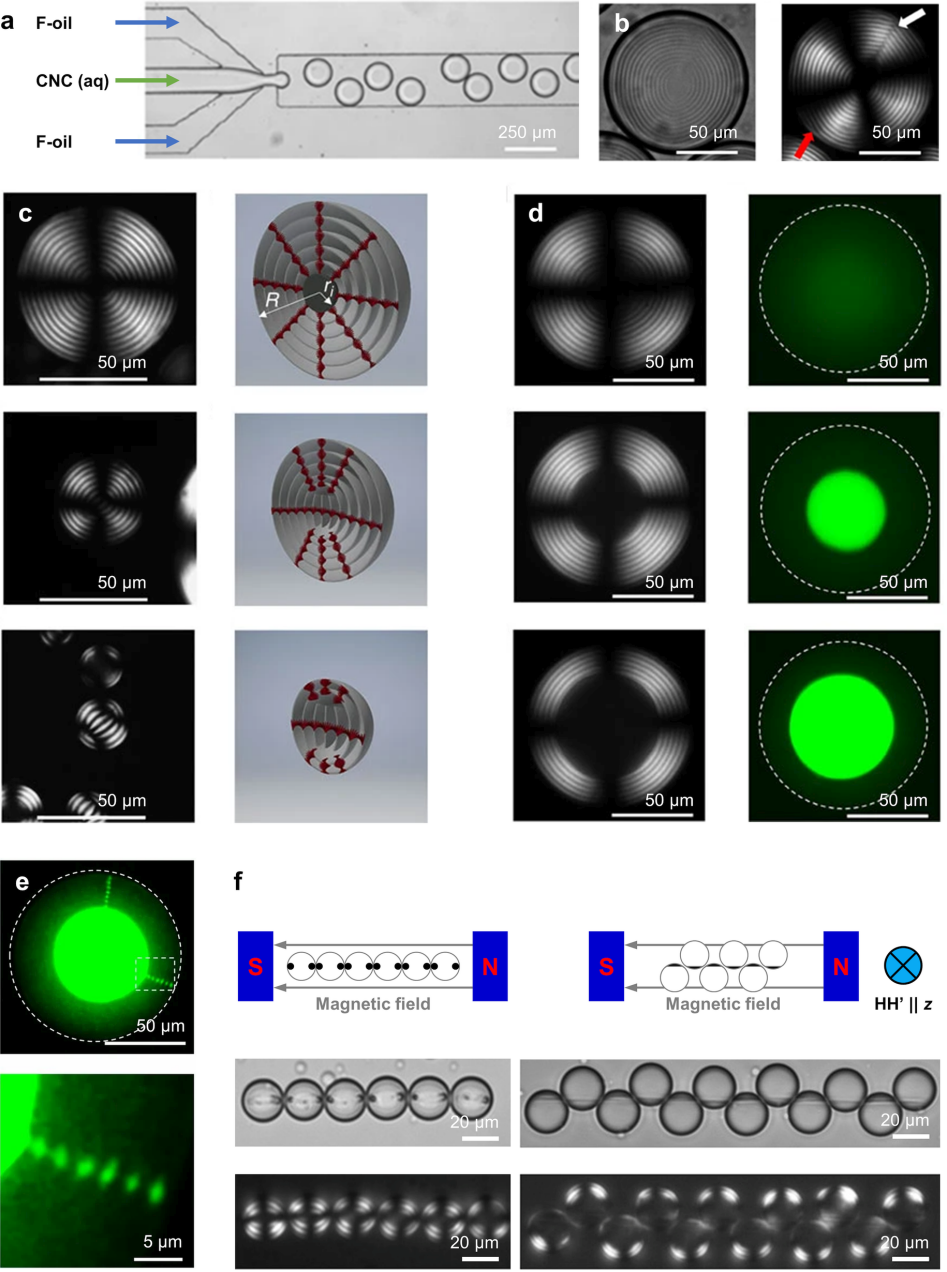
(a) Microfluidic generation of CNC microdroplets as a water/oil emulsion. (b) Optical micrographs of the Frank-Pryce-like organization within the droplet (left transmission, right cross-polarizers). White and red arrows respectively indicate radial disclinations and a local defect. (c) Size-dependence of the organization of the CNC mesophase (d) Localization of nanoparticles within isotropic core is dependent on their size. Reproduced with permission from ref (307) under Creative Commons CC-BY. Copyright 2016 The Authors. (e) Nanoparticle localization within the point defects. Reproduced with permission from ref (422). Copyright 2017 The Authors. (f) Control over the localization of magnetic nanoparticles allows for external manipulation, such as chaining. Reproduced from ref (703) with permission under Creative Commons CC BY-NC. Copyright 2019 The Authors.

The confinement of a CNC suspension within a micron-scale spherical droplet provides an substrate-free approach to cholesteric self-assembly, enabling nanostructured microparticles to be directly produced upon loss of solvent.^288,309^ Emulsions of monodisperse cholesteric CNC microdroplets in an immiscible oil can be formed using a microfluidic flow-focusing device (Figure 81a). As noted in section 4.2, in this spherical geometry the planar anchoring of the CNCs at the liquid–liquid interface enables the cholesteric phase to assemble inward. This results in a monodomain Frank–Pryce-like architecture, recognizable between crossed polarizers as a concentric fingerprint pattern superimposed with a Maltese cross (Figure 81b).^288,307^ In this ordering, the planar anchoring across the surface of the droplet results in the formation of radial disclinations that connect to a topological point-defect in the droplet center. Moreover, the lyotropic nature of the cholesteric phase combined with the lower local density in the defect regions can lead to enhanced phase separation, with the localization of isotropic phase at the center of the droplet. For smaller droplets (radius *r* < 30 μm), this isotropic core is instead replaced by a central tactoid (which becomes increasingly dominant as the radius decreases), surrounded by a concentric shell (Figure 81c), while for very large droplets (*r* > 115 μm) the long equilibration time typically results in a polydomain morphology.^307^ Notably, while a variety of small nanoparticle dopants (e.g polystyrene, gold, carbon dots or metal oxide) with diameter smaller than the average distance between CNCs can diffuse freely through the cholesteric phase, larger nanoparticles (ca. *r* = 100 nm) can be localized within the central defect (Figure 81d) or as periodic arrays along the radial disclinations (Figure 81e), offering additional functionality (Figure 81f).^307,422,703^ Furthermore, with increasing volume fraction of nanoparticle dopant, their expulsion from the cholesteric phase can enhance phase separation leading to a much larger isotropic core than expected for dilution of the CNC suspension alone.

**Figure 82:**
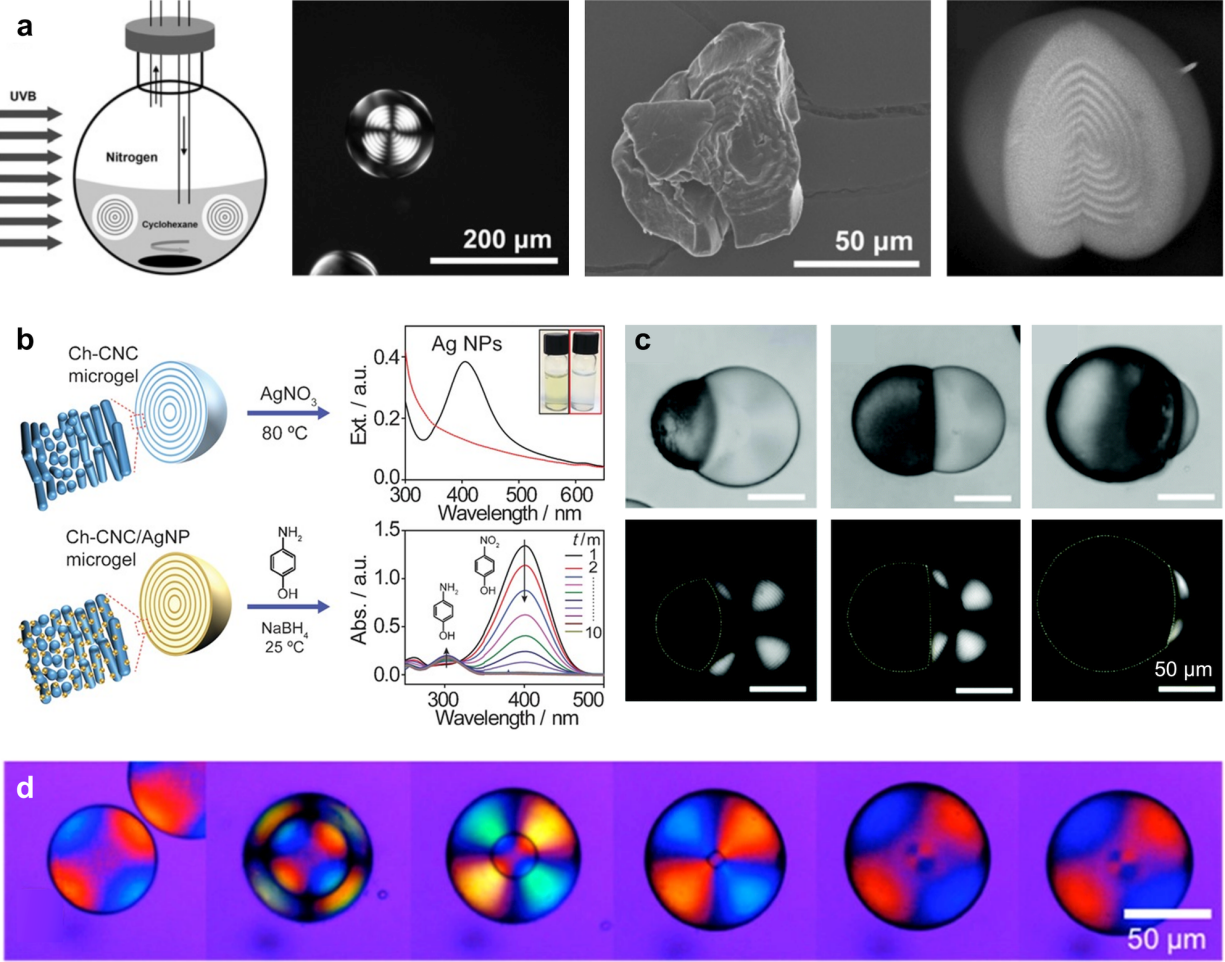
(a) Formation of hydrogel beads (i.e., microgels) via UV-polymerization of an emulsified CNC suspension. Reproduced with permission from ref (384). Copyright 2016 John Wiley and Sons. (b) CNC hydrogel beads can be used as microreactors for catalysis. Reproduced with permission from ref (704). Copyright 2016 John Wiley and Sons. (c) Compound ‘Janus’ droplets can be used to template anisotropic CNC microparticles. Reproduced with permission from ref (705). Copyright 2018 Royal Society of Chemistry. (d) Crosslinked CNC hydrogel beads reversibly swell in water. Reproduced with permission from ref (706). Copyright 2018 American Chemical Society.

Microparticles with a helicoidal internal structure have been obtained from cholesteric CNC microdroplets by two strategies, as summarized below:

i. *Cross-Linking the CNC Droplet to Form a Hydrogel*. By exploiting the rapid photopolymerization of polyacrylamide precursors,^333^ a cholesteric CNC droplet can be transformed into a gel microparticle (“microgel”), as shown in Figure 82a.^384^ While the pitch in such a hydrogel particle is far too large to reflect visible light, they can then be used as precursors to form chiral mesoporous silica microparticles (see section 10.2.3 for methodology), which may have applications in chiral separation or as scaffolds for heterogeneous catalysis. Indeed, owing to their high surface area and the presence of nucleophilic hydroxy groups and negatively charged sulfate half-ester groups, CNC microgels (here cross-linked via poly(ethylene glycol) dimethacrylate) have been shown to be effective microreactors for the synthesis of both small molecules (e.g., 4-nitrophenol) and metal nanoparticles (e.g., silver, which itself can act as a catalyst within the microgel), as shown in Figure 82b.^704^ Furthermore, by changing the dimensions of the cholesteric droplets, the morphology of the resultant microgels can be switched from bipolar to radial, resulting in their swelling in water to be anisotropic or isotropic, respectively.^704^ It is also interesting to note that such microgels can be dehydrated and rehydrated, with the degree of swelling in water (and thus the pitch of the nanostructure) tunable via both ionic strength (i.e., ion-induced polymer dehydration) and temperature (by incorporating e.g. thermoresponsive N-isopropylacrylamide). CNC microgels with nonconventional shapes can also be prepared via compound “Janus” droplets, with the demonstrated geometries ranging from a truncated sphere to a “crescent moon” (Figure 82c).^705^ This results in a distortion of the Frank-Pryce structure, with a corresponding reduction in pitch. Lastly, highly porous microgels have also been fabricated in the absence of photopolymers (and using only green oils and surfactants) by emulsifying a mixture of hydrazide-modified and aldehyde-modified CNCs within microdroplets.^706^ The resultant microgels were found to be stable to rehydration in water, with the CNCs retaining their distinctive radial architecture (Figure 82d).

ii. *Concentrating the CNC Droplet into the Solid State*. Much like for the film geometry, emulsified microdroplets of CNC suspension can be dried to directly produce hierarchical microparticles.^288^ When beginning with a dilute CNC suspension, the progressive loss of solvent (i.e., water) triggers an in situ phase transition and self-organization into a Frank–Pryce-like cholesteric architecture, which upon further drying, leads to a nanostructured microparticle with radial helicoidal order. During this process, the higher concentration near the droplet interface results in an inward nucleation and growth of the cholesteric phase, incorporating any tactoids that may be initially present. In contrast, starting with a fully anisotropic phase can result in the direct formation of a Frank-Pryce structure, assuming sufficient time is allowed for the system to reorganize. It is important to note that while the shear during droplet formation can enhance radial alignment, chaotic advection within the droplet as it flows within a microchannel typically causes any ordering to be lost. As such, it is necessary to control the rate of water loss such that self-organization into a well-ordered structure can occur prior to the onset of kinetic arrest. This can be enhanced by using a phase-separated suspension, whereby a fully anisotropic phase can be accessed at much lower concentrations, and thus viscosities.

The concentration of CNCs within a spherical geometry is also a useful tool to understand the underlying self-assembly mechanisms.^288^ Observation of a shrinking CNC microdroplet allows for monitoring of the evolution of the pitch as a function of concentration over a much wider range than can be accessed via measurement in thin capillaries. Within droplets, it was found that at low CNC volume fraction (Φ), the pitch (*p*) matches well with the bulk suspension and follows a power law of approximately *p* ∝ Φ^–1^. However, above a specific concentration attributed to kinetic arrest (Φ_KA_), the pitch scales further as *p* ∝ Φ^–1/3^. This behavior is expected when further pitch decrease arises solely from the isotropic 3D compression specific to a contracting sphere, as opposed to the 1D compression in the standard film geometry (as described in more detail in section 6). The consequence of this shallower power law is that upon complete drying of the microdroplet, the final pitch is significantly larger than for a film cast from the same suspension, thus shifting the optical bandgap beyond visible wavelengths. Notably, despite their lack of coloration, such CNC microparticles can be used as heterogeneous catalysts, by e.g. doping with titania.^707^

**Figure 83:**
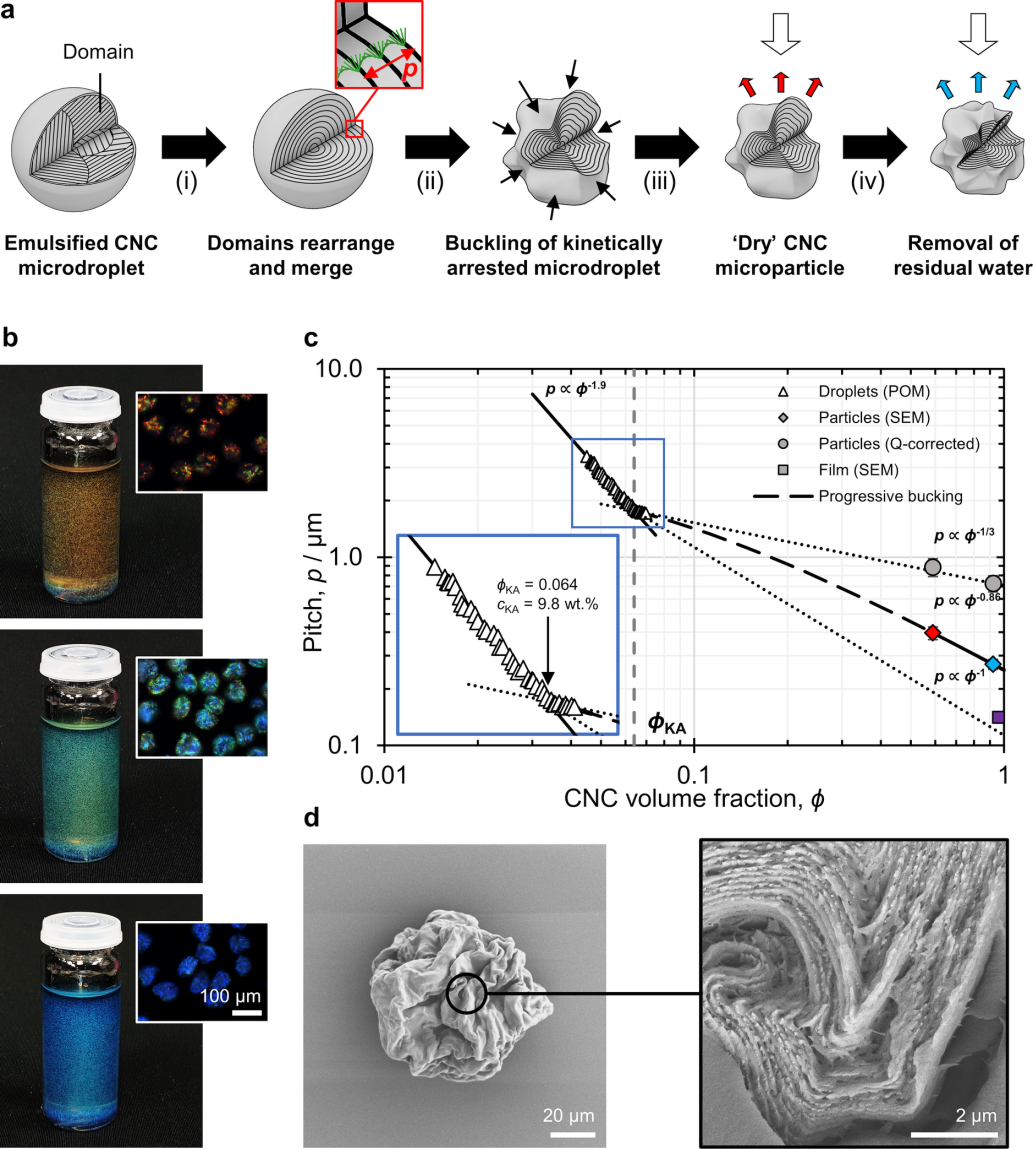
(a) Schematic of the formation of photonic CNC particles from emulsified droplets. (b) Vials containing red, green and blue pigments, with corresponding microscope images inset. (c) Pitch evolution of drying droplets, illustrating the change in power law upon kinetic arrest (KA) and the increased pitch compression following buckling. (d) SEM image of a buckled “blue” microparticle and a cross-section of the buckled surface, showing the characteristic Bouligand structure. Adapted with permission from ref (309) under Creative Commons CC-BY. Copyright 2022 The Authors.

To produce structurally colored microparticles it is necessary to overcome the reduced pitch compression that is inherent to self-assembly under spherical confinement. This was recently achieved by exploiting the interfacial buckling of kinetically arrested CNC microdroplets (Figure 83).^309^ Upon water loss, anisotropic compression of an arrested droplet can be favored through either (i) preferential compression of the cholesteric phase along the helical axis (i.e., radial > orthoradial compression), or (ii) a higher CNC concentration near the droplet interface, which can lead to a more rigid shell that resists orthoradial compression upon further volume contraction (see section 6.2). Irrespective of the precise mechanism, significant buckling of the radially aligned microparticle allows for the structure to locally collapse along the helical axis, resulting in a pitch contraction that is more analogous to that of the film geometry (i.e., *p* ∝ Φ^–1/3^ → Φ^–1^). However, the deformation of the interface associated with buckling makes further volume contraction more difficult. As it contracts, the concentric cholesteric structure undergoes several buckling events resulting in several generations of wrinkles, which are increasingly able to resist further orthoradial compression, despite the low water vapor pressure. By applying post-treatment using either a polar solvent or heating, residual water could be controllably removed, resulting in further collapse of the particle and thus reduction of the pitch. Combined, this enabled the production of vibrant red, green, and blue photonic CNC pigments (Figure 83b).^309^ Interestingly, in contrast to CNC films, the isotropic nature of these radially aligned CNC microspheres gives rise to angular independent color under diffuse illumination.

Relatedly, the evaporation of a dilute drop of CNC suspension (c ≈ 1 wt %, Ø ≈ 1.4–1.8 mm, *V* ≈ 1.5–3 μL) has also been investigated when levitated in air, as can be achieved in a node of a stationary ultrasound wave.^708^ The substantial shear that is applied to the droplet as it levitates, causes the drop to spin and tumble about its stable position as it dries, resulting in a the formation of a smooth, spherical microparticle with a highly buckled, helicoidal internal nanostructure. This process results in a gradient in helicoidal pitch, with smaller values toward the particle center. The smallest pitch values were similar to that of the equivalent film, rather than that expected for an isotropic sphere,^288^ which is consistent with the expected effect of buckling.^309^

##### 9.2.3.2 Extrusion for Hierarchical CNC Fibers

**Figure 84:**
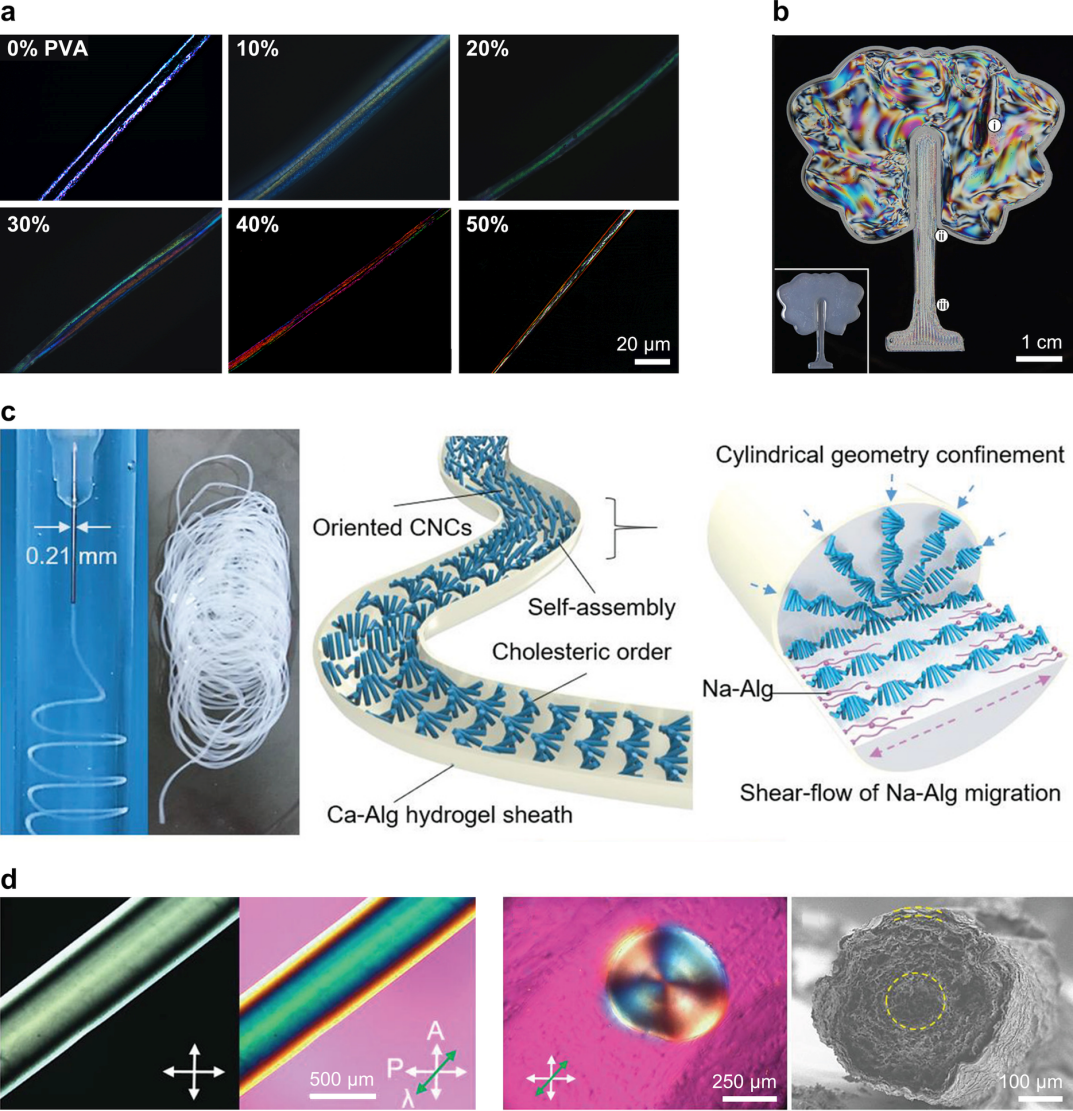
(a) Uniaxially aligned CNC fibers present birefringent color when viewed between crossed polarizers. By increasing the proportion of the additive from 10 to 50% PVA, the birefringence color can be red-shifted. Reproduced with permission from ref (709). Copyright 2018 IOP Publishing. (b) Birefringent pattens and images can be printed by controlling the degree of CNC alignment within an extruded filament. Reproduced with permission from ref (710). Copyright 2018 American Chemical Society. (c) Extrusion of a CNC suspension within an alginate sheath allows time for it to relax from an initially shear- aligned state to form concentric radial ordering in cross-section (cf. Frank Pryce structure), combined with a tilt of the helical axis along the longitudinal direction. (d) This ordering was retained in the resultant CNC fiber, as confirmed by longitudinal and cross-sectional imaging using POM and SEM. Reproduced with permission from ref (711). Copyright 2020 John Wiley and Sons.

Extrusion of CNC suspensions can be used to produce fibers with a microscale cross-section. However, achieving a photonic response using this approach is challenging, as it combines the complexities of both blade-cast films and emulsified droplets. For example, wet-spinning of concentrated CNC/PVA suspensions followed by coagulation in ethanol was reported to produce fibers with birefringence colors visible between crossed polarizers.^709^ In this cylindrical geometry, shear alignment of the kinetically arrested CNC suspension by the extrusion nozzle results in uniaxial, longitudinal alignment of the CNCs within the filament. Upon loss of water, buckling resulting in flattened, twisted fibers with fine surface wrinkles. Similar to that of shear-aligned films, the fibers had a Hermans order parameter of *S* = 0.36. Increasing the amount of PVA doping resulted in greater disorder between the CNCs (e.g., *S* = 0.19 with 50 wt % PVA), leading to a decrease in overall birefringence and thus a change in the perceived color of the fiber (Figure 84a). This concept of tuning the degree of CNC alignment within a filament has also been used to create birefringent images using an extrusion-based 3D printer, with the alignment controlled via nozzle flow and the ink rheology (Figure 84b).^710^

Alternatively, hydrated fibers containing a CNC core encapsulated within a calcium alginate hydrogel sheath can be produced (Figure 84c).^711^ Initially, the CNCs are again longitudinally aligned by the shear upon extrusion, however the formation of the alginate hydrogel at the interface acts as a barrier, allowing for the colloidal CNCs to partially relax into a radially aligned cholesteric, with the domain axis also tilted relative to the fiber axis following the extrusion direction (giving rise to linear birefringence). By doping the initial CNC suspension with 5% glucose, the fibers could be dried in air without significant buckling or uncontrolled deformation. However, it is important to stress that no fibers with structural color under unpolarized illumination have been reported to date. Given that approaches to produce CNC pigments that reflect at visible wavelengths have now been realized,^309^ the next challenge is to translate this understanding to the fiber geometry to unlock new applications, from sustainable filaments for 3D printing^712^ to smart textiles.^713^

### 9.3 Alternative Pathways to Photonic Films

The self-assembly of a colloidal CNC suspension into a photonic film is driven by an increasing CNC concentration that forces the nanorods to pack increasingly close to each other, first inducing a liquid crystal transition and later triggering kinetic arrest and undergoing geometric compression. While this process is typically driven by the evaporation of water, other approaches to concentrate a CNC suspension into the solid state have also been explored, as summarized in this section. Note that methods that disrupt the cholesteric order (e.g., spray-drying, spin-coating, flocculation, or 3D-printing) will not be discussed here and we instead direct the reader to a relevant review on the topic.^714^

#### 9.3.1 Vacuum Filtration

**Figure 85:**
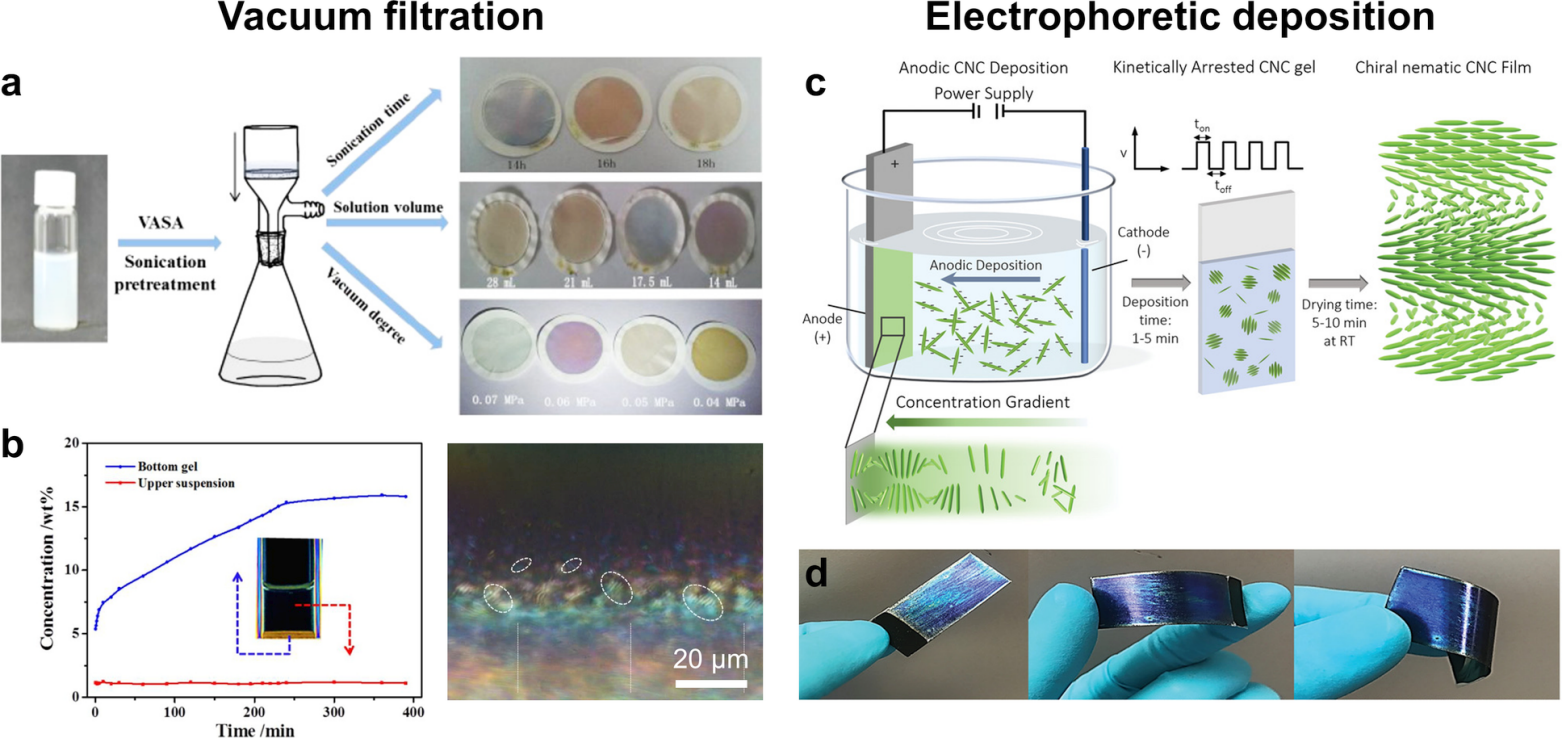
(a) Schematic and photographs of photonic CNC films prepared by vacuum filtration of a CNC suspension. Reproduced with permission from ref (715). Copyright 2014 American Chemical Society. (b) Plot showing the concentrations of the filter cake and supernatant during vacuum filtration (left) and an optical micrograph showing the formation of tactoids and their incorporation into the cholesteric filter cake (right). Reproduced with permission from ref (718). Copyright 2020 Elsevier. (c) Schematic of the electrophoretic deposition process and (d) an example photonic film deposited onto a flexible substrate. Reproduced with permission from ref (724). Copyright 2022 John Wiley and Sons.

Vacuum filtration of a CNC suspension has been reported to produce photonic CNC films on a much faster time scale than by evaporation (several hours instead of days) and with increased visual uniformity (arising from reduced edge effects etc.).^715^ In this method a dilute CNC suspension is filtered on a membrane with very small pores (∼100 nm) to form a CNC “filtration cake” (i.e., a wet gel) with cholesteric order, which can be subsequently dried to yield a photonic film (Figure 85a). While it is unsurprising that the pitch and thus the color of the film is dependent upon the initial CNC suspension (e.g., electrolytes, ultrasonication), it is perhaps less expected that the casting volume and degree of suction applied can influence the color. However, both of these factors affect the rate of local concentration increase for the CNC suspension during cake formation. Furthermore, it is interesting to note that electrolytes are removed with the filtrate and thus do not concentrate in concert with the CNCs during self-assembly (cf. dish casting), which may have implications for the evolution of the cholesteric pitch and the point of kinetic arrest. Lastly, vacuum filtration can unlock additional fabrication strategies: (i) the shorter time scales allow for the formation of photonic composites from metastable nanoparticle suspensions (e.g., graphene oxide to enhance contrast),^716^ or (ii) the ability to perform sequential filtration allows for the still hydrated cholesteric filter cake to be infiltrated with additives (e.g., ionic liquids to enhance plasticity).^717^

The mechanism leading to a well-ordered helicoidal film has been since investigated,^718^ which revealed that during filtration a birefringent CNC phase grows from the membrane interface (with increasing concentration over time), while the upper suspension remains at the initial CNC concentration. This is in stark contrast to dish casting, where an evaporation-driven increase in global concentration leads to the widespread growth and nucleation of tactoids that then subsequently sediment to form an anisotropic layer (see section 3.4). Photopolymerization of the CNC suspension with polyacrylamide (PAAm) during various stages of the filtration process allowed for the growth of the birefringent phase to be probed in cross-section (Figure 85b). This revealed that the nucleation of tactoids is indeed still occurring, but is localized near the filter, where there is a sharp gradient in local CNC concentration. The shallow depth of this biphasic region triggers the continuous formation of small tactoids that are rapidly incorporated into the growing cholesteric phase. Over time, this results in the formation of a cholesteric filter cake containing small domains, which can readily align and merge to remove defects (*cf*. graphene oxide nanosheets^719^). Finally, the presence of a disordered region close to the filter can be attributed to the initially high filtrate flux rate, which does not offer sufficient time for self-assembly to occur.

As a side note, casting a photonic CNC film upon a hydrophilic, porous substrate (such as wood, paper or ceramic),^678,680,720,721^ can be seen as a fusion of these two approaches to concentrate a CNC suspension. In such systems, there is expected to be an initial loss of solvent into the substrate (cf. vacuum filtration); however, once this is saturated, further water loss occurs primarily via evaporation. The balance between these two processes depends on the volume of the suspension relative to the porosity and permeability of the substrate, as well as its dimensions and any anisotropy (especially for wood). Consequently, such films can suffer from an accelerated initial rate of concentration, leading to potentially both a shorter time for assembly prior to kinetic arrest and also reduced edge effects due to weaker Marangoni flows. This can result in the bottom of the film being more disorganized, while larger, more well-aligned domains are found further from the substrate.

#### 9.3.2 Electrophoretic Deposition

Electrophoretic deposition (EPD) is a rapid and versatile coating technique that exploits the movement of charged particles in suspension under an electric field, resulting in coatings with high microstructural homogeneity and tailorable thickness. As an alternative method to deposit photonic films, EPD avoids the disorder caused by induced flows upon solvent removal and the need for a planar substrate.^722^ It was first demonstrated that CNCs can be electrodeposited onto the (positively charged) anode, with the thickness of the resultant disordered film built up by further cycles of EPD (up to 7 cycles: *E* ≈ 2 kV m^–1^, *t*_ON_ = 60 s).^723^ More recently, it has been shown that pulsed EPD (120+ cycles, *E* ≈ 0.4 kV m^–1^, *t*_ON_ = 0.1 s, *t*_OFF_ = 1 s) can be used to produce photonic CNC films (thickness ≈ 2 μm) in under 10 min (Figure 85c,d).^724^ The electrophoretic concentration of CNCs near the anode led to the accumulation and deposition of an aligned helicoidally structured CNC gel. Subsequent, rapid drying of this gel in air resulted in photonic films. As with dish-casting approaches, the color of the produced photonic films was shown to be tunable via the CNC source or by pretreating the suspension prior to deposition (e.g., ultrasonication, electrolytes). Notably, this approach was shown to be compatible with in situ deposition of gold nanoparticles, resulting in films with additional plasmonic properties.

### 9.4 Summary

The method used and the degree of control applied during the production of a solid photonic material from a CNC suspension can have a dramatic effect on the final visual appearance. As such, it is important to holistically consider the key parameters that determine the color. This will not only enable reproducibility and comparability between different academic studies and across multiple laboratories, but also to be able to understand and predict new challenges upon switching to larger-scale or continuous fabrication processes, where parameters such as the slow drying rate required for self-assembly can become a constraint.

While most studies aim to produce well-ordered photonic CNC films, it is also interesting to consider if perfect ordering is in fact always desirable. For colorant applications, angle-independent color (i.e., scattering) may indeed be more useful than mirror-like reflection across only a small range of angles. This prompts a deliberation as to whether the direct reflection from a CNC film is the best approach to color a surface, or whether greater attention should be given to exploiting the so-called negative color (i.e., the light transmitted through the CNC film), which can be efficiently backscattered with minimal iridescence when the CNC film is on a white rather than a dark substrate (see Figure 45 and 93).^566^

## 10 Enhancing Functionality for Photonic Applications

The versatility of CNC-based materials coupled to their biocompatibility offers a broad range of applications, from sustainable colorants or disposable colorimetric sensors to more sophisticated uses, such as anticounterfeiting. Furthermore, by incorporating additives into these photonic materials, their range of functionality can be expanded. This can be achieved by a variety of approaches, with common methods to make a composite film including coassembly within the helicoidal structure, doping with nanoparticles or lamination with other materials.

### 10.1 Co-Assembly in Photonic CNC Films

The simplest method to enhance functionality is by including a water dispersible, “noninteracting” additive into the initial CNC suspension (e.g., glucose,^436,725−728^ glycerol,^680,729,730^ latex^731−733^). Upon casting a film, the presence of a nonvolatile additive prevents the CNC concentration from reaching 100 vol% and therefore reduces the final compression of the CNC mesophase (see section 6.1). As a consequence, the film pitch is larger than that found for an additive-free film, resulting in a red-shift in the reflected color and, depending on the system, a perturbation on the domain alignment.^416^ Furthermore, such additives can also act as a binder or plasticizer, reducing significantly the brittleness of the resultant films, or act as a hygroscopic agent that can facilitate the reswelling of the film, relevant for some postprocessing strategies,^725^ or the use of CNC films as humidity-responsive hygrochromic sensors.^680,726,729,732^ Lastly, while the inclusion of an additive typically results in a red-shift in the dry state, it can also influence the equilibrium pitch of the cholesteric suspension, as discussed in section 8.3.2, and as such the self-assembly pathway is not always trivial.

**Figure 86:**
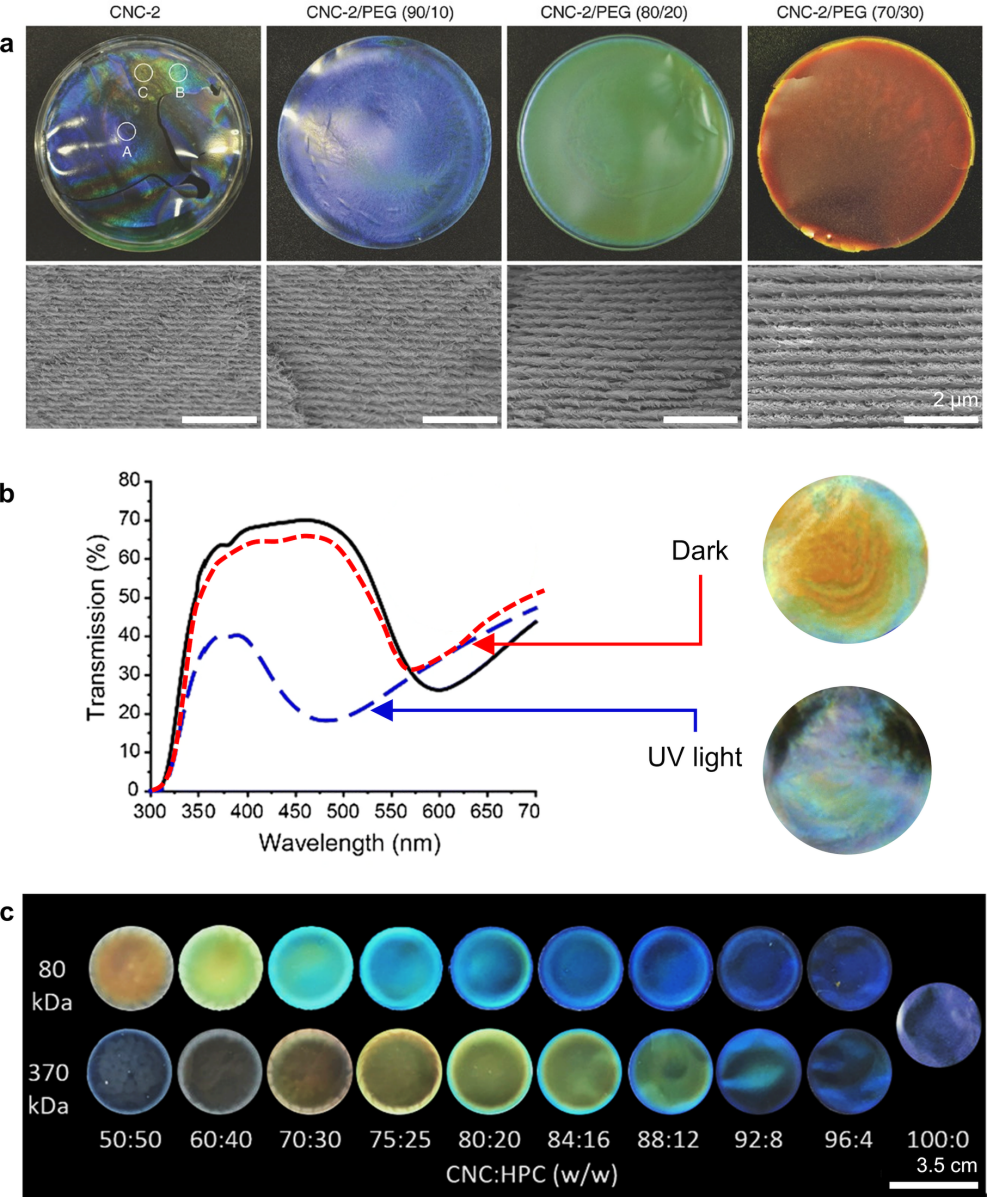
(a) Increasing the proportion of the hydrophilic polymer polyethylene glycol (PEG) within the CNC film results in a strong red-shift in the color combined with a more uniform appearance, as evidenced by photographs (top) and cross-sectional SEM (bottom). Reproduced with permission from ref (648). Copyright 2017 John Wiley and Sons. (b) PEG can photodegrade upon exposure to sunlight, which over time results in a collapse of the nanostructure with a corresponding blue-shift in appearance. In this example, one film was exposed to UV light for 6 weeks (blue dashed line), while the control film was kept in the dark (red dashed line). The black line corresponds to the initial appearance. Adapted with permission from ref (693) under Creative Commons CC-BY. Copyright 2018 The Authors. (c) Polymeric cellulose derivatives, such as HPC, are resistant to photodegradation and as such make them a more robust and sustainable alternative for tuning the color of CNC films. Note the extent of the red-shift of the color is dependent on both the loading and the molecular weight of the additive. Reproduced with permission from ref (746). Copyright 2020 American Chemical Society.

A widely explored example of such an additive is poly(ethylene glycol) (PEG), which can be incorporated up to 30–40 wt % into the dry CNC film, allowing for near-linear tuning of the photonic bandgap across the visible spectrum through the initial formulation (Figure 86a).^648,649^ Moreover, the resulting films typically display a more uniform appearance, which is attributed to the presence of smaller domains (that can assemble faster) and reduced surface tension (minimizing edge effects upon drying). At high molecular weights and/or high loading ratios, polymeric additives can also act as depletants (see section 8.3.2), but also present a lower solubility. This can favor their microphase separation into the isotropic phase, or their crystallization beyond their solubility limit,^648^ with their accumulation acting to stabilize disclination lines and grain boundaries between cholesteric domains. As such, with increasing additive loading, the efficiency to red-shift the color of the film not only decreases, but also the individual domains will become increasingly smaller and more disordered. Notably, this strategy has been exploited to produce CNC laminates with distinct changes in pitch, achieved by depositing layers with different loadings of additive, as reported with PVA.^734^

Thin photonic CNC films are notoriously brittle, which can hinder their application. However, additives such as PEG can acts a plasticizer, resulting in highly flexible films with reduced residual stress from the drying process.^735^ Furthermore, the incorporation of plasticizing additives can endow the film with additional functionality. For example, while pure CNC films adhere strongly to freshly cleaned glass, they typically lift off hydrophobic surfaces during drying, resulting in warped films. By including a small amount of amphiphilic PEG within the film (ca. 5 wt % dry mass), a broad compatibility with a wide range of substrates can be achieved, enabling the production of laminates.^649^ Notably, the presence of a hydrophilic polymer also endows the composite films with a greater sensitivity to ambient moisture, with the composite films found to significantly swell when stored at high humidity (Δλ ≈ 100 nm for 0–80% RH,^648^ versus *Δλ* < 50 nm for pure CNC films^338,648,736^). This property has since been extensively exploited to produce colorimetric humidity sensors using a variety of hydrophilic additives (e.g., PEG,^737^ PVA,^738^ PNIPAM,^739,740^ PAA,^741^ which are often cocast with glycerol to enhance the responsivity) or for distinguishing between organic solvents (e.g., PVP^742^ or alcohols^743^). Conversely, coassembly with UV-cross-linkable poly(ethylene glycol) diacrylate (PEGDA),^744^ or combining a plasticizer with a cross-linker such as glutaraldehyde,^745^ allows for the CNC films to become resistant to swelling, even in water. Finally, such additives have even been shown to delay the onset of thermal degradation in CNC films,^650,739^ potentially by acting as a partial oxygen barrier.

While formulating with a noninteracting additive may seem like a much more straightforward approach to tune the photonic response of a CNC film than previously described methods (e.g., ultrasonication or electrolytes, see sections 8.2.2 and 8.3.1), it can also introduce some complications. Unlike cellulose derivatives, PEG is well-known to photodegrade into volatile ethylene glycol, which in the context of a photonic CNC film results in a blue-shift of the visual appearance over time due to the vertical collapse of the helicoidal structure (Figure 86b).^693^ Furthermore, the incorporation of synthetically derived PEG removes the desirable “all-cellulose” descriptor that makes such biosourced materials attractive as a sustainable replacement to conventional colorants. A recent solution to this has been to instead cocast with the water-soluble cellulose derivative, hydroxypropyl cellulose (HPC).^746−748^ While HPC itself is known to produce cholesteric mesophases with structural color at concentrations above ca. 60 wt %,^749^ when formulated at low concentration into a CNC suspension it acts as a nonvolatile plasticizer (cf. PEG). HPC therefore provides a direct route to tune the photonic response and enhance the mechanical properties of a photonic CNC film, while maintaining a fully cellulosic composition (Figure 86c). However, such films show increased disorder with increasing HPC content (and HPC molecular weight), evidenced by the increasing presence of fingerprint textures on the surface of dry films and a more matte-like macroscopic appearance. Again, this appearance can be attributed to smaller, misaligned domains arising from the increased viscosity of the CNC/HPC suspension, inhibiting reorganization and fusion of the tactoids prior to kinetic arrest. Furthermore, by employing methacrylic anhydride, the HPC within the composite films can be cross-linked, again endowing them with much improved water stability.^746^ Other notable naturally derived additives for coassembly include other simple polysaccharides (e.g., dextran, pullulan, xylan, glucan),^727,750^ starch (which can be cross-linked by tannic acid),^751^ lignin nanoparticles,^752^ amino acids,^753^ or structural proteins (e.g., silk fibroin).^660^ Finally, it is important to make a distinction between these additives and cocasting mixtures of CNCs from different sources, where direct colloidal interactions can influence the self-assembly pathway.^89^ An extreme example is doping with tunicate-sourced CNCs; these high aspect ratio nanocrystals were reported to form cholesteric phases with very small pitches (corresponding to UV reflection in the solid-state) and thus when blended with CNCs from wood or cotton, were reported to have a strong blue-shifting effect.^754^

Given that CNCs are stabilized in suspension due to their surface charge, the effect of formulation with charged functional additives is more complex. For example, it has been shown that when a cationic polymer (e.g., PEI) is mixed with negatively charged CNCs it can destabilize the suspension, while in contrast an anionic polymer (e.g., PAAS) can be successfully used for tuning the color of a photonic film.^650^ Unlike the red-shift observed with increasing loading of neutral polymers, such as PEG, addition of PAAS led to a blue-shift at comparable loadings (<40 wt %), which was attributed to depletion, although the associated increase in ionic strength is also expected to contribute (see section 8.3.2).

Notably, when only a small amount of cationic polymer is added (e.g., ≪ 2 wt % PDDA relative to CNC) the destabilization of the CNCs instead leads to an earlier kinetic arrest, resulting in highly polydomain films that displays desaturated, noniridescent color.^730,755^ Alternatively, inclusion of a zwitterionic surfactant into the aqueous CNC suspension gives rise to a similar red-shift to that observed for neutral additives,^756^ which could be attributed to the surfactant instead forming “noninteracting” micelles within the cholesteric suspension. As such, this can be seen as analogous to when CNC films are cocast with small latex nanoparticles (e.g., *D*_hyd_ ≈ 50 nm).^731^ However, when large, cylindrical micelles (*D*_hyd_ ≈ 150 nm) were instead incorporated into a cholesteric CNC suspension, they were found to disrupt the long-range ordering of the assembling tactoids (in terms of pitch and orientation), resulting in films that displayed broadband chiral reflection.^757^ Again, a similar effect was observed upon doping with large nanoparticles (*D*_hyd_ ≈ 150 nm), which partition into the isotropic phase,^758^ followed by in situ cross-linking with hexamethylenediamine.^731^

It is important to note that the above discussion considers only aqueous CNC suspensions. While this is desirable from an environmental point of view, it significantly limits the range of additives to those that are hydrophilic or sufficiently charged to disperse in water. Alternatively, it has been shown that neutralized CNCs can be freeze-dried and redispersed into hydrogen-bond forming polar organic solvents (e.g., DMF), which allows for coassembly with common polymers, such as polystyrene and polycarbonate.^759^ Again the films were reported to red-shift with the proportion of additive or blue-shift with the concentration of electrolyte (e.g., LiCl), although the hydrophobicity of the polymer also plays a larger role in the final color.

**Figure 87:**
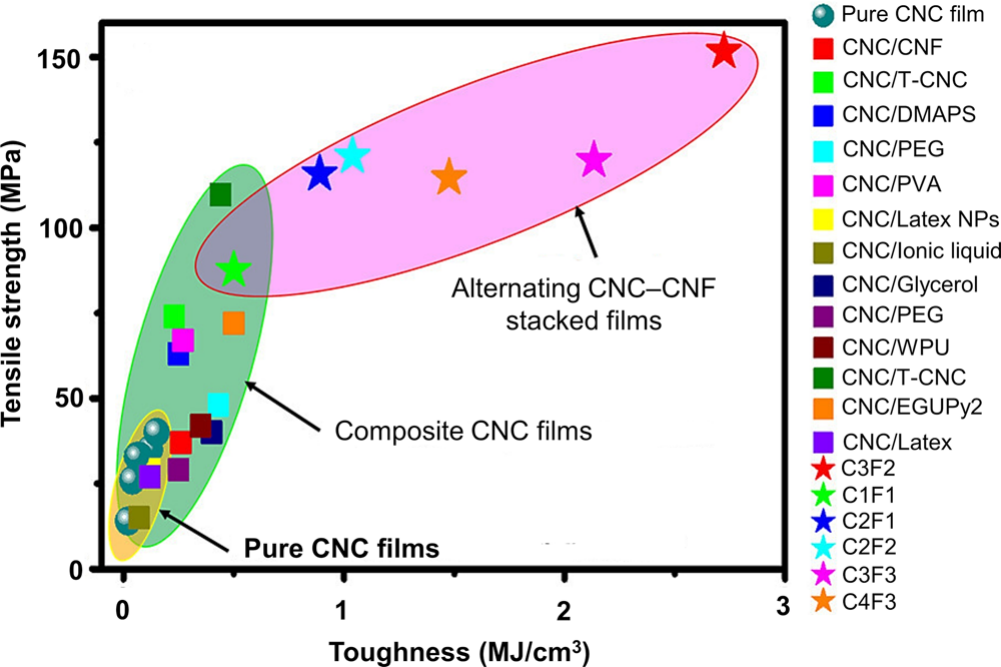
Ashby plot of tensile strength versus toughness for photonic CNC-based films. The yellow, green, and pink regions represent pristine CNC films, CNC-based composite chiral films, and alternating CNC/CNF stacked films, respectively. Adapted with permission from ref (760). Copyright 2020 American Chemical Society. The stacked CNC–CNF films are introduced in more detail in section 10.2.4.

A major drawback of photonic CNC films are their inherent fragility, despite being composed of one of the stiffest natural crystalline materials (see section 2.3.5).^761,762^ While the local alignment of the CNCs within such films can result in improved hardness,^763^ the lack of a matrix to bind the CNCs prevents the desirable mechanical properties of individual CNCs to be transferred to the macroscale. As such, there has been considerable investigation into the use of additives to enhance the mechanical properties of composite CNC films (Figure 87).^734,750,751,764,765^ In general, it has been found that while the ductility of the CNC films may be improved upon inclusion of a plasticizer (e.g., PEG), the enhancement comes at the cost of the elastic modulus. Notably, the ductility of such composite films can offer additional functionality, with the embossing of patterns demonstrated by locally compressing the pitch in CNC films doped with melamine–urea–formaldehyde.^766^ An alternative approach to overcome this issue is to employ supramolecular interactions (e.g., hydrogen bonding).^767^ By casting CNC films with a copolymer of 2-ureido-4-pyrimidinone methacrylate (i.e., a 4-fold hydrogen bonding moiety) and oligoethylene glycol methacrylate, physically cross-linked photonic films can be produced that are not only stable in water, but offer enhanced ductility and higher toughness that can be tuned independently to the visual appearance. Finally, it has been reported that doping the CNC suspension with much longer nanocrystals, such as those extracted from tunicates (*L* ≈ 1–1.5 μm), can significantly enhance the overlap between CNCs resulting in an improvement in all aspects of mechanical performance (even rivaling natural composites).^754,768^

CNCs can also be coassembled with a number of hydrogels, including polyacrylamide, poly(*N*-isopropylacrylamide), poly(acrylic acid), poly(2-hydroxyethyl methacrylate), and poly(ethylene glycol methacrylate), to create photonic films responsive to solvent content, pH or salt.^17^ Furthermore, it is interesting to note that high water content CNC hydrogels can be produced by interrupting the drying process prior to forming a film (e.g., by photo-cross-linking with PAAm),^333^ a technique widely used in colloidal sciences.^447^ While the pitch in these materials is typically too large for structural coloration, cholesteric CNC hydrogels have found use as a tool to directly interrogate the self-assembly process under different conditions and for a range of geometries, including planar films,^333^ cylindrical capillaries,^380,769^ and spherical droplets.^384^

Finally, light-absorbing additives can be included within helicoidal CNC films (e.g., dyes and pigments). Additives with broadband absorption in the visible range, such as carbon black, graphene,^770^ or polydopamine,^275,771^ can be used to enhance the contrast of the structural coloration, as explained in section 7.4.1. Alternatively, the incorporation of complementary pigments (e.g., gold nanoparticles) can suppress scattering from one spectral region without influencing the primary reflection.^554^ Beyond the visible range, UV-absorbing additives (e.g., lignin) can be incorporated into photonic CNC films to offer applications in UVB shielding.^752,772^ As a final note, functional optical dopants (e.g., plasmonic or luminescent nanoparticles) have also been incorporated into helicoidal CNC films, as discussed in section 10.4.

### 10.2 Post-Processing of Photonic CNC Films

The postprocessing of photonic CNC films allows their visual properties to be further tuned or the range of functionalities to be expanded. The simplest form of post-treatment is to chemically modify the CNCs once the photonic film has been formed. For example, redispersion of CNC films upon immersion in water can be inhibited by (i) desulfation by heating (e.g., > 150 °C),^602,625,773^ (ii) mercerization in strong base (e.g., 16% NaOH, 70 °C), where partial and transient dissolution of the CNCs (combined with desulfation) results in a blue-shift of the film and improved mechanical properties,^774^ or (iii) cross-linking with glutaraldehyde (either directly^775^ or via a plasticizer such as PAAm^740^), which can unlock further processing with aqueous reagents. Alternatively, the tendency of CNC films to swell with water, which can be enhanced in the presence of a nonvolatile additive (e.g., glucose^725^), can be exploited to postinfiltrate the films with a broader range of additives that may not be compatible with a coassembly strategy, such as polydopamine (a broadband absorber that enhances visual contrast),^275,771^ structural proteins (e.g., silk^776^), elastomers (enabling mechanoresponsive changes in birefringence^777^ or structural coloration^725^), or latex (allowing for selective patterning with hygroscopic salts^732^). In the following sections, the most common postprocessing methods are introduced in greater detail.

#### 10.2.1 Heat Treatment

**Figure 88:**
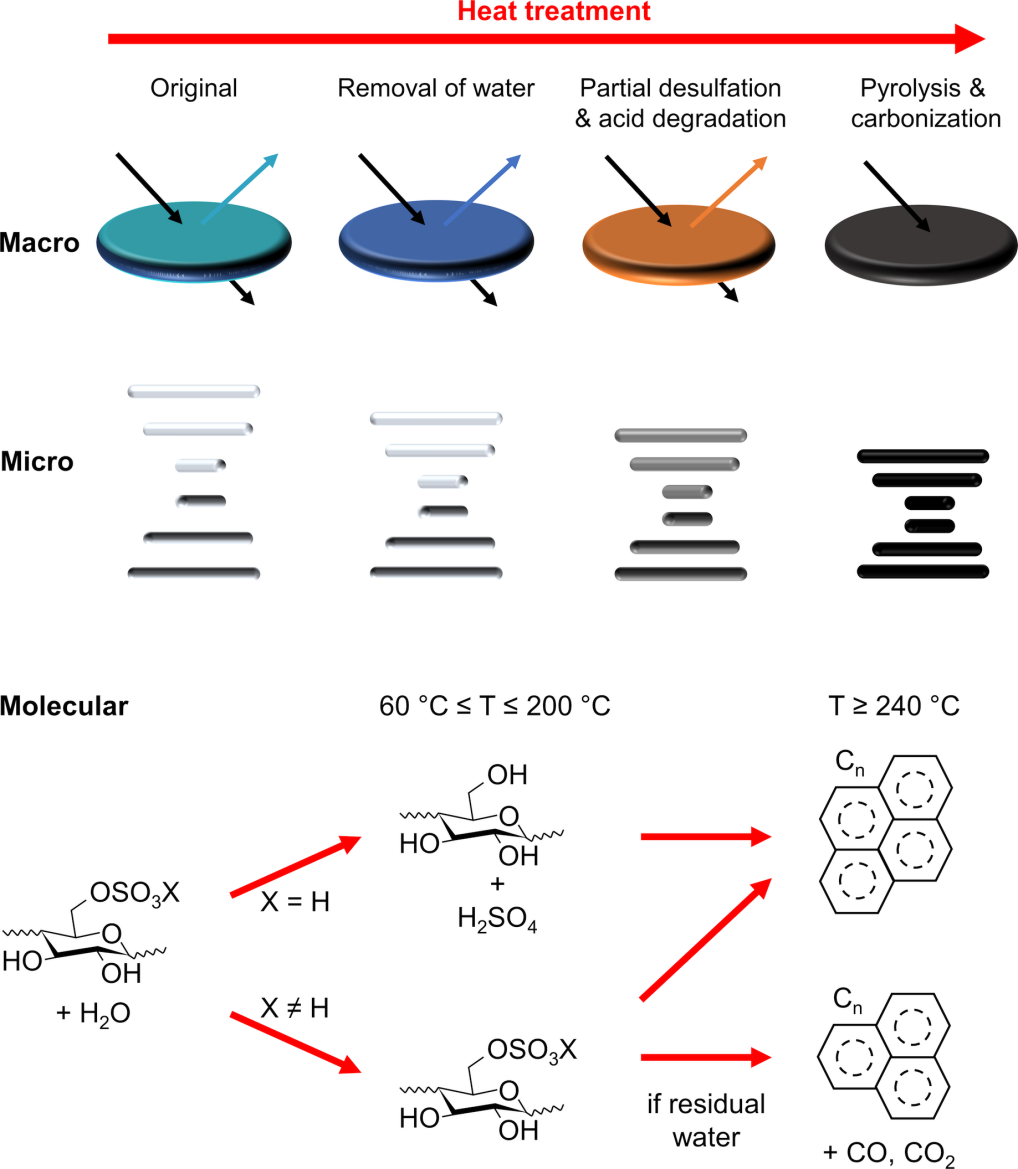
A schematic summarizing the effect of heat-treatment intensity on the macro-, micro-, and molecular structure of photonic CNC films. The color of the photonic CNC film is first blue-shifted due to the pitch contraction upon removal of free water, then the film darkens due to desulfation-induced acid degradation, and finally turns black upon carbonization of the cellulose. Importantly, alkali ion-exchanged CNCs (e.g., Na-CNCs) have an improved thermal stability due to the less labile −OSO_3_^–^ surface groups.

Heat treatment can successively induce multiple changes within a CNC film, from the removal of free water within the structure to desulfation and degradation of the CNCs, and ultimately pyrolysis to form a graphitic film. By controlling the degree of heat treatment, these macro-, micro-, and molecular-scale changes in the photonic CNC film can be targeted for different applications (Figure 88).

In its simplest usage, heat treatment can be used to remove water trapped within the helicoidal CNC film. This residual water can be divided into “bound water”, which is always present and has been linked to aiding their redispersibility,^526,602,620^ and “free water” that is absorbed by the hydrophilic film when stored at nonzero relative humidity. The latter can subtly red-shift the perceived color of the “dry” film under ambient conditions (i.e., *Δλ* ≈ 15 nm for 0 → 50% RH).^338^ While in the planar “film” geometry the amount of residual water is clearly minor (due to the ability for the mesophase to efficiently collapse in the vertical direction),^526^ for the case of radially aligned CNC microparticles the increasing resistance to isotropic compression (combined with distortion of the cholesteric phase, see section 6.2) can lead to significant amounts of retained water (ca. 30 wt %).^309^ Much like cocasting with a nonvolatile additive (section 10.1), this results in incomplete compression of the cholesteric order and thus an apparent red-shift in the photonic response compared to the analogous planar CNC film. This residual water can be removed as a post-treatment, via either heat (≫ 100 °C) or by washing with polar solvent (e.g., methanol), allowing for near-complete compression of the structure to be achieved.

More commonly, heat treatment is used to improve the resistance of CNC films to swelling in polar solvents.^547^ This effect is attributed to desulfation, which liberates sulfuric acid from the CNCs, resulting in the film becoming more hydrophobic (see section 8.2.1 for mechanism). However, it is relevant to note that films made from alkali ion-exchanged CNCs (i.e., Na-CNCs) become nondispersible in water when heated at 105 °C for longer than 16 h without detectable desulfation.^602^ This observation suggests that dehydration-induced irreversible aggregation (cf. hornification^778^) may be critical in controlling the redispersibility of dried CNC films, rather than the increased hydrophobicity from desulfation alone. As a final comment, the redispersibility of CNC films also is reduced when immersed in high-ionic-strength aqueous solutions, due to the weaker colloidal stability of individual CNCs (see section 5.2.3).

Films made from acidic CNCs (H–CNCs) are more sensitive to heat treatment than those prepared from neutralized Na-CNCs.^602^ For example, a few seconds at 200 °C is sufficient to cause significant blackening of H–CNCs films, while Na-CNCs films still display color even after 48 h at 200 °C, albeit with some darkening.^773,774,779^ This can be rationalized by considering that desulfation is an acid-catalyzed process, and as such heat treatment of H–CNCs creates a high sulfuric acid concentration within the dry film that can degrade cellulose, leading to significant darkening at high temperature (e.g., > 180 °C). To avoid this effect, heat-treatment of H–CNC films can be undertaken at lower temperatures (e.g., 100 °C), and at higher relative humidity (where desulfation is slower).^602,625^ Alternatively, darkening can be avoided by heating under vacuum (e.g., 75 °C, 40 mbar, 1 h), which enables the rapid removal of any generated sulfuric acid before it can locally degrade the cellulose.^773^ By this method it was reported that ∼75% of the sulfate half-ester groups can be removed, comparable with heating at ∼100 °C under ambient pressure.^602,773^ Furthermore, prior desulfation at a low temperature was found to improve the subsequent thermal stability of CNCs at a high temperature, with no darkening observed from CNC pigments in a melt-extrusion process with a thermoplastic polymer at 185 °C.^773^ Alternatively, by exploiting the slower release of sulfuric acid from Na-CNC films, a more intense heat treatment can be applied (180 °C for 30 min) such that the resultant CNC film fragments can be stably formulated (1+ years) in various solvents (including water) while retaining coloration.^547^ Finally, a moderate amount of darkening can actually be advantageous,^780^ as it can enhance the visual contrast of the structural color by acting analogously to doping the film with a broadband absorber (see sections 10.1 and 7.4.1).

The pyrolysis of CNCs occurring under anaerobic conditions at very high temperatures has also been exploited for the fabrication of chiral carbonaceous films for use as e.g. electrodes.^779,780^ Under these extreme conditions (900 °C under a flow of inert argon), the constituent cellulose will pyrolyze into conductive, aromatic carbon, while still maintaining the chiral structure of the original helicoidal CNC film (despite the absence of a hard template, such as silica^781^). For this end-use, however, the H–CNC are actually more suitable, since their release of sulfuric acid helps in removing bound water and results in less carbon loss as volatile CO and CO_2._^309,782^

#### 10.2.2 Infiltration

**Figure 89:**
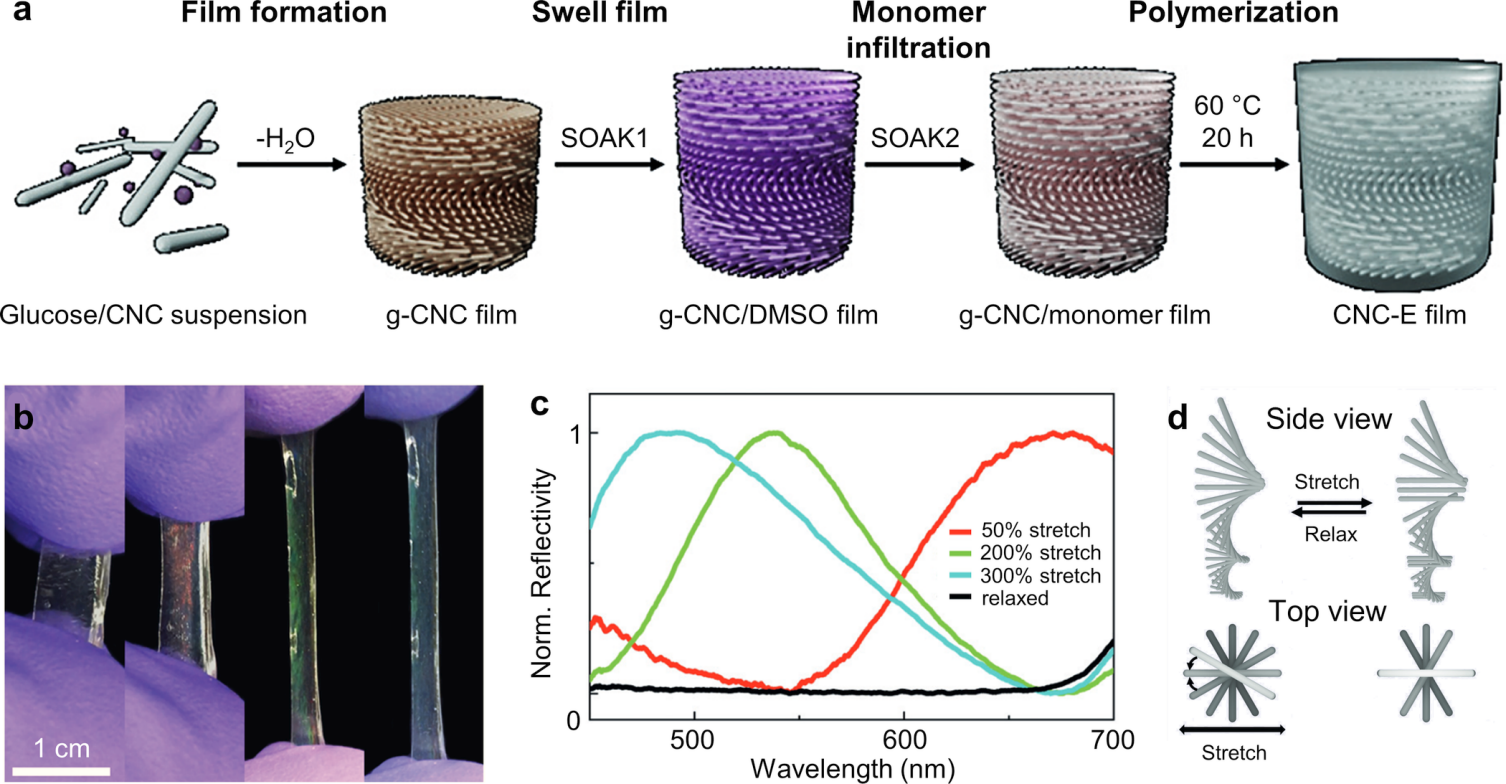
Infiltration as a method to prepare an elastomeric “CNC-E” composite film. (a) Schematic of the fabrication steps to produce the CNC-E film by infiltration. (b) Photographs of the CNC-E film under elongation by 0%, 65%, 250%, and 300% (left to right), showing the visible blue-shift in the color. (c) Normalized reflection spectra of the CNC-E film at a relaxed state (black line, i.e., reflection at infrared wavelengths) and upon stretching up to 300% to give sequentially red, green, and blue colors. (d) Schematic of the proposed model showing the distortion of helicoidal architecture upon stretching perpendicular to the helical axis. Adapted with permission from ref (725). Copyright 2019 John Wiley and Sons.

An alternative route to modifying the properties of a photonic CNC film is to infiltrate it with functional additives. One such example is the formation of CNC-elastomer (CNC-E) composites with strain-induced responsive color.^725,783^ As shown in Figure 89a, a glucose-doped CNC film (g-CNC) underwent a two-step soak process;^725^ first to swell the film with dimethyl sulfoxide (SOAK1) and then to infiltrate with a monomer solution (SOAK2: ethyl acrylate, 2-hydroxyethyl acrylate, AIBN). After polymerization at 60 °C, an elastomer network was formed within the cholesteric CNC film. When stretched, the initially colorless CNC-E composite transitioned through red to green to blue with increasing extension (Figure 89b,c). This significant blue-shift of the reflection peak suggests that the reduction in thickness upon elongation results in a corresponding geometric compression of the pitch. Moreover, while the helicoidal structure is retained upon elongation, it is increasingly distorted as the CNCs become aligned in the stretching direction (Figure 89d). This alignment also imparts an effective linear birefringence in the stretching direction, irrespective of the cholesteric order.^777^ Importantly, stretching perpendicular to the helical axis does not unwind the helicoidal structure: although the periodicity is distorted, the number of pitch repeats is expected to be unchanged.^540,784^ A similar swelling, soaking, and in situ polymerization process with a polymerizable deep eutectic solvent (PDES) can produce an stretchable CNC film with a high ionic conductivity.^783^ This CNC-PDES composite exhibited a similar dynamic optical response to the CNC-E composite, along with good adhesion properties and strain-induced changes in conductivity.

A second example is infiltration with a cationic polyelectrolyte, which cannot be easily incorporated via coassembly as it would destabilize the colloidal CNC suspension (see section 8.3.2).^785^ Infiltration of methacryloxyethyl trimethylammonium chloride (MTAC), followed by UV-polymerization resulted in structurally colored films. Moreover, the degree of swelling in water could be tuned via the hydration capacities of the counterion,^786^ allowing for the possibility of creating spatial patterns or images within the film. Relatedly, photonic CNC films infiltrated with alkali metal ions have been employed as gate dielectrics in amorphous indium–gallium–zinc oxide (a-IGZO) field-effect transistors.^787,788^ By combining the filtering of circular polarized light by the CNC film with the light sensitivity of a-IGZO, such devices have been shown to be capable of discrimination between LCP and RCP light in the photonic band gap, via an increase in photogenerated current.^787^

Finally, impregnation of a CNC film with the molecular liquid crystal, 4-cyano-4′-pentylbiphenyl (5CB), has been exploited to overcome the fundamental reflectivity limit of the helicoidal structure.^789^ In this example, the 5CB was not uniformly distributed throughout the CNC film, but instead localized within voids and cracks. When the 5CB was nematically ordered within a horizontal break in the CNC film (a process that is enhanced by planar surface anchoring to the cellulose) it could act as a (near-half wave) retardation plate allowing for some interconversion of LCP and RCP light. This strategy to enhance reflectivity is introduced in more detail and illustrated with examples in section 10.2.4, while its optical mechanism is described in section 7.2.2.

#### 10.2.3 Replication

**Figure 90:**
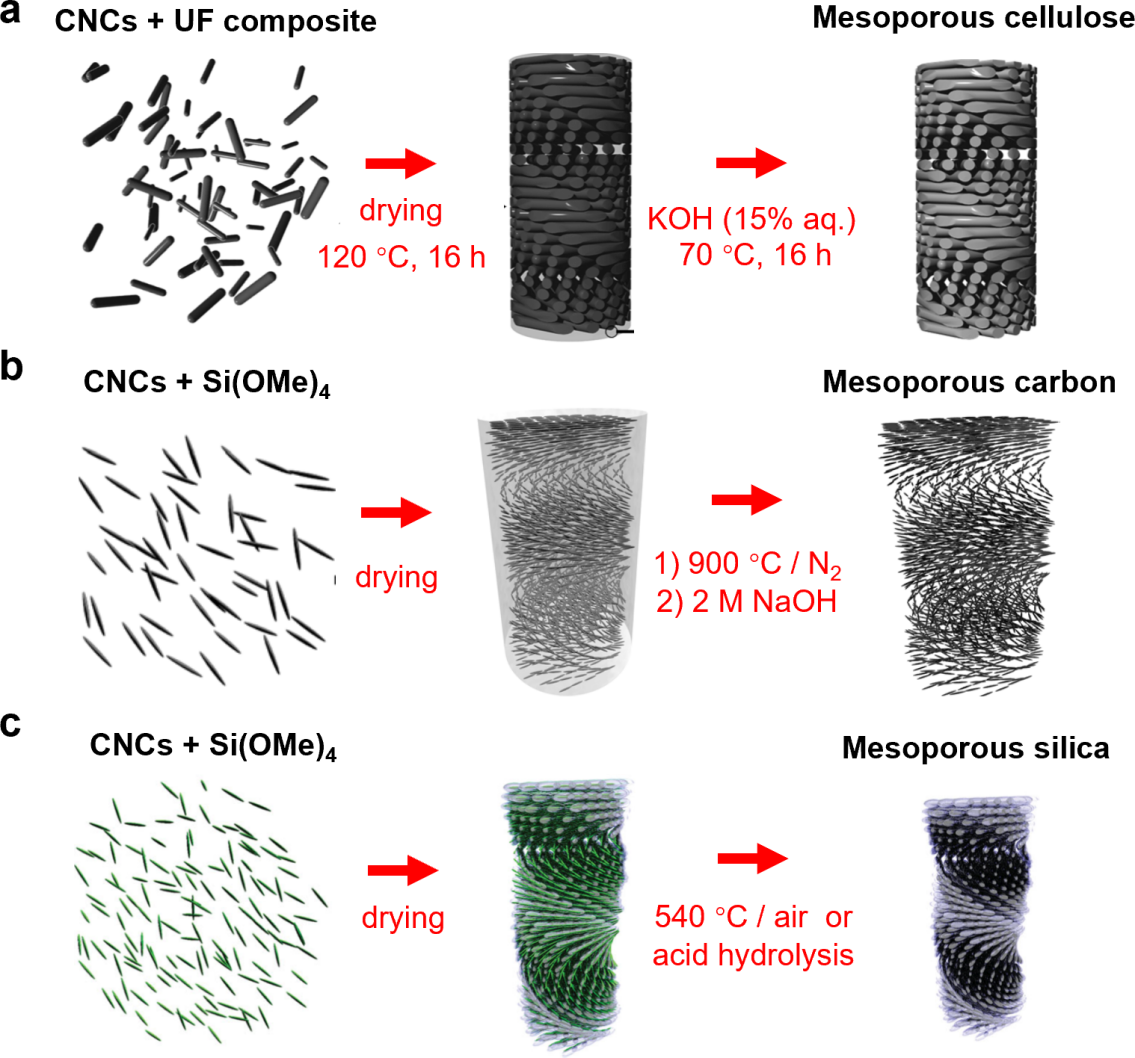
Fabrication of mesoporous helicoidal materials via the selective removal of specific components from a cholesteric CNC composite. (a) CNC and urea formaldehyde (UF) composites were heat treated and then exposed to strong base, leading to mesoporous cellulose films. Adapted with permission from ref (790). Copyright 2014 John Wiley and Sons. (b) CNC and TMOS composites were pyrolyzed at high temperature under nitrogen atmosphere and then exposed to strong base to remove the silica, resulting in mesoporous carbon films. Adapted with permission from ref (781). Copyright 2011 John Wiley and Sons. (c) Alternatively, the same initial composites were exposed to high temperature or strong acid to selectively remove the cellulose, leading to mesoporous silica films. Adapted with permission from ref (800). Copyright 2013 John Wiley and Sons.

Photonic CNC films can be used as a template to impart helicoidal ordering into other materials (Figure 90). For example, by exploiting the aforementioned coassembly approach in section 10.1, mesoporous CNC films can be formed by subsequently removing the additive, e.g. urea formaldehyde (UF) can be removed with an aggressive base treatment without collapse of the CNC film (Figure 90a).^790^ Furthermore, this treatment endows the photonic films with enhanced water stability, which can be attributed to desulfation of the CNCs. Owing to their mesoporosity, such CNC films display (i) a rapid hydrochromic response to water (λ = 330 → 820 nm in <1 min), with the solvent composition found to determine the equilibrium color, and (ii) a reversible mechanochromic response when swollen in water (λ = 630 to 520 nm, upon applying a pressure of 8 MPa). Furthermore, such mesoporous CNC films (templated with a range of additives) can be used as a scaffold to produce e.g. plasmonic gold nanoparticles, which can selectively swell in the presence of thiols.^791,792^ Notably, if the CNCs are carbonized (900 °C, N_2_) prior to removal of the additive (in this case silica), mesoporous carbon scaffolds can be produced with a cholesteric order that may find application as electrodes (Figure 90b).^781^ Lastly, analogous mesoporous CNC films can be prepared by postswelling with an aqueous solution of *N*-methylmorpholine-*N*-oxide (NMMO, a solvent for cellulose that here is used to cross-link the CNCs),^793^ followed by solvent exchange to water and freeze-drying of the CNC hydrogel.^794^ Again, such mesoporous CNC films swell rapidly in the presence of polar solvents giving rise to a spectrum of colors (tunable through surface modification), which was exploited to produce responsive pigments and patterned films.

Relatedly, ice templating of CNC suspensions has been used to prepare so-called “photonic aerogels”.^795^ By heating a fully cholesteric suspension at 95 °C for 48 h, in situ desulfation resulted in gelation of the CNCs, which enabled the structure to be maintained upon flash freezing in liquid nitrogen followed by sublimation of water (i.e., freeze-drying).^796^ The resultant aerogel appears white due to strong broadband scattering from the porous architecture. However, visible color can be observed upon index matching with water, or mechanical compression of the pores. Note that the latter case is irreversible and as such the resulting structurally colored film can no longer be considered an aerogel. Interestingly, the presence of visible blue color suggests that the pitch in the aerogel is much smaller than expected for a CNC hydrogel, suggesting that ice crystallization between the cholesteric domains caused a local compression of the pitch during the dehydration process. Finally, by infiltrating the aerogel with PDMS elastomer, the structural color can be reversibly revealed upon applying pressure, again due to suppression of broadband scattering.

Alternatively, self-assembled CNCs can be used as a sacrificial cholesteric template to produce chiral mesoporous structures from other materials. For example, taking inspiration from the processing routes commonly used in ceramics,^797^ the coassembly of CNCs with silica or organosilica sol–gel precursors, followed by the removal of the cellulose (by e.g. pyrolysis, strong acid or base), results in the formation of a mesoporous glass that inherits chiroptical properties from the templating helicoidal film (Figure 90c).^437,728,798−801^ Notably, such mesoporous glasses can be further exploited as a template for e.g. chiral mesoporous titania,^802^ or as a scaffold for photoactive dyes (e.g., spiropyran^803^) or liquid crystals.^804,805^ Instead, if the CNC suspension is cocast with poly(acrylic acid) and the resultant film subsequently infiltrated with amorphous calcium carbonate (which can be mineralized into calcite), then a photonic film can be produced with significantly enhanced mechanical properties compared to the organic template.^806^ Alternatively, base treatment of a CNC/phenol-formaldehyde composite film produces a pliable mesoporous resin film.^807^ By spatially functionalizing such a resin film, the colorimetric response to solvents can be tuned allowing for multicomponent sensing or anticounterfeiting labels,^808^ or for in situ growth of plasmonic metal nanoparticles.^809^ This approach was later expanded to produce actuators by casting a bilayer resin film, with each layer having a different composition and thus a different swelling behavior in various solvents.^810^ A similar cocasting strategy enabled the production of mesoporous latex photonic films with high flexibility.^811^ Finally, by cocasting with two or more additives it is possible to produce films with asymmetric behavior, arising from vertical gradients in the pitch. For example, graphene oxide (GO) has been shown to partition within a CNC/latex composite which, after removal of the CNCs, yields chiral mesoporous films with different wettability on either film face, resulting in actuation upon differential swelling with water.^812^ This approach was expanded to CNC/resin/GO composites, where increased cross-linking (e.g., with formaldehyde) enabled the films to be shaped into predefined geometries, resulting in actuators with shape memory behavior (e.g., spring, tweezers).^813^

#### 10.2.4 Lamination

Lamination is a process whereby several layers of different composition or functionality are combined to enhance the overall properties. It has been employed in various ways to expand the versatility of photonic CNC films. In this section, three examples are given, focusing on enhancing the optical response, mechanical behavior, and water resistance.

**Figure 91:**
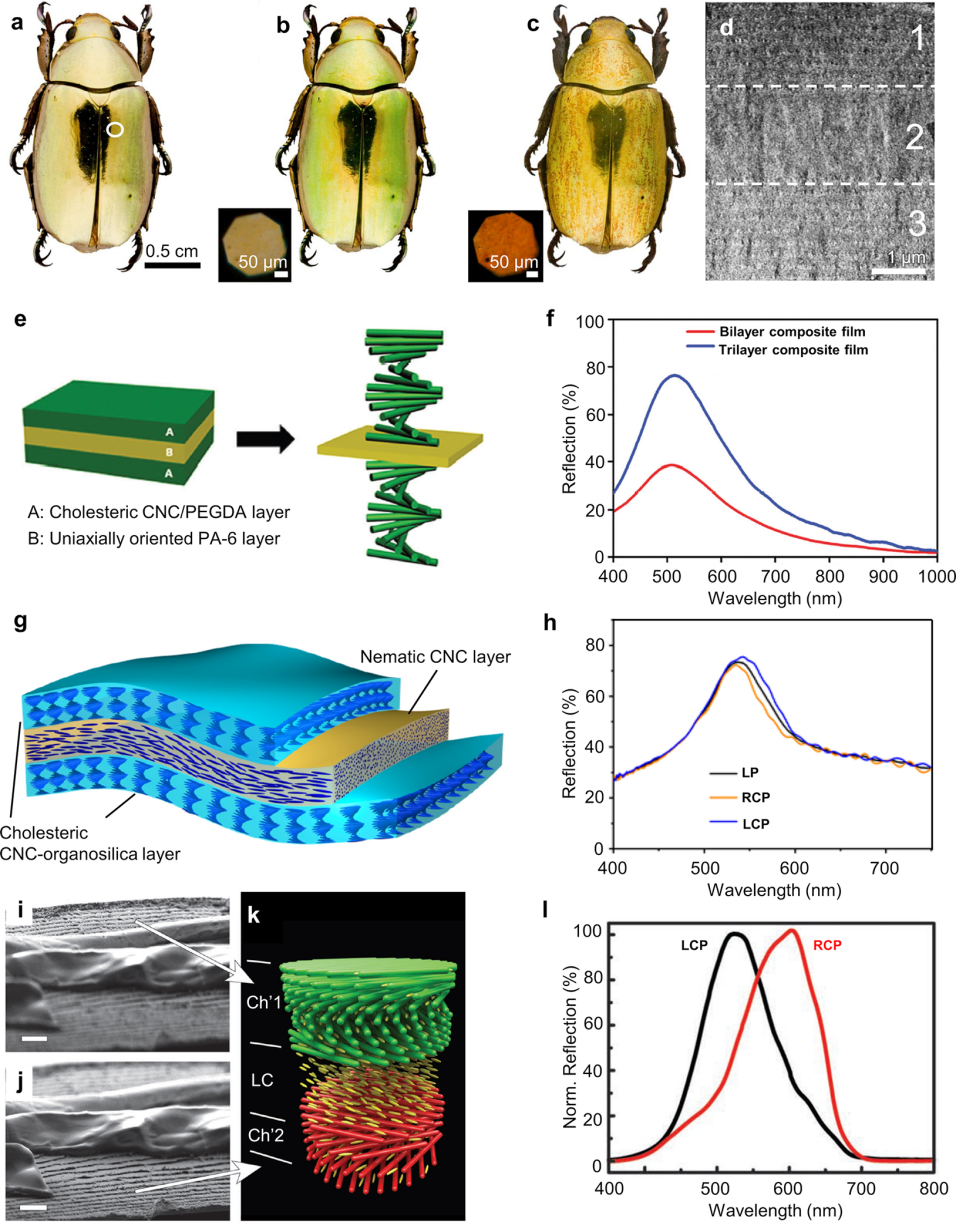
Three strategies to mimic the trilayer structure of the cuticle of the golden scarab beetle with CNC laminates. (a–c) Photographs of *Chrysina resplendens* viewed under (a) unpolarized light, (b) a left circular polarizer, and (c) a right circular polarizer. (d) Cross-sectional TEM image of the cuticle of this beetle showing an aligned layer (2) sandwiched by two helicoidal layers (1 and 3). Reproduced with permission from ref (815). Copyright 2019 Elsevier. (e) Schematic showing an artificial trilayer structure in which two cholesteric CNC/PEGDA layers are separated by a uniaxially oriented PA-6 layer that acts as a half-wave plate. (f) Reflection spectra of bilayer and trilayer composite films, showing the enhanced reflectivity of unpolarized light. Adapted with permission from ref (816). Copyright 2016 Royal Society of Chemistry. (g) Schematic of a similar trilayer structure, in which two cholesteric CNC-organosilica layers are separated by a nematic-like CNC layer.^818^ (h) Reflection spectra of this trilayer composite film under different polarizers. LP refers to linear polarization. Adapted with permission from ref (818). Copyright 2018 American Chemical Society. (i–l) Schematic (k) and SEM images (i-j) of a CNC film infiltrated with the nematic molecular liquid crystal, 5CB. The corresponding reflection spectra recorded through a LCP or RCP filter are reported in (l). Adapted with permission from ref (789). Copyright 2016 John Wiley and Sons.

The cuticle of the golden scarab beetle *Chrysina resplendens* strongly reflects both LCP and RCP light, despite containing no right-handed helicoidal structure (Figure 91a–c). This is due to the presence of a birefringent layer between two left-handed helicoidal domains (Figure 91d), which acts as a half-wave plate that interconverts LCP and RCP light upon transmission (see section 7.2.2). This strategy can be used to overcome the theoretical limit of a maximum of 50% reflection of unpolarized light from a left-handed helicoidal structure.^569,814,815^ To replicate this trilayer cholesteric-nematic-cholesteric structure, a uniaxially oriented Nylon (polyamide-6, PA-6) film was successively coated on each face with layers of CNC/polyethylene glycol diacrylate (CNC/PEGDA), resulting in up to ≈80% reflection of unpolarized light (Figure 91e,f).^816^ Alternatively, a birefringent layer can be produced by depositing a concentrated CNC suspension under high shear to create a nematic-like layer.^817^ Deposition of such an aligned CNC layer between two helicoidal CNC–organosilica layers resulted in a trilayer laminate with strong, near-identical reflection of both LCP and RCP light (Figure 91g,h).^818^ Finally, a similar result has been achieved by impregnating a CNC film with the nematic molecular liquid crystal 5CB, as introduced in section 10.2.2 (Figure 91i–l). Moreover, in this case the proportion of reflected RCP light could be dynamically tuned by varying the alignment (and thus the birefringence) of the 5CB layer using either temperature variation or an applied AC electric field.^789^ Similar optical effects can also be observed by simply placing a nematic liquid crystal droplet atop a photonic CNC film.^819^

Lamination has also been used to enhance the mechanical behavior of cholesteric CNC films. Pure CNC films are typically brittle due to the small overlap lengths between individual CNCs, the lack of energy-dissipating binder, and the heterogeneities of the polydomain ordering. While coassembly with a variety of polymers or nanoparticles can be used to improve the plasticity of the film (see section 10.1), such additives can perturb the CNC self-assembly process, leading to weaker color. Instead, by alternate drop-casting of cholesteric CNC layers with layers of disordered CNFs (up to 7 layers combined), a robust laminate film can be produced while maintaining vibrant structural color.^760^ The presence of the CNF layers improves the load transfer capability leading to a many-fold enhancement in both tensile strength and toughness compared with the pristine CNC films (Figure 87). Alternatively, by laminating the CNC film between layers of a polydiolcitrate elastomer, a photonic composite with thermally triggered shape memory behavior can be produced.^820^

Lastly, laminating a colored CNC film with a hydrophobic layer has been shown to improve water stability.^821^ Inspired by cutin, a polyhydroxylated polyester of condensed fatty acids found in plant cuticles, aleuritic acid was spray-coated onto a CNC film and then polymerized by hot-pressing (200 °C, 160 bar). Compared to a bare CNC film, the CNC-polyaleuritate film exhibited improved mechanical robustness combined with reduced water uptake and water vapor transmission rates, despite being coated only on a single side.

#### 10.2.5 Fragmentation

**Figure 92:**
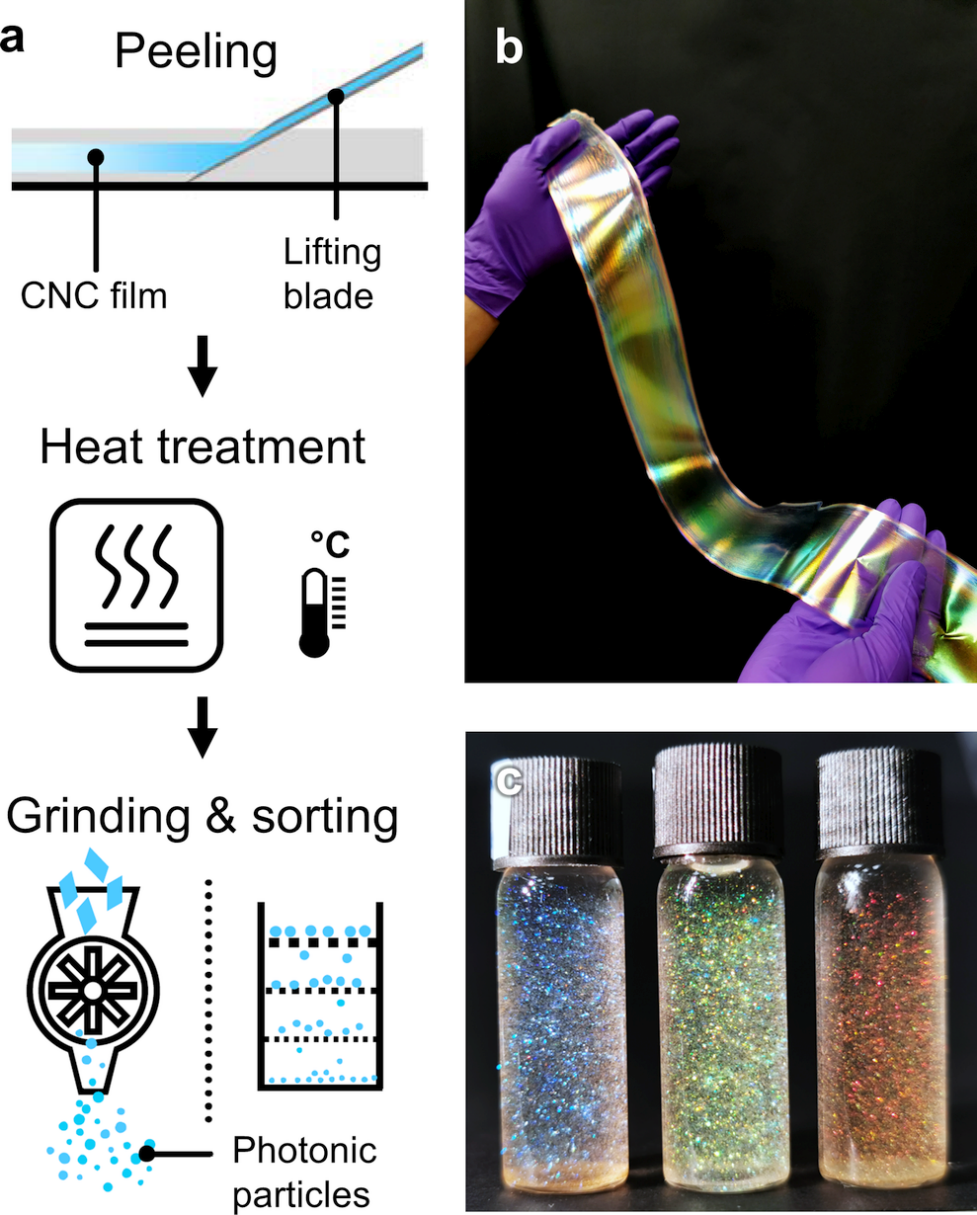
Scalable fabrication of CNC photonic microflakes. (a) Schematic of the process of converting a roll-to-roll-cast CNC film into microparticles. (b) Photograph of a free-standing roll-to-roll-cast CNC film. (c) Blue CNC microflakes dispersed in pure ethanol, 50 vol% ethanol in water, and pure water, from left to right. Adapted with permission from ref (547). Copyright 2021 The Authors.

CNC-based colorants offer a biosourced and biodegradable alternative to existing effect pigments, which are often made from synthetic polymers (e.g., PET), metals (e.g., aluminum) or inorganic minerals (e.g., mica). CNC effect pigments or glitters can be achieved by mechanically grinding photonic films into flake-like microparticles (“microflakes”).^547,773^ As an example of a scalable manufacturing process (Figure 92), CNC films produced via a roll-to-roll deposition process can be delaminated from the substrate, heat treated in an oven (180 °C for 30 min), and ground into microparticles (using a rotary blade).^547^ Notably, heat treatment was critical to dehydrate the CNC film, which increases its brittleness to enable efficient grinding into faceted microparticles. Furthermore, thermally induced partial desulfation combined with the release of tightly bound water improved the resistance of the resultant CNC microflakes to swelling in solvent, as discussed previously in section 10.2.1. This made the CNC glitter colorfast when formulated in water, with microflakes of diameter above 25 μm displaying visible color, even when stored for over a year. Interestingly, the macroscopic appearance of a coating containing CNC glitter was much less iridescent than for the native CNC film, arising from the variation in local microflake alignment and Snell refraction at the coating interface. Finally, besides grinding, ultrasonication has also been employed to produce CNC microflakes.^822^ By sonicating fragments of a brittle CNC film dispersed in tetrahydrofuran and controlling the duration of ultrasonication, microflakes of a specific size range can be produced. However, the efficacy of disintegration plateaus at longer treatment times leading to a minimum flake size for a given power input (e.g., diameters down to 77 ± 30 μm were reported for a 60 min treatment using an ultrasonic cell disruptor rated at 650 W).

### 10.3 Functional Substrates for Photonic CNC Films

The choice of substrate can play an important role in the self-assembly of a CNC film, as it can influence the surface wetting of the suspension and the anchoring of the individual CNCs (see section 9.2.2). Moreover, the substrate can also enhance the optical functionality of the final film (see section 7.4.1). This can range from the strikingly different visual appearance upon casting or laminating a photonic CNC film onto a substrate that is either a broadband scatterer (i.e., white) or absorber (i.e., black), through to patterning the surface with optical motifs or coating complex three-dimensional surfaces.

#### 10.3.1 Manipulating the Visual Appearance

**Figure 93:**
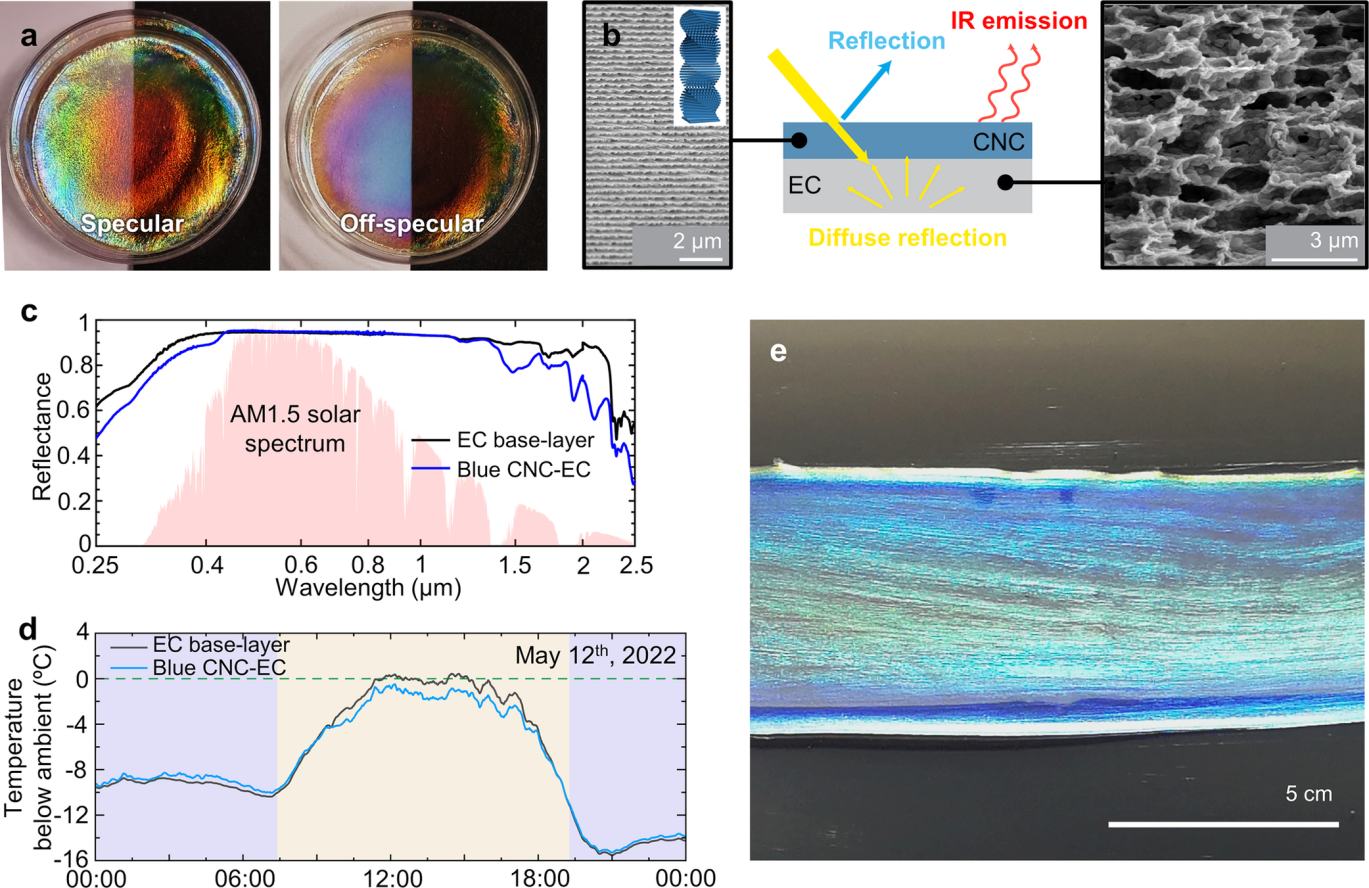
Influence of the substrate on the visual appearance of photonic CNC films. (a) Photograph of a red CNC film in a Petri dish above a black and white substrate, with specular and off-specular illumination. (b) Schematic of light transport in a bilayer film in which a white ethyl cellulose (EC) substrate is beneath a photonic CNC layer. (c) Hemispheric reflectance of an EC film and a blue CNC–EC bilayer film, demonstrating the broadband solar reflection. (d) Temperature below ambient for both the white EC film and blue CNC–EC bilayer film in a field test. (e) Photograph of a large-scale CNC-EC bilayer film showing a vivid blue color at specular angles, when viewed under natural light. (b–e) Adapted with permission from ref (566) under Creative Commons CC-BY. Copyright 2022 The Authors.

Structural coloration arises from the wavelength-selective reflection, transmission or scattering of visible light. In particular, a well-ordered photonic CNC film will reflect a narrow wavelength band (due to the nanostructure), while all other wavelengths are transmitted (due to the near-zero absorption of cellulose in the visible regime). As such the overall visual appearance is highly dependent on how this transmitted light is dissipated (for optical discussion see sections 7.3.1 and 7.4.1). Consequently, the presence of a substrate and its optical properties is a crucial design consideration when looking to exploit photonic CNC materials in real-world applications. The most common solution for photonic CNC films (or indeed most structurally colored materials) is to combine them with a light-absorbing substrate (Figure 93a). In particular, a black substrate will absorb any light that is transmitted through the film, ensuring that only the light reflected by the nanostructure is observed by the observer. This offers the advantage of removing unwanted light without attenuating the photonic response, in contrast to the incorporation of broadband absorbers into the film itself (see section 10.1).

Alternatively, the use of a broadband reflector (i.e., a mirror) or a white scatterer as the substrate can reveal interesting optical effects. An example of where a white substrate is, in fact, desirable is for daytime radiative cooling (DRC), whereby a surface can be passively cooled below ambient temperature by combining high mid-IR emission with low absorption at solar wavelengths.^823,824^ To achieve DRC, materials are designed to maximize reflection across the entire solar spectrum to achieve high cooling performance, which promotes a white or mirror-like appearance. This monotonous appearance limits their appeal in architecture and consumer-oriented industries where visual aesthetics is a key concern. While conventional dyes and pigments can be added into DRC materials to provide coloration, the unavoidable increase in solar absorption is detrimental to the desired cooling effect.^825^ Recently, it has been shown that a helicoidal CNC film can be used to impart coloration without increasing absorption. This has been used to reduce the thermal heating of an absorbing substrate (e.g., silicon^826^) or, when laminated with a white scattering substrate of ethyl cellulose (EC), to achieve subambient DRC from a colored, opaque film.^566^ In the latter example, the films showed a strong structurally colored reflection at specular angles from the photonic CNC layer, while at other angles its complementary color was prevalent (see section 7.4.4 and Figure 45), due to the efficient backscattering of the transmitted light by the porous EC substrate (Figure 93b). Given the optical appearance is derived solely from the nanostructures of the two layers, the colored film retains the advantages of cellulose, namely negligible absorption at solar wavelengths (<5%) and very high mid-IR emission (>90%), which allowed for subambient cooling (Figure 93c,d). Importantly, the simple and robust fabrication process for this bilayer film allows for scale-up by established methods, such as roll-to-roll deposition (Figure 93e, for roll-to-roll see section 9.2.1).

#### 10.3.2 Casting on Structured Surfaces

The visual appearance of a photonic CNC film can be manipulated by employing a structured substrate. For example, casting on top of a relief pattern can be used to emboss the resultant photonic film on the macroscale,^678,827^ while soft lithography can be used to transfer finer features to the CNC film surface, allowing for the inclusion of optical motifs (e.g., periodic surface elements for diffraction gratings or nanoparticle arrays for microlenses).^551,552,828,829^ In the latter systems, the left-handed helicoidal CNC film typically acts as a filter for the optical response from these additional structural features.

Films have also been cast onto a flexible meshed substrate, such as Nylon, with the CNC layer formed atop and around the embedded substrate in an interlocked, “multilayered” configuration.^830,831^ When a small droplet is cast onto a free-standing mesh, a coffee ring stain is formed that is composed of many self-supported, suspended, thin films within the cells of the mesh.^830^ In this geometry, the confined suspension was sheared by capillary forces prior to the onset of kinetic arrest. Furthermore, due to differences in compression of the CNC mesophase upon drying around the filament, compared with that suspended between filaments, a range of pitch values and thus colors are generated. During the drying of much larger films, macroscopic deformation due to capillary forces was inhibited by employing a plasticizer (<60 wt % PEG) in combination with a hydrophilic planar support (contact angle <90°).^831^ The incorporation of PEG also reduced the degree of compression upon drying and thus led to a more uniform visual appearance. Importantly, such mesh-cast CNC films were found to fracture less than analogous films cast on a flat surface, with the tortuous fracture propagation found to follow the film topography.

A CNC suspension can also be dried within a macroscopic hemispherical template, which results in shell-like films (Ø ≈ 4 mm) with a metallic, broadband reflection.^553^ The films took the form of a hollow “cap” that had a relatively uniform thickness and a domain structure aligned to the curved interface, which can be attributed to strong planar anchoring with the curved substrate.

### 10.4 Chiroptical Effects from Composite CNC Films

Optically functional additives, such as plasmonic nanoparticles (NPs) or luminescent dopants, can exploit the architecture of a helicoidally ordered CNC film to produce chiroptical effects that are independent or complementary to the photonic response from the CNCs themselves. This section will summarize the progress made to date on this related topic.

#### 10.4.1 Plasmonics

Plasmonic chiroptical activity arises from the cross-coupling of electric and magnetic fields within a medium. This can occur by dipole–dipole interactions between achiral plasmonic NPs positioned in a geometric configuration that breaks mirror symmetry. Consequently, chiral media containing discrete plasmonic NPs usually exhibit circular dichroism (CD) in the extinction band of the individual NPs, analogous to the Cotton effect produced by exciton coupling in chiral molecules.^832^ As such, chiroptical plasmonic materials with strong natural optical activity have potential as negative refraction materials,^833^ circular polarizers,^834^ sensors for biomolecules,^835^ and detectors for circularly polarized light.^836^

In this regard, it could be expected that the chiral architecture of a helicoidal CNC film can be used to impart chirality to a spatial distribution of plasmonic NPs,^837^ thereby producing a strong CD signal. However, while several studies have produced composite CNC films containing plasmonic NPs,^556,792,837−839^ it has been challenging to deconvolute the CD signal into contributions from the helicoidal matrix, any intrinsic CD signal arising from NP interactions, and the properties of the spectropolarimeter used. The primary issue arises from the LCP-selective reflection at the photonic bandgap of the CNC film (discussed in section 7.3.1). This corresponds to a strong, positive peak in the measured CD spectrum (see sections 7.3.3 and 7.4.3), which can mask or interfere with any signal arising from the plasmonic dopants. Furthermore, an apparent CD signal can arise from the combination of linear dichroism and birefringence in the solid film, which in the case of birefringent CNCs could arise from the varying alignment of multiple helicoidal domains. Finally, artifactual CD signals can arise from the imperfect optics (mainly residual static polarization from the photoelastic modulator) and electronics (from the photomultiplier and lock-in amplifier) of commercial CD spectrometers.^840^ These effects can be deconvoluted by Mueller matrix ellipsometry or by comparing CD spectra for different sample configurations.^841−843^

**Figure 94:**
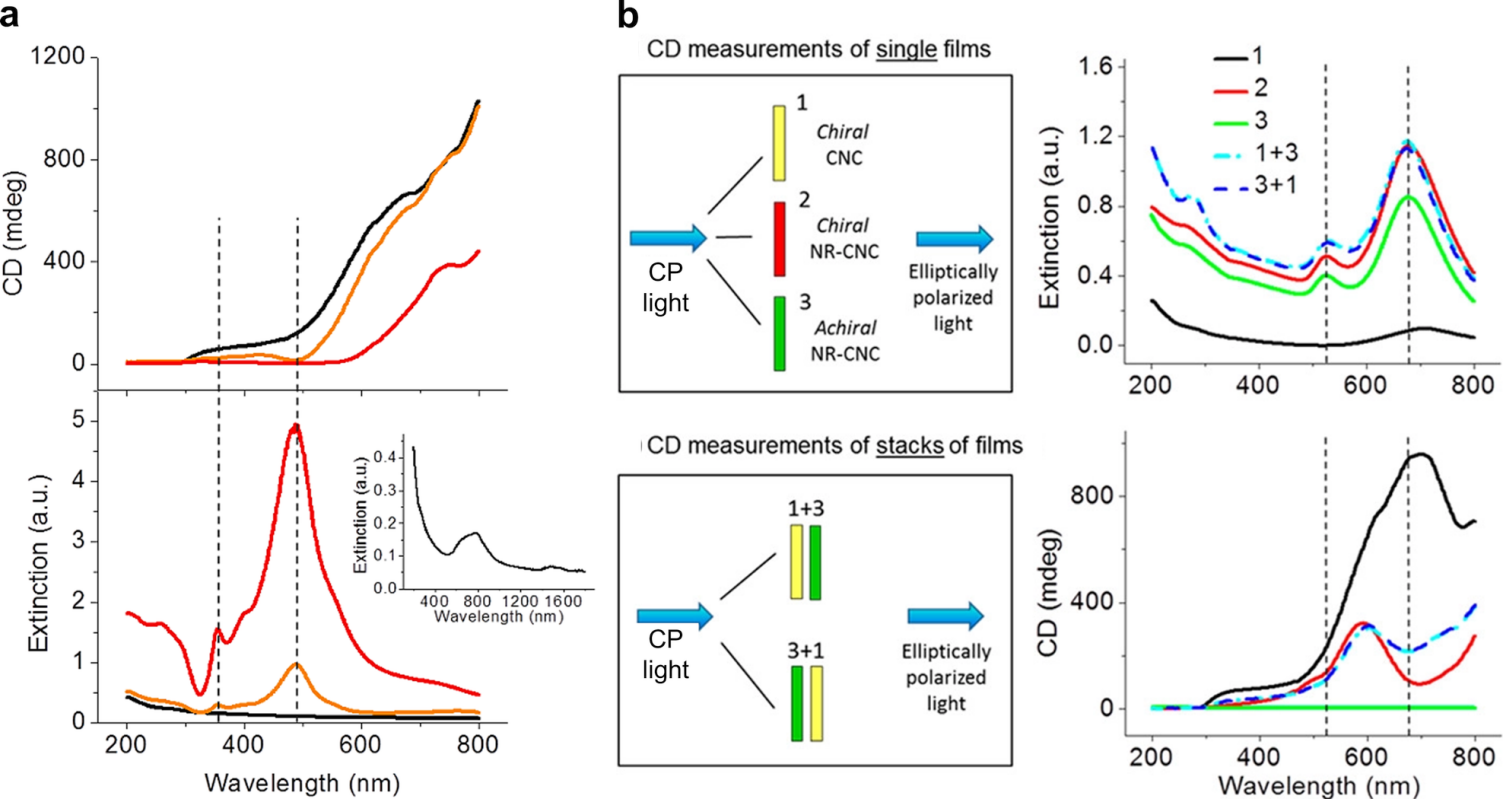
Spectral analysis of gold nanoparticle (AuNP) and gold nanorod (AuNR) composite CNC films with varying spectral overlap between the photonic bandgap of the helicoidal CNC structure and the AuNP extinction peak. (a) Example of when there is negligible spectral overlap: CD and extinction spectra of a pure CNC film (black line) and AuNP-CNC composite films with increasing loading of AuNPs (orange: 2.8 and red: 28.4 pmol_AuNP_/g_CNC_). The dashed vertical lines indicate the most prominent plasmonic extinction bands of AuNPs. The inset in the extinction spectrum for the pure CNC film, showing the spectral separation of the CNC extinction peak from that of the AuNPs. No CD signal from the AuNPs is observed in this case. (b) Example of when there is significant spectral overlap. By comparing CD and extinction spectra for single films and stacked films the optical response can be understood. The apparent dip in the CD spectra appears at the primary plasmonic extinction band. However, this dip is primarily due to the strong absorption of the AuNPs, as the stacking of an achiral AuNP-CNC film and a chiral CNC film produces the same apparent CD spectra as the chiral AuNP-CNC composite film. Reproduced with permission from ref (556). Copyright 2015 American Chemical Society.

To investigate whether a helicoidal CNC template can impart chirality onto a plasmonic dopant, several studies have coassembled CNCs with gold nanoparticles (AuNPs).^556,838,839^ These systems can be divided into two classes, depending on whether the spectral responses of the photonic CNC template and the plasmonic AuNPs are distinct or overlapping. In the former case, the absence of apparent CD signals at the plasmonic extinction bands suggests that there is no chiral templating effect (Figure 94a).^556^ However, the apparent CD spectrum is more complex when there is significant spectral overlap. The observation of a dip in CD signal in the AuNP extinction band initially led to the conclusion that there was an induced plasmonic chiroptical effect.^574,838^ However, this feature is fundamentally different to the negative CD band expected for optically active materials.^212,844^ Indeed, a subsequent article by the same authors overturned their previous conclusion,^556^ proposing instead that the two apparent peaks in the CD spectra were more rationally explained by a local reduction of the CD signal from the CNC matrix due to strong absorption from the AuNPs (Figure 94b).^556^ As a final comment, it has been reported that the CD signal from the photonic CNC film can be enhanced by plasmonic inclusions, which was attributed to the increased permittivity contrast between the CNC-AuNP structure and the air background.^839^

**Figure 95:**
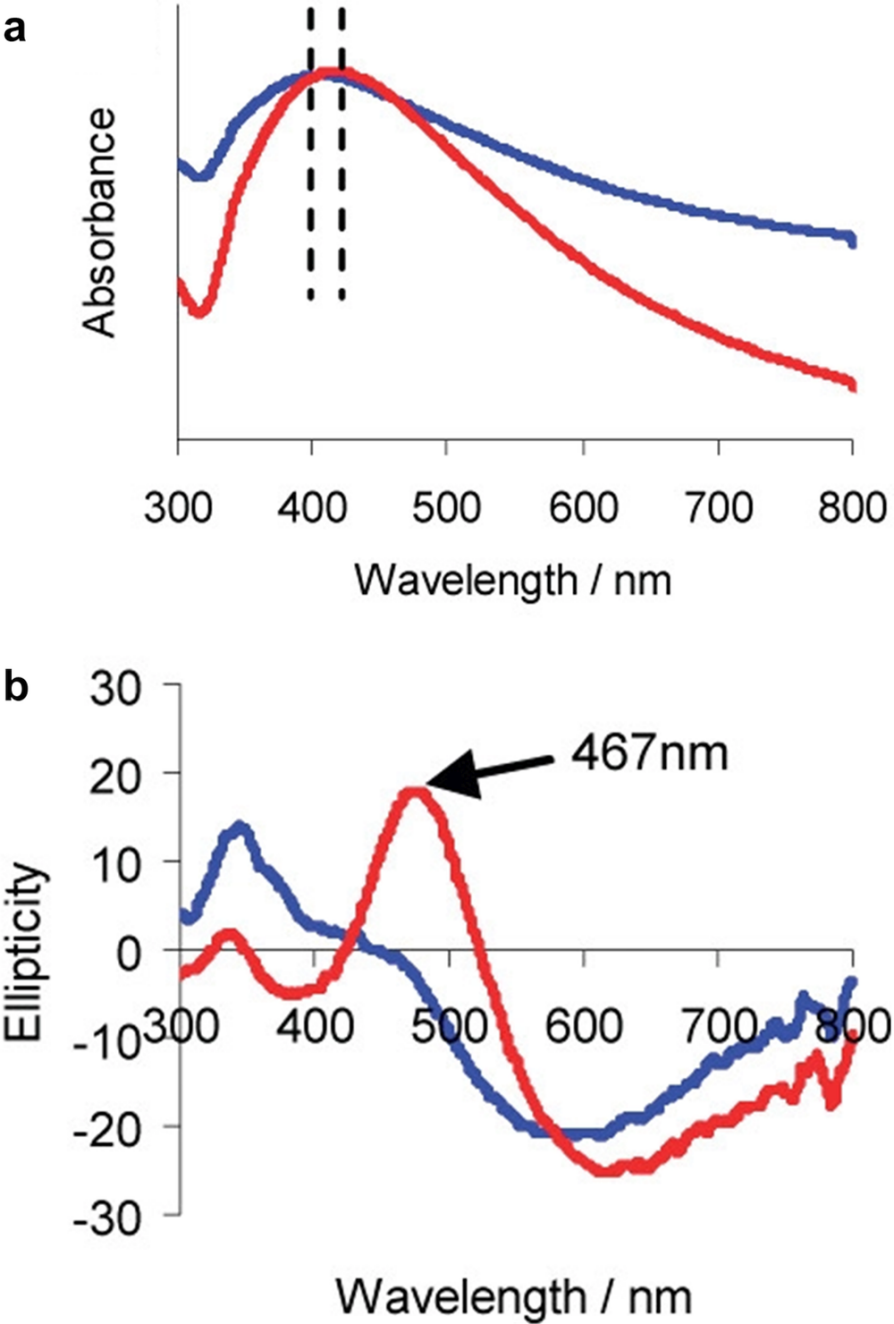
Chiroptical properties of an AgNP-loaded chiral mesoporous silica film before (blue) and after (red) soaking with water. The redshift in both the absorption peak in (a) and the CD spectra in (b) can be attributed to an increase in the background refractive index upon displacement of air with water within the structure. Reproduced with permission from ref (837). Copyright 2011 American Chemical Society.

Lastly, silver nanoparticles (AgNPs) were grown in situ within a chiral mesoporous silica matrix, which was obtained by the sacrificial templating of a composite silica-CNC helicoidal film (see section 10.2.3).^837^ The photonic response of these silica films were chosen to be spectrally distinct from the extinction band of the AgNPs (>1000 nm and ∼400 nm, respectively). Both positive and negative bands were observed in the CD spectra of such composites, whereas they were not observed in equivalent measurements on an achiral silica matrix. Furthermore, infiltration with water, which increases the refractive index of the medium, resulted in a red shift of the extinction and CD peaks (Figure 95). While these observations may suggest that the AgNP film displayed plasmonic chiroptical properties, this conclusion requires further verification. For example, (i) the observed CD signals are small and of similar intensity to the silica template, making precise baselining challenging, (ii) the spatial distribution of the AgNPs is unknown, and thus requires detailed structural analysis, and (iii) additional control experiments are required, e.g., taking CD spectra of the same sample after rotating it about several complementary axes to identify possible artifacts from linear anisotropies.^841−843^

#### 10.4.2 Luminescence

The coassembly of achiral luminescent dopants and CNCs has been proposed as a straightforward and cost-effective way to prepare composite films with circularly polarized luminescence (CPL), as an alternative to the use of chiral luminophores.^845^ CNC-emitter films can be considered as a “host-guest” binary system in which the luminescent guests are incorporated into the left-handed helicoidal CNC nanostructure. Various achiral emitters have been employed as luminescent guests, including rare-earth doped nanoparticles,^846^ organic luminophores,^847−849^ carbon dots,^850,851^ quantum dots,^852^ quantum rods,^853^ BSA-stabilized gold nanoclusters,^854^ and upconverted nanoparticles.^855,856^

The purity of CPL emission can be quantified using by the luminescence dissymmetry factor (*g*_lum_), which is expressed in terms of the LCP and RCP emission intensities (*I*_*L*_ and *I*_*R*_, respectively) according to the following equation:^857^
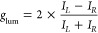
139

To date, values for *g*_lum_ from CNC-emitter films are primarily negative, indicating greater emission of right-circularly polarized luminescence (R-CPL), with the strongest dissymmetry observed when the photonic stop band overlaps with the emission band of the luminophore. These observations are consistent with the polarization-sensitive reflection of the photonic CNC film and the experimental configuration typically used for CPL measurements. When the emission band of the luminophore overlaps with the stop band of the CNC helicoidal structure, LCP light is partially reflected back into the film, while RCP light is directly transmitted. As CPL emission is typically measured in a 180° excitation–emission geometry (i.e., forward-scattering mode), the detected emission therefore has a greater RCP component. In contrast, if the emission band lies outside the stop band, no CPL is expected. While a positive dissymmetry factor (indicating greater LCP emission) has been reported for an emitter layer placed in front of a CNC-only photonic film,^847^ this observation can be attributed to LCP emission in the reverse direction that is then selectively reflected from the CNC film into the forward direction. A positive *g*_lum_ value is also expected for CNC-emitter films measured in other experimental configurations.

**Figure 96:**
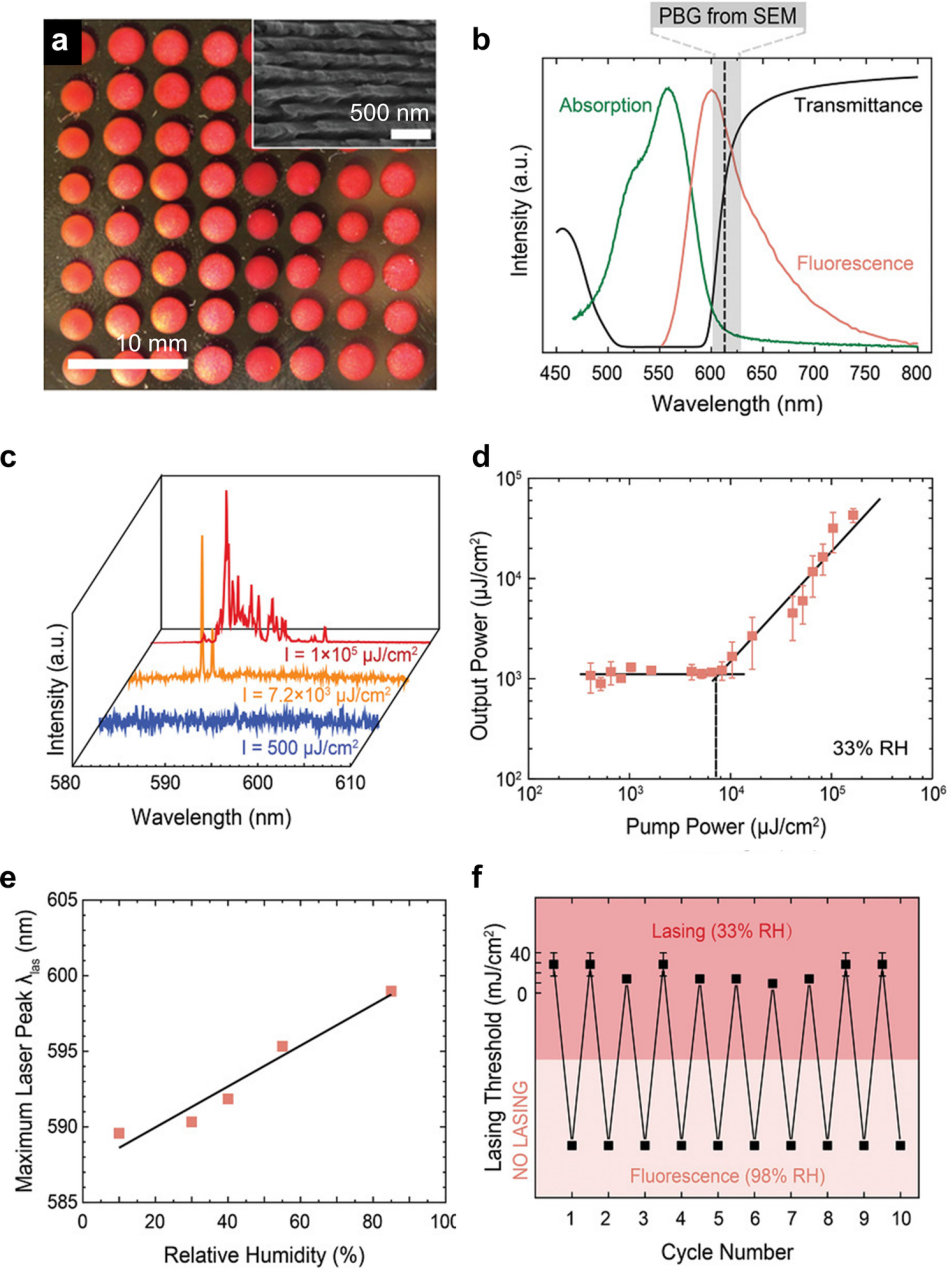
(a) Photograph of a CNC-based laser array. Inset: SEM cross-section of helicoidal structure. (b) Spectra for CNC-polymer composite: transmittance (black), dye absorption (green) and dye fluorescence (red). Also shown is the stop band based on SEM cross-sections (labeled photonic band gap, PBG, in gray shading). (c) Emission spectra for increasing fluence of the excitation pulse, showing the transition from fluorescence to lasing. (d) “Output power” (emission fluence) versus “pump power” (excitation fluence) at 33% relative humidity, showing the onset of lasing. (e) Peak wavelength of laser emission versus relative humidity. (f) Switching from lasing to fluorescence by cycling the humidity. Adapted with permission from ref (858) under Creative Commons CC BY-NC-ND 4.0. Copyright 2020 The Authors.

CNC films incorporating luminescent emitters can also act as lasers,^858,859^ analogous to those demonstrated for molecular cholesteric liquid crystals.^860,861^ The luminophore act as the gain medium, while a well-ordered CNC helicoidal structure acts as a chiral resonant cavity, resulting in LCP laser emission. This principle was demonstrated for CNCs coassembled with a fluorescently doped water-soluble copolymer, enabling the printing of microfilm laser arrays (Figure 96a).^858^ The optimal conditions for lasing are achieved when the short-wavelength edge of the cholesteric stop band lies at the emission wavelength of the luminophore (Figure 96b), as the highest photon density of states is expected at this wavelength.^862^ The transition from fluorescence to lasing is observed by the emergence of a narrow emission spectrum at the stop band edge when the fluence (radiant exposure) of the excitation pulse exceeds a threshold value (Figure 96c,d). The incorporation of the water-soluble copolymer, poly(oligoethyleneglycol methacrylate-*co*-hydroxyethyl methacrylate, enabled the emission to be spectrally tuned using humidity (Figure 96e), or reversibly switched between lasing and fluorescence (Figure 96f).

### 10.5 Summary

The range of optical effects and functions that can be achieved using photonic CNC films is expanded by combining CNCs with additional materials to make composites, either as a pre- or postprocessing step. By localizing the application of these treatments, it is possible to impart patterning into the film (e.g., embossing, localized doping), which could unlock additional visual effects for e.g. anticounterfeiting or colorimetric sensing.

However, the range of additives that can be included by coassembly is constrained by the need to preserve CNC colloidal stability and cholesteric ordering. Furthermore, it is important to reiterate that while a wide range of functional additives have been explored, their effect can often be approximated to that of a “nonvolatile solvent” that prevents geometric collapse of the kinetically arrested structure, following a predictive relationship between volume fraction and the pitch of the film. Moreover, while many of these additives can increase the water uptake of CNC films, allowing for them to be promoted as humidity sensors, it is relevant to note that often a significant color change is only observed at fairly high relative humidity (e.g., > 80%), limiting their feasibility.

While modification of the CNCs themselves has only been demonstrated in simple ways, such as via heat-treatment, there remains significant scope to functionalize the CNCs to tune the properties of the resultant films. However, this has not been widely exploited by the community due to the significant effects such modifications can have on the colloidal properties of the CNCs and thus their self-assembly behavior.

## 11 The Self-Assembly of Chitin Nanocrystals for Photonics

Chitin is a polysaccharide found in many organisms (e.g., arthropods), where it is often arranged into helicoidal nanoarchitectures for a variety of functions, including mechanical reinforcement and structural coloration.^814,863^ However, despite chitin being both highly abundant and biodegradable, the potential of sustainable chitin-based photonic materials has not yet been realized. Analogous to CNCs, naturally derived chitin nanocrystals (ChNCs) form a left-handed cholesteric liquid crystal phase in aqueous suspension, and can therefore be used to produce nanostructured films. However, ChNCs differ from CNCs in terms of surface chemistry and crystal structure, and therefore independent investigation of both their self-assembly behavior and optical performance in the solid state is required to achieve photonic films.

This section starts by introducing chitin as a sustainable resource, its hierarchical ordering in natural organisms, and the key steps required for its extraction. Chitin nanomaterials are then introduced, with an emphasis on the colloidal and liquid crystalline behavior of ChNCs. Finally, chitin-based photonic materials are described and contrasted with those from CNCs, with strategies to improve their performance.

### 11.1 Chitin as a Resource

**Figure 97:**
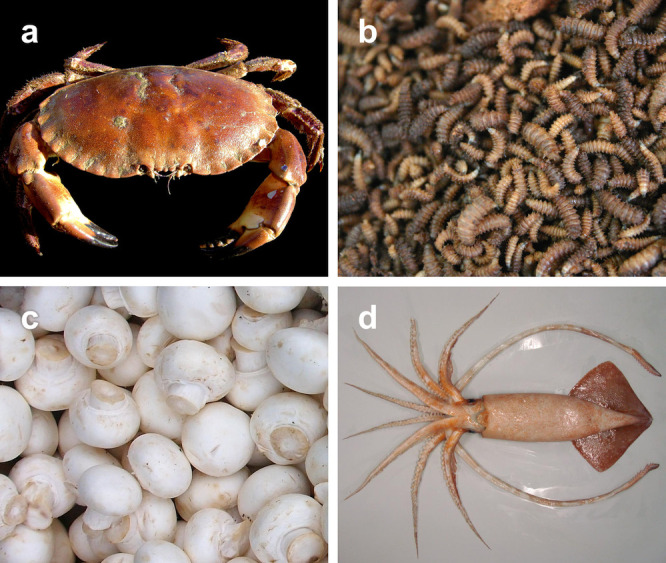
Natural sources of α-chitin include the exoskeletons of (a) crustaceans and (b) insects, as well as the cell walls of (c) fungi. The most prominent source of β-chitin is (d) mollusks such as cephalopods. Reproduced under Creative Commons CC BY-SA 4.0, BY-SA 3.0, BY-SA 2.5, and BY-SA 3.0, respectively.

Chitin is predominantly found in the exoskeletons of arthropods (e.g., crustaceans, insects, and arachnids), in the beaks of mollusks (e.g., cephalopods), as well as in fungi (Figure 97), where it serves primarily mechanical and structural functions.^864^ Commercially, chitin is largely sourced from the marine food industry as a waste product from crustacean shells, as the inedible exoskeleton corresponds to approximately 60% of the total crustacean mass.^865−868^ In 2020 alone, approximately 17 million tonnes of crustaceans were harvested globally, with fisheries contributing approximately 6 million tonnes, and aquacultures 11 million tonnes.^869^ From the resulting 10 million tonnes of crustacean waste, up to 3 million tonnes of chitin could potentially be extracted. These figures are expected to rise in the years to come, as the demand for seafood has been steadily increasing over the last several decades.^869^ In addition, emerging initiatives toward large-scale farming of insects and fungi are likely to significantly increase the availability of chitin as a resource.^870−872^

### 11.2 Hierarchical Structure of Chitin

**Figure 98:**
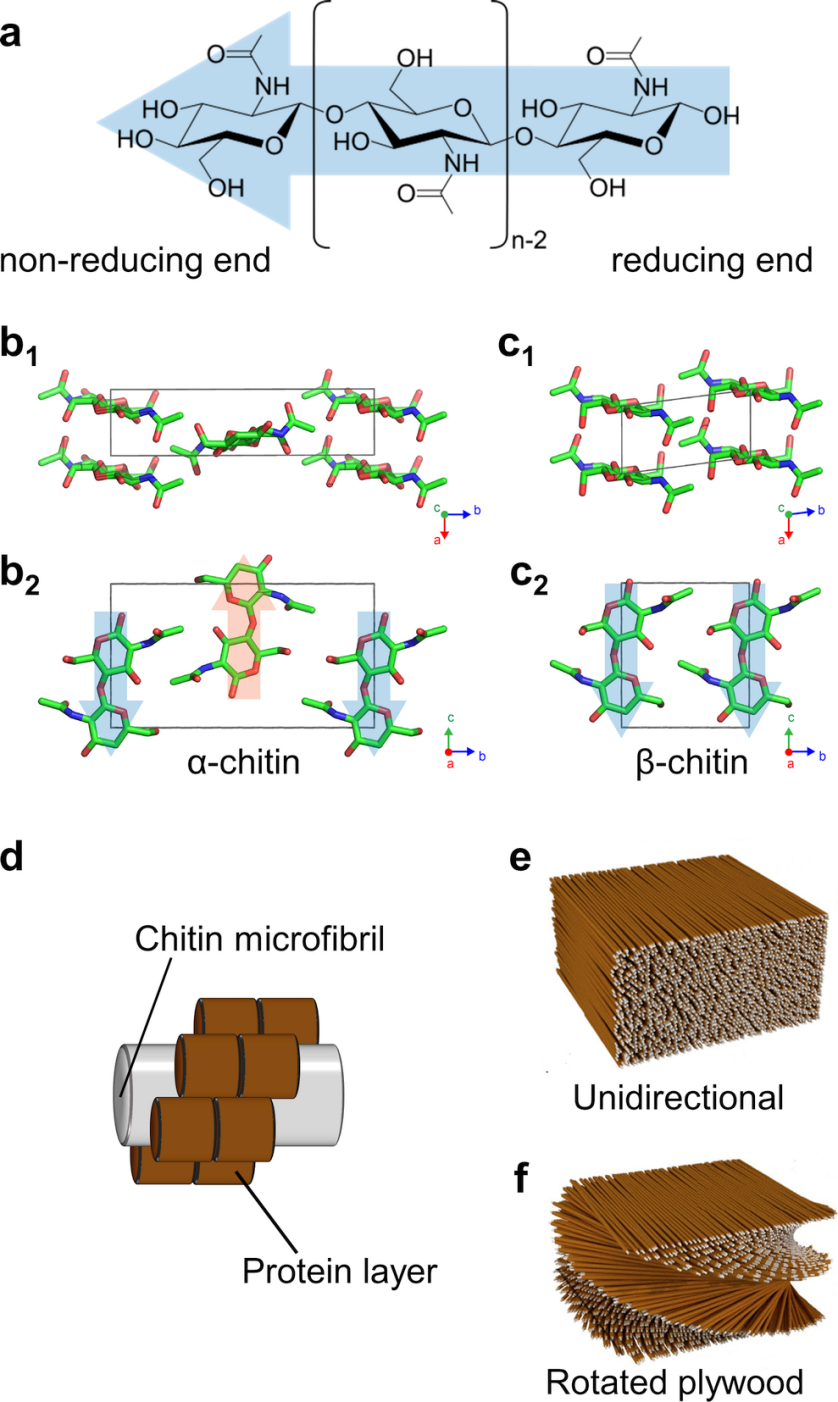
Hierarchical architecture of chitin in the arthropod exoskeleton. (a) Molecular structure of a chitin chain, illustrated as the building block of chitin allomorphs. (b, c) Crystal structures of (b) α-chitin and (c) β-chitin, in the cross-section (b_1_, c_1_) and side (b_2_, c_2_) projections. Arrows indicate the molecular polarity of cellulose chains. (d) In the organism, crystalline chitin microfibrils are enveloped by proteins, and then further arranged into higher-order structures, such as (e) unidirectional or (f) helicoidal architectures. Adapted from ref (895) under Creative Commons CC-BY. Copyright 2021 The Authors.

Chitin is a linear polymer composed of *N*-acetyl-d-glucosamine monomers connected via β-1,4 glycosidic bonds (Figure 98a), with a degree of polymerization that varies between source materials and typically exceeds 1,000.^873^ Naturally occurring chitin is found in the form of elongated crystalline domains known as microfibrils, which contain one of two crystal allomorphs, denoted α and β.^874^ The most abundant and thermodynamically stable allomorph is α-chitin, in which the chains are arranged in an antiparallel fashion to form stacked sheets held together by hydrogen bonds (Figure 98b).^875^ This crystalline structure results in exceptional mechanical properties despite a relative low density (1.425 g/mL).^876^ For example, the Young’s modulus of a crystalline α-chitin microfibrils along the chain axis was calculated to be around 120 GPa based on DFT calculations, and experimentally determined to be 60 ± 10 GPa using X-ray diffraction.^877^ The transverse modulus is calculated to be much lower, around 30 GPa, as the crystal structure in this direction is only stabilized by noncovalent interactions (primarily hydrogen bonds).^878,879^ Furthermore, the stability conferred by the intersheet hydrogen bonding network inhibits swelling in most solvents, making chitin generally insoluble, and thus the majority of chemical reactions are restricted to the microfibril surface. Investigation of native α-chitin from arthropod cuticle using X-ray diffraction and TEM suggests that the microfibrils comprise 18–25 tightly packed molecular chains, resulting in a polygonal cross-section with a diameter of 2–5 nm (usually 2.8 nm when measured from TEM images) and a typical length of over 300 nm.^874,875,880−884^ The β-chitin allomorph is significantly less common than α-chitin, and is found mainly in squid beaks, diatom spines, tubes of pogonophora, or insect trachea.^864^ It is also less thermodynamically stable due to the parallel arrangement of chitin chains (Figure 98c), which obstructs the intersheet hydrogen bonding.^874,885,886^ As such, β-chitin is more susceptible to swelling in polar solvents. Furthermore, it can be irreversibly converted into α-chitin upon treatment with a concentrated acid or base.^874^ The predominance of α-chitin raises questions about the mechanism of its biosynthesis, as β-chitin, as well as naturally occurring cellulose allomorphs (see section 2.1), are composed of parallel chains.^875^ For conciseness, whenever referring to chitin in the following text, α-chitin is implied unless otherwise stated.

In natural biomaterials, chitin microfibrils form part of a composite hierarchical material.^887,888^ For example, chitin constitutes around 30% of the arthropod exoskeleton by weight, with proteins, minerals and water accounting for the remainder (with precise composition varying with the species and tissue chosen).^883^ In the arthropod cuticle, some proteins include specific chitin binding domains that enable them to tightly envelop the chitin microfibrils (Figure 98d).^889−892^ Such microfibrils enveloped by chitin binding proteins are usually found to be arranged into one of two principal architectures.^874,883,893^ The unidirectional architecture (Figure 98e) is typically found in the arthropod tendons, though some arthropods also include it into their exoskeleton (e.g., wasp ovipositors^892^ or locust tibia^894^) impacting their optical or mechanical properties.^814,878,883^ In contrast, the helicoidal architecture (Figure 98f) is found in the cuticles of almost all arthropods, where it can vary in pitch not only between species but also between different body parts of the same organism.^883,893^ This “twisted plywood” arrangement improves the mechanical properties of the cuticle, by increasing resistance to crack propagation or intense compressive forces.^878^ Importantly, when the pitch is on the length scale of visible light (i.e., 250–450 nm), the helicoidal architecture can also provide structural coloration, as most commonly observed in scarab beetles.^863^

### 11.3 Purification of Chitin

As chitin is almost always found as part of a composite with proteins and minerals, it must first be purified. This is generally achieved by a combination of physical processing techniques (e.g., cleaning, size reduction by milling, heating, drying) and chemical treatments (e.g., demineralization, deproteinization, and depigmentation), as extensively reviewed elsewhere.^864,867,868^ Typical reagents include strong mineral acids, alkalis, and bleaches, which could chemically alter chitin and its downstream products if applied too aggressively. However, mild conditions for demineralization (0.25 M HCl at RT) and deproteinization (1 M NaOH at 50–70 °C) have been shown to be sufficient to obtain purified chitin, although several cleaning cycles may be required to obtain a high-purity product.^870,896^ The purification process is especially important for fungi-derived chitin, as some bound polysaccharides, known as β-glucans, are not removed by the standard purification methods. However, these β-glucans are reportedly still susceptible to hydrolysis at highly acidic conditions.^897^ Biological processes can also be employed to purify chitin in a more sustainable manner that minimize energy and water usage, though such processes are typically time-consuming.^865,898,899^ Regardless of the method employed, purified chitin is found to be partially deacetylated, and therefore differs from the idealized molecular structure depicted in Figure 98a. The degree of acetylation (DA), which describes the proportion of amides relative to amines, is typically 90–95%, as identified by solid state NMR spectroscopy, infrared spectroscopy or conductometric titration. This partial deacetylation implies that either naturally occurring chitin is already slightly deacetylated or that this is a consequence of the purification process.^864,868^ Highly deacetylated chitin (with DA below 50%) is referred to as chitosan, which has markedly different material properties of interest for various applications.^864,868,900^

### 11.4 Colloidal Suspensions of ChNCs

**Figure 99:**
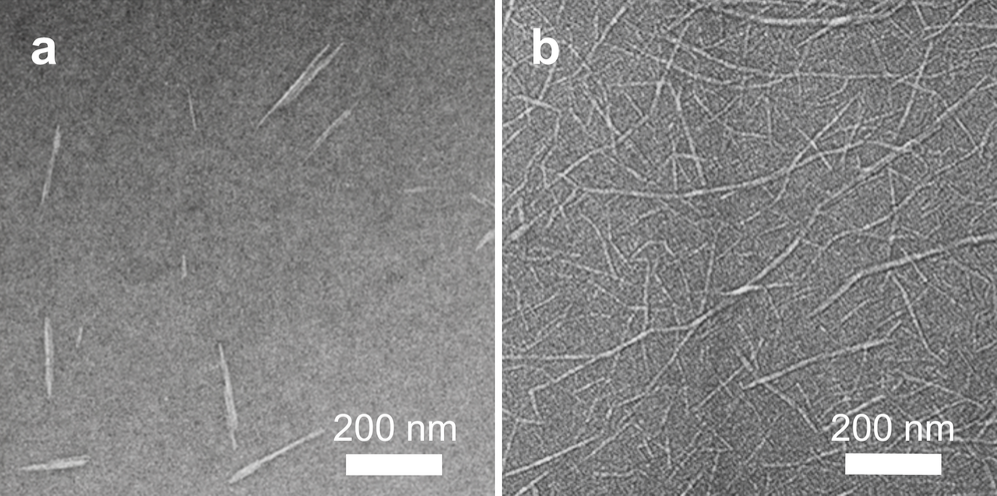
Nanomaterials of chitin. Representative negatively stained TEM micrographs of (a) polycrystallite ChNCs produced from crustacean α-chitin. Reproduced with permission from ref (270). Copyright 2019 American Chemical Society. (b) High-aspect-ratio ChNFs. Adapted with permission from ref (904). Copyright 2014 American Chemical Society.

Chitin nanomaterials, or nanochitin, can be broadly divided into two categories: chitin nanocrystals (ChNCs)^19,22,270,447^ and chitin nanofibers (ChNFs).^25,901−903^ ChNCs, which are sometimes referred to as chitin nanowhiskers or chitin nanospindles, are typically a few hundred nanometers in length and tens of nanometers in width, exhibiting an aggregated morphology (Figure 99a).^22^ In contrast, ChNFs are typically several micrometers long and several nanometers wide and thus resemble the microfibrils found in the arthropod cuticle (Figure 99b).^870,904^ As ChNFs have been thoroughly reviewed elsewhere,^25,901−903^ and have not yet been reported to form cholesteric mesophases, the following discussion will focus solely on ChNCs.

ChNCs can be produced from virtually any natural chitinous resources, though the majority of studies employ chitin from crustacean shells^22^ or fungi.^905^ In a standard procedure, purified shrimp chitin is hydrolyzed in 3–5 M HCl under reflux at a solid:liquid ratio of approximately 1:30.^19,22,270,447^ The reaction is allowed to proceed from 1.5 to 10 h, prior to quenching using cold deionized water. The suspension is then further processed by several cycles of centrifugation to remove soluble residue and acid, dialyzed against deionized water to remove excess ions and finally ultrasonicated to disperse aggregates.^19,22^ This process has a typical yield of 65–88 wt %, which is found to reduce with the harshness of the conditions.^270^ While the optimal conditions depend on the source, hydrolysis of shrimp chitin using 3 M HCl for 4.5 h has been reported to yield suspensions with good colloidal stability that can also form a cholesteric mesophase.^270^ The resulting ChNCs are highly polydisperse in size and appear to be made of bundles of individual chitin crystallites. ChNCs obtained by the protocol above were reported to have lengths of 184 ± 98 nm (coefficient of variation σ̂ = 0.53) and widths of 14 ± 9 nm (σ̂ = 0.64), with the number of surface exposed amines amounting to 233 mmol/kg_ChNC_.^22,270^ Milder hydrolysis conditions (e.g., 3 M HCl, 1.5 h) can lead to particle sedimentation, especially at larger nanoparticle concentrations, likely due to poor colloidal stability or remnants of unhydrolyzed chitin.^22,270,447^ Conversely, excessively aggressive conditions (e.g., 5 M HCl, 4.5 h) can lead to overhydrolysis, as evidenced by severe discoloration of the produced ChNC suspension.^270^ In summary, the hydrolysis conditions have a strong influence on the colloidal and liquid crystalline properties of the suspension.

ChNCs prepared by HCl hydrolysis, unlike CNCs, have ionizable amine groups on the crystallite surface, which originate from a combination of imperfect acetylation of natural chitin (typically 90–95% as discussed above, the purification process, and some degree of deacetylation occurring during acid hydrolysis.^22,270,864^ ChNCs are colloidally stable below pH 6 due to the protonation of surface amine groups, which have average p*K*a value of 6.0 ± 0.2 and a typical density of 150–300 mmol_NH2_/kg_ChNC_.^22,270,447,906^ Despite the stabilizing effect of the highly positively charged ChNC surface at low pH, aggregation occurs below pH 2 due to the excessively high ionic strength of the medium.^447^ Analogously, adding monovalent salts (such as NaCl) beyond concentrations of 5–10 mM has been reported to induce aggregation.^447,905,907^

### 11.5 Self-Assembly of Chitin Nanocrystals

ChNC suspensions are known to exhibit lyotropic cholesteric behavior,^22^ analogous to that reported for CNC suspensions, and much of the discussion in sections 3–6 is therefore directly applicable. However, ChNCs differ from CNCs in several ways, most notably their pH-dependent surface charge and larger cholesteric pitch, which necessitates greater understanding of the evolution of the cholesteric phase during the self-assembly process. In this section, key parameters affecting ChNC cholesteric mesophases are summarized.

#### 11.5.1 Impact of Chitin Source

While ChNCs can be produced from a variety of sources, nearly all studies on cholesteric ordering in ChNC suspensions have used chitin originating from crustaceans.^22,270,447^ A notable exception is a recent study comparing ChNCs derived from fungi and shrimp.^538^ Fungi-derived ChNCs were found to have a higher aspect ratio than shrimp-derived ChNCs, when prepared under similar hydrolysis conditions. This resulted in lower biphasic threshold concentrations (i.e., smaller *c*_*b*1_ and *c*_*b*2_) and a narrower biphasic region (i.e., smaller Δ*c*_*b*_). Furthermore, fungi-derived ChNC suspensions had significantly lower pitch values when compared to suspensions made from shrimp chitin at similar concentrations. While the origin of this smaller pitch is not clear, it is noteworthy that the fungi-derived chitin was never dried, whereas the shrimp chitin was purchased as partially purified dry powder and then further purified. Additionally, chitin microfibrils from fungal cell walls are known to have tightly bound amorphous polysaccharides (e.g., β-glucans) that are resistant to removal during purification and may therefore still be present in the resulting ChNCs.^897^ As a final comment, cholesteric self-organization of ChNCs derived from insects, arachnids, or β-chitin has not been reported to date.

#### 11.5.2 Impact of Hydrolysis Conditions

Broadly speaking, more extensive hydrolysis (i.e., increasing the duration or acid concentration) leads to shorter and thinner ChNCs with a higher surface charge.^22,270^ An investigation of the impact of hydrolysis conditions on ChNC phase behavior reported that the hydrolysis duration and acid concentration had little effect on the first biphasic threshold *c*_*b*1_, while the second threshold *c*_*b*2_ moved to higher ChNC concentrations with more aggressive hydrolysis conditions, suggesting an increasing proportion of low-aspect-ratio ChNCs. Furthermore, the cholesteric pitch of these biphasic suspensions was found to be correlated with ChNC surface charge.

#### 11.5.3 Impact of Surface Modification

The maximum possible surface charge of ChNCs is determined by the density of amine groups, which are protonated under acidic conditions.^22^ As discussed in section 11.3, purified natural chitin is always partially deacetylated (DA = 90–95%) and the resulting ChNCs thus have a surface amine density of ca. 250 mmol_NH2_/kg_ChNC_. This can be increased by further deacetylation of surface amides using a strong base treatment (e.g., KOH),^908−910^ which can be applied to the chitin source material or to the ChNCs after hydrolysis.

Deacetylation of the chitin source material prior to hydrolysis does not substantially change the surface charge of the resulting ChNCs, but reduces their length and width and therefore increases their aspect ratio.^270,911^ These observations suggest that the deacetylated surfaces are etched away during hydrolysis, exposing the native (i.e., more acetylated) chitin beneath. ChNCs prepared from deacetylated chitin were found to have a higher second threshold concentration, *c*_*b*2_, and a consistently lower cholesteric pitch.^270^

Either by applying shorter hydrolysis time to reduce the surface etching effect or directly deacetylating ChNCs, their dimensions and morphology can be preserved while the surface charge is increased.^908−911^ With the increase in surface charge, the biphasic threshold concentrations move to higher values. For example, *c*_*b*1_ significantly increases from 3.0 wt % for nontreated ChNCs to 6.0 wt % for ChNC with surface charge of around 400 mmol_NH2_/kg_ChNC_ (both samples had 0.5 mM HCl per wt% of ChNC).^909^ However, the phase-separation of such higher-charged ChNCs is compromised as it fails to phase separate clearly beyond concentrations of ca. 7.5 wt %. Overall, ChNCs with surface charge beyond ca. 350 mmol_NH2_/kg_ChNC_ start to exhibit compromised self-organization, possibly because the high viscosity of the suspension hinders phase separation.^909,910^ Interestingly, while the boundary concentrations are strongly affected, the cholesteric pitch seems to remains mostly unchanged.^909^ For ChNCs to successfully self-organize, it is also important that the surface charge is not too low, as illustrated by a study on ChNCs with sulfonated amines.^912^ Such suspensions were stable at basic pH and started to exhibit self-assembly when the surface charge reached values equivalent to ca. 200 mmol_NH2_/kg_ChNC_.^912^ However, with lower surface charges, sulfonated ChNCs suspensions appeared to lack sufficient colloidal stability to allow self-organization to occur. Overall, it seems that ChNCs form a cholesteric phase only in a limited range of surface charge, roughly 200–350 mmol_NH2_/kg_ChNC_ and while the exact values may stretch depending on the pH, the ionic strength and the reparation conditions, it helps to explain the lack of liquid crystalline behavior for highly charged ChNFs or ChNCs.^270,908,910^

#### 11.5.4 Impact of Fractionation

The phase separation behavior of ChNC suspensions can be applied to fractionate ChNCs based on their aspect ratio, in analogy to CNCs (see section 8.2.3).^913^ When comparing ChNC sizes using the hydrodynamic diameter *D*_hyd_ determined by DLS, an initial suspension with *D*_hyd_ = 243 nm was separated into fractions with *D*_hyd_ ranging from 170 to 293 nm. An investigation of the phase separation of the fractionated suspensions revealed that higher-*D*_hyd_ fractions separated rapidly (around 1 day), whereas the lowest-*D*_hyd_ fraction was not fully separated after 1 week. The biphasic threshold concentrations, and the cholesteric pitch at a given concentration, were reported to increase with decreasing *D*_hyd_, similar to the behavior seen with fractionated CNC suspensions. Assuming a linear relation between helical twisting power (HTP, see sections 3.3.3 and 8.2.2–8.2.3) and hydrodynamic diameter (i.e., HTP = *k*′ *D*_hyd_), a scaling factor of *k*′ = 0.015 was reported, which is an order of magnitude smaller than the fitting parameter between HTP and length (HTP = *kL*) reported for CNCs.^272^ While this suggests that ChNCs have a weaker chiral interaction than CNCs, the hydrodynamic diameter is a crude measure of particle morphology, and more detailed characterization of individual ChNCs is required to validate these findings.

#### 11.5.5 Impact of the Suspension Formulation

**Figure 100:**
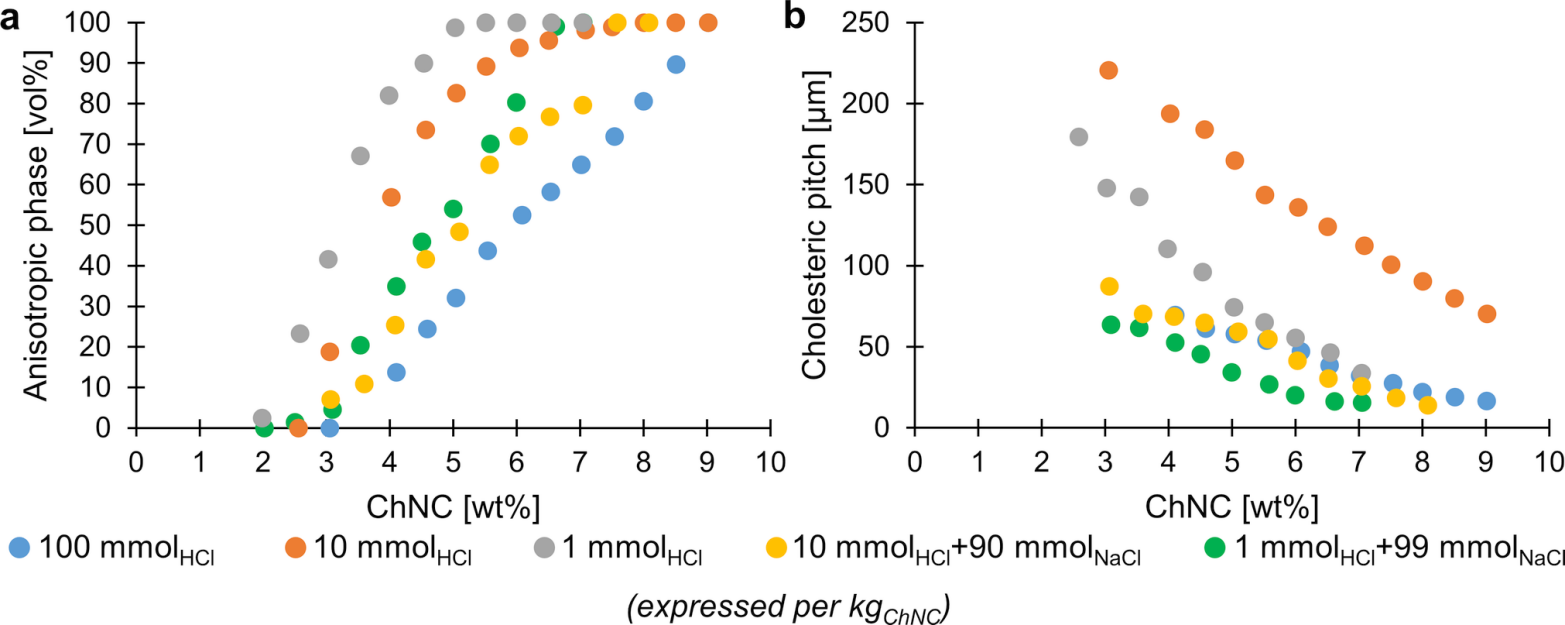
Liquid crystalline behavior of ChNCs is tunable via suspension formulation. (a) The proportion of the anisotropic phase formed and (b) the cholesteric pitch in suspension can be tuned via added HCl and NaCl (expressed in mmol per kg of ChNC). Data from ref (270).

Due to the nature of the ChNC surface charge, the colloidal and liquid-crystalline properties of ChNC suspensions are highly sensitive to *both* ionic strength and pH (Figure 100a,b), which can be adjusted either via dialysis or direct addition of electrolytes (e.g., NaCl, HCl) into the suspension.

First, independent of the effect of pH, increasing the ionic strength shifts the biphasic thresholds, *c*_*b*1_ and *c*_*b*2_, to higher concentrations, and significantly reduces the pitch.^270,447,914^ Second, the pH of the suspension can be used to vary the ChNC surface charge while keeping the total ionic strength constant using NaCl. In this case, the second biphasic threshold *c*_*b*2_ shifts to higher concentration with increasing surface charge, while the pitch slightly increases.^270^ However, if only HCl is added, the surface charge is varied without compensating for the associated change in ionic strength, and these competing effects lead to more complex variations in pitch values (Figure 100b).

#### 11.5.6 Impact of Ultrasonication

Ultrasonication of ChNC suspensions decreases their turbidity but without a significant increase in conductivity or cholesteric pitch.^270^ Drawing analogy to CNCs (see section 8.2.2), this would suggest that ultrasonication breaks apart large disordered aggregates, but not the crystallite bundles that are believed to mediate cholesteric ordering, or that the HTP of ChNC bundles is significantly weaker than the HTP of CNC bundles. However, further investigation is needed to elucidate the morphological changes of ChNCs upon ultrasonication.

#### 11.5.7 Origin of Mesophase Chirality in ChNC Suspensions

The cholesteric phase in ChNC suspensions is invariably left-handed,^22,447,538^ but how this mesophase chirality arises from the morphology of the individual ChNCs is not currently totally clear. Analogous to CNCs (section 3.3), the microscopic twist on individual ChNCs is expected to play a role. Molecular dynamic simulations interestingly suggest that when around 20 molecular chains are arranged in the α-chitin like crystal, the energy start to plateau and as the crystal diameter increases, the axial-chirality of the α-chitin microfibril decreases, nevertheless, the simulated configuration resembling that of naturally observed α-chitin in arthropod cuticle has a small axial chirality. The chirality originates from the intrinsic chirality of chitin molecules, where stacking along the *a*-direction of the crystal preserves the chirality whereas stacking along the *b*-direction suppresses the chirality transfer from the molecule to the nanofibril, which results from the dipole–dipole interactions between acetamide groups (Figure 98b). The simulated twists along the α-chitin fibril is right-handed and is calculated to be 15.4 ± 5.8° per simulated length of 10.6 nm (1.5 ± 0.5°/nm), which can be then recalculated that the pitch of the α-chitin microfibril averages to 250 nm (with minimum of 180 nm and maximum of 400 nm).^915^ The twisting morphology of ChNCs was confirmed by cryogenic electron tomography with twisting angle observed to be of 0.3–0.8°/nm, with twist being generally observed to be higher for shorter nanoparticles.^911^ Nevertheless, the measured twist is smaller when compared to molecular dynamic simulations, though it is important to take into account that the simulated nanofibril was around 30 times shorter than the actual one that could have affected the simulated twist.^915^ Lastly, the tomographic data did not appear to show that there is any preferential bias in the ChNC twisting handedness, which is surprising, given exclusive formation of the left-handed cholesteric structures.^911^ Notably, the ChNCs used in this study were produced from never-dried crustacean chitin and did not reportedly form a cholesteric phase. However, never-dried chitin, albeit of fungal source, was shown to form cholesteric phases.^538^ Nevertheless, further investigations are warranted to reveal the origin of chirality in ChNC mesophases, both in terms of computer simulations and single-particle characterization. However, experimental determination of ChNC helical twisting power is expected to be challenging, as the large pitch values typically observed in ChNC suspensions (up to several hundred microns^270,447^) imply only a weak morphological chirality, which can be affected by the sample processing such as material drying.

### 11.6 Helicoidal ChNC Films

Analogous to the cholesteric self-assembly of CNCs, the ChNC cholesteric phase evolves as the suspension dries until it reaches a point of kinetic arrest, beyond which the ChNCs are unable to rearrange to reach thermodynamic equilibrium.^22,270,416,437^ Further water loss leads to uniaxial compression of the structure until a dried film is obtained with a final pitch around 1 order of magnitude smaller than in suspension.

**Figure 101:**
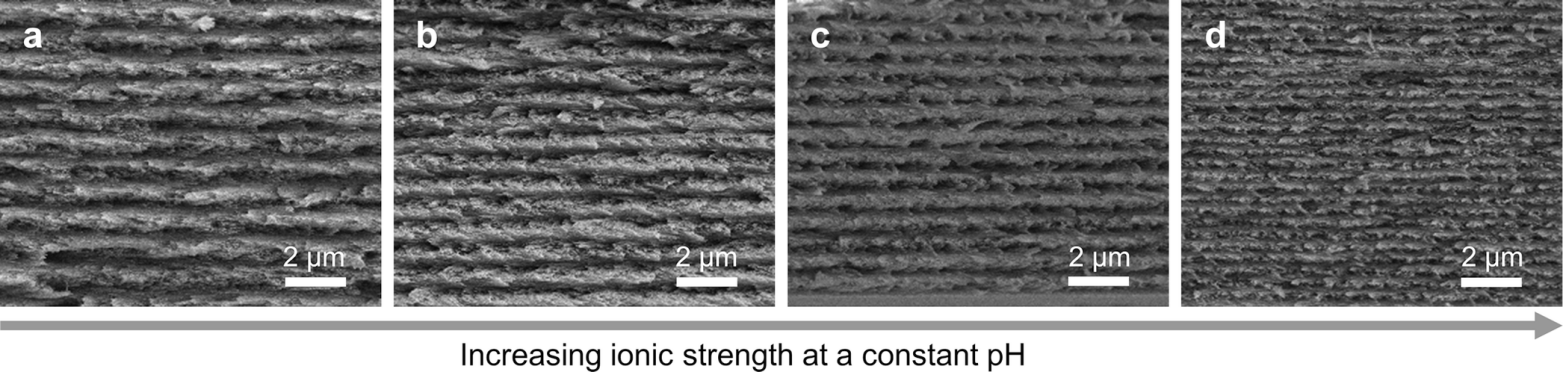
Pitch in ChNC films can be tuned by adding increasing amount of NaCl of (a) 30, (b) 60, (c) 90, and (d) 120 mmol/kg_ChNC_, with the amount of added HCl kept constant to 100 mmol/kg_ChNC_. Unpublished data for ChNCs produced from shrimp shell powder by the method described in ref (270).

To achieve visible structural coloration from helicoidal ChNC films, pitch values in the 240–480 nm range are required, given that the refractive index of chitin ranges from 1.535 to 1.572 in the visible spectrum.^916^ As discussed in section 11.6, the cholesteric pitch in suspension can be reduced by adding electrolytes (e.g., NaCl), which directly translates into a smaller pitch in the final film (Figure 101).^270^ However, applying these strategies to crustacean-derived ChNCs has resulted in films with pitch values still too large for visible coloration (i.e., *p* = 650 to 4,000 nm),^22,270,538^ although such helicoidal films may be useful for mechanical applications. In this section, we summarize recent approaches to overcome this limitation and achieve visible structural color.

**Figure 102:**
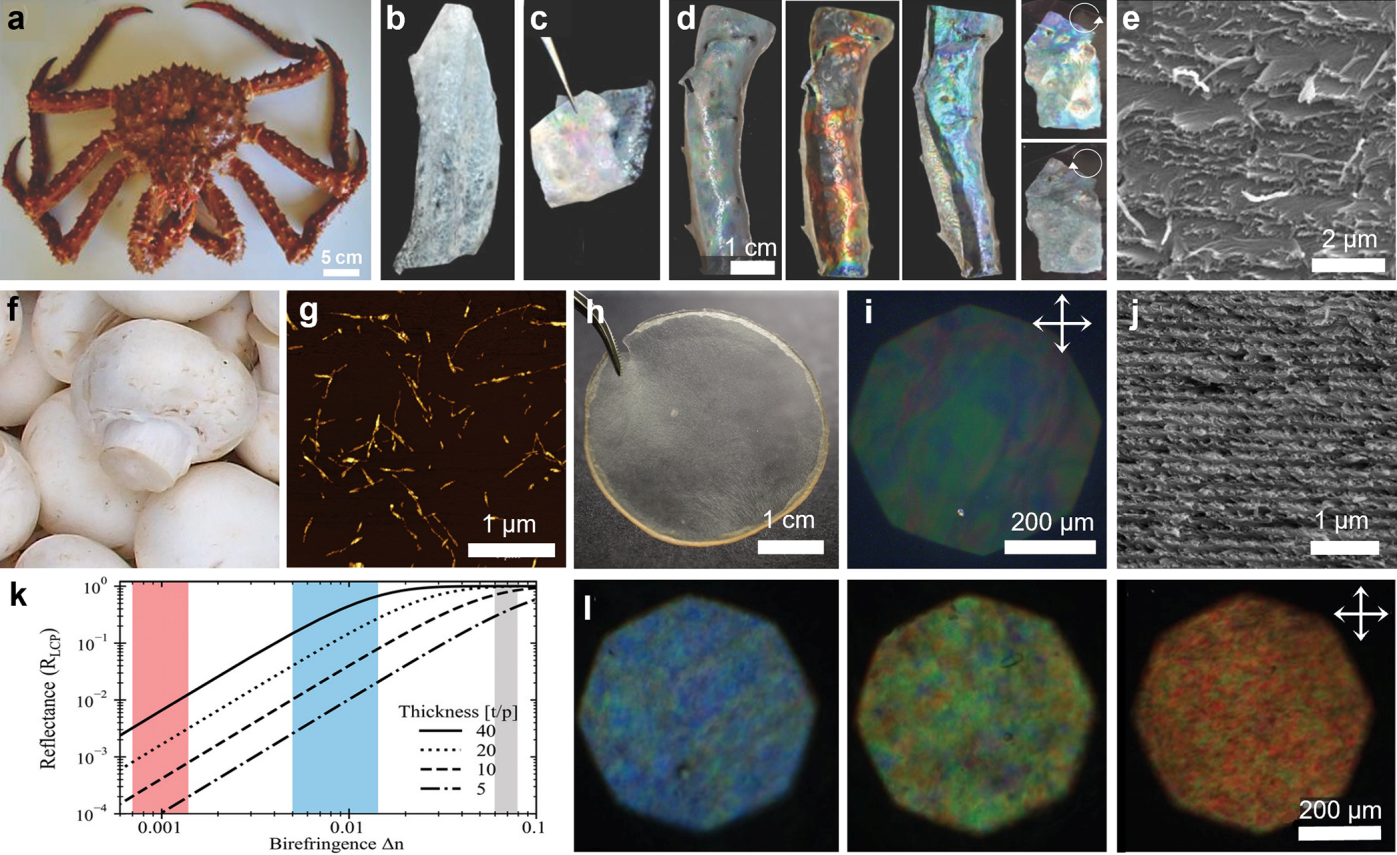
Structurally colored materials derived from chitin. (a) King crab (*Paralithodes camtschaticus*). (b) Purified inner cuticle extracted from king crab with weak structural coloration. (c) Stronger coloration of (b) after wetting. (d) Enhancement of the structural coloration of the cuticle and LCP-selective reflection following successive rounds of deacetylation. (e) SEM cross-section of the deacetylated cuticle preserving the native helicoidal architecture. Adapted with permission from ref (917). Copyright 2014 John Wiley and Sons. (f) Common mushroom (*Agaricus bisporus*) from which fungal ChNCs can be extracted. Reproduced under CC BY-SA 2.5. (g) AFM image of fungal ChNCs. (h) Helicoidal film from fungal ChNCs, displaying no apparent coloration. (i) POM image of the films in (h) viewed between crossed polarizers. (j) SEM cross-section of the film in (h), displaying a helicoidal structure with submicron pitch. (k) Calculated reflectance of an ideal cholesteric monodomain viewed between crossed polarizers, *R*_XP_, as a function of local birefringence and domain thickness. The red shaded region corresponds to the estimated intrinsic birefringence of chitin, while the blue shaded region corresponds to the estimated intrinsic birefringence after full deacetylation into chitosan. (l) POM images of deacetylated ChNC films viewed between crossed polarizers in reflection. Reproduced from ref (538) under Creative Commons CC-BY license. Copyright 2022.

One approach to achieving smaller pitch values in chitin-based helicoidal structures is to exploit the native helicoidal architecture found in organisms such the king crab (*Paralithodes camtschaticus*) (Figure 102a).^917^ While the native pitch is too large for reflection in the visible range, the removal of proteins and minerals leads to shrinking of the helicoidal structure, resulting in weak coloration in the dry state (Figure 102b), which can be enhanced slightly upon wetting due to an increase in form birefringence and reduction of incoherent scattering (Figure 102c). Interestingly, the reflectance from these naturally derived helicoidal samples could be greatly enhanced by successive cycles of deacetylation, which progressively convert chitin into chitosan (Figure 102d).^917,918^ The selective reflection of LCP light of these structures as well as the observation of the Bouligand texture in cross-sectional SEM (Figure 102e) confirm the preservation of the native helicoidal architecture.^917−919^ However, this top-down approach suggests that achieving smaller pitch values alone is insufficient to obtain intense structural coloration from ChNC films.

Recently, helicoidal films with small pitches have been achieved via the self-assembly of ChNCs derived from never-dried fungal chitin (Figure 102f).^538^ Although the films appear transparent to the naked eye (Figure 102h), polarized optical microscopy revealed faint structural coloration (Figure 102i), with a peak wavelength consistent with the pitch measured from SEM cross-sections (Figure 102j). The weak reflectance of these ChNC films can be largely attributed to the low intrinsic birefringence of chitin (Δ*n* = 0.001–0.005),^565,814,920^ which is more than 1 order of magnitude smaller than that of cellulose (Δ*n* = 0.064–0.081).^223,337^ As discussed in section 7.3.1, the maximum LCP reflectance  for an ideal cholesteric monodomain is given by , where *t* is the domain thickness. Curves based on this equation while varying *t*/*p* and Δ*n* are shown in Figure 102k. Given the low intrinsic birefringence of chitin, a ChNC helicoidal monodomain would need to be over 200 μm thick to reflect the majority of the incident LCP light. While the alternating electric fields unwind the cholesteric phase of ChNCs into well-ordered nematic phase within seconds,^921^ magnetic fields can improve the alignment of the ChNC cholesteric phase.^447,909,913^ But even with the magnetic fields, such thick ChNC domains would be difficult to obtain in practice, and their photonic properties would be impaired by the absorption of chitin. Consequently, pure chitin is not a prime material for helicoidal photonic materials.

In nature, this limitation of chitin is sometimes resolved by the inclusion of nonchitinous materials (e.g., proteins, uric acid nanocrystals), which enhance local birefringence.^814^ In practice, the conversion of chitin into chitosan has been shown to significantly improve the optical signal for several helicoidal chitin-based materials, including films produced via ChNC self-assembly.^538,917,918^ The observation can be attributed to the birefringence of chitosan (ca. 0.015), which is about ten times higher than that of chitin (Figure 102k).^538,565,922^ Deacetylation of ChNC helicoidal films also leads to a significant mass loss, which causes a reduction of the helicoidal structure pitch and a blue-shifted optical response.^538^ As such, it provides an additional way to achieve smaller pitch values, which is otherwise difficult for ChNCs, and thus tune the final reflection band across the visible range (Figure 102l). Interestingly, it also allows for achieving red-reflecting films from crustacean-derived ChNC films, which usually have an excessively large helicoidal pitch to reflect visible light.^538^ At present, chitosan photonic films do not compare favorably to those made from cellulose, as the birefringence of chitosan is around 4-fold lower (Figure 102k). However, the use of chitosan creates opportunities for chemical modification not available with cellulose, and further investigation is therefore needed to explore the potential of chitosan-based photonic materials.

### 11.7 Summary

The chiral self-assembly of ChNCs shares many analogies with CNCs but also specificities, and thus represents a valuable system to explore further. From a fundamental point of view, their comparison with CNCs can help understand the origin of chiral interactions in twisted elongated colloidal particles and polysaccharide nanocrystals. The chiral arrangement of chitin plays a role in natural chitin-based materials, such as the solid chiral plywood architectures naturally found in crustacean and arthropod cuticles, where they are mostly revelvant for their anticrack propagation properties,^923,924,924^ but their cholesteric properties in suspension can also be relevant to understand how chitin assembles in vivo. ChNC films can find applications as a suitable chiral templating agent where CNCs cannot be used, e.g., in coassembly with positively charged species.

The larger pitch and lower birefringence of ChNCs make them less suitable to produce structural color, but these limitations can be partially overcome by various methods (using fungal chitin source, postcasting deacetylation or coassembly with species of contrasting refractive indices). Despite these treatments, the resulting reflection remains much less intense than that of typical CNC films. This shortcoming, leading to nearly transparent films, could potentially become an advantage if ChNC films are used as a chiral template, where the optical response of the template itself may not be desirable.

## 12 Overview and Outlook

### 12.1 Summary

The widespread concern over the impact of human activity on the environment has resulted in a desire to replace artificial functional materials with naturally derived alternatives. As such, polysaccharides are drawing increasing attention due to offering a renewable, biodegradable, and biocompatible route to functional nanomaterials, which is key to transitioning to a more sustainable society. Over the past few decades, nanocrystals of cellulose and chitin have emerged as versatile and sustainable nanomaterials for diverse applications, ranging from mechanical reinforcement and emulsion stabilization to structural coloration and optics. Much of this interest has arisen from the tendency of these colloidally stable nanoparticles to self-organize in water into a lyotropic cholesteric liquid crystal, which can be readily manipulated in terms of its periodicity, structure, alignment, and geometry. Upon drying, this helicoidal ordering can be retained into the solid-state, offering an accessible route to complex nanostructured films, coatings, and particles.

In this review, we have focused primarily upon the application of cellulose nanocrystals to the development of sustainable photonic materials. While the pathway to produce a colored film from CNCs often appears trivial in the literature, each of the intermediate steps, from hydrolyzing cellulose, to forming an isotropic suspension of colloidally stable CNC nanoparticles, through its self-organization into a cholesteric liquid crystal, its kinetic arrest and ultimately its drying into a solid film, is complex and is affected by a diversity of intrinsic and extrinsic factors. Moreover, while there is a common toolset employed by the community to empirically tune the CNC suspension (salt, ultrasonication, additives, etc.) to produce the desired color in the resultant film (exclusively in terms of peak wavelength), the understanding necessary to truly design the complex visual appearance of a photonic CNC film (e.g., vibrancy, color purity, iridescence, sheen) has not yet been achieved.

Fundamentally, the ability of CNCs and ChNCs to form cholesteric structures stems from the properties of the individual particles, namely their composition, morphology and surface functionality. At the next level, their interactions are mediated by their local environment, dictated by factors such as the choice of solvent, their electrostatic interactions and the presence of additives. Beyond this, the evolution of the suspension into the solid state is strongly influenced by both thermodynamic factors (mesophase formation, geometrical surface anchoring, interaction with fields) and kinetic factors (tactoid formation, coalescence and defect dynamics), which combine to produce the final appearance. Lastly, alternative self-assembly conditions beyond the default dish-casting offer new materials and applications and also revealed the key role of the geometry.

Moving forward, further work on CNCs and ChNCs may focus on addressing fundamental scientific questions about their self-assembly mechanisms, develop additional functionality or tackle engineering challenges related to their up-scaling.

### 12.2 Unresolved Scientific Questions

#### 12.2.1 Where Do Crystallite Bundles Originate from?

Recent findings on the role of crystallite aggregates as chiral dopants for the CNC mesophase prompt several further questions.^27,89^ For instance, to what extent are these “bundles” intrinsic to the cellulose source, or a consequence of the production process? What is the optimal morphology of such aggregates, and to what extent can their aggregated state be controlled? Furthermore, understanding whether this conceptualization can be generalized to other colloidal cholesteric mesophases, from the closely related ChNCs reviewed here, to suspensions of amyloid fibrils or fd-viruses, can be useful to gain insight in the chirality transfer mechanisms at play in these systems. Addressing these questions is expected to improve the reproducibility (and thus scalability) of CNC photonic materials, as their self-assembly behavior could then be decoupled from their fabrication and processing history.

#### 12.2.2 Can the Handedness of the Cholesteric Mesophase Be Inverted?

In colloidal suspension, CNCs and ChNCs have only ever been observed to form a left-handed mesophase, which invariably leads to reflection of LCP light in the solid state. However, while natural helicoidal systems are also predominantly left-handed, a subset of the cells in the epicarp of the *Pollia condensata* fruit instead reflect RCP light,^925^ and it has been shown that the handedness of the structure is correlated with the presence of other cell wall components such as hemicelluloses.^926^ As such, it is interesting to understand whether chiral additives can influence the handedness of the CNC mesophase, as this will yield new insights into the origin of chirality in colloidal systems, and offer a simple route to producing CNC photonic films with greater than 50% reflection in a given wavelength range, which is highly desirable for their exploitation as sustainable colorants.

#### 12.2.3 What Are the Mechanisms of Kinetic Arrest?

While the cholesteric self-organization of CNC suspensions prior to kinetic arrest is well-understood, and the role of geometry upon loss of solvent after kinetic arrest has recently been elucidated, the precise mechanisms of kinetic arrest itself are still not completely clear. Greater understanding of kinetic arrest will require consideration of how the mesophase solidifies on the microscale, and how this process can vary depending on the properties of individual CNCs (e.g., surface charge, morphology, functionalization), their formulation (e.g., electrolytes, rheology modifiers, cross-linkers), and the drying history of the suspension (e.g., concentration rate, phase composition and ordering). The challenges here arise from the fact that this process is kinetic in nature, and thus varies over time and across the system, as opposed to being a time-independent, volume-spanning thermodynamic transition (cf. isotropic-cholesteric phase coexistence). As discussed in section 5.4, it is likely that different degrees of freedom become kinetically arrested at different points in time. Deconvoluting these nonergodic processes would require the discrimination of related yet distinct phenomena at different length scales, including CNC aggregation, evolution of the rheological properties of the system, and kinetic arrest of the cholesteric order. Furthermore, the onset of mesophase formation and that of kinetic arrest are not instantaneous and are therefore not necessarily distinguishable or sequential, which can lead to more complex architectures. A better understanding of the kinetic arrest transition as a whole would lead to greater control of the visual appearance of the resulting films: for example, strategies to delay kinetic arrest could extend the self-assembly window (*c*_*b*1_ → *c*_KA_) and lead to improved ordering, while strategies to trigger early kinetic arrest could be exploited to enhance some of the orientational effects discussed in section 4.

#### 12.2.4 Can Structural Color Be Achieved with Other Polysaccharide Nanocrystals?

In this review, we have summarized how nanoscale crystallites of cellulose and chitin can both form a colloidal cholesteric mesophase, which leads to a helicoidal film upon drying. However, these systems offer different strengths and weaknesses arising from the chemistry of the constituent polysaccharides. As such, it would be interesting to understand if the library of polysaccharide nanomaterials for structural color can be expanded. For example, while the self-assembly of ChNCs into a film results in only very weak reflection, visible coloration can be achieved by converting the chitin into chitosan as a postprocess.^538^ This then raises the question as to whether chitosan nanocrystals (which have only recently been reported with high crystallinity and degree of deacetylation^902,927^) could instead be directly assembled into a photonic material.^902^

### 12.3 Outstanding Technical Challenges

#### 12.3.1 The Need for “Greener” CNCs and ChNCs

While natural materials, such as cellulose and chitin, are inherently sustainable in terms of both their renewable supply and biodegradable disposal, their processing into nanomaterials still typically requires strong acids and significant water consumption.^163^ As such, further optimization of production methods is required to reduce their environmental footprint, in terms of minimizing the consumption of reagents, solvents and energy used in conventional hydrolysis processes, and exploring alternative processing methods (e.g., enzymatic hydrolysis, ionic liquids). Furthermore, the valorization of biowaste is an exciting opportunity to implement a circular economy approach to CNC-based materials; however, a greater understanding of what feedstocks can be implemented into existing CNC production methods is required, combined with studies into whether these recycled sources produce CNCs suitable for photonic applications.

#### 12.3.2 Standardized Metrology of Nanocrystals

In contrast to model colloidal systems, the large variation between nanoparticles within a single CNC/ChNC suspension introduces significant complexity when investigating the underlying self-assembly processes, or when validating observations between different laboratories.^193,194,928,929^ As such, population-averaged measurements on simple parameters such as length and width are insufficient to benchmark naturally derived nanocrystals for photonic applications^196^ and thus need to be replaced with more precise and robust morphological measurements at the individual nanoparticle level while also maintaining sufficient throughput so that ensemble trends can still be deconvoluted. As such, while semiautomatic TEM image analysis has been demonstrated to speed up analysis,^197^ the development of a full automation of a suite of particle characterization techniques (potentially exploiting recent advances in machine learning) would unlock the adoption of a fast, reliable and directly comparable particle morphology analysis. Furthermore, this transition needs to be combined with industry-standardized methods of sample preparation and data collection, which together will remove the subjective bias of the human operator. Such protocols would also be of significant benefit in the industrial scale-up of such photonic materials as, for example, the batch-to-batch variation from commercial CNC suppliers can lead to laborious formulation recalibration to maintain production with an invariant optical appearance.

#### 12.3.3 Efficient Self-Assembly for Large-Scale, Continuous Production

Photonic films made from CNCs and ChNCs are usually nonuniform in visual appearance. This inhomogeneity can occur at all length-scales, ranging from the macroscopic accumulation of material at the edges, leading to color gradients, to microscale dispersity in the pitch and alignment of domains. While the optical effects arising from inhomogeneities are sometimes desirable, uniform coloration is usually the target for industrial production to maximize yield and ensure consistent optical performance. While methods such as cocasting with additives or size fractionation can respectively help to alleviate these issues, they are not strategies that can be readily or universally applied.

Finally, the production of such photonic films is constrained by the trade-off between the desire to dry the film quickly (to maximize throughput) and the need to allow sufficient time for self-assembly to occur (to maximize quality).^547^ To date, the drying conditions have usually been kept fixed and not precisely controlled. Instead, it will be imperative to optimize each stage of the drying process individually, such that more time is allowed in the self-assembly window (i.e., *c*_*b*1_ → *c*_KA_) but minimized elsewhere (e.g., *c* > *c*_KA_, where the suspension is arrested and thus its local organization cannot improve further).

### 12.4 Conclusions

To meet the demand for functional materials with tailored properties, the pursuit of optimal performance must be balanced against a consideration of the sustainability of the source materials and its production process. By exploiting the most abundant biopolymer on the planet, cellulose, and replicating the natural assembly processes found within the plant cell, the potential for CNCs to revolutionize the colorant industry (e.g., food, cosmetics) can be enormous. Indeed, the wide accessibility and low cost of CNCs have been major factors in democratizing photonic research, with laboratories across the world contributing to this global effort to promote CNCs as the sustainable optical material of the future and to reveal new functions or applications. While the sourcing of ChNCs from aquacultural waste offers an alternative to reliance on plant-based renewable materials, further breakthroughs are needed to match the optical performance of CNC-based analogues. However, ChNCs offer much greater scope for chemical modification and coassembly, which may unlock their full potential for functional optical composites.

## Supporting Information Available

Further discussion of DLVO theory for charged cylinders, analysis of angle-resolved optical spectroscopy data, experimental methods for optical characterization of CNC photonic films (PDF)
