# Supplementary material for: Structural Color from Cellulose Nanocrystals or Chitin Nanocrystals: Self-Assembly, Optics, and Applications
Source: Chem Rev. 2023 Nov 27;123(23):12595–756. doi: 10.1021/acs.chemrev.2c00836 (PMC10729353; doi:10.1021/acs.chemrev.2c00836)
Supplement: Supplementary file 1 — cr2c00836_si_001.pdf [file cr2c00836_si_001.pdf]

Supporting Information

For

Structural Color from Cellulose Nanocrystals or Chitin Nanocrystals: Self-Assembly, Optics and Applications

*Bruno Frka-Petesic[1,2] * †, Thomas G. Parton[1] †, Camila Honorato-Rios[3], Aurimas Narkevicius[4], Kevin Ballu[1], Qingchen Shen[1], Zihao Lu[1], Yu Ogawa[5], Johannes S. Haataja[6], Benjamin E. Droguet[1], Richard M. Parker[1] *, and Silvia Vignolini[1] *.*

1 Yusuf Hamied Department of Chemistry, University of Cambridge, Lensfield Road, Cambridge, CB2 1EW, United Kingdom.

2 International Institute for Sustainability with Knotted Chiral Meta Matter (WPI-SKCM^2), 1-3-1 Kagamiyama, Hiroshima University, Higashi-Hiroshima, Hiroshima, 739-8526, Japan.

3 Department of Sustainable and Bio-inspired Materials, Max Planck Institute of Colloids and Interfaces, Am Mühlenberg 1, 14476 Potsdam, Germany.

4 B CUBE – Center for Molecular Bioengineering, Technische Universität Dresden, Dresden, Germany.

5 CERMAV-CNRS, CS40700, 38041 Grenoble cedex 9, France.

6 Department of Applied Physics, Aalto University School of Science, P.O. Box 15100, Aalto, Espoo FI-00076, Finland.

* Email: bf284@cam.ac.uk; rmp53@cam.ac.uk; sv319@cam.ac.uk.

† These authors contributed equally.

S1 DLVO theory for charged cylinders

Pair interaction of charged rod-like CNCs

In DLVO theory as initially developed for spherical particles, the mutual orientation of the particles is irrelevant and the pair potential is determined by the distance r between the centers of mass of the particles, or the gap $h = r - R_1 - R_2$ between the particle surfaces, assuming their radii are respectively R_1 and R_2. For rod-like particles however, the interactions strongly depend on their mutual orientation.

Let us define the distance r_{axes} as the shortest distance between the axes of two spherocylinders of radii respectively R_1 and R_2, and lengths $L \gg (R_1, R_2)$ oriented either parallel or perpendicular to one another, and the gap $h = r_{\mathrm{axes}} - R_1 - R_2$.

The pair interaction potential between rod-like particles in these specific directions can then be written as:

$$V_{\mathrm{DLVO}}^{||}(r_{\mathrm{axes}}) = V_{\mathrm{vdW}}^{||}(r_{\mathrm{axes}}) + V_{\mathrm{elec}}^{||}(r_{\mathrm{axes}}) \tag{1}$$

$$V_{\mathrm{DLVO}}^{\perp}(r_{\mathrm{axes}}) = V_{\mathrm{vdW}}^{\perp}(r_{\mathrm{axes}}) + V_{\mathrm{elec}}^{\perp}(r_{\mathrm{axes}}) \tag{2}$$

where the two potentials correspond to parallel and perpendicular configurations, respectively. The attractive (van der Waals) and repulsive (electrostatic) contributions are evaluated in the following sections.

Attractive potential

The attractive van der Waals contribution $V_{\mathrm{vdW}}(r)$ between two spherocylindrical particles of radii R_1 and R_2, spaced from one another by a surface-to-surface distance h, can be approximated by:[1]

$$V_{\mathrm{vdW}}^{||}(h) = -\frac{AL}{24h^{3/2}}\sqrt{\frac{2R_1R_2}{R_1 + R_2}} \tag{3}$$

$$V_{\mathrm{vdW}}^{\perp}(h) = -\frac{A}{6h}\sqrt{R_1R_2} \tag{4}$$

where A is the Hamaker constant, which is specific to the case of the materials of the particles 1 and 2 and the bulk medium in between. For CNCs in water, estimations of the Hamaker constant were estimated to $A = 8 \times 10^{-21}$J (from a film of cellulose II allomorph).[2] While this expression assumes spherocylindrical rods, the radii values relevant for CNCs can be approximated as $R_1 = R_2 \approx \sqrt{WT}/2$, where W is the width of a CNC (typically 10-20 nm for cotton) and T its thickness (typically 5-7 nm for cotton). Note that there are alternative models for interactions between rods, such as that derived by Sparnaay,[3] and later corrected by Buining et al.[4] These models assume that the interior of the particle is made of a homogeneous, isotropic material. For refined geometrical models and anisotropic materials potentially more suitable for CNCs, the interested reader can find refined expressions of van der Waals potentials in this comprehensive handbook.[5]

Repulsive potential

The repulsive electrostatic contribution $V_{\mathrm{elec}}(r)$ between similarly shaped particles is given, under the assumption of large κR, in a 1:1 electrolyte (i.e., made of monovalent ions), by:[4]

$$V_{\mathrm{elec}}^{||}(h) = 64\sqrt{\pi}\, nk_BT\,\gamma^2\, L\frac{\sqrt{\kappa R}}{\kappa^2}\exp(-\kappa h) \tag{5}$$

$$V_{\mathrm{elec}}^{\perp}(h) = 128\pi\, nk_BT\,\gamma^2\,\frac{R}{\kappa^2}\exp(-\kappa h) \tag{6}$$

$$\gamma = \tanh\left(\frac{e\psi_0}{4k_BT}\right) \tag{7}$$

Here, $n = 10^3\, N_A\, I$ is the number density of ions (in #/m^3), where N_A is the Avogadro constant and I the ionic strength (in mol/L), k_B the Boltzmann constant, T the temperature, e the elementary charge and ψ_0 is the surface potential. The Debye length κ^{-1} is defined in section 5.1 of the main text.

S2 Analysis of Experimental Data from Angle-Resolved Optical Spectroscopy

Specular Scan

The specular scan is sensitive to the domains pointing normal to the sample surface, and the wavelength of its highest reflection will follow Bragg's law for the pitch of the domains with no tilt:

$$l\,\lambda_m = p'(0)\sqrt{n_{\mathrm{Ch}}^2 - n_{\mathrm{air}}^2\sin^2\theta^{\mathrm{ext}}} \tag{8}$$

Where $p'(0)$ is the pitch of local domains with tilt $\beta' = 0$. Here, l is an integer corresponding to the diffraction order, with $l = 1$ for the main diffraction peak. As explained in section 7.3.5, higher orders are expected for non-normal incidence ($\theta^{\mathrm{ext}} \neq 0$), and even at normal incidence if the domains are distorted (see section 7.3.6).

Scattering Scan

The reflected wavelength measured in a scattering scan, $\lambda_m(\theta_o^{\mathrm{ext}})$, is given using a parametric expression of $\lambda_m(\beta')$ and $\theta_o^{\mathrm{ext}}(\beta')$ derived from the Fergason's law as:

$$l\,\lambda_m = n_{Ch}\, p'(\beta')\cos\theta_{\mathrm{loc}} \tag{9}$$

$$\theta_o^{\mathrm{ext}} = \arcsin[n_{Ch}\sin(\theta_{\mathrm{loc}} + \beta')] \tag{10}$$

$$\theta_{\mathrm{loc}} = \beta' + \arcsin\left[n_{Ch}^{-1}\sin\left(\theta_i^{\mathrm{ext}}\right)\right] \tag{11}$$

where the pitch variation $p'(\beta')$ has to be accounted for using the expressions derived in section 6.1.

Tilt Scan

Finally, the reflected wavelength measured in a tilt scan, $\lambda_m(\theta_s)$, is given as:

$$l\,\lambda_m = n_{Ch}\,p'(\beta')\cos\theta_{\text{loc}} \tag{12}$$

$$\theta_{\text{loc}} = \frac{1}{2}\sin^{-1}\left[n_{Ch}^{-1}\sin(\Delta\theta^{\text{ext}} + \theta_s)\right] + \frac{1}{2}\sin^{-1}\left[n_{Ch}^{-1}\sin(\Delta\theta^{\text{ext}} - \theta_s)\right] \tag{13}$$

$$\beta' = \frac{1}{2}\sin^{-1}\left[n_{Ch}^{-1}\sin(\Delta\theta^{\text{ext}} + \theta_s)\right] - \frac{1}{2}\sin^{-1}\left[n_{Ch}^{-1}\sin(\Delta\theta^{\text{ext}} - \theta_s)\right] \tag{14}$$

where, again, the pitch variation $p'(\beta')$ has to be accounted for.

Dispersion of Film Flakes in a Cylindrical Vial

For a suspension of CNC flakes freely floating inside a cylindrical vial, the tilt angle becomes irrelevant by symmetry, and the sample characterization is made by scanning the detector angle. The normal incidence through the air-vial interface justifies discarding the Snell's law correction there, but the refractive index mismatch between the fluid and the flakes can still require Snell's law adjustment. The optical response is then well described by an adapted Fergason's law, where the local specular response of the films is dominating their individual off-specular contributions:

$$\lambda_m = p'(0)\sqrt{n_{Ch}^2 - n_{\text{liquid}}^2\sin^2\theta_{\text{loc}}} \tag{15}$$

$$\theta_{\text{loc}} = \beta' = \Delta\theta^{\text{ext}} \tag{16}$$

Where p is the pitch in suspension (expected to be a constant).

Cholesteric Suspensions in a Cylindrical Vial

For a cholesteric CNC suspension in a cylindrical vial, the sample characterization is made by scanning the detector angle, and the normal incidence through the air-vial and the vial-suspension interfaces allows for discarding Snell's law correction. The optical response is then given by Bragg's law:

$$\lambda_m = n_{Ch}\,p\cos\theta_{\text{loc}} \tag{17}$$

$$\theta_{\text{loc}} = \beta' = \Delta\theta^{\text{ext}} \tag{18}$$

Where p is the pitch in suspension (expected to be a constant).

S3 Experimental Methods for Optical Characterization

This section provides the experimental and instrumental details used for the optical characterization of the example CNC film provided throughout the section 7.

S3.1 Angle-Resolved Optical Spectroscopy

Angle-resolved optical spectroscopy measurements were performed using a custom goniometer setup, which allowed free rotation of the sample and detector relative to the fixed illumination direction.[6] A broadband xenon lamp (HPX2000, Ocean Optics) was coupled to a reflective collimator (RC08SMA-F01, Thorlabs) via a 200 µm fiber-optic cable (FC-UV200-2, Avantes) and used to illuminate the sample with a circular spot of diameter approx. 2mm. Detection was performed using a second reflective collimator on the rotating detection arm, which was coupled to a UV-vis spectrometer (AvaSpec-HS2048, Avantes) via a 600 µm fiber-optic cable (FC-UV600-2, Avantes). The recorded light intensity was normalized to the specular reflection from a white Lambertian diffuser (WS-2, Avantes) measured at an angle of incidence of 5°. To accurately capture the scattered light across a wide range of intensities, the spectrometer integration time was adjusted automatically using a high dynamic range (HDR) method with integration times from 1.05 ms to 2000 ms.

S3.2 UV-vis Transmission Spectroscopy

Measurements were performed using the goniometer setup described above, with the detector facing the illumination direction. In this case the sample was illuminated using a 1000 µm diameter fiber-optic cable (FC-UV-1000-2-SR, Avantes) to provide a larger spot size (approx. 15 mm). As the spot was larger than the aperture of the detection collimator, the directly transmitted light was focused using a plano-convex lens (LA1027-A, Thorlabs). To prevent saturation of the spectrometer, the transmitted intensity was reduced using a neutral density filter (ND20A, Thorlabs, optical density 2.0). Spectra are referenced to light transmission with no sample in the beam path.

S3.3 Double-ended Reflection Probe Spectroscopy

Measurements were performed using a double-ended reflection probe (R200-7-SR, Ocean Optics) as illustrated in **Figure 40** of the main text. A broadband halogen lamp (SLS201L/M, Thorlabs) was used to illuminate the sample via six fiber cores arranged hexagonally, with the reflected light collected by a seventh central fiber and relayed to the spectrometer. Spectra were collected with a distance of 15 mm between the sample and probe, and reference to reflection from a silver mirror (PF10-03-P01, Thorlabs) at 15 mm.

S3.4 Optical Spectroscopy using an Integrating Sphere

For total reflectance and transmittance measurements, the sample was mounted onto an integrating sphere (LabSphere) as illustrated in **Figure 40** of the main text. The sample was illuminated with a broadband xenon lamp coupled to a reflective collimator using a 200 µm fiber-optic cable, with a spot diameter of approx. 5 mm. Diffuse scattered light from the sample was collected using a 1000 µm fiber-optic cable connected to a spectrometer. Total reflectance spectra were referenced to a Lambertian white diffuser, while total transmittance spectra were referenced to illumination without a sample.

S3.5 Polarized Optical Microscopy

POM was performed using a Zeiss Axioscope upright optical microscope in reflection and transmission modes, as illustrated in **Figure 46** of the main text. Three objective lenses were

employed: Zeiss EC Epiplan Neofluar 5x/0.13, EC Epiplan Apochromat 10x/0.30 and EC Epiplan Apochromat 20x/0.60. Images were acquired using a digital CMOS camera (UI-3580LE, IDS), with the white balance set using a white Lambertian diffuser (WS-2, Avantes). Image magnification was verified using a microscope slide scale bar.

For unpolarized (UP) imaging, no polarizer or analyzer were used. For imaging between parallel polarizers (PP) and crossed polarizers (XP), the polarizer and analyzer were both broadband wire-grid linear polarizers (Thorlabs, WP25M-UB) arranged with polarizing axes parallel or perpendicular respectively. LCP and RCP imaging were performed with no polarizer in the light path, and an analyzer consisting of a superachromatic quarter-wave plate (B-Halle) followed by a linear polarizer arranged with the wave plate fast axis at 45° or 135° to the polarizer respectively.

Optical micro-spectroscopy was performed using the microscope setup in **Figure 46** of the main text. A beamsplitter was used to split collected light between the microscope camera and a 100 μm diameter fiber-optic cable mounted in confocal configuration, allowing spectra to be obtained from a given spatial position and for a given polarization state. Reflection spectra were normalized to a silver mirror (Thorlabs, PF10-03-P01). UP spectra were normalized to a mirror illuminated in UP configuration. PP and XP spectra normalized to a mirror illuminated in PP configuration. LCP and RCP spectra were normalized to a mirror illuminated in the respective configurations, with separate references for LCP and RCP.

S4 References for the Supporting Information

(1) Israelachvili, J. N. *Intermolecular and Surface Forces (Third Edition)*; Academic Press: San Diego, 2011.

(2) Bergström, L.; Stemme, S.; Dahlfors, T.; Arwin, H.; Ödberg, L. Spectroscopic Ellipsometry Characterisation and Estimation of the Hamaker Constant of Cellulose. *Cellulose* **1999**, *6* (1), 1–13. https://doi.org/10.1023/A:1009250111253.

(3) Sparnaay, M. J. The Interaction between Two Cylinder Shaped Colloidal Particles. *Recueil des Travaux Chimiques des Pays-Bas* **1959**, *78* (9), 680–709. https://doi.org/10.1002/recl.19590780908.

(4) Buining, P. A.; Philipse, A. P.; Lekkerkerker, H. N. W. Phase Behavior of Aqueous Dispersions of Colloidal Boehmite Rods. *Langmuir* **1994**, *10* (7), 2106–2114. https://doi.org/10.1021/la00019a016.

(5) Parsegian, V. A. *Van Der Waals Forces, A Handbook for Biologists, Chemists, Engineers, and Physicists*; Cambridge University Press: Cambridge, New York, Melbourne, Madrid, Cape Town, Singapore, São Paulo, 2006.

(6) Vignolini, S.; Moyroud, E.; Glover, B. J.; Steiner, U. Analysing Photonic Structures in Plants. *Journal of the Royal Society, Interface* **2013**, *10* (87), 20130394. https://doi.org/10.1098/rsif.2013.0394.
